# Proteome Adaptation to High Temperatures in the Ectothermic Hydrothermal Vent Pompeii Worm

**DOI:** 10.1371/journal.pone.0031150

**Published:** 2012-02-10

**Authors:** Didier Jollivet, Jean Mary, Nicolas Gagnière, Arnaud Tanguy, Eric Fontanillas, Isabelle Boutet, Stéphane Hourdez, Béatrice Segurens, Jean Weissenbach, Olivier Poch, Odile Lecompte

**Affiliations:** 1 Adaptation & Diversité en Milieu Marin, CNRS UMR 7144, Roscoff, France; 2 Station Biologique de Roscoff, UPMC Université Paris 6, Roscoff, France; 3 Department of Structural Biology and Genomics, IGBMC, Illkirsh, France; 4 Genoscope – Centre National de Séquençage, Evry, France; University of Wyoming, United States of America

## Abstract

Taking advantage of the massive genome sequencing effort made on thermophilic prokaryotes, thermal adaptation has been extensively studied by analysing amino acid replacements and codon usage in these unicellular organisms. In most cases, adaptation to thermophily is associated with greater residue hydrophobicity and more charged residues. Both of these characteristics are positively correlated with the optimal growth temperature of prokaryotes. In contrast, little information has been collected on the molecular ‘adaptive’ strategy of thermophilic eukaryotes. The Pompeii worm *A. pompejana*, whose transcriptome has recently been sequenced, is currently considered as the most thermotolerant eukaryote on Earth, withstanding the greatest thermal and chemical ranges known. We investigated the amino-acid composition bias of ribosomal proteins in the Pompeii worm when compared to other lophotrochozoans and checked for putative adaptive changes during the course of evolution using codon-based Maximum likelihood analyses. We then provided a comparative analysis of codon usage and amino-acid replacements from a greater set of orthologous genes between the Pompeii worm and *Paralvinella grasslei*, one of its closest relatives living in a much cooler habitat. Analyses reveal that both species display the same high GC-biased codon usage and amino-acid patterns favoring both positively-charged residues and protein hydrophobicity. These patterns may be indicative of an ancestral adaptation to the deep sea and/or thermophily. In addition, the Pompeii worm displays a set of amino-acid change patterns that may explain its greater thermotolerance, with a significant increase in Tyr, Lys and Ala against Val, Met and Gly. Present results indicate that, together with a high content in charged residues, greater proportion of smaller aliphatic residues, and especially alanine, may be a different path for metazoans to face relatively ‘high’ temperatures and thus a novelty in thermophilic metazoans.

## Introduction

Adaptation to high temperatures is a complex evolutive process that can involve modifications of the intrinsic stability of proteins, and/or interactions with other proteins (e.g. chaperones) that stabilize or help the re-folding of the partner. Thermal adaptation has been extensively studied in hyperthermophilic microorganisms (archaea and bacteria) by analysing amino acid replacements in translated proteomes and GC-content in coding sequences [1]–[3]. In most cases, adaptation to thermophily is associated with great modifications of genomes in terms of codon usage [4], high GC content in structural RNAs [5] and purine content in mRNAs [6], but also relies on lower residue volume, higher hydrophobicity, more charged amino acids (especially Glu, Arg, and Lys), along with a decrease in uncharged polar residues in the proteome [1], [7]–[8]. However, two distinct evolutionary strategies seem to have emerged in prokaryotes depending on whether the thermophilic character appeared early during the course of evolution (e.g. *Pyrococcus furiosus*) or has been more recently derived during the colonization of extreme environments (e.g. *Thermotoga maritima*). As previously proposed, the choice of a particular strategy depends on the evolutionary history of an organism [3]. Proteins from organisms that evolved early at high temperatures are significantly more compact and more hydrophobic than their mesophilic counterparts. Alternatively, organisms that recently colonized hot environments evolved under a rather “key residue-based” mechanism of thermostability in which a few charged amino acids replacements or amino acid deletions favored hydrogen bonds and inter- and/or intra-subunit electrostatic interactions [9], decreased length of surface loops [10], or solely affected specific chaperones/structural proteins in order to improve their efficiency [11]. Although the molecular basis of eukaryote thermophily is far less understood, subtle similar amino acid replacements are likely to occur. Theoretical models indeed showed that the proportion of some amino acids and protein stability are linearly related over a wide range of temperatures leading to small adjustments of the average amino acid frequencies [12]. Because most thermophilic eukaryotes are only able to live at the lower thermal range of thermophilic bacteria (*i.e.* 40–60°C), the level of protein compactness and high GC genome content are probably less relevant. However, such strategies may still hold depending on how ancestral the thermophilic character is and how severe the thermal selection is.

To date, the only empirical study that attempted to explore potential adaptation of eukaryotes to temperature used an indirect approach based on correlations between the amino acid usage and the ability of vertebrates to regulate their internal temperature (ectothermic *vs* endothermic species) [13]. Although controlling for the phylogenetic non independence of traits is possible in prokaryotes using an independent contrasts approach, such methods require pairs of thermophilic/non-thermophilic sister taxa throughout the phylogeny [14]–[15]: a situation never met in vertebrates as endothermy is fully derived in the species phylogeny. To this extent, alvinellid worms represent an ideal biological model to study thermophily as this polychaete family displays several species, some of which being thermophilic and other mesophilic. In addition, fossil tube imprintings similar to those of present-day alvinellid suggest that their ancestors inhabited vent chimneys since at least the Devonian period [16]–[17] and, as a result, had enough time to develop molecular adaptations to high temperatures. They are thus particularly relevant to study the evolution of thermal adaptation and determine its role in speciation as they invaded nearly all vent microhabitats from the hottest part of the environment [18]–[20] to more diffuse and cooler habitats [18], [20]. As an example, on Juan de Fuca vent chimneys, temperature preferences of two alvinellid species differ markedly, with experimental temperature optimum of 40–50°C for *Paralvinella sulfincola*, while *P. palmiformis* consistently avoids temperatures above 35°C [20]. Taking into account these differences, an allozyme-derived phylogenetic study proposed that thermal habitats could have shaped present patterns of the species distribution prior to their geographic separation associated with tectonic plate dynamics [21].

The hydrothermal vent worm *Alvinella pompejana* (Pompeii worm) is currently considered as the most thermotolerant eukaryote on Earth, withstanding the widest thermal and chemical ranges known [22]–[23], as well as the highest concentrations of potentially toxic compounds [19]. It inhabits chimney walls on the East Pacific Rise [19], in contrast to its sympatric congener *Paralvinella grasslei*, which lives in a much cooler diffuse venting habitat [18]. To survive, it developed numerous adaptations at the structural and the physiological levels, in particular thermal stability of some proteins and protein complexes such as collagens [24], mitochondrial respiratory chain proteins [25], hemoglobins [26] and cytoplasmic proteins [21], [27]–[28], with, in some cases, putative thermal adaptive protein polymorphisms [29]. Structural studies on its first overexpressed proteins showed that these proteins had a higher stability than their homeotherm homologs [27]–[28] and confirmed the worm was thermophilic. Recently, the nearly complete Sanger sequencing of the Pompeii worm's transcriptome (NCBI unigene database, [30]) coupled together with an EST sequencing of a closely-related species *P. grasslei* (10 000 reads) allowed us to perform the first comparative sequence analysis in order to trace back putative ‘adaptive’ mutations associated with eukaryote thermophily. We first tried to detect an amino-acid signature of metazoans to ‘high’ temperatures using the well-conserved ribosomal protein genes within the Lochotrophozoan group. Then, a more detailed comparison of a set of 335 orthologous genes was performed between the two worms to study the codon usage and depict putative codon or residue biases attributable to thermophily. This work represents therefore the first alternative to study the molecular adaptation of eukaryotes towards thermophily, from a more restrictive phylogenetic context using a pair of sister species inhabiting very contrasted thermal environments.

## Results

### Amino acid composition of ribosomal proteins in *Alvinella* and its evolution within lophotrochozoans

**Figure 1:**
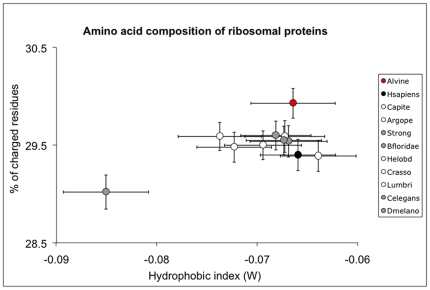
Distribution of eukaryotic species according to the hydrophobic and charged residues signature of ribosomal proteins. Amino acid composition of ribosomal proteins obtained using a concatenated set of 48 primary orthologous sequences (5191 site patterns) using lophotrochozoan and model species. Alvine (red circle), lophotrochozoan species (Capite, Helobd, Lumbri, Argope, Crasso: white circles), model organisms (Strong, Bfloridae, Dmelano, Celegans: grey circles), and *Homo sapiens* (Hsapiens: black circle). The X-axis represents the hydrophobic index W and the Y-axis corresponds to the percentage of charged residues. Bars are standard deviations estimated from 100 re-arrangements (bootstrap) of the dataset. For species name abbreviations, see Materials and Methods.

**Figure 2:**
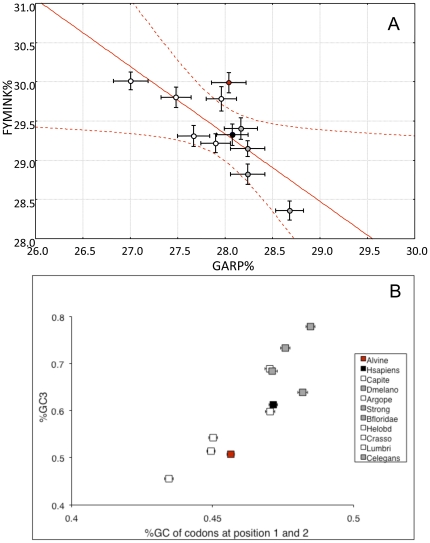
Distribution of eukaryotic species according to their expected GC-content background. (A) Linear regression and confidence interval (99%) of GARP and FYMINK residues and (B) relationship between GC_3_ and GC_1+2_ content for the same species as in Figure 1 (*A. pompejana* = red circle, lophotrochozoan species = white circles, model species = grey circles). Bars represent standard deviations estimated from 100 re-arrangements (bootstrap) of the dataset.

For lophotrochozoans, over the 8200 codons examined from ribosomal protein genes, amino-acid frequencies were almost the same among taxa with the noticeable exception of the nematod *C. elegans* and the polychaete *A. pompejana*. These differences are summarized in Figure 1 as a biplot of the hydrophobic index W and the percentage of charged residues. *A. pompejana* clearly has the highest proportion of charged amino acids (nearly 30%) while *C. elegans* exhibited the lowest values for both parameters. Because excess in lysine may be biased by the overall GC-content, the relationship between AT-rich (FYMINK residues) and GC-rich codons (GARP residues) was examined. *A. pompejana* was the only outlier of this negative linear correlation (Figure 2A: N = 11, *F* = 11.653, r^2^ = 0.564, p-value = 0.0077) and showed a significant excess of Arg and Pro when compared to their expected residuals (see Figure S1). Overall, most amino-acid residues indeed displayed either a positive (A, E, G, L, R, V) or a negative (C, D, K, I, T) relationship with the GC_3_ content. However, in *A. pompejana*, Arg, Pro, and Tyr, on one hand, exhibited higher than expected frequencies, and Gly and Phe, on the other hand, exhibited a lower frequency than expected when compared to their genome-biased expectations (Figure S1). The linear relationship between GC_3_ and GC_1+2_ contents (Figure 2B) clearly indicated that the Pompeii worm displayed an AT-rich genome similar to other lophotrochozoans (*e.g.* bivalves).

**Figure 3:**
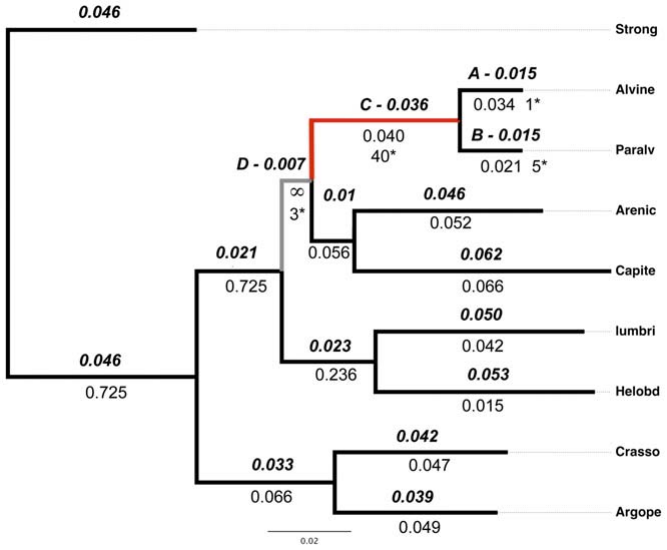
Maximum likelihood d_N_ tree of lophotrochozoan invertebrates, including alvinellids and selective pressures among branches. The tree was obtained from a concatenated set of ribosomal protein orthologous sequences using the ProtML programme of PHYLIP with d_N_ branches subsequently optimized by fitting the codon dataset on the Goldman & Yang (1994) codon substitution model [62] using the free-ω ratio model implemented in CodeML. Branch length (above the branch), and ω (below the branch) obtained from the free-ratio M_1_ model together with the number of positive codon sites with a BEB p-value>0.90 obtained from the M_2A_ model.

Phylogenetic reconstructions were in good agreement with previous phylogenetic analyses on annelids [31]. The best tree (Figure 3) was therefore used as a reference tree for all d_N_/d_S_ analyses performed using CodeML. Results are shown on this tree (Figure 3) and in Table 1. The fit of the branch model M_1_ (ω free to vary among branches) was significantly better than that of model M_0_ (one ω for all branches) to explain substitutions patterns between lochotrophozoans in the codon alignment of ribosomal protein genes (Dataset S1). From the ML d_N_ tree, only one internal branch leading to polychaetes provided a ω value close to infinity but results clearly indicated that both alvinellid lineages are under strong purifying selection when compared to most other lineages. A nested branch-site model (M_2A_ in which site-specific ω in the foreground lineage are partitioned into three ω classes: ω<1, ω = 1 and ω>1) and the site model M_1A_ (‘nearly neutral model’ in which ω falls in only 2 categories: ω<1 and ω = 1) were subsequently tested alternatively using the terminal branches *A. pompejana* (branch A), *P. grasslei* (branch B), as foreground lineages. The positive selection model M_2A_ was significantly better than the ‘nearly neutral’ model M_1A_ for *P. grasslei* (branch B) with only one positive change but not for *A. pompejana* (branch A) when used as foreground lineage. This suggests that excesses in hydrophobic and positively-charged residues are not recently-derived in the thermophilic worm *A. pompejana* but rather was already present in the common ancestor.

**Table 1:** Parameter estimates and Log-likelihood values under models of variable ω ratios among branches and sites.

Model	*p*	Parameter	*lnL*	*Significance*	Positive sites
Branch models					
One ratio M_0_ model	18	κ = 1.066	−111402.36		Null Branch model
		ω = 0.048			site pattern = 5191
Free ratio M_1_ model	33	κ = 1.038	−111112.12	*df* = 15	
		ω(#D) = ∞		δ = 580.5***	
		ω(#C) = 0.039			
Two-ratio M_2_ model	20	κ = 1.063	−111376.07	*df* = 2	
* with branches C & D		ω(#D) = ∞		δ = 52.6***	
		ω(#C) = 0.048			
		ω(tree) = 0.047			
Two-ratio M_2_ model	19	κ = 1.066	−111402.17	*df* = 1	
* with branch C (Alvi)		ω(#C) = 0.051		δ = 0.38^NS^	
		ω(tree) = 0.048			
Site models					
Nearly-neutral M_1A_	19	κ = 1.134	−109947.75	K = 2	Null Branch-site model
		*p*_0_ = 0.926			
		ω_0_ = 0.040			
		*p*_1_ = 0.074			
		ω_1_ = 1			
Branch-site models					
M_2A_ model	21	κ = 1.132	−109922.22	K = 4	37 sites
* branch D (Poly)		*p*_0_ = 0.885, ω_0_ = 0.040		*df* = 2	1 with BEB>0.95
		*p*_1_ = 0.070, ω_1_ = 1		δ = 51.04***	
		*p*_2(NEB)_ = 0.041, ω_2_ = ∞			
		*p*_2(BEB)_ = 0.003, ω_2_ = ∞			
M_2A_ model	21	κ = 1.133	−109866.50	K = 4	68 sites
* branch C (Alvi)		*p*_0_ = 0.914, ω_0_ = 0.039		*df* = 2	29 with BEB>0.95
		*p*_1_ = 0.071, ω_1_ = 1		δ = 162.5***	
		*p*_2(NEB)_ = 0.014, ω_2_ = 14.3			
		*p*_2(BEB)_ = 0.001, ω_2_ = 14.3			
M_2A_ model	21	κ = 1.134	−109945.54	K = 4	14 sites
* branch A		*p*_0_ = 0.926, ω_0_ = 0.040		*df* = 2	1 with BEB>0.95
		*p*_1_ = 0.074, ω_1_ = 1		δ = 4.42^NS^	H→S
		*p*_2(NEB)_ = 0.0004, ω_2_ = 106.9			
		*p*_2(BEB)_ = 0.0000, ω_2_ = 106.9			
M_2A_ model	21	κ = 1.135	−109944.44	K = 4	10 sites
* branch *B*		*p*_0_ = 0.925, ω_0_ = 0.040		*df* = 2	1 with BEB>0.95
		*p*_1_ = 0.074, ω_1_ = 1		δ = 6.62*	K→L
		*p*_2(NEB)_ = 0.0010, ω_2_ = 91.9			
		*p*_2(BEB)_ = 0.0001, ω_2_ = 91.9			

Comparisons across neutral and selective models of codon replacements were done with the set of concatenated genes encoding ribosomal proteins in lophotrochozoans using different foreground internal branch/lineages as a target for assessing codon classes (n_codon = 5965, ns = 9) with the following Best user tree: (((((*Alvine*,*Paralv*),(*Capite*,*Areni*)),(*Helobd*,*Lumbri*)),(*Crasso*,*Argope*)),*Strong*); Positive sites retained with BEB p-value>0.95. ‘Poly’ corresponds to the branch leading to Polychaeta (branch #D) and ‘Alvi’ to the branch leading to Alvinellidae (branch #C). Model parameters: κ = transition/transversion kappa ratio, ω = d_N_/d_S_ ratio estimated for either the tree or specific foreground branches, K = number of ω categories, *p* = proportion of sites in each ω category, *df* = degrees of freedom.

**Figure 4:**
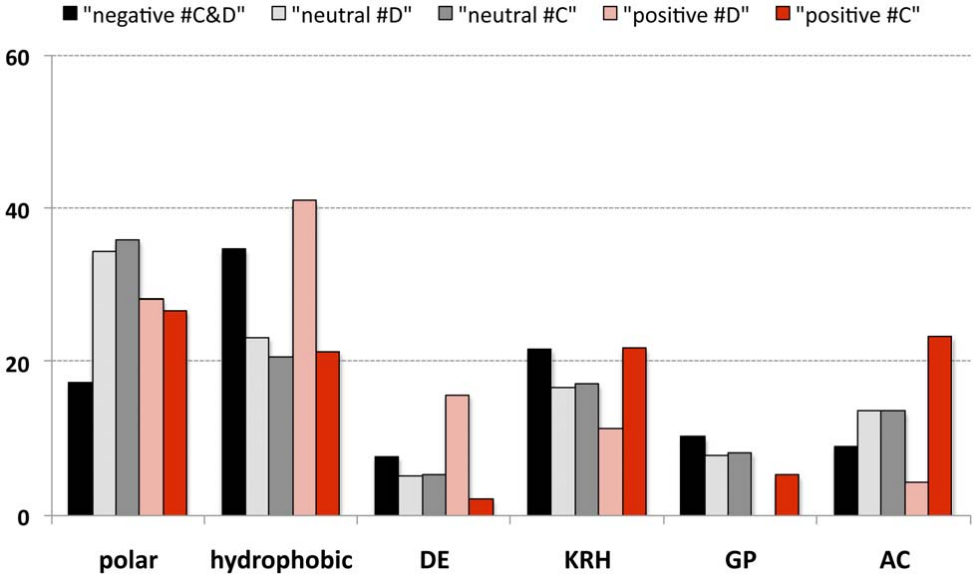
Evolution of amino acid replacements within branches leading to the polychaete and alvinellid lineages. Bars represent the proportions of polar, hydrophobic, charged and small residues found in the 3 ω categories when applying the selective branch-site model onto the codon dataset with the user tree shown in Figure 3. ω≪1 (black bar), ω = 1 (grey bars) and ω>1 (red bars) classes. Light- and dark-red colored bars represent positively selected sites in the internal branches leading to the polychaete (#D) and alvinellid (#C) lineages, respectively and light- and dark-grey colored bars represent sites behaving neutrally within the same branches.

We then compared the nested models M_1A_ and M_2A_ in the foreground branches leading to the Alvinellidae (branch C) and the polychaete (branch D) lineages. For the long branch leading to Alvinellidae (branch C), the M_2A_ model was significantly better than the ‘nearly’ neutral one, with a high number of codon sites (68) found into the ω>1 category, among which 29 had a Bayesian Empirical Bayesian (BEB) probability greater than 0.95 and 40 above 0.90. Looking at these replacements showed that residues changed for more hydrophobic ones, with a noticeable increase of cysteine residues. In the branch leading to the polychaete lineage for which ω = ∞ (branch D), the M_2A_ model was also significantly better than the “nearly” neutral one but with only 37 codon sites positioned in the ω>1 category out of which only three had a BEB probability greater than 0.90. No trend was detected towards a more hydrophobic signature with about the same amount of replacements in both directions. The only noticeable observation was that negatively-charged replacements were all directional from Glu to Asp and nearly all positively-charged ones from Lys to Arg. Figure 4 summarizes the proportions of residues placed into each ω categories with p>0.5 according to their residue attribute (amino acid classes). When assigning an equal weight to each amino acid class, most positive replacements accumulated Ala, Cys, Ser and positively charged residues in the alvinellid lineage. In addition, the 168 alvinellid-specific amino acid replacements found over this branch displayed a positive trend towards a greater hydrophobicity with a global loss of charged residues toward hydrophobic residues (67%) and a strong conversion of polar residues to hydrophobic ones (77%). According to the Sweet & Eisenberg's OMH scale [32], a closer look at replacements showed that residues tended to be more hydrophobic (eg. N→T) within the polar category and more hydrophilic (eg. V→A) within the hydrophobic category.

Because Cys residues seemed to accumulate positively in the branch leading to the alvinellid lineage, they may represent another mechanism of thermal stabilization for extracellular proteins by creating disulfide bonds or, alternatively a mean of detoxification in the animal's tissue as free Cys residues were found to be under positive selection in globins of polychaetes inhabiting reduced environments [33]. As the number of disulfide bonds appears to be anti-correlated with the aliphatic index of proteins [34], this assumption was tested using the proportion of Cys residues as a proxy. No relationship could be detected from a set of 150 complete orthologous proteins ranging from 60 to 1000 amino acids but the additional Cys residues were mostly found in small proteins (Figure S2).

### A similar codon usage with GC-terminated ‘optimal codons’ between *Alvinella* and *Paralvinella*

**Figure 5:**
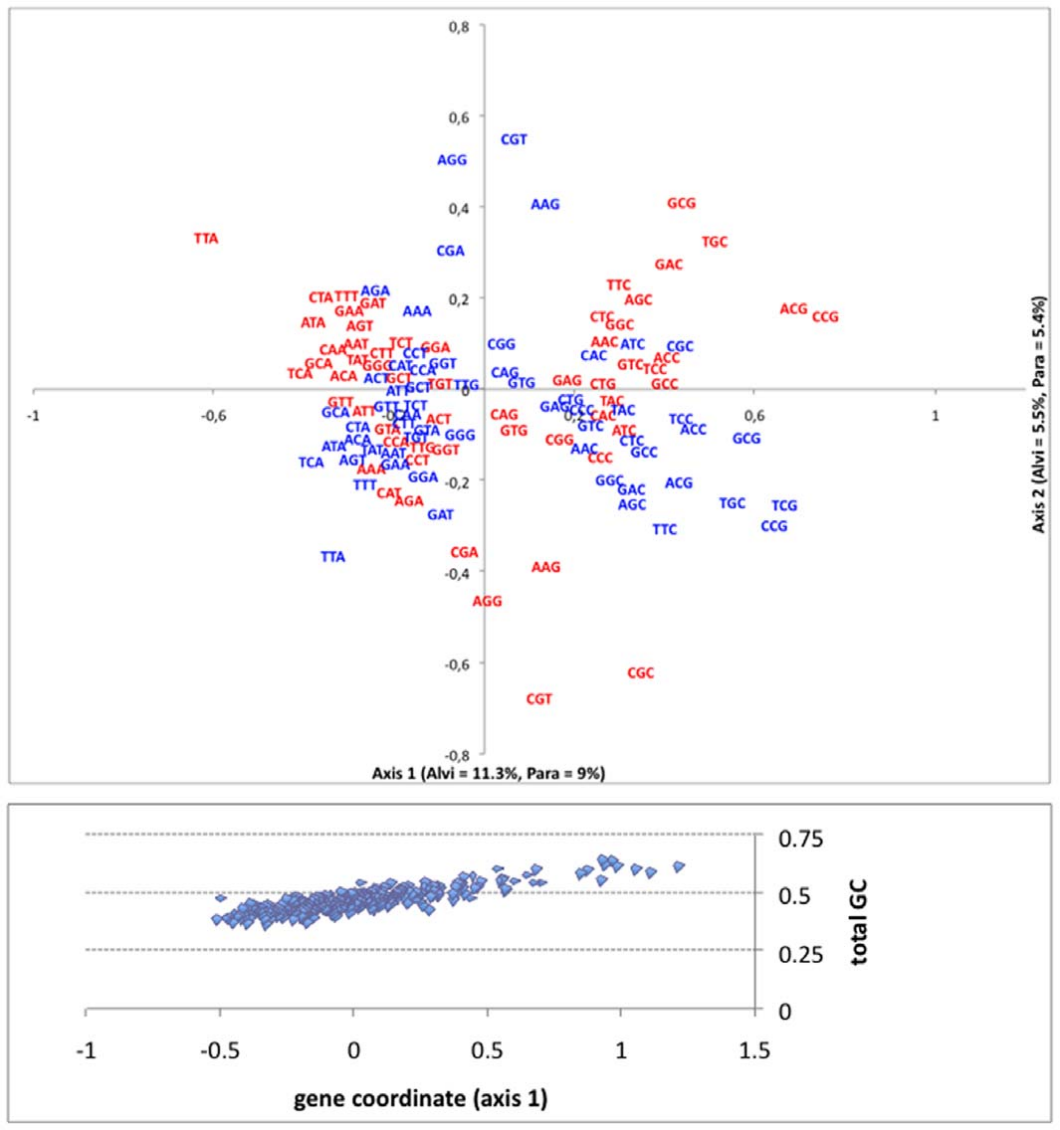
Correspondance analysis (CA) of codon usage in alvinellid polychaetes. CA was performed using Codon W software from a series of 335 orthologous sequences between *A. pompejana* and *P. grasslei*. A: Distribution of codons for both species (red = *A. pompejana* and blue = *P. grasslei*) obtained from two independant runs and displayed in mirror (sign inversion) along axis 2 for greater clarity. The graph shows a clear separation of GC-terminated and AT-terminated codons along axis 1. B: Distribution of genes along the CA axis 1 according to their total GC content.

The correspondance analysis of the codon usage from both alvinellid species showed a clear separation of GC- and AT-terminated codons on axis 1 (Figure 5) with a complete superimposition of the 2 codon clusters that correspond to the two species. This separation was clearly correlated with the GC_3_ content of the genes. This result conforms with the mutational bias hypothesis in which a fraction of genes are highly GC-biased. The 24 preferred (Fop) codons detected were all GC-terminated. Looking at the gene annotation according to gene coordinates on axis 1 showed that GC_3_-rich genes are mainly made of housekeeping, hemoglobin and stress-response genes. On the opposite, genes with low GC_3_ mostly corresponded to mitochondrial protein and signal genes, as well as genes involved in the proteasome. Axis 2 further separated the ribosomal-protein and histone genes from other genes with a clear optimal use of the arginine codons probably reflecting the KR enrichment in this group of proteins. Examining the distribution of the effective number of codons (Nc) against GC_3_ also provided information about the importance of the mutational bias in alvinellid worms with a greater number of biased genes in *A. pompejana* when compared to *P. grasslei* (Figure S3). The codon usage was however quite similar with the same putative Fop codons between the two species, and the distribution of Nc was not significantly different (on average Nc_alvi = 48.4±7.7 *vs* Nc_Para = 49.9±7.0) although slightly down-shifted in the case of the Pompeii worm when GC_3_ increases.

### Evolution of amino-acid composition towards positively-charged and alanine residues in *A. pompejana*

**Figure 6:**
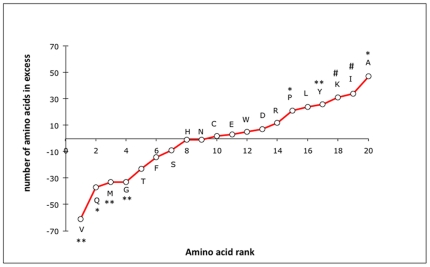
Observed number of residue changes between the two alvinellid species using 335 orthologous genes. Differences in the number of residues are ranked among the 20 amino-acid classes using the 2844 oriented (*Paralvinella*→*Alvinella*) amino acid replacements between the 2 alvinellid species. Binomial tests were performed between observed amino acid counts and their expected values under the assumption of no directional replacements: # = p<0.10, * = p<0.05, and ** = p<0.01.

**Figure 7:**
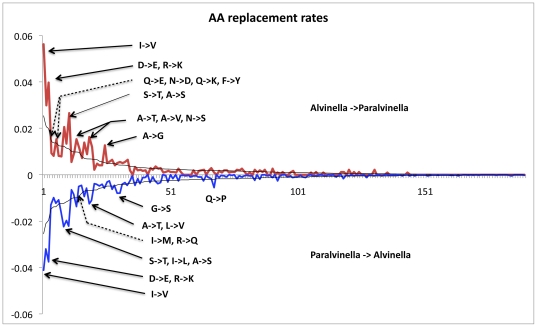
Observed and expected distributions of oriented amino-acid substitutions between *P. grasslei* and *A. pompejana*. Detection of amino acid substitutions that significantly depart from the neutral expectations of the WAG matrix. Blue and red lines correspond to the observed directional frequencies of residue replacements (eg. A→G and G→A) observed between the two alvinellid species. The black lines correspond to the expected directional frequencies of amino acid substitutions obtained using the WAG matrix multiplied by amino-acid frequencies typifying the two alvinellid worms. Arrows show ‘outlier’ substitutions that significantly depart from neutral evolution using the Normal distribution of differences at a 5% threshold.

The comparison of 335 orthologous proteins between the two species yielded a total of 2844 non-synonymous changes out of 40338 amino acid positions (*i.e.* overall protein divergence of 7.05%). This set of substitutions was examined from both directions in order to detect preferred residue changes. Results are presented in Figure 6 and showed a significant excess of substitutions favoring Ala, Ile, Lys, Tyr and Pro together with a significant deficit of substitutions favoring Val, Met, Gly and Gln in *A. pompejana* (one-tail binomial test, p<0.05). The strong decrease of Gln and the increase of Lys led to the global increase of the E+K/Q+H ratio [8] from 1.7 in *P. grasslei* to 2.3 in *A. pompejana*. Among aliphatic residues, amino-acid changes in the Pompeii worm were mostly oriented from Met to Leu or Ile and from Val to Ala (*i.e.* a preference toward smaller residues). Observed distributions of amino-acid substitutions were also compared to their neutral expectations as estimated from the WAG matrix multiplied by the amino-acid frequencies of the worms (Figure 7). This allowed us to examine more carefully both the intensity and asymmetry of mutations between the two alvinellid worms. The difference between observed and expected distributions (both directions) was highly significant (*Chi*2 test, p<0.0001). When looking at the oriented residue replacements between species, DE, RK, AT, and ST were significantly more frequent than neutral expectations in both directions (outliers falling in the distribution tail at a 5% level), indicating that mutations involving charged residues and small amino acids (Ser, Thr, Ala, Val) occur at a much higher rate in the two alvinellid worms. On the contrary, SA, VA, VI, QE, QK, QR MI, GA, GT appeared to be strongly asymmetric toward *A. pompejana*, favoring alanine, isoleucine and threonine and, unfavoring methionine and glutamine (for a more hydrophobic/charged situation).

### Search for structural differences between alvinellid species in 49 complete ribosomal proteins

To evaluate the structural impact of the amino acid replacements between the two alvinellid worms, 3D models of 49 complete ribosomal proteins (29 large and 20 small subunits) were determined and checked to identify the position (exposed *vs* core regions of the protein) and the level of burial of the mutated residue.

**Figure 8:**
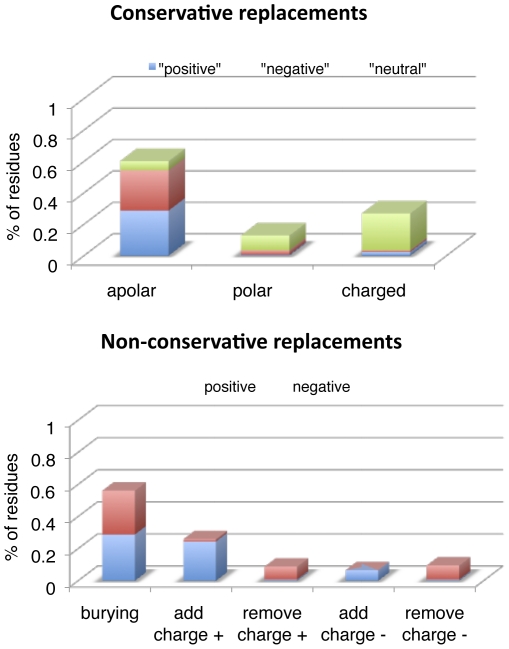
Expected structural effect of the 305 residue replacements found between the two alvinellid species in ribosomal proteins. Putative positive (blue bar), neutral (green bar) or negative (red bar) effect in terms of solvant exposure and charge modification on the 3D predicted structure of the 49 complete orthologous ribosomal proteins retrieved from the two alvinellid species. Replacements have been categorized into (above) conservative (i.e. polar to polar or apolar to apolar) and (below) non-conservative changes (i.e. polar to apolar or apolar to polar). Positive or negative effect was defined following three criteria: the level of residue burial when exposed or buried in the protein, the formation of hydrogen bonds and the formation of electrostatic bonds or exposed charges. (Above) Distribution of conservative changes from *P. grasslei* to *A. pompejana* between apolar, polar and charged residues. In the apolar category, positive = increase of the residue volume, negative = decrease of the residue volume and neutral = no volume change. In the charged and polar categories, positive = bond addition and negative = bond removal. (Below) Distribution of non conservative changes from *P. grasslei* to *A. pompejana* in terms of residue burial, hydrogen bonds, electrostatic bonds and solvation (positive: buried at a buried position or exposed at an exposed position, addition of hydrogen or electrostatic bonds, charged at exposed positions, negative: buried at an exposed position or exposed at a buried position, removal of bonds).

The structural analysis of amino acid replacements led to the identification of 305 non-synonymous changes that fell within the predicted 3D structure of the protein. Out of these 305 changes, about 55% were conservative (*i.e.* polar for polar, apolar for apolar) with 103 changes in the apolar category, 46 in the charged category and 22 in the polar one (Figure 8A). In the hydrophobic (aliphatic+aromatic) residues, calculation of volume difference between residue replacements did not show any significant difference between the two worms with 49 changes leading to a volume increase *vs* 45 leading to a volume decrease (see Figure 8A). Out of the 46 charged replacements, some of them led to the modification of the electrostatic network (4 additional bonds *vs* 2 bond removals) and thus can be seen as beneficial (+2: Figure 8A). In the polar ones, most changes were neutral with a net difference of 5 replacements leading to the removal of a hydrogen bond (Figure 8A). Most of these conservative changes were thus quite balanced and likely to behave neutrally but could also either affect the protein function or provide a pre-arranged protein environment improving the thermal stability of the ribosomal edifice. The remaining 139 non-conservative (45%) plus only 3 conservative changes are expected to have a beneficial or detrimental effect on the protein stability of the Pompeii worm by affecting either the core compactness or the level of interactions in the exposed surfaces (Figure 8B). However, most of the replacements were balanced in terms of positive (77 changes) *vs* negative (65 changes) effect when looking at their burial contribution in the exposed or buried parts of the protein or the formation of additional hydrogen or electrostatic bonds (see Figure 8B). Net difference in the formation of additional electrostatic bonds was only −2 in favor of *P. grasslei*. The only significant difference between the two alvinellid worms in terms of positive effect was the finding of an excess of positive charges at the surface of proteins (net difference = +15), favoring solvation or protein-protein interactions. Positive effects were mostly distributed in the coil regions of the protein (67%) and to a much lesser extent in helices (21%). This result indicated that replacements may have a more subtle stabilizing effect in terms of protein thermostability than expected (surface electronic interactions). This could be due to the fact that apparent neutral non-synonymous changes may also have a synergistic beneficial effect over the whole molecule.

## Discussion

Thermal adaptation has been extensively studied in thermophilic microorganisms by analysing amino acid replacements and codon usage, taking advantage of the massive genome sequencing effort done on these specific prokaryotes [4], [35]. In contrast, very little information has been collected on the molecular ‘adaptive’ strategy of thermophilic eukaryotes, and restricted to studies opposing endothermic *vs* ectothermic animals [13]–[35]. Although the molecular bases of eukaryote thermophily is far from being understood, specific amino acid replacements are likely to occur. Excess in positively-charged and hydrophobic residues may be a way to increase protein stability in thermophilic eukaryotes. However, these changes must be examined while taking into account the wide-genome mutational GC bias of metazoans because amino-acid composition strongly depends on the GC content of genes [35], which in turn relies both on phylogenetic constraints attributable to the taxon under scrutiny and the gene-specific selective pressures associated with the codon usage during the translation process [36]. Comparing the Pompeii worm to other eukaryotes, two interesting results can be highlighted: first, protein hydrophobicity is high and second, the worm displays the highest proportion of charged residues (mainly Lys and Arg) even when compared to homeotherms. Enrichments in Tyr, Ile and Leu are also characterizing ribosomal proteins of *A. pompejana*. However, a high hydrophobicity seems to be a general trend of annelids and especially polychaetes. Because *A. pompejana* is more closely related to AT-rich genomes (Figure 2B, [30]), especially to those of bivalves [37], the genome-wide mutational bias associated with lophotrochozoans may be partly responsible for such an amino-acid composition. Ribosomal proteins of *A. pompejana* however tend to maintain their high level of hydrophobicity despite a significant increase in polar charged residues. This may indicate a trade-off between the need for maintaining a high level of protein compactness (Tyr and Ile enrichments) but also a higher number of hydrogen/ionic bonds (Lys and Arg) to increase protein stability. Considering the GARP *vs* FYMINK relationship, the only outlier point corresponds to the Pompeii worm, which displays an unexpectedly high proportion of GARP residues and especially, of Arg and Pro residues. It therefore indicates that, even if the amino acid composition is biased towards AT-rich codons, Arginine and Proline have likely been favored by selective processes compared to other eukaryotes studied to date. In contrast, the proportion of Alanine conforms with its genome GC bias expectation while very depleted in the case of glycine.

To better understand the relationship between amino-acid composition and codon usage, the codon mutational bias was examined for both alvinellid species. Most of the variance associated with the codon usage was attributable to the GC_3_ content of genes and, thus suggested that the alvinellid genome is likely to be structured in AT- and GC-rich isochores, a situation similar to vertebrates [36], including ectothermic animals [38]. However, in contrast to most organisms, the 24 preferred codons were all GC-terminated. A closer examination of gene annotation indicated that two sets of genes are likely separated: housekeeping and highly expressed genes as opposed to less-expressed signaling and mitochondrial protein genes. This indicates that a strong translational selection is also likely to occur in a situation analog to other invertebrates [36]–[37]. Because *Alvinella*'s ORFs are overall AT-rich [30], the fact that the whole set of preferred codons is GC-terminated is very surprising. Indeed, the distribution of GC-terminated Fop codons so far seems to always correlate the genome-wide GC bias of organisms with a large proportion of GC-terminated codons in GC-rich genomes and *vice versa* [36]–[38]. This suggests that the translational machinery of alvinellids may compensate for the low GC phylogenetic trade-off of the worms by selecting the most stable tRNAs, at least for highly expressed proteins. This finding therefore supports the hypothesis that the translational machinery itself evolved under strong environmental, possibly thermal and/or pressure, selective constraints.

Examination of the amino acid composition to see if these patterns could have changed during the course of evolution is also a powerful way to test whether the thermotolerance of the Pompeii worm is recently-derived or more ancestral. If recently-derived, positive selection may still be detectable at sites involved in the thermal stability of proteins in the Pompeii worm. If only found in the alvinellid lineage, adaptive amino-acid patterns may reflect a more ancestral character that is likely to be maintained by purifying selection in thermophilic species. Alternatively, if those patterns only reflect phylogenetic constraints, then they should be preserved in all Lophotrochozoa. Analyses showed that the Pompeii worm genes are under strong purifying selection, suggesting that thermophily is not a recently-derived trait. In contrast, about 40 positively-selected codons were detected within the internal branch leading to the alvinellid lineage, with substitutions favoring polar and positively-charged residues but also cysteines. Although the positive accumulation of cysteines was detected in the specific case of ribosomal proteins (and not extracellular proteins), these residues may play a role in protein-protein assemblage of the ribosome. Here, the proportion of Cys residues was not directly anti-correlated to the aliphatic index as opposed to the strong negative relationship between the number of disulfide bonds and hydrophobicity (LMIV) [34]. Additional cysteines observed in alvinellids were however mainly restricted to small and extracellular proteins over our set of complete orthologous proteins. This could be indicative of the formation of extra disulfide bonds in the Pompeii worm, another way to rigidify proteins in the face of increasing hydrostatic pressure and temperature [39]. While the proteome of polychaetes appears to usually be hydrophobic, the alvinellid genome adaptation to the deep-sea vent habitat seems to have favored the emergence of more charged and hydrophilic proteins in a way similar to the ‘key residue-based’ mechanism already proposed for bacteria that recently colonized hot habitats [3]. Adaptation to the deep-sea vent habitat was however not as simple. Replacements that are specific to alvinellids (not only positive sites) also indicated a clear trend of hydrophobicity increase in both the polar and hydrophobic categories. This trend was accompanied with a global loss of charges in favor of hydrophobicity in a way similar to the compactness-based mechanism of thermophily [3]. Although both hydrophobicity and charged residues play a crucial role in the thermal stability of prokaryote proteins [7]–[8], [35], [40], such antagonistic results could depict a more complex situation in which protein evolution could be the consequence of two complimentary structuring forces: temperature and depth that do not necessarily act in the same direction. A closer look at the direction of amino acid replacements between the two alvinellid species (living at the same depth) showed that some specific replacements affect both species in the same way, suggesting that these changes are either the result of an adaptation to depth or, an adaptive trait of a putative thermophilic alvinellid ancestor: a plausible scenario as several other *Paralvinella* species also live under ‘hot’ conditions [20]–[21]. Although not yet clarified, such a scenario seems to be partly validated by the fact that positively-selected sites have only been detected in the branch leading to the alvinellid family and not in terminal branch leading to the Pompeii worm. This suggests that the common ancestor was likely thermophilic and differences in the amino-acid composition should be attributed to a relaxed selection for thermal stability in *P. grasslei*.

Postulating that the unique obvious environmental difference between the two alvinellid species is temperature, any amino-acid difference between these two species is likely to be due to either neutral evolution or diversifying selection in response to temperature. Replacements favoring charged residues and against glutamine were greater than expected under neutral evolution. Although far from reaching the threshold value of 4.5 classically put forth to distinguish hyperthermophilic prokaryotes [8], the (E+K)/(H+Q) ratio also notably increases in *A. pompejana* in a way similar to endothermic vertebrates [13]. Excess of charged residues against polar ones is expected to be the major component of thermophilic species (*i.e.* the ERK parameter, [40]) as it promotes stronger electrostatic interactions in protein surfaces [7]–[9]. Looking at the first enzyme crystal obtained for the Pompeii worm strenghened this view as the enhanced stability of the worm protein when compared to the human one was likely due to the formation of additional ionic bridges which negative effect in humans (ALS disease) is compensated by a charge neutralization at an adjacent site [28]. Arg-to-Lys substitutions were also more frequently found biased toward the Pompeii worm and could result in noticeable stabilization of proteins because of their residual dynamics of rotamer isomerization [41]. In this particular case, influence of the genome GC-content does not hold as the codon usage is exactly the same between the two species. Our results based on 3D predicted models clearly agree with such an assumption, but locations of amino acid changes also indicated that mechanisms towards protein thermostability may be more subtle. In our case, 3D structure models of ribosomal proteins suggested that about 45% of amino acid replacements (139 residues) could have an effect on the structure stability of ribosomal proteins. These replacements were however quite balanced between positive and negative effects, sometimes favoring compactness within the protein core and interactions at the exposed surface of the same protein, sometimes not. Moreover, volume difference calculation between hydrophobic residues did not show any trend of a specific increase of protein compactness between the two worms, with a global bulk difference of less than a glycine size (26 Å^3^). Looking more specifically at replacements affecting the charge of the protein, one of the major findings in terms of positive effect was the excess of positive charges at the surface of proteins in the Pompeii worm. Because this work was only performed on the ribosome edifice, these replacements may play a major role either in increasing solvation of exposed surfaces or the protein-protein/protein-RNA interactions: a situation that also fits well with the high proportion of cysteines found in positively-selected sites detected in the branch leading to the alvinellid lineage. Results however showed that most replacements are likely to behave neutrally and therefore fit theoretical expectations of neutral evolution predicting that most mutations are neutral or slightly deleterious. However, some of them could also represent a series of ‘neutral’ mutations leading to a protein pre-arrangement towards greater protein stability. This is particularly the case when a buried charge (expected to have a negative effect) is stabilized by a prearranged protein environment and is therefore tolerated by the hydrophobic core of soluble proteins without a loss of structure or activity and, even could induce stabilization by creating an additional electrostatic bond [42]. Similarly, residue pre-arrangement could also result in a protein polarity improvement by exposing more charged residues at the surface of multimeric proteins.

Protein evolution however seems to be more complex to explain the thermophilic life style of the Pompeii worm in the sense that selection may have also promoted a shift from the most hydrophilic polar residues to the less hydrophilic ones, and especially Ala. The depletion of Met towards Leu and Ile and the net imbalance of Tyr may also have a non-negligeable impact on the worm's life style. Depletion of Met towards Leu and Ile has been reported to occur in thermophilic proteins [43] and could be correlated to the absence of methionine sulfoxide reductases in *Alvinella* cDNA database [30], favoring its ability for a greater thermolability at high temperatures. Tyrosine has also been reported to increase in frequency in thermophiles as Tyr, due to its large side chain, is prone to create both local and long range interactions [44]. Hyperthermophily indeed seems to involve the progressive increase of a combination of amino acids IVYWREL [45]: a situation that fits well the dramatic increase of Tyr and Ile residues but not Val in *A. pompejana*. However, the main characteristic that distinguishes our findings from the extensive litterature dealing with prokaryote thermophily is the unusual increase of Ala residues in the Pompeii worm when compared to its cooler-living counterpart. The increase in Ala, and more generally speaking the modification of the aliphatic index [46], here Val to Ala/Ile or Met to Leu/Ile, may therefore be a specific way of metazoans to face relatively ‘high’ temperatures (*i.e.* exceeding 45°C). The increase of Ala is due to a strong mutational imbalance of Gly, Ser, Thr and Val toward Ala in the Pompeii worm. Replacements of Gly, Ser, Thr are known to only increase the protein stability in α-helices because of their high helical propensity [47]–[48]: a situation that fits well the position of Ala replacements in the Pompeii worm proteins, which is twice more common in helices. A higher Ala frequency in the well-buried positions of thermophilic proteins when compared to mesophilic ones has also been described in literature and was explained by the fact that residues with short alkyl group tend to interact more closely with neighbouring sites, and thus are likely to modify the packing shape [44]. This fits well the study performed on thermophilic and mesophilic *Corynebacterium* genomes, which suggested that Ala and Thr are likely to strenghen the hydrophobic interaction inside proteins, and thus provide a better proteome thermostability [49]. However, although β-branched residues are often found to destabilize α-helices [44], the general depletion of Val to either Ala or Ile is not easily explainable and must be analyzed on a case-by-case basis. The Gly-to-Ala and Val-to-Ala substitutions have also been reported to induce higher protein stability in mutant versions of enzymes [50]–[52] and, fit very well the alvinellid dataset in which these two changes together with Ser-to-Ala and Thr-to-Ala are often found, biased towards the Pompeii worm. Mutating such residues into Ala are likely to decrease the configurational entropy of protein unfolding mainly because Ala lacks the branched-β carbon of the other residues and thus has a much more flexible backbone in terms of configurational entropy [50]. However, mutations of more hydrophobic residues to Ala are often unpredictable and tend to be as often stabilizing as destabilizing due to the creation of a larger cavity [53]–[54]. In the specific case of a Val to Ala substitution, the cavity however is not large enough to allow the entrance of solvent molecules, and thus to have a destabilizing effect [54]. Despite its positive effect on helices, alanine accumulation is however puzzling as also frequently observed in coils and its effect(s) would need to be tested using mutagenesis experiments. One alternative explanation could be that such an increase is a direct consequence of a change in genome GC content. However, the two alvinellid worms display exactly the same codon usage and observed alanine proportions found in the worms conform to the genome-wide mutational bias predictions. A second more adaptive explanation would be that high hydrostatic pressure associated with the worm's habitat could have compensated for the creation of larger cavities, at least in replacements involving Thr, Ser and Val, while increasing flexibility in the backbone of the molecule. As a consequence, evolution towards positively charged residues, isoleucine, tyrosine and alanine seems to represent a convenient adaptive way to increase protein stability in the face of moderate-to-high temperatures, at least for deep-sea metazoans.

### Conclusion

Thermal adaptation has been extensively studied by analysing amino acid replacements and codon usage in thermophilic/ultrathermophilic microorganisms [1]–[3], [36]. In most cases, adaptation to thermophily relies on higher residue hydrophobicity and more charged amino acids with indices such as ERK or IVYWREL positively correlated with the optimal growth temperature of the prokaryotes [13], [41], [45]. In contrast, eukaryote thermophily has been neglected so far. Opposing endothermic and ectothermic animals [13] leads to a partial examination of the problem as endothermy represents a derived trait when compared to ectothermy, and thus cannot be controled for phylogeny without closely-related species pairs sampled in different thermal regimes. Here, we therefore present the first dataset comparing two closely-related eukaryotes, one being thermophilic, the other mesophilic. This analysis contrasts with previous theoretical background, which predicted that molecular ‘adaptive’ strategy of thermophilic eukaryotes is only reflecting gradual readjustments of the amino-acid composition when compared to prokaryotes [12]. Even if hydrophobicity and charged residues are still important molecular mechanisms by which eukaryote thermophily can be explained, our results indicate these kinds of replacements are likely to be ancestral, and thus a putative predisposition of the alvinellid lineage to high temperature. In contrast, present patterns of the amino-acid composition between two vent species are mainly based on more subtle replacements favoring isoleucine and alanine residues in the Pompeii worm. Even if there is no single outstanding amino acid patterns which account for thermostability, such changes appear to be a novel adaptive strategy for metazoans facing moderate thermophily. Such a strategy however needs to be more robustly tested by a long series of biochemical analyses comparing over-expressed mutant proteins between ‘cold’ and ‘hot’ adapted alvinellid lineages.

## Materials and Methods

Alvinellid specimens were collected on the surface of deep-sea hydrothermal vent chimneys along the East Pacific Rise (EPR) using the telemanipulated arm of either the manned submersible Nautile or the ROV Victor6000 and brought back to the surface inside an insulated basket during the oceanographic cruises Phare2002 (13°N/EPR) and BioSpeedo2004 (18°25S/EPR). Individuals were dissected in RNALater and subsequently frozen in liquid nitrogen following their recovery on board. Although not subjected to specific property regulations, authors already obtained permission to use samples for genomic analyses from both chief-scientists. The EST sequencing of *A. pompejana* and *P. grasslei* cDNA libraries yielded 15,858 transcripts encoding 9,221 proteins [30] for the former (accession numbers: FP489021 to FP539727 and FP539730 to FP565142), and 1653 unigenes (including the 335 orthologous genes used in the comparative analysis: see DatasetS2) for the latter, from which 259 and 80 ORFs encoding ribosomal proteins, respectively. A search for a set of orthologous ribosomal protein genes was performed across our own libraries, and complete sequenced genomes or mollusc/annelid EST libraries in Genbank/EMBL and JGI databases using reciprocal tBLASTx (p-value threshold of e^−20^). Abbreviations and corresponding species from which the concatenated set of ribosomal protein transcripts has been obtained from either complete genomes (*) or EST libraries (**) are listed as follow: Hsapiens: *Homo sapiens* (*), Dmelano: *Drosophila melanogaster* (*), Celegans: *Caenorhabtidis elegans* (*), Strong: *Strongylocentrotus purpuratus* (*), Capite: *Capitella teleta* (*), Helobd: *Helobdella robusta* (*), Bfloridae: *Branchiostoma floridae* (*), Lumbri: *Lumbricus rubellus* (**), Areni: *Arenicola marina* (**), Crasso: *Crassostrea gigas* (**), Argope: *Argopecten irradians* (**).

Sequences were translated using Se-AL v2.0 [55] and subsequently aligned with Clustal-W [56]. Amino-acid/codon alignments (Dataset S1) were then checked by eye, concatenated and exported in a PHYLIP format from a series of partial ribosomal protein genes (L5, L7, L7A, L9, L10, L10A, L12, L13, L13A, L14, L17, L17A, L18, L18A, L19, L21, L23A, L26, L27, L27A, L30, L31, L32, L35, L36, L37, L37A, L39, P2, S2, S3A, S4, S6, S7, S8, S13, S15, S15A, S16, S17, S18, S21, S23, S24, S25, S27, S27A). Regions containing gaps, misalignments or uncertainties were excluded from the analysis.

### Amino acids, codon frequencies and d_N_/d_S_ ratios

Observed amino acid and codon frequencies were estimated from 46 concatenated ribosomal protein genes (containing 5991 codons) using the codeML package of the software PaML v3.14 [57] and the ‘universal’ genetic code. Standard deviations on frequencies were obtained from 100 rearrangements (bootstrap) of the dataset. The user tree was obtained using the packages ProML of the PHYLIP v3.65 software [58] using the JTT model of amino-acid substitutions, the hidden Markov Model (HMM) and a Gamma+I distribution. Sequence hydrophobicities were calculated using the hydrophobic index based on the Sweet & Eisenberg's OMH scale [32] (weighted sum of D: −1.31, E: −1.22, N: −0.92, Q: −0.91, G: −0.67, K: −0.67, H: −0.64, R: −0.59, S: −0.55, P: −0.49, A: −0.40, T: −0.28, C: +0.17, W: +0.50, V: +0.91, M: +1.02, L: +1.22, I: +1.25, Y: +1.67, F: +1.92). This index takes into account the ability of an amino acid to be replaced by another during the course of evolution. The regression curve between GARP and FYMINK residues and the calculation of confidence intervals at 99% were performed using the software Statistica 8. The search for branches and codons under positive selection was performed under PaML v3.14 using LRTs between codon models and the Lophotrochozoan group as a phylogenetic background. We tested whether the ribosomal proteins evolved under different selective constraints across lineages by comparing the single d_N_/d_S_ ( = ω) ratio neutral model (M_0_) and the free d_N_/d_S_ ratio branch model (one ratio for each branch) implemented in codeML. We then identified sites in these lineages that have experienced a positive change during the course of the annelid evolution by considering a branch-site model of selection with *A. pompejana*, *P. grasslei*, alvinellid internal branch and the polychaete internal branch alternatively specified as the foreground lineage. This model includes four categories of omega (ω<1, ω = 1, ω>1 against the average omega background and ω>1 against a neutral omega background). The significance of this model is evaluated using LRT against a null model, the ‘nearly neutral’ site model M_1A_ with two categories of d_N_/d_S_ (ω close to zero and ω equal to 1). Bayes empirical Bayes (BEB) are used to compute posterior probabilities for ω classes and to identify sites under selection in case of significant LRT. We finally determined whether inferred positively-selected sites could be associated with a specific amino-acid ‘category’ in the foreground lineage/internal branch. This allowed us to get a better picture of whether thermal/depth adaptation is a recently-derived or an ancestral character.

### Fop codons and amino acid biases between alvinellid species

Fop codons, effective number of codons (Nc), and GC_3_ were estimated for each alvinellid species from a multivariate analysis (CA) implemented in the CodonW software [59] using a set of 335 orthologous ORFs (40338 codons: Dataset S2). Distributions of observed amino-acid replacements between the two species (2844 changes) were tested against the ‘empirical’ neutral distribution based on the WAG amino-acid mutability matrix [60] multiplied by the amino acid frequencies found in the two worms. Differences between observed and expected distributions based on 2844 substitutions were tested using a *Chi-2* test to see both the mutation asymmetry and its departure from neutral expectations. Outliers were selected from the distribution of differences for each amino acid replacement class at a threshold of 5% assuming that these differences are normally distributed. Finally, differences in amino acid counts between orthologous proteins of the two species were tested using a Binomial test.

### Structural comparisons

3D models from the 49 complete ribosomal proteins were obtained by comparative modelling using MODELLER 9v8 software. The structures of *Saccharomyces cerevisiae* or *Tetrahymena thermophila* homologues of large (29, pdb entry 1S1I) and small (20, pdb entry 1S1H or 2XZM) ribosomal subunits were used as templates following a series of parameters. The alignments used for homology modelling were improved manually, taking into account the predicted secondary structures of the query subunits and those of the templates. Fifteen models were generated for each subunit and their quality assessed using the Modeller Objective Function parameter; the best model was retained and the side chains were repositioned in optimized conformations, using SCWRL. The different ribosomal subunits models were finally subjected to energy minimization using the Gromos 96 force field (Deep View 4.0/Swiss PDB viewer). The accessible surface area (ASA) for each residue was then determined with the ANAREA program using a 1.4 Å probe radius and Shrake and Rupley's atomic parameters for Van der Waals radii. Residue was defined as buried if the ASA value between 0 Å^2^ and 20 Å^2^, intermediate if the ASA value is between 20 Å^2^ and 60 Å^2^ and exposed for those with ASA value ≥60 Å^2^. The twenty residues were classified as hydrophilic, charged or hydrophobic and replacements were sorted as structurally neutral, negative or positive following their buried/exposed status and their ability to introduce/suppress electrostatic or hydrogen bonds. Basically, replacement of a residue between *Alvinella* and *Paralvinella* was considered conservative if both belonged to the same category. Conservative replacements were considered as neutral if they did not affect much the volume of the residue and if they were not involved in the formation of additional ionic or hydrogen bonds. Non-conservative replacements were those producing a modification of the hydrophilic *vs* hydrophobic status of the residue. Both replacements of a hydrophilic residue by a hydrophobic one at a buried position or of a hydrophobic residue by a hydrophilic one at an exposed position were considered as positive because they usually have a stabilizing effect on the protein. Conversely, both replacements of a hydrophilic by a hydrophobic one at an exposed position or of a hydrophobic residue by a hydrophilic one at a buried position were considered as destabilizing [61]. This criterium was however refined by considering separately replacements involving charged residues. Polar and charged residues found in a buried position were considered as negative unless they are able to produce an additional electrostatic/hydrogen bond since charges do not always have a destabilizing/stabilizing effect when placed in a buried/exposed environment [34]. The 3D models were also used to examine the position of alanine replacements in the ribosomal proteins between the two alvinellid species.

## Supporting Information

Figure S1 **Relationship between the frequency of each amino-acid residue in ribosomal proteins and the GC_3_ content of the associated coding gene using lophotrochozoan and model species.** Alvine (red circle), lophotrochozoan species (Capite, Helobd, Lumbri, Argope, Crasso: white circles), model organisms (Strong, Bfloridae, Dmelano, Celegans: grey circles), and *Homo sapiens* (Hsapiens: black circle).
(TIF)

Figure S2 **Biplot graphs showing the relationship between the proportion of cysteine residues together with the aliphatic index and the length of the protein in alvinellid worms.** (Above) graph with the cDNA length and (Below) graph with the aliphatic index. The analysis was performed from a set of 150 complete proteins ranged between 60 and 1000 residues within which an extra Cys residue has been found for either *A. pompejana* or *P. grasslei*.
(TIF)

Figure S3 **Biplot graphs of the observed effective number of codons (Nc) as a function of the GC_3_ content.** Obtained from the 335 orthologous genes together with their expected bell-shaped theoretical curves (dashed line) for (A) *P. grasslei* and (B) *A. pompejana*.
(TIF)

Dataset S1 **Full sequence alignment of the concatenated set of ribosomal proteins used in the phylogenetic analysis leading to the search of positive codon sites in the foreground branches leading to the polychaete and alvinellid lineages.**
(DOC)

Dataset S2 **Pairwise alignments of sequences obtained from the 335 orthologous genes used to compare the amino acid composition and codon usage between the two alvinellid worms.**
(DOC)
