# Supplementary figures and images for: Proteome Adaptation to High Temperatures in the Ectothermic Hydrothermal Vent Pompeii Worm
Source: PLoS One. 2012 Feb 10;7(2):e31150. doi: 10.1371/journal.pone.0031150 (PMC3277501; doi:10.1371/journal.pone.0031150)

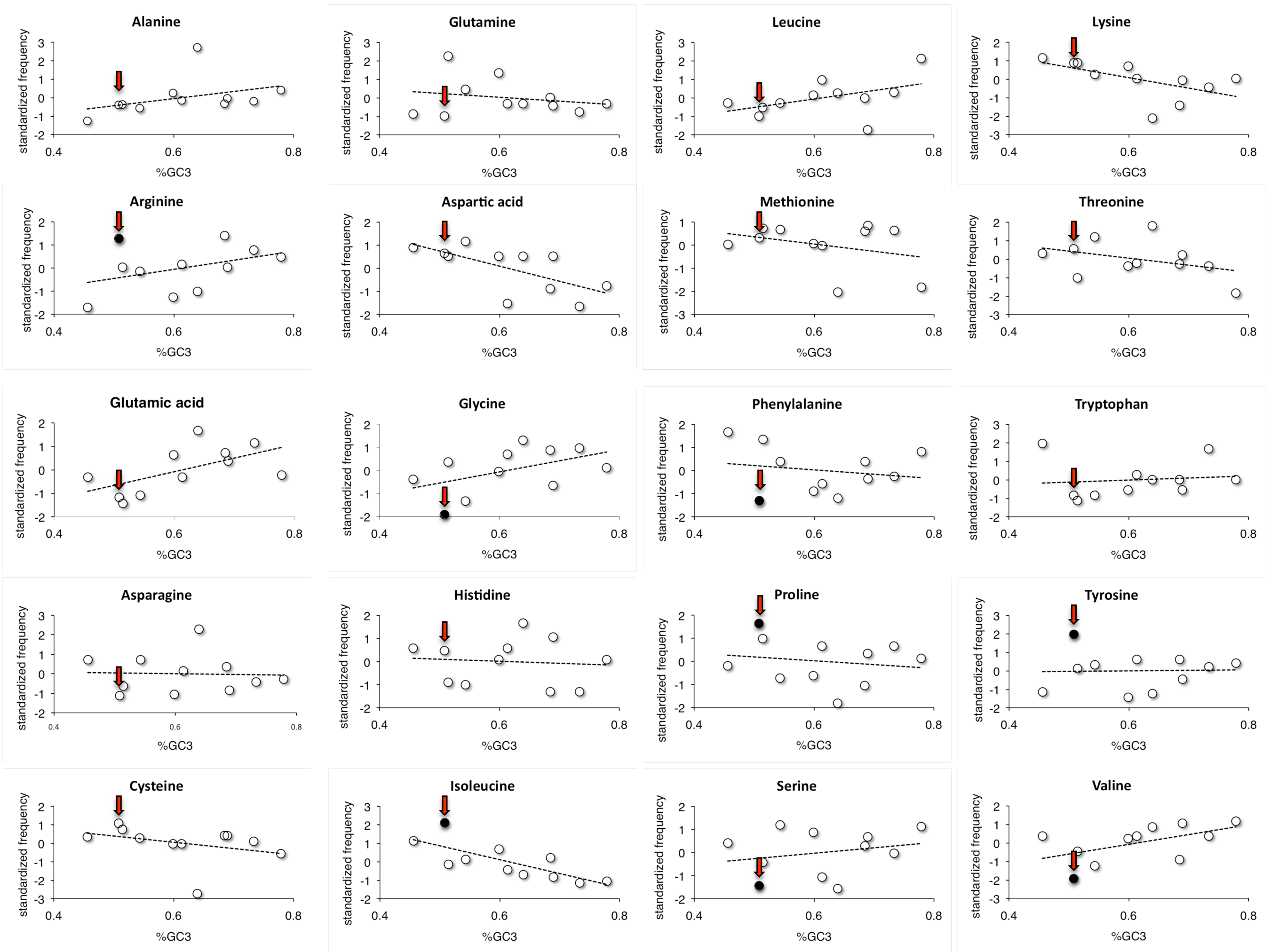

Supplement: Figure S1 — Relationship between the frequency of each amino-acid residue in ribosomal proteins and the GC3 content of the associated coding gene using lophotrochozoan and model species. Alvine (red circle), lophotrochozoan species (Capite, Helobd, Lumbri, Argope, Crasso: white circles), model organisms (Strong, Bfloridae, Dmelano, Celegans: grey circles), and Homo sapiens (Hsapiens: black circle). (TIF) [file pone.0031150.s001.tif]

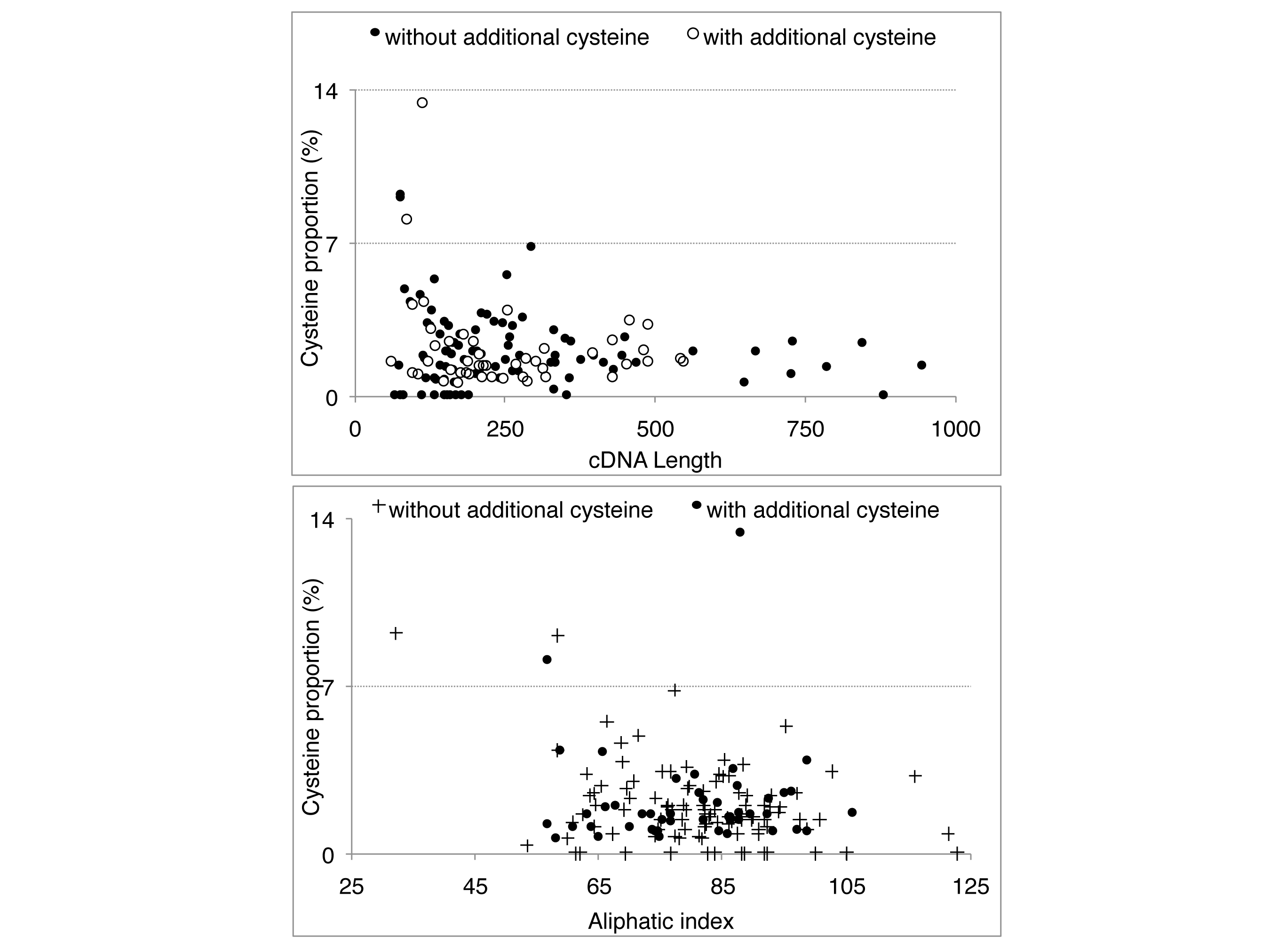

Supplement: Figure S2 — Biplot graphs showing the relationship between the proportion of cysteine residues together with the aliphatic index and the length of the protein in alvinellid worms. (Above) graph with the cDNA length and (Below) graph with the aliphatic index. The analysis was performed from a set of 150 complete proteins ranged between 60 and 1000 residues within which an extra Cys residue has been found for either A. pompejana or P. grasslei. (TIF) [file pone.0031150.s002.tif]

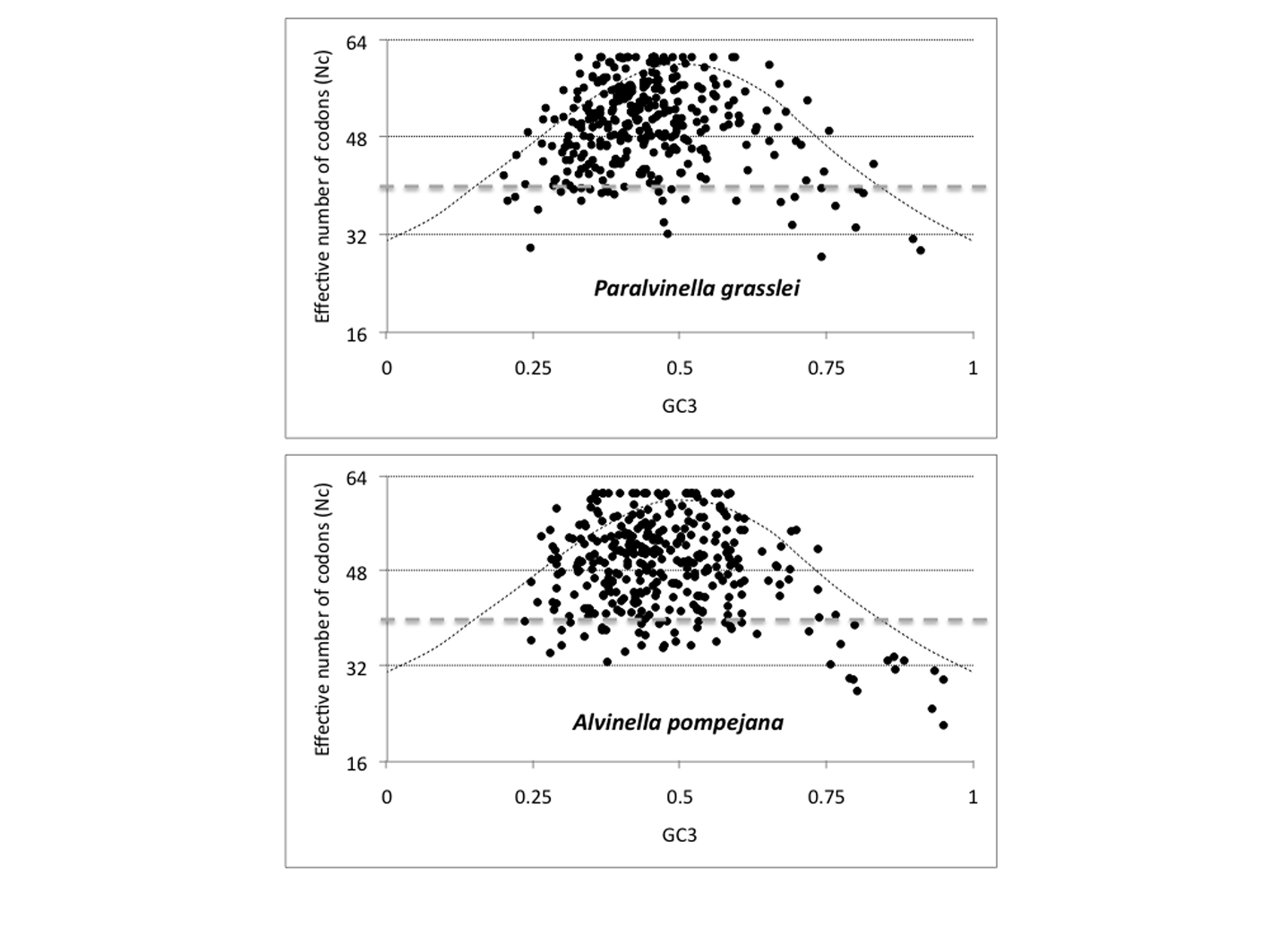

Supplement: Figure S3 — Biplot graphs of the observed effective number of codons (Nc) as a function of the GC3 content. Obtained from the 335 orthologous genes together with their expected bell-shaped theoretical curves (dashed line) for (A) P. grasslei and (B) A. pompejana. (TIF) [file pone.0031150.s003.tif]
