# Supplementary material for: Proteome Adaptation to High Temperatures in the Ectothermic Hydrothermal Vent Pompeii Worm
Source: PLoS One. 2012 Feb 10;7(2):e31150. doi: 10.1371/journal.pone.0031150 (PMC3277501; doi:10.1371/journal.pone.0031150)
Supplement: Dataset S1 — Full sequence alignment of the concatenated set of ribosomal proteins used in the phylogenetic analysis leading to the search of positive codon sites in the foreground branches leading to the polychaete and alvinellid lineages. (DOC) [file pone.0031150.s004.doc]

**Additional data file 3.**

Alignments of the concatenated set of ribosomal protein transcripts used in (A) the amino acid composition of the lophotrochozoan and model species and (B) the phylogenetic search of sites under positive selection in the lophotrochozoan group.

**A. Lophotrochozoan and model species.**

>Alvine

TCGCATCGCAAGTTCTCCGCGCCTCGTCACGGATCGCTGGGCTTCTTGCCCAAAAAGCGAAGTAGTAGGCATCGGGGTAA

GGTTAAAGCCTTTCCGAAGGATGACAAAAACAAGCCATTACACCTGACTGCTTTCTTGGGATACAAAGCAGGCATGACCC

ACATCGTTAGGGATGTAGACAGGCCCGGATCAAAGGTTAATAAGAAGGAAGTGGTGGAACCTGTCACCATCATCGAGACG

CCTCCAATGATAATTGTTGGTGTGGTGGGCTACATTGAGACTCCTCATGGTTTGAGGACCTTCAGGACTCTGTTTGCTGA

GCATCTGAGTGACGAATGCAAGCGTCGCTTTTACAAGAACTGGTACAAGTGCAAGAAGAAGGCATTCACCAAGGCCTGCA

AAAAGTACCAGGATGAACTGGGCAAGAAGGAGATCCAGAAGGATTTGAACAAGATGAAGAAATACTGCACTGTCATAAGG

GTGATTGCCCATACCCAGATGAAGATGCTGAAGAAACGCCAGAAGAAGGCCCACATCATGGAGATCCAAATTAATGGTGG

GACAATTGCTGATAAGGTTGACTGGGCGTATGCACATTTTGAGAAGCCAGTACAAGTGTCTGATGTGTTTGCCCAGGATG

AGATGATAGATATCATTGGTGTGACAAAGGGCAAGGGATTCAAAGGTGTCACTTCACGTTGGCATCCAAAGAAACTGCCA

CGCAAAACCCACAAGGGTCTGCGTAAGGTTGCCTGTATTGGAGCCTGGCATCCTGCCAGAGTGGCCTTCTCTGTAGCTCG

TGCT---AGGCCGCTAATAACCGTCTACAATGAGAAAGGTCAGGCCATCACTTTGCCGGCAGTTTTCCGTTCTCCAATCA

GGCCTGATATTGTGACCTTTGTGCACTCAGAAATGAGAAAGAATTCGAGACAGCCTTATGCTGTGTCATCTAAAGCTGGT

CACCAGACATCAGCCGAGTCATGGGGTACTGGCCGTGCTGTGGCTCGTATTCCTCGTGTGCGTGGTGGTGGCACACATCG

TTCTGGCCAGGGCGCCTTTGGAAACATGTGCCGTGGTGGTAGGATGTTTGCCCCAACCAAAACCTGGCGTAGGTGGCACA

GGCGCATTAATGTGAACCAGAGGCGGTATGCCTTGACTTCAGCCATTGCTGCAACAGGCATTCCTGCACTGGTCATGTCC

AAAGGGCATCGCATTGAGCAGACGCCTGAAGTGCCATTGGTAATTGCTGATAGCATTGAAGAAATCAAGAAGACAAAGCA

GGCAGTTGGTGTATTGCGCAGGTTTAAGGCTTGGTCTGATATTGAAAAGGTAAAAAACTCGAATCAGGATAATGGCATTA

CTAGAGCCTTCCGCAATATCCCAGGTGTGACCTTGCTGAATGTCTCTCGCCTGAACCTTTTGAAGATTGCACCAGGTGGA

CATGTTGGTCGTTTCTGCATCTGGACTGAATCGGCCTTTAAGAAGTTGGATAGCATTTAT---AGGAGAGAGGGCAAGAC

CGACTACTATGCCCGCAAGCGCCTCATCTTCCAGGCCAAGAACAAGTACAACACACCCAAGTACAGGATGATTGTACGCT

TCACCAACAAGGATATCGTATGCCAGATAGCCTATGCCAAGATTGAGGGTGATGTTGTTATCTGTTCTGCCTATGCCCAT

GAGCTGCCACGTTATGGGGTCACGGTTGGATTAACCAATTATGCTGCAGCATACTGTGTTGGCCTACTTTTGGCTCGCAG

GATGCTGAAGAAGTTTGGTTTGGATAAAATATATGCTGGTAGTGTGGATGTGACTGGAGATGAGTACATTGTGGAGATTG

ATGGACAGCCAGCACCATTCCGATGCTACCTAGATGTCGGCTTGGCAAGGACCAGCACTGGTGCCAAGGTGTTTGGTGCT

CTG---GTCAATCCGCTGTTTGAGAAGAGGCCTAAGAATTTTGGAATAGGCCAGGATATTCAGCCGAAGAGAGATTTGAG

CCGATTTGTAAAATGGCCAAAATACATCCGACTGCAGCGTAGGGCAATACTTTACCAGAGGCTAAAGGTTCCGCCTCCAA

TCAACCAGTTCACACAGGCCCTGGATAGACAGACAGCCACACAGCTGTTCAGACTTCTCGATCGCTACAGACCAGAGACC

AGACAGGCAAAGAAGATCCGACTGCGACTTCGTGCTGAGGAGAGAGTCAAGGGCAAGCCAGATGTGCCGACAAAACGTCC

TCCAGTTGTGAGATCTGGTGTCAACACAGTCACATCCTTGATTGAGCAGAAGAAGGCTCAGCTGGTCATCATAGCACATG

ATGTTGACCCTGTTGAGTTAGTTTTGTTCATGCCTGCTCTGTGCCGTAAGATGAACGTCCCTTATTGTATCGTGAAAGGA

AAGGCTCGACTTGGGCGTGTCGTACGTCGGAAGACGGCCACTTGTCTGGCTTTGACACATGTAAACGAGGACAAATCATC

ATTAAACAAGTTGGTTGAAGCTGTTAAGACGAACTTCAATGAGAGATACGATGAGATTCGTCGTCATTGGGGAGGTGGTA

TCATGGGTGCCAAGTCGCAGGCTCGT---ATGGGTCGAGTAGTGTTTAAGGCACACACAAAACACAGGAAGGGTCCGGCA

TTGAGAGCGTTCGACTTTTCCGAGAGACACGGATACATCAAAGGCGTTATAAGGGACATAATTCATGATCCAGGACGTGG

GGCTCCCCTGGCCCATGTAGTGTTCCGTGATCCTTACAGATACAAGCTGAAACATGAATTCTTCTACTGTGGCAAAAAAG

CCACACTGCAGATTGGCAACATCTTGCCAGTGGGTGTGATGCCTGAAGGCACAGTGGTCTGCTCTCTGGAGGAGAAAACT

GGTGACCGTGGACGACTGGCCAAGTGCTCTGGAAATTATGCCACTATCATCTCACACAATCCAGAAACGAAAAGGACGAG

GGTAAAGCTTCCATCTGGCACCAAGAAGGTCATTCCTTCAACCAACAGAGCATGTATTGGTGTTGTTGCTGGAGGTGGTC

GTATTGACAAGCCAATGTTGAAGGCTGGCCGTGCTTACCACAAGTACAAGGCCAAGAGGAACTGCTGGCCAAAAGTTCGT

GGTGTGGCCATGAACCCTGTTGAGCATCCCCACGGTGGTGGTAACCATCAGCACATTGGAAAAGCATCCACTGTCAGGAG

A---TCCGGTGACAGACTGACGCGTGCTGCAAAGGTATTAGAACAACTGACAGGACAGCAACCTGTCTTCTCTAAAGCTC

GTTACACCGTCAGATCATTTGGAATCCGAAGAAATGAGAAAATCGCTGTGCACTGCACCGTCCGAGGAGCCAAGGCAGAG

GAGATCCTAGAGAGAGGTCTGAAGGTTCGTGAGTATGAGTTGCGCAAGAACAACTTCAGCAAC---ACGTTCAAGCGTTT

TGTGGAGATTGGCCGTGTGGCTTACATTGCCACTGGACCAGATGCAGGCAAACTGGTTGCCATTGTGGATGTTGTTGATC

AGAATCGGGTGCTAGTTGATGGACCATGCACTGGAGTAGGCCGTAGGATTCTGAACCTGAAGCAGCTTGATCTAACAAAC

TTTGTCCTGAAGTTCTCGCACACTGCCAGGACAAGAGTCGTGCGAAAAGAATGGGAGAAGGCCGAGATTGACAAGAAGTG

GGCCGAGACCACCTGGGCCAAGAAGATGGAGCGTTTCAAGCTTATG---TACAAGTATATGCAGGAGCTCTACCGCAAGA

AGCAGAGCGACACGATGCGCTTCCTGCTTCGAATCCGTTGTTGGCAATACCGTCAGCTGTCAAAGATACACAGAGCACCA

CGACCTTCGCGCCCAGACAAGGCCAGAAGACTTGGATACAAGGCCAAACAAGGGTTTGTCATCTATCGTGTCCGTATCCG

TCGTGGAGGACGCAAGAGGCCAGTACCCAAAGGTCAGGTCTATGGTAAGCCATCCAACTGTGGCATCAACCAACTGAAGA

ACCAGAGGTCACTTCAGGCCATTGCTGAGGAACGTGTTGGTCGTAAATGTAAGGCCCTGCGTGTGTTGAACAGCTACTGG

GTTGCCCAGGACTCGACATACAAGTTCTTTGAGGTGATCGCGATTGATCCGTTCCACAAAGCCATCCGACGTGATCCAAC

ACTGCAGTGGATCTGTAAGCCAACTGCCAAGCACCGTGAGATGCGTGGTCTGACATCGGCAAACAGGAAGTCTCGTGGAC

TGGGCAAGGGACATCGCTTCACGAAGACTATTGGTGGTTCTCGTCGTGCTGCCTGGAAGAGAAACAACACGCTGCAAGCT

CACAGACGACGA---CGCTTTCTGGCCAGACGGACAGGATGCAAGTTTAACAGAATAATTCTGAAGAGACTTTTCATGAG

CAAGACTAACAGGGCGCCTATGTCTGCTTCAACTTTGAGACAGATGAAGAAGCCTGGACGTCAAGATAAGATAGCTGTTT

GTGTGGGCACAATTACTGATGACTCGCGTGCCTACAAGTTTCCAAAGCTGAAGGTCTGTGCCCTGCGTGTCACTCGTGCT

GCTCGTGCTCGTATCCTGAAGGCTGGTGGTGAATTGATGACCTTTGACCAGTTGGCGCTGAAGTCTCCCAGAGGAAAGAA

CACTGTACTTCTACAAGGTCCACGCAAGGCTCGTGAAGCCTACCGCCACTTTGGTCCAGCTCCTGGTGTACCCCACAGTC

ATACAAAGCCCTAT---AAAGAGTACAAGGTGATTGGGCGAATGCGCCCCACTGAAAAGACTCCTCAGCCTCCACTCTAC

CAGATGAGGATCTTTGCTCCAGACAAACCTACAGCAGTCTCTCGTTACTGGTACTTCTCCTCTCAACTGAAAAAAATGAA

GAAGACGCATGGAGAAATATTACTTTGTCAAGAGATTCGTGAGAAGAGGCCAAAAAAAATCAAGAATATTGGCATCTGGT

TGCGTTATGATTCTCGCAGTGGAACGCACAACATGTACAAAGAATACCGCGACCTTTCTGTTGCTGGAGCTGTCACTCAA

TGTCGTGACATGGCCGCAAGGCACAGAGCTAGAGCAGGATCAATCCAGGTGATCCGATGTGAAGTAATCCCAGCCAGCAA

GTGTCGAAGACCCTACATCAAACAGTTCCATGATAGCAAGATCAAGTTCCCTCTGCCACACAGAGTGTCCAGGCTGCATC

AACCTCAGTTCACAACTGTCCGGCCATCAACA---CTCCGTTTGCAGAAGCGGTTGGCCGCTTCAGTTCTGAAATGTGGC

CGTAATAAGATTTGGCTTGATCCAAATGAAACCAATGAGATAGCTAATGCTAATTCAAGACAAAACATCAGAAAATACAT

CAAGAATGGTTTGATCATCCGTAAACCTGTGGCAGTCCACTCACGTGCACGTGTCCGCAAGAACACAGAGGCTCGCCGCA

AGGGTCGTCATACTGGCCATGGTAAAAGGAAGGGTACGCGCAATGCCCGTATGCCACAGAAAATCATCTGGATGCGTCGC

ATGAGAGTACTGCGACGTCTTCTGAAACGATACAGGGAATCGAAGAAGATTGACAAGCATCTGTATCATCTGCTGTACAT

GAAGTGCAAAGGTAACGTCTTCAGAAACAAGAGGGTCTTGATGGAGCACATCCACAAGGAGAAGGCAAGAAAGGCTAGAA

TGAAGATGCTG---GACAGATATGCAAAATTATGGCGTAAGCCTCGTGGTATTGACAACAGAGTCAGGAGGAAGTTCAAA

GGTCAATATTTGATGCCAAACATTGGTTATGGCAGCAACAAGAAGACAAAACACATATGCCCTGATGGCTTCAGAAAATT

CCTTGTTCACAATGTTAGGGAGCTGGAGATTCTTATGATGCAAAACAAACGATTCTCTGCCGAGATAGCTCACACTGTGT

CAAGCAAGAAGAGGAAGGACATTGTGGAGCGAGCTCAACAGCTGTCCATTAAAGTAACTAATGCCAATGCTCGCATC---

CAGCTACGTGTGTCCAAGGTTACCGGAGGTAGTGCCTCCAAGCTGTCAAAGATTGTTCGTAAATCAATTGCCCGAGTCAT

GACAGTGATGCACCAGACACAGAAGGAAAACCTGCGCAAGTTTTACAAAGGTAAAAAGCACAAGCCAAAGGACTTGAGGC

GAAAGAAGACGAGGGCCATGCGCAAAGCTCTGACAAAGCATGAACTCAGCCTGAAGACAGCTAAACAGAGGAGGAAGAGT

CGTGCATTTCCCCTGAGAAAATATGCACTGAAA---TCAAAGCAGATGCAGCACATCCGCATAGCTCTGCGTGGCGCAGT

TGTGATGATGGGGAAGAACACCATGATGAGGAAGGCAATACGAGGTCATGTTGAGACTAATCCAGCCCTGGAAAAGCTGT

TGCCTCACATTAAGGGCAATGTTGGTTTTGTCTTTACCAAATGTGATCTGTCTGAAATCAGAAAGGTCATCAGCGAAAAC

AGGGTGGCAGCTCCTGCTAGGGCTGGTGCCATTGCCCCACTGGATGTGACACTGCCTGCCCATAACACAGGACTTGGCCC

AGAGAAGACCTCATTCTTCCAGGCTCTTGCTATTCCAACAAAGATTTCCAGAGGCACCATTGAAATCCAGAGTGATGTCA

AGTTGATTCGTGAAGGAGAGAAAGTTGGAGCCTCTGAAGCAACCCTGCTGAACATGTTGAAGATCTCGCCATTCACATAT

GGTTTGGTCATCAAGGTCTATGACTCGGGCACCATCTTTGATCCAGCCATTTTGGATGTGACAGATGAAGACCTCCGTTG

TAGGTTCATGGAGGGTGTTGCAAACATTGCCTCAGTGTCACTG---ACAAGAGCTGGTCAAAGAACTAGGTTCAAGGCTT

TTGTTGCCATTGGAGATTACAATGGTCATGTTGGTTTGGGTGTGAAGTGTTCTAAGGAAGTAGCCACTGCCATCCGTGGA

GCCATCATCTTGGCCAAACTGTCCATTGTTCCAGTACGCAGAGGTTACTGGGGTAACAAGATCGGAAAGCCCCACACTGT

TCCCTGCAAGGTTACTGGAAAGTGTGGTAGTGTACTGGTACGTCTGATTCCAGCACCACGTGGTACAGGTATTGTCAGTG

CACCTGTGCCAAAGAAGCTGCTCCAGATGAGTGGCATTGATGACTGCTACACTTCAGCCAGAGGACAGACGGCCACCCTT

---CTTGCCGAAGATGGATACAGTGGTGTAGAGGTACGCGTCACACCAGCACGGACAGAAATTATCATCCTGGCCACCAG

AACACAGAATGTCCTTGGTGAGAAGGGCCGTCGCATCAGAGAACTGACCTCTGTTGTGCAGAAGAGGTTCAACTTCCAAG

AAGGCACTGTTGAGCTGTATGCTGAGAAGGTGGCTACTCGAGGCCTGTGTGCCATTGCCCAAGCTGAATCATTAAGGTAC

AAGCTTATTGGAGGCCTGGCTGTTAGAAGGGCATGTTATGGTGTGCTGAGGTTCATCATGGAATCTGGAGCCAAGGGCTG

CGAGGTTGTGGTTTCTGGTAAGCTGAGAGGTCAGCGAGCCAAGTCCATGAAGTTTGTAGATGGTCTGATGATTCACAGTG

GTGAACCTTGCAATGAGTATGTGGACACAGCTGTCAGACATGTATTGCTCAGACAAGGTGTTCTGGGAATTAAGGTCAAG

ATCATGTTGCCATGGGATCCATCTGGTAAGATAGGACCAAAGCGTCCCCTTCCTGATAACATCAGCATTGTTGAGCCAAA

GGATGAA---GGTCCAAGGAAGCACTTGAAGAGGCTTCATGCCCCTAAACACTGGATGTTGGATAAGCTTGGGGGTGTTT

TTGCCCCACGCCCAAGCACTGGTCCACACAAGATGCGAGAGTGTCTCCCTCTGGTGGTGTTCTTGAGGAATCGCCTAAAG

TATGCTTTGACATATGATGAGGTGAAGAAGATTGTAAATCAACGTCTGATCAAGGTTGATGGCAAAGTGAGAACTGACAA

GACGTACCCTGCTGGCTTTATGGATGTGATCACAATAGAGAAGACAGCTGAAAACTTCCGTCTCATTTATGATGTCAAGG

GTCGTTTCACCATTCATCGTATCACTTCCCAAGAAGCCAAGTACAAACTGTGCATGATTACTGGTGGGCATAACTTGGGT

CGTGTTGGCCTTGTGACACACAGAGAACGTCATCCTGGTGGCTTTGACATTGTTCACATCAAGGACAGCATGGGCCACTC

ATTTGCCACACGGTTGAACAACGTGTTCATCATTGGTAAAGGTAACAAGCCATGGGTATCGCTGCCAGCTGGCAAGGGAT

TGAGGCTGACAATTGCAGAGGAACGTGACAAGAGAATCGCAGCA---TTGAACATATCTTACCCAGCCACGGGCTGTCAG

AAGCTCATCGAAGTGGACGATGAACACAAACTGCGACATTTTTACGAGAAGCGTATGGCAGCCGAGTTGCCGGCAGACCG

ACTTGGAGACGAATGGAAGGGATACATCGTACGTATTTCCGGTGGCAACGACAAACAAGGCTTTCCAATGAAGCAAGGCG

TGCTGACGTCGGGTCGTGTCAAGCTGCTGCTGAGTAAAGGCCACTCATGCTTCAGACCGAGACGTAGCGGTGAGAGGAGG

AGGAAATCTGTCCGTGGATGTATAGTGGACGCCAACCTCAGTGTGCTTAACCTGGTCATCGTCAGGAAAGGTGTTAATGA

TATTCCTGGTCTTACTGACAAGACTATCCCCCGTCGTCTTGGACCCAAAAGGGCCAGCAAAATCCGCAAGCTGTTCAACC

TGTCAAAGGAAGATGACGTCAGACAATATGTCGTTAAGAAGCCAAAGACAAAGGCCCCAAAGATCCAACGTCTTGTTACA

CCTTTGGTCTTA---ATCTCCCGGGACAAATGGCACAAGCGCAGGAAGACCGGTGGGCGTATGAACCCCATCCGGAAGAA

GAGGAAGTTCGAATTGGGAAGACCCGCTGCAAACACGAAGCTTACCGTAAGAACTCGAGGTGGTAACAAGAAGTACAGAG

CTTTGAGGCTGGACATGGGGAACTTCTCTTCTGAAGCCATCACCAAGAAGACACGTATCACTGATGTTGTATACAATGCC

AGCAGTAACGAGCTTGTACGTACAAAGACCCTCGTTAAAAACTGCATAGTTGTCATTGATGCATCACCCTTCAGATTGTG

GTATGAGGCTCACTATGCCATGCCTCTGATTAAGAAGAAAGGAGCTAAGGCCTGTGTTGCTTCACGACCAGGAAGGTGTG

GTAGGTGTGACGGATACATCTTGGAAGGCAAGGAGCTAGAATTCTACATGAAAAAGATTAAAGCAAAGAAA---AAAACA

TACGCCACTCCTAGACGTCCCTTCGAGAAGGAACGTCTCGACCAGGAGTTAAAATTAATCGGAGAGTATGGTCTGAGGAA

CAAGCGCGAGGTGTGGAGGGTCAAGTACACGCTGGCCAAAATCCGTAAGGCTGCTAGAGAACTGCTGACCTTGGACGAGA

AGGACCCGAGGCGTCTATTCGAAGGTAATGCTCTGTTGCGAAGACTCGTGCGTACGGGCGTGCTCGACGAGTCCAAGATG

AAGCTCGATTATGTGTTGGGTCTGCGTCTTGAGGACTTTCTGGAACGCCGTCTTCAGACGCAGGTGTTCAAGCTCGGACT

GGCCAAGAGCATTCATCATGCTCGTGTGCTGATTCGCCAGAGACACATCAGAGTACGTAAACAGGTGGTCAACATCCCTT

CCTACATTGTGAGACTGGACTCGCAGAAACACATAGACTTCTCACTGAGATCACCTTATGGTGGTGGT---TTCCAGAAG

CAGCCTGGGGTTTTTTTGAACAAAAAGCGTTATGTGAAGAACATTGGTCTTGGCTTTAAAACGCCGAGGGATGGCACCTA

CATTGACAAGAAATGTCCATTCACTGGACGAATAAGCATCCGTGGCCGTATCCTGACAGGTGTTGTGATGAAGATGAAGA

TGCAGCGAACGATTGTCATCCGACGAGACTACCTGCACTATGTCAGAAAGTACAATCGATTTGAAAAACGGCACAAGAAT

GTATCAGTCCACTGCAGTCCTTGCTTCAGAGATATTGCTGTTGGTGACATCGTTACTGTAGGTGAATGCCGGCCTCTCAG

CAAGACTGTC---GGTATTTCCCAGTCAGCCCTGCCCTATAGGAGAAGTGTTCCAACATGGTTGAAGTTAACACCTGATG

ATGTCAAGGAGCAGATCTATAAGTTGGCCAAGAAGGGTTTGACTCCATCTCAGATTGGTGTGATCCTTAGGGATTCCCAT

GGAGTTGCCCAGGTTAGATTTGTCACTGGCAACAAGATCCTGAGAATCCTGAAAGCCAAAGGTTTGGCACCAGACCTGCC

AGAGGATTTGTATTACTTGATCAAGAAGGCTGTGGCCATTAGGAAACATCTGGAACGTAATAGGAAGGACAGAGATGCCA

AATTCCGTCTGATTTTGGTTGAAAGCAGAATTCATAGACTGGCTCGATACTACAAGACCAAACGTGTGCTGCCACCAAAC

TGGAAG---ATGGTGCGTATGAACGTGCTGGCCGATGCCTTGAAGTCTATTTGTAATGCCGAGAAACGAGGCAAGAGGCA

GGTTCTGATCCGACCGTGTTCGAAGGTCATCGTCAGATTCCTGACCGTCATGATGAAGCATGGTTACATTGGTGAGTTTG

AGATAATTGATGACCACAGAGCAGGAAAAATTGTTGTTAATTTGACAGGAAGACTAAACAAGTGTGGTGTGATAAGTCCA

AGGTTTGATGTTTGCATCAGAGATATGGAGAAATGGACAACTAACCTATTGCCATCAAGACAGTTTGGGTACATTGTGCT

CACAACATCAGGTGGGATTATGGACCATGAGGAGGCCAGACGAAAACATTTGGGTGGAAAAATTCTAGGCTTCTTTTTC-

--GAAACTCAGATACATCGCATTCGTATTACCCTCACCAGCAGAAATGTAAAATCGTTAGAAAAAGTCTGTGCTGACCTG

ATCACTGGGGCAAAGGAGAAGAACCTGAAAGTGAAAGGACCTGTTCGCATGCCAACCAAGATTCTCCGTATCACAACACG

CAAGACACCTTGTGGTGAAGGATCTAAAACATGGGATCGTTTCCAAATGAGGATCCACAAGCGTCTCATTGACCTGCACA

GTCCATCAGAGATCGTGAAGCAGATCACATCCATCAGCATTGAGCCTGGTGTAGAGGTGGAAGTTACAATTGCTGAT---

ACCATAAGAACGAGAAAATTTATGACAAACCGTCTTCTATCAAGACGACAAATGGTTGTGGATGTTTTACATCCTGGACG

TGCAACGGTGCCAAAAACAGAGATCCGTGAGAAGTTGGCCCGCATGTACAAGACCACTTCTGACGTCATATTCTGTTTTG

GATTCCGTACACAATTCGGTGGCGGAAAGACGACTGGCTTCGCTCTGATCTATGATAATCTGGACTATGCCAAGAAATTC

GAGCCCAAATACAGATTAGCAAGG---GGCAAGCCTCATGGACTTAGGACAGCCCGTAAGATGCGGGACCACCGCCGTGA

CCAGAGGTGGCATGATAAAGACTACAAAAAGAGTCATTTGGGTACTCGCTGGAATCCATTTGGTGGCGCATCCCATGCTA

AGGGAATCGTCCTCGAGAAAGTAGGTGTTGAAGCCAAACAGCCCAACTCTGCCATTCGTAAATGTGTCAGAGTGCAGTTG

ATAAAAAATGGCAAGAAAATTACTGCTTTTGTACCAAGGGACGGTTGTCTGAACTACATCGAGGAGAATGATGAAGTTCT

CATTGCTGGATTTGGTCGTAAAGGTCATGCTGTAGGAGATATTCCTGGTGTACGCTTCAAGGTTGTTAAAGTGGCAAATG

TGTCCCTGTTGGCCTTGTACAAGGAGAAGAAGGAAAGACCAAGGTCTATG---AGGTTGGCCAGGAAACATGGTAACTTC

TATGTGGCTCCAGAGGCCAAGGTTGCCTTTGTAATCAGGATCAGAGGCATCAATGGCCTTCATCCAAGACCACGTAAAGT

GCTGAAGCTATTCCGTCTGCTTCAGATTAACAATGGCACCTTTGTTAGATTAAACAAAGCCACACTGCACATGCTGAGGA

TAGCTGACCCATATGTCACATGGGGGTATCCAAATCTGAAGAGTGTTCGTGAACTGATTTACAAACGAGGCTATGGAAGG

ATTGGTCGGATACCTCTGACTGACAATCTTATTGAGAGAAGTCTCATCATCTGTATTGAGGACTTGATCCATGAAATCTA

CACATGTGGGCCACATTTCAAGGAAGCCAACAGGTTCCTGTGGACTTTCAAACTGAACACTCCAACAGGAGGATGGGTGA

AGAAGAACAACCACTTTGTTGATGGTGGCGACTTTGGCAACAGA---GAGGAGAAGAAGAGGAAGTTCACAGAGAGCGTT

GAACTTCAGATATCACTGAAGAACTATGATCCTCAAAAAGACAAGCGTTTTGCTGGCACTGTCAAGTTGAAGTACATCCC

ACGTCCAAAGATGAAGGTCTGTGTTTTGGGTGACCAGCAGCATTGTGATGAAGCTAAAGCCAATGACATCCCATGTATGG

ATGTAGAGGCCCTGAAGAAGCTTAACAAGAACAAGAAACTTGTGAAAAAGCTGGCCAAGCGATATGATGCTTTTATCGCC

TCGGACAGTTTGATCAAACAGATTCCTCGTATTCTTGGTCCTGGCTTGAACAAGGCTGGTAAATTTCCAACCATGATCAC

TCATTCTGACTCCATGGTTAGCAAGGTTAATGAAGTGAAGGCCACCATCAGATTCCAGATGAAGAAGGTGTTGTGTCTTG

CTGTGGCTGTCGGTCATGTCAACATGAGCACAGAGGAGTTGGTTTCCAACATCATGATGGCCATCAACTTCCTCGTCTCG

CTGCTGAAGAAGAACTGGCAGAATGTACGAGCTCTGTACATCAAGTCCACCATGGGCCATGCATACCGCATTTAC---GC

CCACTTTCACAAGGACTGGCAGCGCTATGTGAAGTGCTGGTTCAACCAGCCTGCTCGTAAGAAGAGGAGGAGGAGGAAGC

GTATTGAGAAGGCACGGAAGATAGCTCCTAGACCGCTTCATACCCTCAGACCGATCGTACGCTGTCAGACCGTCCAATAC

AACACTCGTGTTCGTCTTGGACGGGGATTTACTCTTGAAGAGCTCAAGGCTGGAATCAACAAGCGTGAAGCCAGGACAAT

TGGCATCTCTGTAGACTTCCGACGTAAGAATCGATCAGTTGAGTCACTACAGCATAATGTCCAGCGTCTGAAGGAATATC

GCAGCAAATTGATCATCTTCAGGAAGCGCGGCAAACCA---ATGACTCGCTACAGTTTAGATCCAGAAAATGCTACGAAA

TCTGCTAAGGCTAGAGGGTCTCATCTCCGAGTACATTTCAAGAACACAAGAGAGACTGCTCAGACCATAAAGCATATGCA

TCTGCGACGTGCTGTCAGCTTTCTGAAGAATGTAAAAGCTCACAAGGAATGTGTTCCATTCCGCAGATACAATGGTGGAG

TCGGTCGCTGTGCCCAGGCAAAGAACTGGAAGACTACACAAGGCCGTTGGCCACAGAAGAGTGCTGAATTTCTTCTTCAG

CTGCTTAAGAATGCAGAAAGCAATGCTGAATTCAAGGGTTTAGATACTGATCATCTAGTAATTGAACACATCCAAGTGAA

TAGGGCTCCAAAAATGAGAAGACGAACATACAGAGCTCATGGAAGAATTAACTACATGAGTAGTCCATGCCACGTTGAAG

TTATCCTT---ATGAAGTTCAACCCGTTTGTATCTTCCTCTCGTAGGAAGAACAGAAAGCGTCACTTCAATGCTCCTTCC

CACATCAGAAGAAAGATAATGAGTGCCCCATTGTCAAAGGAACTCCGACAAAAGTACAACGTACGCAGTATTCCGATCCG

GAAAGACGACGAAGTCCAGGTTGTCCGAGGCCACTATAAAGGTCAGCAGGGCAAGGTTGTCCAAGTTTACAGAAAGAAAT

ATGTAATCCACATCGAGAGGATACAACGTGAAAAGGCCAATGGAGCCACAGTTCCTGTTGGCATACATCCATCAAAAGTT

GTCATCGTGAAGTTGAAGATGGATAAAGATCGTAAAGCAATTCTGGATCGTAAAGCCAGGTCAAAGGGAAAA---TCGGG

GAAGGTGGTCCTGGTCCTCAGCGGCCGCTACGCTGGCAGGAAAGCCGTCATCGTCAAGAACTATGATGATGGCACAACTG

ACAAGCCATATGGTCATGCCCTAGTTGCAGGAATTGATCGTTACCCTCGCATTGTCACAAAGAAAATGGGAAAAAAGAAG

ATCAAGCAGAGGTCAAAGATCAAGGCATTTGTCAAGGTGTACAACTTCAATCACCTGATGCCAACACGTTATTCAGTGGA

TGTGAGGTACAAGACTGGCAAAAACAAGTGGTTCTTCCAGAAACTACGATTC---CTACGTGGCCACGTCAGTCATGGAA

AGGGCCGTGTTGGCAAACACAGGAAACATCCAGGTGGACGTGGTAATGCTGGTGGCATGCATCATCACAGAATAAATTTT

GACAAATACCATCCTGGTTACTTTGGTAAATTGGGTATGCGTTACTACCACAAGACCAAACAGAAGTTTTACTGCCCAAC

AATAAATGTTGACAAACTGTGGTCACTAGTACCTGACCGGACACGTGAAGTAGCACCTGTTATTGATTGCTTGCGTGCTG

GTTATTACAAAGTACTGGGCAAGGGTCACCTACCAAGGCAGCCACTCATCGTTAAGGCAAAGTTCTTCAGTCAGTCTGCT

GAGAAAAAAATTAAAGATGTTGGTGGCTGCTGTATCTTGGTGGCA---ATGGCTGCCCACAAAACGTTCATCATAAAGAC

CAAGCTGGCGAAGAAGATGAAACAGAATAGGCCAATACCCCAGTGGGTCAGAATGAAAACGGGCAATACCATCAGGTACA

ATGCCAAGAGGAGACACTGGAAGAGGACGAAATTAAAGTTG---ATGGCACGTACGAAGCAGACTGCCCGTAAGTCAACC

GGAGGAAAGGCTCCGCGTAAGCAGCTGGCCACGAAGGCTGCTCGTAAGAGCGCCCCGTCCACCGGTGGAGTGAAGAAGCC

CCATCGTTACAGGCCCGGTACCGTCGCCCTCCGAGAGATCAGGCGTTACCAGAAGTCGACTGAGCTGCTTATCAGGAAGC

TGCCATTCCAGCGACTCGTCCGTGAGATTGCTCAGGACTTCAAGACGGATCTGCGTTTCCAGAGTGCTGCTATCGGTGCA

CTTCAGGAGGCCAGTGAAGCATACCTGGTTGGTCTGTTTGAGGACACTAACTTGTGCGCCATCCACGCCAAGCGTGTAAC

CATCATGCCAAAAGACATCCAGCTGGCACGAAGAATACGTGGAGAGCGTGCT---CTGGCTTGCGTTTATTCGGCTTTAA

TTCTTGCCGATGACGATGTACCAATCACTGCTGACAAGTTGAACACGATCTTGAAGGCGGCCAAAGTTGAAGTGGAACCA

TACTGGCCA---ATGCGTTACGTGGCTGCCTACTTGCTTGCTGTTTTGGGTGGTAATTCCAACCCTACAGCTAACGACAT

CAAACAGATCCTCAGTAGCGTTGCTGTTGACGTTGATGAGAATTCTCTGAGCAAGGTTATCAAGGAACTCCAAGGAAAAG

ATATCAAGGAACTTATGGCTGAAGGT---GACAAGGAATGGATGCCAGTAACCAAGCTGGGACGACTCGTCAAAGACATG

AAGATCAAGTCCATTGAAGAGATCTACCTGTTCTCGTTGCCCATAAAGGAGTATGAGATCATTGACCATCTGCCCACGCT

GAAGGATGAAGTGTTGAAAATCATGCCAGTACAGAAGCAA---AAATATGTACCACACTCTGCTGGTAGATACCAAGTTA

AGAGATTCCGCAAGAGTCAATGTCCAATAGTCGAGCGCCTTGCTTGCTCACTGATGATGCATGGACGTAACAATGGCAAG

AAGCTGATGGCCATACGTATCGTCAAGCATGCTTTCGAGATCATACATCTGCTGACTGGAGAGAATCCCCTACAAGTTGT

GGTTAATGCCATCATCAACAGTGGTCCCCGTGAGGACTCGACTCGTATAGGACGAGCTGGTACTGGAGCTAGGGAAGCAG

CCTTCAGGAACATCAAGACAATAGCTGAGTGTTTGGCTGACGAACTGATCAATGCTGCCAAGGGCTCGTCCAACTCGTAT

GCCATCAAGAAAAAGGATGAA---AGGGCGAAGATAATTAAGCCTCGCGGCAGAGAACCTGATGAATTTGAAACGTCGAT

CTCCCAGGCTCTCCTGGAGCTGGAGATGAACAGTGACCTAAAGGCACAGCTTAGAGAATTGTACATCACTGCGGCTAAAG

AGATAGATGTCCAGGGTAAGAAGGCCATCATTTTATTTGTGCCTGTGCCACAGCTGAAAGCCTTCCAGAAGATCCAGACT

CGTTTAGTCAGAGAGCTTGAAAAGAAGTTCAGTGGGAAGCATGTTGTTGTAATAGCACAGAGAAGAATTCTGCCCAAGCC

TACAAGGAAAAGTCGAAAGAATAAACAAAAGCGACCTAGAAGTCGAACTCTGACTGCAGTACACAACAACATTCTGGATG

ACCTGTGTTTTCCAGCAGAAATTGTTGGTAAAAGGATTAGAATTTGTCTTGATGGATCACGAGTGATTAAAGTTCATCTG

GACAAGACACAGCAAACCAATGTGGAACATAAGTTGGACACATTCTCTGCGGTGTATAAGAAGCTTACTTCAAAGGAAGT

CACCTTTGAATTCCGAGAGGCAACATTG---ATGCCAAAGAAGAACCGCGTTGCCATTTATGAGCTGCTCTTCAAGGAGG

GCGTCATGGTAGCCAAGAAGGATTATTATGCCCCCAAACACCCCGAGCTCGATGTACCGAACCTTCAGGTCATTAAGGCC

ATGCAGTCTCTGAAGTCTCGTGGTTTAGTCAACGAGCAGTTTGCCTGGAGACATTACTACTGGTATCTGACGAACGAAGG

CATCCAGTACCTTAGGGACTTTCTCCACCTGCCGCCGGAGATCGTACCAGCCACTCTGAAA---CATGTGACAGATCTGT

CTGGAAAGGAGACCATTGTCCGTGTAACTGGTGGCATGAAAGTCAAGGCAGATCGTGATGAAGCTTCCCCCTACGCTGCT

ATGTTGGCAGCTCAGGATGTAGCTGAGAGGTGTAAATCCCTTGGCATTAATGCTCTGCACATTAAACTTAGAGCCACTGG

TGGAAACAAAACAAAGACCCCAGGTCCAGGAGCACAGTCTGCCTTACGTGCATTAGCACGATCTGGAATGAAAATTGGTC

GTATAGAGGATGTG---AAAAAGAAGAGACGTACCTTCAGAAAGTACACCTATCGTGGCGTCGACCTCGACCAACTGTTA

GATATGTCAAGTGATCAGCTCATGCAACTTTTCCATTGTCGTGCTCGAAGACGTTTTTCTCGTGGTCTGAAACGCAAACC

AATGGCTCTGTTGAAAAGACTACGTAAAGCCAAGAAAGAAGCTCCACCCATGGAAAAGCCAGAGTGCATCAAGACACATC

TGCGTGACATGATCATTGTTCCTGAAATGATTGGCAGCATTGTTGGTGTGTACAATGGCAAGACTTTCAATCAAGTAGAG

ATTAAGCCTGAGATGATTGGTCACTATCTTGGAGAGTTCAGCATCACCTACAAGCCAGTTAAACATGGAAGGCCTGGTAT

TGGTGCCACACACTCATCACGATTCATACCTCTGAAA---ATAATCGAGAAGTATTACACTCGCTTGACCCTGGACTTCC

ACACAAACAAGAGAATATGTGAAGAAATCGCCATAATTCCCAGCAAGAAGCTGAGAAACAAGATCGCAGGCTTTGTCACT

CACCTGATGAAGAGGATCCAGAAAGGTCCAGTGCGAGGTATTTCCATCAAGTTACAGGAAGAAGAAAGGGAGAGGAGAGA

TAACTATGTGCCTGAGATATCAGCTATTGAGCAAGATATCATTGAAGTTGATCCAGACACTAAGGAGATGTTGAAGTTA-

--CCCAGAAAATGCTCAGCCAGCACAAGAATTATTGGAGCTAAAGATCATGCTTCTATCCAGATAAACTTGGCAGAGGTT

GATGAGACAACAGGTCGTATGACAGGCCAATACAAGACATATGCCATCTGTGGGCAGATAAGGAGAATGGGTGAATCTGA

TGACTGCATTACCAGATTGGCAAAGAAAGATGGACTTGTTGCTAAG---CACGTACAACCTGTCCGCTGTACTAACTGTG

CCAGATGTGTACCAAAAGACAAGGCTATTAAAAAGTTTGTCATCCGAAACATAGTGGAGGCAGCAGCTGTTAGAGATATA

GCAGATGCATCTGTCTATGAAAGTTATGCTCTACCCAAGCTGTATGCAAAGCTTCACTACTGTGTATCATGTGCCATTCA

CAGCAAGGTAGTAAGGAACAGATCCCGAGAGGCCCGTAAGGATCGTACACCACCTCCCAGGTTCAGGCCT---ATGATCT

TTGTCAAGACTCTGACGGGTAAAACCATTACCTTAGAGGTTGTAAAGACAAAAATTCAAGAAGGCATTCCTCCAGATCAG

CAGAGACTGATCTTTGCTGGAAAGCAGCTTGAGGATGGAGACATCCAGAAGGAGTCCACTCTTCACCTTGGTGGTGCAAA

GAAGAGGAAGAAGAAGAACTATACCACACCAAAGAAGAACAAGCACAAGAAGAAGAAGGTCAAGTTGGCTGTACTGAAGT

GCTACAAGGTGGATGAGAATGGCAAGATCACACGTCTTCGCCGTGAGTGTCCTAATGAGGAATGCGGTGCTGGTGTTTTT

ATGGCCTCTCACTTTGACAGACAATACTGTGGCAAATGTAGTCTGACCTATGTCTACAACAAA---AAGCCCTATCCAAA

GTCACGTTTCTGTAGAGGTGTGCCTGATCCCAAGATCCGTATCTATGACTTGGGCAGGAAGAAGGCTCGTGTAGACGAGT

TCCCTCTGTGTGTGCATCTGGTATCGGATGAACTGGAGCAGATATCATCCGAAGCATTGGAGGCTGGCCGTATCTGTGCC

AACAAATACATGGTGAAGAACTGTGGCAAAGATGCCTTCCATCTGAGAGTCCGGGTGCATCCTTTCCATGTGCTGCGCAT

AAACAAGATGTTGTCATGTGCTGGAGCTGATAGGCTTCAGACAGGCATGCGTGGTGCTTTTGGTAAGCCACAGGGTACAG

TGGCTCGTGTACGTATTGGCCAGGCTCTGATGTCGGTACGCTGTAAAGATCAGCACCAAGAAAAGGCTGTGGAAGCTCTG

AGGAGGGCCAAGTTCAAGTATCCAGGCAGGCAAAAGATCTACATCAGCCGTAAATGGGGCTTCACT---AAACGAACGAA

GAAGGTGCACATCACTGGCAAATATGGCACCCGGTACGGGGCCTCTTTAAGAAAGGACGTCAAGAAGATGGAGATAACGC

AGCACTCCAAGTATACCTGCTCCTTTTGTGGCAAGGATGCCATGAAGAGGAAAGCTGTTGGTATATGGAGCTGCAAATCT

TGCAAGAAAACTGTGGCAGGTGGTGCTTATGTATATAGTACAACAGCTGCAGCCATGGTTAGGAGCACAATTCGTCGTCT

GCGAGAAATG---CACATCCAGAAGAAGACCTGTGCGAGCTGTGGATATCCGTCTAAGAGAATGCGAAAGTACAACTGGA

GCGAAAAAGCAAAGAGGAGGAGGACACAAGGTGTAGGACGTATGAGACATTTGAAAAAGGTCTTCAGACGATTTAGAAAT

GGCTTCCGTGAGGGTACTGTT---AAGGGTAGAGACTCCCTGTCTGCTCAGGGTAAAAGGCGATATGATAGAAAACAGGC

AGGATATGGTGGACAAACAAAACCCATCTTCAGGAAGAAGGCCAAGACAACAAGGAAAATTGTATTGAGGATGGAGTGTA

CTAAATGCAAGTATAAGAAACAGTTGCCTATCAAGCGTTGCAAACACTTTGAGTTGGGAGGTGACAGAAAGAAGAAG---

TTGACCAAACATAACAAGTTCATACGTGATCTAATCAGAGAAATTGCTGGACAAGCACCCTATGAGAAGAGAACAGTGGA

GTTGCTGAGAATTTCAAAAGATAAACGTGCATTGAAGTTCTGCAAGAAAAGGTTGGGATCACATCTTCGTGCCAAGAGGA

AGCGTGAAGAGATGGCAAACATCATCCAACAGATGAGGAAAGCACAA---AAGGCCATAGAGAGTATTAATTCTCGATTG

GCTCTTGTTATGAAGAGTGGTAAATATGTGCTTGGCTATAAGCAGACGTTGAAGACACTCCGACAGGGCAAAGCCAAGCT

TGTCATCATTGCAAACAACACGCCACCACTCAGAAAGAGTGAGATAGAGTACTATGCAATGTTGGCCAAAACAGGTGTTC

ATCATTATAATGGCAACAACATTGAACTTGGCACAGCTTGTGGAAAGTACTTCAGAGTTACAACACTGAGTATCACTGAT

CCTGGT---CATAAGAAACGCAGGTTCAAGGATCATAAACACAAGCTTCGCCGCTCCATCACACCTGGCACTATACTGAT

TCTTCTGGCTGGTAGACACAAGGGCAAGAGAGTCATCTTCCTGAAGCAGTTGAAATCGGGTCTTCTTCTGGTAACTGGGC

CACACCCAATCAATGGTTGTCCACTGAGACGTATCAATCAGATCTACGTAATTGCCACAAAAACAAAGATTGACATCAGT

GGAATCAACATACCTGAAAGGCTGACTGATGATTATTTCCGCCGTAAGAAGAATGAGGGCGAGATATTTGACACAAAGAA

AGAGTACAAAGTCACTGAAGAAAGAAAAGAAGATCAGTGTATTGTTGACAAGCAAATTCTT---GTTGTCATTACTGGAC

CTCGTGGCACACTTAGACGTGACTTCCGTCACATGGACATCAAGAAGGTAAGGACGGTGTGCTCCCACATTGAAAACATG

ATCAAGGGTGTGACTGGCTACAGGTACAAGATGAGGTCTGTGTATGCCCATTTCCCTATCAACATTAGCATCACAGAAGA

CAACACCATGGTTCAGATCCGCAATTTCTTGGGCGAGAAGTACACTCGCAATGTGAAGATGTTGCCAGGTGTTACCTGTG

TGGCTTCCGAAAAACAAAAGGATGAATTCATCCTGGAAGGCAATGACATTGAGCTGGTTTCAAGATCAGCTGCTTTGATC

CATCAATCAACAAAAGTG---GTATGCCTGAGGGCTGTGGGTGGTGAAGTGGGTGCTACGTCTTCGTTGGCTCCCAAAAT

TGGTCCACTCGGTCTGTCTCCAAAGAAGGTTGGAGATGATATTGCCAAGGCAACACAGGACTGGAAAGGTTTGAGAATAA

CAGTCCAGTTAACAATCCAAAACCGTCAAGCCAAGGTGTCTGTGGTACCCACAGCCTCGGCTCTTGTTATCAAGGCATTG

AAGGAACCACCACGTGACAGAAAGAAGGTGAAACATGTCAAGCACAGTGGTAATATTTCTATGGATGAAATCTACACCAT

TGCCCGTACTATGAGGCAAAGGAGTATGGCAAGGACATTCAAAGGCACTGTCAAAGAAATCTTAGGTACTGCTCAGTCTG

TCGGTTGCACTGTTGATGGTCAGCATCCT---GATGCCCGCGGGCATCTGTTGGGCCGTCTCGCCGCAGTTGTCGCTAAA

AATATCCTTCAAGGTCAGAGGATAGTGGTAGTACGATGCGAAGGTATCAACATTTCAGGAAGCTTCTACAGGAACAAGTT

AAAATACTTGGCTTTCTTGCGGAAGCGAATGAACACTAATCCGAGCCGTGGTCCTTTCCACTTCCGAGCTCCAAGCAAGA

TATTCTGGAGAACTGTGCGTGGCATGTTGCCACACAAGCTTACTCGTGGAAAGGTGGCTTTGTCCCACCTGAAGGTGTTT

GAGGGAATTCCACCTCCATATGACAAGAAGAAACGCATGGTAGTGCCCTCAGCTCTGAGAGTCCTTAGACTGAAGGCTAG

ACGCAAGTACTGTGTTCTTGGACGCCTTTCCCATGAGGTTGGTTGGAAGTACCAGAATGTGATTGAGACTCTAGAA---A

TGACGAATACAAAGGGTTACCGCCGTGGTACCAGGGATCTCTTTTCCCGACCCTTCCGTAGGAAGGGAGTCATCCCACTG

TCTACCTATATGAAAGTGTACAAAGTGGGCGACATAGTGGATATTAAGGGTAATGGAGCTGTCCAAAAAGGCATGCCTCA

CAAATACTACCATGGCAAGACTGGTCGTGTGTACAATGTTACCCAGCATGCTGTTGGTGTGATAGTTAACAAACGAGTTA

GGATCATTGCCAAGAGGATCAACTTGCGCATTGAACATGTCAAGCACTCCAAATGCCGTCAGGAGTTCCTGAACCGTGTC

AAGGAAAATGAAAGGAAAAAGGCAGAAGCCAAAGAGAAGGGAATTCGTGTTTGCTGCAAGAGGCCTCGTCCTGGATACAT

TGTCAGGAGGAAACAGAAGCCAGAATTGTTGCAACCAATTCCATATGAATTCATTGCC---GTCACCAACGAGGTGGTGA

CGCGGGAATACACCATCAACATCCACAAACGAATTCATGGAATAGGATTCAAGAAGAGGGCACCACGAGCTATCAAGGCC

ATCAGACAGTTTGCCTACAAGCAAATGGGAACTGAAGATGTACGAATAGAAACAAGACTAAACAAACACATTTGGTCAAA

GGGAATTAGAAATGTTCCATTCCGAGTACGAGTACGATTGGCTCGTAAGAGGAATGAGGATGAGGACTCGCCTCACAAGC

TCTACACCTTGGTGTCCTATGTACCTGTCACCACATTCAAA---ACCTTGGTCACAAGAACTCAAGGAACAAAGATTGCC

TCTGATGGTCTAAAGGGTCGTGTATTTGAGGTGTCACTGGCTGATCTACAGAATGATGAAGTTGCCTTCCGTAAATTCAA

GTTGATGGCTGAAGATGTTCAGGGTCGCAATGTGTTGACCAACTTCCATGGCATGAGTTTGACTACTGACAAGCTACGAT

CCATGGTCAAAAAGTGGCAGACTCTGATTGAGGCCACCGTCGATGTACGTACAACTGATGGTTATTTGCTGAGACTGTTC

TGTATTGGCTTTACATCCAAGCAGAATCAGATCAAAAAGACCTGCTATGCTCAGAGCACTCAAGTTCGTGCCATCAGGAA

GAAAATGGTTGAAATCATGACCCGCGAAGTATCTTCAAATGACCTCAAGGAAGTTGTCAATAAACTGATCCCAGATAGCA

TTGGTAAGGACATTGAGAAGGCCTGCCAGGGTATCTACCCTCTTCATGATGTACTGATACGTAAAGTGAAGATCCTCAAG

AAACCCAAGTTTGATGGAGAGTCTGGTGAGAAGGTGACCCGCCCAGACAACTATGAGCCACCAATACAGAAGGAAGTC--

-GGACGTCCACTGGATCAGCTCCAACCAGAAATGCTTCGATACAAGCTTCAAGAACCTGTTCTGCTGCTAGGAAAGAAGA

GATTTGAAGGTGTTGACATCCGTGTCCGTGTGAAGGGTGGTGGTCACATAGCCCAGGTTTATGCTATTAGGCAAGCCATT

TCCAAGGCACTTGTGGCTTATTACCAAAAAGTTGACGAGGCATCAAAGAAGGAGATAAAGGACATGCTGATTGCCTATGA

CAGAACTCTTTTGGTTGCTGATCCAAGAAGATGTGAACCCAAAAAGTTTGGAGGCCCTGGCCCCAGAGCTCGCTACCAGA

AGTCATACCGA---AGATATTCCCACATTATTTTGAAGAAGGCAGATGTTGATCCAAGTAAGCGTGCTGGAGAGTTGGAC

GAGGATGAGAAAGTGGTGACAATTATGTCCAATCCACGCCAGTACAAGATTCCGGATTGGTTCCTGAACAGACAGAAAGA

TATCAAGGATGGAAAATACAGTCAGGTCTTGTCCAATGTGCTTGACAGCAAGATCCGTGAAGATTTGGAACGACTAAAGA

AGATCCGTGCCCACAGAGGTCTGCGACACTACTGGGGTCTGCGTGTCCGTGGTCAGCACACGAAGACAACGGGTCGCCGT

GGAAGGACTGTTGGTGTGTCCAAGAAG---TTAAAGGTACCTGAATGGACAGATATTGTGAAGAATGGACACCACAAAGA

AATGGCACCATATGATGATGACTGGTACTATGTCAGAGCTTCCGTAGCCAGGCATCTGTACATTAGGCCAGCTGGTGTCG

GTGCTTTCACCAAAGTCTATGGAGGGCGTAAAAGGAATGGCACATGTCCAAGTCATTTCTGTCGTGGTAGCTCGTCTGTA

GCCAGGAAAGTCCTACAGTCTCTGGAGGGACTCAAGCTTGTAGAGAAAGATCCTAATGGTGGCCGTCGTCTGACTTCACA

AGGTCGTCGTGATCTTGATCGCATTGCTTCACAGTTG

>Hsapiens

TCTCACAGAAAGTTCTCCGCTCCCAGACATGGGTCCCTCGGCTTCCTGCCTCGGAAGCGCAGCAGCAGGCATCGTGGGAA

GGTGAAGAGCTTCCCTAAGGATGACCCGTCCAAGCCGGTCCACCTCACAGCCTTCCTGGGATACAAGGCTGGCATGACTC

ACATCGTGCGGGAAGTCGACAGGCCGGGATCCAAGGTGAACAAGAAGGAGGTGGTGGAGGCTGTGACCATTGTAGAGACA

CCACCCATGGTGGTTGTGGGCATTGTGGGCTACGTGGAAACCCCTCGAGGCCTCCGGACCTTCAAGACTGTCTTTGCTGA

GCACATCAGTGATGAATGCAAGAGGCGTTTCTATAAGAATTGGCATAAATCTAAGAAGAAGGCCTTTACCAAGTACTGCA

AGAAATGGCAGGATGAGGATGGCAAGAAGCAGCTGGAGAAGGACTTCAGCAGCATGAAGAAGTACTGCCAAGTCATCCGT

GTCATTGCCCACACCCAGATGCGCCTGCTTCCTCTGCGCCAGAAGAAGGCCCACCTGATGGAGATCCAGGTGAACGGAGG

CACTGTGGCCGAGAAGCTGGACTGGGCCCGCGAGAGGCTTGAGCAGCAGGTACCTGTGAACCAAGTGTTTGGGCAGGATG

AGATGATCGACGTCATCGGGGTGACCAAGGGCAAAGGCTACAAAGGGGTCACCAGTCGTTGGCACACCAAGAAGCTGCCC

CGCAAGACCCACCGAGGCCTGCGCAAGGTGGCCTGTATTGGGGCATGGCATCCTGCTCGTGTAGCCTTCTCTGTGGCACG

CGCT---CGCCCACTGATATCGGTGTACTCCGAAAAGGGGGAGAATGTCACTTTGCCTGCTGTATTCAAGGCTCCTATTC

GACCAGATATTGTGAACTTTGTTCACACCAACTTGCGCAAAAACAACAGACAGCCCTATGCTGTCAGTGAATTAGCAGGT

CATCAGACTAGTGCTGAGTCTTGGGGTACTGGCAGAGCTGTGGCTCGAATTCCCAGAGTTCGAGGTGGTGGGACTCACCG

CTCTGGCCAGGGTGCTTTTGGAAACATGTGTCGTGGAGGCCGAATGTTTGCACCAACCAAAACCTGGCGCCGTTGGCATC

GTAGAGTGAACACAACCCAAAAACGATACGCCATCTGTTCTGCCCTGGCTGCCTCAGCCCTACCAGCACTGGTCATGTCT

AAAGGTCATCGTATTGAGGAAGTTCCTGAACTTCCTTTGGTAGTTGAAGATAAAGTTGAAGGCTACAAGAAGACCAAGGA

AGCTGTTTTGCTCCTTAAGAAACTTAAAGCCTGGAATGATATCAAAAAGGTCTATGCCTCTAATGAGGATAATGGTATCA

TCAAGGCCTTCAGAAACATCCCTGGAATTACTCTGCTTAATGTAAGCAAGCTGAACATTTTGAAGCTTGCTCCTGGTGGG

CATGTGGGACGTTTCTGCATTTGGACTGAAAGTGCTTTCCGGAAGTTAGATGAATTGTAC---CGACGAGAGGGTAAAAC

TGATTATTATGCTCGGAAACGCTTGGTGATACAAGATAAAAATAAATACAACACACCCAAATACAGGATGATAGTTCGTG

TGACAAACAGAGATATCATTTGTCAGATTGCTTATGCCCGTATAGAGGGGGATATGATAGTCTGCGCAGCGTATGCACAC

GAACTGCCAAAATATGGTGTGAAGGTTGGCCTGACAAATTATGCTGCAGCATATTGTACTGGCCTGCTGCTGGCCCGCAG

GCTTCTCAATAGGTTTGGCATGGACAAGATCTATGAAGGCCAAGTGGAGGTGACTGGTGATGAATACAATGTGGAAATTG

ATGGTCAGCCAGGTGCCTTCACCTGCTATTTGGATGCAGGCCTTGCCAGAACTACCACTGGCAATAAAGTTTTTGGTGCC

CTG---GTGAATCCCCTGTTTGAGAAAAGGCCTAAGAATTTTGGCATTGGACAGGACATCCAGCCCAAAAGAGACCTCAC

CCGCTTTGTGAAATGGCCCCGCTATATCAGGTTGCAGCGGAGAGCCATCCTCTATAAGCGGCTGAAAGTGCCTCCTGCGA

TTAACCAGTTCACCCAGGCCCTGGACCGCCAAACAGCTACTCAGCTGCTTAAGCTGGCCCACAAGTACAGACCAGAGACA

AAGCAAGAGAAGAAGCAGAGACTGTTGGCCCGGGCCGAGAAGAAGGCTGCTGGCAAAGGGGACGTCCCAACGAAGAGACC

ACCTGTCCTTCGAGCAGGAGTTAACACCGTCACCACCTTGGTGGAGAACAAGAAAGCTCAGCTGGTGGTGATTGCACACG

ACGTGGATCCCATCGAGCTGGTTGTCTTCTTGCCTGCCCTGTGTCGTAAAATGGGGGTCCCTTACTGCATTATCAAGGGA

AAGGCAAGACTGGGACGTCTAGTCCACAGGAAGACCTGCACCACTGTCGCCTTCACACAGGTGAACGAAGACAAAGGCGC

TTTGGCTAAGCTGGTGGAAGCTATCAGGACCAATTACAATGACAGATACGATGAGATCCGCCGTCACTGGGGTGGCAATG

TCCTGGGTCCTAAGTCTGTGGCTCGT---ATGGGCCGTGTGGTGTTCCGCGCGCACGTGAAGCACCGTAAAGGCGCTGCG

CTGCGCGCCGTGGATTTCGCTGAGCGGCACGGCTACATCAAGGGCATCGTCAAGGACATCATCCACGACCCGGGCCGCGG

CGCGCCCCTCGCCAAGGTGGTCTTCCGGGATCCGTATCGGTTTAAGAAGCGGACGGAGCTGTTCTATTGCGGCAAGAAGG

CCCAGCTCAACATTGGCAATGTGCTCCCTGTGGGCACCATGCCTGAGGGTACAATCGTGTGCTGCCTGGAGGAGAAGCCT

GGAGACCGTGGCAAGCTGGCCCGGGCATCAGGGAACTATGCCACCGTTATCTCCCACAACCCTGAGACCAAGAAGACCCG

TGTGAAGCTGCCCTCCGGCTCCAAGAAGGTTATCTCCTCAGCCAACAGAGCTGTGGTTGGTGTGGTGGCTGGAGGTGGCC

GAATTGACAAACCCATCTTGAAGGCTGGCCGGGCGTACCACAAATATAAGGCAAAGAGGAACTGCTGGCCACGAGTACGG

GGTGTGGCCATGAATCCTGTGGAGCATCCTTTTGGAGGTGGCAACCACCAGCACATCGGCAAGCCCTCCACCATCCGCAG

A---AGTGGAGACAGACTGACGCGAGCAGCCAAGGTGTTGGAGCAGCTCACAGGGCAGACCCCTGTGTTTTCCAAAGCTA

GATACACTGTCAGATCCTTTGGCATCCGGAGAAATGAAAAGATTGCTGTCCACTGCACAGTTCGAGGGGCCAAGGCAGAA

GAAATCTTGGAGAAGGGTCTAAAGGTGCGGGAGTATGAGTTAAGAAAAAACAACTTCTCAGAT---GTGTTCAGGCGCTT

CGTGGAGGTTGGCCGGGTGGCCTATGTCTCCTTTGGACCTCATGCCGGAAAATTGGTCGCGATTGTAGATGTTATTGATC

AGAACAGGGCTTTGGTCGATGGACCTTGCACTCAAGTGAGGAGACAGGCCATGCCTTTCAAGTGCATGCAGCTCACTGAT

TTCATCCTCAAGTTTCCGCACAGTGCCCACCAGAAGTATGTCCGACAAGCCTGGCAGAAGGCAGACATCAATACAAAATG

GGCAGCCACACGATGGGCCAAGAAGATTGATCGTTTTAAAGTTATG---TACAAGTACATCCAGGAGCTATGGAGAAAGA

AGCAGTCTGATGTCATGCGCTTTCTTCTGAGGGTCCGCTGCTGGCAGTACCGCCAGCTCTCTGCTCTCCACAGGGCTCCC

CGCCCCACCCGGCCTGATAAAGCGCGCCGACTGGGCTACAAGGCCAAGCAAGGTTACGTTATATATAGGATTCGTGTTCG

CCGTGGTGGCCGAAAACGCCCAGTTCCTAAGGGTGCAACTTACGGCAAGCCTGTCCATCATGGTGTTAACCAGCTAAAGT

TTGCTCGAAGCCTTCAGTCCGTTGCAGAGGAGCGAGTTGGACGCCACTGTGGGGCTCTGAGGGTCCTGAATTCTTACTGG

GTTGGTGAAGATTCCACATACAAATTTTTTGAGGTTATCCTCATTGATCCATTCCATAAAGCTATCAGAAGAAATCCTGA

CACCCAGTGGATCACCAAACCAGTCCACAAGCACAGGGAGATGCGTGGGCTGACATCTGCAGGCCGAAAGAGCCGTGGCC

TTGGAAAGGGCCATAAGTTCCACCACACTATTGGTGGCTCTCGCCGGGCAGCTTGGAGAAGGCGCAATACTCTCCAGCTC

CACCGTTACCGC---AGGTTTCTGGCCAGAAGAACCAACTCCACATTCAACCAGGTTGTGTTGAAGAGGTTGTTTATGAG

TCGCACCAACCGGCCGCCTCTGTCCCTTTCCCGGATGCGGAAGATGAAGCTTCCTGGCCGGGAAAACAAGACGGCCGTGG

TTGTGGGGACCATAACTGATGATGTGCGGGTTCAGGAGGTACCCAAACTGAAGGTATGTGCACTGCGCGTGACCAGCCGG

GCCCGCAGCCGCATCCTCAGGGCAGGGGGCAAGATCCTCACTTTCGACCAGCTGGCCCTGGACTCCCCTAAGGGCTGTGG

CACTGTCCTGCTCTCCGGTCCTCGCAAGGGCCGAGAGGTGTACCGGCATTTCGGCAAGGCCCCAGGAACCCCGCACAGCC

ACACCAAACCCTAC---CGAGAGTACAAGGTAGTGGGTCGCTGCCTGCCCACCCCCAAATGCCACACGCCGCCCCTCTAC

CGCATGCGAATCTTTGCGCCTAATCATGTCGTCGCCAAGTCCCGCTTCTGGTACTTTGTATCTCAGTTAAAGAAGATGAA

GAAGTCTTCAGGGGAGATTGTCTACTGTGGGCAGGTGTTTGAGAAGTCCCCCCTGCGGGTGAAGAACTTCGGGATCTGGC

TGCGCTATGACTCCCGGAGCGGCACCCACAACATGTACCGGGAATACCGGGACCTGACCACCGCAGGCGCTGTCACCCAG

TGCCGAGACATGGGTGCCCGGCACCGCGCCCGAGCCCACTCCATTCAGATCATGAAGGTGGAGGAGATCGCGGCCAGCAA

GTGCCGCCGGCCGGCTGTCAAGCAGTTCCACGACTCCAAGATCAAGTTCCCGCTGCCCCACCGGGTCCTGCGCCAGCACA

AGCCACGCTTCACCACCAAGAGGCCCAACACC---CTCAGGCTTCAGAAGAGGCTCGCCTCTAGTGTCCTCCGCTGTGGC

AAGAAGAAGGTCTGGTTAGACCCCAATGAGACCAATGAAATCGCCAATGCCAACTCCCGTCAGCAGATCCGGAAGCTCAT

CAAAGATGGGCTGATCATCCGCAAGCCTGTGACGGTCCATTCCCGGGCTCGATGCCGGAAAAACACCTTGGCCCGCCGGA

AGGGCAGGCACATGGGCATAGGTAAGCGGAAGGGTACAGCCAATGCCCGAATGCCAGAGAAGGTCACATGGATGAGGAGA

ATGAGGATTTTGCGCCGGCTGCTCAGAAGATACCGTGAATCTAAGAAGATCGATCGCCACATGTATCACAGCCTGTACCT

GAAGGTGAAGGGGAATGTGTTCAAAAACAAGCGGATTCTCATGGAACACATCCACAAGCTGAAGGCAGACAAGGCCCGCA

AGAAGCTCCTG---GACCGATATGTCAAAATTTGGCGGAAACCCAGAGGCATTGACAACAGGGTTCGTAGAAGATTCAAG

GGCCAGATCTTGATGCCCAACATTGGTTATGGAAGCAACAAAAAAACAAAGCACATGCTGCCCAGTGGCTTCCGGAAGTT

CCTGGTCCACAACGTCAAGGAGCTGGAAGTGCTGCTGATGTGCAACAAATCTTACTGTGCCGAGATCGCTCACAATGTTT

CCTCCAAGAACCGCAAAGCCATCGTGGAAAGAGCTGCCCAACTGGCCATCAGAGTCACCAACCCCAATGCCAGGCTG---

CAGCTGCGCGTCGCCAAAGTGACAGGCGGTGCGGCCTCCAAGCTCTCTAAGATCGTCCGGAAATCCATTGCCCGTGTTCT

CACAGTTATTAACCAGACTCAGAAAGAAAACCTCAGGAAATTCTACAAGGGCAAGAAGTACAAGCCCCTGGACCTGCGGC

CTAAGAAGACACGTGCCATGCGCCGCCGGCTCAACAAGCACGAGGAGAACCTGAAGACCAAGAAGCAGCAGCGGAAGGAG

CGGCTGTACCCGCTGCGGAAGTACGCGGTCAAG---TCCAAGCAGATGCAGCAGATCCGCATGTCCCTTCGCGGGGCTGT

GGTGCTGATGGGCAAGAACACCATGATGCGCAAGGCCATCCGAGGGCACCTGGAAAACAACCCAGCTCTGGAGAAACTGC

TGCCTCATATCCGGGGGAATGTGGGCTTTGTGTTCACCAAGGAGGACCTCACTGAGATCAGGGACATGTTGCTGGCCAAT

AAGGTGCCAGCTGCTGCCCGTGCTGGTGCCATTGCCCCATGTGAAGTCACTGTGCCAGCCCAGAACACTGGTCTCGGGCC

CGAGAAGACCTCCTTTTTCCAGGCTTTAGGTATCACCACTAAAATCTCCAGGGGCACCATTGAAATCCTGAGTGATGTGC

AGCTGATCAAGACTGGAGACAAAGTGGGAGCCAGCGAAGCCACGCTGCTGAACATGCTCAACATCTCCCCCTTCTCCTTT

GGGCTGGTCATCCAGGTGTTCGACAATGGCAGCATCTACAACCCTGAAGTGCTTGATATCACAGAGGAAACTCTGCATTC

TCGCTTCCTGGAGGGTGTCCGCAATGTTGCCAGTGTCTGTCTG---ACCCGTGCCGGCCAGCGCACCAGGTTCAAGGCAT

TTGTTGCTATCGGGGACTACAATGGCCACGTCGGTCTGGGTGTTAAGTGCTCCAAGGAGGTGGCCACCGCCATCCGTGGG

GCCATCATCCTGGCCAAGCTCTCCATCGTCCCCGTGCGCAGAGGCTACTGGGGGAACAAGATCGGCAAGCCCCACACTGT

CCCTTGCAAGGTGACAGGCCGCTGCGGCTCTGTGCTGGTACGCCTCATCCCTGCACCCAGGGGCACTGGCATCGTCTCCG

CACCTGTGCCTAAGAAGCTGCTCATGATGGCTGGTATCGATGACTGCTACACCTCAGCCCGGGGCTGCACTGCCACCCTG

---CTGGCTGAAGATGGCTACTCTGGAGTTGAGGTGCGAGTTACACCAACCAGGACAGAAATCATTATCTTAGCCACCAG

AACACAGAATGTTCTTGGTGAGAAGGGCCGGCGGATTCGGGAACTGACTGCTGTAGTTCAGAAGAGGTTTGGCTTTCCAG

AGGGCAGTGTAGAGCTTTATGCTGAAAAGGTGGCCACTAGAGGTCTGTGTGCCATTGCCCAGGCAGAGTCTCTGCGTTAC

AAACTCCTAGGAGGGCTTGCTGTGCGGAGGGCCTGCTATGGTGTGCTGCGGTTCATCATGGAGAGTGGGGCCAAAGGCTG

CGAGGTTGTGGTGTCTGGGAAACTCCGAGGACAGAGGGCTAAATCCATGAAGTTTGTGGATGGCCTGATGATCCACAGCG

GAGACCCTGTTAACTACTACGTTGACACTGCTGTGCGCCACGTGTTGCTCAGACAGGGTGTGCTGGGCATCAAGGTGAAG

ATCATGCTGCCCTGGGACCCAACTGGTAAGATTGGCCCTAAGAAGCCCCTGCCTGACCACGTGAGCATTGTGGAACCCAA

AGATGAG---GGTCCCAAGAAGCATCTGAAGCGGGTGGCAGCTCCAAAGCATTGGATGCTGGATAAATTGACCGGTGTGT

TTGCTCCTCGTCCATCCACCGGTCCCCACAAGTTGAGAGAGTGTCTCCCCCTCATCATTTTCCTGAGGAACAGACTTAAG

TATGCCCTGACAGGAGATGAAGTAAAGAAGATTTGCATGCAGCGGTTCATTAAAATCGATGGCAAGGTCCGAACTGATAT

AACCTACCCTGCTGGATTCATGGATGTCATCAGCATTGACAAGACGGGAGAGAATTTCCGTCTGATCTATGACACCAAGG

GTCGCTTTGCTGTACATCGTATTACACCTGAGGAGGCCAAGTACAAGCTGTGTATGGTGACTGGAGGTGCTAACCTAGGA

AGAATTGGTGTGATCACCAACAGAGAGAGGCACCCTGGATCTTTTGACGTGGTTCACGTGAAAGATGCCAATGGCAACAG

CTTTGCCACTCGACTTTCCAACATTTTTGTTATTGGCAAGGGCAACAAACCATGGATTTCTCTTCCCCGAGGAAAGGGTA

TCCGCCTCACCATTGCTGAAGAGAGAGACAAAAGACTGGCGGCC---CTGAACATCTCCTTCCCAGCCACTGGCTGCCAG

AAACTCATTGAAGTGGACGATGAACGCAAACTTCGTACTTTCTATGAGAAGCGTATGGCCACAGAAGTTGCTGCTGACGC

TCTGGGTGAAGAATGGAAGGGTTATGTGGTCCGAATCAGTGGTGGGAACGACAAACAAGGTTTCCCCATGAAGCAGGGTG

TCTTGACCCATGGCCGTGTCCGCCTGCTACTGAGTAAGGGGCATTCCTGTTACAGACCAAGGAGAACTGGAGAAAGAAAG

AGAAAATCAGTTCGTGGTTGCATTGTGGATGCAAATCTGAGCGTTCTCAACTTGGTTATTGTAAAAAAAGGAGAGAAGGA

TATTCCTGGACTGACTGATACTACAGTGCCTCGCCGCCTGGGCCCCAAAAGAGCTAGCAGAATCCGCAAACTTTTCAATC

TCTCTAAAGAAGATGATGTCCGCCAGTATGTTGTAAAGAAACCTAGGACCAAAGCACCCAAGATTCAGCGTCTTGTTACT

CCACGTGTCCTG---ATCTCTCGGGACAACTGGCACAAGCGCCGCAAAACCGGGGGCAAGAGAAAGCCCTACCACAAGAA

GCGGAAGTATGAGTTGGGGCGCCCAGCTGCCAACACCAAGATTACAGTCCGTGTGCGGGGAGGTAACAAGAAATACCGTG

CCCTGAGGTTGGACGTGGGGAATTTCTCCTCAGAGTGTTGTACTCGTAAAACAAGGATCATCGATGTTGTCTACAATGCA

TCTAATAACGAGCTGGTTCGTACCAAGACCCTGGTGAAGAATTGCATCGTGCTCATCGACAGCACACCGTACCGACAGTG

GTACGAGTCCCACTATGCGCTGCCCCTGGGCCGCAAGAAGGGAGCCAAGGCGTGCATCGCTTCAAGGCCGGGACAGTGTG

GCCGAGCAGATGGCTATGTGCTAGAGGGCAAAGAGTTGGAGTTCTATCTTAGGAAAATCAAGGCCCGCAAA---AAAACT

TATGTGACCCCGCGGAGACCCTTCGAGAAATCTCGTCTCGACCAAGAGCTGAAGCTGATCGGCGAGTATGGGCTCCGGAA

CAAACGTGAGGTCTGGAGGGTCAAATTTACCCTGGCCAAGATCCGCAAGGCCGCCCGGGAACTGCTGACGCTTGATGAGA

AGGACCCACGGCGTCTGTTCGAAGGCAACGCCCTGCTGCGGCGGCTGGTCCGCATTGGGGTGCTGGATGAGGGCAAGATG

AAGCTGGATTACATCCTGGGCCTGAAGATAGAGGATTTCTTAGAGAGACGCCTGCAGACCCAGGTCTTCAAGCTGGGCTT

GGCCAAGTCCATCCACCACGCTCGCGTGCTGATCCGCCAGCGCCATATCAGGGTCCGCAAGCAGGTGGTGAACATCCCGT

CCTTCATTGTCCGCCTGGATTCCCAGAAGCACATCGACTTCTCTCTGCGCTCTCCCTACGGGGGTGGC---TACCAAAAG

CAGCCGACCATCTTTCAAAACAAGAAGCGGTACTACAAGAACATCGGTCTGGGCTTCAAGACACCCAAGGAGGGCACCTA

CATTGACAAGAAATGCCCCTTCACTGGTAATGTGTCCATTCGAGGGCGGATCCTCTCTGGCGTGGTGACCAAGATGAAGA

TGCAGAGGACCATTGTCATCCGCCGAGACTATCTGCACTACATCCGCAAGTACAACCGCTTCGAGAAGCGCCACAAGAAC

ATGTCTGTACACCTGTCCCCCTGCTTCAGGGACGTCCAGATCGGTGACATCGTCACAGTGGGCGAGTGCCGGCCTCTGAG

CAAGACAGTG---GGCCTGTCCCAGTCGGCTTTACCCTATCGACGCAGCGTCCCCACTTGGTTGAAGTTGACATCTGACG

ACGTGAAGGAGCAGATTTACAAACTGGCCAAGAAGGGCCTTACTCCTTCACAGATCGGTGTAATCCTGAGAGATTCACAT

GGTGTTGCACAAGTACGTTTTGTGACAGGCAATAAAATTTTAAGAATTCTTAAGTCTAAGGGACTTGCTCCTGATCTTCC

TGAAGATCTCTACCATTTAATTAAGAAAGCAGTTGCTGTTCGAAAGCATCTTGAGAGGAACAGAAAGGATAAGGATGCTA

AATTCCGTCTGATTCTAATAGAGAGCCGGATTCACCGTTTGGCTCGATATTATAAGACCAAGCGAGTCCTCCCTCCCAAT

TGGAAA---ATGGTGCGCATGAATGTCCTGGCAGATGCTCTCAAGAGTATCAACAATGCCGAAAAGAGAGGCAAACGCCA

GGTGCTTATTAGGCCGTGCTCCAAAGTCATCGTCCGGTTTCTCACTGTGATGATGAAGCATGGTTACATTGGCGAATTTG

AAATCATTGATGACCACAGAGCTGGGAAAATTGTTGTGAACCTCACAGGCAGGCTAAACAAGTGTGGGGTGATCAGCCCC

AGATTTGACGTGCAACTCAAAGACCTGGAAAAATGGCAGAATAATCTGCTTCCATCCCGCCAGTTTGGTTTCATTGTACT

GACAACCTCAGCTGGCATCATGGACCATGAAGAAGCAAGACGAAAACACACAGGAGGGAAAATCCTGGGATTCTTTTTC-

--GAGGTGGCAATTCACCGAATTCGAATCACCCTAACAAGCCGCAACGTAAAATCCTTGGAAAAGGTGTGTGCTGACTTG

ATAAGAGGCGCAAAAGAAAAGAATCTCAAAGTGAAAGGACCAGTTCGAATGCCTACCAAGACTTTGAGAATCACTACAAG

AAAAACTCCTTGTGGTGAAGGTTCTAAGACGTGGGATCGTTTCCAGATGAGAATTCACAAGCGACTCATTGACTTGCACA

GTCCTTCTGAGATTGTTAAGCAGATTACTTCCATCAGTATTGAGCCAGGAGTTGAGGTGGAAGTCACCATTGCAGAT---

ACTATCCGCACTAGAAAGTTCATGACCAACCGACTACTTCAGAGGAAACAAATGGTCATTGATGTCCTTCACCCCGGGAA

GGCGACAGTGCCTAAGACAGAAATTCGGGAAAAACTAGCCAAAATGTACAAGACCACACCGGATGTCATCTTTGTATTTG

GATTCAGAACTCATTTTGGTGGTGGCAAGACAACTGGCTTTGGCATGATTTATGATTCCCTGGATTATGCAAAGAAAAAT

GAACCCAAACATAGACTTGCAAGA---GGCAAGTGTCGTGGACTTCGTACTGCTAGGAAGCTCCGTAGTCACCGACGAGA

CCAGAAGTGGCATGATAAACAGTATAAGAAAGCTCATTTGGGCACAGCCCTAAACCCTTTTGGAGGTGCTTCTCATGCAA

AAGGAATCGTGCTGGAAAAAGTAGGAGTTGAAGCCAAACAGCCAAATTCTGCCATTAGGAAGTGTGTAAGGGTCCAGCTG

ATCAAGAATGGCAAGAAAATCACAGCCTTTGTACCCAATGACGGTTGCTTGAACTTTATTGAGGAAAATGATGAAGTTCT

GGTTGCTGGATTTGGTCGCAAAGGTCATGCTGTTGGTGATATTCCTGGAGTCCGCTTTAAGGTTGTCAAAGTAGCCAATG

TTTCTCTTTTGGCCCTATACAAAGGCAAGAAGGAAAGACCAAGATCAATG---AGGATGGCAAGAAAAGCTGGCAACTTC

TATGTACCTGCAGAACCCAAATTGGCGTTTGTCATCAGAATCAGAGGTATCAATGGAGTGAGCCCAAAGGTTCGAAAGGT

GTTGCAGCTTCTTCGCCTTCGTCAAATCTTCAATGGAACCTTTGTGAAGCTCAACAAGGCTTCGATTAACATGCTGAGGA

TTGTAGAGCCATATATTGCATGGGGGTACCCCAATCTGAAGTCAGTAAATGAACTAATCTACAAGCGTGGTTATGGCAAA

ATCAATCGAATTGCTTTGACAGATAACTTGATTGCTCGATCTCTTATCATCTGCATGGAGGATTTGATTCATGAGATCTA

TACTGTTGGAAAACGCTTCAAAGAGGCAAATAACTTCCTGTGGCCCTTCAAATTGTCTTCTCCACGAGGTGGAATGAAGA

AAAAGACCACCCATTTTGTAGAAGGTGGAGATGCTGGCAACAGG---AACCAGAAGCGCCGCAAGTTCCTGGAGACGGTG

GAGTTGCAGATCAGCTTGAAGAACTATGATCCCCAGAAGGACAAGCGCTTCTCGGGCACCGTCAGGCTTAAGTCCACTCC

CCGCCCTAAGTTCTCTGTGTGTGTCCTGGGGGACCAGCAGCACTGTGACGAGGCTAAGGCCGTGGATATCCCCCACATGG

ACATCGAGGCGCTGAAAAAACTCAACAAGAATAAAAAACTGGTCAAGAAGCTGGCCAAGAAGTATGATGCGTTTTTGGCC

TCAGAGTCTCTGATCAAGCAGATTCCACGAATCCTCGGCCCAGGTTTAAATAAGGCAGGAAAGTTCCCTTCCCTGCTCAC

ACACAACGAAAACATGGTGGCCAAAGTGGATGAGGTGAAGTCCACAATCAAGTTCCAAATGAAGAAGGTGTTATGTCTGG

CTGTAGCTGTTGGTCACGTGAAGATGACAGACGATGAGCTTGTGTATAACATTCACCTGGCTGTCAACTTCTTGGTGTCA

TTGCTCAAGAAAAACTGGCAGAATGTCCGGGCCTTATATATCAAGAGCACCATGGGCAAGCCCCAGCGCCTATAT---CC

CCACTTCCACAAGGACTGGCAGCGGCGCGTGGCCACGTGGTTCAACCAGCCGGCCCGGAAGATCCGCAGACGTAAGGCCC

GGCAAGCCAAGGCGCGCCGCATCGCCCCGCGCCCCGCGTCGGGGATCCGGCCCATCGTGTGCTGCCCCACGGTTCGGTAC

CACACGAAGGTGCGCGCCGGCCGCGGCTTCAATCTGGAGGAGCTCAGGGCCGGCATTCACAAGAAGGTGGCCCGGACCAT

CGGCATTTCTGTGGATCCGAGGAGGCGGAACAAGTCCACGGAGTCCCTGCAGGCGAACGTGCAGCGGCTGAAGGAGTACC

GCTCCAAACTCATCCTCTTCAGGAAGCCCTCGGCCCCC---ATGGTTCGCTATTCACTTGACCCGGAGAACCCCACGAAA

TCATGCAAATCAAGAGGTTCCAATCTTCGTGTTCACTTTAAGAACACTCGTGAAACTGCTCAGGCCATCAAGGGTATGCA

TATACGAAAAGCCACGAAGTATCTGAAAGATGTCACTTTACAGAAACAGTGTGTACCATTCCGACGTTACAATGGTGGAG

TTGGCAGGTGTGCGCAGGCCAAGCAGTGGGGCTGGACACAAGGTCGGTGGCCCAAAAAGAGTGCTGAATTTTTGCTGCAC

ATGCTTAAAAACACAGAGAGTAATGCTGAACTTAAGGGTTTAGATGTAGATTCTCTGGTCATTGAGCATATCCAAGTGAA

CAAAGCACCTAAGATGCGCCGCCGGACCTACAGAGCTCATGGTCGGATTAACTACATGAGCTCTCCCTGCCACATTGAGA

TGATCCTT---ATGAAGTTTAATCCCTTTGTGACTTCCGACCGAAGCAAGAATCGCAAAAGGCATTTCAATGCACCTTCC

CACATTCGAAGGAAGATTATGTCTTCCCCTCTTTCCAAAGAGCTGAGACAGAAGTACAACGTGCGATCCATGCCCATCCG

AAAGGATGATGAAGTTCAGGTTGTACGTGGACACTATAAAGGTCAGCAAGGCAAAGTAGTCCAGGTTTACAGGAAGAAAT

ATGTTATCTACATTGAACGGGTGCAGCGGGAAAAGGCTAATGGCACAACTGTCCACGTAGGCATTCACCCCAGCAAGGTG

GTTATCACTAGGCTAAAACTGGACAAAGACCGCAAAAAGATCCTCGAACGGAAAGCCAAATCTAAGGGCAAA---CCTGG

GAAGGTGGTGCTTGTCCTGGCTGGACGCTACTCCGGACGCAAAGCTGTCATCGTGAAGAACATTGATGATGGCACCTCAG

ATCGCCCCTACAGCCATGCTCTGGTGGCTGGAATTGACCGCTACCCCCGCAAAGTGACAGCTGCCATGGGCAAGAAGAAG

ATCGCCAAGAGATCAAAGATAAAATCTTTTGTGAAAGTGTATAACTACAATCACCTAATGCCCACAAGGTACTCTGTGGA

TATCAGATACAAGACAGGCAAGAACAAGTGGTTCTTCCAGAAACTGCGGTTT---CTTAGGGGCCACGTGAGCCACGGCC

ACGGCCGCATAGGCAAGCACCGGAAGCACCCCGGCGGCCGCGGTAATGCTGGTGGTCTGCATCACCACCGGATCAACTTC

GACAAATACCACCCAGGCTACTTTGGGAAAGTTGGTATGAAGCATTACCACTTAAAGAGGAACCAGAGCTTCTGCCCAAC

TGTCAACCTTGACAAATTGTGGACTTTGGTCAGTGAACAGACACGGGTGGGGGCTCCCATCATTGATGTGGTGCGATCGG

GCTACTACAAAGTTCTGGGAAAGGGAAAGCTCCCAAAGCAGCCTGTCATCGTGAAGGCCAAATTCTTCAGCAGAAGAGCT

GAGGAGAAGATTAAGAGTGTTGGGGGGGCCTGTGTCCTGGTGGCT---ATGTCTTCTCACAAGACTTTCAAGATTAAGCA

ATTCCTGGCCAAGAAACAAAAGCAAAATCGTCCCATTCCCCAGTGGATTCGGATGAAAACTGGTAATAAAATCAGGTACA

ACTCCAAGAGGAGACATTGGAAAAGAACCAAGCTAGGTCTG---ATGGCTCGTACAAAGCAGACTGCCCGCAAATCGACC

GGTGGTAAAGCACCCAGGAAGCAACTGGCTACAAAAGCCGCTCGCAAGAGTGCGCCCTCTACTGGAGGGGTGAAGAAACC

TCATCGTTACAGGCCTGGTACTGTGGCGCTCCGTGAAATTAGACGTTATCAGAAGTCCACTGAACTTCTGATTCGCAAAC

TTCCCTTCCAGCGTCTGGTGCGAGAAATTGCTCAGGACTTTAAAACAGATCTGCGCTTCCAGAGCGCAGCTATCGGTGCT

TTGCAGGAGGCAAGTGAGGCCTATCTGGTTGGCCTTTTTGAAGACACCAACCTGTGTGCTATCCATGCCAAACGTGTAAC

AATTATGCCAAAAGACATCCAGCTAGCACGCCGCATACGTGGAGAACGTGCT---CTCGCCTGCATCTACTCGGCCCTCA

TTCTGCACGACGATGAGGTGACAGTCACGGAGGATAAGATCAATGCCCTCATTAAAGCAGCCGGTGTAAATGTTGAGCCT

TTTTGGCCT---ATGCGCTACGTCGCCTCCTACCTGCTGGCTGCCCTAGGGGGCAACTCCTCCCCCAGCGCCAAGGACAT

CAAGAAGATCTTGGACAGCGTGGGTATCGAGGCGGACGACGACCGGCTCAACAAGGTTATCAGTGAGCTGAATGGAAAAA

ACATTGAAGACGTCATTGCCCAGGGT---GATAAGGAGTGGATGCCCGTCACCAAGTTGGGCCGCTTGGTCAAGGACATG

AAGATCAAGTCCCTGGAGGAGATCTATCTCTTCTCCCTGCCCATTAAGGAATCAGAGATCATTGATTTCCTGGCCTCTCT

CAAGGATGAGGTTTTGAAGATTATGCCAGTGCAGAAGCAG---AAGTACCTGCCTCACAGTGCAGGGCGGTATGCCGCCA

AACGCTTCCGCAAAGCTCAGTGTCCCATTGTGGAGCGCCTCACTAACTCCATGATGATGCACGGCCGCAACAACGGCAAG

AAGCTCATGACTGTGCGCATCGTCAAGCATGCCTTCGAGATCATACACCTGCTCACAGGCGAGAACCCTCTGCAGGTCCT

GGTGAACGCCATCATCAACAGTGGTCCCCGGGAGGACTCCACACGCATTGGGCGCGCCGGGACAGGCGCTCGTGAGGCTG

CCTTCCGGAACATTAAGACCATTGCTGAGTGCCTGGCAGATGAGCTCATCAATGCTGCCAAGGGCTCCTCGAACTCCTAT

GCCATTAAGAAGAAGGACGAG---AGCGCCAAGATCGTGAAGCCCAATGGCGAGAAGCCGGACGAGTTCGAGTCCGGCAT

CTCCCAGGCTCTTCTGGAGCTGGAGATGAACTCGGACCTCAAGGCTCAGCTCAGGGAGCTGAATATTACGGCAGCTAAGG

AAATTGAAGTTGGTGGTCGGAAAGCTATCATAATCTTTGTTCCCGTTCCTCAACTGAAATCTTTCCAGAAAATCCAAGTC

CGGCTAGTACGCGAATTGGAGAAAAAGTTCAGTGGGAAGCATGTCGTCTTTATCGCTCAGAGGAGAATTCTGCCTAAGCC

AACTCGAAAAAGCACAAAAAATAAGCAAAAGCGTCCCAGGAGCCGTACTCTGACAGCTGTGCACGATGCCATCCTTGAGG

ACTTGGTCTTCCCAAGCGAAATTGTGGGCAAGAGAATCCGCGTCAAACTAGATGGCAGCCGGCTCATAAAGGTTCATTTG

GACAAAGCACAGCAGAACAATGTGGAACACAAGGTTGAAACTTTTTCTGGTGTCTATAAGAAGCTCACGGGCAAGGATGT

TAATTTTGAATTCCCAGAGTTTCAATTG---ATGCCTAAAAAAAACCGGATTGCCATTTATGAACTCCTTTTTAAGGAGG

GAGTCATGGTGGCCAAGAAGGATGTCCACATGCCTAAGCACCCGGAGCTGGCAGTGCCCAACCTTCATGTCATGAAGGCC

ATGCAGTCTCTCAAGTCCCGAGGCTACGTGAAGGAACAGTTTGCCTGGAGACATTTCTACTGGTACCTTACCAATGAGGG

TATCCAGTATCTCCGTGATTACCTTCATCTGCCCCCGGAGATTGTGCCTGCCACCCTACGC---CATGTCACTGATCTTT

CTGGCAAAGAAACCATCTGCCGTGTGACTGGTGGGATGAAGGTAAAGGCAGACCGAGATGAATCCTCACCATATGCTGCT

ATGTTGGCTGCCCAGGATGTGGCCCAGAGGTGCAAGGAGCTGGGTATCACCGCCCTACACATCAAACTCCGGGCCACAGG

AGGAAATAGGACCAAGACCCCTGGACCTGGGGCCCAGTCGGCCCTCAGAGCCCTTGCCCGCTCGGGTATGAAGATCGGGC

GGATTGAGGATGTC---CAGAAGAAGAAGCGGACCTTCCGCAAGTTCACCTACCGCGGCGTGGACCTCGACCAGCTGCTG

GACATGTCCTACGAGCAGCTGATGCAGCTGTACAGTGCGCGCCAGCGGCGGCGGCTGAACCGGGGCCTGCGGCGGAAGCA

GCACTCCCTGCTGAAGCGCCTGCGCAAGGCCAAGAAGGAGGCGCCGCCCATGGAGAAGCCGGAAGTGGTGAAGACGCACC

TGCGGGACATGATCATCCTACCCGAGATGGTGGGCAGCATGGTGGGCGTCTACAACGGCAAGACCTTCAACCAGGTGGAG

ATCAAGCCCGAGATGATCGGCCACTACCTGGGCGAGTTCTCCATCACCTACAAGCCCGTAAAGCATGGCCGGCCCGGCAT

CGGGGCCACCCACTCCTCCCGCTTCATCCCTCTCAAG---ATCATAGAAAAGTACTACACGCGCCTGGGCAACGACTTCC

ACACGAACAAGCGCGTGTGCGAGGAGATCGCCATTATCCCCAGCAAAAAGCTCCGCAACAAGATAGCAGGTTATGTCACG

CATCTGATGAAGCGAATTCAGAGAGGCCCAGTAAGAGGTATCTCCATCAAGCTGCAGGAGGAGGAGAGAGAAAGGAGAGA

CAATTATGTTCCTGAGGTCTCAGCCTTGGATCAGGAGATTATTGAAGTAGATCCTGACACTAAGGAAATGCTGAAGCTT-

--CCGCGGAAATGCTCCGCTAGCAATCGCATCATCGGTGCCAAGGACCACGCATCCATCCAGATGAACGTGGCCGAGGTT

GACAAGGTCACAGGCAGGTTTAATGGCCAGTTTAAAACTTATGCTATCTGCGGGGCCATTCGTAGGATGGGTGAGTCAGA

TGATTCCATTCTCCGATTGGCCAAGGCCGATGGCATCGTCTCAAAG---CACGTGCAGCCTATTCGCTGCACTAACTGTG

CCCGATGCGTGCCCAAGGACAAGGCCATTAAGAAATTCGTCATTCGAAACATAGTGGAGGCCGCAGCAGTCAGGGACATT

TCTGAAGCGAGCGTCTTCGATGCCTATGTGCTTCCCAAGCTGTATGTGAAGCTACATTACTGTGTGAGTTGTGCAATTCA

CAGCAAAGTAGTCAGGAATCGATCTCGTGAAGCCCGCAAGGACCGAACACCCCCACCCCGATTTAGACCT---ATGATTT

TCGTGAAAACCCTTACGGGGAAGACCATCACCCTCGAGGTTGTAAAGGCCAAGATCCAGGAAGGAATTCCTCCTGATCAG

CAGAGACTGATCTTTGCTGGCAAGCAGCTGGAAGATGGAGACATTCAAAAGGAGTCTACTCTTCATCTTGGTGGTGCTAA

GAAAAGGAAGAAGAAGTCTTACACCACTCCCAAGAAGAATAAGCACAAGAGAAAGAAGGTTAAGCTGGCTGTCCTGAAAT

ATTATAAGGTGGATGAGAATGGCAAAATTAGTCGCCTTCGTCGAGAGTGCCCTTCTGATGAATGTGGTGCTGGGGTGTTT

ATGGCAAGTCACTTTGACAGACATTATTGTGGCAAATGTTGTCTGACTTACTGTTTCAACAAA---AAGCCGTACCCAAA

GTCTCGCTTCTGCCGAGGTGTCCCTGATGCCAAGATTCGCATTTTTGACCTGGGGCGGAAAAAGGCAAAAGTGGATGAGT

TTCCGCTTTGTGGCCACATGGTGTCAGATGAATATGAGCAGCTGTCCTCTGAAGCCCTGGAGGCTGCCCGAATTTGTGCC

AATAAGTACATGGTAAAAAGTTGTGGCAAAGATGGCTTCCATATCCGGGTGCGGCTCCACCCCTTCCACGTCATCCGCAT

CAACAAGATGTTGTCCTGTGCTGGGGCTGACAGGCTCCAAACAGGCATGCGAGGTGCCTTTGGAAAGCCCCAGGGCACTG

TGGCCAGGGTTCACATTGGCCAAGTTATCATGTCCATCCGCACCAAGCTGCAGAACAAGGAGCATGTGATTGAGGCCCTG

CGCAGGGCCAAGTTCAAGTTTCCTGGCCGCCAGAAGATCCACATCTCAAAGAAGTGGGGCTTCACC---AAACGTACCAA

GAAAGTCGGGATCGTCGGTAAATACGGGACCCGCTATGGGGCCTCCCTCCGGAAAATGGTGAAGAAAATTGAAATCAGCC

AGCACGCCAAGTACACTTGCTCTTTCTGTGGCAAAACCAAGATGAAGAGACGAGCTGTGGGGATCTGGCACTGTGGTTCC

TGCATGAAGACAGTGGCTGGCGGTGCCTGGACGTACAATACCACTTCCGCTGTCACGGTAAAGTCCGCCATCAGAAGACT

GAAGGAGTTG---CACCTTCAGAAGTCGACCTGTGGCAAATGTGGCTACCCTGCCAAGCGCAAGAGAAAGTATAACTGGA

GTGCCAAGGCTAAAAGACGAAATACCACCGGAACTGGTCGAATGAGGCACCTAAAAATTGTATACCGCAGATTCAGGCAT

GGATTCCGTGAAGGAACAACA---AAGGGCAAGGATTCTCTGTACGCCCAGGGAAAGCGGCGTTATGACAGGAAGCAGAG

TGGCTATGGTGGGCAAACTAAGCCGATTTTCCGGAAAAAGGCTAAAACTACAAAGAAGATTGTGCTAAGGCTTGAGTGCG

TTGAGTGCAGATCTAAGAGAATGCTGGCTATTAAAAGATGCAAGCATTTTGAACTGGGAGGAGATAAGAAGAGAAAG---

CTGACCAAACACACCAAGTTCGTGCGGGACATGATTCGGGAGGTGTGTGGCTTTGCCCCGTACGAGCGGCGCGCCATGGA

GTTACTGAAGGTCTCCAAGGACAAACGGGCCCTCAAATTTATCAAGAAAAGGGTGGGGACGCACATCCGCGCCAAGAGGA

AGCGGGAGGAGCTGAGCAACGTACTGGCCGCCATGAGGAAAGCTGCT---AAGTCGCTGGAGTCGATCAACTCTAGGCTC

CAACTCGTTATGAAAAGTGGGAAGTACGTCCTGGGGTACAAGCAGACTCTGAAGATGATCAGACAAGGCAAAGCGAAATT

GGTCATTCTCGCTAACAACTGCCCAGCTTTGAGGAAATCTGAAATAGAGTACTATGCTATGTTGGCTAAAACTGGTGTCC

ATCACTACAGTGGCAATAATATTGAACTGGGCACAGCATGCGGAAAATACTACAGAGTGTGCACACTGGCTATCATTGAT

CCAGGT---CGAAAGCTGTTGAGCTTCAGTCAGCACGTGAGAAAACTGCGAGCCAGCATTACCCCCGGGACCATTCTGAT

CATCCTCACTGGACGCCACAGGGGCAAGAGGGTGGTTTTCCTGAAGCAGCTGGCTAGTGGCTTATTACTTGTGACTGGAC

CTCTGGTCCTCAATCGAGTTCCTCTACGAAGAACACACCAGAAATTTGTCATTGCCACTTCAACCAAAATCGATATCAGC

AATGTAAAAATCCCAAAACATCTTACTGATGCTTACTTCAAGAAGAAGAAGCAGGAAGGTGAGATCTTCGACACAGAAAA

AGAGTATGAGATTACGGAGCAGCGCAAGATTGATCAGAAAGCTGTGGACTCACAAATTTTA---GTTATCGTGAAGGGCC

CCAGAGGAACCCTGCGGAGGGACTTCAATCACATCGAACTCAGCCTTGTTCGGACTATTTGTAGTCATGTACAGAACATG

ATCAAGGGTGTTACAGGCTTCCGTTACAAGATGAGGTCTGTGTATGCTCACTTCCCCATCAACGTTGTTATCCAGGAGAA

TGGGTCTCTTGTTGAAATCCGAAATTTCTTGGGTGAAAAATATATCCGCAGGGTTCGGATGAGACCAGGTGTTGCTTGTT

CAGTATCTCAAGCCCAGAAAGATGAATTAATCCTTGAAGGAAATGACATTGAGCTTGTTTCAAATTCAGCGGCTTTGATT

CAGCAAGCCACAACAGTT---GTATACCTGAGGTGCACCGGAGGTGAAGTCGGTGCCACTTCTGCCCTGGCCCCCAAGAT

CGGCCCCCTGGGTCTGTCTCCAAAAAAAGTTGGTGATGACATTGCCAAGGCAACGGGTGACTGGAAGGGCCTGAGGATTA

CAGTGAAACTGACCATTCAGAACAGACAGGCCCAGATTGAGGTGGTGCCTTCTGCCTCTGCCCTGATCATCAAAGCCCTC

AAGGAACCACCAAGAGACAGAAAGAAACAGAAAAACATTAAACACAGTGGGAATATCACTTTTGATGAGATTGTCAACAT

TGCTCGACAGATGCGGCACCGATCCTTAGCCAGAGAACTCTCTGGAACCATTAAAGAGATCCTGGGGACTGCCCAGTCAG

TGGGCTGTAATGTTGATGGCCGCCATCCT---GATGGTCGAGGCCATCTCCTGGGCCGCCTGGCGGCCATCGTGGCTAAA

CAGGTACTGCTGGGCCGGAAGGTGGTGGTCGTACGCTGTGAAGGCATCAACATTTCTGGCAATTTCTACAGAAACAAGTT

GAAGTACCTGGCTTTCCTCCGCAAGCGGATGAACACCAACCCTTCCCGAGGCCCCTACCACTTCCGGGCCCCCAGCCGCA

TCTTCTGGCGGACCGTGCGAGGTATGCTGCCCCACAAAACCAAGCGAGGCCAGGCCGCTCTGGACCGTCTCAAGGTGTTT

GACGGCATCCCACCGCCCTACGACAAGAAAAAGCGGATGGTGGTTCCTGCTGCCCTCAAGGTCGTGCGTCTGAAGCCTAC

AAGAAAGTTTGCCTATCTGGGGCGCCTGGCTCACGAGGTTGGCTGGAAGTACCAGGCAGTGACAGCCACCCTGGAG---A

TGACGAACACAAAGGGAAAGAGGAGAGGCACCCGATATATGTTCTCTAGGCCTTTTAGAAAACATGGAGTTGTTCCTTTG

GCCACATATATGCGAATCTATAAGAAAGGTGATATTGTAGACATCAAGGGAATGGGTACTGTTCAAAAAGGAATGCCCCA

CAAGTGTTACCATGGCAAAACTGGAAGAGTCTACAATGTTACCCAGCATGCTGTTGGCATTGTTGTAAACAAACAAGTTA

AGATTCTTGCCAAGAGAATTAATGTGCGTATTGAGCACATTAAGCACTCTAAGAGCCGAGATAGCTTCCTGAAACGTGTG

AAGGAAAATGATCAGAAAAAGAAAGAAGCCAAAGAGAAAGGTACCTGGGTTCAACTAAAGCGCCCCAGAGAAGCACACTT

TGTGAGAAATGGGAAGGAGCCTGAGCTGCTGGAACCTATTCCCTATGAATTCATGGCA---GCCATCAACGAAGTGGTAA

CCCGAGAATACACCATCAACATTCACAAGCGCATCCATGGAGTGGGCTTCAAGAAGCGTGCACCTCGGGCACTCAAAGAG

ATTCGGAAATTTGCCATGAAGGAGATGGGAACTCCAGATGTGCGCATTGACACCAGGCTCAACAAAGCTGTCTGGGCCAA

AGGAATAAGGAATGTGCCATACCGAATCCGTGTGCGGCTGTCCAGAAAACGTAATGAGGATGAAGATTCACCAAATAAGC

TATATACTTTGGTTACCTATGTACCTGTTACCACTTTCAAA---ACGCTCGTCACCAGGACCCAAGGAACCAAAATTGCA

TCTGATGGTCTCAAGGGTCGTGTGTTTGAAGTGAGTCTTGCTGATTTGCAGAATGATGAAGTTGCATTTAGAAAATTCAA

GCTGATTACTGAAGATGTTCAGGGTAAAAACTGCCTGACTAACTTCCATGGCATGGATCTTACCCGTGACAAAATGTGTT

CCATGGTCAAAAAATGGCAGACAATGATTGAAGCTCACGTTGATGTCAAGACTACCGATGGTTACTTGCTTCGTCTGTTC

TGTGTTGGTTTTACTAAAAAAAACAATCAGATACGGAAGACCTCTTATGCTCAGCACCAACAGGTCCGCCAAATCCGGAA

GAAGATGATGGAAATCATGACCCGAGAGGTGCAGACAAATGACTTGAAAGAAGTGGTCAATAAATTGATTCCAGACAGCA

TTGGAAAAGACATAGAAAAGGCTTGCCAATCTATTTATCCTCTCCATGATGTCTTCGTTAGAAAAGTAAAAATGCTGAAG

AAGCCCAAGTTTGAAGACGAGACAGGTGCTAAAGTTGAACGAGCTGATGGATATGAACCACCAGTCCAAGAATCTGTT--

-GGGCGGCCCCTGGAGATGATTGAGCCGCGCACGCTACAGTACAAGCTGCTGGAGCCAGTTCTGCTTCTCGGCAAGGAGC

GATTTGCTGGTGTAGACATCCGTGTCCGTGTAAAGGGTGGTGGTCACGTGGCCCAGATTTATGCTATCCGTCAGTCCATC

TCCAAAGCCCTGGTGGCCTATTACCAGAAAGTGGATGAGGCTTCCAAGAAGGAGATCAAAGACATCCTCATCCAGTATGA

CCGGACCCTGCTGGTAGCTGACCCTCGTCGCTGCGAGTCCAAAAAGTTTGGAGGCCCTGGTGCCCGCGCTCGCTACCAGA

AATCCTACCGA---AGATATGCTCATGTGGTGTTGAGGAAAGCAGACATTGACCTCACCAAGAGGGCGGGAGAACTCACT

GAGGATGAGCGTGTGATCACCATTATGCAGAATCCACGCCAGTACAAGATCCCAGACTGGTTCTTGAACAGACAGAAGGA

TGTAAAGGATGGAAAATACAGCCAGGTCCTAGCCAATGGTCTGGACAACAAGCTCCGTGAAGACCTGGAGCGACTGAAGA

AGATTCGGGCCCATAGAGGGCTGCGTCACTTCTGGGGCCTTCGTGTCCGAGGCCAGCACACCAAGACCACTGGCCGCCGT

GGCCGCACCGTGGGTGTGTCCAAGAAG---CTGAAAGTCCCCGAATGGGTGGATACCGTCAAGCTGGCCAAGCACAAAGA

GCTTGCTCCCTACGATGAGAACTGGTTCTACACGCGAGCTTCCACAGCGCGGCACCTGTACCTCCGGGGCGCTGGGGTTG

GCTCCATGACCAAGATCTATGGGGGACGTCAGAGAAACGGCGTCATGCCCAGCCACTTCAGCCGAGGCTCCAAGAGTGTG

GCCCGCCGGGTCCTCCAAGCCCTGGAGGGGCTGAAAATGGTGGAAAAGGACCAAGATGGCGGCCGCAAACTGACACCTCA

GGGACAAAGAGATCTGGACAGAATCGCCGGACAGGTG

>Capite

TCTCACAGGAAGTTCTCCGCTCCCCGTCACGGGTCTCTGGGTTTCCTTCCCAGAAAACGCAGTACAAGGCACCGTGGTAA

GGTGAAGGCCTTCCCCAAGGATGATCCCTCCAAGCCGGTTCATCTGACCTCCTTCCTGGCCTACAAGGCTGGAATGACTC

ATGTGGTGCGCGAGGTTGACAGACCTGGCTCCAAGTCACACAAGAAGGAAGTTGTGGATGCCGTCACTATTCTGGAGGCA

CCTCCCATGATTGCCATCGGTGTCATTGGATACATTGAGACCCCCAGAGGTCTCCGTGCCCTCAAGACCGTCTGGGCTGA

GCACTTGAGCGAGGAATGCAGACGCCGCTTCTACAAGAACTGGTACAGAAGCAAGAAGAAGGCTTTCACGAAGGCTTCCA

AAAAATGGCAAGATGAGGTTGGGAAGAAGGAGATCGAGAAGGATCTTGCCAAGATGAAGAAGTACTGCTCTGTCATCCGT

GTGATTGCTCACACCCAAATGAAGCTTCTGAAGAAGCGCCAGAAGAAGGCTCACATCATGGAGATCCAAGTCAACGGCGG

CACCGTCGCCCAGAAAGTTGATTGGGCCCGTGAGCATTTTGAGAAGCAGATCCCAGTTGACTCTGTCTTTGCTCCCGATG

AGATGATCGATTGCATTGGAGTCACAAAGGGTCACGGGTTCAAGGGCGTCACCTCCAGGTGGCACACCAAGAAGCTGCCC

CGCAAGACCCACAAGGGTCTGCGTAAGGTTGCCTGTATTGGAGCCTGGCATCCGGCCCGTGTTGCTTTCTCTGTTGCCCG

TGCT---AGGCCTCTGGTATCAGTACACAATGAGAAGGGTGAGAACATCACTCTGCCTGCCGTCTTCAAGGCACAGATCA

GACCTGACATCGTCAGTTTCGTCCACTTCGAGATGAAGAAGAATGGACGTCAGCCATACGCTGTGTCCGAGAAGGCAGGT

CACCAGACCTCTGCTGAGTCATGGGGAACTGGTCGCGCTGTGGCCCGTATTCCACGTGTGCGTGGTGGCGGTACCCACAG

ATCTGGCCAGGGTGCTTTTGGTAACATGTGTCGTGGTGGTCGCATGTTTGCCCCCACCAAGACATGGCGCCGCTGGCACC

GTCGCATCAATATCAACCAGAGGCGTTACGCTATGTGCTCTGCCATTGCTGCCACTGGCATCCCCGCTCTGGTCATGTCA

AAGGGTCATCAAATCAACGAAATTGCCGAGGTTCCCCTGGTTGTTGACGACAAAATTGAAGAGTGCAAGAAGGCAAAGGA

GGCAGTGCTGTGCCTGCGCAAACTGAAGGCGTATGCTGATGTTGAGAAGGTCAAGGACTCCAACCAGGACAATGGTCTTG

TCAAAGCTTTCCGCAATATCCCCGGCATCACCACTCTTCCCGTTGACAAACTCAACCTCCTTAAAATCGCCCCTGGTGGA

CATGTGGGTCGTTTCTGCATCTGGACTGAGTCTGCATTCAAGAAGTTGGACGGTCTTTAC---CGCAGAGAGGGTAAAAC

CGATTACTATGCTCGCAAACGTCTTGTCATCCAGGACAAAGATAAATACAACACTCCAAAGTACCGCATGATCGTTCGCT

TCACCAACAAAGATGTTGTTTGCCAGGTAGCGTACGCCAGAATCGAGGGTGATTACATCGTGGCTGCGGCTTACGCCCAC

GAGTTGCCCAGATACGGCGTCAAGGTCGGGTTGACAAACTATGCGGCTGCATACTGCACTGGACTTCTGCTTGCACGCAG

AATTCTCAAGAAGTTCAAGTTGGACTCAATCTATGCTGGACAGAAGGATGTCGATGGTGAGGAGTACCATGTGGAGGAGG

ATGGACAGCCAGGTGCCTTCAGGTGCTACTTGGATGTTGGTCTTGCACGTACCAGCACTGGTGCCAAGGTGTTTGCTGCC

ATG---GTCAATCCCCTTTTTGAGAAGAGGCCCAAGAACTTCGGCATTGGTCAAGACATCCAGCCCAAGCGTGATTTGAC

CCGTTTTGTGAGATGGCCCAAGTATGTGCGCCTGCAGCGCAAGGCTACCCTTCTGAAGCGTCTGAAGGTCCCTCCCCCAA

TCAACCAGTTCAACCAGACCCTGGACAGGCAGACCGCTACCCAGATGTTCCGTGTCCTGGACAAATACAGGCCTGAGAGC

AAGCAGGAGAAGAAAGCTCGCCTGAGTGCCAGAGCTGAGGAACGCGTGAAGGGAGGCGCTGATGTGCCCACCAAGCGCCC

CCCAGTTGTCCGCTCGGGAATCAACACCATCACCTGCCTGGTTGAGCAGAAGAAGGCTCAGTTGGTCGTCATTGCACACA

ATGTCGATCCTCTCGAGGTGCGTCTTTTCCTGCCTGCTCTGTGCAGGAAGATGGGAGTTCCTTATTGCATCGTCAAGGAT

AAGGCTCGTTTGGGACGTGTCGTGAGACGCAAGACCGCCACTGCCCTCGCTCTGACCCACGTCAACGAGGACAGAAGCTC

CCTGAACAAGTTGGTCGAAGCCGTCAAATGCAACTACAATGACCGCGTTGATCAGATCAAGAAACACTGGGGAGGTGGCA

TCAACAGTTCCCGTTCACAGGCCAAA---ATGGGACGTGTGGTCTTCAAGGCACACAGCAAGCACAGGAAAGGACCAGCA

CTGCGTACTCTAGACTTCGCCGAGCGTCGTGGTTACATCAAGGGAGTTGTCAAGGATATCATCCATGACCCTGGCAGAGG

CGCTCCACTTGCCAAGGTGGCGTTCCGTGATCCATACAGATACAAGCAGCGAATGGAGACTTTCTATGCCGGCAAGAGAG

CCACTTTGACCGTTGGCAACATCCTGCCCGTTGGCCAGATGCCCGAGGGAACCATTGTGTGCGCTGTGGAGGAGAAGTCA

GGTGACCGTGGCAAACTGGCCAAGACCTCTGGCAACTACGCCACTGTCATCTCCCACAACCCTGACGCCAAGAAGACTCG

CGTGAAGCTCCCCTCTGGCGCCAAGAAACAATTGTCGTCCTCCAACAGAGCCATGATTGGAATTGTCGCTGGCGGTGGTC

GAATTGACAAACCCATGCTGAAGGCTGGTCGTGCCTACTACAAGTACAAGGCTAAGCGTAACTGCTGGCCTAAAGTGCGT

GGTGTGGCTATGAACCCCGTTGAGCATCCCCACGGAGGTGGAAACCATCAACATATTGGTATGGCTTCAACTGTGCGCAG

A---TCCGGTGATCGTTTGACCCGTGCCGCTAAGGTGCTGGAGCAGCTGACGGGGCAACAGCCCGTCTTCTCCAAGGCCC

GTTACACTGTCCGATCCTTCGGTATCAGACGTAACGAGAAGATTGCCGTGCACTGCACAGTGCGTGGTGCTAAGGCCGAG

GAGATCCTGGAGAAGGGTCTGAAGGTGCGCGAGTACGAGCTGAGGAGACACAACTTCTCTGCC---ACTTTCAACAAGTA

CGTTCAGATCGGCCGTGTGACTCTCGTGGCCTACGGGCCTGACCAGGGAAACCTTGTGGTCATCATTGACGTCATTGATC

ACAACAGAGCCCTGGTTGATGGACCCTGCACTGGCGTCAAGCGCCAGGCCCTGCAGTTCAAGGGCATGCACCTGACGAAA

TTTGTCATTCCCGTGCCCCGTTCTGCTCGTACTTGCATCATTAAGAAGAAGTGGGAGAAGAGCGGCATCACTGAGAAGTG

GCAGGAGACGTTCTGGTCCAAGCGTCTCGACCGCTTCAAGCTGATG---TACAAGTACATGCAAGAGCTTGACCGTAAGA

AGCAGACGGACGCTATGCGTTTTCTGCTTCGCGTGAGGACCTGGCACCTTCGCCAGCTGAACAGCATTCATCGTGCTCCC

AGGCCTACTCGCCCCGAGAAGGCCAGGCGCCTTGGTTACAGGGCTAAGCAGGGATACGTCATCTACCGTGTGCGCATCCG

CCGCGGTGGTCGCAGAAGGAAGGCCCCCAGGGACCAGGTTTACGGAAAGCCCGTCAACCATGGAATCACTCAATTGAAGA

ACCAGAGGTCTATCCAGGCTGTTGCTGAGGTAAGTGTCGCTCGCAAGTGCCAAGAGCTGCGTGTCATCAACAGTTACTGG

GTTGCCCAGGATGCCACTTACAAGTTCTTTGAGGTAGTGATCGTCGATCCTTTCCACAAGGCCATTCGCCGCGACCCCAA

GATCCAATGGATCTGCGAGTCTAATGCCAAGCACCGTGAGATGCGAGGTCTTACCTCCGCCAACAAGAAATCCCGTGGTT

TGGGTAAGGGACACAGATACACCAAGACCACTGGTGGATCCCGTCGCGGCAACTGGAAGAGGCGCAACACCCTGCAACTG

CGCAGGAAGCGT---CGTCACTTGGCCAGAAGGACCGATGCCAAGTTCAACAAGATCATCCTCAAGAGACTCTTTATGAG

CCGCACCAACAGAGCTCCTCTGTCCATCCACAGACTGCGTTTGATGAAGAAGCCTGGTCGCGAGAACAAAGTCGCTGTGG

TCGTTGGAACCATCACCAATGATGTCAGAATCTTTGAGATCCCCAAAATGAAGGTGTGCGCCCTTCACGTCACTGAGGAG

GCTCGTGCTCGCATCATCAAGAGCGGTGGTGAGATCATGACCTTTGACCAGTTGGCCCTGAAGGCCCCCAAGGGACAGAG

GACCGTCCTCGTCCAGGGTCCCCGAAGCCAGCGTGAATCCCAGAAGCACTTTGGCGCTGCACCCGGTCTGCCCCACAGCC

ACTCCAAGCCCCAT---AGAGAATTCAAGGTGATTGGGCGCATGATGCCCTCGCCCAAGAACCCCAGGCCTGCTATGTAC

CAGATGCACATCTTTGCTCCTGATTACATTGTGGCTAAGTCTCGTTTCTGGTACCACGTCACTCAGCTGCGTAAGATGAA

GAAGACTGGTGGAGAGATTGTCTGCTGCCAGAGAGTGTATGAGAAGCGTCCTCTGACCATCAAGAACTTCGGCGTGTGGC

TGCGTTACGATTCCCGTAGCGGCACCCACAACATGTACAAGGAGTACAGAGACCTCACCACTGAGGCTGCTGTCACCCAG

TGCCGTGACATGGGTGCCCGCCACAGAGCCAGGGCTCACTCCATCCAGATCATGCGAGTGGCCCCCATTGCTGCTGGAAA

GTGCCGCCGAGCTGGCGTGAAGCAGTTCCACGACTCTGAGATCAAGTTCCCTCTTCCCCACAGAGTGAACAGGCTGCACC

ACCCTCGCTTCACCACAACCAGGCCACACACC---CTTCGGCTACAGAAGCGCCTTGCAGCGTCCGTGCTCAAGTGCGGC

AGAAACAAGATTTGGCTGGATCCCAATGAGACCAACGAGATCTCAAATGCCAACTCCAAGCAGAACATCCGCAAACTGGT

GAAGGATGGTTTGATCATCCGCAAGCCAGTGGCTGTGCACTCCCGTGCCCGTGTTCGCAAGAATGCCGAGGCTCGCAGGA

AGGGTCGCCATACTGGCACTGGAAAGAGGAAGGGTACTACCAACGCCCGTATGCCTCAGAAGGTCATCTGGATGAGACGT

ATGCGTGTCCTCCGCAGACTCCTCAAGCGTTACAGGGAAGCCAAGAAAATCGACAGACACATGTACCATATGCTGTACCT

CAAGTGCAAGGGTAATGTGTTCAAGAACAAGCGTGTCTTGATGGAGCACATCCACAAGAAGAAGGCTGAGAAGACCCGCG

CCAAGATGCTC---GACCGATATGTGAAAGTATGGAGGAAGCCCAGGGGTATTGACAACAGAGTGCGTAGGCGCTTCAAG

GGTCAGTACTTGATGCCCAACATTGGTTATGGCTCCAACGCTAAGACCAGACACGTCTGCCCTGATGGGTTCAAGAGATT

TGTCATCCACAATGTTCGGGATCTTGAGGTTCTTCTGATGCAGAACCGCACTTACTCGGCCGAGATTGCCCATGGTGTTT

CCAGCAAGCGCAGGAAGGATATTGTTGAGCGTGCCCAGCAGTTGTCCATCAAAGTTACCAACCCCAGTGCTCGTATT---

CAGCTCCGTGTGGCCAAAGTCACCGGTGGTGCTGCTTCCAAGCTCTCCAAGATGGTTCGCAAGTCCATCGCTCGCGTCAT

GACCGTCATCAACCAGACCCAGAAGGAGAACCTGCGAAAGTTCTACCGTGACAAGAAATACATGCCCAAGGACCTGCGCC

CCAAGAAGACCCGCGCCATGCGTCGTGCCCTCACTCCCCACGAGAGGAGCCTCAAGACGAGGAAGCAGGCGAGGAAAGAC

AGCCTGTACCCTCTGCGCAAATATGCCCTCAAA---TCCAAGCAGATGCAGCAGATCCGTATTGCTCTCAGGGGCTCAGT

TGTCCTCATGGGCAAGAACACCATGATGCGCAAGGCCATCCGTTCCCACTTGGAGAACAACCCTTCTCTTGAGAAGCTCC

TCAACTACATCAAAGGCAACGTTGGCTTTGTCTTCACCAAGGGAGATCTCACTGAAGTGCGTAAGATCATCCAGGAGAAC

CGCGTGGGTGCCCCTGCCAAGGCTGGTGCTCTCGCTCCTCTCAGCGTCATGGTTACCGCTCGCAACACTGGTCTCGGCCC

AGAGAAGACCTCTTTCTTCCAGGCTTTGGCCATCCCCACTAAGATTACCAAGGGAACCATTGAAATTCTCAATGATGTTC

AACTGATCAAGGAAGGAGAGAAAGTTGGAGCTTCTGAGGCCACCCTTCTGACCATGTTGAAGATTATGCCTTTTTCATAC

GGTCTGGTCATCCAAGTCTACGACTCTGGCACCGTGTTCCATCCATCCATCCTGGACATGAGTGATGATGACATCAGAGG

CAAGTTCATGTCTGGAGTGAGCAGAGTGGCCAGTCTGTGCCTG---ACCCGTGCCGGTCAGCGTACCCGCTTCAAGGCTT

TTGTCGCCATTGGTGACTTCAACGGCCATGTTGGTCTGGGTGTCAAGTGCTCAAAGGAGGTGGCCACTGCCATCCGTGGT

GCCATCATCCTCGCTAAACTGTCCGTCATCCCTGTGCGCAGAGGTTACTGGGGAAACAAGCTGGGAAAGCCCCACACCGT

TCCCTGCAAGGTTACCGGCAAGTGCGGCAGTGTTGTTGTGCGTTTGATCCCTGCCCCACGTGGAACTGGCATCGTCAGTG

CTCCCGTTCCCAAGAAGCTGCTGACCATGGCTGGAATTGACGATTGCTACACTTCCGCTAGCGGACAGACTGCCACCCTT

---CTGGCTGAAGATGGTTACAGTGGTGTTGAGGTCCGTGTGACCCCCACTCGCACAGAGATCATCATCCTGGCCACCCG

TACCCAGAACGTTCTCGGTGAGAAGGGCAGGAGGATCCGTGAGCTGACATCTGTCGTCCAGAAGAGGTTCAACTTCCAGG

AGGGTACCGTTGAGCTGTATGCTGAGAAGGTGGCTACCCGTGGTCTGTGCGCCATTGCTCAGGCCGAGTCTCTTCGTTAC

AAGCTGATCGGTGGTCTGGCTGTGCGCAGGGCTTGTTATGGTGTGCTTCGCTTCATCATGGAGAGCGGCGCCAAGGGCTG

CGAAGTCGTTGTTTCTGGCAAGCTGCGAGGCCAGAGGGCCAAGTCGATGAAGTTCGTCGATGGCCTGATGATCCACAGTG

GAAACCCCACCAACGACTACGTTGACAAGGCTGTGCGCCACGTGCTCCTGCGCCAGGGAGTGCTCGGAATCCAGGTGAAA

ATCATGCTTCCCTGGGATCCCACCGGCAAGATCGGACCCAAGCGCCCCCTGCCGGATCATGTCAGCATCGTTGAGCCCAA

GGACGAG---GGTCCGCGGATGCATTTGAAGCGCCTTGCAGCGCCCAAGCATTGGATGCTGGACAAACTTGGAGGTGTTT

TCGCCCCACGTCCCAGCACTGGTCCCCACAAGTTGCGTGAATGTTTGCCGCTGGTGATTTTCCTGAGGAATCGTCTGAAG

TACGCTCTGACCTACACAGAGGTCATGAAGATTCTGAATCAGAGGTTGATCAAAGTGGACAACAAAGTGAGGACTGACAA

GACCTACCCCGCTGGATTCATGGATGTCATCTCGATCGAGAGGACCAACGAGAACTTCCGTCTCATCTACGATGTGAAAG

GCAGGTTTGCCGTCCACAGAATCACCCCAGAGGAGGCCAAGTACAAGCTGTGCATGATCACTGGAGGTCGCAATTTGGGT

CGTGTGGGTCTGATCACCCACAGGGAGAAGCATCCCGGCTCATTCGACATCATCCACGTGAAGGACACGACCGGACACAT

CTTTGCCACCAGGATGAGCAACGTGTTTGTGATTGGCAAGGGAAACCGAGCGTGGGTCTCGCTGCCCAAAGGAAAGGGAG

TCAAGCTGAGCATTGCTGAGGAGAGGGACAAGCGTCTGGCCAGC---CTAAATATTTCCTATCCAGCCACTGGCTGCCAG

AAGCTGATTGAAGTCGACGATGAGCGCAAGCTGAGACATTTCTACGAGAAGCGCATCTCTGCCGAGGTGAACGCTGACCA

TTTGGGCGATGAGTGGAAGGGATACGTGTTGAGGATCTCTGGCGGCAATGACAAACAGGGCTTCCCCATGAAGCAGGGTG

TGCTGACAGTTGGACGTGTTCGTTTGCTGCTGTCCGCTGGACACTCTTGCTACCGCCCTCGTCGTTCTGGCGAGAGGAAA

AGGAAGTCTGTTAGGGGTTGCATCGTGGATGCCAACCTCAGCGTTCTTTCTCTGGTCATTGTCAAGAAAGGTGAGCAGGA

GATTCCCGGTCTGACCGACTCCTCTGTGCCCCGTCGCTTGGGACCCAAGAGAGCGAGCAAGATCCGCAAACTCTTCAACC

TGGCCAAGGACGATGATGTGCGCCAGTACGTCGTGAAGAAGCCCAAGTCCAAGGCCCCCAAGATCCAGCGCCTGGTCACT

CCCCTTGTGCTG---ATCTCCCAGGACAAATGGCACAAGAGGAGGAAGACTGGTGGGCATATGCCCATCATGCGCAAGAA

GAGGAAGTTCGAGCTCGGAAGACCCGCAGCCAACACCAAGGTACTCGTCCGCATAAGGGGTGGTGGATACAAAATGAGGG

CCCTGCGCTTGGAAACAGGCAACTACTCCTCTGAGGCCCAGACCCGCAAGACCCGTCTCTTGGACGTTGTCTACAATGCC

AGCAACAACGAGTTGGTCAGAACCAAGACCCTTGTGAAGAACTGCATTGTGCAGATTGACGCCACACCATTCAGACAGTG

GTATGAGGCTCACTATGCTGTTCCCCTCGGCAGGAGGAAGGGCATGAAGGCTTGTGTCACGTCACGCCCCGGCATCCACG

GTCGTTGTGATGGTTACATTCTCGAAGGCAAGGAGCTCGAATTCTATGCCAGGAAAATCAAGGCCAAGAAA---AAAACT

TACGCGACACCTCGTCGTCCCTTCGAGAAGGAGCGTCTTGACCAGGAGTTGAAGATCATCGGCGAGTTCGGTTTGCGTAA

CAAGCGCGAGGTATGGCGTGTGAAGTACACCCTTGCCAAGATCCGTAAAGCCGCTCGTGAGCTGCTCACCCTGGACGAGA

AGGATCCCAAGCGTCTGTTCGAGGGCAATGCCCTGCTCAGACGTCTCGTGCGCATTGGAGTGCTCGACGAGGGAAAGATG

AAGCTTGATTACGTGCTCGGTCTGCGCGTGGACGATTTCTTGGAGAGGCGTCTGCAGACCCAGGTCTTCAAGCTGGGACT

CGCCAAGAGCATTCACCACGCTCGTGTGCTCATTCGCCAGAGACACATCCGGGTGCGCAAGCAGGTTGTGAACATCCCCT

CCTTTGTGGTGCGCCTGGATTCCCAGAAGCACATCGACTTCACCCACAACTCTCCATACGGCTGTGGA---TTCCAGAAG

CAGAAGGGGATCTTCCTCAACAAGAAGCGTTACGTCAAGAATGTTGGCCTTGGATTCAAGACTCCCAGAGAAGGCACATA

CATCGACAAAAAGTGCCCCTTCACCGGCAATGTCAGCATCCGTGGTCGCATTCTCAGCGGCATTGTCATGAAGACCAAGA

TGCAGAGGACCATTGTCGTGCGCAGGGATTACTTGCACTATGTGCGCAAGTACAACCGTTTTGAGAAGCGCCACAAGAAC

ATTTCTGCCCATTGCAGTCCTTGCTTCAGGGATGTGGCTATTGGAGATATTGTCACGATGGGTGAATGCAGACCTCTCAG

CAAGACAGTC---GGTATTTCCCAGTCTGCTCTGCCTTACAGAAGGACAGTGCCCACCTGGCTGAAACTGTCTTCTGACG

ATGTCAAGGAACAGATCTTCAAGCTGGCCAAGAAGGGTCTGACCCCCTCTCAGATTGGTGTCATCCTCAGGGATTCCCAC

GGAGTTGCTCAAGTGCGTTTCGTCACTGGCAACAAGGTCCTCCGTATCCTCAAGTCAAAGGGTCTCGCCCCTGAGATCCC

CGAGGATCTGTACCACCTCATCAAGAAGGCTGTCTCCATGAGGAAGCACCTCGAGAGGAACAGGAAGGACAAGGATACCA

AGTTCCGTCTGATTTTGGTGGAGAGCCGTATCCACCGTTTGGCCAGATACTACAAGACCTCCAAGGTCCTTCCCCCCAAC

TGGAAA---ATGGTGCGCGTGAACGTTCTAGCGGATGCCTTGAAATCTATCTGCAATGCAGAGAAGCGTGGCAAGAGACA

GGTGCTCATTAGGCCCTGCTCTAAAGTTATCGTCCGCTTCCTCACTGTAATGATGAAGCATGGTTACATTGGTGAGTTCG

AGATCATCGATGACCACAGGTCAGGCAAGATTGTGGTCAACCTCACTGGACGACTGAACAAATGTGGAGTCATCAGTCCT

AGATTCGATGTCAGAATGAGTGACTTGGAAAAGTGGACCACAAACCTCCTCCCCTCCAGGCAGTTTGGGTATCTGGTTTT

GACTACCTCCGGAGGCATCATGGATCACGAGGAAGCCAGACGAAAGCACCTTGGAGGAAAAATCCTTGGCTTCTTCTTC-

--GAACCCACCATCCACAGAATCCGCATCACTCTCACCAGCCGCAATGTGAAGAGCCTGGAGAAGGTGTGCGCAGATCTG

ATCCGTGGTGCTAAGGACAAGAACCTGAAGGTGAAGGGCCCCGTACGCATGCCCACCAAGGTCCTTCGCATCACTACCCG

CAAGACCCCCTGTGGAGAGGGCTCCAAGACGTGGGATCGCTTCCAGATGCGCATCCACAAGCGCCTCATCGATCTGCACT

CCCCGTCTGAGATCGTGAAGCAGATCACTTCCATCAGCATTGAGCCTGGTGTAGAGGTCGAGGTCACCATCGCCGAT---

ACGATTAGAACTCGCAAGTTCCTGACCAACCGCCTGCTTAACAGGCGTCAAATGGTAGTGGATGTCCTTCATCCTGGACG

CGCCACCGTACCCAAGACCGAGATTCGCGAGAAGCTCGGTCGCATGTACAAGACAACCGCTGACGTTGTGTTCTGCTTTG

GATTCAAGACCCAGTTCGGTGGCGGAAAGACCACTGGATTCGCTCTGATCTACGACACTTTAGATTACGCCAAGAAGCTC

GAGCCCAAGTTCAGACTTGCAAGG---GGTAAGCCACGTGGTTTGAGGACAGCTCGCAAACAGAAAAACCACCGCCGCGA

ACAACGCTGGCACGATAAGGATTACAAGAAGTCTCACTTGGGAACCCGCTGGAACCCCTTCGGAGGTGCTTCTCACGCCA

AGGGAATCGTGCTCGAGAAAGTGGGTGTTGAGGCCAAACAGCCCAACTCTGCCATCCGTAAGTGCGTCAGGGTCCAACTG

ATCAAGAACGGAAAGAAGATCACAGCCTTCGTACCCCGTGATGGTTGCTTGAACTACATTGAGGAGAATGACGAGGTTTT

GGTAGCTGGATTCGGTCGCAAGGGTCACGCCGTTGGAGATATCCCTGGTGTGCGTTTCAAGGTGGTGAAGGTCGCCAACG

TGTCTCTGCTGGCTTTGTACAAGGAGAAGAAGGAGAGGCCCAGGGCTATG---CGTGATGCCCGCAAGCATGGCAACTTC

TACGTGCCCCCAGAGCCCAAGGTTGCTTTCGTGATGCGTATTCGAGGGGTCAACGGTGTTGCTCCCAAGCCCCGTAAGGT

CATGCAGCTGTTCCGTCTTAGGCAGATCAACAACGGAACCTTCGTCAAGTTGAACAAGGCCACGCTTCAAATGATCCGTC

TGGCCGATCCCTTCATCACCTGGGGGTACCCCAACCTGAAGAGCGTGCGTGAGCTCATTTACAAGAGAGGATTCGGCAAA

GTGAACAGAGTGCCTCTGGCTGACAACATCATTGAGGAGAAACTTATCATCTGTATGGAGGATCTGATCCACGAGATCTT

CACCTGCGGCCCCAACTTCAAGGCTGCCAACAACTTCCTGTGGCACTTCAAACTGAACACGCCCACTGGAGGCTGGAGAA

AGAAGAACAACCATTTCGTTGATGGTGGTGACTTTGGCAACCGG---GAAGACAAGAAGCGCAAGTTTACCGAGACTGTG

GAACTGCAGATCTCCTTGAAGAACTACGATCCCCAGAAGGATAAGCGTTTCGCTGGTACCGTCAAGCTGAAGCACATTCC

CCGTCCTCGCATGAAGGTCTGCATCCTGGGCGATCAGCAGCATTGTGATGAAGCCAAGGTCAACAACCTTTCCAACATGG

ATGTCGACTCTCTGAAGAAGCTGAACAAGAACAAGAAATTGGTCAAGAAGCTTGGTCGGAAGTACAGTGCTTTCCTCGCA

TCTGACTCCCTCATCAAGCAGATCCCCCGTATCTTGGGACCTGGTCTGAACAAGGCTGGCAAGTTCCCCACACCCCTGAG

TCATGGAGAATCCATGTCTGCTAAGGTCGAGGAGGTTAAAGCCACCATCAAGTTCCAGATGAAGAAGGTCCTCTGTCTGT

CTGTTGCCGTTGGACACGTCCAAATGAGCGCTGATGAGCTGACCTCCAACATCATTCTGTCGGTCAACTTCCTGGTTTCT

CTGCTGAAGAAGAACTGGCAGAACGTCCGCGCTCTGTACATCAAGAGCACCATGGGCCCCGCTATCCGCATCTTC---TC

TCATTTTCACAAGCACTGGCAAAGGAGAGTAAAGACCTGGTTCGCCCAGCCTGCCCGTAAGGACCGCAGAAGGACTCTGC

GTGCTGCCAAAGCCGCTGCTATCGCACCTCGTCCCGTTGCTGGCATCAGGCCCATGGTCCGTTGTCCTACCTTCAGGTAC

CAGACCCAGGTCAGAGCTGGTAGAGGATTCACCTACGAGGAGCTTAAGGTTGGCATCAACAAGAGAGAGGCCCGCTCGAT

CGGCATCTCTGTTGACCACCGTCGCAAGAACCGATCCGTTGAGTCTCTGCAGCATAATGTGCAGCGTCTGAAGGAGTACA

AATCCAAGCTGATCCTCTTCAGGAAGGCGTCCGCACCC---ATGACCCGTTACTCGCAGGACGCCGAGAATGCCACAAAA

GCGGCGAAGGCCAAGGGATCTCATCTCCGTGTTCACTTCAAAAACACGCGCGAGACAGCTAATACCATCAAGCGTATGCA

TCTGCGTCGTGCCGTTGCTTTCCTGAAGAATGTCACCCAGATGAAGGAGTGCGTACCCTTCCGTAGGTACAATGGAGGTG

TCGGACGTTGCGCCCAGGCAAAGAACTGGAACACCACCCAAGGAAGGTGGCCCAAGAAGAGTGCCGAGTTCTTGCTGCAG

CTTCTCAAGAATGCTGAGAGCAACGCCGAATACAAGGGCCTGGACACTGATCACCTTGTGATTGAGCACGTGCAGGTGAA

CCGCGCTCCTAAGATGCGCAGGAGGACGTACAGAGCGCACGGCCGCATCAACTACATGAGCTCCCCCTGCCACATTGAGA

TCATCTTG---ATGAAGATTAACAAGCTGGTGACGTCCTCCAGGAGGAAGACCCGAAACAGGTACTTCAATGCTCCCTCT

CACCTGAGGCGCAAGATCATGAGCGCTCCCCTCTCCAAGGAGTTGAAACAGAAGTACAACGTGCGCAGCATCCCCATCAG

GAAAGACGATGAGGTCCAGGTAGTGCGTGGTCACTACAAGGGACAGCAGGGCAAGATCGTGCAGGTGTACCGAAAGAAGT

ATGTCGTGCACATCGAGCGCATCCAGAGAGAGAAGGCCAACGGTGCCTCAGTCCATGTGGGAATCCACCCCTCAAAACAG

GTTGTCGTGAAACTGAAGATGGACAAGGATCGCAAGAGCATCCTGGAGCGCAAGGCTCGTGGCAAGGGCAAG---TCAGG

CAAGGTTGTCCTTGTCTTGGGCGGACGCTATGCTGGCAGGAAAGCTATCATTGTAAAGAACTACGATGATGGCTCAAGTG

ACAAACCATTCGGCCATGCTCTCGTTGCTGGCATCGAGCGATACCCTCGCAAGGTGACCAAGTCAATGGGCAAGAAGACC

CTGAAGAAGAGGTCCAAGATAAAGACCTTCGTCAAGGTCTACAACTACAACCATCTTATGCCCACTCGGTATTCCGTGGA

TGTCAGATACAAGTCGGGCAAGAACAAGTGGTTCTTCCAGAAGCTTCGATTC---CTGAGAGGCCACGTCAGCCATGGGC

ATGGACGTATTGGCAAGCACAGGAAGCATCCTGGTGGTCGTGGTAACGCTGGTGGCCAGCACCATCACAGAATCAACTTC

GACAAATACCATCCTGGTTACTTCGGTAAAGTAGGTATGCGTTACTTCCACAAGACCCAGAACAAGTTCTTCTGCCCAAC

CATCAACGTGGAGGCCCTGTGGACCCTGGTCACTGAGCAGAAGCGTCTGAAGGCCCCCGTCATTGACTGCAACAGAGCTG

GTGTCTACAAGGTGCTCGGCAAGGGTAACCTGCCCAAGCAGCCTGTCATTGTGAAGGCCAAGTTCTTCAGCAGCAAAGCT

GAACAGAAGATCAAGGAAGTTGGTGGCTGCTGTGTCCTCACTGCT---ATGGCGGCGCACAAGAGTTTCCGTATCAAGAA

GAAGTTGGCAAAGAAGCAGAAGCAGAACAGACCCATTCCCCAATGGGTTCGCATGCAGACTGGTAACACCATCAGGTACA

ATGCCAAGAGGCGTCACTGGAAGCGTACTAAGCTGAAGTTG---ATGGCTCGTACGAAGCAAACCGCTCGCAAGTCCACC

GGAGGCAAGGCCCCCAGGAAGCAACTGGCCACCAAGGCCGCTCGCAAGAGCGCCCCGGCCACCGGAGGCGTAAAGAAGCC

CCACAGATACAGGCCCGGAACCGTCGCTCTGCGTGAGATTAGGAGATACCAAAAGTCCACCGAGCTGCTCATCAGGAAGC

TGCCCTTCCAGCGTCTGGTCCGTGAAATCGCCCAGGACTTCAAGACTGACCTGCGCTTCCAGAGCTCCGCCGTCATGGCT

CTTCAGGAGGCCAGCGAGGCCTACCTGGTCGGTCTGTTCGAGGACACCAACCTGTGCGCCATCCACGCCAAGCGTGTGAC

CATCATGCCCAAGGACATCCAGCTGGCCCGCCGTATCCGTGGCGAGCGCGCT---CTTGCTTGCGTTTACAGCGCCCTGA

TCCTCGCCGATGATGAAGTTCCCATCACCAGTGAGAAGATCTCAACCATCCTGAAGGCTGCCAAGGTTGCAGTTGAGCCA

ATCTGGCCC---ATGCGTTACGTGGCTGCATACTTGCTTGCTGTGTTGGGTGGCAACAGAGCCCCCCAGGAGGCTGACAT

CAAGGCCATCCTCAGCAGCGTTGGCATTGACGCCGACAGCGCCAACATCAAGAAGGTGATCAGTGAGCTGAAGGGCAAGA

GCATTGAGGACGTCATTGCTGAAGGC---GACAAGGAGTGGGTTCCCGTCACCAAGCTGGGACGTTTGGTAAAGGACATG

AAGATCAAGACGCTGGAGGAGATCTACCTCTTCTCCCTGCCCATCAAGGAGTTTGAGATCATCGACTACCTGGCTACCCT

TAAGGATGAGGTGCTGAAGATCATGCCCGTCCAGAAGCAG---AAGTATCTGCCCCACTCTGCTGGCCGATACCAGGTTA

AGCGTTTCCGTAAGGCTCAGTGCCCGATTGTGGAGCGTCTTGCTTGCTCCCTCATGATGCACGGGCGCAATAACGGAAAG

AAGATCATGGCCATGCGCATTGTGAAGCACGCCTTTGAGATCATCCATCTGCTCACCTCCGAGAACCCCCTCCAGGTCCT

CGTCAACGCCATCATCAACAGTGGCCCCCGTGAGGACTCCACTCGTATCGGTCGCGCTGGTCATGGAGCGCGTGAGGCTG

CCTTCAGGAACATCAAGACCATCGCTGAGTGCTTGGCTGATGAGCTCATCAATGCCGCCAAGGGGTCCTCCAACTCTCAC

GCCATCAAGAAGAAGGATGAG---TCGGCGAAGATTGTGAAGCCTCGTGATGAGAAACCCGACGAGCTTGAGTCGCAAGT

GTCTCAGGCTCTGTTGGAGTTGGAGATGAACAGTGACCTCAAGGCTCAGCTGAGAGAACTCCACATCACTGGCGCCAAGG

AGGTTGATGTGGCTGACAAGAAGGCCATTATGATCTTTGTGCCAGTCCCCCAGCTGCGTGCGTACCAGAAGATCCAGGCA

CGTCTCGTGCGTGAGTTGGAGAAGAAGTTCAGCGGCAAATACGTAGTCTTCATTGCGCAGCGTCGTATCCTGGCCAAGCC

CACGAGGAAGATGGTTCGCACAAAGAGCAAGCAGAAGAGGCCCCGTACCCTGACCACTGTCCACGAAAACATTCTCGATG

ATCTCGTGTTCCCCGCTGAGATTGTTGGAAAGAGAATTCGCATCAAGTTGGACGGATCTCGTTTGATCAAGGTTCACTTG

GACAAGACCCAACAGACAAACATTGAACACAAGCTGGATACCTTCACTGCAGTCTACAAGAAGCTGACTGGCAAGAGCGT

GACTTTTGAATTCCCCGAATTTGTTCTC---ATGCCCAAGAAGAACCGTGTGGCCATCTATGAGCACCTCTTCAAGGAGG

GTGTCATTGTCGCCAAGAAAGACTTCCGTGCTCCCAAGCACCCCGAATTGGAGGTGCCCAACCTGCACGTCATCAAGGCC

CTCCAGTCACTGAAGAGCAGAGGTCTTGTGAAGGAGCAGTTTGCATGGAGGCATTACTACTGGTACCTGACGAATGAGGG

TATCCAATACCTGAGGGACTTCCTCCATCTGCCCCCTGAGATTGTGCCCGCAACTCTCAAA---CATGTCACCGATCTGT

CTGGCAAGGAAACCATCGTCCGAGTGACGGGCGGTATGAAGGTGAAGGCCGACAGAGATGAGGCCTCCCCCTACGCCGCT

ATGTTGGCAGCTCAAGATGTGGCTGAGAGGTGCAAGGTGATTGGCGTGACTGCCCTTCACATCAGACTGCGTGCCACGGG

TGGCAACAGGACCAAGACCCCTGGACCCGGAGCCCAGTCCGCTCTCCGTGCTCTGGCCCGTTCCGGCATGAAGATCGGCC

GCATTGGTGAGCTA---AAGAGGAAGAAGAGGACCTTCCGTAAATACACCTACCGTGGTGTGGACCTCGACCAGCTCTTG

GACATGTCCAGTGAGCAGCTGATGGAGATGTTCCCCTGCCGTGTCCGTAGGCGCATGACCCGTGGTCTGAAGCGCAAGCC

CATGGCTCTGATCAAGCGTCTGCGCAAGGCCAAGAAGGATGCTCCAGCTCTGGAGAAGCCTGAGTGCGTGAAGACCCATC

TGCGTAACATGATCATCGTTCCTGAGATGATCGGCAGCATCGTTGGCGTCTACAACGGAAAGACCTTCAACCAGGTTGAG

ATCAAGCCTGAGATGATCGGACATTACTTGGGTGAGTTCAGCATCACATACAAACCTGTGAAGCACGGTCGCCCTGGTAT

TGGAGCCACTCACTCTTCACGTTTCATCCCTCTCAAG---ATGATTGAGAAGTACTACCCTCGTCTTACTCATGATTTCC

ACACCAACAAGCGCATTGTTGAGGAGATCGCTATCGTTCCTAGCAAGAAGCTCCGCAACAAGATTGCTGGGTTCATCACC

CATTTGATGAAGCGAATTCAGAAGGGATCCGTGCGTGGAATCTCCATCAAACTGCAGGAGGAGGAGCGTGAACGCAGGGA

TAACTTTGTGCCTGAGACTTCTGCCATCGACTGCGACATCATCGAGGTTGATATCGACACCAAGGAGATGTTGAAGATG-

--CCCAGAAAGTGCTCCACGAGCAACCGCATCATCGGAGCCAAGGACCATGCCTCCATTCAGCTGAACCTGGCTGAGGTT

GATGAGACCACTGGACGTATGACCGGCCAATACAAGACATATGCCATCTGTGGCGAGATCCGCAGAATGGGAGAGTCTGA

TGACAGCATTTGCCGACTGGCAAGAAGGGATGGTGTCGTTGCCAAG---CACACCAAGCCAGTGCGTTGCACCAACTGCG

CTCGTTGCGTGCCCAAGGACAAGGCCATCAAGAAATTTGTTATTCGCAACATTGTTGAGGCGGCTGCCGTCCGTGATTTG

ACCGAGGCTTCCGTGTACACAGAGTATGCTCTGCCTAAGCTGTATGCAAAACTCCACTACTGCGTGAGCTGCGCAATCCA

CAGCAAGGTTGTGCGTAATAGGTCTAAGGAGAGCCGCAAGGACAGAACCCCTCCCCCAAGGTTCAGGCCC---ATGATCT

TCGTCAAGACCCTCACGGGCAAGACTATCACTCTCGAGGTCGTCAAGGCTAAGATTCAGGAGGGAATCCCTCCCGATCAG

CAGAGGTTGATCTTTGCTGGCAAGCAGCTTGAGGATGGCGACATCCAGAAGGAGTCGACCCTCCATCTGGGTGGTGCCAA

GAAGCGCAAGAAGAAGAATTACACGACCCCCAAGAAAAACAAGCACAAGAAGAAGAAGGTCAAGCTCGCGGTACTCAAGT

ACTACAAGGTTGACGAGAACGGCAAAATTACTCGTCTGCGCAGGGAGTGCCCCAACGACGAGTGCGGAGCCGGAGTCTTC

ATGGCCTCTCACTTCGATCGCCAGTACTGTGGAAAGTGCTGCCTCACTTTTGTCTTCAACAAG---AAGCCATACCCCAA

GTCTCGTTTCTGCAGGGGTGTGCCTGATCCCAAGATCCGTATCTTCGATTTGGGTCGCAAGAAGGCGCGTTCTGATGACT

TCCCTCTTTGCGTGCATCTCGTGTCTGATGAGTGGGAACAGATCTCCTCTGAGGCTCTGGAGGCCGGCCGTATCTGCGCC

AACAAGTACTTGACCAAGCACTGTGGCAAAGACGCTTTCCATCTGCGTGTGCGCCTCCATCCCTTCCACGTTGTGCGCAT

CAACAAGATGTTGTCCTGTGCTGGAGCTGATAGGCTCCAGACCGGTATGCGTGGTGCCTTTGGCAAACCCCAGGGCACTG

TCGCCCGTGTGCACATTGGTCAGGTAATCATGTCCGTGCGCGCCAGGGACACTCACACCGCTAAAGTGATTGAGGCCCTG

AGGAGAGCCAAGTTCAAGTACCCTGGACGCCAGAAGATCTACATCAGCTCCAAATGGGGATTCACC---AAACGCACGAA

GAAGGTGGGAATCTGCGGCAAGTACGGCACCAGATATGGTGCCTCACTCCGTAAGGACATCAAAAAGATGGAAATCTCCC

AGCACAGCAAATACACATGTTCTTTCTGCGGCAAGGAGAACATGAAGAGGAAGGCTGTTGGTATCTGGAAGTGCAAGAGC

TGCAAGAAAACCGTCGCTGGTGGCGCCTACGTGTACAGCACCAACGCTGGGGCTACCGTCAGGAGCACCGTGCGTCGTCT

GCGTGAGAAC---CACATCCAGAAGAAGAAATGCTCTCGTTGTGCATATCCTGAAGCTAAAATGAGGAAGTATAACTGGA

GTGAGAAGGCTAAGCGCAGGAGGACACAAGGTGTTGGCCGCATGAGGTACCTCAAGAAGGTCTTCAGGAGATTCAGGAAT

GGATTCAGAGAGGGAACAGTC---AAGGGCAAGGACTCAATCTGTGCCCAGGGTAAGAGGCGTTACGACAGGAAACAGTC

CGGATACGGAGGACAGACCAAACCTATCTTCCACAAGAAGGCCAAGACTACCAAGAAGATTGTGTTGAAGATGGAGTGCA

CTGAGTGCAAGTTCCGCAAGCAGCTCCCCATCAAGAGATGCAAGCATTTCGAGTTGGGAGGTGACCGCAAGAAGAAG---

ATGACCAAGCACAACAAGTTCATCCGGGACGTTGTCAGGGAGGTTTGCGGTTTTGCTCCGTACGAGAGAAGAGTGATTGA

ATTGCTGAGGATCTCCAAGGACAAGAGAGCTCTCAAGTTCTGCAAGAAGAGGGTCGGAGGTCACGTCCGTGGCAAGAAGA

AGCGTGAGGAGATGCAGTCTGTCATTATGCAAATGAGGAAGGCTGCA---AAGGCTGTTGAGAGCATCAACTCTCGTCTT

GCTCTTGTGATGAAGAGTGGTAAATATGTCCTCGGCTACAACCAAACCCTGAAGAGCCTGCGCCAGGGCAAGGCTAAGCT

TGTCATCATTGCCAACAACACTCCTCCCCTGAGGAAGAGTGAGATTGAGTACTATGCCATGTTGGCCAAGACAGGAGTCC

ATCACTACAATGGAACCAACATTGATCTTGGAACCGCCTGCGGAAAGTACTACAGGGTTACCACCCTGAGCATCACTGAC

CCTGGT---CGCAAGATCAAGGGATTCAAGGATCACAAGCGCAAGATCCGTTCCTCCATCACCCCAGGAACCATCCTCAT

CCTGGTCGCTGGACCACACAAGGGGAAGAGAGTTGTTTTCCTGAAGCAGTTGGCATCCGGTCTCCTGTTGGTCACTGGTC

CTTACTTTGTCAATGGTTGCCCCTTGAGGCGCATCAACCAGATCTACGTCATTGCCACTAAGACCAAGTTGAACATCGAC

AGCGTCACTTTGCCCGATCGGTTGACTGATGACTACTTCCGTCGTCAGAAGGGTGAGGGTGATATCTTCGAATCCAAGAA

GGAGTACTCCGTGTCTGAGGAGCGCAAGGAGGATCAGAGCTCAGTGGATAAGCAGTTGTTG---GTGACGGTTAAGGGAC

CCAGGGGTACCCTGTCCCGTAACTTCAGCCACTTGGACATCCGAATGGTCCGCACTGTCTGCTCTCACATCGAGAACATG

ATCAAGGGAGTCATCGGCTACCGTTACAAGATGCGCTCCGTGTACGCTCATTTCCCCATCAACATCGCCATCGCTGAAGA

CAAGAAACACGTTGAGGTGCGTAACTTCTTGGGAGAGAAGTACACTCGTCACGTGAAGATGCTGGGAGATGTCGTCATTG

AGGCTTCATCCACCACCAAGGATGAGTTCATTCTGGAAGGAAACGACATCGAGCTGGTCTCTCGCTCAGCTGCTCTGATC

CAACAGTCAACCACTGTA---GTGTGCCTGAGGGCTGTTGGAGGTGAGGTTGGTGCGACATCAACCCTGGCCCCAAAGAT

TGGTCCCCTTGGTCTGTCCCCCAAGAAGGTGGGAGATGACATCGCCAAGGCTACTCAGGAGTGGAAGGGTCTAAAGATCA

CTGTCCAGTTGACGATTCAGAACCGTCAGGCCAAGGTCTCGGTGGTCCCCTCGGCGTCCTCGCTAGTCATCCGGGCTCTC

AAGGAGCCCCCACGTGACCGCAAGAAGCAGAAGAACATTAAGCACTCGGGAAACATTCCCTTCGACGAGATCCTGGCCAT

TGCACGTACCATGCGTCCTCGCAGCATGTCCCGCAAGCTGTCTGGCGTCTGCAAGGAGATCCTCGGCACATGCCAGTCCG

TCGGCTGCACAGTAGACGGATGTCACCCC---GATGGTAGGGGTCACCTTATGGGTAGGCTCGCTACCCTTGTGGCCAAA

TCACTGTTGCAAGGTCAACGCGTTGTGATTGTGAGATGTGAGGGAATCAATATTTCTGGTACCTTCTACAGGAACAAGTT

GAAGTACCTGGCTTACCTTCGCAAGAGGATGAACACAAACCCCTCCCGTGGCCCATTCCACTTCCGTGCTCCCAGCAAGA

TCTTCTACCGTGTTGTCAGAGGTATGCTGCCCCACAAACAGCACCGTGGACGTGAGGCTCTTGGTCGCCTGAAGGTCTTC

GAGGGCATCCCACCCCCATATGACAAGCAGAAGCGCGTTGTTGTTCCCTCTGCACTCAAGGTACTGCGTCTGAAGAGCCG

TCGTCCCTTCTGCAGCATTGGCCGTCTGTCCTCGGAGGTCGGCTGGAAATACCAGGGAGTTGTTGCTACTCTGGAG---A

TGACCAACACTAAGGGATATCGTCGTGGCACCAGGGACTTGTTCTCCCGTCGCTTCCGCAAGAAGGGTGTCATCCCTCTG

TCGACTTACATGATCCAATACAAGAGAGGAGATATCGTTGACATTAAGGGTTTCGGAGCGCAGCAGAAGGGTATGCCCCA

CAAGTCATACCATGGAAGAACCGGAACTATCTACAATGTGACGAAGCATGCTCTGGGTGTCATTGTCAACAAGAGAGTCC

GCATCATTCCTAAGCGCATCAATGTGCGTGTGGAGCATGTGAAGCAGTCCAAGTGCCGTCAGGATTTCTTGAACCGCGTC

AGTGCCAACGAAACTGCCAAGAAGGAGGCCAAGGAGTCCGGAAAGAAAATCAACTTGAGGAGACCTGTCCAACCCCATTT

CGTGACGAAGAACAACCTGCCCCAGGTGTTGGAGCCCATCCCTTACGAGTTCATCGCT---GTCACCAGTGAGGTGGTGA

CTCGTGAACACACCATCAACCTTCACAAACGCATCCATGGCGTGGGCTTCAAGAAGAGGGCTCCTCGTGCCCTGAAGGAG

ATCCGCAAGTTTGCTGAGAAGCACATGGGAACTCCTGATGTTCGTGTAGACACTCGTCTTAACAAGTTCATCTGGCAACA

TGGCATCAGCAACGTGCCCTTCCGTGTCCGCGTCAGGATGGCACGCAAGCGTAACGAGGATGAAGATTCTACCCACAAGC

TGTACACTCTGGTCTCCTATGTGCCAGTCACCACTTTCAAG---ACCGTGGTGAACCGAACCGTCGGAACAGAACTGGCT

GCGGATGGTCTTAAAGGCCGCGTCTTCGAGGTTTCCATGGCTGATCTTCAGGATGATGAGGTTGCTTTCCGCAAATTCCG

TCTGATTGCTGAGGAGGTCCAGGGCCGCCATGTGCTGACCAACTTCCATGGCATGAACATGACTACCGATAAAGTGCGAT

CCATGGTCAAGAAGTGGCAGACTTTGATCGAGGCCAATATTGATGTGAGGACAACTGATGGCTTCCTTCTCAGGATGTTC

TGTATCGGCTTCACCAAGCGTGTCAATCAAGTCAAGAAGACCTGCTATGCTCAGAGCAACCAGGTGCGCGCCATCAGAAA

GAAGATGGTTGAGATCATGACTCGCGAGGTGTCGGCCGCCGACCTCAAGGAGGTCGTCAACAAACTTATTCCAGACAGCA

TCGGCAAGGACATTGAGAAGGCCTGTATGGGCATCTACCCCCTGCATGACGTGTGCGTCCGGAAGGTCAAGATCCTCAAG

AAACCCAAGTTCGACACAGAAGGCGGCGAGAAGGTGGACCGGCCCGACAACTACGAGCCCCCGGTGCTGGAGTCTGTA--

-GGCCGCCCTCTGAACCAGCTGGAGCCCCAGATGCTCCGCTACAAGGTCCAGGAACCCCTTCTGCTTCTGGGCAAAGAGA

GGTTTGCTGGTGTTGACATCCGTGTGCGCGTGAAGGGAGGTGGACGTGTGTCTCAGGTGTACGCCATCAGACAGGCCATC

TCCAAGGCCCTCGTTGCCTACTACCAGAAAGTTGATGAGGCCTCCAAGAAGGAGATCAAGGACACCCTGGTCGCTTATGA

TAGAACTCTCCTGGTCGCTGACCCCCGTCGTTGTGAGCCCAAGAAGTTTGGTGGACCTGGCGCCCGTGCTCGCTACCAGA

AGTCTTACCGT---CGTTACTCTCACGTCGTCTGCAAGAAAGCCGATGTCGACCTCACCAAGCGAGCTGGAGAGCTTTCC

GAGGAGGAGAAGGTTATCACCATCATGTCAAACCCCCGCCAGTACAAGATCCCAGATTGGTTCTTGAACAGACAGAAGGA

CGTGAAGGATGGCAAATACAGCCAGGTCATGGCCAATATTCTCGACTCCAAGATCCGTGATGATCTCGAACGTCTGAAGA

AGATCCGTGCCCACAGGGGTCTCCGTCATTACTGGGGGCTGCGCGTGCGCGGTCAGCACACGAAGACGACCGGTCGCCGA

GGAAGGACCGTCGGTGTGTCCAAGAAG---TTAAAGGTCCCTGACTGGACTGACTTGGTCAAGCTGGGCAAGCACAAGGA

ACTCTCTCCATATGAGGAGGACTGGTACTACATCCGTGCTTCCCTGGCCCGTCACTTGTACATCCGGCCTTGCGGAGTTG

GTTCCTTCGCCAAGGTCTATGGAGGTCGCAAGAACAACGGCACCCGCCCCAGCCACTTCTGTGAGGGATCCTCCTCCATC

GCCAGGAAGGCGCTGCAGTCCCTGGAGGGCGTCAAGATGGTAGAGAAGGACCCAACTGGTGGCCGCCGCCTGTCCTCCCA

AGGTCGACGTGACCTTGACCGCATCGCCTCCCAAATG

>Dmelano

TCTCATCGTAAGTTCTCGGCACCCCGCCATGGCTCCATGGCCTTCTACCCCAAGAAGCGCTCAGCTCGCCATCGCGGTAA

GGTTAAGGCCTTCCCCAAGGATGACGCCAGCAAGCCAGTCCATCTGACCTGCTTCATCGGCTACAAGGCCGGCATGACCC

ACATTGTGCGCGAGGCCGATCGTCCTGGCTCCAAGATCAACAAGAAGGAGGTGGTCGAGGCCGTCACCGTTCTGGAGACC

CCGCCCATGATTGTGGTCGGTGCTGTCGGCTACATCGAGACTCCCTTCGGTCTGCGTGCCCTGGTCAACGTTTGGGCCCA

GCATCTCTCCGAGGAGTGCCGTCGTCGCTTCTACAAGAACTGGTACAAGAGCAAGAAGAAGGCATTCACCAAGGCCAGCA

AGAAGTGGACCGATGATCTCGGCAAGAAGAGCATCGAGAATGACTTCCGCAAGATGCTGCGCTACTGCAAGGTGATCCGT

GTGATTGCCCACTCGCAGATCCGCTTGATCAAGCAGCGCCAAAAGAAGGCCCATGTCATGGAGATCCAGCTGAACGGCGG

CTCCATCGAGGACAAGGTCAAGTGGGCTCGCGAGCATTTGGAGAAGCCCATCCAGGTCAGCAACGTCTTCGGCCAGGACG

AGATGATCGACTGCGTTGGTGTGACCAAGGGTAAGGGTTTCAAGGGTGTCACCTCGCGTTGGCACACCAAGAAGCTGCCC

CGCAAGACGCACAAGGGTCTGCGCAAGGTGGCCTGTATTGGTGCCTGGCATCCGTCGCGTGTGTCCACCACCGTGGCCCG

TGCC---AGGCCATTGGTCTCCGTGTACACGGAAAAGAACGAGAACATATGCCTGCCGGCAGTGTTCAAGGCGCCCATTC

GTCCGGATGTGGTCAACGAGGTTCACCAGCTGCTCCGCCGCAACAACCGACAGGCCTACGCCGTCAGCGAGCTGGCTGGT

CACCAGACCTCCGCCGAGTCCTGGGGTACCGGACGTGCTGTGGCCCGTATTCCCCGTGTGCGTGGTGGCGGTACCCACCG

TTCCGGCCAGGGAGCCTTCGGCAACATGTGCCGTGGTGGACGCATGTTCGCCCCCACCAAGACGTTCCGTCGCTGGCACC

GCAAGGTGAATGTCAACCAGCGTCGCTACGCCCTGGTGTCGGCCATCGCCGCCTCAGGAGTGCCAGCTCTGGTCCAGTCC

AAGGGACATGTCATCGACGGCGTCTCCGAGTTCCCGCTGGTCGTGTCCGATGAGGTGCAAAAGGTCCAGAAGACCAAGCA

GGCTGTCATCTTCCTGCGTCGCCTGAAGATCTGGGCTGACATCCAGAAGGTGTACAAGTCGGACAAGGACGAGGGTCTGC

GCAAGGCCTTCCGCAACATCCCCGGCATTGAGACCATCAACGTGGACAAGCTCAATCTGCTGAAGCTGGCCCCCGGCGGT

CATGTCGGTCGCTTTGTCATCTGGACCGAGTCGGCTTTCGCCCGCCTGAACGATCTGTTC---CGTCGCGAAGGAAAGAC

CGATTACTATGCCAGGAAACGCCTAACATTTCAGGACAAGAACAAGTACAACACTCCCAAGTACCGTTTGATCGTACGTT

TGTCCAACAAGGACATCACAGTACAGATCGCCTATGCTCGCATCGAGGGTGATCGCGTGGTGTGCGCTGCTTATTCCCAT

GAGCTTCCCAAATACGGGATCCAGGTTGGATTGACCAACTACGCTGCTGCTTACTGCACAGGCCTGCTGGTCGCCCGTCG

TGTCCTTAACAAGTTGGGACTGGACTCCCTATATGCAGGATGCACTGAAGTGACTGGTGAGGAGTTCAACGTCGAGGTTG

ATGACGGCCCAGGGGCATTCCGTTGCTTCTTGGATGTTGGACTCGCTCGTACTACAACTGGTGCCCGTGTGTTTGGCGCT

ATG---GTTAACCAGCTGTTCGAGAAGCGTCCCAAGAACTTCGGAATCGGCCAGAATGTGCAGCCCAAGCGCGATCTGTC

CCGTTTCGTTCGCTGGCCCAAATACATCCGGGTGCAGCGTAAGGCTGTGCTCCAGAAGCGCCTGAAGGTCCCACCACCAA

TCCACCAGTTCAGCCAGACTCTGGACAAGACCACCGCCGTGAAGCTGTTCAAGCTGCTGGAGAAGTACCGCCCCGAGTCG

CCGCTGGCCAAGAAGCTGCGCCTGAAGAAGATCGCCGAGGCTAAGGCCAAGGGCAAGGATGTGGAGCCCAAGAAGAAGCC

CAGCTATGTGTCCGCCGGTACCAACACGGTGACCAAGCTGATCGAGCAGAAGAAGGCCCAACTGGTGGTCATTGCCCACG

ATGTTGATCCTCTGGAGCTGGTGCTCTTCCTGCCCGCCCTGTGCCGCAAGATGGGCGTGCCCTACTGCATTGTGAAGGGT

AAGGCTCGTCTGGGTCGCCTGGTGCGTCGCAAGACCTGCACCACCCTCGCCCTGACCACCGTCGATAACGACAAGGCCAA

CTTCGGCAAGGTCCTGGAGGCTGTGAAGACCAACTTCAACGAGCGCCACGAGGAGATCCGTCGCCACTGGGGCGGTGGTA

TCCTTGGCTCCAAGAGTCTGGCCCGC---ATGGGTCGCGTTGTGTTCAAGGCGCACGTGAAGAAGCGCAAGGGAGCCGCC

CTGCGTTCCCTGGACTTCGCCGAGCGTTCCGGCTACATCCGCGGAGTTGTCAAGGACATCATCCACGATCCCGGCCGTGG

CGCTCCTCTGGCCGTCGTCCACTTCCGCGACCCCTACCGCTACAAGATCCGCAAGGAGCTGTTCTACTGCGGCCGCAAGG

CCACCCTTCAGATCGGCAACGTGATGCCCCTCAGCCAGATGCCCGAGGGTACCATCATCTGCAACCTGGAGGAGAAGACC

GGTGATCGCGGCCGTTTGGCCCGCACCTCTGGCAACTACGCCACCGTGATTGCCCACAACCAGGACACCAAGAAGACGCG

TGTCAAGCTGCCATCCGGCGCCAAGAAGGTCGTGCCCTCGGCCAACCGCGCCATGGTTGGCATCGTCGCCGGCGGCGGTC

GTATCGACAAGCCCATCCTGAAGGCCGGTCGTGCCTACCACAAGTACAAGGTGAAGCGCAACAGCTGGCCTAAGGTGCGT

GGTGTGGCCATGAACCCCGTGGAGCATCCTCACGGTGGTGGTAACCATCAGCACATTGGTAAGGCCTCCACCGTCAAGCG

A---TCCGGTGACAGGCTGACCCGTGCCGCCAAGGTGCTGGAGCAGCTGACTGGTCAGCAGCCAGTGTTCTCCAAGGCCC

GCTACACGGTCCGTTCGTTCGGTATTCGCCGTAACGAGAAGATCGCTGTCCACTGCACGGTGCGCGGCGCCAAGGCTGAG

GAGATTCTGGAGCGTGGCCTGAAGGTGCGCGAGTACGAGCTGCGTCGGGAGAACTTCTCCTCC---CCTTTCGAGAGATT

CGTACAAACTGGTCGCATTGCCAAGGCCTCCGCCGGTCCCCTGAAGGGGCGCCTGGTGGCCATTGTCGACGTCATTGACC

AAAACAGAGTTCTGGTAGATGGTCCTCTGACTGGCGTGCCCCGTCAGGAATACAGATTGAACAATCTGCATCTGACCAAG

TACCGCATCAAGTTCCCCTACACCGCGCCCACGCGCATCGTGCGCAAGGCCTGGACAGAGAGCGACCTGAAGGCCCAGTG

GAAGGTCAGCCCCTGGTCCGTCAAGGCAGACCGCTTCAAGCTGCGT---TATCGGTACATGCAGGAACTTTATAGGAAGA

AGCAGAGCGATGTGATGCGCTACTTGCTACGTATTCGCGTTTGGCAATACCGCCAACTAACGAAATTGCATCGTTCGCCA

AGACCTACTCGCCCGGATAAAGCAAGACGTTTAGGATACAGAGCCAAACAGGGGTTCGTGATTTATAGAATCCGTGTTCG

CCGCGGAGGTCGCAAGCGTCCAGTTCCCAAAGGATGCACTTATGGCAAGCCGAAGAGTCATGGTGTAAACCAGTTAAAAC

CATATCGTGGTTTGCAATCCATTGCTGAGGAACGTGTTGGTCGTAGACTTGGCGGCTTGCGAGTTTTGAACTCGTATTGG

ATTGCGCAAGATGCTTCTTATAAATATTTTGAAGTAATCTTAATTGATACTCATCACAGTGCTATTCGTCGTGATCCAAA

AATTAACTGGATCTGCAAGCATGTCCACAAGCATCGTGAATTGCGTGGCCTTACATCAGCTGGAAAAAGTTCGCGTGGCA

TTGGCAAGGGATATAGATACTCCCAGACAATTGGTGGATCTAGGCGTGCTGCTTGGAAGCGCAAGAACCGTGAGCACATG

CACAGAAAACGA---CGCTTCCTTCAGCGCCGCACCAACAAGAAGTTCAACCGCATCATCCTGAAGCGTTTGTTCATGAG

CAAGATCAACAGGCCGCCGCTATCGCTTCAGCGCATCCGCTTCTTCAAGGCCGCCAACCAGCCGGAGTCTACCATCGTGG

TCGTCGGCACCGTCACCGACGATGCCCGCCTCCTGGTGGTGCCCAAGCTCACCGTGTGCGCCCTGCACGTCACGCAGACC

GCCAGGGAGCGCATCCTGAAGGCCGGCGGTGAGGTCCTGACCTTCGATCAACTGGCTCTCCGATCGCCCACCGGCAAGAA

CACGCTGCTGCTGCAGGGCAGGCGTACCGCCCGCACCGCCTGCAAGCACTTCGGCAAGGCTCCCGGTGTGCCCCACTCGC

ACACCCGCCCCTAT---AAGGAGTACGAGGTCGTGGGCCGCAAGCTGCCCAGCGAGAAGGAGCCCCAGACTCCCCTCTAC

AAGATGCGCATCTTTGCCCCCGACAACATCGTGGCCAAATCCCGCTTCTGGTACTTCCTGCGCCAACTGAAGAAGTTCAA

GAAGACCACCGGCGAGATCGTGTCCATCAAGCAGGTGTACGAGACGTCGCCCGTGAAGATCAAGAACTTCGGCATCTGGC

TGCGTTACGATTCCCGCTCGGGCACCCACAACATGTACCGCGAGTACCGTGACCTGACTGTCGGCGGTGCCGTCACTCAG

TGCCGCGACATGGGAGCCCGCCACCGTGCCCGTGCCCACTCCATCCAGATCATTAAGGTGGACTCGATCCCTGCCGCCAA

GACCCGCCGCGTGCACGTGAAGCAGTTCCACGATTCAAAGATCAAGTTCCCTCTGGTCCAGCGTGTCCACCACGGCAACA

GGAAACTGTTCTCGTTCAGGAAGCCCAGGACC---CTAAAGCTCCAGAAGAGGCTCGCAGCCTCCGTGCTGCGATGCGGC

AAGAAGAAGGTCTGGTTGGATCCCAATGAAATCAACGAGATCGCTAACACAAACTCGCGTCAGAACATTCGCAAGCTTAT

CAAGGATGGTCTGATCATCAAGAAGCCCGTCGTGGTCCACTCCCGTTACCGTGTGCGCAAAAACACCGAGGCCCGCCGCA

AGGGACGTCACTGCGGATTCGGAAAGCGTAAGGGTACTGCGAACGCCCGCATGCCTACCAAGCTGCTGTGGATGCAGCGC

CAGCGCGTTCTGCGCCGCCTGTTGAAGAAGTACCGCGACAGCAAGAAGATTGACAGGCACCTGTACCACGACCTGTACAT

GAAGTGCAAGGGTAACGTATTCAAGAACAAGCGCGTCCTCATGGAGTACATCCACAAGAAGAAGGCTGAGAAGCAGCGCA

GCAAGATGCTG---GATCGATATGCTAAGCTGTGGCGCAAGCCCAAGGGTATCGACAACAGAGTGCGTCGCCGCTTCAAG

GGACAGTATCTGATGCCCAACATCGGTTACGGATCGAACAAGCGCACCCGCCACATGCTGCCCACCGGATTCAAGAAGTT

CCTGGTGCACAACGTGCGCGAGCTGGAGGTCCTGCTCATGCAGAACCGCGTTTACTGCGGCGAGATCGCCCACGGCGTCT

CCTCCAAGAAGCGCAAGGAGATTGTCGAGCGCGCCAAGCAGCTGTCGGTCCGCCTCACCAACCCCAACGGTCGCCTG---

AGCCTGCGCGTGGCCAAGGTGACCGGCGGATCTCCCTCCAAGCTCTCCAAGATCGTCCGCAAGGCCATCGCTCGCGTCTA

CATTGTGATGCACCAGAAGCAGAAGGAGAATCTGCGCAAGGTCTTCAAGAACAAGAAGTACAAGCCCCTGGATCTGCGCA

AGAAGAAGACCCGCGCTATCCGCAAGGCCCTGTCTCCGCGCGACGCCAACCGCAAGACCCTCAAGGAGATCCGCAAGCGC

TCCGTCTTCCCCCAGAGGAAGTTCGCCGTCAAG---TCCAAGCAGATGCAGAACATCCGTACCAGCCTGCGTGGAGCCGT

CGTGCTTATGGGCAAGAACACCATGATGCGCAAGGCCATCCGCGGTCATCTGGAGAACAACCCGCAGCTGGAGAAGCTGC

TACCCCACATCAAGGGCAACGTGGGATTCGTGTTCACCAAGGGCGATCTCGCCGAGGTGCGCGACAAGCTGCTGGAGTCC

AAGGTGCGCGCCCCCGCCCGTCCCGGCGCTATTGCCCCTCTGCACGTCATCATCCCGGCGCAGAACACCGGCTTGGGACC

CGAGAAGACCAGTTTCTTCCAGGCCCTGTCCATCCCGACCAAAATTTCCAAGGGAACAATTGAAATCATCAACGATGTGC

CCATCCTGAAGCCTGGCGACAAGGTCGGCGCCTCCGAGGCGACACTGCTCAACATGTTGAACATCTCGCCCTTCTCGTAC

GGTCTGATTGTCAACGTCTACGACTCCGGCTCGATCTTTTCGCCGGAGATCCTGGACATCAAGCCCGAGGATCTGCGCGC

CAAGTTCCAACAGGGAGTGGCCAACTTGGCCGCCGTTTGTTTG---ACCCGTGCTGGTCAGCGTACCCGTTTCAAGGCCT

TCGTTGCCATCGGCGACAACAATGGCCACATTGGTCTGGGCGTTAAGTGCAGCAAGGAAGTGGCCACCGCCATCCGTGGT

GCCATCATTCTGGCCAAGCTCTCCGTGGTGCCCGTGCGCCGTGGCTACTGGGGCAACAAGATCGGCAAGCCCCACACCGT

GCCCTGCAAGGTCACCGGCAAGTGCGGTTCCGTCTCCGTGCGCCTCATCCCCGCTCCCCGTGGTACTGGCATTGTCTCGG

CCCCCGTGCCAAGAAAGCTGCTGACCATGGCCGGTATTGAGGATTGCTACACCTCGGCCCGTGGCTCCACTGGAACCCTC

---CTCGCCGAGGATGGCTACTCCGGCGTGGAGGTCCGTGTGACCCCCTCTCGCACTGAGATCATCATCATGGCCACCAA

GACCCAGCAGGTGCTGGGCGAGAAGGGCCGTCGCATTCGGGAGCTGACCGCCATGGTGCAGAAGCGTTTCAACTTCGAGA

CCGGACGCATTGAGTTGTACGCCGAGAAGGTGGCCGCTCGTGGCCTGTGCGCCATTGCCCAGGCTGAGTCGCTGAGGTAC

AAGCTCACCGGAGGACTGGCCGTCCGTCGTGCTTGCTATGGTGTGCTCCGCTACATCATGGAGTCGGGAGCCAAGGGCTG

CGAGGTCGTCGTGTCCGGCAAACTGCGTGGTCAGCGTGCCAAGTCGATGAAATTCGTCGATGGCCTGATGATCCATTCGG

GAGATCCGTGCAACGACTATGTCGAGACCGCCACCCGTCATGTGCTCCTCCGCCAGGGAGTGCTTGGTATCAAGGTCAAG

GTCATGTTGCCCTACGACCCCAAGAACAAGATCGGCCCCAAGAAGCCGCTGCCCGACAATGTGTCCGTTGTGGAGCCCAA

GGAGGAG---GGCCCCAAGAAGCATTTGAAGCGTTTAGCCGCCCCCAAGGCATGGATGTTGGACAAGCTGGGAGGCGTCT

TCGCCCCGCGTCCCTCGACCGGTCCACACAAGCTCCGTGAGTCGCTGCCCCTGCTGATCTTCTTGAGAAACCGCTTGAAG

TACGCCCTCAATGGCGCCGAGGTCACCAAGATCGTCATGCAGCGCCTGGTTAAGGTCGATGGAAAGGTCCGCACCGACCC

CACCTATCCCGCTGGCTACATGGATGTCATCACCCTCGAGAAGACCGGTGAGTTCTTCCGTCTGGTCTACGACGTGAAGG

GACGCTTCGTCATCCACCGCATCTCCGCCGAGGAGGCCAAGTACAAGCTCTGCATGATCACCGGAGGCAGGAATTTGGGA

CGTGTCGGCACCGTTGTCAACCGTGAGCGTCATCCCGGTTCCTTCGACATTGTGCACATTAAGGACTCGCAAGGTCATGT

GTTCGCCACCCGTTTGACCAACGTGTTCATCATTGGCAAGGGCAACAAGCCCTACATCTCCCTGCCTAAGGGCAAGGGTG

TCAAGCTGAGCATTGCGGAGGAGCGCGACAAGCGTCTGGCCGCC---CTCAACGTTTCCTATCCCGCGACGGGATGCCAA

AAGCTATTCGAAGTGGTCGACGAGCACAAGCTGCGCGTCTTCTACGAGAAGCGTATGGGACAGGTTGTGGAGGCCGATAT

CCTCGGTGACGAGTGGAAGGGCTACCAGCTGCGCATCGCCGGCGGCAACGACAAGCAGGGATTCCCCATGAAGCAGGGTG

TCTTGACCCACGGCCGTGTGCGTCTGCTCCTGAAGAAGGGACACTCCTGCTACCGTCCACGCCGCACTGGCGAGCGTAAG

CGCAAGTCTGTGCGTGGATGCATCGTGGACGCCAACATGTCTGTGCTGGCTCTGGTCGTCTTGAAGAAGGGTGAGAAGGA

CATTCCCGGTCTCACCGACACCACCATCCCACGTCGCCTGGGACCCAAGCGTGCTAGCAAGATCCGCAAGCTCTACAACT

TGAGCAAGGAAGATGATGTGCGTCGCTTCGTTGTGAAGAAGGCCACCTCCAAGGCCCCCAAAATTCAGCGCCTGATCACC

CCCGTTGTGCTG---ATTAGCCGCGATAGTGCACACAAACGCCGGGCCACCGGAGGCAAGCGCAAGTCGCTCCGCAAGAA

GCGCAAGTTCGAGTTGGGACGCCCCGCCGCCAACACCAAGCTTAAGGTGCGCACCCGTGGTGGAAACACCAAGCTCCGTG

CTCTGCGCCTGGAAACCGGAAACTTCGCCTCCGAGGGAGTGGCGCGCAAGACCCGTATCGCCGATGTTGTGTACAACGCC

TCCAACAACGAGCTGGTGCGAACCAAGACCTTGGTGAAGAACAGCATCGTGGTCATCGATGCCACGCCCTTCCGCCAGTG

GTACGAGGCTCACTACGTGCTGCCCCTGGGACGCAAGCGTAACCCCAAGGCTTGCATTTCTTCCCGCCCCGGACAGTGCG

GTCGCTCCGACGGCTACATTTTGGAAGGCAAGGAGTTGGAATTCTACCTTAAGAAGATCAAGTCTAAGAAA---AAGACC

TACGTGACTCCCCGTCGCCCCTATGAGAAGGCGCGTCTGGACCAGGAGTTGAAGATCATCGGCGAGTATGGTCTGCGCAA

CAAGCGCGAAGTGTGGCGCGTCAAGTACGCCCTGGCTAAGATCCGTAAGGCCGCTCGTGAGCTGCTGACCCTCGACGAGA

AGGACGAGAAGCGTCTGTTCCAGGGTAATGCCCTGCTGCGCCGTCTGGTCCGTATCGGTGTCCTGGACGAGTCCCGCATG

AAGCTCGATTACGTGCTGGGTCTGAAGATTGAGGACTTCTTGGAGCGTCGTCTGCAGACGCAGGTGTTCAAGCTGGGACT

TGCCAAGTCCATCCATCATGCTCGCGTCCTGATCCGTCAGCGTCACATTCGTGTCCGCAAGCAGGTGGTCAACATCCCGT

CGTTCGTCGTGCGCCTGGACTCCCAGAAGCACATCGACTTCTCCCTGAAGTCGCCCTTCGGCGGCGGC---TTCCAAAAA

CAATTCGGTGTCAACCTAAACCGCAAGCGTCGCTCCCGCGATGTGGGACTCGGTTTCAAGACCCCACGTGATGGTACCTA

CATCGACAAGAAGTGCCCCTGGACCGGTGATGTGAGGATCCGTGGTCGCATTCTGACCGGCGTGGTCCGCAAGGCCAAGA

TGCAGCGCACCATTGTCATTCGGCGCGACTACCTGCACTTTGTGCGCAAATACAGCCGTTTCGAGAAGCGTCACCGCAAC

ATGAGCGTCCACTGCTCCCCTGTGTTCAGAGATGTTGAGCATGGCGATATTGTCACCATTGGTGAGTGCCGTCCTCTGTC

CAAGACTGTG---GGTATTTCCCAATCAGCCCTCCCCTACAGACGCACTGTCCCATCCTGGCTGAAACTGAACGCAGATG

ATGTCAAGGAGCAGATTAAGAAGCTGGGCAAGAAGGGTCTGACTCCCTCCAAAATCGGCATCATCCTGCGTGACTCGCAC

GGAGTTGCCCAGGTGCGTTTCGTCAACGGAAACAAGATCCTGCGCATCATGAAGTCGGTGGGTCTGAAGCCCGACATTCC

CGAGGATCTGTACCACATGATCAAGAAGGCCGTCGCCATCCGCAAGCACTTGGAGCGCAACCGCAAGGACAAGGACGGCA

AGTTCCGTCTGATTCTGGTCGAGTCCAGGATCCACCGCCTGGCCCGCTACTACAAGACCAAGAGCGTCCTGCCCCCCAAC

TGGAAA---ATGGTGCGTATGAACGTATTGGCCGATGCCTTGAAGTGCATAAACAACGCCGAGAAGCGTGGCAAGCGGCA

GGTGCTGCTGCGTCCCTGCTCCAAGGTGATCATCAAGTTCCTGACCGTGATGATGAAGCATGGCTATATCGGCGAATTCG

AGATCGTCGACGATCATCGTTCCGGCAAGATCGTTGTCAACCTGACCGGTAGGCTAAACAAGTGCGGCGTCATCTCGCCC

CGCTTCGATGTGCCCATCAACGACATCGAGAAATGGACCAACAATCTGTTGCCCTCGCGTCAGTTTGGTTACGTTGTGCT

CACCACCTCTGGCGGCATCATGGACCACGAGGAGGCTAGGAGAAAACATTTGGGAGGCAAAATTCTCGGCTTCTTCTTC-

--TCTGCCTCTGTGCACCGCATCCGCATCACCCTGACATCCAGGAACGTGCGTTCGCTGGAGAATGTGTGCCGCGACCTG

ATCAACGGTGCAAAGAACCAGAACTTGCGCGTCAAGGGCCCCGTGCGCATGCCGACCAAGACCCTTCGCATCACCACCCG

TAAGACTCCTTGTGGTGAGGGTTCCAAGACCTGGGATCGCTTCCAGATGAGAATCCACAAGCGCATCATCGACTTGCACT

CGCCCTCTGAGATCGTCAAGAAGATTACCTCCATCAACATCGAGCCCGGCGTAGAGGTTGAGGTCACCATCGCCAAC---

ACCATCCGCACCCGCAAGTTCATGACCAACCGCCTGCTCGCCAGGAAACAGATGGTCTGCGATGTCCTCCACCCAGGATT

GTCGTCGGTGAACAAGACCGAGATCCGCGAGAAGCTCGCCGCCATGTACAAGGTCACCCCCGATGTGGTCTTCGCCTTCG

GATTCCGCACCAACTTCGGTGGCGGCCGCTCCACCGGCTTTGCCCTCATCTACGACACCCTGGACTTCGCCAAGAAGTTC

GAGCCCAAGTACCGCCTGGCCCGT---GGCAAGCCAAGAGGTCTGCGCACTGCCAGGAAGCATGTGAACCACCGTCGCGA

CCAGCGTTGGGCCGACAAGGACTACAAGAAGGCTCATTTGGGCACCAGATGGAATCCCTTCGGAGGTGCTTCCCACGCCA

AGGGAATCGTCCTGGAGAAGGTCGGCGTCGAGGCCAAGCAGCCTAACTCTGCCATCCGCAAGTGCGTGAGGGTGCAGCTC

ATCAAGAACGGCAAGAAGATCACCGCCTTCGTGCCCCGTGACGGTAGCTTGAACTACATTGAGGAGAACGACGAGGTCCT

GGTTGCCGGTTTCGGTCGTAAGGGTCATGCCGTCGGTGATATTCCCGGTGTGCGCTTCAAGGTTGTCAAGGTCGCCAACG

TCTCCCTGTTGGCCCTCTACAAGGAGAAGAAGGAACGCCCAAGATCTATG---CGCTTGGCCAAGAAGCGCAACCAGTTC

TACGTGCCCGCCGAGGCCAAATTGGCCTTTGTCGTCCGTATCCGCGGTATCAACAAGGTGGCTCCCAAGGTCCGCAAGGT

TCTGCAGCTGTTCCGTCTGAGGCAGATCAACAACGGTGTGTTCATCAAGCTGAACAAGGCCACCATCAACATGCTGCGCA

TCGCCGAGCCCTACATCACCTGGGGCTATCCCAATCTGAAGTCCGTGCGGGAGCTGATCTACAAGCGTGGATTCGTGAAG

CATAACCGCGTGCCCATCACCGATAACGTGATCGAGCGGAAGCTGATTCAGTGCGTCGAGGATCTTGTCCATGAGATCTT

CACCGTGGGGCCCAACTTCAAGTACGCCTCCAACTTCCTGTGGCCCTTCAAGCTAAACACCCCCACCGGCGGCTGGCGCA

AGAAGGCCAACCATTATGTCAACGGTGGTGACTTCGGCAACCGC---TCGGCGAAGAAGAAGCGCTTCCTCGAGACGGTG

GAACTGCAGATCGGCCTGAAGAACTACGATCCCCAGAAGGACAAGCGTTTCTCCGGCACCGTCAAGTTGAAGCACATTCC

TCGTCCCAAGATGAAGGTGTGCATCCTTGGCGATCAGCAGCATTGCGACGAAGCCAAGGCCAACAACGTCGACTTCATGG

ATGCCGAGGCTCTGAAGAAGCTGAACAAGAACAAGAAGCTGGTGAAGAAGCTGGCCAAGTCCTATGATGCCTTCCTGGCC

TCTGAGTCGCTGATCAAGCAGATTCCTCGTTTGCTTGGCCCTGGCTTGAACAAGGCCGGCAAGTTCCCTGCCCTGCTGTC

GCATCAGGAGTCTATGATTGGCAAGATCGAGGAGGTTAAGTCGACCATCAAGTTCCAGATGAAGAAGGTGCTCTGCCTGT

CCGTCGCCGTCGGCCACGTTGGCATGAAGTCCGACGAGCTGGCCCAAAACGTCAACTTGTCGATCAACTTCTTGGTGTCG

CTGTTGAAGAAGAACTGGCAGAATGTGCGCTCCCTGCACGTCAAGTCCTCCATGGGACCGCCCCAGCGTCTTTAC---CA

GCACTACCACAAGTGGTGGCAGCGGCATGTGAAGACCTGGTTCAACCAGCCGGCCCGCAAGGTCCGCAGGCATGCGAACC

GCGTCAAGAAGGCTAAGGCCGTCTTCCCCCGCCCAGCCAGCGGTCTGCGCCCTGTGGTTCGCTGCCCCACCATCCGCTAC

CACACCAAGCTGCGTGCCGGCCGTGGTTTCACCCTGGAGGAGCTGAAGGCCGGCATTGGCGCCAACTTCGCCAAGACCAT

CGGCATTGCCGTCGACAGGAGGCGCAAGAACAAATCCCTGGAGTCCCGCCAGCGTAACATCCAGCGCCTCAAGGAGTACC

GCAGCAAGTTGATCCTGTTCAAGAAGATCCGCGCCGGC---ATGGGCCGTTACTCACGCGAGTCAGACAACGTGGCCAAG

TCGTGCAAGGCGCGCGGGCCCAATCTACGTGTGCACTTCAAGAACACACATGAGACCGCCCAGGCCATCAAGCGCATGCC

CCTGCGCCGTGCCCAGCGTTACCTGAAGGCCGTCATCGACCAGAAGGAGTGCGTGCCCTTCCGTCGCTTCAACGGAGGAG

TCGGTCGTTGTGCCCAGGCCAAGCAGTGGAAGACCACGCAGGGTCGCTGGCCCAAGAAGTCCGCCGAGTTCCTGCTGCAG

CTGCTGCGCAACGCCGAGGCCAACGCTGATTGCAAGGGTCTGGATGCCGACCGCCTGGTCGTCCACCACATTCAGGTGAA

CCGCGCTCAGTGCCTGCGTCGCCGCACGTACCGTGCCCATGGTCGCATCAATTACATGTCGTCGCCATGCCACGTCGAGG

TCATCCTC---ATGAAACAGAACCCGTTCGTGTCGTCGTCGCGCAGGAAGAACCGCAAGCGTCACTTCCAGGCTCCCTCC

CACATTCGCAGGCGCCTCATGTCCGCTCCCCTCTCCAAGGAGCTGCGCCAGAAGTACAATGTGCGTTCCATGCCCATCCG

CCGCGACGATGAGGTCCAGGTGATCCGCGGCCACTTCAAGGGCAACCAGGGCAAGGTGGTCCAGGCCTACCGCAAAAAGT

TCGTCGTCTACGTCGAGAAGATCCAGCGCGAGAACGCCAACGGCACCAACGTCTACGTGGGCATCCATCCCAGCAAGGTG

CTGATCGTCAAGCTGAAGCTCGACAAGGACCGCAAGGCCATCCTGGAGCGTCGCGGCAAGGGTAAGGGCAAG---CAGGG

CAAGATCGTAATCGTCCTTAGCGGACGTTACGCCGGTCGCAAGGCCATCATCGTCAAGACCCACGACGATGGAACCCCGG

AGAAGCCCTTCGGACACGCCCTCGTCGCCGGTATCGATCGCTACCCGCGCAAGGTGACCAAGAAGATGGGCAAGAACAAG

CTGAAGAAGAAGTCCAAGGTCAAGCCCTTCCTGAAGAGCCTGAACTACAATCATCTGATGCCCACCCGCTACACGGCGGA

CATCGTCTACAAGGAGGGCAAGAACAAGTGGTTCTTCCAGAAGCTGCGTTTC---CTGCGTGGTCATGTGAGCCACGGTC

ACGGCCGTATCGGCAAGCACCGCAAGCATCCCGGAGGTCGCGGTAACGCTGGTGGCATGCACCATCACCGCATCAACTTC

GACAAATACCATCCTGGTTACTTCGGCAAGGTGGGCATGAGGAACTTCCATCTGCGTCGCCAGCACAAGTTCAGGCCCGA

GATCAACCTGGACAAGCTCTGGTCCCTGGTCGGAGCCGAGAAGTTCGCCAAGGCTCCCGTCATCGACTTGGTTAAATTCG

GCTACTACAAGCTGCTGGGCCGTGGTCACCTGCCCGCCCGCCCCGTCATCGTGAAGGCCAAGTACTTCTCTAAGAAGGCT

GAGGACAAGATCAAGAAGGCCGGCGGTGTGTGCTTGCTGAGCGCC---ATGGCTGCACACAAGTCGTTCAGAATAAAGCA

GAAGCTGGCTAAGAAGCTGAAGCAGAACAGATCCGTTCCCCAATGGGTTCGCCTACGTACTGGCAACACTATTCGTTACA

ACGCTAAGCGCCGTCACTGGAGGCGTACCAAGTTGAAGCTG---ATGGCTCGTACTAAGCAGACTGCCCGTAAGTCGACC

GGAGGAAAGGCTCCTCGTAAGCAGCTAGCCACCAAGGCTGCCCGTAAATCGGCGCCATCCACCGGCGGAGTGAAGAAGCC

CCATCGTTATCGTCCCGGAACTGTGGCTCTTCGTGAGATCCGTCGTTACCAGAAGTCGACCGAGTTGCTCATCCGCAAGC

TGCCCTTCCAGCGTCTGGTTCGTGAAATCGCTCAGGATTTCAAGACCGATCTGCGTTTCCAGTCGGCTGCCATCGGTGCC

TTGCAGGAAGCATCTGAGGCGTACCTGGTGGGTCTCTTCGAGGACACCAACTTGTGCGCTATCCACGCCAAGCGCGTGAC

AATCATGCCTAAGGACATCCAGTTGGCGCGCCGCATCCGTGGCGAGCGTGCT---CTCGCCTGCGTCTACGCCTCCCTCA

TCCTCGTCGATGACGATGTCGCCGTCACCGGTGAGAAGATCAACACCATCCTGAAGGCCGCCAACGTCGAGGTGGAGCCC

TACTGGCCC---ATGCGTTACGTGGCTGCTTACCTTCTGGCCGTCCTCGGTGGCAAGGACTCGCCCGCCAACAGCGATCT

GGAGAAGATCCTCAGCTCTGTGGGCGTTGAGGTCGACGCCGAGCGTCTGACCAAGGTCATCAAGGAGCTGGCTGGCAAGA

GCATCGACGACCTGATCAAGGAGGGT---TCCAAGGAGTGGGTGCCAGTGACCAAGCTAGGACGCCTGGTGCGCGAGGGC

AAGATCAAGTCTTTGGAGGAGATCTACCTGTACTCGCTTCCCATCAAAGAGTTCGAGATCATCGACTTCCTGTCCTCGCT

GAAGGATGAGGTGCTGAAGATCATGCCCGTCCAGAAGCAG---CGCTATCTTCCCCATTCCGCCGGACGTTATGCCGCCA

AGCGTTTCCGCAAGGCCCAGTGCCCCATTGTGGAGCGTTTGACCTGCTCCCTGATGATGAAGGGTCGCAACAACGGCAAG

AAGCTGATGGCCTGCCGCATCGTCAAGCACTCGTTCGAGATCATTCATCTGCTCACCGGGGAGAACCCTCTGCAGATCCT

GGTCAGCGCCATCATCAACTCGGGACCCCGTGAGGACTCCACCCGTATTGGACGTGCCGGTACTGGAGCTCGTGAGGCTG

CCTTCAGGAACATCAAGACCATCGCCGAGTGCCTGGCTGATGAGCTGATCAACGCTGCTAAGGGATCTTCCAACTCGTAC

GCCATCAAGAAGAAGGATGAG---GGCTCCAAGATTATCAAGCCCGGCGGCTCGGATCCCGATGACTTCGAGAAGTCCAT

TGCCCAGGCGTTGGTGGAACTCGAGGCCAACAGCGACCTGAAGCCCTACCTGCGCGATCTGCACATCACCCGTGCCCGCG

AGATCGAGTTCGGCAGCAAGAAGGCCGTCATCATCTACGTGCCCATTCCACAGCAGAAGGTGTTCCAGAAGATCCAGATC

ATCCTGGTCCGCGAGCTGGAGAAGAAGTTCTCGGGCAAGCACGTCGTCGTGATTGCGGAGCGCAAGATCCTGCCCAAGCC

CACGCGCAAGGCCAACCCCCTCAAGCAGAAGCGTCCACGCTCCAGGACTCTGACCGCTGTGTACGACGCCATCCTTGAGG

ATCTGGTCTTCCCCGCCGAGATTGTGGGCAAGCGCATCCGCGTCAAGCTGGACGGCTCCCAGCTGGTCAAGGTGCACCTG

GACAAGAACCAGCAGACCACCATTGAACACAAAGTCGACACCTTCACCTCGGTCTACAAGAAGCTGACTGGTCGCGATGT

TACCTTCGAATTCCCCGACAACTACCTG---ATGCCAAAGGCCCATCGTGTCGCGATCTACGAGTACCTCTTCAAGGAGG

GCGTGATCGTGGCCAAGAAGGATTTCCATGCCCAGAAGCACCCGGAACTGGAGATCCCCAACTTGCACGTGATCAAGGCG

ATGCAGTCGCTCCACTCGCGCGGACTGGTTAAGGAGCAGTTCGCCTGGCGCCACTACTACTGGTACCTCACCAACGAGGG

AATCGAGGAGCTGCGCAGCTACCTCCACCTGCCGCCCGAGATCGTGCCCTCGACGCTGAAG---CATGTCACTGATCTGT

CCGGCCGTGAGACCATCGCTCGTGTCACCGGAGGCATGAAGGTGAAGGCCGATCGTGATGAGGCTTCGCCCTACGCCGCT

ATGTTGGCCGCTCAGGATGTGGCTGAGAAGTGCAAGACACTGGGCATTACTGCCCTGCATATTAAGCTGCGTGCCACCGG

CGGCAACAAGACCAAGACCCCCGGACCCGGCGCCCAGTCCGCTCTGCGTGCTTTGGCCCGTTCGTCCATGAAGATTGGCC

GCATCGAGGATGTG---CTGAAGAAGAAGCGTACCTTCAAGAAGTTCACCTACCGCGGTGTCGACTTGGACCAGCTTCTG

GACATGCCCAACAACCAGCTGGTGGAGCTGATGCACAGCCGTGCCCGCAGGCGTTTCTCCCGCGGACTGAAGCGCAAGCC

AATGGCTCTGATCAAGAAGCTGCGCAAGGCCAAGAAGGAGGCACCGCCAAATGAGAAGCCCGAGATTGTCAAGACCCACC

TGAGGAACATGATCATCGTACCCGAGATGACCGGCTCCATCATTGGCGTCTACAACGGCAAGGACTTCGGACAGGTGGAG

GTCAAGCCCGAGATGATCGGTCACTACCTGGGCGAGTTCGCCCTGACCTACAAGCCCGTCAAGCACGGTCGTCCTGGTAT

CGGTGCCACCCACAGCTCCCGTTTCATTCCTCTGAAG---ATCATCGAGAAGTACTACACTCGCCTGACGTTGGACTTCC

ACACCAACAAGCGCATCTGCGAGGAGGTGGCCATCATTCCCACCAAGCCCCTGCGCAACAAGATTGCCGGCTATGTCACC

CATTTGATGGGCCGCCTGCGTCACTCCCAGGTGCGTGGTATCTCCATCAAGTTGCAGGAGGAGGAGCGTGAGCGTCGTGA

CAACTACGTCCCGGCCGTCTCCGCTCTGGAGCAGGACATCATCGAGGTCGACGCCGACACCAAGGAGATGTTGAAGCTT-

--CCCCGCAAATGCTCGGCGTCCAACAGGATCATCCACGCCAAGGATCACGCCTCCGTGCAGCTGAGCATCGTGGATGTG

GACCCCGAGACCGGTCGCCAGACCGACGGTTCCAAGACCTACGCCATCTGCGGCGAGATCCGTCGCATGGGCGAGTCCGA

CGACTGCATCGTGCGTCTGGCCAAGAAGGACGGCATCATTACCAAG---CATGTGAAGCCCGTGCGCTGCACCAACTGCG

CCCGCTGCGTGCCCAAGGACAAGGCAATCAAGAAGTTCGTCATCCGCAACATCGTGGAGGCTGCCGCCGTGCGCGACATC

ACGGAGGCCAGCATCTGGGACTCGTACGTGCTGCCCAAGCTCTATGCGAAGCTCCACTACTGCGTGTCCTGCGCAATCCA

CTCCAAGGTGGTGCGCAACCGTTCGCGCGAGGCCCGCCGCATCCGCACGCCGCCACTGCGTTTCAAGCCC---ATGATCT

TCGTTAAGACCCTCACGGGCAAGACCATCACTCTTGAGGTCGTCAAAGCCAAGATTCAGGAAGGAATTCCTCCAGATCAG

CAGCGTCTGATCTTCGCTGGCAAGCAGCTGGAAGATGGCGACATTCAGAAGGAGTCCACTTTGCACTTGGGTGGTGCCAA

GAAGCGCAAGAAGAAGAACTACTCCACTCCCAAGAAAATCAAGCACAAGAGGAAGAAGGTCAAGCTAGCTGTCCTGAAAT

ACTACAAGGTTGACGAGAACGGCAAGATCCACCGTCTCCGTCGCGAGTGCCCTGGTGAGAACTGCGGCGCCGGCGTCTTC

ATGGCTGCCCACGAAGATCGTCACTACTGCGGCAAGTGCAACCTGACCTTTGTCTTCAGCAAA---AAGCCGTACCCTAA

ATCTCGGTTTTGCCGTGGTGTCCCTGATCCAAAAATTCGTATATTTGATTTGGGAAGAAAAAAAGCTACAGTTGAGGATT

TTCCCCTGTGTGTTCATTTGGTTTCTGATGAATACGAACAGCTGAGTAGTGAAGCTTTGGAAGCTGGACGCATTTGTTGC

AACAAGTACTTGGTGAAGTACTGCGGTAAGGACCAGTTCCACATCAGAATGCGCCTGCATCCATTCCACGTCATTCGCAT

CAACAAAATGTTGTCGTGCGCTGGAGCTGATAGGCTTCAAACTGGAATGCGAGGAGCGTTTGGAAAACCGCAGGGCACGG

TTGCTCGAGTTCGTATTGGTCAACCCATTATGTCTGTCCGCTCTAGCGATCGTTACAAGGCTCAAGTTATTGAAGCTTTG

CGGCGTGCTAAGTTTAAGTTCCCTGGACGTCAAAAGATCTATGTTTCAAAGAAGTGGGGATTCACC---AAGCGCACCAA

GAAGGTTGGAATCGTTGGTAAATATGGTACCCGTTATGGTGCCTCCCTGCGTAAGATGGTCAAGAAGATGGAAATCACCC

AGCACAGCAAGTACACTTGCTCCTTCTGCGGCAAGGACTCCATGAAGCGCGCCGTTGTGGGCATCTGGTCCTGCAAGCGC

TGCAAAAGGACCGTCGCCGGTGGCGCCTGGGTGTACTCCACCACCGCCGCCGCCTCCGTGCGATCCGCCGTCCGTCGTCT

GCGTGAAACC---CACATCCAGAAGTCCACTTGCGCCCAGTGCGGCTACCCCGCCGCCAAGTTGCGTTCCTACAACTGGT

CCGTGAAGGCCAAGAGGAGGAAGACCACCGGCACCGGTCGCATGCAGCACCTGAAGGTTGTGCGCCGCCGTTTCCGCAAC

GGATTCCGCGAGGGCACCCAG---AAGTCCAAGGAGCGCAAGGGAGCTCAGGGCAGGCGTCGTTACGACAGGAAACAGCA

GGGTTTCGGAGGTCAGACCAAGCCCATCTTCAGGAAGAAGGCCAAGACCACCAAGAAGATTGTGCTGCGTATGGAGTGCA

CCGAGTGCAAATACCGCAAGCAGACTCCCCTGAAGCGTTGCAAGCACTTCGAGCTGGGCGGTGACAAGAAGCGCAAG---

CAAACCCGCCACACCAAGTTCATGCGCGACTTGGTCCGCGAGGTCGTTGGCCACGCTCCCTATGAGAAGCGCACCATGGA

GTTGCTGAAGGTGTCCAAGGATAAGAGGGCCCTGAAGTTCCTCAAGCGCCGCCTGGGCACCCACATCCGTGCCAAGAGGA

AGCGTGAGGAGTTGTCCAACATCCTCACCCAGCTGAGGAAGGCCCAG---AAGGCTCTGGAGAGCACCAACGCCCGTCTG

GCGCTGGTGATGAAGTCCGGCAAATACTGCCTGGGCTACAAGCAGACCTTGAAGACCCTGCGCCAGGGCAAGGCCAAACT

GGTGCTCATCGCCAGCAACACGCCCGCCCTGAGGAAGTCCGAGATCGAGTACTACGCTATGCTGGCCAAGACTGAAGTCC

AGCACTACAGCGGCACCAACATCGAGCTGGGCACCGCCTGTGGTAAATACTTCCGCGTGTGCACCCTGTCCATCACCGAT

CCTGGA---TTCGTGAAGAAGCGCTTCAGCGAGCACAAACGCAACACGCGCCGCAACCTGACCCCCGGAACCGTGCTCAT

CCTGCTGGCCGGACGCCACCAGGGCAAGCGCGTCGTCCTGTTGAAGGTCTTGGCCTCCGGACTCCTGCTGGTCACCGGAC

CCTTCGCCCTCAACTCGTGCCCGCTGCGTCGCGTGTCCCAGCGCTACGTGATCGGCACCTCCTCGAAGGTGGATCTCGGT

GCCTTCAAGGTGCCCGAGCACCTGAACGACGCCTACTTCCGCCGTCTGAAGGGCGAGGCCGACATCTTCGCGGCCAAGAA

GGAGTTCGTGCCCAACGAGCAGCGCAAGAAGGACCAGAAGGAGGTGGACGCCGCCCTGCTG---GTGACCATCACCGGCA

CCCGCGGCACCCTGAAGCGCAGCTTCAAGCACTTGGACATGTACATGGTCCGCACCGTCTGCAGTCACATCGAGAACATG

ATCAAGGGAGTCACGGGATTCCAGTACAAGATGCGTGCTGTGTACGCCCATTTCCCCATCAACTGTGTCACCTCCGAGAA

CAACACGGTCATTGAGATCCGTAACTTCTTGGGTGAGAAGTACATCCGTCGTGTGGAGATGGCTCCTGGCGTCACCGTGG

TCAACTCCACTGCCCAGAAGGACGAACTTATCGTGGAGGGAAACGATATTGAGTCTGTCTCCGGATCTGCCGCCCTCATC

CAGCAGTCCACGACCGTC---GTGTACCTGCGTTGCGTGGGCGGAGAAGTCGGTGCCACATCCTCCCTGGCCCCAAAGAT

CGGTCCCCTCGGTCTGTCGCCCAAGAAAATCGGTGATGACATCGCCAAGGCCACCTCCGACTGGAAGGGTCTGAAGATCA

CCGTCTGCCTGACCATCCAGAACCGACAGGCCGCCATCTCCGTGGTTCCCTCCGCCGCCTCGCTGATCATCAAGGCTCTG

AAGGAACCCCCACGTGACCGCAAGAAGCAGAAGAACATCAAGCACAGTGGAAACATTGGCTTCGAAGACATTCTGGCCAT

CGCCCGCGTGATGAGGCCCAGGTCCATGGCCCGTGAGTTGAAGGGAACCTGCAAGGAAGTTCTGGGAACCGCCCAGAGCG

TCGGCTGCACCGTTGACGGAAAGCACCCT---GATGGTCGCGGCCATTTGCTCGGTCGCCTGGCCTCCGTGGTGGCCAAG

TACCTGTTGCAGGGCGGCAAGGTGGCCGTGGTCCGCTGCGAGGAGCTGAACCTCTCGGGACACTTCTACAGGAACAAGAT

CAAGTTCCTGGCCTACCTGCGCAAGCGGTGCAACGTGAACCCAGCCCGTGGTCCATTCCACTTCCGTGCCCCCTCTCGCA

TCTTCTACAAGGCAGTCCGAGGCATGATCCCACACAAGACCAAGCGTGGCCAGGCCGCCCTCGCCCGTCTACGTGTGTTC

GACGGCATCCCATCGCCCTACGACAAGCGTCGCCGCGTCGTCGTGCCCATCGCTATGCGTGTGCTGACCCTGCGCTCCGA

CCGCAAGTACTGCCAGGTGGGTCGCCTGTCGCACGAGGTCGGCTGGCACTACCAGGACGTGATCAAGAGCCTGGAG---A

TGACCAACTCAAAGGGCTATCGTCGCGGCACACGGGACATGTTTTCCCGTCCCTTCCGAAAGCATGGAGTTATTCCGTTG

TCCACATACATGCGGGTTTTCAAGATCGGCGACATCGTTGACATTAAGGGACATGGTGCTGTACAGAAGGGTTTGCCCTA

TAAGGCATATCATGGCAAAACCGGGCGCATATTCAATGTGACTCAGCACGCCGTTGGTGTAATAGTAAACAAACGCGTAA

AAATCCTTGCTAAGCGGGTCAATGTGCGCATTGAGCACATCCACCACTCCAAGTGCCGCGAAGATTTTTTGCGCCGCGTA

AAGGAAAATGAGCGATTGCTGAAGGAGGCGAAGGAAAAGGGACAATGGGTCAGTCTGAAGCGCCCGAAGAAGGCGCACTT

TGTTAAGAAACTTGAAGAACCGATTGCTCTGGCCCCGATTCCATACGAATTCATTGCC---GCGATCAACGAAGTCGTGA

CGCGCGAGTGCACCATTCACTTGGCCAAGCGTGTCCACAACATCGGCTTCAAGAAGCGCGCACCCCGCGCCATCAAGGAG

ATCCGCAAGTTCGCCGAGCGGGAGATGGGCACCACCGATGTGAGGATCGACACCCGTCTGAACAAGCACATCTGGTCCAA

GGGTATCAGGTCCACTCCATTCCGCATTCGCGTGCGCCTGGCGCGTCGCCGCAACGACGATGAGGACTCCCCCAACAAGC

TGTACACTTACGTGACCTATGTGCCGGTGTCCACGTTCAAG---ACACTTGTAAACCGCACCCAGGGTCAAAGAATAGCA

TCGGATTATTTGAAGGGTCGCGTTTTCGAAGTGTCTTTAGCAGACTTGCAAAAGGATATTGATTCTTTTCGCAAGTTCCG

TCTTATTGCAGAAGATGTTCAAGACCGTAATGTGCTCTGTAACTTTCACGGAATGGACTTGACTACGGACAAGTACAGGT

CGATGGTTAAAAAGTGGCAAACACTAATTGAAGCTATTGTCGAAGCAAAGACTGTAGATGGGTACCTCTTGCGAGTGTTT

TGTATCGGATTTACTGCCAAGCAGCAGTCTCAGCGCAAAACATGTTATGCTCAGCAATCGCAAGTCCGAAAGATTCGTGC

TCGCATGACCGACATTATTACTAATGAAGTTAGTGGTGCCGATCTAAAGCAGCTTGTTAACAAGCTGGCCTTGGACTCGA

TTGCGAAAGATATCGAAAAAAGCTGTCAGCGCATATATCCATTACATGATGTTTACATTCGTAAAGTAAAGGTATTGAAG

AAACCGCGCTTCGATTCATCCGAGGGAGCCGTAATCGACCGCCCTGAAGGTTATGAGCCCCCAGTACAGGAAGCTGTT--

-GGTCGTCCTCTGGAGCAGATTGAACCCAAGGTCCTGCAATACAAACTGCAGGAGCCCCTTCTGTTGCTCGGCAAGGAGA

AATTCGCCGGCGTCGACATCCGCGTGCGCGTTAGCGGTGGTGGTCATGTAGCCCAGATCTACGCCATCCGCCAGGCCATT

TCCAAGGCTCTCGTTGCCTTCTACCAGAAAGTCGATGAGGCCTCCAAGAAGGAGATCAAGGACATTCTGGTGCAGTACGA

CAGAACCCTGCTGGTCGGCGATCCGCGTCGCTGCGAGCCCAAGAAGTTCGGCGGTCCAGGTGCCCGTGCTCGCTACCAGA

AGTCGTACCGT---CGCTACTCCAACATTGTGCTGAAGAAGGCCGATGTCGATCTTACCAAGCGCGCCGGTGAGTGCACC

GAGGAGGAGAAGGTGGTGACCATCATCTCGAACCCTCTGCAGTACAAGGTGCCCAACTGGTTCCTCAACAGGCAGAAGGA

CATCATCGATGGCAAGTACTGGCAGCTGACCTCCTCCAACTTGGACTCGAAGCTGCGTGACGATCTGGAGCGTCTGAAGA

AGATCCGCTCCCACCGTGGTCTGCGTCACTACTGGGGCCTCCGTGTGCGTGGCCAGCACACCAAGACCACCGGTCGTCGT

GGTCGCACCGTGGGTGTGTCCAAGAAG---TTGAAGGTGCCCGACCAGATGGACATCGTCAAGACCGCCAAATTCAAGGA

GCTGGCGCCCTACGATCCCGACTGGTTCTATGTGCGTGCCTCGATCCTGCGCCATCTGTACCACCGCCCCGCTGGAGTCG

GTTCGATCACCAAGATCTACGGCGGACGCAAGCGCAACGGTGTCCACCCCTCGCACTTCTGCCGCGCCGCCGACGGTGCT

GCCCGCAAGGCTCTGCAGGCCTTGGAGCACGCCCGTTTGGTCGAGAAGCACCCGGACGGTGGTCGCAAACTGAGCTCCAT

TGGACAGCGTGATCTGGACCGTATTGCTAACCAGATC

>Argope

TCGCACAGGAAATTTTCAGCACCCCGTCATGGGTCGAAAGGCTTTCTACCGAAGAAGAGGGCCAGCCGTCATAGGGGCAA

AGTCAAGGCTTTCCCTACAGATGACAAAAGCAAGCCTGTCCATTTGACTGCTTTTATTGGTTACAAGGCTGGCATGTCCC

ACATTGTTCGTGATGTAGACCGACCTGGTTCGAAATCCAACAAGAAAGAAATTGTGGAGGCAGTTACCATCATTGAGACA

CCACCCATGGTCATCGTTGGTTTGGTTGGCTACATTGAAACACCAAATGGCCTGAGGTCATTCAAGACCATCTTTGCTGA

GCATCTGAATGAGGATTGCAGGAGGCGATTTTATAAAAACTGGTACAACTGCAAGAAAAAGGCATTTACCAAGTATGCCA

AAAAATGGACTGATGAGAGTGGAAAGAAGGAGATTGAACGTGACTTCCAGAAAATGAAGAAATACTGCAAGGTTATCCGT

GTCATTGCCCACACACAGACTAAGTTGATGAAGAAGCGTCAGAAGAAGGCACATATCATGGAGATCCAGGTAAATGGAGG

CACCATTGCACAGAAGGTGGACTGGGCTAGGGAGAAGATGGAATCCTCTGTCCCTGTCGGCTCAGTCTTTGCCATGGATG

AAAACATTGATATCATTGGTGTTACCAAGGGTAAAGGAGTCAAAGGTGTGACCTCCAGATGGCACACCAAAAAGTTGCCC

AGGAAGACGCACAAGGGTCTGAGAAAAGTTGCCTGTATTGGAGCCTGGCATCCAAGTCGTGTCCAGTTCACTGTTGCTAG

GGCT---AGACCATTGATCAGCGTTTATGATAATAAGGGAGAGAATGTCACCCTGCCATCAGTGTTCAGAGCCCCCATCA

GGCCAGACATAGTCTCATTTGTGCACTCTGAGATGAGGAAGAACTCCAGGCAGCCATACGCTGTCAGCAAATATGCTGGT

CACCAAACATCTGCCGAGTCTTGGGGAACTGGACGTGCTGTGGCTCGTATCCCCCGTGTACGTGGAGGTGGTACCCACAG

ATCTGGCCAGGGAGCCTTTGGTAACATGTGTCGTGGAGGACGTATGTTTGCCCCTACAAAGACCTGGCGTAGGTGGCACC

GTAGGATCAACATCAACCAGAGGCGTTACGCTCTGTGTTCAGCCATCGCTGCCACTGGCATCCCAGCCCTGGTGATGTCC

AAAGGTCACAGGATTGAGGAAACACAAGAAGTGCCATTGGTGGTCAGCGACAAAGTCCAGGAGTTTAAAAAAACAAAGGA

GGCAGTCAGTCTGCTGAAGACTCTCAAAGCATGGAGTGACATCCAAAAGGTATACAACTCAAGAGAAGATAATGGCATAT

CTAGGGCCTTCAGAAATATTCCAGGTATTACCCTCATAAATGTTTCACGTTTGAATCTTTTGAAAATCGCACCTGGTGGT

CATGTTGGACGTTTCTGTATTTGGACCGAAAGTGCATTCAGGCAACTGGATGATTTATAC---AGGCGTGAGGGCAAGAC

AGATTTCTATGCCAGGAAACGCCTCGTCATTCAAGAGAAAAACAAGTACAATACACCCAAATACAGGCTTATTGTTCGCT

TCACCAACAAGGACATCATATGCCAGATAGCATACGCCAGGATCGAAGGTGATCACATTATTTGCAGTGCATATGCACAT

GAACTGCCAAGATATGGCGTGAAGGTAGGGCTGACCAACTATTCTGCTGCTTACTGCACCGGATTGCTGCTTGCAAGAAG

GCTTCTTAAGCAGTTTAAACTGGACTCCGTGTACGCTGGCCAGGAAGAGGTGGACGGTGATGAGTACTACGTTGAGGTGG

ACGGTGAGGCTGGTGCCTTCAGGTGTTACCTGGACGCTGGTCTAGTCCGCACTTCCACTGGTGCACGTGTCTTTGGTGCA

TTG---GTTAATCCCCTTATTGAGAAACGACCAAGAAACTTTGGAATTGGTCAGGATATTCAGCCCAAGAGAGATCTTAG

CCGTTTTGTCAAGTGGCCCAAGTACATCCGTCTCCAGAGAAAGGCAGTACTCCTACAACGTCTGAAGGTTCCTCCACCAA

TCAACCAGTTCAAACAGACTTTAGATGGAAAGACCGCAACACAAGTTTTCCGTCTAATGGACAAGTACAAGCCAGAATCG

AAACAGGCCAGGAAGGCTAGGTTAAAGGCTCGTGCTGAGCAGCGTGCCAGCGGTGGTGAAGATGTACCAACAAAGCGTCC

CCCAGTTGTGAGGTCAGGAGTTAACAACATCACTTGTCTAGTGGAACAAAAAAAAGCACAGTTGGTCGTCATCGCCCATG

ATGTGGATCCATTAGAAATTGTGTTGTTCCTGCCCGCTCTCTGCAGAAAAATGGGAGTTCCCTACTGTATTGTGAAAGGA

AAGGCACGTTTAGGACGTGTTGTCAACAGGAAAAATGCCACCTGTCTGGCTCTCACAAGTGTCAACGAGGACAAGTCATC

ACTCAGTAAATTGGTTGAGGCTGTCAAGACCAACTTCAATGAAAGACATGAAGAGATCCGCCGTCACTGGGGTGGAGGAC

AGATGGGCAGCAAGTCCCAGGCCCGT---ATGGGTCGTGTAATCTTTAAGGCCCACACAAGAACCAGGAAAGGCAAGGCC

CTTAGACCAGTTGACTTTGCTGAACGTCATGGATACATCAAGGGTGTGATCAGGGACATCATCCATGACCCAGGCAGAGG

TGCTCCTCTCGCTAAGGTAGCATTCCGTGATCCCTACAAGTACAGACAAAGGACCGAGACTTTTTACTGTGGAAAGAAGG

CATCCCTTCAAATTGGAAACATTTTGCCTGTTGGAGTTATGCCTGAAGGAACCATTGTGTGTTGTCTTGAAGAAAAGGCA

GGAGATCGTGGTAAATTGGCCCGTGCCTCAGGAAATTACGCTACTGTCATCTCTCACAACCCTGACACCAAGAAAAGCCG

TGTAAAACTGCCATCTGGCTCAAAGAAAGTCATCCCATCAGCCAACAGAGCTATGGTTGGTATTGTTGCTGGAGGTGGAA

GGATAGACAAGCCCATGCTGAAGGCTGGTCGTGCTTACTTCAAGTACAAGGTCAAGAGGAACTGCTGGCCTAAAGTTCGT

GGTGTGGCTATGAACCCAGTTGAACATCCTCATGGTGGTGGTAACCATCAGCATATTGGTAAAGCATCCACAGTCAGACG

T---TCTGGTGACAGATTGACTAGGGCAGCCAAGGTGTTGGAACAGCTGACTGGTCAGCAGCCTGTTTTCTCTAAGGCCC

GTTACACTGTGCGTTCTTTTGGAATCAGAAGGAATGAAACCATCGCTGTGCACTGTACAGTCCGTGGAGCCAAGGCAGAG

GAGATCCTGGAACGTGGACTGAAGGTGAGAGAATACGAGTTGAGGAAGAACAACTTCTCAGCC---GGCCAGTTCCACTT

CGTAGAAATTGGGCGGGTGGCCCTTTTTGCCTATGGCCCAGATAAAGGCAAACTCTGCGTCATCGTAGATGTTATTGACC

AGAACAGGGCATTAGTTGATGGACCAGCCACAGGCGTTAAAAGGAAGCAGGTCAACTACAAATCTCTACATCTCACACAG

TTTAAGTTAAACATCGGTAGAGCCATGCGTACAGGAAACCTGCTGAAGGTCTGGAATAAAGAAAGCATGCAGTCCAAGTG

GGATGCTACATCCTGGGCCAAGAAGATTGACCGCTTCAAGTTGATG---TACAAGTACATTCAGGAGTTATACAGGAAGA

AGCAAACTGATGTCATGAGGTTTCTTCTTCGAGTGAGGTGCTGGCAATACCGTCAGTTGTCTGCTATCCATAGGGCCACC

CGTCCCACTCGACCAGACAAGGCCAGGAGGCTGGGATACAGAGCTAAACAAGGTTACGTTATCTACAGGGTCCGAGTTCG

TCGTGGTGGACGCAAACGCCCCGTTGCCAAGGGATGTACCTATGGTAAACCAAAGACCCATGGAGTAAATCAGCTGAAGT

TCCAACGGTCCCACAGGTCTATTGCAGAGGAACGTGTTGGTCGCAAGTGTGGAGCACTACGAGTCCTTAACTCCTACTGG

GTAGCCCAGGATTCCAGCTACAAATTTTTTGAAGTTATCCTTGTAGATACCTTCCATAAGGCTATCCGCAGAGACCCAAA

AATACAATGGATCTGCAAGGCTGTCCACAAACACAGGGAGCTTCGTGGTCTTACTTCTGCTGGAAAGAGCTCACGTGGCC

TTGGAAAGGGTCATGGATTCCACAAAACTACAGGAGGTTCTCGCAGGGCAAACTGGAAGAAGGGCAATTCTCTCAGTCTC

CGCAGAAAGCGT---AGGTTCCTTGCCAGACGTACCAATGCCAAGTTTAACAAGATAGTGCTAAAAAGGCTCTTTATGAG

CAAGACCAACAGGCCACCAATGTCTTTGGCAAGAGTACGACTCATGAAAAAACCTGGTCGTGATAGTAAGATGGCTGTAG

TTGTTGGAACTATCACTGAAGATTTAAGGTTATTGGAAGTTCCTAAACTTAAAATTTGTGCCCTGAAAGTCACAGATCGT

GCTCGTGCTCGTATCCTTAAATCTGGTGGCGAAATTATTACATTTGATCAGTTGGCACTCCGGTCACCCAAAGGACAAAA

CACAGTACTGATGCAAGGAGCCCGTAAAGCCAGGGATGCTTACAGACACTTTGGCCGTGCTCCTGGAGTACCACACAGCC

ACACTGCACCCTAT---ACCGAATACAAAGTCATTGGGCGCATGTTGCCCACTGACAAGAACAGAACCCCATCTCTGTAC

CAGATGAGGATCTTCGCCCCAGACAAATGCACTGCCAAGTCAAGGTTCTGGTACTTTGTCACCCAACTCAGGAAGATGAA

AAGGACCCAGGGTGAAATTCTTTGCTGTCAGAAGATCTATGAGAAGAGACCAGAAAAGATCAAGAACATTGGTATCTGGC

TGCGTTACGTGTCACGAAGTGGCAACCACAACATGTACAGGGAGTACAGAGACTTGACCGCTGCTGGTGCCGTCACACAG

TGCCGTGACATGGGAGCCAGACACAGGGCAAGATCTTCTACTATTCAGATCATCAAAGTTCAGGAAGTGGAAGCCAGTAA

GACACGCAGGGCCAATGTCAAACAGTTCCATAACTCCAAGATCCGATTCCCATTGACACACAGGATCCAAAAGCTTCACG

AGCCCAGGTTCACAACCACTAGGCCACACACT---CTCAGGTTGCAGAAGAGGCTGGCCGCAGCCGTGCTTAAATGCGGC

AAAAACAAAGTTTGGCTTGATCCCAATGAAACAAATGAGATAGCCAATGCAAATTCACGTCAAAACATCAGGAAGCTGAT

CAAGGACGGTCTCATTATCCGTAAACCTGTGGCAGTTCACTCTCGTGCTCGTGTGAGGAAGAATGCCATTGCTCGCAGGA

AGGGTCGTCACATGGGACACGGAAAGAGGAAGGGTACACTGAATGCCCGTATGCCAACCAAAATCCTTTGGATCAGGAGA

ATGAGGGTCCTCAGGAGACTACTCAAACGCTACAGAGAAGCCAAGAAAATCGACAAGCATCTGTACCACGAGCTGTACAT

GAAATGCAAAGGTAACGGCTTCAAGAACAAGCGTGTGTTGATGGAGCATATTCACAAGAGGAAGGCCGAGAAGGCTAGAA

CGAAATTACTC---GACAGATATGACAAAGTCTGGAGGAAACCCAAGGGTATTGACAACAGGGTGAGGAGACGCTTCAAG

GGACAGTACAAGATGCCCAACATTGGTTATGGAACAGCCAAGAAGACAAGGCACTTAATGCCCGATGGCTTCAGGAAGTT

CCGCATTCACAATGTTAAGGAACTCGAAGTACTCCTTATGCAGAACAGATCCATTGCTGCCGAGATAGCACACAACGTTT

CCAGTAGGAAGAGGAAAGAAATCGTTGAGCGTGCCCAGCAATTGTCCATCAAAGTGACCAATCCTAATGCTAGACTG---

ACCCTGAGGGTTGCCAAGGTGACTGGTGGAGCTGCATCAAAGCTCTCAAAGATTGTCCGCAAGTCTATTGCCCGTGTGTT

GACTGTCATTCATCAGACACAGAAAGAAAACCTAAGGAAATACTACAGGACAAAGCGCTACAAGCCCAAGGATCTTCGTC

AAAAGAAAACCCGTGCTATTCGCCGCAAGCTCACCCCATCAGAACTTGCTCTGAAGACGAGAAAGGAGCGTAGGAAGAAC

AGTCTCTTCCCCATGAGAAAATACGCTGTAAAA---TCGAAGCAGATGCAGGACGTGCGTGGTGCGCTTCGCGGCGCAGA

GATCCTCATGGGCAAGAACACGATGATCCGCAAGTGTCTTCGTGAACTCGCGGCGGAGAAGCCGGAATTGAGCGAGCTTT

TGCCCAGCATTGTTGGCAACATTGGCTTCTGCTTCACTGACATGGACCTTCTTGAGCTGCGTGACATCCTGGACTCGTAC

AAGGTGCAATCGTCGGCCAAGGCTGGTGTCCTTGCTCCAGTTGATGTTGTCATTCCGGCTGGTGGTACGGGGATGGGACC

AGAGAAGACCTCGTTCTTCCAGGCCCTCAACCTGCCGACCAAGATCACCAAGGGTACCATCGAGATCCTCAGCAATGTGA

CTGTCGTCTCGGCGGGTGCCAAGGTCGGTCTCTCTGAAGCCAAGTTGCTCAACATGTTGAACATTTCGCCCTTCTCGTAT

GGTCTCGTCATCGAAGTATACGATTCTGGTACCCTGTTCAGCCCCAGCATCCTCGACATCACCGATGACGATCTCCGCAG

CAAGTTCATGATTGGAGTGAAGAACATCGCCGCCCTGTCCCTG---ACAAGAGCCGGTCAGAGAACCAGGTTCAAGGCAT

TTGTTGCCATTGGTGACCATAATGGTCATGTTGGTCTGGGAGTCAAGTGCTCAAAGGAAGTGGCAACTGCTATCCGAGGG

GCTATAGTCTTAGCCAAGCTGTCCGTTATCCCTGTACGCAGAGGCTATTGGGGTAACAAGATCGGAAAACCACATACAGT

ACCGTGCAAGGTGACTGGTAAGTGTGGCAGTGTCACAGTGAGATTGATCCCAGCACCCCGTGGAACAGGTATTGTCAGTG

CTCCCGTCCCCAAGAAGCTGTTGAACATGGCTGGTATTGAGGATTGTTACACCAGTGCTAGAGGCTGCACAGCAACACTG

---CTTGCTGAGGATGGTTACAGCGGGGTAGAAGTGCGCGTCACACCGACACGTACAGAAATTATCATTCTGGCCACAAG

GACCCAGAATGTCCTCGGAGAGAAGGGACGTAGGATCAGGGAACTCACATCTGTCGTACAGAAACGATTCAGCTTCCCCG

AGGGAACAGTTGAATTGTACGCAGAGAAGGTTGCCCAGAGAGGTCTGTGTGCCATTGCCCAGTGCGAGTCCCTTAGATAC

AAACTCATTGGCGGTCTTGCTGTCAGGAGGGCATGCTACGGTGTGTTGAGATTCATCATGGAAAGTGGCGCCAAGGGATG

TGAAGTTGTTGTGTCTGGTAAGCTGAGAGGTCAGCGAGCCAAGTCCATGAAGTTTGTTGACGGACTGATGATCCACAGTG

GAGAGCCTATCAACGACTATGTAGATACAGCTGTCAGACACGTACTCCTCCGACAGGGTGTACTGGGAATCAAAGTAAAG

ATTATGTTACCATGGGACCCCAGCGGTAAGATCGGCCCCAAGAAACCTCTGCCAGATCATGTAAACATTGTAGAGCCAAA

GGATGAA---GGCACGAGGAGGAAGATGAAGCGCCTTACGGCGCCAAAGTCATGGATGCTTGACAAATTAAGTGGTGTCT

TTGCTCCTCGACCCAGCACTGGCCCACACAAGCTGAGGGAATGTCTTCCCATGATCGTATTCCTGCGTAACAGGCTGAAG

TATGCCCTGACATACGACGAAGTTAAAAAGATTGTAAACCAGAGGCTGATTAAGGTCGATGGAAAAGTAAGAACAGCCAA

GGACTACCCAGCTGGATTTATGGATGTCATCACCATTGAAAAGACCAATGAAAACTTTAGACTCTTGTATGATGTAAAAG

GTCGATTTACAGTTCATCGCATCAAACCAGAAGAAGCCAAGTACAAACTGTGTATGATCACTGGTGGCCATAACTTGGGA

CGAGTTGGTGTCATCCAGCACAGAGAAAGACATCCAGGCTCCTTCGACATAGTACACATCAAGGACGCTTTAGGACACAC

ATTTGCTACCAGATTGAGCTATGTATTTGTGATTGGCAAAGGAAACAAACCCTGGATCTCCCTGCCCCGCGGTAAGGGTG

TGAAGCTGTCCATCGCTGAGGAGAGAGACAGGAGAATGGCAGCC---CTGAATATATCATATCCGGCCACTGGCTGTCAA

AAACTGATTGAAGTTGATGATGAGAGAAAGTTGCGTCCATTTTACGACAGACGTATTGCAACTGAAGTCTCTGCTGAACA

TCTTGGAGATGAATGGAAGGGTTACATTTTGAGAATTACAGGAGGTAACGACAAGCAGGGATTCCCCATGAAACAGGGAA

TTTTGACAAATGGCAGAGTTCGTCTTCTTTTGAGCAAGGGTCACTCTTGCTTCAGACCCAGGAGAAGCGGTGAGAGACGC

AGGAAGTCCGTCAGGGGCTGCATCGTAGACAGCAATCTCAGCGTTCTTTCTATGGCCATTGTCAAAAAAGGTGAGCAGGA

TATCCCGGGCTTGACCGACACCAATGTTCCCAGAAGGCTTGGACCTAAAAGGGCAAGCAAGATCAGAAAGCTCTTCAACT

TAACAAAGGAGGATGATGTTCGCCAGTATGTCGTCAAAAAACCAAAAACAAAGGCCCCCAAGATCCAGCGACTTGTGACC

CCAGTTGTTCTC---ATCTCCAGAGACAAATGGCACAAGAGGAGGAAGACTGGAGGCCGTATGGCCCAGCTTCACAAGAA

GAGGAAGTTTGAGTTGGGTCGCCCTGCCTCAAACACCAAGCTTACTGTGAGAACCATGGGTGGAAACAAGAAGTTCCGTG

CTCTACGGTTAGACAAAGGAAACTTCTCATCTGAGGCTATCACCAGACAGACACGTATCATCGATGTCGTCTACAACGCC

AGTAACAACGAGCTGGTGAGAACCAAGACTCTTGTCAAAAGCTGTATTGTCCAAATTGATGCTACCCCGTTCAGACAGTG

GTACGAGGCTCATTATGCCCTTCCCCTGGGCAGGAAGAAGGTTGCCAAAGCTAAGATTGCCTCAAGGCCAGGTCAGTGTG

GTCGCTGTGACGGATACATCCTTGAGGGTAAGGAGCTGGAATTCTACCTGAGGAAGCTCAAGACCAAGAAA---AAGACC

TACACCACTCCCCGACGTCCCTTTGAGAAGGAACGATTGGACCAGGAGTTGAAACTTATCGTGGAATATGGTCTGAGAAA

CAAACGTGAAGTATGGCGTGTGAAGTACACCTTGACCAAAATCAGGAAAGCTGCCCGAGAGTTGCTTACCCTGGATGAGA

AGGAACCAAAACGACTTTTTGAAGGTAATGCCCTGTTGAGAAGACTGGTTCGTATCGGTGTGTTGGATGAAACCAAGATG

AAGCTTGATTACGTGCTTGGTCTCCGCCAGGAAGATTTCTTGGAGCGCCGTCTTCAGACTCAAGTGTTCAAATTGGGATT

GGCCAAGAGCATCCATCATGCCCGTGTGTTGATCAGACAGAGACACATCAGAGTAAGGAAACAGGTGGTAAACATCCCAT

CCTACATGGTCCGTCTGGACTCTCAGAAACACATCGACTTCTCCCTGAGATCACCATACGGTGGTGGC---TTCCAGAAG

CAGAACAATGTCTTCTTAAACAAGAAGCGATATGTCAAGTCTATTGGTCTAGGATTCAAAACACCCCGAGATGGTACATA

CATTGACAAAAAGTGTCCCTTCACTGGCAACGTGAGCATCCGAGGACGAATCCTCACCGGGGTTGTCATGAAGATGAAGA

TGCAGAGGACCATTGTTATCCGTAGGGATTACCTCCACTACGTCAAGAAGTACAACAGATTCGAGAAGCGCCACAAGAAC

ATGTCTGTCCATATCAGCCCATGTTTTAGGGATGTAATGCCTGGAGATGTGGTGACCGTTGGAGAGTGCAGACCCCTCAG

CAAGACCGTG---GGTATTTCCCAGTCAGCTCTTCCCTACAGAAGATCAGTCCCAACATGGCTGAAGCTGACATCGGATG

ATGTGCAGGAACAAATATTTAAACTGGCAAAGAAGGGACTCACCCCATCCCAGATTGGTGTCATCCTTAGGGATTCCCAT

GGAGTAGCACAGGTGCGCTTTGTAACTGGGAACAAGATTCTGCGTATCCTTAAGGCCAAAGGTTTGGCTGCAGATATCCC

AGAGGATCTCTACCATCTTATCAAAAAGGCTGTCAACATCCGCAAACACATGGAACGTAACAGGAAGGACAGGGACGCCA

AATTCCGTCTTATCCTTGTGGAAAGCAGAATCCACCGTCTGGCCAGATACTACAAGAGGAAGAGGGTTCTAGCACCAAAC

TGGAAA---ATGGTGCGGATGAACGTTTTGGCAGATGCCTTGCGGACAATTGCTGCCGCTGAAAGACGCAACAAAAGACA

GGTGCTGATCAGGCCCTGCTCTAAGGTCATCATCAAGTTTTTAACTGTGATGATGAAACATGGCTATATTGGAGAATTTG

AAATAGTTGATGACCACAGAAATGGAAAAATCGTTGTAAACCTTACAGGAAGGTTAAACAAGTGCGGTGTGATCAGTCCC

AGGTTTGATGTAGCCCTACGTGACATGGAAAAATGGACCACAAATCTTTTACCTTCCCGACAGTTTGGATACATTGTATT

AACAACATCTGGTGGAATCATGGACCATGAAGAAGCCAGAAGGAAGCACTTGGGAGGAAAGGTGTTGGGATTCTTCTTC-

--GAGGCCCCAATCCACAGGATCAGAATTACATTGACCAGCAGGAATGTCCAGAGCTTGGAAAAAGTTTGTGCTGACCTT

ATTAAGGGAGCCAAGGACAAACAGCTTAAGGTCAAGGGGCCGGTCCGTATGCCCACCAAGGTGTTAAGGATCACCACCAG

GAAAACACCTTGTGGTGAGGGTTCCAAAACCTGGGATCGCTTCCAAATGCGTATCCACAAACGCCTGATTGACCTTCACA

GTCCATCTGAGATCGTCAAGCAGATTACCTCCATCAGTATTGAGCCAGGTGTCGAGGTTGAAGTAACTATCGCTGAC---

ACCATCCGTACAAGGAAGTTCATGTCTAATCGTCTGCTGTCCCGGAAGCAGATGATTGTTGACGTCTTGCATCCGGGAAA

AGCCACAGTTGCCAAGACTGATATCAGAGAAAGACTGGCAAAGATGTACAAAACAACCCCAGACACAATCTTCTGCTTTG

GATTCCGCACCAAGTTTGGCGGTGGCAAGACGTCCGGATTTGCTCTCGTTTATGACAGTTTAGACCAAGCCAAGAAGTTT

GAACCCAAATACAGACTGGTTAGG---GGAAAACCAAGGGGTTTGAGGACAGCTCGTAAACTTAAGAATCACCGTCGTGA

TCAGAAGTGGCACGATAATGATTACAAAAAGGCCCATTTGCCCTCAAGATGGAAGCCCTTCCAGGGTTCATCTCACGCTA

AAGGAATTGTCCTCGAAAAAGTTGGTGTAGAAGCCAAACAGCCCAACTCTGCTATCAGGAAGTGTGTCCGTGTCCAACTC

ATCAAGAACGGAAAGAAGATTACTGCTTTTGTTCCCAACGATGGCTGTCTCAACTATGTTGAGGAAAACGACGAAGTATT

GGTCGCTGGATTTGGACGTAAAGGTCACGCTGTAGGAGATATTCCCGGTGTACGTTTCAAGATTGTCAAGGTCGCCAACG

TTTCACTGCTTGCTCTATTCAAGGGAAAGAAGGAAAGGCCAAGGTCAATG---AGAGAAGCAAGGAAACACAACAACTTC

TATGTACCCGACGAACCCAAACTTGCATTTGTTATGAGAATCAGAGGTATCAACGGTGTCCATCCAAGACCCAGGAAGGT

TATGCAGCTGTTCCGTCTGAGACAGATCAACAACGGTGTATTCATTCGTCTGAACAAGGCCACTATCAACATGCTCAGAA

TCGCCGAGCCTTTCATTACATGGGGATACCCCAACCTGAAGAGTGTGAAGGAGCTGGTTTACAAACGTGGTTACGCCAAG

GTTGATAGAGTACCACTCACAGACAACATCATTGAGGAAAAGTTAATCATCTGTATGGAGGACTTGATCCACGAGATCTA

CACAGTCGGACCCAACTTCAAGCCCGCCACCAACTTCCTGTGGCACTTCAAGATGAACACGCCCAACGGTGGCTGGAAGA

GGAAGTACAACCACTACAATGACGGTGGTGACTTCGGCTGTCGT---AAGACCAAAAAGAGAAAGTTCACTGAATCCATT

GAATTGCAGATTGGTTTGAAGAATTATGATCCACAGAAGGACAAGCGTTTCTCTGGAACCATTCAGTTACGTCATATACC

AAAACCTAAGATGAAGGTTTGCATTCTTGGTGATCAGATTCATTGTGATCAAGCCAAGGCCAATGAAATGCCTTGCATGG

ATGCTGATATGCTAAAAAAATTAAACAAGGACAAGAAGCTTGTTAAGAAATTAGCCAAGAGATACGATGCTTTCCTGGCC

TCTGAGACTCTGATTAAACAGATTCCCAGATTATTGGGACCAGGTTTAAACAAGGCTGGTAAATTCCCTACCCCAATCTC

CCATAATCAATCCCTTGTCAGCAAGGTACAACAAGTAAAGGGAACCATCAAATTTCAAATGAAAAAGGTCTTGTGTTTGT

CTGTCTGTATTGGACACATCAACATGAGCCAAGAAGAAGTTTACACCAATGTCAATTTGGCCATTAACTTTTTGGTTTCC

CTTTTGAAGAAGAATTGGCAGAACGTGAGAACTCTACACATCAAAAGTTCCATGGGACCTGTCCAGAGAATTTAT---GG

GCATTTCCACAAGGATTGGCAGCGTATGGTGAGGACATGGTTCAACCAGCCCATGAGAAAGAAAAGGAGGCACGAGACTC

GTGTCAAGAAAGCTCACAGAATCGCACCTCGTCCAGTTGCAGGACTAAGACCCATTGTCCGATGCCCAACATTCAAATAC

AATACAAAACTTAGAGTTGGAAAGGGATTCACACTTGATGAGCTGAAGGCAGGCATCAATAAGAGAATGGCACTTACCAT

TGGTATTGCTGTGGACTACAGGAGGAGGAATAAGTCTGTTGAGTCACTCCAGCAGAATGTTCAGAGACTGAAGGAATACA

GGAGCAAACTCATCCTCTTCAAGAAGCAGAGCAAACCC---ATGACACGATATTCGTCAGAGCCTGAGAACCCCACAAAG

TCTGCCAAGGCTAGGGGTTCATACCTCAGAGTTCACTTCAAGAACACCAGGGAAACGGCTCAGGCAATCAAGCGTATGCA

CATCTCGAGAGCCACCAAGTACCTTAAAAATGTGATCGACAAGAAGGAATGTATTCCCTTCCGCAGATTCAACGGTGGTG

TTGGTAGGAATGCACAGACCAAGCCATTCAAAGCATCCCAGGGTAGATGGCCCAAGAAAAGTGCAGAGTTTTTGCTTCAG

CTTTTGAAGAATGCTGAGAGCAATGCTGAGTACAAGGGTCTTGACACCGACCATTTAGTCATTGAACACATCATGGTCAA

TGAGGCCCCAAAGATGAGACGACGAACCTACAGGGCTCACGGACGTATCAATTACATGAGCAGTCCTTGTCACATTGAAG

TGATTCTA---ATGAAGTTTAACAAGATGGTCACCTCCTCCAGGAGGAAGAACAGGAAGCGCCATTTCAATGCTCCCTCT

CATGTCAGGAGGAAGATCATGAGCACTCCACTATCTAAGGAACTTCGACAAAAGTACAATGTCAGAAGTATGCCTATCAG

AAAGGATGATGAAGTACAGGTTGTCCGTGGTAATCATAAAAGCCAGCAGGGCAAAATTGTCCAGGTGTACAGGAAGAAGT

TTGTTGTACACATTGAAAGAATCCAGCGTGAGAAGGCCAACGGTGCCTCTGTCCATGTCGGAATCCACCCTTCAAAGATC

CTTATTGTAAAGCTGAAAATTGACAAAGATAGAAAGAGAATCCTTGAGCGCAAGGCCCGATCCAAGGGCAAA---CAGGG

GAAGGTGGTGCTTATCTTGAACGGGCGATTTGCTGGCCGTAAAGCGGTCATCGTCAAGAACTACGATGATGGCACACAAG

AGAAGGGATATGGTCATGCTTTAGTAGCTGGCATTGACCGATACCCTCGCAAGGTCACCCGTGATATGGGCAAGAAGAAG

ACCAAGGACAGGTCCAAGATGAAGACTTTTATGAGAGTATACAACTACAACCATCTGATGCCCACAAGATACTCTGTTGA

TGTGAGGTACAAGACAGGAAAGAACAGATGGTTTTTCCAGAAGCTGCGGTTC---CTGAGAGGTCATGTCAGTCATGGAC

ATGGTCGTATCGGCAAACACAGGAAGCATCCTGGTGGTCGTGGTAACGCTGGTGGTCAACATCACCACAGGATCAACTTT

GACAAATACCATCCAGGTTACTTCGGAAAAGTTGGTATGAGATACTACCACAAGACCCAGAATAAGTTTTTCTGCCCCAC

AGTAAATTTGGACAAACTGTGGTCCCTCGTGTCCGACCAGACCAGGGAAAAGGCTCCTGTTATCGATGTTGTGCGTGCAG

GATACTACAAGGTCTTAGGAAAGGGACATTTGCCCAAGCAGCCTGTGATTGTGAAGGCCCGCTTCTTCAGCAGATCAGCA

GAACTTAAAATCAAGGCAGCTGGTGGCGCCTGTGTTCTAGTGGCA---ATGGGCGCCCACAAATCTTTCAGAATCAAGAT

GCGTCTGGCAAAAAAACAGAAGCAGAACAGGCCAGTGCCACAGTGGGTCAGAATGAAGACTGGCAACACAATCAGGTACA

ATGCTAAGAGGCGTCACTGGAGGAGGACAAAGCTGAAGATG---ATGGCACGTACAAAGCAGACCGCCCGTAAATCCACT

GGAGGCAAGGCCCCACGTAAACAGCTGGCCACCAAGGCCGCTCGTAAGAGTGCCCCAGCTACCGGAGGTGTTAAGAAACC

TCACAGGTACAGGCCAGGAACAGTAGCTCTCCGAGAAATCCGTAGGTACCAGAAGAGCACTGAACTCCTCATCAGGAAAC

TCCCCTTCCAGCGTCTCGTCCGTGAAATCGCACAAGATTTCAAGACCGACCTGAGGTTCCAGAGCTCAGCTGTTATGGCT

CTGCAGGAGGCTAGCGAAGCTTACCTGGTTGGACTTTTCGAGGACACCAACCTGTGCGCCATCCACGCCAAGCGTGTCAC

CATCATGCCCAAGGATATCCAGCTGGCTCGCCGTATCCGCGGAGAGCGTGCT---CTCGCATGCATATATGCCGCACTCA

TCCTCGCTGACGACAATGTAACCATTACTGATGACAAACTTGCAACCATTCTCAAGGCTGCCAAGGTGAACGTGGAACCA

TATTGGCCA---ATGCGTTACGTAGCAGCCTATTTGCTTGCAAGTCTTGGAGGAAATGCAAACCCCTCCGCAGCAGACCT

TGAGAAGATCATTGGGTCTGTTGGCATTGAGGCTGAGGCCGACAAGATCAAAAAAATTATATCTGAGCTGAAGGGAAAGA

ACATTGAAGAGCTCATCACTGCAGGA---GAAAAGGAATGGATGCCTGTCACTAAACTAGGACGTTTGGTGAAGGACCTT

AAGATAAAGTCTCTAGAGGAGATTTATCTGTTTTCATTGCCTATCAAGGAGTTTGAAATCATTGACTTTCTTTCTGCCCT

GAAGGATGAAGTACTGAAGATCATGCCTGTTCAGAAACAG---AGGTACCTGCCCCACTCTGGAGGCCGCTATGCCGCCA

AGCGTTTCCGTAAGGCCCAGTGCCCAATCGTGGAGCGTCTGACCAACTCCATGATGATGCACGGCCGCAACAACGGCAAG

AAGCTGATGACTGTGCGCATCGTGAAGCATGCCTTCGAGATCATCCACCTGCTGACCGGAGAGAATCCCCTTCAGGTCTT

GGTGAACGCCATCATCAACAGTGGACCCCGTGAGGACTCCACCCGTATCGGTCGTGCTGGTACAGGTGCCCGAGAGGCCT

CCTTCAGAAACATTAAAACCATCGCTGAATGTCTGGCAGATGAGCTCATCAACGCAGCCAAGGGATCTTCTAACTCCCAT

GCTATCAAGAAGAAGGACGAG---AGCGCTAAAATAATTAAGCCTCAAGGCGAGAAGCCAGATGACTTCGAGCAGTCTGT

CTCACAGGCCCTGCTTGAGCTGGAGATGAACAGTGACCTGAAGGCCCAACTTCGTGAACTGTACATTTCAGGCGCTAAGG

AAATTGATGTTGCTGGAAAGAAAGCTATCATCATCTTTGTGCCAGTTCCCCAGCTAAGACAATACCAGAAAATCCAGACC

CGTTTGGTCAGGGAGCTGGAAAAGAAGTTCAGCGGAAAGCATGTTGTATTTATTGCACAGAGGAGAATTCTTCCCAAACC

AACAAGAAAAACCAGGAAGAACAAGCAGAAGCGTCCCAGATCCCGTACTCTCACCGCAGTACATGACTGCATCTTGGACG

ATCTGACCTTCCCTGCAGAAATCGTTGGCAAGAGAATCCGCATCAAGTTGGATGGTTCCAGGCTGATCAAAGTCCACCTC

GATAAAACTCAGCAGACAAACATTGAGCACAAGATGGATACATTCACAGCTGTGTACAAGAAACTGACTGGGAAGGACGT

TACATTTGAGTTCCCAGAATATGTGCTT---ATGCCCAAGAAGAACCGTGTGCAGATCTACGAGTACTTGTTCAAGGAGG

GTGTCCTTGTTGCAAAGAAGGATTTTGGAGCACCCAAACATCCCGAGATTGATGTCCCCAATCTGCATGTCATCAAGGCA

TTGACATCTCTGAAGTCCCAGGGCTATGTCAGGGAACAGTTTGCGTGGCGTCACTTTTACTGGTACCTTACCAATGAGGG

TATCCAGTATCTGAGAGACTTCCTCCATCTCCCCGCAGAGATTGTCCCTGCCACACTCAAG---CATGTCACAGATTTGT

CAGGAAAAGAAACCATTGCCCGTGTGACTGGAGGTATGAAGGTCAAAGCCGACAGAGATGAGGCTTCCCCATACGCTGCT

ATGTTGGCTGCTCAGGATGTTGCCGAGAGGTGCAAGACTCTTGGAATCAATGCCCTGCACATTAAACTGAGGGCCACTGG

TGGTAACAGAACTAAAACTCCAGGACCTGGTGCCCAGTCAGCTCTGAGAGCTCTGGCTCGTTCAGGAATGAAGATCGGCA

GAATCGAGGATGTC---AAGAAGAAGAAGCGTACTTTTAGGAAGTACACTTTCAGGGGTGTTGATCTTGACCAACTCTTG

GACATGTCAAGCGAACAGTTGATGGAGTTGCTCTCTGCCAGGGCCCGTCGTAGGTTCTCAAGGGGTCTGAAGAGGAAACC

CATGGCACTCATCAAGCGTCTTAGAAAGGCTAAAAAGGAGGCCAAACCTTTAGAAAAGCCAGAGGTAGTGAAGACACATT

TGAGAAACATGATCATTGTACCAGAAATGATCGGTAGCGTTATTGGAGTATACAATGGCAAGACCTTCAACCAAATCGAA

GTCAAGCCTGAGATGATCGGCCACTACCTTGGAGAGTTCAGCATCACATACAAGCCAGTCAAGCATGGTCGTCCCGGTAT

TGGTGCCACTCACTCCTCACGATTTATTCCTCTCAAA---ATTATTGAGAAATACTACACCAAGTTGACCCTCGACTTCC

ACACTAACAAAAGAGTATGTGAAGAAATCGCTATTTTGCCTAGCAAGAAGCTTAGGAACAAAGTAGCAGGGTTTGTCACC

CATTTGATGAAGCGTATCCAGGCTGGGCCAGTCCGAGGTATCTCCATCAAGCTACAGGAAGAAGAGAGGGAAAGGAGGGA

TAACTACGTCCCAGAGGTTTCTGCGCTTTTGCATGACGTTATTGAAGTTGATCCCGATACAAAGGACATGTTGAAGTCT-

--CCTAGAAAATGTTCTGCCAGTAACAGAATTATTGCAGCTAAAGACCATGCATCCATCCAACTGAACATTGCTGATGTT

GATGAGAGCACTGGACGTATGACTGGTTCAAACACAACCTACGCCATCTGTGGCACAATCAGAAGGATGGGAGAGTCTGA

CGACTCCCTGAACAGGTTGGCCAAAAGGGACAACATTCTCGCTAAG---CACACCAAACATGTCCGCTGTACAAACTGTG

CCCGTTGTGTGCCCAAGGACAAGGCCATCAAGAAATTTGTGATCAGGAACATCGTAGAGGCCGCCGCTGTTCGTGACATC

TCGGATGCCAGTGTATATGACGTGTATGCCCTCCCCAAGCTGTATGCCAAACTACTGTACTGTGTATCCTGTGCTATCCA

CTCCAAGGTCGTGAGGAACAGGTCTCGGGAGGCACGAAAAGACCGTACTCCTCCGCTACGCTTTAGGCCA---ATGATCT

TTGTCAAGACCCTCACGGGCAAAACCATCACCCTTGAGGTTGTGAAGGCCAAGATCCAAGAAGGCATTCCTCCTGATCAG

CAGAGATTGATCTTTGCTGGAAAGCAGCTTGAGGATGGAGACATCCAGAAAGAATCAACCTTGCACTTGGGTGGTGCCAA

GAAAAGGAAGAAGAAGAACTACACCACCCCAAAAAAGAACAAGCACAAGAAAAAGAAGGTCACCCTGGCCGTCCTAAAGT

ACTACAAGGTGGACGAGAACGGAAAGATCACCCGTCTGCGACGTGAGTGTCCCAATGATGACTGTGGTGCTGGTGTATTC

ATGGCTTCCCACTTTGACCGCCAGTACTGTGGAAAATGCTGCCTCACTTTTGTATTTAATAAG---AAGCCATACCCCAA

GTCAAGGTTTTGTCGTGGTGTCCCAGATCCCAAGATCCGTATTTTTGATCTTGGCAAGAAAAAGGCCAGGGTAGACGAAT

TCTCATTATGTGTGCATCTTGTCTCTGACGAGTATGAACAGCTGTCATCTGAGGCATTGGAAGCCAGCCGTATCTGTGCC

AACAAATACCTTGTGAAGAACTGTGGCAAGGACTCCTTCCATTTACGAATGCGAGTGCATCCCTTCCACGTTTGCAGAAT

CAACAAGATGTTGTCGTGTGCTGGAGCTGATAGGCTCCAGACAGGGATGCGTGGTGCCTTCGGCAAACCCCAAGGAACCG

TAGCCCGTGTGCACATTGGTCAGCCCATTATGTCCTGCCGTGCCAGGGAAACCAACCAGGCTGCTGTCATCGAGGCTCTC

AGGAGAGCAAAGTTCAAGTTCCCAGGCAGACAGAAGATCTTTGTCTCCAAGAAATGGGGCTTCACC---AAAAGAACAAA

GAAGGTGGGAATTTGCGGGAAATATGGAACCCGATATGGTGCCTCTCTCCGTAAAACCATCAAAAGAATGGAAGTCTCTC

AGCATTCTAAATACACATGTCAATTCTGTGGAAAGGATGCCATGAAGAGGAAAGCTGTGGGGATCTGGAGCTGTAGTCGA

TGCAGGAAGACCGTGGCCGGAGGTGCATGGGTTTACAGCACAACTGCCGCCGCTACCGTCAGAAGTGCAGTCCGTCGTCT

CCGTGAAATG---CATATCCAGAAAAAAACATGTGCTTCATGTGGATATCCTAGTCCAAAGAAGAGGTCATACAACTGGA

GTGAGAAATCAAAGAGGAGGAGGACAACAGGCACTGGCCGTATGAGGCATCTCAAGGATGTCTTCAGGAGATTCAGGAAC

GGCTTCCGTGAGGGTACAGTC---ACAGGAAGGGCTTCTCTTTATGCCCAAGGAAAGAGAAGATACGACAGAAAACAAAG

TGGATATGGAGGTCAAACGAAGCCTATTTTCCGAAAAAAGGCAAAGACCACAAAGAAAATCACACTGAGAATGGAATGTA

CAACATGCAAGTACAGGAAACAGCTGTCACTAAAAAGATGTAAGCACTTTGAGTTGGGAGGTGACAAGAAGAGGAAG---

CAGACCGCCCATGTGCGACGTGTCCGTGACCTTATCCGTGAAGTGACAGGCTTTGCTCCATATGAGAGGAGAATGCAGGA

GTTGCTGAGGATCTCCAAAGATAAGAGGGCCCTCAAGTTTGCCAAGCGAAGGCTGGGCGCACATGTAAGAGGAAAGAAGA

AGAGGGAAGAAATGCAGACAATCTTACAGAAGATCAGGAAGGCGCAA---AAGAGTATTGAGAACATCAACTCTCGATTG

GCTCTTGTTATGAAGAGTGGGAAGTATGTCCTTGGTTACAAGCAGACACTGAAGACACTCCGACAGGGCAAGGCTAAGCT

GGTCATCATCGCCAACAACACCCCACCCCTCAGGAAGAGCGAGATTGAGTACTACGCTATGTTGGCTAAAACTGGCGTAC

ATCACTACACTGGAAACAACATAGAGTTGGGTACTGCCTGTGGTAAATACTTCAGAGTCTGCACCCTCAGCATCACTGAC

CCAGGT---AGAAAGTTGAGGTCATTCTCCAAGCACAAGCACAGCTTGAGGTCATCAATTACCCCTGGAACTGTTCTTAT

CCTAGTGGCCGGTCGTCACAAGGGAAAGAGAGTTGTCTTCCTGAAACAATTGGATTCTGGATTACTGTTGGTTACAGGTC

CATTCCATCTTAATGGTTGCCCTCTGAGAAGAATCAACCAAATTTACACTATTGCCACTAAAACAAAGCTGGACATCAGT

GGTGTTAAATTGCCCGAACGCCTGAATGACAAATATTTCAACCGTAAACAGACAGAAGGAGAAATCTTTGACACCAAAAA

GGAGTATCAAGTCAGTGATGATCGTGAACAAGATCAGGTGGATGTAGACAAACAGGTCCTC---GTCACTATCACTGGGC

CGAGAGGTACACTCACCAGAAATTTCCGTCATCTGGACATCAGTATGGTCAGAACTGTGTGCAGTCACATCGAAAACATG

ATGAAGGGCGTCACCGGTTACTTGTACAAGATGAGATCTGTATATGCTCATTTCCCGATCAACATTGCTATCCTTGAAGG

CGGACACTCTGTGGAGGTCAGGAATTTCTTGGGTGAGAAATACACCCGTAAAGTCAACATGTTGCCTGGAGTGACCTTCT

CCACCTCTCCTGGTATGAAGGACGAATTCTTCATCCAGGGAAATGACATCGAGCTGGTATCCCAGTCAGCTGCTCTGGTC

CAACAGTCTACTACAGTA---GTGTACGTGAAGGCTGTGGGAGGTGAAGTACCAGCAACATCATCCCTGGCTCCAAAGAT

TGGTCCACTCGGTTTGTCCCCAAAGAAAATTGGTGACGACATCTCCAAAAGCACCCAGGAGTGGAAGGGACTGAAGATTA

CCGTGAAGTTGGTCGTCCAGAACCGTCAGGCCAAGGTAGAAGTTGTGCCAAGCGCATCTTCTCTGGTGATCAAAGCCCTC

AAGGAACCCCCAAGGGACAGGAAGAAGGTGAAACATGTTAAACATAATGGCAACATCAGCATGGACGAAATCATCAGGAT

TGCTCGTATCATGAGGCCCAGGAGTATGGCCAGGAACTTAGCCGGAACCTGTAAGGAAATTCTTGGCACTGCCCAGTCTG

TTGGCTGTTCTGTGGAGAAATGCCCTCCT---GATGCCAGAGGGCATTTATTGGGTCGACTGGCAGCCTTAGTTGCAAAG

ACCATTTTGCAAGGGCAACGAGTCGTGGTTTTGAGATGTGAAGGTATCAACATCTCTGGAAACTTCTACAGGAACAAGCT

GAAGGTATTGAAATACCTGAAGCTGAGGTGTAATGTGAAGCCTACAAGAGGACCTTACCATTTCAGGGCTCCAAGCAAGA

TGTTCTACAAGGCTGTGAAAGGTATGTTGCCTCACAAACTTGCTAGAGGAAAGGAGGCCCTTGCCAGACTAAAGGTGTTT

GAAGGAATTCCAGCTCCTTATGACAAACAGAAGAGGATGGTTGTTCCCTCCGCACTCAAAGTCCTAAGGATCCACCCAGG

CAGAAAGTTCTGTGACCTCAACCGTCTGGCCCATGAAGTTGGATGGAAGTATCAGACTGTGATTTCTACCCTTGAA---A

TGACCAACACAAAGGGATATAGGCGCGGTACGCGCTACATGTTCTCTCGTCAATTCAAGACAAAGGGAGTCATTCCATTG

TCTACGTATATGAAAATATACAGAAGAGGAGACATCGTCGATGTTAAGGGCCACGGAGCTGTACAGAAGGGTATGCCCCA

CAAGGTCTACCACGGAAAGACTGGCCGAGTATTCAATGTCACTCCTCACGCTGTTGGTGTTGTTGTAAACAAGCGTGTCC

GAGTTCTGCCCAAGAGAATCAACTTGAGGATTGAACACGTGAAACACTCAAACTGTCGTCTGGACTTCGTGAAGCGTGTG

AAGGAAAACGCCGAAAAGAAGAAGGAGGCTAAGGAGAAGGGAATCATGCTTAGCTTGAAACGCCCCAACACATCCCACTT

TGTGAGGAAATACAACAAGCCTCAGCTGATTGAACCAATTCCATACGAGTTCATTGCC---ACGATCAACGAGGTCATCA

CAAAAGAATGTACCATCAACGTCCACAAGAGGATCCATGGAATCGGCTTCAAAAAGAGGGCTCCCCGTGCCATCAAAGCT

GTGCGTAAATTCGCAGAGAAGATGATGGGAACTCCCGATGTCAGGATAGACACAAGGCTCAACAAGCATGTGTGGTCTCA

GGGTGTCAGGAATGTTCCATACAGAGTACGTGTGAGGTTAGCTCGTAAGAGGAACGACGACGAAGACTCCACAAACAGAT

TTTACACACTTGTTACATATGTACCTTGTGCATCCTTCAAA---ACCTTGGTCACCAGAACTCAAGGAACCAGGATTGCT

TCTGATGGCCTGAAGGGGCGAGTGTTTGAGGTCTCCCTGGCTGATCTTCAGAATGATGAAGTATCCTTCAGGAAGTTCAA

ACTGATGGCTGAAGAAGTTCAGGGACGCAATGTTCTGACCAACTTCCACGGCATGGATCTGACCCGTGACAAACTCTGCT

CTATGGTCAAGAAATGGCAGACCATGATCCAGGCTCATGTTGATGTCAAGACCACTGATGGTTACCTTCTTAGGATGTTC

TGTATTGGTTTCACCGAGAAGCAGTTTCAAGTGAAGAAAACTTGTTACGCCCAAACCACACAGATCAAAACCATCAGAAG

AAAGATGGTTGATATCATCACTAAGGAAGTGACATCAAATGACATGAAAGAAGTTGTCAACAAACTAATCCCAGACAGCA

TTGGCAAGGATATTGAGAAAGCCTGCCAAGGAATTTATCCCCTTCACGATGTTTTCATCCGTAAGGTCAAAGTGCTGAAA

AAACCTAAGTTCGACACAGACTCCGGCGAAACTGTGGCGCGGCCCGAGGGATACGAACCTCCAGTCCAACAATCTGTA--

-GGACGCCCTCTTGAGAATATTGAACCCCAAATTCTCCGGGCAAAGCTCCAGGAACCTTTATTACTCCTGGGAAAGGAAA

AGTTTGCTGGGGTTGATATCCGAGTCCGTGTCCGAGGAGGTGGTCATGTAGCTCAAATCTATGCGATCCGGCAGTCCATC

TCCAAAGCTCTGGTAGCCTACTATCAAAAAGTTGACGAAGCATCGAAGAAGGAGATTAAAGATATCCTCATAAGCTATGA

CCGTACACTACTGGTCGCCGATCCCAGACGTTGTGAGCCCAAAAAGTTTGGAGGACCTGGTCCACGTGCACGTTACCAGA

AGTCTTACCGT---CGGTATGCCAACGTTGTCTGCAAGAAGGCTGATGTTGACATCACAAAACGTGCTGGAGAGCTGAGC

GAGGATGAGAGGGTGATCACAATCATGTCCAACCCACGACAGTACAAGATTCCTAACTGGTTTCTCAACAGACAAAAAGA

TGTCAAAGACGGTAAATACAGCCAGGTCATGTCCAACGCCCTCGACAACAAGCTCCGTGAGGACCTTGAAAGACTAAAGA

AAATCCGGGCACACAGAGGTCTCCGACACTACTGGGGTTTGAGGGTGCGTGGACAGCACACAAAGACAACTGGCAGGAGA

GGAAGAACTGTTGGTGTGGCCAAGAAG---ATGAAGATTCCAGAGTGGGCCACTATTATCAAGCTGTCAAAGTTTAACGA

GCTGTCCCCATATGATGAGGACTGGTTCTACACACGGGCATCCATCTGCCGCCACTTGTACATCCGTCCAGCTGGTGTTG

GTGCCCTGACAAAAATCTATGGAGACCGTAAGCGTAATGGAACAGTCCCCAGCCATTACTGTAGGTCATCAGGCTCAGTG

GCTCGACGAGTACTCCAGGCTCTCGAAACACAGAAGTTGGTGGAAAAGGACGCCAATGGTGGACGTAAACTGACCAGCCA

AGGACAGAAAGATCTGGACAGAATCGCGGCCCAGGTG

>strong

TCTCACAGAAAATTCTCAGCTCCCAGGCACGGTTCCTTGGGCTTCCTGCCCCGCAAGCGCAGCAGGCGTCACCGAGGCAA

AGCCAAGGCTTTCCCCAAGGATGACAAGAGCAAGCCAGTGCATCTGACCTGCTTCCTTGGATACAAGGCTGGCATGACCC

ATATTGTGAGGGACGTGGACAGACCCGGTTCAAAGGTGAACAAAAAGGAAGTAGTTGAAGCCGTCTCCATCCTTGAAACA

CCTCCCATGATCATTGTCGGTCTTGTGGGTTACATCGAGACCCCATCCGGTCTCCGTGCCCTCAAGACTGTCTGGGCTGA

ACATCTCAGTGACGAGTGCAAGCGCCGATTCTACAAAAACTGGTACCGCTCCAAGAAGAAGGCTTTCACCAAGTCTTCCA

AGAAGTGGGCCGATGAGATGGGCAAGAAGGAGATCGAGAAGGACCTAAACATCATGAAGAAATATTGCACCGTCATCCGC

GTCATTGCTCACACCCAGATGAAACTCATGAACCAGCGCCAGAAGAAGGCTCACGTCATGGAGATCCAGCTCAACGGTGG

CAGCATCGGAGACAAGATCGAGTGGGCCAGGGAGCGCCTGGAGAAGAACATTCCAGTCTCTGAGGTCTTCGGACAGGACG

AGTTGATCGACATCATCGGTGTCACCAAGGGACATGGGTTCAAGGGTGTGACCTCCCGTTGGGGAACCAAGAAGCTGCCC

CGTAAGACCCACAAGGGTCTGCGCAAGGTGGCCTGTATCGGAGCCTGGCATCCAGCCCGTGTGGGCTACGGAGTTGCCCG

TGCC---AGACCACTTGTTACAGTGTACAATGAGAAGAATGAGAATGTGACCCTCCCAGCTGTCTTCAAGGCACCAATCA

GGCCTGACATTGTCAACTATGCCCACACCAACATGAGGAAGAACAGCCGTCAGCCCTACGCTGTCAAGAGGATCGCTGGT

CACCAGACCAGTGCTGAATCTTGGGGTACAGGACGTGCCGTCGCCCGTATCCCCCGTGTCCGGGGTGGTGGTACCCACCG

CTCTGGTCAGGGAGCCTTCGGTAACATGTGCCGTGGCGGACGCATGTTTGCCCCTACCAAGATCTGGCGTAAGTGGCACA

CACGCACCAACATCAGCCAGAAGCGCTTCGCCATGTGCTCCGCTCTCGCTGCCTCGGCAGTCCCAGCACTCGTCATGTCC

AAAGGTCACATGATCATGGGCACACCAGAAGTACCCCTTGTTGTCAGTGACAAAGTCCAAGACTTCAAGAAGACCAAGGA

AGCAGTCTACTTCCTCAAGCGCTTCAAGGCGTGGGAAGACATCAAGAAGGTGTACAAGTCTGATCAAGACAACGGCATCA

CAAGGGCCTTCAGGAACATCCCAGGTGTGACACTTCTCCAGGTCTCAAGACTGAACCTCTTGAGGCTAGCTCCCGGTGGC

CATGTTGGAAGGTTCTGCGTATGGACAGAGTCTGCATTCAACAAGCTTGATGGCCTCTAT---CGCCGTGAAGGCAGGAC

TGACTACCAGGCCCGCCGCTTCTTGGTTGTACAAGACAAGAACAAGTACAACACACCCAAGTACCGCATGATCGTTCGCT

TCACCAACAAGAATATCACATGTCAGGTAGCGTACGCCAAGCTGGAGGGAGATGTCATTGTATGCGCCGCATACTCTCAT

GAGCTCCCCCGCTATGGTGTCAAGGTCGGGCTGACCAATTATGCTGCAGCATACTGCACAGGACTACTCCTGGCTCGCAG

GATTCTGAAGAAGTTCAACTTGGACGGTATCTACGAGGGTCAGACAGAGCCAGATGGCGATGACTACATGGTAGAGGAGG

AGGGTAAGGCAGGAGCCTTCCGTTGCTTCCTGGATGTCGGTCTAGCCAGGACCACCACCGGCGCCAAGGTGTTTGGGGCC

CTC---GTCAACCCCCTTTTTGAGAAGAGGACAAGGAACTTTGGAATTGGCAATGAGGTCCAGCCCAAGAGGGACCTGTC

CCGCTTTGTCCTCTGGCCCAAGTATGTACGCCTCCAGCGCAAGGCTGTCCTTTACCAGCGTCTGAAGGTTCCTCCTTCCA

TCAACCAGTTCAGCCAGGCTCTCGATAGACAAACAGCTACACAGCTGTTTCGTCTGATGCACAAGTACCGCCCCGAGACC

AAGCAGGAGAAGAAGGCCAGGCTCAGGGCCCGTGCCGAGGATCGCGTCAAGGGACGTGAAGAGGTTCCCACTAAGCGTGA

AGCTCGCATCATGTCCGGTGTCAACACTGTCACCCGTTTGATCGAGTCCAAGAAAGCTCAGCTTGTTGCCATTGCTCATG

ATGTCGAACCCATCGAGATCGTGATGTTCCTCCCAGCACTCTGCCGTAAGATGGGTGTTCCCTACTGCATCGTGAAGGGA

AAGTCTCGGCTCGGTCAGGTGGTCCATCGCCGTAACGCAGCTTGCCTTGCGTTCACCCAGGTCAACGAGGACAAGAGCGC

CCTCTCCAAGCTGGTAGACACAGTCAGGACCAACTACAATGACAGATTTGAAGAGATCCGTCGTCACTGGGGTGGTGGAC

AGGTGGGTAGCAAGAGCCAGGCCCGT---ATGGGTCGAGTGGTCTTCAGATCGCACAACAAGCACAGGAAGGGAGCCCCC

CTCCGATCCATCGACTACGCAGAGCGTCATGGATACCTCAAGGGCATCGTCAAGGAAATCATCCACGACCCCGGACGTGG

TGCTCCCCTTGCCAGGATCCAGTTCCGTGACCCCTACAGGTACAAGAAGCGTACTGAACTCATGTACTGCGGCAAACGAG

CCACCCTGAACATTGGCAACGTGCTTCCCCTCGGCTCCATGCCCGAAGGAACCGTCATCTGCTCTGTGGAGGAGAAGGCA

GGAGACAGGGGCAAGCTGGCTCGCTGCTCTGGCAACTACGCCACTGTGGTCTCCCACAACCCTGATACCAAGAAGTCCCG

TATCAAGCTTCCTTCCGGCTCCAAGAAGGTCGTGCCCTCAGCCAACAGGGCCATGGTCGGAGTTGTTGCTGGTGGCGGAC

GTATCGACAAGCCCCTCCTGAAGGCTGGACGTGCCTACTTCAAGTACAAGGTCAAACGCAACTGCTGGCCCCGAGTGCGT

GGTGTTGCTATGAACCCCGTTGACCATCCCCATGGTGGTGGTAACCATCAACATATTGGTACCCCCTCTACAGTCAGGAG

G---AGTGGTGACAGACTCACCCGTGCTGCCAAGGTTCTGGAAGCTCTGACTGGACAATCTCCAGTCTTCTCCAAGGCCC

GCTACACCGTGCGATCTTTTGGCATCCGTAGGAACGAGAAGATTGCCGTCCATTGCACCGTTCGTGGTCCTAAGGCCGAG

GAGATCCTTGAAAAGGGATTGAAGGTTCGTGAGTACGAGTTGCGCAAGGGCAACTTCTCCTGC---GTGTTCAGGAGGTT

CGTCGAGGTCGGCCGTGTGGCCTACATCGCCAGCGGACAAAACAAGGGCAAGCTATGTGTCATCGTTGATGTCATTGACC

AGAGGAGGGCCCTTATTGATGGACCACTCTCCGGCGTGAAGCGCCAGGGAATGCGCTTCAAGCAGCTTCACTTGACAGAC

TTTGTTATCCGCATTCCTCATTCTGCGCGTAACAGCACCGTAAGGAAGGCGTGGGAAAAGGACGAGATCACCAGCAAGTG

GGACGCCACCATCTGGGCCAAGAAGCTCGACCGCTACAAGCTCATG---TACAAGTACATGTCAGAAGTATGGCGTAAGA

AGCAGAGCGATGTGATGAGGTTCCTCGTCCGAGTCAGGACCTGGCAGTTCCGTCAGCTGTCAGCCATCCACCGAGCCACC

AGGCCCATGCGTCCAGACAAAGCAAGGAGCATGGGATACAGGGCCAAGCAGGGATACGTTATCTACCGTATCCGTCTTCG

CCGTGGAGGTCGCAAGAGGCCCGTGCCCAAGGGAGCCACCTATGGCAAGCCCACCAACCAGGGAGTCAACCAGCTCAAGT

ACCAGAAGTCTCTCCAGTCCACCGCTGAGGAGCGTGTCGGACGCAGGTGCGGTGCCCTCCGTGTCCTGAACAGCTACTGG

ATCTGTGAGGACTCAACCTACAAATACTTTGAGATCATCCTTATTGATCCAATGCACAAAGCCATCAGACGAAACCCAGA

CACTCAATGGATCACAAAGTCAGTCCACAAACATCGCGAATGCCGTGGACTCACCTCAGCCAGCAGGAAGAGTCGTGGTC

TTGGCAAGGGACATCTTTACAAGCAGACTTCAGGTGGTTCCAGGAAGGCATCCTGGAAACGCAGGAACCTTCTCCGTCTC

CAAAGATATCGC---AGGTTTCTGGCCAGGCGTACAAATGCCAAGTTCAACAACATCGTCCTCAAGAGGCTGTTTATGAG

CCGCAGCAACAAGGCTGCCATGTCTCTCGCACGTGTGCGCTTCATGAACAAAGATGGCCGTGAGGGCAAGATTGCCGTCG

TAGTCGGGTGCATCACCGATGACGTGCGTATCCACAAGCTGCCCAAGCTGAAGATCTGTGCCCTGAGGGTGACTGCCCGT

GCTAGGGCTAGGATCATCAAGGCTGGAGGAGAGATCATCACCTTCGATCAGCTCGCCCTCCAATCCCCCAAGGGACAGAA

CACAGTGCTCATGCAAGGATGCCGAACCCATCGCAAGGTGTACAGACATTTCGGAAGGGCCCCAGGAACCCCAAGTGCAC

ACACTGCACCATAC---ACAGAGTTCCTGGTGGTGGGACGGGAGATGCCTACAGAGAGGCAGTCTCTCCCACCCCTCTTC

AAGATGCGGATCTTTGCACCAGATGAAGTCACTGCCAAGTCTCGCTTCTGGTACTTTGGTAACAGACTACGAAAGATGAA

GAAGACTCGTGGAGAGATCTTGGGCTGCAGGAAGATCAACGAGAAGAAGCCCCAGAAGATCAAGAACTTTGGTATCTGGC

TGCGCTACGACTCCAGGAGTGGAACTCACAACATGTACAGGGAGTACCGTGACCTGACTACTGCTGCAGCAGTCACCCAG

TGCCGTGACATGGGAGCAAGGCATCGTGCCCGTGCCCATTCCATACAGATCATCCGAGTGGAAGAAATCCCAGCTTCCAA

ATGCAGGAGACCTCACATCAAGCAGTTCCTGACCTCAAAGATCAGCTTCCCCCTCCCACATCGCACACAGAGGCAAAACA

AGCCCATGTTCGTCACCCGCAGACCCAGCACC---CTACGGTTGCAGAAGCGCTTAGCCGCAAGCGTGATGAGCTGCGGC

AAGAACAAGGTCTGGCTGGATCCCAACGAAATCAACGAGATCGCCAACGCCAACTCAAGGCAGAACATCCGCAAGCTCAT

CAAGGATGGTCTGATTATCCGCAAGCCAGTTGCTGTACATTCCAGGGCCAGGGTTCGCAAGAATGCCGAGGCTCGTAGGA

AAGGTCGACACAGTGGACGTGGTAAGCGCAAGGGTACAGCCAATGCTCGTATGCCAACGAAAGTGATCTGGATCAGGCGG

ATGCGAGTCCTTAGGAGGCTACTCAAGAAGTACAGGGAGAACAAGAAGATCGACAGGCATCTGTACCACGAGCTGTACAT

GAAGGTCAAGGGTAATGTCTTCAAGAACAAGCGTGTTCTCATGGAGTTCATCCACAAGAAGAAGGCAGAGAACCAACGTG

GCAAGATGCTT---GATCGCTACCTGAGAGTCTGGCGTCGTCCTAAGGGTATTGACAATCGTGTGAGGAGGCGATTCAAG

GGTCAGCTTCGTATGCCAAACATCGGTTATGGAAGCGCCAAGAGCACCCGCCACATGCTACCCACTGGCTTCCGCAAGTT

CCTTGTACACAACGTTAAGGAGCTCGAAGTTCTTATGATGAGCAACAGGAAGTTCGCCGCTGAGATTGCCCATGGAGTCT

CCTCTCGCAAGCGCAAGTCCATCGTCGAGCGCGCCCAGCAGCTGGCCATCAAGGTCACCAATGGCAACGCCCGTCTC---

CAGCTTCGAGTCGCCAAGGTGACCGGTGGCCAGGCATCCAAGCTTGCCAAGATCATCCGCAAGAGTATTGCCCGTGTGAG

GACTGTCATGCACCAGACCCAGAAGGTTGAGCTGCGCAAGTACTACCGCAACCGCAAGTTCAAGCCCCTGGACCTCCGCC

CCAAGAAGACACGAGCCATCCGCCGTGAGCTCAGCCGATCAGAGCTGAACCGCCGTACCAGCAAACAGATCCGCAAGGCC

AGCATGTACCCCATGCGCAAGTACGCCGTCAGG---TCCAAGCAGATGCAGGCGATCCGTTCCTCGCTTCGAGGAGGTGT

CGTCCTCATGGGCAAGAACACCATGATCCGCAAGGCCATCAGAGGTCACATGGAGAATGATCCTGGTCTGGAAAAGCTCC

TTCCCCACATGAAAGGCAATGTTGGATTTGTATTCACCAAGGGAGAATTGCTGGAGGTCCGAGAAAAGATCCTGGCTAAC

AAGGTCGCTGCCCCAGCCAAGGCTGGTGCCCTCGCACCAATTGATGTGTTCATTGACAGCGTCAACACCGGCTTGGGACC

CGAGAAGACCTCTTTCTTCCAGGCTCTTTCCATCGCCACCAAGATCGCCAGGGGAACCATTGAAATCTTGAACAAAGTAC

ATCTGATCAAGCTGGGCGAGAAGGTAGGAGCTTCTGAGGCCACTCTGCTTCAGATGTTGAAGATCTACCCCTTCTCGTAT

GGTCTCCAGATCCGCGTGTACAAGGCAGGCTCTGTGTTCCATCCCACCATCTTGGACATCACCGATGAGGACATTCCCGT

TCAGTTCATCCAGGGTGTGGCCAACGTGGCTGCCCTCTGTCTG---ACCCGTGCTGGACAGCGCACCAGGTTCAAGGCTT

TTGTAGCTATTGGTGACCACAATGGCCACATCGGTCTTGGAGTGAAGTGCTCCAAGGAAGTAGCGACAGCCATCCGTGGA

GCCATTATCCTGGCCAAGCTTTCCATCATCCCAGTCAGGCGAGGCTATTGGGGTAACAAGATCGGCAAGCCTCACACAGT

ACCCTGCAAGGTTACAGGAAAGTGCGGCAGTGTATTGGTGCGTTTGATCCCTGCACCCCGTGGTACTGGTATTGTCAGTG

CCCCAGTACCCAAGAAGCTGCTTCATATGGCTGGTATCGAGGATTGCTACACCAGCGCCTGCGGACAGACCGCTACCCTC

---CTGGCCGAGGATGGGTACAGCGGTGTGGAGGTCCGTGTGACCCCTACCAGGACCGAGATCATCATCCTGGCCACTCG

CACCCAGAGTGTCCTTGGGGAGAAGGGCAGGCGCATCAGGGAGCTCACCGCTGTTGTGCAGAAGAGGTTCAACTTCGCAG

AGAATACCGTTGAGCTGTATGCTGAGAAGGTTGCCACCCGTGGTCTCTGTGCCATCGCCCAGTGCGAGTCCCTTCGCTAC

AAGCTGATTGGAGGTCTTGCCGTCAGGAGGGCCTGCTACGGAGTCTTGCGCTTCATCATGGAGAGTGGCGCCAAGGGCTG

CGAGGTGGTGGTCTCTGGCAAGCTGCGTGGACAGAGAGCCAAGTCCATGAAGTTTGTGGACGGTCTCATGATCCACAGTG

GTGCTCCAGTCAAGGACTACGTCGATGTGGCCATCCGTCACGTCATGTTGAGGCAGGGTGTGCTTGGGATCAAGGTGAAG

ATCATGCGTCCCTTCGATGTGACCGGTAAGACTGGACCCAAGAAACCACTCCCTGATGTCATCAGCATCTCAGAACCCAA

GGAGGAG---GGGCCAAAGAAGCATCTGAAGCGCTTAAATGCTCCCAAGCATTGGATGCTTGCGAAGCTCACCGGAAATT

TTGCTCCTCGTGCATCTACCGGACCCCACAAGCTAAGAGAATGCTTACCTCTCATCATCTTCCTCCGCAACCGTCTCAAG

TACGCCCTGACCTACGTCGAGACCAAGAAGATCATGATGCAGCGTCTGATCAAGGTTGACGGCAAGGTGCGCACTGATAT

CACCTACCCGGCTGGATTCATGGATGTGATCTCCATCAACAAGACTGGAGAGAACTTCCGTCTCATCTACAACGTCAAGG

GACGCTTTGTTGTCCACAGGATCGGCAGCGAAGAAGCCAAGGTAAGATTGTGCATGATCACCGGAGGTCGTAACTTGGGA

CGTGTAGGTACCATCACCCACAGGGAGAAGCACCCTGGATCTTTCGAGATCGTCCACATCAAGGATGCCTCAGATCACAC

CTATGCCACCCGTCTGGGCAACGTGTTTGTCATCGGCAAGGCCAACAAGGCCTATGTCTCCCTCCCCAAGGGCAAGGGAA

TCCGTCTCACCATCGCAGAGGAGCGCGAGAAGAGGATCGCCCAG---CTGAATATAAGTTTTCCCGCTGTGGGCACACAG

AAGCTCATCGAGGTTGATGATGAGCACAAGCTGAGGGCTTTCTATGACAAGCGTATGGCTCATGAGATGACTGCCGAGTC

CCTCGGAGATGAGTGGAAGGGCTACGTTGTGCGCATCAGTGGAGGAAACGACAAGCAAGGTTTCCCCATGAAGCAAGGTA

TCCTGACCAATGGACGTGTACGTCTTCTCCTCAGCAAGGGCCACTCCTGCTATCGCCCAAGGAGAAGGGGAGAGCGCAAA

CGCAAGTCTGTCCGAGGCTGTATTGTCGATGCTAATCTCTCAGTTCTCAACCTGGTCATTGTCAAGAAGGGTGAAGGTGA

GATCCCAGGCCTGACCGACAAGTCCATCCCACGTAGGTTGGGACCCAAGCGTGGTGGCAAGATCCGCAAGCTCTTCAACC

TTACCAAGGAGGATGATGTCAGGCAGTATGTTGTCAAGCAGACCAAGTACAAGGCACCCAAGATCCAGCGTCTGATCACT

CCTCTGCGTCTT---ATCTCACGTGATAGGTGGCACAAAAGACGAAAGACGGGAGGAAGATGCCCTCCCATTCGCATGAA

GCGAAAGTTTGAGCTGGGTCGCCCACCTGCACTCACCAAGCTTCTTGTTCGATGCATGGGAGGAAACATCAAACGCCGTG

CTCTGCGTCTAGACAATGGAAACTTCTCATCAGAACACACTACCCGCAAGACCCGTATCATCGATGTTGTGTACAATGCC

AGCAACAACGAGTTGGTGCGTACCAAGACTCTGGTGAAGAATGCCATCGTTCAGATCGACTCCACACCATTCCGTCAGTG

GTATGAGGCTCACTATGCCCTGCCTCTTGCCAGGAAGAAGGGAGCTAAGGCATGCATTGCTTCAAGGCCTGGCCAATGTG

GACGTGCTGATGGCTACATCCTGGAAGGAAAGGAGCTTGACTTCTACATGAGGAAGATGCGTGCCAAGAAG---AAAACC

TTTGTGACGCCCCGTCGTCCATTCGAGAAGGAACGCCTTGACCAGGAGTTGAAGCTCATCGGCGAGTATGGACTCCGTAA

CAAGCGTGAGGTGTGGCGTGTCAAGTACACCCTAGCCAAGATCAGGAAGGCTGCCAGAGATCTGCTCACCAGGGAGGAGA

AAGACCCCAAGAGGTTGTTCGAAGGTAACTCCTTGTTGCGTCGTCTCGTGCGTATCGGTGTGCTGGATGAGAGCAAGATG

AAGCTGGATTACGTGCTGGGTCTGCGAATTGAAGATTTCTTGGAGCGCCGCCTCCAGACCCAAGTCTTCAAGCTTGGACT

TGCCAAGAGCATCCATCATGCTCGTGTGCTCATCCGTCAGCGTCACATCCGTGTCCGCAAGCAGGTTGTGAACATCCCCT

CGTACATCGTTCGTCTGGACTCCCAGAAGCACATTGACTTCTCCCTTCGCTCGCCATACGGTGGTGGA---TTTCAGAAG

CAACCAACTGTGTTCCAGAACAACAAGAGGCTCGTCCGCAATGTTGGTCTTGGATTCAAGACTCCTCGCGATGGCCACTA

CATCGACAAGAAGTGCCCTTTCACTGGCAACGTTCACATCCGCGGGCGAATCCTGACCGGTGTCATCACCAAGATGAAGA

TGCAGAGGACAATTGTCATCAGGCGTGACTACCTCCACTACATCAAGAAGTACAACCGATTTGAGAAGAGGCACAAGACT

ATGTCTGTCCATCTCTCACCCTGCTTCAGGGACACCAAGACCGGAGACATCGTGACCGTAGGTGAATGTCGCCCTCTCAG

CAAGACCGTC---GGTATCTCTGCGTCAGCACTTCCATACAGGAGAAGTGTGCCAACATGGCTGAAGCTGTCCTCAGATG

ATGTGAAGGAACACATTCAAAAGATGGCTAAGAAGGGTCTCACTCCATCACAGATTGGTGTGATGCTTCGTGATTCCTAC

GGAGTCGCCCAGGTGCGCTTCGTCACCGGCAACAAGATCCTAAGGATCCTGAAGGCCAAGGGTCTGGCCCCATCTCTCCC

GGAGGACCTCTACTCTCTCATCAAGAAGGCCGTCGCCGTACGCAAGCATCTTGAGAGGAACCGCAAGGACAGAGACTCCA

AGTTCCGTCTGATTCTGATCGAGAGCAGGATCCACAGACTGGCCCGATACTACAAGACCAAGCGTATCCTTCCACCCAAC

TGGAAA---ATGGTGCGTATGAATGTATTGGCAGACGCACTGAGGTCGATCTGCAATGCTGAGAAACGATGCAAGCGTCA

AGTGCTGATCAGACCATGCTCCAAGGTTACTGTCAAGTTCCTTATGGTGATGATGAAGCATGGATACATTGGGGAATTTG

AGATCGTAGACGACCACAGGGGAGGCAAGATCATTGTCAACCTCAACGGCAGACTCAACAAGTGTGGTGTGATCAGTCCT

CGCTTTGATGTGCCCATCAACGAAATGGAGAAATGGACCAGCAATCTCCTTCCATCTAGACAGTTTGGGTATGTAGTCCT

GACCACATCGGGAGGTATCATGGATCACGAGGAGGCGAGGAGAAAACACGTCGGTGGCAAGATCCTGGGCTTCTTCTTC-

--GAAGTCCCAGCCCATCGCATCCGCATCACCCTCACCAGCCGCAATGTCAAGAGCCTGGAGAAGGTGTGCGCAGATCTC

AAGCGCGGAGCCCAGGAGAAGAACCTGAAGGTGAAGGGACCGGTCCGCATGCCGACCAAGACCCTGCGTATCACCACCAG

GAAGACACCCTGTGGTGAAGGGTCCAAGACCTGGGATCGCTTCCAGATGCGCATCCACAAACGTCTCATCGACCTGGAGA

GCCCCTCTGAGATCGTCAAGCAGATCACCTCTATCAGCATCGAGCCTGGTGTCGAGGTTGAGGTCACCATCGCCGAC---

ACGATCAGAACAAGGAAGTTCATGACCAACCGTCTTCTGAACAGAAGGCAAATGGTTGTGGATGTGATCCATCCTGGCCA

AGCCACCGTCAAGAAGACGGAAATCCGTGAGCGCCTTGCACAGGCCTACAAGACCACCCCTGATGTCATCTTCGCCTTTG

GCTTCAAGACCCAGTTTGGCGGTGGACGTACCTCGGGCTTTGCCCTCATCTACGACACTCTGGACTCTGCCAAGAAATAC

GAACCCAAATACAGATTAGCAAGA---GGAAAGCCAAGAGGTCTTAGGACAGCTCGCAAGCTGCGCACCCATCGCCGAGA

TCAGCGATGGCATGACAAAGATTACAAGAAGGCTCACTCCAGTACTGCTCTAAACCCGTTCGGAGGTGCCTCTCACGCCA

AGGGCATTGTTCTGGAGAAGGTTGGAGTGGAGGCTAAGCAGCCCAACTCTGCTATCCGCAAGTGTGTCAGGGTGCAGCTC

ATCAAGAACGGCAAGAAGATCACAGCTTTCGTTCCCAATGACGGTTGCCTCAACTTCATCGAGGAGAATGACGAGGTGTT

GGTTGCTGGATTCGGTCGTAAGGGACATGCCGTCGGTGATATCCCCGGAGTTCGATTCAAGGTTGTGAAGGTTGCCAATG

TCTCGCTCTGGGCTCTCTTCAAGGGAAAGAAGGAACGTCCCAGGTCAATG---CGCCAGGCACGCAAGCATGGCAACTTC

TACGTCGAAGATGAACCTAGACTAGCGTTTGTCATCAGGATCAGGGGTATCAACGGCGTGAGCCCCCGTGTTCGTAAGGT

CCTTCAGCTGCTTCGTCTGCGACAGATCCACAATGGAGTCTTTGTCCGTCTCAACAAGGCTACCCTGCAGATGCTGAGGT

TGGTCGAGCCCTACATTGCCTGGGGGTACCCCAATCTGAAGTCAGTCCGTGAGCTGATCTACAAGCGAGGATTCGCCAAG

ATTAAGCGCATCCCTATGACAGACAACATCATTGAGGGTGCTCTTATCATCTGTGTGGAGGATCTGATCCATGAGATCTT

CACCACTGGCGAAAACTTCAAGCAGGCCTCCAACTTCCTCTGGCCCTTCAAACTCAGCTCACCCCGCAACGGCTACCGCA

AGAAGGGCAACCACTTCGTCGAGGGTGGTGACTACGGCAACCGC---AAGGAGAAGAAGAGGAAGTTTCTGGAGACGATC

GAGCTTCAGGTCAACCTCAAGAACTACGACCCCCAGAAGGATAAGCGTTTCTCCGGAACCGTCAAATTGAGGCACATCCC

TCGCCCCAAGTTCTCCATGTGCATCCTTGGAGATCAGCAGCATCTTGATGAGGCCAAGGCCAATGGCATCCCCTGCATGG

ACATGGAGGCCCTCAAGAAGCTGAACAAGAACAAGAAGCTTGTCAAGAAGCTAGCTAAGAGGTATGACGCCTTCCTTGCC

TCCGACTCGCTCATCAAGCAGATCCCACGTATCCTGGGACCCGGTCTTAACAAGGCAGGCAAGTTCCCCACCCTCCTGAC

CCATACCGACTCCATGGTTTCAAAGGTTGAAGAGGTCAAGGCTACCATCAAGTTCCAGATGAAGAAGGTTCTGTGCTTGG

CCGTTGCCGTCGGACACGTGGAAATGGACGAGGAGGATCTGGTGTCCAACATCAACCTGGCCATCAACTTCCTGGTGTCC

CTGCTCAAGAAGAACTGGCAGAACGTGCGTTCCCTGTACATCAAGAGCACCATGGGAAAACCACAGCGCCTCTAC---GT

CCATTTCCGCAAGGAATGGCAAAACTATGTCAGGACGTGGTTTGACCAACCAGCCCGCAAGAAGAGACGACACAACAACC

GTGTCCAGAAGGCCCGCAAGATCGCCCCTAGACCCATCGCAGGACTTCGACCCCAGGTTACTTGTCAGACATTCAAGTAC

CACACCAAGCTGAGGGAGGGACGTGGCTTCACGCTTGAGGAGCTCAAGGCTGGTATCCACAAAAAGTATGCCCCAACCAT

CGGCATCTCTGTTGACCACAGGAGGAAGAACAGGTCAGTCGAAGGGCTCCAGGCCAACGTCCAGCGCCTCAAGGAGTACA

GATCCAAACTCATCCTCTTCAAGAAGCTGAGCAAACCC---ATGACGCGCTACTCCCAAGAACCGGAGAATTCTGCAAAG

TCCTGCAAGGCAAGGGGCTCCTACCTTCGTGTTCACTTCAAGAACACCCGCGAGACCGCTCAGGCCATCAAGCACATGCA

TGTCCGCAAGGCCATCCGCTTCCTGAAGGATGTAACCAACAAGAAGCAGTGCGTTCCCTTCCGCAGGTTCAATGCCTGCA

TTGGACGCAAGGCCCAGGCTAAGGCATGGAACCACACCCAAGGTCGCTGGCCCAAGAAGAGTGCCGAGTTCCTTCTTCAG

CTCCTGAAGAATGCTGAGAGCAACGCAGAGTACAAGGGTCTCGATGTCGACTCCCTTGTCATCGATCACATCCAAGTCAA

CGCTGCCCCCAAGATGAGGAGGAGGACATACCGTGCCCATGGTCGTATCAATTACATGAGTTCTCCATGCCACATTGAGC

TGATCCTT---ATGAAGCGCAATCCTTTAGTCTCATCCTCGAGGAGGAAGAACCGAAAGCGCCACTTCAATGCCCCTTCC

CACGTGCGCAGAAAGCTGATGAGCTCACCCCTCTCCAAGGATCTGAGGCAGAAGTACAATGTCCGCAGCATCCCCATCCG

CAAAGATGATGAAGTTCAGGTAACTCGTGGACACTACAAGGGCCAGCAGGGAAAGGTTGTGCAGGTTTACAGGAAGAAGT

TTGTTATCTACATTGAGAGGATCCAGAGAGAGAAGGCCAACGGTGCCCAGGTCTACGTTGGAATCCACCCCAGCAAGGTT

GTGATCACCAAGCTGAAGATGGACAAGGACCGTAAGCTCATCCTGGACCGCAAGGCCAAGTCCAAGGGCAAG---TCGGG

CCGTGTGGTCCTTGTCCTTTCAGGACGATACGCTGGACGCAAGGCAGTAATTGTCAAGAATCACGATGATGGGAGCTCTG

ACCGTCCATACAGCCATGCCCTTGTGGCCGGCATTGACCGTTACCCACGCAAGGTGACCAAGGACATGAGCAAGAAGAAG

ATCAAGAGGCGATCCAGGATCAAGACCTTTGTCAAGCTCTTCAACTACAACCATCTCATGCCAACCAGGTACTCCGTTGA

TGTGAGGTACAAGACTGGCAAGAACAAGTGGTTCTTCCAGAAGCTGCGATTC---CTGCGTGGTCACGTGAGCCACGGCC

ATGGTCGTATCGGCAAGCACCGCAAGCATCCCGGAGGAAGGGGACGTGCTGGTGGTCAGCATCATCACAGAATCAACTAT

GATAAATACCATCCTGGATACTTCGGTAAAGTTGGTATGAGGCATTTCCATCTGACCAAACAGAAGTACTACAAGCCTGC

CATCAACTGTGACAAGCTCTGGAGTCTGGTCAGTGAGCAGACAAGGCTGAAGGCCCCCGTCATCGATGCCGTTCGAGCGG

GCTACTACAAGATCCTAGGAAAGGGAGTCCTCCCTAAGCAGCCGGTCATCGTCAAGGCCAAGTTCTTCTCCCGACTCGCT

GAGGAAAAGATCAAGAAGGCCGGCGGATGCTGCGTGCTCGTCGCC---ATGCCTTCCCACAAGACATTCAAAATCAAGCA

GAAGCTTGCTAAGAAGCAAAAGCAGAATCGCCCCATCCCACAGTGGGTGCGTCTCAGGACAGGCAACAGGATTCGCTACA

ACGCCAAGAGACGTCACTGGAAGCGAACGAAGCTGAACTTC---ATGGCTCGTACCAAGCAGACGGCTCGAAAGTCTACT

GGAGGAAAAGCCCCTCGTAAGCAGCTTGCTACCAAGGCAGCCCGTAAGAGTGCCCCCTCTACAGGTGGAGTCAAGAAACC

TCATCGTTACAGGCCAGGTACTGTTGCCCTTCGAGAGATCCGTCGCTACCAGAAGAGTACCGAGCTGCTCATCCGCAAGC

TGCCATTCCAGCGTCTGGTCCGTGAGATTGCTCAGGATTTCAAGACTGAGTTGAGGTTTCAGAGTGCTGCTATTGGTGCT

CTGCAGGAGGCCAGCGAAGCTTACCTAGTTGGTCTCTTTGAGGACACCAACCTGTGCGCCATCCATGCCAAGCGTGTCAC

CATCATGCCCAAGGACATCCAGCTGGCTCGTCGCATCCGTGGAGAACGTGCT---CTTGCCTGCGTATACAGCGCACTTA

TCCTTCAAGATGACGACGTCCCCATCACGGCTGACAAGCTGCAGACCCTGATCAAGGCAGCCGGTGTGTCAGTTGAGCCC

TACTGGCCC---ATGCGTTACGTGGCCGCATACCTCCTACTCGCCCTTGGCGGCAAACCCAATGCTTCAGCTGCTGATGT

GAAGAAAGTTCTCCAGAGTGTCGGAGTCGAGATCGACGATGAGAAGCTCGGTATCGTCATCAAGGAGCTGGAAGGCAAGA

AAGTGAAGGATCTGATCGAAGAGGGC---GACAAGGAATGGCAGCCAGTAACCAAGCTCGGCAGGTTGGTCAAAGACCTG

AAGATCAAGGAACTGGAGGAGATTTACCTGTTCTCTCTCCCCATCAAGGAATTTGAGATCATTGACTTCCTTCCCGCCCT

CAAGGACGAGGTGCTCAAGATCATGCCCGTCCAGAAGCAG---AAGTACCTCCCCCACAGCTCTGGCCGCTACCAGGTCA

AGAGGTTCCGCAAGGCCCAGTGCCCCATCGTGGAGCGCATCACCAACTCCATGATGATGCACGGCAGGAACAACGGCAAG

AAGCTGCTCACCATGAGGATCATCAAGCACGCCTTTGAGATCATCCACCTGCTCACCGGAGAGAACCCTCTACAGGTTCT

TGTGAACGCCATCATCAACAGTGGACCTCGTGAGGACTCTACCCGTATCGGTAGGGCTGGTACTGGAGCTCGTGAGGCTG

CATTCAGGAACATCAAGACCATCGCTGAGTGCTTGGCTGATGAACTCATCAATGCCGCCAAGGGATCCTCCAACTCGTAC

GCAATCAAGAAGAAGGACGAG---GGTGCGAAGGTGATCAAGCCTCAGGGCGAAAGTGCAGACCCATTTGAACTCACAGT

TTCACAGGCTTTGATTGAGCTCGAGTCCAACTCAGAACTCAAAGGACAGTTGAGGGAGCTCTTCATCACAGGAGCAAAGG

AGATTGATGTTGGTAACAGGAAGGCTGTCATCGTCATGGTTCCCGTACCTCAACTCAAAGCTTTCCAGAAGATCCAGGTC

AGGCTAGTCCGAGAGCTTGAGAAGAAGTTCAGCGGCAAGCATGTGCTCTTCATCGCTCAGAGGAGAATCTTGCCCAAGCC

AACAAGGAAGTCTAACAAGAGCAAGCAGAAGCGCCCTCGCAGCCGAACCTTGACCATGGTCCACGACAACATCCTGGAGG

ACCTCGTCTACCCATCAGAGATCGTGGGAAAGCGTACTCACATCAAGCTGGACGGCAGCAGGAACATCAAGGTCCATCTC

GACGTTGGACAGAAGACACAGATTGAGCACAAGACGGAGACCTACGGTGCCGTATACAAGAAACTGACCGGAAAGGACGT

CACCTTTGAGTTCCCAGAATTCACAGTT---ATGCCAAAGGTGAACCGTGTTGCCATCTATGAGAACCTCTTCAAGGAGG

GCGTGATTGTGGCTAAGAAAGATTTCTACAGCCCCAAGCATCCTGAACTGGAGGTACCCAACCTTCATGTCATCAAGGCT

TGTCAGTCTCTGAAGTCCCGTGGCTACGTCAAGGAGCAGTTCGCCTGGCGTCACTACTACTGGTACCTGACCAACGAGGG

TATCCAGTACCTCAGGGACTACCTTCATCTCCCACCAGAAATCGTTCCTGCCACCCTCAAG---CATGTGACTGATCTGT

CTGGCAGGGAAACCATCGCCAGGGTGACCGGTGGCATGAAGGTGAAGGCTGACCGTGACGAGGCCTCCCCGTACGCTGCC

ATGTTGGCTGCCCAGGACGTTGCCATCAAGTGCAAGGATCTCGGCATCACTGCTCTTCACATCAAGCTCAGGGCCACCGG

AGGAAACAAGACCAAGACCCCTGGACCTGGTGCTCAGTCTGCCCTACGTGCCCTGGCTCGCTCTGGCATGAAGATTGGAC

GTATTGAGGATGTC---GTAAAGAAGAAGCGTACCTTCAGGAAGTACACCTTCCGAGGTGTTGACCTTGATCAACTCTTG

GACATGTCCAATGAGGTCCTGAGCGAGCTGCTCCATAGCCGTGCCAGGAGACGATTCAAGCGTGGACTCAAGCGCAAGCC

CCTGGCCCTCCTCAAGAAGCTCCGCAAGGCCAAGAAGGAGTGCCCAGCCCTGGAGAAGCCCGAGGTTGTCAAGACCCATT

TGAGGAATGTCATCATCGTCCCCGAGATGGTTGGATGCATGGTCGGTGTCTACAACGGCAAGGTCTTCAACCAAGTGGAG

ATCAAGCCTGATATGATTGGTCACTACCTGGGAGAGTTCAGCATCACCTACAAGCCTGTGAAGCACGGTAGGCCCGGTAT

CGGAGCCACCCACTCCTCCAGATTCATCCCACTCAAG---ATCATTGAGAAGTACTACACCCGCCTTACACTTGATTTCC

ACACAAACAAGAGAGTATGCGAGGAGATTGCCATCATCCCCAGTAAACGTCTGCGAAACCAGATTGCTGGATTCGTGACG

CATCTTATGAAGCGTATCCAGAAAGGTGCTGTCCGTGGTATCTCCATCAAGCTTCAGGAAGAGGAGCGTGAGAGGAGGGA

TAACTACGTTCCTGAGGTCTCCGCCCTGGAACAGGACATCATCGAGGTCGACCCTGACACCAAGGACATGCTCAAACTC-

--CCACGAAAGTGTTCTGAGAGCAACAGGGTTATTGGTGCTAAGGATCATGCATCAATCCAGATCAATGTAGGAGAGGTT

GACCCTGTGACTGGCATCTACACACGTAGCTTCAAGACCTATGCCATCTGTGGCCCCATCCGTGGAATGGGCGAGTCGGA

CGACAGCATCAACAGGTTGGCCAAGAGGGACGGATACATTGCAAGC---CATGTGAGGCCCGTCCGCTGCACCAACTGTG

CCCGCTGCGTGCCTAAGGACAAGGCTATCAAGAAGTTTGTTATCAGGAACATTGTTGAGGCTGCAGCCATCCGTGACATC

CAGGATGCCAGCGTCTACGAAGTGTATGCTCTTCCTAAACTCTACGCCAAGCTCCACTACTGTGTGAGCTGCGCCATCCA

TTCCAAGGTCGTGCGTAACCGTTCCAGGGAAGCTCGTCGTGACAGGACCCCACCCCCAAGATTCCGCCCA---ATGATTT

TCGTGAAAACACTGACGGGGAAAACCATCACCCTCGAGGTGGTCAAGGCTAAAATTCAAGAAGGTATCCCTCCCGATCAG

CAGCGTTTGATCTTCGCCGGCAAGCAGCTGGAAGATGGCGACATCCAGAAGGAGTCGACCCTCCATCTGGGTGGCGCCAA

GAAGCGCAAGAAGAAGAACTACACCACCCCAAAGAAGACCAAGCATAAGAAGAAGAAGGTCAAGCTCGCTGTCCTCAAAT

TCTACAAGGTCGACGAGAACGGCAAGATCACCCGTCTGCGTCGTGAGTGCCCCAGCGAGGAGTGCGGTGCCGGTGTCTTC

ATGGCTTCCCACTTTGACCGCCAGTACTGCGGAAAGTGCTGCCTGACCTACGTCTACAACAAG---AAGCCCTATCCCAA

ATCCCGGTTCTGCCGTGGTGTCCCAGACCCCAAGATCCGTATCTTTGACTTGGGTCGCAAGAGGGCTCTGGTTGACGAGT

TCCCTATTTGCATCCATCTTGTGTCTGATGAATGGGAGCAGCTGTCGTCCGAAGCCTTGGAGGCCGGGCGCATCTGTGCC

AACAAGTACATGGTGAAGAACTGCGGTAAGGACGCCTTCCACATCAGGATCAGGCTCCATCCATTCCACGTCATCCGCAT

CAACAAAATGTTGTCGTGCGCTGGAGCTGATAGGCTTCAGACTGGAATGCGTGGAGCTTTTGGCAAGCCCCAGGGTACCG

TAGCCCGTGTGAAGATCGGTCAGACCATCATGTCTGTCAGGACCAAGGAGGGTAACAAGGCTGCTGCACATGAAGCTCTC

AGGAGGGCAAAGTTCAAGTTCCCTGGACGTCAGAAGATCTTTGACTCCAACAAGTGGGGCTTCACC---AAACGTACAAG

GAAAGTAGGAATCACTGGAAAGTATGGAACCCGTTATGGTGCTTCCCTCCGTAAGATGGTGAAGAAGATTGAAATCTCCC

AGCACGGCAAATGCACCTGTGTTTTCTGTGGAAAGGATTCCATGAAGCGTAAGGTCGTTGGCATCTGGCACTGCAAGATC

TGCCGCAAGACTGTGGCTGGAGGTGCCTGGGTATACAGCACCACAGCTGCTGCCACCGTCAGGTCCGTAGTACGTCGTCT

CCGTGAAATG---CACATCCAGAAGTTAACATGCTCGAGCTGCGGATACCCAGCCAAGACCATCAGGAAATATAACTGGA

GTGCAAAGGCAAAGCGCCGAAAGACAACTGGCACTGGCCGCATCAAGCATCTTAGGGCAGTCAACAGGAAATTCAGTAAT

GGATTCCGTGAAGGCACTAAG---GCTGGTAAAGCTTCACTCTACACTCAGGGTAAGAGGCGTTACGACAGGAAACAGAT

GGGATATGGAGGACAGACCAAACCTATCTTCCGAAAGAAGGCCAAGACCACCAAGAAGATTGTACTGAGGATGGAATGCA

CAGACTGTAAATTCCGCAAGCAGCTTCCCATCAAGAGGTGTAAGCACTTCGAGCTGGGAGGAGACAAGAAGAGGAAG---

ATTAACAAGCATGCTAAGTTCGTGCGTGACCTGATTCGTGAGGTGACTGGACTTGCTCCATACGAGAAGAGGTGCATGGA

GTTCCTCAGGGTTGGCAAGGACAAGAAGGCTCTCAAGTTCTGCAAGAGGAGGTTGGGAACTCTTGGCCGTGGCCGCAGAA

AGCGTGAGGAGATGAACGCCATCTTAGCCGCCCAGAGGAAGGCCGCC---AAGCAGATGGAAAGCATCAACACTCGCCTT

CAACTGGTGATGAAAAGTGGCAAGTACAATCTTGGCTACAAGCAGACATTGAAGGCTCTCCGCAATGGCAAGGCAAAGCT

GGTCATTCTTGCTAACAACACACCCCCACTTAGGAAAAGTGAAGTAGAATACTACGCTATGTTGGCCAAGACTGGTGTCC

ATCACTACAGCGGTAACAACATTGAACTCGGGACAGCCTGCGGAAAGTACTTCAGGGTGTGCACACTCTGCATCACAGAC

CCTGGT---CGTAAGTTGAACAACTTCAGCATGCACAAGTCCAAGCTGAGGCCATCTATCACCCCCGGCACTGTCCTCAT

CCTCCTGGCTGGACGCTACAAGGGAAAGAGGGTTGTGTTCGTCAAGCAACTCGATTCCGGTCTCCTCCTTGTCACAGGTC

CCTTTGACCTGAACGGTGTACCTCTGCGCAGGATCAACCAGACTTACGTCATCGCTACCCAGACCAAGCTGGACATCAGC

TCTGTCAAGGTCCCTGAGAGGTTGAACGACCTCTACTTCAAGCGCATGAAGCAAGAGGGAGAGATGTTCGAATCTAAGAA

GGAATATACCGTCTCAGAAGAACGCAAGGAGGACCAGAAGTCTGTCGACGGCCAGCTCCTG---GTGACGGTCAAGGGAC

CGCGTGGGACTCTTGTGCGGACATTCAAGCATCTCGAGATCCGTTGTGTCCGAACTCTCTGCTCTCACATCGAGAACATG

ATCAAGGGAGTCACTGGTTACCGCTACAAGATGAGGTCTGTGTACGCTCACTTCCCCATCAACGTCAACATCAAGAATGA

GGGAACCCAGGTAGAGATCAGGAATTTCTTGGGAGAGAAGTTCATCAGGAAGGTGGATATGAAGGAAGGAGTCACCTGTA

CCACGTCCACCAAGCAGAAGGATGAGATCATCCTTGATGGCAACGATGTCGAGCTCGTCTCACAATGTGCTGCTCTCATC

CAGCAATCCACCACCGTA---GTCTGCCTGAGGGCTGTAGGAGGTGAGGTCGGAGCAACCTCAACCCTCGCTCCCAAGAT

CGGTCCCCTTGGTCTGTCACCTAAGAAGGTAGGAGATGACATCGCCAAGGCCACCCAGGAATGGAAGGGGCTGAAGATCA

CCGTCATGTTGACCATTCAGAACCGTCAGGCCAAGGTCAGCGTGGTGCCCAGCGCATCATCTCTCATCATCCGTGCCCTC

AAGGAGCCCCCTCGTGACAGGAAGAAGGTCAAGAACATCTCTCACAGCGGGAACATCGGCCTGGACGTCATCATTGAGAT

CGCCAAGACGATGCGTGAGCGTTCCATGGCCCGTGAGCTGAAGGGAACGGTCAAGGAGGTCCTGGGAACCGCCCAGTCTG

TCGGATGCACCGTGGACGGGGTCAAACCC---GATGGGAGAGGTCACTTACTTGGACGCCTGGCGTCCATAGTGGCCAAA

AACCTCCTCCAAGGTCAGAAGGTTGTCGTGGTACGATGTGAGCTCATCAACATCTCAGGCAGCTTCTACAGGAACAAGCT

GAAGTACATGCAGTTCATGCGCAAGCGCACAAACACCAAGCCCTCCCGTGGGCCCTACCATCTCCGCAGTCCTAGCCGTA

TGTTCTGGAGAGTCATCAGGGGAATGTTGCCACACAAGAGGACACGTGGCAAGGATGCTCTTGAGAGGCTCAAGGTCTTT

GAGGGCTGCCCTGCCCCCTATGACAGGAAGAAGAGGTTCGTTGTGCCCTCTGCACTGAGAGTGATGCGCCTCAAGCCCAA

CAGGAAGTTCTGCGTGCTCGGTCGTCTGGCTCATGAGGTCGGCTGGAAGTACAAGAACATCATCGAGGCTCTCGAG---A

TGACAAACACCACAGGTTACCGCCGCGGTACGCGGTACATGTTCTCTAGGGCCTTCAAGACCAATGGAGTAGAAAAGCTG

TCAACCTTCCTGAGGGTGTACAAGCGTGGCGACTATGTCGACATCAAGGGTTGTGGAGCCTTCCAGAAGGGTATGCCGTA

CAAGGTATACCATGGACGTACTGGTCGTGTCTTCAACGTCACCCCCCATGCCGTTGGTGTGGTTGTGAACAAGCAAGTCC

GCGTCCTGCCCAAGCGTATCAATGTTCGCATTGAGCACATCAAGGCATCCAAGAGTCGAGAGGGATTCTTGGAGAGGAGG

AGAACCAACGACATCCTCAAGAAGCAAGCCAAGGAGAAGGGTGAATTCTACCAGCTCAAGAGACCAAGGAAGGCTCACTT

TGTTAGTAAGAACAATGCTCCCCAACTGATTGAGCCCATCCCATACGAGTTCATTGCA---GCGCTCAACGAGGTTGTCA

CCCGTGACTACACCATCCATCTCCATAAGCGCATCCATGGCGTGAGTTTCAAGAGGAGGGCTCCACGTGCCATCAAGGAG

ATCCGCACCTTTGCCACCAAGATGATGGGCACAGATGACATCAGGATTGACACCAGGCTCAACAAGCACATCTGGTCTCA

GGGTGTCAGGAGTGTTCCCTTCAGGGTGAGGGTAAGGCTTGCCAGGAAGAGGAACGAGGATGAGGATTCCCCACACAAGC

TCTACACCCTCGTCACACACGTAGCTGTCCCATCATTCAAG---ACTCTCGTAACCAGGACACAGGGAACAAAAATCGCC

TCTGACGGTCTGAAGGGACGTGTGTTTGAGGTCTCTCTGGCTGATCTGCAGAACGATGAGGTTGCATTCCGCAAGTTCAG

GCTGATGGTTGAAGATGTCCGCGGGAAGAACTGCCTGACCAACTTCCATGGCATGAACCTGACCAGGGACAAGTACTGCT

CCATGGTCAAGAAGTGGCAGACTCTGATCGATGCCAACATTGACGTGCGTACCACCGATGGCTACCTTCTGCGTCTGTTC

TGCATTGGCTTCACCAAGCGCCAGAACCAGATCAAGAAGACGTCCTACGCCCAGAGCACCCAGGTCCGTGCTATCCGCAG

GAAGATGATTGAGATCATGGCCCGCGAGTGCGCAGCCAACGACCTCAAGGAAGTCGTCAACAAGCTGATCCCAGACAGCA

TTGCCAGGGACATTGAGAAGGCTTGCCATCGCATCTACCCTCTCCATGATGTCCACATCCGCAAGGTCAAGGTCCTCAGG

AAGCCCAAGTTTGATGATGACACCGGAGCTCAGGTCGACAGGCCAGACGGCTACGAACCACCAGTCCAAGAAAGCGTC--

-GGTCACCCTCTGGAGCTCGTCCAGCCTCTGGCCCTCCAATCAAAGCTTATGGAGCCTGTGTATCTCCTGGGTAAGGACC

GCTTCTCTGCTGTAGACATCAGGGTGCGAGTCAAGGGAGGTGGACGTATCGCACAGATCTATGCCATCAGGCAAGCTATC

TCCAAGTCTCTGGTTGCTTATTACCAGAAGGTTGACGAAGCCTCCAAGAAAGAGATCAAGGACATCCTGATCCAATACGA

CCGAGCGCTCCTGGTCGCTGATCCCAGGCGCTGCGAGCCCAAGAAGTTTGGTGGTCCAGGAGCCCGTGCCCGCTACCAGA

AGTCCTACCGT---CGATATGCCAACATTTGCTGTCGCAAGGCTGACATCGATGTGTCAAAGAGGGCAGGAGAACTCACC

GATGATGAGAAGTTGATGACCATCCTGCAGAACCCCCGCCAGTACAAGATCCCCGACTGGTTCCTCAACAGGCAGATGGA

CGTCAAGGACGGCAAGTACGGTCAGATGATGGCCAACAACCTGGACAACAAGCTCCGTGAGGATCTGGAGCGCCTCAAGA

AGATCAGGGCTCACCGTGGTCTCCGACATTACTGGGGTCTGCGTGTGAGGGGACAGCACACCAAGACTACTGGCAGGAGA

GGAAGGACTGTTGGTGTGTCCAAGAAG---ATGAAGGTTCCTGATTGGGTCGACATCGTCAAGACTGGAACACACAAGGA

ACTGGGACCTGTTGATCCCGACTGGTTCTACACAAGGGCTTCTACCGCCCGTCACCTGTACTACCGTGGTGTAGGAGTTA

ATGCTATCGCCAGGATCTATGGAGGACGTATGCGCCGTGGAACGCGCCCCAGCCATTTCCACAGTGGCTCCACCTCCGTA

GCCCGTAAGGTTCTGCAGGCTCTGGAAGGCGTCCAGATCATCGAGAAGGAAGGCAACTGCGGACGTCGCATCACCAGCCA

GGGACAGAGGGATCTGGACAGGATAGCAGCACAGGTC

>Bfloridae

TCTCACCGAAAGTTCTCGGCGCCACGCCACGGCTCCCTGGGTTTCCTGCCCCGCAAGAGGTGCAGGCGTGCCAAGGGAAA

AGTCAAGGCTTTCCCCAAGGATGATGCCAGCAAGCCGCCCCACCTTACCGCCTTCATGGGCTACAAGGCCGGCATGACCC

ACGTGCTGAGGGAAGTGGAGAGAACCGGAGCGAGTATGAACAAACGAGAAGTGGTGGATGCTGTGACCATCATAGAGACC

CCTCCCATGGTGGTGGTAGGCGTGGTCGGCTACATCGCCACACCCAAAGGCAGGCGTGCCTTCAGGACCATCTTTGCTGA

ACACCTGAGTGAAGAGTGCAGGCGCCGCTTCTACAAGAACTGGTACTCCTCCAAGAAGAAGGCTTTCACCAAGTACGCCA

AGAAGTGGCAGGACGATGACGGCAAGAAGCAGATTGAGAAGGACTTCAAGCAGATGAAGAAGTACTGCATCAACATCCGT

GTCATCGTCCACACTCAGATGAAGGACCTGCCTCACCGCCAAAAGAAGTCCCACATCATGGAGATCCAAGTGAACGGTGG

CACCATCGCGGAGAAGGTGGACTGGGCGCGGGAACACCTGGAGAAGCAGATCCCCGTCTCCCAGGTCTTCGGTCAGGACG

AGATGATTGACATCATCGGCATCACCAAGGGAAAGGGCTTCAAGGGTAAGGAATCCCGTTGGGGAACCAAGAAGCTGCCC

CGTAAGACCCACAAGGGTCTGCGTAAGGTCGCCTGTATCGGTGCCTGGCATCCCGCTCGTGTGGGCTACGGTGTGGCCCG

TGCC---CGCCCGTTGATAACGGTGCACAATGAGAAGGGCGAGACCGTGACGATGCCCGCGGTGATGAAGGCGCCGATCC

GGCCCGACATCGTCCAGTCGGTGCATGCCAACGTTCGCAAGAACAGCAGGCAGCCGTACTGCGTCAGCAAGCTCGCAGGT

CACCAGACAAGCGCTGAGTCCTGGGGAACTGGCCGTGCCGTGGCACGTATCCCCCGTGTTCGCGGTGGCGGAACCCACCG

CTCCGGCCAGGGTGCGTTCGGCAACATGTGCCGCGGCGGGCGCATGTTCGCTCCCACCAAGACGTGGCGGCGCTGGCACC

GCCGTGTGAACATCAACCAGCGCCGTTACGCCATCTGCTGTGCTCTGTCCGCCTCCGCTCTCCCTGCACTAGTCATGGCA

AAGGGTCACCGTATCGAGGAGATCCCCGAGGTTCCCCTGGTGGTAGGAGACAAGGTCCAGGAGATGAAGAAGACCAAGGA

GGCCGTCCAGCTGCTCCGCAAGCTCAAGGCCTGGAACGACATTCAGAAGGTTTATAACAGCGACAAGGACAACGGTATCG

TCAGAGCCTTCAGGAACATACCAGGCATAACATTGCTGCCAGTGGACAAGTTGAACCTCCTGAAGATCGCTCCCGGTGGT

CATCTCGGCCGTTTCCTCATCTGGACAGAGAGCGCCATCCAGAAACTGGACGCTCTGTAC---CGTAGAGAGGGGGTAAC

TGACTACTATGCCAGGAAACGTTTGGTGATCCAAGAGAAGAACAAGTACAATACGCCTAAGTACAGGCTTATAGTGCGCT

TCACCAACAAGGACATCATTTGTCAGGTAGCGTATGCCAGGATAGAGGGAGACATCATTGTGAGTGCTGCGTACGCACAT

GAACTTCCTCGCTATGGCATTAAGGTCGGGTTGACAAACTATGCAGCTGCATATTGCACCGGACTCCTGCTCGCTCGCAG

GATTTTGAAGAAATTCAGCCTTGACACCATCTACGAAGGCTGCACTGAGATCGACGGTGACGAGTACAACGTTGAGGTGG

ACGGGCAGCCCCGCCCGTTCCGCTGCTTCCTGGACGTGGGTCTCGTCCGCACCAGCACCGGTGCACGCGTCTTCGGCGCC

ATG---CAGAACCCACTGTTCGAGAAGAGGCCTCGCAACTATGGCATCGGCCAGGACATCCAGCCGAAGCGCGACTTGAG

CCGTTTCGTGCGTTGGCCTAAGTACGTTCGTCTCCAGCGTCGGGCGATCCTGTACAAGCGTCTGAAGGTTCCACCACCAA

TCAACCAGTTCACACAGGCACTAGACAAGCAGACAGCCACACAGCTGTTCCGCCTTGCTCAGAAGTACCGCCCAGAGACC

AAAGTCGAGCGCAAGAAGCGTCTCATGGAGCGTGCCAAGAAGCGCGTGTCTGGTAAGGAAGACGTCCCAACAGAACGCCC

CGCTGTCATACGTTCTGGTGTCAACACGATCACCACCCTGGTGGAGTCCAAGAAGGCACAGCTGGTGGTCATCGCTCATG

ATATCGACCCTATCGAGTTGGTGCTCTTCCTGCCAGCGCTTTGTCGGAAAATGAACGTTCCCTACTGCATCGTCAAAGGG

AAGGCACGTCTGGGCAGACTGGTGCACCGCAAGACCTGCACCTGTGTAGCCTTCAGTAAAGTTAACGAGGACAAGGGACA

ACTAGCCAAGCTGATTGACGCTGCCAAGAACAACTACAACGAAAGATTTGACGAGATCCGCCGGCACTGGGGTGGTGGTG

TCATGGGCAACAAGTCGCTTGCCCGC---ATGGGGCGTGTGGTCTTTACCGCCCACACTAAACATCGCAAGGGGGCTGCC

CTCCGAGCCCTGGACTTCGCAGAGAGACATGGATACATCAAGGGTCTAGTCAAGGATATTATCCACGACCCTGGCCGTGG

TGCCCCTCTAGCCAGAGTGATGTTCAGGGATCCTTACCGGTACAAGAAGCGCACGGAGACATTCTACTGTGGCAAGAAGG

CCATCCTGAAGGTTGGAAACACCCTGCCTGTGAGTCAGATGCCTGAAGGGACCATCATCTGTAACGTAGAGGAGAAGGGC

GGAGACCGTGGTTCTTTGGCCCGCTGCTCCGGAAACTACGCCACCGTCATCTCCCACAACCTGGAGAACAAGACCACCCG

CATCAGACTGCCGTCAGGCTCCAAGAAGGTGGTGGCATCTGGCAACAGGGCTACCATCGGTATTGTTGCTGGAGGTGGTC

GTATTGACAAGCCCATGCTGAAGGCTGGCAGGGCGTACTTCAAGTACAAGGCCAAGAGGAACGCCTGGCCACGTGTTCGT

GGTGTGGCTATGAACCCTGTTGAGCATCCCCATGGTGGTGGTAACCATCAACATATCGGTATGCCCTCAACTGTACGGAG

G---AGCGGTGACAGACTGACACGTGCCGCCAAGGTGCTGGAACAGCTGACAGGACAGACCCCCGTCTTCTCCAAAGGTA

CATACACAGTGAGGTCTTTCGGCATCCGTAGAAACGAGAAGATCGCGGTCCACTGTACGGTGCGTGGCGCCAAGGCAGAG

GAGATCTTGGAACGCGGGCTGAAGGTCAAGGAATACGAGTTGCGGAAGAACAATTTCTCGGCC---GTGTACAGACGGTT

TGTCGAGATCGGCCGTGTGGCCTTCGTGCGTTTTGGGCAGGATGAGGGCAAGCTGTGCGCCGTGGTCGACATTGTAGACC

AGAACAGGGCAGTGGTACAGACTCCATGCAGTCCAATCCGCCGCCAGGCCATCAGCTTCAAGTTCCTCCACCTGACCCCA

GTTGTGATCACCATCGGACACGGCTGCGGCACCAGCGCCGTGAAGAAGGCCTGGGAGAAGGAGGGTGTGGAGGAGAAGTG

GCGCCAGTCCACCTGGTACAAGAAGCTCGACCGCTTCAAGTTGATG---TACAAGTATGTACAAGAATTATGGCGGAAGA

AGCAGTCGGACGTGATGCGGTTTCTCCTCCGTGTCCGCTGCTGGCAGTACCGTAACCTGTCTGCGATCCATCGCGCACCC

CGCCCCACGCGTCCGGAGAAGGCCCACAGGCTGGGATATAAGGCCAAGCAAGGGTACGTTATCTACCGTGTACGCCTCCG

CCGTGGTGGCCGCAAGAAGCCCGTGCCCAAGGGTGCCACGTACGGAAAGCCTGTCCACCAGGGAGTCAACCAAATGAAGT

TTGCAAGGAACCTTCGATCTGTCGCTGAGGAGAGAGTTGGCCGTGCCTGTGGTGCCCTGCGTGTGATGAACAGCTACTGG

GTGGGCCAGGACTCAACCTATAAATTCTTCGAGGTTATCTTGGTTGATCCATTCCATAAAACTGTCCGTCACGACCCAGA

AGTACAATGGATCTGCAAGCCGGTCATGAAGCACCGCGAGCTGCGCGGCCTGACGTCGGCCGGCAAGAGGTCGCGCGGCC

TGGGCAAGGGTCACTTCCACCACAAGACAAAGGGCGGGTCCTGGAGGGCCTCCTGGAAGCGGCGCAACACCCTGTCGCTC

CGCCGATACCGC---CGCTTCCTGGCCAGGCGTACCAATGCCAAGTTCAACCAGGTGGTGCTGAAACGCCTGTTCATGTC

CCGCACCAACCGCCCTCCCATGTCTATAGCACGACTGCGTCTGATGAAGAAGCCCGGCCGCGAGGGTAAGACTGCCGTTG

TTGTTGGCACCATCACTGATGACGTCAGGATCTTTGAAATCCCCAAGCTGAAGATTTGCTGCCTTCGTGTGACAGAGGGA

GCCCGTGCCAAGATCCTGAAGGCTGGTGGGGAGATCATCACCTTTGATCAGCTGGCTCTAAGGTCTCCCAAGGGGAAGAA

CACTGTTCTTATTCAAGGTCAACGTAGGGCTCGTGAGACTTGCAGGCACTGGGGCCGAGCTCCTGGTGCACCTCACAGCC

ATACCAAGCCGTTT---AAAGAGTTCTGCGTGGTGGGGCGTCGTCTTCCCAGCAAGACGCAGGGCACCCCACCCCTGTAC

AAGATGCGCATCTTCGCCCCCGACCATGTCGTGGCCAAGTCTCGCTACTGGTACTTCATGAAGCAGCTCAAGAGTGTGAA

GAAGGCCAACGGTGAAGTTGTGTCATGTCAAAAGGTGCAGGAAAAGAACCCCACCAGCATCAAGAACTACGGCATCTGGC

TGCGCTATGACTCCAGGAGTGGGACCCACAACATGTACAGGGAGTACCGTGACCTGACTGTGGCCAGCGCTGTCACCGCC

TGCCGTGACATGGGTGCCCGTCATCGTGCCCGCGCTCACTCCATCCAGATTATGAAGATCGAGGAGATCCCGGCAGCGAA

GTGCCGCAGACCTAGCGTCAAGCAGTTCCATCAGTCCAAGATCAAGTTCCCTCTGCCCCATCGTGTGACCAAGCTGCACA

ACCCACGCTTCACCACCAGGCGTCCCAACACT---CTACGCTTACAGAAGCGGCTGTCAGCCAGTGTCCTTAAGTGTGGC

AAGAACAAGGTCTGGTTGGACCCCAACGAGACAAATGAAATCGCCAACGCCAACTCAAAGCAAAACATCAGGAAGTTGGT

AAAAGATGGTCTGATCATCCGTAAGCCGGTGGCTGTCCACTCCCGGGCACGTGTCCGCAAGGCGCACATCGCCCGCCGCA

AGGGCAGACACCCTCACCCAGGTAAGAGGAAGGGTACAGCCAACGCACGTATGCCAGAGAAGGTCATCTGGATGCGCCGT

ATGCGTGTTCTGCGCAGAATGCTGCGGAAATACCGCGAGTCCAAGAAGATCGACAAGCATCTGTACCACCAGCTGTACAT

GAAGGCCAAGGGTAACGTGTTTAAGAACAAGCGACTGATGATGGAGTACATCCACAAGAGGAAGGCCGAGAAGCAGCGAT

CCAAGCTGTTG---GACAGATATGACAAACTATGGCGTAAGCCCAAGGGTATCGACAACCGTGTGCGTAGGCGCTTCAAG

GGCCAGTACCTCATGCCCAACATTGGTTATGGGAGTGCCAAGAAGACAAAGCACCTTCTGCCCTCCGGCTTCAAGAAATT

CCTCGTTCACAATGTCAAGGACCTGGAGGTCTTGTTGATGCAGAACCGAACCTTCTGTGCCGAGATCGCTCACAACGTGT

CATCACGCAAGAGGAAACTCATCGTGGAGCGCGCCCAGCAGCTGGCCATCAAGGTCACCAACCCCAACGCTCGTCTC---

CAGCTGCGGGTCGCCAAGGTGACGGGCGGAGCTGCTTCCAAGCTCTCCAAGATGGTTCGCAAGTCCATTGCCCGTGTCAT

GACAGTCATCCACCAGACACAGAAGGAAAACCTCCGCAAGTTCTACAAGAAGAAGAAGTACATCCCTCTGGACTTGCGCT

ACAAGAAGACCAGAGCGATCCGACGTGCACTAACCAAGGGCGAGAAGAACATCAAGTCCAAGAAGACACAGAAGAAGCTC

CGGTTGTACCCCATGAGGAAGTATGCCGTCAAG---TCCAATCAGATGCAGCGGATCCGCATGTCCCTCCGAGGATCAGA

GATTCTCATGGGCAAGAACACCATGATGCGGAAGGCTATCCGTGGGCACATGGAGAACAACCCAGCCCTGGAGAAGATCC

TCCCTCACATCTCGGGCAATGTTGGCTTCGTCTTCACCAAGGGAGACCTGAAGGAAGTTCGTGACAAGATCCTGGAGAAC

AAGGTAGAAGCTCCTGCCAAAGCTGGTGCTATTGCTCCGGTGGACGTTTACCTGGACCCACTGAACACTGGCCTGGGTCC

GGAGAAAACCTCCTTCTTCCAGGCTCTGTCCATCCCCACCAAGATCTCAAGGGGCACCATTGAAATTCTGGTAAATGTGT

ACCTGATCAAGAAGGATGAGAAGGTGGGGGCTTCTGAAGCAACCTTGTTGAACATGTTGAAGATTTTCCCCTTCACATAT

GGGTTGGGGATCCAAGTGTATGACAGTGGGTCAGTGTTCCATCCATCCATCCTGGACATCACTGATGATGACATCCTAGC

CAAGTTCATGCAGTTGCAGGGTAACGTCGCCAGTGTCTGCCTG---ACCCGTGCCGGTCAGCGCACCAGGTTCAAGGCCT

TTGTGGCAATCGGTGATTCCAACGGCCACATTGGGCTGGGTGTGAAGTGCTCCAAGGAAGTGGCGACAGCCATCCGTGGT

GCCATCATCCTGGCCAAGCTGTCCATTGTACCTGTCAGGCGAGGCTACTGGGGAAACAAGATCGGCAAGCCCCATACTGT

GCCTTGCAAGGTGACAGGGAAGTGTGGCAGTGTTCTGGTGCGTTTGATTCCCGCACTCCGTGGTACTGGCATCGTGAGCG

CTCCTGTTCCAGAGAAGCTGCTGCAAATGGCGGGTATCGATGATTGCTACACCTCGGCTACAGGCTCTACCCGCACACTG

---CTGGCCGAGGATGGCTACAGTGGCGTGGAAGTCCGTGTAACCCCCACACGGACTGAGATCATCATCCTAGCGACTCG

CACACAAAATGTTCTAGGCGAGAAGGGTCGTCGGATCCGCGAGCTCACCTCAGTGGTACAGAAACGTTTTGGCTTCGCCG

AGGGCACTGTCGAGCTGTATGCTGAGAAGGTTGCCCAGCGTGGTCTGTGTGCCATCGCCCAGGCTGAGTCTCTTCGGTAC

AAGCTACTCGGTGGTCTTGCTGTCAGAAGGGCGTGCTATGGTGTGCTGAGGTTTATCATGGAGTCAGGCGCCAAGGGTTG

TGAGGTTGTTGTGTCGGGTAAGCTGCGTGGACAGCGTGCCAAGTCCATGAAGTTTGTGGATGGTCTGATGATCCACAGCG

GCGAACCCGCCAACTCATACGTGGACTCTGCTGTCAGACACGTCATGTTGCGCCAAGGTGTCCTGGGCATCAAGGTGAAG

ATCATGTTGCCGTGGGACCCAACTGGCAAGACTGGCCCCAAGAAACCTCTGCCTGACCACGTCAGTATCGTGGAGCCTAA

AGATGAG---GGTCCGAAAAAGCATTTGAAGCGCCTTGCTGCGCCGAAGCACTGGATGTTGGACAAGCTGTCCGGCGTTT

TCGCTCCTCGCCCGTCCTGTGGTCCCCACAAGCTGCGCGAGTGCCTCCCGCTCTGCCTGTTCCTCAGGAACCGACTCAAG

TACGCGCTGACCTACGATGAGGTCAAGCGAATCCTCATGCAGCGCCTCATCAAGGTGGACGGCAAGGTCCGCACCGACCG

CTGCTACCCCTCCGGATTCATGGATGTGATCACCATTGAGAAGACTGCCGAGAACTTCCGGCTGATCTACGACGTGAAGG

GCCGTTTCACCATCCATCGGATCACTCCTGAGGAGGCCAAGTACAAGGTGTGCATGGTCACCGGAGGCCGTAACTTGGGG

CGTGTCGGGCTGGTCACGAACCGCGAGCGCCATCCCGGATCGTTCGACATTGTTCACGTGAAGGACACAGCCGGGCACAC

CTTCGCCACCCGTCTCGCCAACATCTTCATTATCGGACGGGGTAACAAGCCGTGGGTGTCGCTGCCGAAGGGCAAGGGTA

TTCATCTGTCGATCGCTGAGGAGCGCGACAGGCGGCTGGCCATG---ATCAACATTTCGTACCCGGCCACGGGCTGCCAG

AAGCTCATCGAGGTGGACGATGAACGCAAACTTCGTCCTTTCTACGAGAAGCGCATGTCTCATCAGATGACAGCTGAAAG

CCTTGGAGATGAATGGAAGGGTTATCTGGTGAGGATCAGCGGAGGCAACGACAAGCAAGGCTTCCCCATGAAGCAGGGTG

TTCTGACCAACGGCCGTGTGCGTCTGCTGCTGGGGAAGGGCCACTCCTGCTACCGGCCACGCCGTACCGGAGAGCGCAAA

CGCAAGTCTGTCCGAGGCTGCATCGTGGATAGTAACCTGTCGGTGCTCAACCTTGTCATCCTCAAGAAGGGTGAGCAGGA

CATTCCGGGTCTGACCGACACCACCATCCCTCGCCGTTTGGGCCCCAAGCGTGCCGGCAGGATCCGCAAGCTCTTCAACC

TCAACAAGGAGGACGACGTGCGTCAGTACGTGGTCTCTCCTGTAAGAAGCAGAAGTTCAAGACTCCAGCGACTGATCACC

CCTCAGCGCCTG---ATCTCCCGTGACAACTGGCACAAGAGGCGCTCGACAGGAGGTCGGCGCAACCCCATCAGGAAGAA

GAGAAAGTACGAGCTCGGCCGTCCGCCAGCGAACACCAAGCTTCTTGTACGATGCAGAGGCGGGAACACGAAGTTCCGCG

GACTGCGTTTGGATACTGGCAACTTTTCCTCGGGGGCCATCTCCCGTAAGACGCGTGTCATCGATGTCATGTACAACGCC

AGCAACAACGAGTTGGTCCGTACCAAGACGCTGGTGAAGGGTGCCATCGTACAGATCGACAGCACGCCCTTCCGCCAGTG

GTACGAGGCACACTACGCTCTTCCTCTCGGACGCAAGAAGGGCCAGAAAGCTTGCATCGCGTCGAGACCAGGACAGGTCG

GGCGGTGTGACGGCTACATTCTTGAAGGGAAGGAACTAGAGTTCTACTCCAGGAAGATCAAGGCCAAGAAG---AAGACC

TACGCCACACCCCGACGTCCTTTTGAGAAGGAACGTCTGGATCAGGAGTTGAAGCTCATTGGGGAATATGGGCTTCGTAA

CAAGCGTGAGGTTTGGCGTGTGAAGCTGACCCTCGCTAAGATCCGTAAGGCTGCTCGTGAGCTGCTGACACTGGAGGAGA

AGGACCCCAGACGTCTGTTTGAGGGTAATGCCCTACTGCGCCGACTGGTGCGTGTTGGTGTGCTGGATGAGGGGAAGATG

AAGCTTGATTACGTGCTGGGCCTGCGCGTGGAGGACTTCTTGGAGCGACGTCTCCAGACCCAGGTCTTCAAGCTGGGATT

AGCCAAGAGTATCCACCACGCCCGCGTGCTGATCCGCCAGAGGCACATCAGGGTACGTAAGCAGTTGGTGAACATCCCAT

CCTACGTGGTGCGCGTGGACTCCCAGAAGCACATCGACTTCAGCCTGCAGTCTCCGTTCGGAGGTGGT---TTCCAGAAG

CAGCCCACCATCTTCTTGAACAAGAAGCGCTATGTTAGAAACGTCGGCCTGGGCTTCAAAACTCCCAGAGATGGGACGTA

CATTGACAAGAAGTGCCCGTTCACTGGCAATGTGTCCATCCGTGGGCGAATCCTGACAGGCATCGTTGTGAAGCTGAAGA

TGCAGCGCACCTGTGTGATCCGCAGGGACTTCCTCCACTACGTCAAGAAGTACAACCGTTTCGAGAAGCGCCACAAGAAC

GTGTCTGTGCATCTTTCGCCTGCCTTCAGGGATGTGGCGCTAGGTGATGTGGTGACTGTTGGCGAGTGTCGGCCACTCAG

CAAGACCGTG---GGTATCTCCTCCTCAGCCCTCCCGTACCGACGCAGTGTGCCCACGTGGCTGAAGCTGTCTGCTGATG

ATGTGAAAGACCAGATCTACAAGCTGGCCAAGAAGGGCCTTACACCCTCACAGATAGGTGTGATCCTCCGTGACTCCCAT

GGAGTGGCTCAGGTCAGGTTCGTGACGGGAAACAAGATCCTGCGTATCCTGAAGTCCAAGGGCCTGGCACCTGACCTCCC

AGAGGACCTGTACTTCCTCATCAAGAAGGCTGTGTCCATCCGCAAACATCTGGAGAGGAACAGGAAGGACAAAGACGCCA

AGTTTCGCCTTATCCTTGTGGAGAGCCGTATCCACCGGTTGGCACGTTACTACAAGACCAAGCTGGTGCTGCCACCCAAC

TGGAAG---ATGGTACGTATGAACGTGCTGGCTGATGCGCTCAACGCCATCAACAATGCGGAGAAGCGTGGTAAGCGTCA

GGTGATGCTGAGGCCCAACTCGAAGGTCATTGTCAAGTTCCTGACTGTCATGATGAAGCATGGTTACATCGGTGAGTTTG

AGATCGTGGACGACCACCGAGCCGGCAAAATCGTGGTGAACCTGAACGGCCGGATGAACAAGTGTGGCGTGATCAGCCCC

CGCTACGACGTGGCCGTGCGAGATCTGGAGCGCTGGCAGACCAGACTCCTGCCCTCCAGACAGTTTGGGTACATTGTGCT

GACTACGTCTTCAGGCATCATGGACCACGAGGAGGCCAGGAGAAAGCACACAGGAGGGAAAATCTTAGGCTACTTCTTC-

--GAGACCCAGATCCACCGCATCAGGATCACACTCACCAGCCGCAACGTCAAGAGTCTGGAGAAAGTGTGTGCTGATCTG

ATCCGTGGTGCTAAGGAAAAGAACCTCCAGGTGAAGGGGCCCGTCCGCATGCCCACAAAGGTCCTGCGCATCACCACCCG

TAAGACCCCCTGTGGTGAGGGCTCCAAGACCTGGGACCGCTACCAGATGCGCATCCACAAGCGCCTGATCGATCTGCACA

GTCCCTCTGAGATCGTCAAGCAGATCGTATCCATCAGCATCGAGCCTGGTGTTGAGGTGGAGGTGACCATCGCTGAT---

ACTGTAAGAACGAGGAAATTCCTCACCAACCGCTTGATGCAGCGGAAGCAGATGGTCGTGGATGTTCTGCATCCGGGGCG

AGCCACCATTCCCAAGACAGAAATCCGCGAAAAGCTCGCCAAGATGTACAAGACCACACCCGACGTCGTCTTTGTCTTTG

GGTTCCGCACCCAGTTTGGCGGTGGCAAGACCACCGGCTTTGGTCTGATCTACGATACCTTGGACTTCGCAAAGAAGATG

GAACCCAACTACAGGCTGGCCAGG---GGTAAGCCCCGTGGACTGCGTTCTGCCCGTAAGCTGAAGGATCACCGCCGGCA

GCAGCGCTGGCACGACAAGTCCTTCAAGAAGGCCCATCTGGGTACTGCTGTGAGCCCCTTCGGAGGAGCCTCTCACGCTA

AGGGCATCGTGCTGGAGAAGATGGGTGTTGAGGCCAAGCAGCCTAACTCTGCCATCAGGAAGTGCGTCCGTGTCCAGCTT

ATCAAGAACGGCAAGAAGATCACAGCCTTCGTACCCAACGACGGCTGCCTCAACTACATCGAGGAAAACGACGAGGTTCT

GGTTTCTGGATTCGGTCGTAAGGGCCGTGCCGTGGGTGACATCCCCGGTGTCAGGTTCAAGGTCGTGAAGGTCGCCAACG

TGTCGCTCCTCGCCCTGTTCAAGGAGAAGAAGGAGAGGCCCAGATCGATG---CGTGAGGCCAAGAAGCATGGAAACTTC

TACGTCCCGCCCGAGGCCAAGCTGGCTTTTGTTGTCAGGATCCGTGGTATCAATGGTGTCAGCCCAAAGGTACGCAAGGT

TCTGCAACTTCTGCGACTGCGCCAGATATTCAACGGAACCTTTGTGAAACTGAACAAGGCCTCTCTGAACATGCTGAAGA

TTGCTGAGCCTTACATTGCTTGGGGATACCCAAACCTGAAGACAGTGAGGGAACTCATCTACAAGCGGGGCTATGGCAAG

ATCCAGAGAATTGCTCTGACAGACAATCTCATTGAAGAGCATTTAATCATCTGTATTGAGGACCTCATCCACGAGATCTT

CAGTGTTGGTCCAAACTTTAAGGCTGCCAACAACTTCCTGTGGCCATTCAAGCTATCGTCACCCCGCGGGGGGATGCGAA

AGAAGGGCCACCACTTTGTAGAAGGCGGGGATTTTTGGAACCAG---AAGAACAAGAAGCGCAAGTTTGTGGAGACCGTG

GAACTTCAGATCGCCCTGAAGAACTATGACCCCCAAAAGGACAAGCGTTTCTCTGGCACCGTCAAGCTGAAGCACACGCC

GCGTCCCAAGTACCGCGTGTGCGTGCTGGGCGACCAGCAGCATATCGACGAGGCCAAGGCCAACGAAATCCCCTGCATGG

ACGTGGAATCCCTGAAGAAGCTCAACAAGAACAAGAAGCTTGTCAAGAAGCTCGGTAGGAAGTATGATGCCTTCCTGGCG

TCGGATTCTCTGATCAAGCAGATCCCCCGTATCCTGGGCCCAGGTCTGAGTAAGGCTGGCAAGTTCCCCACCCTCCTGAC

TCACAACGAGTCCATGACGGGCAAGGTGGAGGAGGTCAAGTCCACCATCAAGTTCCAGATGAAGAAGGTGCTGTGCTTGT

CCGTTGCCATCGGACATGTCAACATGTCTGCGGAGGACCTGGTCGCCAACGTGGTTCTGGCCGTGAACTTCCTGATCTCC

CTGCTGAAGAAGGGCTGGCAGAACGTCAGGGCACTGTACATCAAGTCCACCATGGGCCCCTCTCAGCGCCTGTAC---GG

CCATTTCCACAAGGATTGGCAGAACCGTGTGCGGACATGGTTTAACCAGCCGGCCAGGAAGATCCGAAGGCGCAAGTCCC

GTGTGGAAAAGGCCCGCCGCGTCGCTCCCCGCCCCGCTGGCGGACTGCGCCCGATCGTCAGGTGCTCAACGTTCAAGTAC

CACACCAAGGTCCGGGCAGGGCGTGGCTTCACCCTGGAGGAGATCAAGGCAGGTCTGAACAAGAAGTATGCCCGCACCAT

CGGCATCTCAGTGGACCCCAGGAGGCGTAACAAGTCGACCGAGTCTCTGCAGGCCAATGTCCAGCGCCTCAAGGAGTACA

AGTCCAAGCTCATCCTCTTCAAGAAACTCAGCGCACCC---ATGACTAGGTACTCACTGGACCCGGAGAACGCCACAAAG

TCATGTAAAGCCCGTGGATCCAACTTGCGGGTGCACTTCAAGAATACCCGCGAAACGGCCAATGCAATCAAGGGCATGCA

CATCCGCAAGGCCACGCGTTTCCTGAAAGATGTCACTGCCAAAAAGCAGATCGTGCCCTTCCGCAGGTACAATGGTGGTG

TGGGACGCAAGGCACAGTGTAAGGCCTGGCGCTGGACTCAGGGACGGTGGCCCAAGAAGAGTGCAGAATTCCTGCTGCAA

CTGCTGAAGAATGCAGAGAGCAATGCTGAATTCAAGGGTCTGGATGTGGACTCCCTGATGGTGGAACACATCCAGGTGAA

CCGGGCTGCCAAGATGAGGCGCAGGACATACAGAGCCCACGGCCGTATCAATTACATGAGCTCTCCCTGCCACATCGAGA

TGATCTTG---ATGAAGTTCAACAAGATGGTGTCCTCCTCCCGGAGGAAGAACCGCAAGCGTCACTTCAACGCGCCCTCC

CACGTCCGCAGGAAGATCATGAGCTCGCCCCTGTCCAAGGAGCTCCGCGCCAAGTACAACGTGCGTGCCATGCCCATCCG

CAAGGACGACGAGGTTCAGGTGACCAGAGGTCACTACAAGGGCAGCCAGGGTAAGGTCGTGTCCGTCTACAGAAAGAAAT

TTGTCTGCTACATTGAGAGAATCCAGCGCGAGAAGGCCAACGGTGCAACCGTGTACGTCGGTATCCACCCTTCTAAGATG

GAGATCACGAAACTTAAGATGGACAAGGACCGTAAGGCTATCATCGACCGTAAGGCTGCCAGCAAGCAGAAG---TCGGG

GAAAGTCGTCCTCGTCCTGAAAGGGAAGTATGCAGGCAGGAAAGCTGTCATCGTGAAGAACTATGATGAGGGTACCCACG

ACAGACCCTATGGCCATGCGCTGGTGGCTGGCATGGACCGCTACCCTCGCCGCGTTACCGCCAGGATGGGCAAGAAACGC

CTGGCCAAGAGGTCCAAGGTCAAGGCTTTCGTGAAGGTGTGCAACTACAACCACATCATGCCCACAAGATACTCTGTTGA

TGTTAGATACAAGACCGGCAAGAACAAGTGGTTCTTCCAGAAGCTACGCTTC---CTGCGTGGCCACGTCAGCCATGGCA

AGGGGCGTATCGGCAAGCACAGGAAACATCCAGGTGGCCGTGGTAACGCCGGAGGACAGCACCACCACAGGATTAACTTT

GACAAGTTCCATCCCGGGTACTTCGGAAAGGTGGGCATGCGTCACATCCATCTGAGGAGAAACCAGCAGCATTGTCCTGC

CGTCAACCTGGACAAGCTGTGGACGCTCGTCAGCGATCAGACACGCGAGGTGGCTCCCGTCATCGACGTCGTGAGAGCGG

GATACTACAAAGTCATCGGGAAGGGCCGTCTGCCCAAGCAGCCCGTCATCGTGAAGGCCAAGTTCTTTTCTCGCCGAGCT

GAAGAGAAGATAAAGGACGTTGGCGGATGCTGCATTCTCGTGGCA---ATGGGAGCCCACAAGACATTCCGCATCAAGAA

GAAGCTTGCTAAGAAGCAGAAGCAGAATCGTCCTATCCCACAGTGGGTCCGCATGAAGACTGGCAACACCATCAGGTACA

ATGCCAAGAGACGTCACTGGAGGCGTACCAAGCTCGGCCTC---ATGGCTCGTACCAAGCAGACCGCCCGTAAGTCCACC

GGAGGCAAGGCTCCCAGGAAGCAGCTGGCCACCAAGGCCGCTCGCAAGAGCGCCCCGGCCACCGGAGGTGTCAAGAAGCC

CCATCGTTACAGGCCCGGCACCGTGGCCCTCAGGGAGATCCGTCGTTACCAGAAGTCCACCGAGCTGCTCATCCGCAAGC

TGCCCTTCCAGCGCCTGGTCAGGGAGATCGCCCAGGACTTCAAGACCGACCTGCGCTTCCAGAGCTCTGCCGTCATGGCT

CTGCAGGAGGCTAGCGAGGCCTACCTGGTCGGTCTCTTCGAGGACACCAACCTGTGCGCCATCCACGCCAAGCGTGTGAC

CATCATGCCCAAGGACATCCAGCTGGCCCGCCGTATCCGTGGCGAGCGTGCT---CTCGCCTGTGTGTACAGTGCCCTCA

TCCTTCACGACGATCACGTCGCCATTACTGGTGATAAGATCCAGGCCCTGATCAAGGCAGCCAACGTGGACGTGGAGCCG

TTCTGGCCG---ATGCGTTACGTGGCCGCATATCTCCTGGCTGTCTTGGGTGGGAATGCCAACCCCTCCGCTGGCGACAT

CAAGAAGATCCTGGGCAGTGTAGGCATCGACGCCGAAGATGAGAGGCTCAACAAGGTCATCGGTGAGCTCAAGGGCAAGG

ACATAGAAGAAGTCATGGCTGCAGGT---GAAAAGGAGTGGATGCCAATGACCAAGCTTGGTCGTCTTGTGAAAGACCTG

AAGATCAAGTCCCTGGAGGATATCTACCTGTTCTCACTTCCCATCAAGGAGTTTGAGATCATTGACTTTCTGTCGGCACT

CAAGGATGAAGTGCTGAAGATCATGCCTGTTCAGAAGCAG---AAGTACCTGCCCCACTCTGCAGGCCGCTATCAGACCA

AGCGCTTTCGTAAGGCCCAGTGCCCTATTGTAGAGCGCCTGACCTGTTCCATGATGATGCACGGCCGCAGCAATGGCAAG

AAGCTCATGGCAATGCGCATCGTCAAGCACTCCTTTGAGATCATCCACCTTCTAACAGGCGAGAACCCACTGCAAGTTGT

AGTGAACGCTATCATCAACAGCGGGCCACGTGAAGACTCCACGCGTATTGGCCGTGCTGGTACCGGTGCTCGTGAGGCGG

CCTTCAGGAACGTGAAGACGATTGCTGAGTGCTTGGCTGATGAACTCATCAATGCTGCCAAGGGATCCTCCAACTCCTAC

GCTATCAAGAAGAAGGATGAG---AGTGCGAAGATCATCAAGCCAGCAGGCGAGAAGCCTGATGAGTTTGAGGCCTCCAT

CTCACAGGCTCTTCTTGAGCTGGAGATGAACTCTGACCTGAAGGCACAGCTCAGAGAACTGAACATCACAGCTGCTAAGG

AGGTCGAAGTTGGATCCAGGAAGGCCATCATCATCTTTGTGCCAGTGCCACAGCTGAAGGCCTTCCAGAAGGTGCAGGTG

CGCCTTGTCCGGGAACTGGAGAAGAAGTTCAGTGGCAAGCATGTTGTCTTCATTGCACAGAGGCGCATTTTGCCAAAGCC

CACGAGAAAGACAACAAAGAACAAGCAGAAGCGCCCTCGCAGCCGCACCCTGACATCGGTGCATGACAACATACTTGAGG

ATCTGGTGTTCCCCAGCGAAATTGTTGGGAAGCGCATCAGGATCAAGCTGGATGGAGCCCGTTTCATCAAGGTGCACCTC

GACATGGCCCAGCAGAACAATGTCGAACACAAGGTGGAAACCTACAGTGCAGTGTACAAGAAGCTGACAGGCAAAGATGT

GACATTTGAATTCCCTGAGTACCAGCTC---ATGCCTAAGAAGAATCGTGTTGCGATCTACGAGCTGCTGTTCAAGGAGG

GCGTCATGGTGGCCAAGAAAGACTACCACCTCCCCAAACACCCCGAGCTAGACGTGCCCAATCTTCATGTCATTAAGGCT

CTAACGTCGCTTAAGTCACGAGGGTACGTGAAGGAACAGTTCGCCTGGCGCCATTTCTACTGGTACCTGACCAATGAGGG

CATCCAGTATTTGCGTGACTTCCTGCACCTCCCGCCAGAGATCGTCCCGGCAACACTGAAG---CACGTCACAGATCTCT

CGGGCAGAGAGACGATTGTCCTTGTGACTGGCGGGATGAAGGTCGAGGCCGATCGTGACGAGGCTTCCCCCTACGCCGCC

ATGTTGGCAGCCCAGGACGTGGCCACCCGCTGTAAAGAAATCGGCATCACCGCCCTGCACATCAAGCTCAGGGCTACGGG

AGGAAACAAGACCAAGACTCCAGGCCCAGGCGCCCAGTCTGCCCTGAGAGCGCTGGCAAGATCGGGCATGAAGATCGGCC

GTATTGAGGACGTG---CTGAAGAAGAAGCGTACCTTCAGGAAGTACACCTACCGAGGTGTCGACCTGGACCAGCTTCTG

GACATGTCCAGTGAGCAGCTGATGGAAATGATGAAGGCTCGTCCCCGGCGGCGCTTCTCCCGCGGCCTGAAGAGGAAACA

TCTCGCCCTCATCAAGAAGCTGCGCAAGGCCAAGAAGGAGTGCCCCGCCCTGGAGAAACCCGAGGTGGTGAAGACACACT

TGAGGAACACCGTCATCGTTCCTGAGATGATCGGCAGCATCGTAGCCGTCTACAACGGCAAGACCTTCAACCAGGTGGAA

GTTAAGCCGGAGATGATTGGGCACTACCTGGGGGAGTTCTCCATCACATACAAGCCTGTGAAGCACGGCAGACCTGGTAT

CGGTGCCACCCACTCCTCACGCTTCATCCCACTCAAG---ATCATCGAAAAATACTACACCAGGCTTACGCTCGACTTCC

ACACAAATAAGCGGATATGTGAGGAGATTGCCATTATTCCCAGCAAGAAGCTGAGGAACAAGGTTGCAGGGTTTGTAACC

CATCTGATGAAGCGTATCCAGCGAGGACCAGTCCGTGGTATCTCCATCAAGCTGCAGGAGGAGGAGAGGGAGAGGAGAGA

CAACTACGTTCCTGAGGTGTCTGCAATTGATCAGGACATCATTGAGGTGGATCCTGACACCAAGGAGATGTTGAAGGCA-

--CCTCGCAAGTGCTCATCAACCAACCGCATCATTGGTGCCAAGGATCATGCCTCAATCCAGATCAACTTTGCTGAGGTT

GACCCGACCACTGGCCGCATGACAGGACAGTACAAGACTTACGCCATCTGTGGCTACATCAGGCAAATGGGAGAGTCCGA

TGACTGTCTGAAGCGCCTGGCAATAAAGGATAAAATCATTGACGCA---CACGTCAGGCCGATCCGCTGCACCAACTGCG

GACGTTGCTGCCCCAAGGACAAGGCCATCAAGAAGTTCGTCATCCGCAACATCGTGGAGGCTGCGGCCGTTAGGGATATC

TCCGAGGCCAGCGTGTATGAAGGTGAGGCACTTCCCAAGCTGTATGCCAAGCTTCACTACTGTGTGAGCTGCGCCATCCA

TAGCAAGGTGGTGAGGAACCGGTCTCGCGAGGCTCGTAAGGACCGCACCCCTCCGCCCAGGTTCCGCCCA---ATGATTT

TCGTGAAAACACTCACTGGGAAAACCATCACCCTAGAGGTGGTGAAGGCCAAGATCCAGGAGGGCATCCCTCCTGACCAG

CAGAGGCTGATCTTTGCCGGCAAGCAGCTGGAAGATGGCGACATCCAGAAGGAGTCCACCCTCCACTTGGGTGGTGCCAA

GAAGAGGAAGAAGAAGAACTACACCACTCCCAAGAAGATCAAGCACAAGAAGAAGAAGGTCAAGCTGGCTGTGCTGAAGT

ACTACAAGGTTGATGAGAACGGCAAGATCACACGTCTAAGACGTGAGTGCCCGTCAGAGGAGTGTGGTGCGGGGCTGTTC

ATGGCTAGCCACTTTGACAGGCAATACTGCGGGAAGTGCTGCCTCACATTTGTGTACAACAAG---AAGCCGTATCCCAA

GTCACGGTTTTGCCGTGGTGTGCCAGACCCAAAGATCCGTATCTTTGACTTGGGCAGGAAGAAGGCACGTGTTGAGGAGT

TCCCACTATGTGTCCATCTTGTGTCTGATGAGTATGAACAGGTGTCCTCTGAAGCTCTTGAGGCGGGGCGTATCTGCTGT

AATAAGTACCTTGTGAAGCATTGCGGCAAGGACGCCTTCCACATCCGCATCCGTGCGCACCCCTTCCACGTCATCCGCAT

CAACAAAATGTTGTCGTGTGCTGGGGCTGATAGATTGCAGACTGGGATGCGTGGTGCCTTTGGCAAGCCTCAGGGCACCG

TGGCCCGTGTACACATCGGCCAGCCCCTGATCTCGTGCCGCGCCAGGGACGCCAACAAGGCCCATGTGATCGAGGCTCTG

CGCCGAGCCAAGTTCAAGTTCCCTGGCCGCCAGAAGATCTACGTGTCCAAGAAGTGGGGCTTCACC---AAGCGTACGAG

GAAGGTGGGAATCGTCGGGAAGTACGGCACCCGTTATGGCGCCTCTCTGCGTAAGACCATCAAGAAGATGGAGGTGTCTC

AGCACTCCAAGTACACATGCACCTTCTGTGGCAAGGAAAACATGAAGAGAAAATCTGTTGGAATCTGGTCCTGCAAGAGC

TGTAGGAAGACCATTGCAGGTGGAGCCTGGGTGTACAGCACAACAGCTGCAGCTACGGTACGGTCAGCGGTGAGACGTCT

GCGGGAACTG---CACATCCAGAAGAAGAGATGTGCCGGCTGCGGTTACCCTAGCAAGAAGATGCGGAAGTACAACTGGA

GCATGAAGGCCAAGAGGAGAAAGATGACAGGTACTGGCAGGATGCGTCATCTCAAGCAGGTGCAGCGTAGATTCAGGAAT

GGTTTCCGTGAAGGAACTACG---ACTGGCAAGGCTTCCTTGTACAAGTTGGGTAAGAGACGTTACGACAGGAAACAGTC

TGGTTATGGTGGTCAAACCAAGCCCATCTTCCACAAAAAGGCTAAAACAACCAAGAAGATCGTGCTGCGTCTAGAGTGCA

CGGATTGTAGAGCCAAGCGTATGGTTGCCATCAAGCGGTGCAAGCACTTCGAGTTGGGCGGTGACAAGAAGAGGAAG---

CTGACCAAGCGTGCGAAGTTTGTGCGTGACCTGGTGCGGGAGGTGACAGGCTTCGCTCCGTACGAGAGACGTACTATGGA

GTTGCTGAAGGTCAGCAAGGACAAGCGCGCTCTCAAGTTCCTCAAGAAGAGGGTGGGAACTCTGCAGCGTGCCAAGAGAA

AGCGTGAGGAGATGCAGAACGTCATCGCCGCCCAGAGGAAGGCTCAG---AAGACGATGGAGAGTATCAACTCCCGGCTG

CAGCTGGTGATGAAGTCTGGGAAGTACGTCTTGGGCCTGAAGGAGACCCTGAAGGTGCTGAGGCAAGGGAAGGCCAAACT

CATCATCATAGCTAACAACACCCCCGCACTCAGGAAGAGTGAGATTGAGTACTACGCCATGTTGGCCAAGACAGGTGTCC

ATCATTACTCTGGTAACAACATCGAGCTGGGTACAGCCTGTGGTAAATACTTCAGAGTCTGCACACTGGCCATCACAGAT

CCAGGT---CGCAAACTCCGCAGCTTCTCCCAGCACAAGAAGAAGCTGCGCCCTTCCATCACACCTGGCACTGTCTTGAT

CTTGGTGTCAGGCAGGCATAAGGGAAAGAGGGTGGTTTTCCTGAAGCAGCTGGCTAGTGGCCTCTTGCTCTTTGCAGGTC

CGTTCAAGCTCAACGCTGTCCCCCTGCGCCGTGTCAACCAGATCTACGTGATTGCCACAAAGACGAAGATCGACATCTCC

GGGGTGAAGCTCCCCTCCCGCCTGAATGACCGGTACTTCAAGCGCAAGAGGCAAGAGGGCGAGATCTTTGACACGGAGGA

AGAGTACTCTGTGAGTCAGGAGAGAAAGGAGGACCAGGTTAAAGTGGACAGTCAGATCCTG---GTCACTGTGAAGGGCA

AAAGGGGAACCCTGAAGAAAAGCTTCAAACATCTCAATGTAAGAATCGTGCGGACTGTCTGCAGCCATGTTAACAACATG

ATTAAGGGTGTCACAGGTTACCGCTACAAGATGAGGTCAGTGTACGCCCATTTCCCCATCAACTGCACCGTGTCCGAGGG

GAACTCCGTCCTGGACATCAGGAACTTCCTGGGTGAGAAGGTGATCCGCCGTGTGAGGATGAGCGAAGGCATCAAAGTCA

TCCTCTCCACAGCCATGAAGGACGAGCTGTACCTGGAAGGGAATGATCTGGAACAGGTCTCTCGCTCAGCTGCCCTTGTC

CAACAGAGCACAACTGTG---GTGTACCTGCGTGCCACAGGAGGAGAGGTGGGCGCCACGGCTTCCCTAGCTCCCAAGAT

TGGACCACTGGGATTGTCTCCTAAGAAGGTTGGAGATGACATTGCCAAGGCTACCCAGGACTGGAAAGGCCTGAGGATCA

CAGTGCAGCTGACCATCCAGAACAGGCAGGCTAAAGTGTCAGTGGTCCCATCAGCCTCCTCTCTTATCATCAAGGCTCTC

AAGGAGCCTCCAAGGGACAGGAAGAAGCAGAAGAACGTCCTGCACAACGGTAACGTCAGTTTTGACGACATTGTGGAGAT

CGCTCGTCAGATGAGGCCCCGCTCCATCGCCCGCAAACTGGAGGGAACCGTGAAGGAAATCCTGGGCACGGCGCAGTCTG

TCGGCTGTACGGTGGACGGGATGCCTCCT---GACGCCAATAACCACCTGCTTGGTCGGCTGGCGGCTGTGGTTGCCAAA

AGCATCCTGCAAGGTAAGAGGATAGTTGTCGTAAGATGTGAGGGGATCAACATTTCTGGGAGCTTCTACAGAAACAAGTG

TAAGTGGTTTATATTTCTGCGTAAGAGAACGAACACCAAGCCTTCCCGCGGACCCTTCCATCTCAGGGCCCCCAGCCGCA

TCTTCTGGAGGACTGTCCGTGGTATGTTGCCGCACAAGACAACCCGAGGTGCACAGGCCTTGGAACGCTTGAAGGTCTTT

GAGGGCATTCCACCGCCTTATGACAAGCAAAAGAGACTGGTTGTGCCATCTGCCCTGAGGGTCATCCGTCTCATGCCCAA

CCGTAGGTTCTGTTCACTTGGCCGTCTGTCCCACGAGGTTGGCTGGAAGTACAAGGAGGTGGTCGACACTCTAGAG---A

TGACGAACGGCCATGGTTATCGCCGCGGTACGCGGTATATGTTCAGCCGCAAATTCAGGGCACGTGGTCCCATCCATCTG

TCCACCTACCTTCATGTCTACAAGCGCGGTGACATCGTGGACGTCAAGGGCACGGGCACCGTGCAGAAGGGCATGCCGTA

CAAGGCCTACCACGGCCGCACCGGGCGCATCTTCAACGTCACAAACCATGCTGTCGGTGTCATCGTCAACAAGAGAGTAC

GCATCATCCCGAAGCGCATCAACCTCCGCGTGGAGCACGTGAAGCACTCCCGCTCCAGGGAAGACTTCCTCAAGCGCGTC

AAGAAGAACGATGATCTGAAGAAGCAGCGCAAAGAAACTGGCGTCTTCAAGAACTTGAGGAGACCCCGCGCTGGCCACAT

GGTTAGCAAGAACAACGTGCCTCAGCTGGTGGAACCCATCCCTTACGAGTTCATCGCT---GCCATGAACGAGGTGGTGA

CCCGGGAATACACCGTCAACCTCCACAAGAGAGTCCATGGCATGGGTTTCAAGAAGCGTGCCCCCCGGGCTGTGAAGGAG

ATCCGTAAGTTTGCTGAGAAGATGATGGGCACCCCAGATGTTCGTATCGACACACGTCTCAACAAGCAAGTCTGGGCCAA

GGGTGTCAGGAGTCCCCCATGCAGGCTGCGGGTTCGTCTGGCCAGGAAGAGGAACGAAGACGAGGACTCGCCCAACAAGT

TATATACCCTGGTCACATATGTCCCTGTCACAACCTTCAAA---ACTTTGGTCACCAAGACCACCGGAACAGAGATCGCA

TCTGAGGGTCTGAAGAACCGGGTGTTTGAGGTTTCCTTGGCTGACCTCCAGAACGACGAGGTGGCTTTCCGCAAGTTCAA

GTTGATTGTGGAGGAGGTCCAGGGCCGTAACTGCCTGACCAACTTCCATGGCATGAACCTCACTACGGACAAGCTCAGGT

CAATGGTCAAGAAGTGGCAGACTCTAATTGAGGCCAATGTTGATGTGAGGACGACGGACGGCTACCTGCTGCGTCTCTTT

TGCATCGGCTTCACGAAGAAGCAGAACCAGATCAAGAAGACTGCTTATGCCCAGCACACCCAGGTCCGTGCCATCCGTAA

GAAGATGGTTGAGATCATGACCAGGGAAGTTTCCAGCAACGACTTGAAGGAGGTGGTGAACAAGCTGATACCAGACAGTG

TCGGCAAGGACATTGAGAAGGCCTGCCAGGGAATCTACCCCCTTCATGACGTGTACATCCGCAAGGTCAAGGTCCTCAAG

AAGCCCAAGTTTGATACAGAGACGGGAGAGAAGGTGGACAGACCGGAGGGGTATGAACCACCAATCCAGGAGGCCGTC--

-GGGCACCCCCTGCAGCTGTGGGAGCCTGCCATGCTCAAGTACAAGCTGGAGGAGCCCATCAGACTGCTGGGTAAGGATC

GCTTCGCCGGCGTGGACATCCGTGTCCGTGTCAAGGGTGGTGGTCACGCGGCGCAGATCTATGCTATCCGTCAGTCCATC

TCCAAGGCTCTTGTTGCCTACTACCAGAAGGTTGACGAGGCCTCCAAGAAGGAGATCAAGGACATCCTGATCAGCTACGA

CAGGACGCTGTTGGTCGCCGATCCTCGCCGCAGAGAGCCCAAGAAGTTCGGTGGTCCCGGTGCCCGTGCACGCTACCAGA

AGTCGTACCGT---AGGTATTCCAACATTGTGTGCAAGAAGGCCGACATCGACCTGTCGAAGCGGGCAGGTGAGCTGAGT

GATGAGGAGCGTCTCATCACCATCATGCAGAACCCTCGCCAGTACAAGATCCCTGACTGGTTCCTCAACAGGCAAAAGGA

CGTCAAGGATGGCAAGTACAGCCAGGTGATGGCCAACTCTCTTGACAACAAGCTGCGTGAGGATCTTGAGCGTCTGAAGA

AGATCCGCGCCCATCGCGGCCTGCGCCACTACTGGGGCCTGCGTGTGCGTGGACAGCACACCAAGACCACCGGCCGAAGG

GGTCGCACTGTGGGTGTGTCCAAGAAG---CTGAAGCTGCCTGAATGGGTGGACCTGGTGAAGACCGCGCCCCACAAGGA

ACTGGCGCCCTACGACCCTGACTGGTTTTACCTCAGAGCATCCACAGCCAGACACTTGTACATGCGTGGGGTAGGCGTCG

GTGCCATGTGTAAGATCTACGGCGGTCGCAAGCGCAGAGGTACCAAGCCAGCCAAGTTCCGCGTCTGCTCCAGGGGCGTG

TCCAGGACGGTCCTACAGTCGCTGGAGGGAATCAAGATGGTTGAGAAGGACGCTGCAGGTGGCCGCAGGTTAACTTCCCA

GGGCCAGAGGGATCTGGACCGTATCGCCGGCCAGGTG

>Helobd

TCACATAGAAAGTTTTCAGCACCGAGACATGGTTCTCTTGGATTTTTGCCCAAAAAGCGTAGTAGACGTCATCGTGGAAA

GGTTAAGGCTTTTCCTAAAGACGACAAGTCTAAACCAGTACATTTAACAGCGTTTTTGGGATACAAGGCCGGGATGACCC

ACGTCGTGCGGGAAGTTGACAGACCTGGATCAAGTGTCCACAAAAAAGAAGTTGTAGAGGCAGTAACAATTTTAGAAACT

CCGCCAATGATTGTTGTTGGTATTGTTGGATATATAGAAACCCCAAAAGGATTGCGAACTTTTAAAACAGTTTGGGCTGA

ACATCTTGGTGATGAATGCAAAAGAAGATTTTATAAAAATTGGTTTAGATCAAAGAAGAAGGCTTTTACTAAAGCTTCAC

AAAAGTGGGCTGATGAAACAGGGAAAAAGGAAATTGATAAGGATTTCAACAAAATTAAGAAGTACTGCACTGTTGTTAGA

GTGCTTGCACACACTCAGATGAAGTTAATGAATAGAAGGCAAAAAAAGGCTCATATTATGGAGATTCAATTAAATGGTGG

TAGCATACCCCAGAAAGTGGACTGGGCTCGTCAACACATGGAAAAAGCGGTGCCTGTCAAACAGGTGTTTGATGTAGATG

AAATGGTTGACGTAATTGGAGTTACAAAAGGCAAAGGATTTAAAGGTGTAACCTCGCGTTGGCATACAAGAAAATTGCAA

AGAAAAACCCATAAAGGCCTTCGTAAAGTGGCCTGTATTGGTGCATGGCATCCTTCTAGAGTATCGTTTTCAGTTGCTAG

GGCT---AGACCGTTTATATCAGTTTATGATGAGAAGGGCGTTGCTTTGCCCATGCCTGCCGTTTTCAGAGCTCCGATCA

GGACAGACATTGTTAGCACTGTTCACATGGATATGCTTAAGAATGGAAGACAGCCATACGCCGTGTCCACAAAAGCTGGT

CATCAGACCTCAGCTGAATCCTGGGGTACTGGTCGTGCTGTAGCTCGTATTCCACGTGTCAGTGGCGGAGGAACGAACAG

ATCTGGCCAGGGTGCTTTTGGCAACATGTGTAGAGGGGGAAGAATGTTTGCTCCCACCAAGACATGGAGAAGATGGCACA

GACACATCAATGTCAATCAGAGGCGCTATGCTATCTGCTCTGCAATTGCTGCGACTGGCATTCCAGCAATTGTCCAATCA

AAAGGTCATCTGATTGACAGTGTTCCTGAAATTCCTCTCGTTCTTAGCGATAAAGTTCAAGAAATTAAAAAGACAAAAGA

AGCTGTTGGTGTATTAAGAAAACTTAAAGCCTGGCCTGATATTGAAAAAGTTAAAAATTCCAACCAAGATAATGGCATAA

CGAGAGCATTCAGAAACATACCAGGTATCACCCTGCTCAGTGTTGACCGGTTGAACCTTTTGAAAATTGCTCCCGGTGGT

CACGTTGGTAGGTTTGTCATCTGGACAGAATCAGCTTTCAAACAATTAGATCCCATCTAT---AGGAGAGAGGGTAAAAC

CGATTATTATGCCAGAAAGCGTTTAGTCGTTCAGGACAAAAACAAGTACAACACCCCCAAGTATCGTTTGATTGTTCGCT

TCACTAACAAAGACATTGTGTGTCAGATAGCGTACGCCAGAATCGAAGGTGATGTTGTGATATGTGCTGCATATTCACAT

GAGTTGCCTAAATATGGTGTCAAGGTCGGGCTGACCAACTATGCGGCTGCTTACTGTACTGGATTACTTATTGGACGCAG

GATCTTGAAAAAGTTCAACTTGGATGGCATCTATCAAGGAACCGAGGAAGTCACTGGTGATGCCTATCAGGTCGAGCAAG

ATGGACAGCCAGGTGCATTCAGGTGTTACTTGGATGTTGGATTGGCACGTACATCGACTGGAGCTCGTGTCTTTGGTGTC

TTG---GTAAATCCGCTGTTTGAAAAGAGGGCCAAGAACTTTTGTCAAGGCCAAGACATTCAACCCAAGAGAGATTTGTC

CAGATTTGTGAGATGGCCCAAGTACATTCGTTTGCAAAGGAAACGTATTTTATACCAACGTCTCAAGGTGCCACCACCAA

TTCATCAGTTCAACCAGGCTCTTGATAGGCAGAATGGTAACCAGTTATTCCGACTGATGGATAAATATAAACCAGAGACA

AAAGTTGAGAAAAAAATAAGACTTGTTGAGATGGCAAAGAACAAGGTGAAAGGAAAGAGTGTTGAGCAGACAAAGAGACC

ACCAGTGATGAGATCAGGCATCAACACAGTCACAGCTCTCGTTGAAAGAAAGAAAACTGATCTGGTTGTTATTGCCAGCG

ATGTTGATCCTATCGAGATAGTTCTATTCCTGCCTGCTTTGTGCAGAAAAATGGGTGTTGCCTATTGCATCGTCAAAAAC

AAATCTCGACTAGGTAGAGTTGTCCACAGGAAGACAGTTACATGTCTTGCATTTCCAACAGTCAACGAAGACAGGTCAGC

ATTGAACAAATTAGTTGAAACTGTGAAGACTGACTTCAATGAAAGAGCCGAAGAAGTGCGTCGCCACTGGGGTGGAGGTG

TTATGGGCCACAAATCCCAAGCCCGT---ATGGGTCGAGTTATTTTCAAGGCCCACACAAAACATCGAAAGGGGGCTGCC

CTTCGTGCCGTCGACTATGCAGAACGCCACGGTTATATAAAAGGAGTTGTCAAGGACATTATTCATGATCCTGGTCGTGG

GGCCCCACTAGCAAAAGTGGTCTTCAGGGATCCATACAGATATCGATTGAGGACTGAAACTTTCTACTGTGGAAAAAAAG

CCACCCTTCAAGTAGGAAACATTCTACCACTGGATTCCCTGCCTGAAGGGACATCAGTTTGCTGTCTGGAGGAGAAGATG

GGAGACAGGGGCAGGTTGGCGCGAACATCTGGAAACTGCGCCGTCGTCATCTCACACAATCCCGAGACGAAAAGGTCCAG

AGTTAAACTGCCATCCGGGGCTAAGAAGTTGTTGCCTTCCAGTAACAGGGCTATGGTTGGTATTGTAGCAGGAGGGGGTC

GCATGGATAAGCCAATGTTGAAGGCAGGCAGGGCCTACCACAAGTACAAGGCCAAGAGAAACTGCTGGCCCAGAGTCAGG

GGTGTGGCCATGAACCCTGTTGAGCATCCCCATGGAGGAGGTAACCATCAGCACATTGGTAAGCCATCAACTGTTGCCAG

A---TCGGGAGATAGACTGACGAGGGCAGCAAAAGTGTTGGAACAGCTCACTGGACAGCAGCCAGTTTTTTCCAAAGCCC

GATTCACGGTAAGATCATTTGGAATAAGAAGAAATGAAAAAATTGCCGTACACTGCACAGTCAGAGGTGCCAAAGCCGAA

GAAATTCTTGATAAAGGATTAAAAGTTAAAGAATATGAATTGAGGAAAGTGAATTTCAGTGAG---ACCTTTAAGAGGTT

CGTTGAGATAGGTAGAGTGGCCTACATTGCGTTCGGTCCTGACGAAGGCAAATTAGTCACCATTGTTGATGTCATCGATC

AGAACAGGGCCTTGGTTGATGGACCATGCACAAGTGTTGTTCGTCAAGCCATGAATTTTAACAAATTAGAACTAACAAAG

TTTGTTATTAAATTCCCACACTCGGCTAGAACTGGCGTTGTTAAGAAAGCTTGGGAGAAGGCTGACATCAACAAGCTCTG

GTCTGAAACATCATGGGCCAAGAAAATTGATCGATTCAAATTATTC---TACAAGTATATGCAGGAGTTGTACCGTAAGA

AGCAGAGCGATGTTCTTCGTTTCTTGCTAAGGATTCGTTGTTGGCAACTTCGTCAACTGGCCGCTGTTCACAGGGCTTCT

AGGCCAACTAGACCAGAAAAAGCTAAACGTCTCGGATACAAAGCTAAACAAGGTTTTGTCATCTACCGAGTCAGAATCCG

TAGAGGTGGTCGCAAGAGACCAGCACCCAAGGGTCAAGTATATGGAAAGCCAAAAAGTGTTGGTATTAATCAGTTAAAAA

ATCAGAGGTCCTTGCAAGCTGTTGCCGAGGAAAGAGTTGGTCGCAAATGCAAAGCTCTCAGAGTTCTGAACAGTTATTGG

ATCGGTCAGGACTCAACCTATAAGTTTTTTGAGGTCATCTGTGTCGATCCATTCCACAATGCCATCAGACGTGACAAGGC

AATTCAATGGATATGCAAACCTGTTGCTAAGCACAGAGAAATGCGTGGTCTCACCTCAGCCAACAGAAAATCGAGAGGTC

TCGGAAAGGGTCACGGATACAGCAAAACTATTGGTGGCTCAAGAAGAGCCAACTGGAAGAAAAACAATACCATTGAAATG

CATAGAAAGAGA---AAATTTTTGGCAAGAAGGACAAATGCCAAATTCAATCAAATTGTCTTCAAGCGTCTGATCATGAG

TCGCACCAACAAGCCACCTCTCTCTCTGTCAAGATTGCGTCACATGAAAAAACAAGGCCGTGAAAATAAGTTGGCTGTAG

TTGTTGGCACTGTTACAGATGACGAGAGATTGTACACTGTTCCGAAGTTGAAGGTAGTTGCACTTCGTGTCACTGACACA

GCCCGTACTAGAATCTTGAAAGCCGGCGGTGAGATCCTTACATTTGATCAATTAGCATTGAAGGCTCCAAAAGGAGAAAA

TACTGTTCTTTTGCAAGGTCAAAGAAAGGCAAGGAAAGCTTACAGACACTTTGGATTGGCTCCTGGAGTGCCACACAGCC

ACACCAGGCCTTAT---AAAGAGTACAAAGTTGTCGGGCGCATGATGCCTACCGACAAGATGAGGACGCCCCCCATCTAC

CAGATGAGAATATTTGCCCCAGATCGTCCCCAAGCGAAATCCAGGTTCTGGTTCTACGCTTCCTATCTTAGAAAAGTCAA

AAGAACACAAGGCGAAATTCTTTTGTGTCAACAAATTTATGAGAAAAGGCCAACTTCAATTAAAAATTTTGGAGTTTGGT

TGCGTTATGATTCTCGAAGTGGAACTCACAACATGTACAGGGAGTACAGAGATTTGACTACAGCTGGTGCAATTACACAG

TGTAGAGACATGGGGGCCCGTCACAGAGCTCGTGCCAGTAGCATCCAGATCATTAAGGTTAATCCTATTCCATCAAGCAA

AGTCAGATCCAAGAACTTGAACCAGTTCTTGGACAGCAGGATCAAGTTCCCTCTGACCCACAGAGTCAACAGGCTCCACC

ATCCTCGCTTCACAACCACGAGACCACACACC---TTGCGGTCGCAAAAGAGACTGGCAGCCGCTGTGTTAAAATGCGGC

AGAAACAAAATCTGGTTGGACCCCAATGAAACGAATGAAATTTCCAATGCCAACTCGCGTCAGAACATTCGTAAACTGAT

CAAGAATGGTTTGATCATCAGAAAGCCTGTTGCTGTGCACTCAAGAGCCAGAGTTCGCAAGAACATGATTGCTCGTAGGA

AGGGCAGACACACTGGAACTGGTAAGAGGAAGGGTACAGCCAACGCTCGTATGCCAGAGAAAGTCATCTGGATCAGAAGA

ATGAGAGTTCTCAGACGTCTTCTCAAAAGATACAGAGATGACAAGAAGATAGACAAACACTTGTACCACAGCCTGTACAT

GAAATGCAAGGGAAACGTCTTCAAAAACAAGAGGGTCCTCATGGAACACATCCACAAGAAGAAGGCTGAGAACTCAAGAG

CCAAGATGCTC---GACCGCTATGTCAAGTTATGGAGAAAACCAAAGGGTATTGACAATAGGGTCAGGCGCCGTTTCAAG

GGCCAGTATTTGATGCCAAACATTGGTTATGGTAGCAACAAGAAGACCAAACACGTTTGTCCTGATGGATTCAAAAAAGT

TCTGATCCACAACATAAAGGAGTTGGAAGTTTTACTGATGCAAAACCGTGTTTTCTCAGCTGAAATTGCCCACAGTGTCT

CGAGCAAGAAACGAAAAGAAATTGTCGAGAGAGCCCAACAACTGGCCATCAAAATTACAAACCCCAACGCTCGACTT---

CAGTTACGAGTTGCAAAAGTGACAGGAGGGGCTGCTTCTAAATTGTCAAAAATGGTCAGAAAATCGATTGCCAGAGTTTT

GACAGTTATTCATCAAACACAGAAAGAAAATTTAAGGAAGTTCTATGCCAAGAAGAAGCGAAAGCCCAAGGATTTGCGTA

AAAAATTGACGAGAGCAATGAGAAGGGCTCTAACCCCTCATGAATTGGGCATCCAGACAGCCAAAGAACTTCACAAAAAA

CTGGCCTACCCCCAGAGAGTTTATGCCATCAAG---TCAAGTCAAATGCAGAAGATCAGAATTGCTCTCAGAGGAGCCGT

TGTCTTGATGGGAAAGAACACAATGATGAGGAAAGCTATCAGAGGCCACATTGAGAACAATCCTGCTCTTGAGAAGTTGC

TGCCACACATCAAAGGCAACATCGGTCTGGTCTTCACCAAAGGAGAACTCACAGATGTCCGTAAGATCATAGAAGAAAAT

AAGGTGGCTGCCCCGGCCAAAGCCGGTGCCTTTGCACCTCTCGATGTAACTGTACCTGCCCAGAACACCTCCCTGGGTCC

TGAGAAGACCTCCTTCTTCCAGGCCCTTGCCATCCAGACCAAGATCTCGAAGGGTACTATTGAAATTTTGAATGATGTCA

AGCTGATCAAGGCTGGTGACAAGGTTGGAGCATCTGAGGCGACCCTCTTGAACATGTTGAATATCTCTCCCTTCACCTAT

GGCCTTCTCATTGAAGTCTACGACAGCGGTACTGTCTTTGATGTATCCATTCTTGATATCACTGATGATGATATCAGGTC

TAAATTCATGGAGGGTGTTGCAAACCTGGCCAGCTTGTCCCTC---ACAAGAGCAGGTCAAAGAACTAGGTTCAAGGCAT

TCGTTGCCATCGGAGATTTCAATGGACACGTTGGTTTGGGGGTGAAATGCTCAAAAGAGGTTGCCACTGCAATCAGAGGA

GCTATCATCCTGGCCAAGTTGTCGATCGTTCCAGTCAGGAGAGGTTACTGGGGTAACAAAATTGGTAAACCCCACACCGT

GCCATGCAAAGTAACTGGTAGTTGTGGTAGTGTTGAGGTGAGGTTGATTCCAGCCCCAAGAGGAACAGGTATCGTCGGTG

CCCCTGTCCCCAAAAAGTTGTTGCATATGGCTGGTATTGACGACTGCTACACTTCTGCTAATGGTCAAACTGCTACTCTT

---TTGGCTGAAGATGGTTACAGTGGTGTTGAGGTGAGAGTTACACCAACTCGTACCGAAATTATCATTCTGGCAACAAG

AACACAGAATGTGTTGGGCGAAAAAGGACGCCGTATTCGGGAATTGACGTCTGTGGTTCAGAAAAGGTTCAATTTCCCAG

ATGGTACAGTTGAACTGTATGCAGAAAAAGTTGCCACGAGAGGTTTGTGTGCCATAGCTCAAGCTGAATCACTGAGGTAC

AAACTCATTGGAGGTCTTGCAGTTAGAAGGGCATGTTACGGTGTTCTGAAGTTTATCATGGAAAGTGGAGCCAAAGGATG

CGAAGTAATTGTTTCTGGCAAACTGAGAGGCCAAAGAGCCAAGTCGATGAAGTTTCTTGACGGACTTATGATCCACAGTG

GTGATCCAATGAATGATTACGTTGATCAAGCTGTCAGACACGTTCTTCTCCGACAAGGTGTCTTGGGCATCAAGGTCAAA

ATTATGTTGCCATGGGATCCTACTGGTAAGCTGGGACCTAAACGACCATTACCTGATCATGTGACCATCCTGGACCCCAA

AGATCAG---GGGCCTCGCAAGCATCTGAAGCGTGTTGTCGCTCCCAAGCATTGGATGTTGGACAAGCTTGGTGGAGTAT

TTGCTCCGAGACCAAGTTGTGGGCCACATAAGTTAAGAGAATGTCTGCCATTAGTCATCTTTTTGAGAAACAGATTAAAG

TATGCATTGACCTATGAAGAGGTTAAAAAAATTCTGAAACAACGACTGGTGAAGGTCGATGGAAAAGTCAGAACTGATAA

AACGTACCCAGCTGGTTTCATGGATGTCATCAGCATTGAAAGGACAAACGAAAACTTCAGGCTGATCTACGATGCCAAGG

GGAGATTTGCTGTTCACAGAATCCAATCTGAGGAAGCCAAATACAAAGTGTGCATGATCACTGGAGGACACAATGTTGGC

GTGTCGGTTGCGGTCACGCACAGGGAACGACATCCTGGCTCGTTTGACATTGTCCACATCAAGGATCCCACCGGACATGT

CTTCGCCACAAGGTTGAACAACGTGTTCATAATTGGCAAGGGCAACAAACCCTGGGTCTCTCTGCCTGCTGGCAAGGGTG

TTAGACTGACCATTGCTGAAGAAAGGGACAGAAGGATTCAAGCC---TTAAATATATCCTGCCCGGCCACTGGCTGCCAA

AAATTGATTGAGGTTGACGATGAAAGGAAACTTAGAGCTTTTTATGACAAGAGATTGACTGCCGAAGTTAATGCAGAATG

CCTCGGAGATGAATGGAAAGGCTATGTTGTTCGCATCTCTGGTGGAAATGATAAGCAAGGATTCCCGATGAAGCAAGGAG

TTTTGACTAATGGAAGAGTTCGTTTGCTCCTTAAAGATGGCCACTCTTGCTACAGAATGAGAAGAAGTGGTGAAAGAAGA

AGAAAGTCTGTTCGTGGGTGCATTGTTGATGCTAATCTCAGTGTTCTCAGCTTGGTCATCATCAAAAAAGGTCTGGGAGA

ACAAGAGGGATTGACTGATACCACAATTCCTCGTCGTCTTGGACCAAAAAGAGCTTCCAAAATCAGAAAGTTATTTAATT

TGGATAAAAAAGATGATGTTAGACAATATGTGGTCAAGAAACCTGTGTTCAGAGCACCAAAAATACAAAGATTGGTGACT

CCACTGGTTCTG---ATTTCACGGGACAAATGGCATAAGCGCAGGAAGACTGGAGGTCGCATGAGTCAAGTGCGCAAGAA

GCGAAAGTTTGAGCTTGGTCGACCACCCGCGCATACAAAAGTAACAATTAGAACTCGAGGTGGAAATAAAAAATATAGAG

CTCTAAGATTAGATACTGGAAACTTTTCTTCTGAAGCTATCACGAGAAAGACCAGAATTCTTGATGTAGTTTACAATGCC

AGCAACAATGAGTTGGTCAGGACAAAAACATTGGTGAAGAACTGCATCATCCTCATTGATTCTGCTCCCTTCAGGAACTG

GTATGAAGGGCATTATGCATTGCCTCTCAGCAGGAAGAAGGGTGTAAAAGCTTGCATATCTTCCAGACCGGGGCAATGTG

GTCGTGCTGATGGTTACATATTGGAAGGTAAAGAACTGGATTTCTACAACAGAAAGATCAAGCCAAAAAAG---AAAACA

TACTCAACTCCTCGTCGTCCTTATGAGAAGGAGCGACTTGATCAGGAGTTGAAACTGATCGGTGAATTTGGATTGAGAAA

CAAGCGTGAAGTATGGAGGGTCAAGTACGCACTGACGAAGATCAGAACTGCCGCCAGAGAGCTGCTTACATTGGATGAGA

AAGATCCAAGGCGTTTGTTTGAAGGAAATGCTTTGTTACGTAGGCTGGTTCGTATTGGTGTATTGGAAGAATCCAAAATG

AAGTTGGATTACGTTTTGGGTCTTAAACTAGAAGATTTTTTGGAGCGTCGTCTTCAAACACAGGTCTTCAAGCTGGGACT

TGCCAAAAGTATTCATCATGCTAGAGTTCTGATTAGACAAAGACACATTAGAGTCAGAAAGCAAGTTGTTAACATCCCTT

CATTTATTGTGCGCTTGGATTCGCAGAAGCACATTGATTTCTCTTTGAAGTCACCATACGGTGGTGGC---TTTCAAAAG

CAACCAACGATTTTTCTCAACAAGAAGAGGTACTACAAAAATGTCGGACTTGGATTTAAAACTCCTCGCGAGGGCCACTA

CATTGACAAGAAGTGTCCATTTACTGGTAATGTTTTCATCAGAGGAAGAATCTTGACAGGTGTCGTCTTGAAGATGAAGA

TGCAAAGGACTATTGTCATCCGAAGAGACTATCTGCATTACGTCAGCAAGTATGAGCGTTTTGAGAAACGTCACAAAAAT

CTTTCTGTCCATTTGAGTCCTTGCTTCAGGGATGTACAAATCGGCGACATAGTTACTGTCGGTGAATGTAGGCCTCTGAG

CAAAACTGTC---GGTATTTCCCAGTCATCGCTTCCATACCGTCGAAGTGTTCCAACATGGTTGAAGTTGAGTTCTGATG

ATGTCAAAGAGCAGATCTACAAATTGGCAAAGAAAGGATTGACCCCATCTCAAATTGGTGTGATTTTGAGAGACTCTCAT

GGAGTTGCCCAAGTAAAATCTGTCACTAGCAACAAAATTCTAAGAATTCTGAAGGCCAAGGGTCTAGCCCCAGAAATTCC

AGAGGATCTGTATTTCCTGATTAAGAAAGCTGTTGCCATCAGAAAACACTTGGAAAGAAATAGAAAGGACAAGGATGCTA

AATTTAGACTTATTTTGGTGGAGAGTAGAATCCATAGATTAGCCAGGTATTACAAGACCAAAAAGGTTCTTCCTCCCACC

TGGAAA---ATGGTGCGCATGAATGTTTTAGCAGATGCCCTAAAATCAATATGCAATGCAGAGAAACGAGGCAAGCGACA

GGTCTTGATCAGGCCTTGCTCCAAAGTTATCATCAAATTCCTAACAGTGATGATGAAACATGGTTACATTGGCGAGTTTG

AAATAATTGATGACCACAGAGCAGGCAAAATAGTTGTTAACTTAAATGGTCGACTAAACAAATGTGGTGTGATAAGTCCC

AGGTTTGACATAAAAATGAAGGATCTTGAACATTGGACAGCTTATCTTATGCCATCTCGACAGTTTGGGTATCTGGTAAT

GACAACATCTGGGGGCATAATGGACCACGAAGAAGCCCGAAGAAAACATTTGGGAGGCAAAATATTGGGATTCTTTTTC-

--GAACCCACGATCCATAGAATCAGGATCACATTAACCAGCAAGAATGTCAAAAGTCTGGAAAAAGTATGTGGTGATCTA

ATCAGAGGAGCTAAAGAAAAGCAGCTAAAGGTGAAAGGACCCGTCAGAATGCCAACAAAAATTCTGCGTATTACAACACG

TAAAACGCCATGTGGTGAAGGTTCAAAGACGTGGGATCGATTTCAAATGAGAATTCACAAGAGGCTCATTGATTTACACA

GTCCTTCTGAGATAGTGAAACAGATAGTATCCATTTCGATTGAACCTGGAGTAGAAGTTGAAGTGACTATTGCTGAT---

ACCGTAAGAACGAGAAAATTTCTGACGAACCGGCTTTTATGCCGCAAACAGATGGTAGTCGATGTGCTCCACCCAGGACG

ATCAACTGTTCCTAAGACTGAAATTAGAGATAAACTGGCTTCGATGTACAAAACAACTTCAGATGTTGTATTTTGTTTTG

GCTTCAGGACCCAATTCCAGGGTGGCAAAACAACTGGTTTTGCATTGATATATGATACTCTTGATGTGGCCAAAAAATTT

GAACCAAAATATAGATTGGCGCGA---GGTAAGCCTCGCGGTCTTAGGACAGCTCGTAAGTTGAAGAATCACAGACGTGA

GCAGAGATGGAATGATAAAGATTATAAAAAATCTCACCTCGGTACAAGATGGAATCCGTTCGGTGGGGCCTCCCATGCTA

AGGGAATCGTTCTTGAAAAAGTGGGTGTTGAAGCCAAACAGCCCAACTCTGCCATCAGGAAATGTGTGAGAGTTCAGTTG

ATCAAAAATGGAAAGAAGATTACCGCCTTTGTGCCATGGGATGGTTGCTTAAACTTCATTGAAGAAAACGATGAAGTTCT

GGTAGCTGGATTCGGTCGTAAAGGTCACGCCGTGGGAGATATTCCTGGAGTTCGTTTCAAGGTCGTCAAAGTTGCCAGCG

TCTCCTTGTTGGCCCTCTACAAAGGCAAGAAGGAACGACCCAGATCAATG---AGGCAGGCTCGCCAACATGGGAACTTT

TACGTCCCACCACAGCCAAAACTTGCCTTTGTTATCAGAATCAGAGGGATCAATGGACTGCACCCAAAGCCAAGGAAAAT

ATTACAGTTGTTTCGTTTGAGGCAGATCAACAATGGAGTTTTCATCAAGTTGAACAAAGCTACCATCAACATGTTAAGGA

TCGTTGAGCCATATGTCACTTGGGGGTACCCAAACTTGAAAAGTGTCAAGGAGTTGATCTACAAAAGAGGATTTGGCAAA

GTTGATAGGATCCCCCTAACTTCCAACATCATTGAAAAGAAATTAATGATTTGTATGGAGGATTTGATCCATGAGATCTT

CACCGTCGGTCCCAACTTCAAGTTTGCCTCCAACTTCCTGTGGCACTTCAAACTGAACACACCAAACGGTGGATGGCGTA

AGAAGACAAACCATTTTGTTGATGGTGGTGATTTCGGGTGCAGA---CAGGAAAAGAAGAGGAAGTTCACTGAGACAGTC

GAGTTGCAGATTTCGTTGAAGAACTATGACCCTCAGAAAGATAAACGTTTCTCCGGCACTGTAAAGTTGAGGAATATTCC

AAGGCCAAAGATGGCGATCTGCGTTCTTGGAGATCAGCAGCATTGTGATGAGGCAAAGGCTAACAGCGTTGAATGCATGG

ACGCTGAAGCGCTCAAGAAATTGAACAAAAACAAGAAACTCATTAAGAAATTAGGTAAAAAGTATGATGCTTTCCTGGCA

TCTGATTCCCTGATCAAACAGATCCCTCGTATTGTGGGCCCGGGATTGAACAAAGCTGGTAAGTTTCCTTCCATGCTGAC

CCATGGTGAGTCGATGACAGGCAAAATCGAAGAGGTCAAGGCCACAATAAAGTTTCAAATGAAGAAGGTATTGTGTCTGT

CAGTAGCAGTAGGAAATGTGAACATGACCCAAGATGAACTGGTCTCTAACATCACCATGGCCATCAATTTCTTGGTCTCT

CTCCTCAAGAAAAACTGGCAAAATGTCAGGTCGCTATACATAAAATCAACCATGGGACCCGTTCAGAGATTGTAC---GG

GCATTTCCACAAAGATTGGAAGAAACACGTGAGGACATGGTTTAACCAGCCGGCTAGGAAGTTTCGCCGCTGGACGAAAC

GTAATGATAAAGCAAAAAAGATTGCTCCCAGGCCTGTCGCTGGTTTGAGACCCGTAGTTCGCTGCCCTACCTTCAAATAT

AACTCCAGAATTAGAGCTGGACGAGGATTCACTATTGAAGAGTTGAAAGCTGGTTTCAACAAAAAATATGCCAAAACCAT

TGGCATTGCAGTTGATTTCAGACGCCGCAACAAGTCAGTTGAAGGTTTTCAAGTGAATGTTCAGAGATTGAAGGAGTACA

AGGCCAACTTGATCCTCTTCAAGAAATTGAACAAACCT---ATGACTAGGTATTCGTTAAATCCGGAGAATGCGGCAAAA

GCTGCCAAAGCCAGGGGTTCTCATCTTAGAGTTCACTTTAAGAACACAAGAGAAACAGCCCAAAACATTAAACACATGCA

CCTTAGAAGAGCTGTTGCCTATTTAAAAAATGTCATTGATCATAAAGAATGTGTTCCATTTAGGAGATTCACTGGTGGAG

TTGGACGATGTGCTCAGGCAAAAGCCTGGGGAACAACACAAGGTCGTTGGCCCAAGAAGAGTGCAGAATTTTTGTTGCAA

ATGTTGAAAAATGCAGAAAGCAATGCTGATTTTAAGGGCTTAGATACTGACCATTTGGTGATTGAGCATATCCAAGTCAA

CAGGGCCCCAAAAATGAGAAGGAGGACATACAGAGCACACGGAAGAATTAATTACATGAGCAGTCCCTGCCACATTGAAA

TCATATTG---ATGAAGTTCAACAAGAGTGTGTCTGGTTCACGTCGCAAGAATCGTCGGCGACATTTCAATGCTCCTTCG

CATATTAGAAGGAAGATCATGAGCTCGCCATTGTGCAAAGAGCTTAGACAAAAATACAACGTACGCAGTATCCCCATCCG

CAAAGATGACGAAGTTCAAGTGTGTCATTTGTTTTTTCACAATTCTCAAGGCAAGGTTGTCCAAGTATACAGAAAGAAGT

ACATCATACACATTGAAAGAATCCAAAGGGAAAAAGTCAACGGTGCGACTGTCCATGTCGGCATTCACCCATCAAAAGTT

GTCATTGTCAAACTCAAAATGGACAAAGATCGTAAGTTAATTTTGGAACGCAAGGCCAGATCCAAGGGCAAG---TCAGG

CAAAGTTGTGCTCATACTAGGAGGAAAATATGCTGGACGGAAAGCAATAATCGTCAAGAATTATGATGATGGCACCTCTG

AGAAACAATACAGTCATGCTCTCGTCGCTGGGATCAGCAGATACCCTCTGGCTGTCACCAAAAAGATGAACAAAAAGAAG

ATCAAAACAAGGTCCAAGATTAAGCCATTCATTAGAGTCTACAACTATAATCACTTGATGCCAACTCGGTATACAGTTGA

GGTGAGGTATAGAACAGGCAAGAACAGATGGTTCTTCCAGAAGTTGAGGTTC---ATGAGGGGCCATGTCAGCCACGGCC

ATGGGCGAATTGGCAAACACAGGAAGCATCCCGGTGGTAGAGGTAATGCTGGTGGTCAACATCATCATCGTATCAACTTT

GATAAATACCATCCTGGTTACTTTGGAAAGGTTGGCATGAGGCATTTTCATTTGACCAAACAGAAATATTTCTGTCCAAC

AATAAATGTTGATAAGCTGTGGAGCTTGGTTTCAGAACAGACTCGTGAAGTTGCTCCAGTAATTGATGTCGTCAGAGCCG

TAAGTTACAAAGTGTTGGGCAAAGGCCACCTACCTAAACAGCCAGTTATTGTGAAGGCTAAATTTTTTAGCAAGACAGCT

GAAGACAGAATTAAAAGTGTTGGTGGTGCTTGCGTGTTGGTTGCA---ATGGCCGCGCACAAGACTTTTCGAATTAAAAT

GAAATTGGCAAAAAAGCAAAAGCAGAACAGGCCTGTCCCCCAGTGGGTTAGGATGAAAACCGGCAACACGATTCGTTACA

ATTCGAAGAGGAGACATTGGAAGAGGACCAAGCTAAAGTTG---ATGGCAAGAACCAAGCAGACGGCTCGCAAGTCAACC

GGTGGCAAGGCACCTCGCAAGCAGCTGGCCACGAAGGCTGCCAGAAAAAGCGCTCCGGCTACCGGAGGTGTCAAAAAACC

TCATCGTTACAGGCCTGGTACAGTAGCTCTCAGAGAAATCAGGCGCTACCAGAAAAGCACCGAGCTGCTCATCAGAAAAC

TGCCCTTTCAAAGACTCGTTCGTGAGATAGCTCAGGATTTCAAGACCGACCTTCGCTTCCAGAGCTCGGCAGTCATGGCT

CTGCAGGAAGCAAGCGAAGCTTACCTGGTCGGCCTCTTCGAAGACACCAACCTCTGCGCCATTCACGCCAAACGCGTCAC

CATCATGCCCAAGGACATCCAGCTCGCCCGCAGAATCAGAGGCGAACGTGCT---CTTGCATGTACGTACGGCGCATTGA

TCCTGGCCGACGATGATATCACCATCACGGCAGAAAAGATCAGTACATTGTTGAAGGCTGCAGATGTCGATGTTGAACCA

ATTTGGCCT---ATGAGATACGTAAGCGCGTACTTGCTGGCTCGTCTGGGTGGTAATGAAAATCCGAAAGAAGTTGATAT

CAAAAAGATTTTAAGCAGCGTTGGAATTGATTCAGATGATGCCTTGGTCAAGAAGGTCGTCAGTGAACTATCCGGCAAAA

ACATCGAAGAGTTGATCGCTGCTGGT---GACAAAGAATGGGTACCTGTCACTAAGTTGGGTCGTTTAGTCAAAGAAATG

AAAATCAAAACGATTGAAGAAATTTATCTTTTCTCTCTTCCAATCAAGGAGTTTGAAATCATTGATTTCTTGAGCAGTTT

GAAAGATGAAGTATTGAAAATCATGCCAGTGCAAAAACAG---AAATATTTGCCGCACTCAGCTGGTCGGTACCAAGCCA

AACGATTCCGCAAAGCCCAATGTCCCATCGTTGAGAGGTTGACTGACAGCTTGATGATGCACGGAAGGAACAATGGCAAG

AAGCTGATGGCCATGAGGATTGTGAAGCATGCTTTTGAAATCATCCACTTGCTCACTGGAGAGAATCCTCTACAAATTTT

GGTGAATGCCATCATCAACAGTGGCCCTAGAGAAGATTCAACACGTATCGGTAGAGCTGGGACTGGTGCAAGAGAGGCTT

CATTCAGAAACATCAAGACCATCTCTGAGTGCCTGGCTGACGAACTCATCAATGCTGGAAGGGGCTCATCCAACTCATAT

GCCATCAAGAAGAAAGATGAA---AACGCGAAAATAGTTAAATTACCGGGTGAAAAACCCGATGAGTTAGAAAGCTCTAT

TTCTCAGGCGTTGTTGGAACTGGAGATGAATAGTGATATGAAAACTCAACTGCGGGAACTCACATTCACAGGAGCTAAAG

AAGTAGATGTGCATAACAAAAAGGCAATCATTATTTCTGTTCCTGTTCCTCAATTGAGATCCTACCAGAAAGTTCAATCG

AGATTGGTTCGTGAACTAGAGAAGAAGTTTTCTGGCAAACATGTTGTTTTCATTGCCCAGAGACGAATCCTCCCAAAACC

AACAAGAAAAACTCGAAAGAATAAGCAAAAAAGACCAAGAAGCCGTACCCTAACAGCAGTGCATAATGCTATGCTGGAGG

ATCTCTGCTTTCCTTCTGAAATTGTTGGTAAAAGAATAAGAATCAAGACAGATGGGAGTCAAATAATCAAGGTTCATTTG

GACAAAAGCCAACAGACCAATGTTGAACACAAGGTTGATACATTTACAGCTGTGTACAAGAAACTAACTGGTAAAGATGT

AGTTTTTGAATTTCCAGAATACGTTCTG---ATGCCAAAAAAGAACCGTGTTCTCATCTACGAACATTTGTTCAAAGAAG

GAGTTCTGGTGGCCAAGAAAGATTTTCACGCTCCGAAGCACACAGAGATCGACGTACCCAACCTCCATGTCATCAAGGCC

TTGCAGACGTTGGTTTCTAAAGGTTTTGTAACTGAGCAGTTCTGCTGGCGCCATTATTATTGGTATTTGAAGAATGAAGG

AATTACATATCTCCGTGATTTCTTGCACCTTCCTCCAGAGATTGTGCCATCTACATTGAAG---CACGTTACCGATCTTT

CTGGAAAGGAAACGATTGTGCGAGTAACTGGTGGCATGAAGGTCAAGGCAGACAGAGATGAGTCGTCTCCATACGCCGCC

ATGTTGGCTGCTCAAGATGTCGCTGAGAGATGCAAGCAGATAGGGATTACTGCTCTTCACATTAAATTGAGAGCTACTGG

AGGAAATAGAACAAAGACGCCAGGACCAGGTGCTCAGTCAGCCCTCAGGGCATTGGCCAGATCAGGGATGAAAATTGGAA

GGATTGAGGATGTC---AAAAAAAAGAAACGAACTTTTCGCAAATACACATATAGAGGTGTTGATTTGGATCAGTTACTG

GATATGAACAATTCTCAACTGATGCAGCTGTTTTCATGTAGAGTGAGAAGGAGGTTCCAAAGGGGAATCAAAAGAAAACA

GATGGCTTTTCTTAAGCGATTACGTAAGGCTAAAAAAGAAGCCCCAGCTCTTGAAAAACCTGAGTGCATCAAAACTCATT

TGCGAAACATGGTCATTACCCCTGAGATGATTGGGAGCATTGTAGGCATCTACAATGGAAAAACATTCAATCAAGTTGAA

ATTAAGCCTGAAATGATTGGTCATTATCTTGGCGAGTTCAGCATTACGTACAAGCCTGTGAAACATGGTCGTCCAGGAAT

TGGAGCCACACACTCATCTAGATTTATTCCATTGAAA---ATAATTGAGAAATATTATCCTCGCCTTACTTTGGATTTTC

ATACGAATAAAAGGCTATGTGAAGAAATTGCCATTATTCCAACAAAGAAGTTGAGGAATAAGATTGCTGGGTTTGTCACA

CATTTGATGAGGCGCATCCAAAAGGGGCCAGTCAGAGGTATCTCCATAAAATTGCAAGAAGAAGAGAGGGAAAGAAGAGA

TAACTATGTACCAGAGGTCAGCGCCATTGATGAGGATATCATTGAAATCTGCCCAGACACCAAAGAGATGTTGAAATTA-

--CCAAGAAAATGCTCTGCTAGCAACAGAATAATTTCTGCCAAAGATCATGCCGCCATTCAAATCAACCTTGCTGAGGTT

GATGAGCAAACTGGAAGAATGACTGGTCAGAACAAGACATATGCCATATGTGGAAACATCAGGAGAATGGGAGAATCAGA

TGATTGCCTCAACTTCTTATGTAAAAGAGATTCGATCCTGCCAAAG---CACGTCAACCCAGTGAGGTGCACGAACTGCG

CCAGGTGCGTTCCTAAAGATAAAGCCATCAAGAAGTTTGTCATCAGAAACATCGTCGAAGCTGCTGCCGTACGAGATTTG

ACTGAGGCCTCTGTTTACCAAAGTTGGTCTCTTCCCAAACTGTATGCCAAGCTACATTACTGTGTCAGCTGCGCAATTCA

CAGCAAGGTTGTTCGTAATAGGTCAAGGGAAGCCAGGAAAGATCGTACCCCACCACCAAGATTCAGGCCT---ATGATCT

TTGTAAAGACGTTGACGGGCAAAACCATCACGCTCGAGGTTGTGAAAGCTAAGATCCAAGAAGGTATTCCTCCTGATCAA

CAAAGACTCATCTTTGCCGGAAAACAGTTGGAAGATGGTGACATACAAAAAGAGTCAACCCTCCATCTTGGCGGAGCCAA

GAAGCGCAAGAAGAAGAATTACACCACACCGAAAAAGAACAAGCACAAGAAGAAGAAGGTGAAGCTGTCACTTCTCAAGT

TCTACAAGGTCGATGATAATGGCAAAATCAGCAGACTGAGACGTGAATGCCCGAACGAGGAATGTGGTGCCGGTGTATTC

ATGGCTAACCATTTTGACAGACAGTACTGCGGTAAATGCTGTCTCACGTATGTATTCAACAAA---AAGCCTTACCCCAA

GTCAAGGTTTTGCAGGGGTGTGCCAGATCCAAAGATTAGGATCTTTGATCTTGGGAGGAAGAAGGCCAAAGTGGATGAGT

TTCCACTGTGTGTGCACCTGGTGTCAGATGAATGGGAGCAAATTTCTTCGGAGGCCCTCGAAGCCGGAAGAATTTGTGCT

AATAAATATTTAGTGAAACACTGTGGTAAGGATGCCTTTCATCTGAGAGTTAGACTTCACCCCTTCCACGTCATCAGAAT

CAATAAAATGTTATCATGCGCCGGGGCAGATAGGCTCCAAACTGGGATGAGAGGTGCTTTTGGCAAGCCTCAAGGGACAG

TAGCACGTGTGGACATCGGACAGATTATCCTGTCTGTTCGTGCAAAGGATGCACACAAAGATAAGGTCATTGAGGCTCTG

AGGAGGGCAAAGTTCAAGTTTCCTGGCAGGCAAAAGATCCACGTGAGCAGGAAATGGGGTTTCACA---AAAAGAACCAA

AAAGGTCGGTATTTGCGGTAAATATGGTACAAGATATGGTGCCAGTTTGCGTAAAGATATCAAAAAAATGGAAATAACCC

AACATGGAAAGTACACTTGTAGTTTCTGTGGAAAGGAAACCATGAAGAGGAAAGCTGTTGGCATTTGGACGTGTAAATCC

TGTAAAAAGGTGGTTGCCGGTGGAGCTTATATCTACAGTACAACTGCTGCTGCCACAGCCCGTTCCACCATACGTCGTCT

CAAGGAAAGT---CACATTCAAAAACAAACTTGCGCCAGATGTGGGTACCCCGCCAAACGCATGCGAAAATATAATTGGA

GTGAAAAAGCTTGCAGACGCCGAACTCAAGGCATTGGTAGAATGAAACACCTCAAGAAAGTTTTCAGAAGATTCAGGAAT

GGCATCAGGGAAGGAGGAGTT---AAGGGAAAAGATTCATTGTTTGCTCAAGGTAAAAGGCGTTACGATCGAAAACAGTC

AGGATATGGTGGTCAAACAAAACCTATTTTCCACAAAAAGGCTAAAACAACAAAGAAGATAGTGTTGAAGATGGAATGCA

CTGAATGCAAGTACAGAAAACAACTTGCGATCAAGAGGTGCAAACATTTTGAATTGGGTGGTGACAAGAAGAAAAAG---

CAAACGAAGCATGCCAAATTTGTCCGTGATCTCGTTAGAGATGTCTGTGGATTTGCACCATACGAGAAACGTTCAATGGA

GTTGTTAAAGGTTGGAAAGGACAAAAGGGCACTCAAATTCTGCAAAAGAAGGGTGGGTACCCATTTGAGAGGCAAGCGAA

AGAGGGAGGAGATGTCGACTGTCATTCAGAAGATGAGGAAGGCCCAT---AAGGCGATCGAGAGCATCAACTCTCGCTTG

GCTCTCGTCATGAAAAGTGGTAAATACTTTTTAGGCTACCAGCAGACGTTGAAAACTTTGAGGCAGGGAAAGGCGAAATT

GGTAATCATTGCCAACAACACCCCGCCTCTCAGGAAGAGTGAGATCGAGTACTATGCCATGTTGGCCAAAACAGGGGTCC

ACCATTACAATGGCAACAACATTGAGTTAGGGACTGCTTGCGGAAAATACTTCAGAGTTACAACACTCAGTATTACTGAT

CCAGGT---TGGAGAATGAGATCCTTCTCTCAACATAAACGTTACTTGAGAAGTTCGATTACTCCAGGAACTGTTCTCAT

CATGGTTGCTGGTCGTCATAAGGGAAAAAGAGTTGTGTTCTTGAAGCAGCTTAAATCTGGATTGCTCTTAGTTACTGGAC

CATACCATTTAAATGGGTGCCCCTTGAGAAGAATCAACCAGATTTATGTAATCGCCACAAAAACCAAGTTGGATATTAGT

GCAGTTAAAATTCCTGAGCGATTAACTGATGATTATTTCAAGAGGAAGACAACTGAGGGTGAAATTTTTGACACCAAGAA

GGAGTATGCGGTGACAGAGGAAAGAAAGAAGGATCAGGTTGATGTCGATGGGCAGATCTTA---GTAATAGTCAAGGGAC

CCCGTGGCACCCTAAGACGAAATTTCACTCACATGGATATCAGGAAGGTTAAGACAGTTTGTTCCCACATCGAGAACATG

ATCAAAGGAGTTACTGGATTCAGATACAAGATGAGGTCTGTCTATGCTCATTTCCCCATCAACGTTTCCATTTCTGAAAA

CAACTCCCTCGTCGAGATTAGAAACTTTTTAGGTGAAAAGATCAACAGGCAAGTTAAAATGTTGCCTGGAGTTACCTGTG

TAGCCTCAAAAGACATGAAAGATGAGTTTATACTCGATGGTAACGACATAGAATTGGTTTCCAGATCAGCTGCCCTGATC

CAACAGTCGACAACGGTT---GTATGTTTAAGGGCCGTCGGTGGTGAAGTTGGTGCCACATCAGCTCTTGCCCCAAAAAT

TGGTCCCTTGGGTCTTAGTCCAAAAAAGGTTGGTGAAGATATTGCAAAGGCTTCCCAAGATTGGAAGGGCTTGAGGGTGA

CTGTCCAGCTGACAATTCAGAATCGTCAGGCCCAAGTTACTGTCATACCTTCTGCTTCTTCTCTCATCATTAAGGCCCTG

AAGGAACCCCCAAGAGATAGAAAGAAAGTGAAAAATGTCAAGCACAGTGGAAACTTGACAATCGAGGAGATTATTGGCAT

TGCCAGGATCATGAAAGCAAGAAGTATGGCTAAAAATTTGGCTGGAACTGTCAAAGAAGTTCTAGGTACCTGCCAGTCTG

TTGGATGCACAGTCGAAGGCAGCCATCCT---GATGCCAGAGGTCATCTTCTGGGTCGACTGGCTGCCACCACGGCCAAA

TCAATTCTGCAAGGTAAACGTATAGTTGTTCTAAGATGCGAGGGCATAAACATTTCTGGAAATTTTTACAGGAACAAGGT

GAAGTATTTAGATTTTTTGAAAAAAAGAATGAATACCAATCCAAGCCGAGGTCCTTACCATCATAGAGCTCCCAGCAAAA

TATTTTGGAGAACTGTTAGAGGTATGTTGCCACACAAGTTACATCGTGGTAAGGAGGCTTTGGATCGTCTGAAAGCATTT

GAGGGAGTACCTCCACCTTATGACAAACAAAAAAGATTTGTTGTTCCTTCTGCTCTTCGCATAATTAGATTGAAGCCACG

AAGAAAGTTTTGCCAGTTAAGCAGGCTGTCGCATGAAGTTGGTTGGAAGTATCAAGGTGTTGTAGCAACTCTTGAA---A

TGACAAACTCCGGAGGTTATAGAAGAGGAACGAGACACTTGTTCTCTCGCAAGTTCAGGAAGAGGGGAGTCATACCCATC

TCTACATACCTCAAGACATACAAAAGAGGTGACATGGTAGATATCAAGGGTAATGGTGCAGTTCAGAAGGGTATGCCTCA

CAAATCGTACCATGGAAGGACAGGTGTGGTGTACAATGTCACCCCACATGCTGTTGGTGTCATTGTCAACAAGAGAGTCA

GAATCCTTCCTAAGAGAGTAAACATCAGAGTGGAGCACATCAAACATTCAGCTTGCAGGCAGGAGTTTCTCAAGAGGGTA

GCTGAGAATGAGAGGAAGAAGAGAGAGGCCAAGGAGAGCAAGACTGTTGTCTTCTGCAAGAGACCAAGACCAGGTCATTT

TGTATCAAAATATAACAAGCCCATCATACTGGAGCCAATCCCATACGAGTTCCTCGCT---GTAGCCAACGAAGTGGTTA

CGAGGGAATACACCATCAACATTCACAAAAGAATTCATGGGATGGGCTTCAAAAAGCGTGCCCCCAGAGCTATCAAAGAA

ATCAAGAAATTTGCTCAAAAGCAAATGGGGACAATGGACGTGAGAATTGAAACTCGCTTGAACAAGCATATGTGGTCCAA

AGGCATCAGGAATGTGCCTTACAGAGTAAGAGTAAGACTTGCAAGAAAGAGAAACGAAGATGAGGACTCACCTCACAAGT

TGTACACACTCGTTTCTTATGTTCCCATGGTCAACTTTAAA---ACCATTGTTTCTAGAACCGTCGGAACCGAAATTGCT

TCTGATGGCTTGAAGGGACGTGTTTTTGAAGTCTCGTTGGCTGATTTGCAGAACGACGAGATTACCTTCCGTAAGTTCAA

ATTGATGTCCGAGGAAGTTCAAGGTCGTCACGTTCTCACCAACTTCCATGGCATGGACATGACCACTGACAAGCTGAGGT

CCATGGTCAAAAAGTGGCAGACCCTGATCGAAGCACATGTTGATGTGAAGACAACTGATGGCTATCTCCTGAGACTCTTC

TGCATCGGATTCACCACAAAGCAGAACCAGCTGAAGAAGACCTCGTATGCACAGAGCACCCAAGTTAGGGCAATTAGGAA

GAAGATGGTTGAGGTGATGTTGAGAGAAGTGGTCGCCAGTGACTTGAAGGATGTTGTTAACAAATTGATCCCAGACAGCA

TTGGAAAGGACATTGAGAAATACTGCCAGGGTATCTACCCACTCCACGATGTCTACGTCAGGAAAGTTAAAATCCTCAAG

AAGCCAAAGTTTGATACTGAGGGTGGCGAGAGAATTGACAGACCAGGCAACTACGAACCTCCTGTTGTGGACAGTGTC--

-GGACGACCGATCGATCTGCTTGAGCCAGAGCTCCTTCGATACAAGGTCCAGGAACCATTGCTGTTGTTAGGCAAGGAAC

GTTTTTCAGCTGTCGACATCAGAGTCAGAGTGTCCGGGGGTGGTCGAGTTTCTCAAATTTATGGTATCCGTCAGGCAATT

TCAAAAGCTCTGGTTGCTTACTACCAGAAAGTTGATGAAGCATCAAAGAAAGAAATTAAAGATATCCTAATCAGTTATGA

TAGGACTCTTCTGGTGGCTGACCCGAGAAGATGCGAGCCCAAAAAGTTTGGTGGTCCCGGAGCAAGAGCCCGTTACCAAA

AATCATACAGA---CGTTATGCTCACGTTGTCTGCAAGAAAGCCAACGTCGATGTAACAAAAAGAGCAGGAGAACTTACT

GAGGAAGAGAAGTTGGTGACGATAATGGGCAACCCTCGCCAGTACAAAATCCCGGATTGGTTTTTGAATAGACAAAAGGA

CATTAAAGATGGCAAATACTCGCAGGCCATGTCCAACATTTTGGACAGTAAGATTCGTGAGGATCTTGAAAGATTAAAGA

AGATAAGAGCACACAGAGGATTGAGACATTTCTGGGGGTTGAGGGTGAGAGGACAGCACACAAAGACCACAGGAAGAAGA

GGAAGGACCGTTGGTGTGTCCAAAAAG---TTAAAGGTTCCAGACTGGACAGATGTTGTCAAGTTGGGTGTGCACAAGGA

ACTGGCTCCATATGATGAAGATTGGTTCTACACCAGAGCCTCGATCGCTCGCCACATGTACATCAGACCTGTTGGAGTTG

GAGGACTAACAAAAATTTATGGAGGTCGCAAAAACAATGGCACTCGTCCCTCGCATTTTGTCAGGGGGTCCAGTTCTGTT

GCCAGGAAAGCTGTGCAAGCTTTGGAAGGTCTCAAATGGGTTGAAAAGGATGCCAATGGGGGCCGCAGGCTAACAGCAGC

AGGAAGGAGAGATTTGGACCGCATAGCTGTCAAGTTG

>crasso

TCCCATCGGAAATTTTCGGCACCTCGCCACGGATCTAAGGCGTTCCTCCCCAAGAAGCGCTCCAGGCGCCATAGGGGAAA

ATGCAAGTCTTTCCCGAAGGATGATAAATCTAAACCCTGTCATTTAACAGCATTTTTGGGCTACAAAGCTGGGATGACCC

ATATTGTGCGGGAAGTTGATAGACCTGGATCTAAGTCCAACAAGAAGGAGATTGTCGAGGCTGTCACTATTCTGGAGACT

CCGCCCATGATGATCGTTGGGGTTGTCGGATACATTGAAACCCCCAAGGGACTGAGAGCTTTCAAAACCGTCTTTGCCGA

GCATCTGAGTGAAGACTGTAGACGCCGTTACTACAAGAACTGGTACCGTGCCAAGAAGAAGGCCTTCACCAAGTATTCCA

AGAAGTGGCAGGACGAAGCTGGCAAGAAGGAGATCGAGAGGGACTTTGCCAAGATGAAGAAGTACTGCAAAGTCATCCGT

GTGATTGTCCACACTCAGATGAAGTTGCTGAAGAAACGCCAGAAGAAGGCTCACATCATGGAGGTCCAAGTCAATGGTGG

CACAGTCAGCCAGAAGGTGGACTGGGCCAAGGAAAAGCTGGAGAAGGCTGTCAACATTGATCAGGTGTTCTCCCAGGATG

AGAACATTGACTGCATCGGAGTCACTCGAGGAAGAGGCTTCAAAGGTGTGACCTCCAGGAGGCACTGTAAGAAGTTGCCC

AGGAAGACCCACAAGGGTCTGCGTAAGGTGGCCTGTATTGGAGCCTGGCATCCCAGTCGAGTTCAGTACAGCGTGGCCCG

TGCT---AGGCCACTTATAAGTGTGTATGGGGACAAGGGGGAGAATGTTACACTGCCTGCAGTGTTTCGGGCGCCCATCA

GGCCAGACATAGTTGTGTTTGTCCATTCTCAGATGAGGAAGAACTCCAGACAGCCATATGCTGTCAGCCAAAAAGCAGGT

CACCAGACTTCAGCTGAGTCCTGGGGTACAGGAAGAGCTGTGGCCCGTATCCCCCGTGTAAGGGGAGGTGGTACCCACAG

GTCAGGCCAGGGAGCTTTCGGAAACATGTGTCGAGGTGGTAGGATGTTCGCCCCCACCAGAACGTGGAGACGCTGGCACA

GAAAGATCAATGTCAACCAGAAGAGATATGCCATCTGCTCTGCTATTGCTGCCACTGGAATCCCTGCTATTGTCATGTCA

AAAGGTCACAGAATTGAAGAAATTCCAGAGGTCCCCTTGGTGGTCAGTGACAAAGTGGAGGAGTTCAAAAAGACCAAGGA

AGCTGTAGCCCTCCTGAAGCGCGTGAAGGCCTGGGGAGATATCCAGAAGGGTTACAGATCTAACTCAGACAATGGCATTT

GTAGAGCATTCAGAAATATTCCAGGTATCACACTCATCAATGTATCTCGTCTGAATCTTCTGAAGATCGCTCCTGGTGGT

CATGTAGGAAGATTTTGTATCTGGACAGAGTCTGCCTTCCGCAAATTGGACAAAATATAT---AGGAGAGAGGGCAAGAC

AGATTTTTTTGCAAGAAAACGCCTTGTAATTCAAGAGAAAAATAAGTACAACACTCCAAAGTATCGAATGATTGTTAGAT

TTACCAACAAAGACATCATTTGTCAGATAGCGTACGCTCGTATTGAGGGGGATATTATTATCTGTGCTGCTTATTCCCAT

GAGCTGCCTCGATACGGTGTCAAGGTCGGGCTTACTAACTATGCAGCTGCCTACTGTACTGGACTCCTGTTAGCCAGAAG

ACTTCTACAAAAACTGAAGTTGGATGACATCTATGAGGGACAGAAGGAAGTTGACGGAGAACAGTTCAATGTTGAGGTAG

ATGAAAAACCAGGTGCATTCAGGTGCTACTTGGACGTTGGTCTCGTGCGCACAACCACAGGTGCTCGTGTGTTTGGAGCC

ATG---GTTAACCCCCTGCTTGAAAAAAGACCACGAAACTATGGAATAGGTCAAGATATTCAGCCTAAGCGTGACTTGAG

TCGCTTTGTAAGATGGCCCAAATACATCCGTCTCCAGCGCCGAGCTGTGTTGATGCAGAGACTGAAGGTTCCCCCGCCAA

TCAACCAATTCAGTCAAACTCTTGACAGACAGACAGCTACTCAGCTGTTCCGTCTGATGGACAAGTACAAGCCTGAGTCT

AAGCAACAGAAGAAGGCTCGTCTAAAGGCCAGAGCTGAGGAACGAGCTGCTGGTAAGGAGGACAAGCCTACCAAACGACC

CCCTGTCATGAGGTCTGGAGTCAACACAGTGACTGCCCTTGTGGAACAGAAAAAGGCACAGCTGGTTGTCATTGCACATG

ATGTTGATCCTATTGAAGTCGTCATTTTCTTACCAGCATTGTGTAGGAAGATGGGAGTCCCCTACTGCATCGTGAAAGGA

AAAGCAAGACTAGGACGTGTCGTTTACAGGAAAACTGCCACATGTCTGGCCTTCACAAATGTCAATGAGGACAAAAATGC

ATTGAGTAAACTCTGTGAAGCTGTCAAGACCAACTTCAATGACAGATATGACGAGATTCGTCGACACTGGGGAGGTGGAC

AGATGGGATCTAAATCTCAAGCTCGC---ATGGGCCGAGTGATCTTCAAGGCCCGCACCAAGAACAGGAAGGGCGCAGCC

CTCAGGGCTGTGGACTTTGCCGAGAGACATGGATATGTGAAGGGAGTAGTCAAGGACATTATTCACGATCCAGGTCGTGG

TGCTCCTCTGGCAAAGGTTGTGTTCCGTGATCCATACAGGTTCCGCCTGCGCACGGAAACTTTCTACTGTGGAAAGAAAG

CTAATCTTCAAATTGGAAACATTCTGCCCGTGGGTGCCATGCCTGAGGGAACCATTGTGTGCTGTTTGGAGGAAAAGATG

GGAGACCGAGGCAAGATTGCCCGGGCCTCTGGAGACTATGCCACAATTATTTCCCATAATCCTGACACCAAAAAGTCCAG

GGTGAAGCTGCCTTCAGGGTCCAAGAAACTCATCCCCTCTGCCAACAGGGCCATGGTTGGTATTGTTGCCGGAGGTGGTC

GTATTGACAAACCCATGCTGAAGGCTGGCAGGGCTTACTTCAAATACAAGGCAAAGAGAAACTGCTGGCCTAAAGTTAGA

GGAGTGGCCATGAACCCCGTTGAGCATCCCCACGGAGGAGGTAACCATCAGCACATCGGTAAAGCTTCTACTGTCAGGAG

A---TCTGGTGATAGACTGACACGAGCATCAAAGGTGCTGGAACAGCTGACTGGCCAGCAGCCTGTCTTCTCTAAAGCCC

GCTACACTGTCAGGTCCTTTGGCATCAGAAGAAACGAAAAGATTGCTGTACATTGCACCGTGAGAGGAGCCAAAGCAGAG

GAAATTCTGGAGAGAGGACTCAAAGTTAGAGAATATGAATTAAAAAAGAACAACTTTTCTGAC---AGTCAGTTCCGCTT

TGTGGAAGTTGGCCGTGTGGCGTATATTTCCTATGGGCCCGATGCAGGAAAGCTCGCCGTTATTGTTGATGTTATAGACC

GGAACAGGGCTCTCGTAGATGGACCCTGCTCTGGTGTATCCAGAAAACAAATGAACTTCAAGGCTCTTGAGCTGACATCA

ATTGTGATGAAAATTCCACGAGGTCTGCGTCCAGGAGACCTGCGTAAGGCTTGGGAAAAGCAGGAAGTACAGAAGAAGTG

GGATAGCACAACATGGGCACAGAAAATTGACAGATTCAAGCTGATG---TACAAATACATGCAGGAGATTTATCGGAAGA

AGCAGAGTGATATCTTGAGGTTTCTGCTACGTGTTCGCAGCTGGCAGTATCGCCAGTTGTCGTCATGTCACCGGGCCCCG

CGTCCTACTCGTCCTGAAAAAGCCAGAAAATTAGGATACAAGGCTAAACAAGGTTTTGTTATTTACCGCGTCCGTGTTCG

TCGTGGTGGCAGAAAAGTACCTGTACCCAAGGGAATTACCTATGGTAAACCCACAACTAGTGGTGTAAACCAGAAGAAAT

TTCAACGATCACACAGATCCATTGCTGAGGAACGTGTTGGAAAGAAATGCGGTGGCCTGCGTGTGCTTAGCTCCTACTGG

GTAGCCCAGGACTCCTCGTACAAGTTCTACGAGATCATTCTGGTCGACCCATTCCACAAGACCATTCGTCGTGACCCCAA

GCTTCAATGGATCTGCAACCCAGTACACAAACACAGGGAACTGCGTGGGCTTACCTCGGCTGGCAAGATGTCTCGTGGTC

TGGGCAAGGGACACAGATACACCAAGACTATTGGAGGCTCTCGTCGTGCTGCATGGAAGAGGAACAATTTGACCAAAATG

CGCAGAAAGCGT---AGGTTCCTAGCTAGAAGAACTAATGCCAAGTTCAACAAGATTATCCTGAAGCGTCTGTTTATGAG

CAAGACCAACAGACCACCTCTGTCCGTTGCCAGACTGCGTCTGATGAAGAGAACAGGCAGAGACAACAAAACTGCTGTGG

TTGTTGGCACTGTGACTGATGATCTTCGACTTTTAGACGTTCCCAAGCTTAAGTTGTGTGCTCTCAATGTGACAGAGAGA

GCCAGGGCTCGTATCCTGAAGTCTGGGGGACAGATCATCACTTTTGATCAGCTGGCTCTCAAGTCTCCCAAAGGACAAAA

TACAGTGCTGATGCAAGGACCCAGAAAGTCGCGCAAGGCCTTCAGACACTTCGGAAGAGCACCTGGTGTTCCACACAGCA

GTACTGCACCATAC---AAGGAGTACCAGGTGGTAGGGCGGATGATGCCCCGCCCCAGGGATCGCACCCCTGCCCTGTAC

CAAATGAAAATCTTTGCCCCGGACATGTGTACAGCCAAGTCTAGGTTCTGGTACTTTGTATCACAGCTGAAAAAAATGAA

GAAGACCCAGGGCGAGATTCTCCAATGCAAAGAGATGTATGAGAAGCACCCCCTGGTCATCAAGAATATTGGCATCTGGT

TGAGATATGTATCCCGTAGTGGAATCCACAACATGTACCGTGAATACCGTGATTTGACAGCCGCTGGTGCAGTCACACAG

TGCCGTGACATGGGAGCCCGTCACAGAGCGAGAGCATCAACGATTCAGATCATCCGTGTGGAAGTTGTGGAGGCCTCTAA

GACCCGCCGACCCAACATCAAACAGTTCCACAACAGCAAGATCCGATTCCCTCTGACACACAGATTGAACAGGTTGCACA

AGCCAAGATTCAGCACATCTCGACCTCACTTA---CTCAGGTTGCAAAAACGGCTGGCCTCCGCCGTGTTGAAGTGTGGC

AAAAACAAGGTTTGGCTTGATCCCAATGAAACAAATGAAATAGCCAATGCCAACTCAAGACAAAATATTCGTAAATTAAT

TAAGGATGGTTTGATCATCCGTAAACCTGTTGCTGTGCACTCACGGGCCAGAGTTCGCAAGAACGCTATTGCCAGGAGAA

AGGGTCGCCACATGGGTCATGGAAAGAGGAAGGGTACAGCCAACGCACGTATGCCAGTCAAGGTCTTATGGATGCGCAGA

ATGAGAGTGTTGCGTCGTCTTCTTAAGCGCTACAGAGAAGCCAAGAAAATCGACAAGCACTTATACCATGAACTGTACAT

GAAGAGCAAAGGTAACGGATTCAAGAACAAGAGAGTCTTGATGGAGCACATCCACAAGAGGAAGGCAGAGAAAGCCAGAT

CTAAACAACTC---GACAGGTACCTCAGAGTCTGGAGGAAACCAAAAGGAATTGACAACAGAGTCCGTCGCAGATTCAAG

GGACAATACAAAATGCCAAACATTGGTTATGGATCCAACAAGAAAACAAGACATGTTATGCCCGATGGTTTCAAGAAATT

CCTTATTCACAATGTTAAGGAATTAGAGGTACTTCTGATGCAGAACAGGACTTTTGCTGCAGAAATTGCCCACAATGTTT

CCAGCGAGAAAAGGAAGGACATCGTGGAGCGTGCCCAGCAGCTGTCTATTAAGTTGACTAATCCTAATGCTCGTTTA---

ACACTGAGAGTTGCCAAGGTAACTGGTGGTGCAGCATCAAAACTGTCAAAAATTGTAAGAAAATCCATTGCTCGAGTGTT

GACAGTGATGCACCAGACCCAGAAAGAAAACTTGAGGAAATATTACAAATCTAAGCGCTTAAAGCCCACTGACCTCCGCA

AAAAGAAGACTCGCGCAATGAGAAGGGCCCTGACACCTTTCGAAAAGTCCATCAAGTCACGAAAACAGCAGCGCAGAGAA

AGACTCTACCCCATGAGAAAGTTTGCTGTTAAA---TCCAAACAGATGCAGCAGATTCGTCAAGCTCTGCGAGGAGCCAT

TGTGTTGATGGGCAAAAACACCATGATGAGGAAGGCCATCAGAGGTCACTTGGACAAGAACCCCCAACTGGAAAGACTCC

TACCCCATATTAAGAACAACATTGGGTTGGTGTTCTTGAAGGGTGATCTTGTAGAAACCAGGGACCTACTGGTGGCCAAT

AAGGTCAAAGCTCCAGCCAAGGCAGGTGCTCTCGCTCCCCTGGATGTCCATGTGCCCGCCCAAGCCACCACCCTGGGGCC

TGAGAAAACCTCTTTCTTCCAAGCTCTGCAGATCCCCACCAAGATTACCAGAGGGTGCATTGAAATCTTGAATGAGGTGC

ATCTGATTAAAGCTGGAGACAAGGTCGGTTCATCCGAGTCCACACTGCTTAACATGTTGGGCGTCTCTCCATTTTCCTAT

GGACTTGCTGTGGAGGTGTACGACTCTGGTACCATTTTTGAGCCTGAGATTCTGGACATCACCGATTCTGATCTGAGAGA

CAAGTTCTTGTTGGGTGTGAGGAACATTGCTGCCCTCTCCCTC---ACCCGTGCAGGTCAAAGAACCAGGTTCAAGGCAT

TTGTTGCTATTGGAGACCACGACGGTCATGTTGGATTGGGGGTGAAATGCTCCAAAGAAGTGGCAACAGCCATTCGTGGA

GCCATCATCCTGGCCAAGCTGACTGTTATTCCTGTCAGGAGAGGCTACTGGGGTAACAAGATCGGAAAACCCCACACTGT

ACCCTGCAAGGTGACTGGAAAGTGTGGCAGTGTTGTGGTGAGGTTGATCCCTGCACCCAGAGGTACTGGTATTGTCAGTG

CACCTGTCCCCAAGAAGCTGCTTCAGATGGCTGGAATTGACGATTGCTACACCTGTGCTAGGGGATGTACAGCCACACTC

---CTGGCAGAAGATGGCTACAGTGGTGTAGAAGTCCGTGTCACACCAACACGTACTGAAATCATTATCCTGGCCACCAG

AACACAGAATGTCCTCGGTGAAAAAGGCAGGAGAATCAGAGAACTGACCTCAGTAGTGCAAAAAAGGTTCAACTTCCCAG

AGGGAACTGTTGAGCTTTATGCAGAAAAGGTTGCACAGCGAGGTTTATGTGCTATTGCACAGTGTGAGTCTCTCAGATAC

AAGTTGATCGGAGGTCTGGCAGTCAGGAGGGCCTGTTATGGAGTGCTGCGATTTATCATGGAGAGTGGTGCTAAGGGCTG

TGAAGTCGTAGTATCAGGAAAGCTGAGGGGACAGAGAGCCAAGTCCATGAAGTTTGTGGATGGCCTTATGATCCACAGCG

GTGACCCCATTAATGATTACGTAGACACAGCCGTCAGGCACGTCTTACTCAGACAAGGTGTGCTTGGAATCAAGGTTAAG

ATCATGTTGCCCTGGGATCCCAGTGGCAAGATTGGTCCTAAGCGTCCCCTGCCTGACCATGTCAGTGTGGTGGAGCCTAA

GGATGAG---CGAGGACGCAAGAAGTTGAAGCGCTTGACTGCGCCGAAATCATGGATGCTGGACAAACTCGGAGGTGTCT

TTGCCCCCCGACCCAGCACTGGACCCCACAAGCTGAGGGAATCACTCCCCCTCATTGTGTTCTTGAGGAACCGTCTAAAG

TATGCCTTGACATATGATGAGGTTAAGAAGATCACCATGCAGAGATTGATCAAAGTCGACGCCAAAGTCAGAACTGACAA

GAACTTCCCCACAGGATTCATGGATGTTATTTCCATTGAGAAGACCAATGAAAATTTCCGTCTTCTCTACGATGTCAAAG

GACGCTTCACTGTTCACCGTATCCAGAAAGAAGAGGCCAAGTACAAGCTGTGCATGGTGACTGGAGGCCATAACTTGGGG

CGAGTGGGTGTGATTCAGCACCGAGAGAGACATCCAGGGTCATTCGACATTGTACACATCAAGGACTCTGTGGGCCACAC

ATACGCCACCAGATTGAGCTATGTGTTTGTGATTGGAAAGGGAAACAAGCCTTGGATTTCACTTCCAAAAGGAAAAGGTG

TCAAACTGTCTATTGCAGAGGAAAGAGACAGGCGTCTTGCTGCA---CTCAACATTTCATATCCAGCCACAGGCTGTCAA

AAACTAATTGAAGTCGATGATGAAAAGAAATTGCGTCCATTCTACGAAAAGAGGATGGCATCAGAAGTGTCAGCAGACAG

TCTTGGAGATGAGTGGAAGGGATATGTTCTCAGGATTGCTGGAGGCAATGACAAACAGGGATTCCCCATGAAACAGGGAA

TTCTCACCAACGTCCGTGTGAGGCTACTACTGAGCAAGGGACACTCCTGCTACCGCCCCCGGCGTACTGGAGAGCGTCGC

AGAAAGTCCGTCAGAGGGTGTATTGTGGACTCCAATCTCAGTGTTTTGGCCATGGTCATCGTCAAAAAAGGTGAAAAGGA

TATTCCTGGCTTGACTGACACGACAATTCCTCGTCGTCTGGGACCCAAGAGAGCTAGCAAGATCAGGAAGTTGTTTAATC

TCTCAAAGGAGGATGATGTTCGCCAGTACGTCGTCAAGAAGGCCAAGACAAAGGCCCCTAAAATTCAGAGACTGGTCACC

CCCGTGGTTCTC---ATATCTCGTGATAAATGGCACAAGAGGAGGAAGACGGGTGGGCGTATGACCCAGATCCGCAAGAA

GAGAAAGTTTGAATTGGGTCGTCCAGCTGCTAACACCAAGCTGTCGGTGCGTACAATGGGTGGCAACAAAAAATACCGAG

CCCTTCGTCTGGAATCGGGGAACTTCGCTTCAGAGGCTATCACAAGAAAGACGCGTATCATTGACGTCGTCTACAATGCC

AGCAACAATGAACTGGTCAGAACCAAGACTCTTGTCAAATCCTGTATTGTGCAGATTGATGCCACCCCATTCCGACAGTG

GTACGAGGCTCACTATGCCAAACCTTTGGGGAGGAAGGCTGGAGTGAAAGCTAAAGTGGCATCCCGCCCAGGACAGTGTG

GTCGATGTGACGGTTACATTTTGGAGGGAAAAGAACTCGAGTTCTACCAGAGAAAACTTAAAACTAAGAAG---AAAACC

TACACCACACCACGACGTCCCTTTGAGAAGGAACGTTTGGACCAAGAGTTGAAACTCATTGGCGAGTTTGGTCTCAGGAA

CAAACGTGAGGTATGGCGCGTAAAGTACACTCTCGGAAAGGTACGAAAGGCTGCCAGAGAATTGCTTACCTTGGATGAAA

AAGACCAGAGAAGACTTTTTGAAGGTAATGCCTTGTTGAGACGTCTTGTCAGAATCGGAGTGTTGGATGAAGGCAAAATG

AAGCTCGATTACGTGTTGGGTCTGAAATTGGAAGACTTTCTGGAGAGGAGACTGCAGACACAGGTCTTCAAGTTGGGGCT

TGCTAAGAGCATCCATCATGCCCGAGTTCTTATCCGACAGAGACACATTAGAGTCCGAAAACAGGTGGTCAATATTCCAT

CCTTCATTGTGAGATTGGACTCGCAGAAACACATTGACTTCTCCCTCAGATCACCATACGGTGGTGGC---TTTCAGAAA

CAGCCAATTGTTTTTCTGAACAACAAGCGCCATTTCAAGAGTGTTGGTCTAGGATTTAGGACACCAAGACTTGGAACCTA

CATTGACAAGAAATGTCCCTTCACAGGAAACGTGTCCATCCGTGGACGCATTCTGACAGGTGTTGTCCAGAAAATGAAAA

TGCAGAGAACAATTGTGATCCGACGCGACTACCTGCACTACGTTAAAAAGTACAAGAGATTCGAGAAGAGGCATAGAAAC

ATGTCTGTGCACCTTAGTCCATGCTTCAGGGACGTGGAGATGGGAGACATTGTGACTGTAGGTGAATGTAGACCACTCAG

CAAAACTGTC---GGTATATCCCAGTCAGCCCTACCATACAGGAGATCTGTACCCACATGGCTAAAGTTAACATCTGAAG

ATGTACAGGAGCAGATAATGAAATTGGCCAAGAAAGGCCTTACACCATCTCAGATTGGTGTAATTCTGGGAGATTCTCAT

GGTGTAGCCCAGGTGAGATTCGTAACAGGAAACAAAATATTGAGGATCCTTAAGGGTAAAGGCATGGCCCCTGATTTACC

AGAGGATCTATACCACCTCATCAAGAAGGCTGTCAACATCAGAAAACACATGGAGAGGAACAGGAAGGACAGAGATTCCA

AATTCCGTTTGATTCTGGTAGAGAGCAGAATTCACAGATTGGCTAGATATTACAAAACAAAGAAAGTTTTGCCACCAAAC

TGGAAA---ATGGTGCGGATGAATGTATTGGCAGATGCCCTCAAATGCATAGCAGCAGCTGGGAAAAAGGGCAAACGTCA

GGTGCTAATAAGGCCCTGCTCAAAAGTCATCGTCAGATTTCTCACAGTGATGATGAAGCATGGTTACATTGGGGAATTTG

AAATTGTTGATGACCACAGAAATGGCAAAATTGTTGTCAATCTTACAGGGCGATTAAACAAATGTGGTGTCATCAGTCCT

AGATTTGACATTTCCATTAGAGACATGGAAAGATGGACAACAAATTTATTGCCATCTCGACAGTTTGGATTTATTGTTAT

GACAACGTCAGGGGGTATAATGGATCATGAAGAAGCCAGACGGAAACATTTAGGAGGAAAAATTCTTGGATTCTTTTTC-

--GAGGCTCCCATCCATAGAATTCGTATTACACTTACCAGCAGGAATGTTAAAAGTCTGGAAAAGGTGTGTGCTGATCTT

ATCAAGGGAGCCAAGGAGAAGTCCCTGAAGGTGAAGGGACCAGTGCGCATGCCCACCAAGGTTCTGCGCATCACCACCAG

GAAGACGCCCTGTGGAGAAGGTTCCAAGACCTGGGATAGATTCCAGATGCGCATCCACAAGCGCCTCATCGATCTGCACA

GTCCCTCCGAGATTGTCAAGCAGATTACATCTATCAGTATCGAACCCGGAGTTGAGGTCGAGGTCACCATTGCTGAT---

ACCATCCGAACAAGAAAGTTCATGACAAATCGTTTGCTATGCAGAAAGCAAATGATTGTAGATGTATTGCATCCAGGCAA

AGCCACAATTCCTAAAACAGAAGTCCGAGAAAAACTGGCCAAAATGTACAAGACCACGCCAGATGTCATTTTCTGCTTTG

GATTCAGGACTAAGTTTGGAGGAGGCAAAACCTCAGGATTTGGTTTAATTTATGATAGCTTGGACTTTGCCAAGAAGTTC

GAACCCAAATACAGATTACAGAGA---GGTAAACCCAGGGGTTTGAAGACAGCTAGAAAGTGTGTCAACCACAGACGGGA

CCAGAAATGGCACGATAATGACTACAAGAAAGCCCATCTACCCTCAAGATGGAAGCCCTTCCAGGGGTCCTCCCACGCCA

AGGGAATCGTCTTGGAAAAAGTTGGAGTTGAGGCTAAGCAGCCAAACTCTGCCATCAGAAAGTGTGTCAGAGTACAGCTC

ATCAAGAACGGAAAGAAGATCACAGCCTTCGTCCCTAACGACGGTTGTCTGAACTACGTAGAGGAAAACGATGAGGTTTT

GGTGGCTGGATTCGGTAGAAAAGGTCACGCCGTCGGAGATATTCCCGGTGTCCGCTTCAAGATCGTCAAAGTGGCCAACG

TATCACTCCTCGCTCTGTTCAAGGGAAAGAAAGAACGACCTAGGTCAATG---AGACAAGCCAGGAAGAATGGCAACTTC

TATGTCCCTGATGAACCCAAGCTAGCTTTTGTGATGCGAATCAGAGGTATCAATGGTGTTCATCCCAAGCCAAGGAAGGT

TATGCAGCTTTTCCGTTTGAGGCAGATTAACAATGGTGTTTTTGTGAGACTTAACAAGGCCACGATTCACATGTTGAGAA

TCGCTGAACCCTTCATTGCATGGGGGTACCCCAACCTCAAGAGTGTGAGGGAATTGGTGTACAAAAGGGGATATGGAAAA

GTTGGACGTATTCCTCTCACAGACAATCTCATTGAGAAACACCTCATCATCTGTATGGAAGATTTGATCCACGAGATCTA

CACAGTTGGACCCAACTTCAAGTATGCTGCCAACTTCTTGTGGCCATTCAAACTGAACACACCTAATGGTGGATGGAGAA

GGAAGTACAACCACTTCAACGACGGTGGTGACTTTGGCCTGCGT---CAAGACAAGCCAAGGAAGTTTACTCAATCCGTA

GAACTTCAGATTGTGTTGAAAAATTATGATCCCCAAAAGGACAGGCGTTTTGCTGGAACAGTGAGATTGAGGAATATTCC

AAGACCCAAGATGAAGGTCTGTATCCTTGGAGATCAGATTCATTGTGACCAAGCAAAAGCTAACAATTTGCCCTGCATGA

CTGCTGATGACCTGAAGAAGCTAAACAAGGACAAAAAATTGATCAAGAAATTGGCCAAGAAGTACGATGCCTTCCTTGCC

TCTGATTCCTTGATCAAGCAGATTCCCAAGATCTGTGGGCCTGGATTTAACAGAGCTGGAAAGTTCCCTACCCAGGTATC

CCATCAGGAATCCTTGGTACAGAAAGTGGAGGAAGTCAAGGCAACCATAAAGTTCCAGCTTAAAAAAGTGCTGTGTTTGG

CAACTTGTGTTGGACATGTAAACATGACCAAAGAGGAGTTGTATGGAAATATTACCCTGGCCATCAACTTTTTGGTTTCC

CTGCTGAAGAAGAACTGGCAGAACGTCAAGGCCTTGTACATCAAAGGAACCATGACTCCAGCTCAGAGAATCTAC---GG

GCATTTCCATAAAGATTGGCAACGGTATGTGAGAAACTGGTTCAACCAACCAGCCAGGAAGAAGAGGCGACACGACAACA

GAGTCAATAAGGCCAGACGTATCGCTCCCAGACCTGTGGCTGGACTGAGGCCCATTGTGAGATGTCCAACCTTCAAATAC

AACACTAAAATTAGGGCTGGCAAGGGATTCACTCTGGATGAACTGAAGGCTGGGATCAACAAGAAAGTAGCTCTTACGAT

CGGGATTGCTGTGGACTACAGGCGTAGAAACAAGAGCCTTGAATCTTTGCAGCAAAATGCCCAAAGACTGAAGGAGTACA

AATCTAAACTGATCCTCTTCAAGAAACAGAGCAAACCC---ATGGTTCGATACGCTACAGAACCAGATAACCCAACCAAA

TCGGCCAAGGCCAGGGGGTCTTATCTCCGTGTCCACTTTAAGAACACAAGAGAAACTGCACAGGCTATTAAGCGTATGCA

CGTTAAGAGAGCCACCAGATACCTAAAGAATGTGATGGCGAAGAAGGAGGTGGTTCCTTTCCGTAGATTCAGTGGTGGAG

TGGGGCGTTGTGCTCAGGCTAAGGCCTTCAAGCATTCCCAAGGTCGTTGGCCATCGAAAAGTGCTGAATTCTTGTTGCAG

CTTTTGAAGAATGCTGAGAGTAATGCCGAATACAAGGGATTGGATACAGATCACTTGGTCATTGAACACATCATGGTGAA

TGCTGCACCCAAGATGAGAAGGAGAACCTACCGTGCTCATGGTCGCATTAACTACATGAGCAGTCCCTGCCACATTGAAC

TGATCCTG---ATGAAGTTCAACAAGATGGTGACCTCCTCTAGGAGGAAGAATAGGCAGCGTCATTTCAACGCTCCTTCA

CATATTCGTAGGAAGATGATGAGTTCCCCCCTTTCAAAAGAACTTCGTCAAAAATACAACGTTAGAAGCATGCCCATTAG

GAAGGATGATGAAGTTCAGGTTGTTAGAGGCCATTACAAAGGGCAACAAGTTGGTAAAGTTCAAGTTTACCGCAAAAAAT

TTGTGGTTTACATTGAAAGAATCCAGAGGGAGAAGCTAACGGTGCATCAGTCCATGGTGGGAATCCATCCCTCTAAGGTG

GTGATTGTGAAATTAAAGATTGACGAGGATCGCAAGAGGATCCTGGACAGGAACGGCGCTCCAAAGGGCAAA---CCAGG

CGAAGTGGTGCTTGTGTTAAGTGGACGATTTGCCGGAAGAAAGGCAGTCATTGTTAAGAACCATGATGATGGTACACAAG

ACAAACCTTACGGACATGCCCTTGTAGCAGGAATCGACCGCTACCCACGTAAAGTCATCAGAAAGATGGGCAAGAAGAAG

ATGAAGGACAGGTCCAAGCTCAAGGCTTTCTTAAGGGTGTACAACTACAACCACCTTATGCCCACAAGATACTCAGTGGA

TGTTAGGTACAAGACAGGAAAAAACAAATGGTTTTTCCAGAAGTTACGGTTC---TTGCGTGGTCATGTCAGTCACGGAC

ATGGTCGCATAGGTAAACACCGTAAGCATCCAGGAGGTCGTGGTAATGCTGGTGGTCAGCATCATCACAGAATAAACTTT

GATAAATACCATCCTGGTTACTTTGGAAAAGTTGGTATGAGGTATTTCCATAAGACAAACAACAAATTTTACTGTCCAAC

AATGAATGTTGATAGAATTTGGTCCCTCGTCACAGAGCAGACAAGAGAAAAGGCACCTGTCATCGATTGTGTCAGAGCAG

GCTATTTCAAGGTTCTTGGAAAGGGACATCTGCCTAAGCAGCCATGTATTGTGAAGGCTAAATTTTTCAGTCAGTTGGCC

CAGAAGAAAATTCGACAAGCTGGAGGAGCCTGTGTCCTGGTGGCT---ATGGGTGCCCACAAAAGTTATCGCACCAAAAT

GCGATTAGCCAAGAAACAGAAGCAGAACCGCCCAGTCCCACAGTGGGTTAGAATGAAAACTGGAAACACTATCAGATACA

ACGCCAAGAGGAGACACTGGAGGCGTACAAAGCTAAAGATG---ATGGCACGTACAAAGCAGACAGCAAGAAAATCTACA

GGAGGTAAAGCTCCAAGAAAACAACTGGCCACAAAGGCAGCACGTAAAAGTGCCCCTTCTACTGGCGGAGTGAAGAAACC

CCATAGATACAGGCCAGGAACTGTCGCTCTCCGAGAAATCAGGAGATACCAAAAGTCAACTGAGTTGCTCATCCGCAAAC

TACCTTTCCAGCGTTTGGTGAGAGAAATTGCTCAGGACTTCAAGACAGACTTGAGATTCCAAAGTGCAGCTATTGGGGCT

TTGCAAGAGGCCAGTGAAGCTTACTTGGTGGGATTATTTGAGGACACCAATTTGTGCGCTATCCACGCAAAGAGAGTCAC

AATCATGCCCAAAGACATTCAGTTGGCCAGAAGAATCCGTGGTGAACGTGCA---CTTGCCTGCGTTTATGCAGCTTTGA

TTCTCGCTGACGACCAAGTTGCGATCACTAGTGATAAAATTGCAACCATCTTGAAGGCTGCTGGAGTGTCTGTTGAACCA

TACTGGCCA---ATGCGTTACGTAGCAGCCTACTTGCTTGCAGCCCTCAGTGGCAACAACAGTCCATCCGCAGATGACCT

GAAGAAAATCATCGGGTCTGTAGGAATTGACTGTGAGGCTGATAAAATCACAAAAATCATCGGTGAATTGAAAGGAAAGA

ACCTTGAAGAACTCATCACTAAAGGT---GAAAAGGAATGGATGCCAGTGACCAAGCTTGGCAGACTTGTCAAGGATCAA

AAGATTAAAACTCTTGAAGAGATTTATCTCTTTTCTCTGCCCATCAAGGAGTTTGAGATCATTGACTATCTTCCATCCCT

GAAGGATGAAGTTCTCAAGATTATGCCTGTGCAGAAACAA---AAATATTTGCCACACTCCTCAGGCAGATACCAAGTAA

AGAGATTTAGAAAATCACAGTGCCCAATTGTTGAACGCCTGACATGTTCACTTATGATGCATGGAAGGAACAATGGAAAG

AAACTCTTGACAACCCGCATTGTGAAACATGCCTTTGAAATCATTCACTTGCTCACAGGAGAGAACCCTCTCCAAGTTTT

GGTGAATGCCATCATCAACAGTGGCCCCCGTGAGGACTCCACTCGTATTGGTCGTGCTGGTACCGGGGCACGTGAAGCCT

CCTTCAGGAATATCAAGACTATTGCTGAGTGTTTGGCTGATGAGCTGATCAATGCTGCCAAGGGATCTTCAAACTCCCAT

GCTATCAAGAAGAAGGATGAA---AGCGCAAAAATCGTTAAGCCCCAAGGGGAAAAGGTTGACGAGTTTGAATCGTCAAT

TTCTCAGGCTCTGCTTGAGTTGGAGATGAACAGTGACATGAAGGCCCAACTGAGGGAGCTGTTCATCACTGGTGCCAAGG

AGATCGACGTGCAGGGCAAGAAGGCCATCATCATCTTCGTGCCAGTTCCCCAGCTGCGCGCCTTCCAGAAGATCCAGATC

AGACTGGTCCGTGAGTTGGAGAAGAAGTTCAGCGGCAAACATGTAGTATTTATTGCACAGAGGAGAATCCTCCCTAAACC

AACCAGAAAGACAAGAAAGAGCAAGCAGAAGAGACCTAGATCACGTACACTGACTGCAGTCCATGATGCCATGCTTGAGG

ATCTAGCTTTCCCTGCTGAAATTGTCGGCAAAAGAATCAGAATCAAGCTGGATGGATCGCGACTCATTAAAGTACACCTG

GACAAGAACCAACAGACAAACATTGAACACAAAGTGGATACATTCTCTGCTGTGTACAAAAAACTTACAGGAAAAGACGT

TGTTTTTGAGTTTCCTGAATTTGTGTTG---ATGCCCAAGAAAAACCGTGTGCAAATATTTGAATACTTGTTCAAGGAAG

GCGTTTTGGTAGCCAAGAAAGACTTCTCAGCTCCAAAGCATCCAGAAATTGATGTCCCTAACCTTCATGTTATTAAGGCA

TTAACATCTTTGAAGTCACAAGGCTATGTAAGAGAACAGTTCTCATGGCGTCATTATTACTGGTACCTGACAAATGAAGG

CATCCAGTATTTACGTGATTATCTCCATCTGCCAGCAGAGATTGTTCCTGCTACACTAAAG---CATGTCACCGATTTGT

CTGGAAAAGGGGCCATTGCACGTATCACTGGTGGTATGAAGGTCAAGGCAGACAGAGATGAAGCTTCTCCATACGCTGCT

ATGTTGGCTGCTCAGGATGTCGCAGAGAAGTGCAAGACCTTGGGAATCACTGCTCTCCACATCAAACTGAGGGCTACTGG

AGGAAACAGGACCAAGACCCCAGGACCTGGAGCACAGTCTGCTCTGAGAGCCTTGGCCCGTTCAGGAATGAAGATTGGAA

GAATTGAGGATGTG---AAGAAGAAGAAGCGTACCTCCAGAAAGTATGCTTACAGAGGAGTTGATCTTGACCAGCTTTTG

GATATGTCCAATGAGCAGTTGATGGAACTCTTCCCCTGCAGAGCTAGACGCAGGATTTCTCATGGTTTGAAACGCAAACC

TCTCGGCCTCATTAAACGTCTTCGTAAGGCCAAGAAAGAAGCAGGTCCACTTGAGAAACCAGAGGTAGTGAAAACGCACC

TGCGAAACATGATCATTGTCCCAGAAATGATCGGAAGCATCATTGGAGTTTACAATGGCAAGACCTTCAACCAGGTGGAA

GTCAAGCCCGAGATGATCGGCCATTACCTTGGGGAGTTTAGCATTACATACAAACCTGTTAAACATGGTCGTCCAGGTAT

TGGTGCCACCCACAGCTCCAGATTTATACCACTGAAG---ATTATTGAAAAATATTACACTAAGCTCACTTTGGATTTCC

ATACCAACAAACGAGTATGTGAGGAAATTGCAATCCTGCCCAGTAAGAAACTGAGGAACAAGGTTGCAGGGTTCGTCACT

CACTTGATGAAGCGTATTCAGAGAGGGCCAGTGAGAGGTATCTCCATCAAACTGCAGGAGGAGGAACGTGAGCGCAGAGA

CAACTACGTCCCAGAGATCTCCGCTTACATTCATGACATTATTGAAGTTGATCCAGACACTAAAGAAATGTTGAAAGCC-

--CCAAGAAAATGCTCTGCAAGTAACAGAATTATTGCTGCAAAGGACCATGCTTCTATCCAGATCAACATCGCTGATGTC

GATCCCACCACTGGACGCATGACTGGATCCAACACAACATATGCCATCTGTGGAAATATCAGGCGTATGGGAGAATCAGA

TGACTTCTTGATCAGACTGGCTAAAAGAGATCACCTCATAGCTCAG---CATGTGCAGTTTGTTCGTTGCACAAACTGTG

CTCGATGTGTACCAAAAGACAAGGCCATCAAGAAGTTTGTCATTAGGAATATTGTAGAGGCTGCTGCAGTCCGTGACATC

GCTGACGCTAGTGTTTATGAAGTATATGCTCTCCCCAAACTCTATGCTAAATTGTTGTACTGCGTTTCCTGTGCAATCCA

CTCTAAGGTGGTCAGGAACAGATCCCGTGAGGCCAGGAAGGACAGATCACCACCCCTCAGGTTCAGACCA---ATGATCT

TTGTTAAAACCCTCACGGGTAAAACAATTACCCTAGAGGTTGTCAAAGCCAAGATTCAGGAAGGAATTCCCCCTGATCAA

CAGAGGCTGATCTTTGCCGGTAAACAGCTGGAGGATGGCGACATCCAGAAGGAGTCCACCCTCCATCTGGGAGGGGCCAA

GAAGAGGAAGAAGAAGAACTACACCACCCCTAAAAAGAACAAGCACAAGAAGAAGAAGGTCAAGCTGGCCGTCCTCAAGT

ACTACAAGGTGGATGAGAACGGAAAGATCACACGTCTGAGGAGGGAGTGTCCCAATGAGGAATGTGGAGCAGGTGTCTTC

ATGGCATCCCACTTTGACAGACAGTACTGTGGGAAGTGCTGTCTCACCTATGTATTCAATAAA---AAACCATATCCCAA

GTCCAGATTTTGTCGTGGTGTCCCAGATCCTAAGATCCGTATCTTTGATCTTGGGAGGAAGAAGGCCAGGGTAGATGAAT

TTTCTCTGTGTGTGCATCTAGTTTCTGGTGAGTATGAACAGTTGTCATCAGAAGCGTTAGAGGCTGGCCGTATCTGTGCC

AACAAGTACTTGGTCAAGAACTGTGGTAAAGACGCTTTCCACATGCGGATACGAGTGCACCCCTTCCACGTCATCAGAAT

CAACAAGATGTTGTCGTGTGCCGGAGCCGATAGGCTTCAGACAGGAATGAGAGGAGCTTTTGGTAAGCCACAAGGAACCG

TTGCTCGCGTACACATTGGACAGCCCATCATGTCCGTCCGTGCCCGTGAGAACCACCGGGCATCAGTGATAGAAGCTCTC

CGTAGAGCCAAGTTCAAGTTCCCAGGACGTCAGAAGATCCACATTTCCAAGAAGTGGGGCTTCACG---AAAAGAACAAA

GAAGGTGGGAATTTGCGGGAAATACGGAACCCGATATGGTGCCTCTCTCCGTAAAACCATCAAAAGAATGGAAGTCTCTC

AGCATTCTAAATACACATGTCAATTCTGTGGAAAGGATGCCATGAAGAGGAAAGCTGTGGGGATCTGGAGCTGTAGTCGA

TGCAGGAAGACCGTGGCCGGAGGTGCATGGGTCTACAGCACAACTGCCGCCGCTACCGTCAGAAGTGCAGTCCGTCGTCT

CCGTGAAATG---CACATACAGAAAAAAACTTGTGCGTCATGTGGATACCCAAGTCCAAAGAGGAGGCATTTTGAATGGA

GTGAAAAAGCTAAAAGGAGGAGGACAACCGGTACTGGTAGAATGAAACATTTAAATAAAGTTTTCAAAAGATTCAAGAAT

GGCTTTAGAGAAGGTCAACCA---GCTGGTAAAGCGTCACTTCATGCACAAGGCAAACGTCGTTACGACAGAAAACAGAA

AGGTTATGGTGGACAAACTAAACCTATTTTTCACAAAAAGGCCAAGACAACCAAAAAAGTTGTATTAAGGTTAGAATGTT

TGAGATGTAATTTAAGAAAACAAGTGGTCATTAAACGATGTAAACATTTTGAATTGGGTGGAGACAAGAGAAAGAAG---

TTAACTAAGCATGCAAGATTTGTCAGAGACTTAGTGAGAGAAATCACAGGATTTGCTCCCTACGAAAGACGCATGCAGGA

GTTGTTGAGAATTCAGAAAGACAAAAGAGCACTCAAATTTGCCAAGAGAAGGCTTGGATCACACATTAGGGCCAAGAGGA

AGAGGGAAGAAATGCTGGCAGTATTGCAGAAGATGAGAAAGGCACAG---AAGACCATTGAGAACATCAATTCAAGGCTT

GCCTTGGTGATGAAATCTGGGAAATATGTTCTTGGATACAGACAGACCTTGAAAACTCTCAGACAAGGCAAAGCCAAGGT

GGTCATCATTGCCAACAACACTCCTCCCCTCAGGAAAAGTGAGATTGAGTATTATGCCATGTTAGCCAAGACTGGAGTTC

ATCATTACACAGGAAACAACATTGAGTTGGGAACAGCCTGTGGTAAATACTTCAGGGTGTGCACAATGAGCATTACTGAT

CCAGGC---AGAAAGTTGAGGTCATTCTCCAAGCACAAGCACAGCTTGAGGTCATCAATTACCCCAGGAACTGTTCTTAT

CCTAGTGGCTGGTCGTCACAAGGGAAAGAGAGTTGTCTTCCTGAAACAATTGGATTCTGGATTACTGTTGGTTACAGGCC

CATTCCATCTTAATGGTTGCCCTCTGAGAAGAATCAACCAAATTTACACTATTGCCACTAAAACAAAGCTGGACATCAGT

GGTGTTAAATTGCCCGAACGCTTGAATGACAAATATTTCAACCGTAAACAGACAGAAGGAGAAATCTTTGACACCAAAAA

GGAGTACACTGTCAGTGATGAAAGGAAAGAAGACCAAGTGGCTGTTGATAAACAAATTCTG---GTAATCATCAAAGGAC

CTCGAGGAACTTTAACAAGAACCTTCCGTCACTTGGATATCACCATGGTAAGGACTGTGTGCAGTCACATAGAGAACATG

ATCAAGGGGGTTACGGGCTTTCTGTACAAAATGAGGTCAGTGTATGCCCATTTCCCCATCAACTGTGCAGTACAAGAGGG

TGGAGGTTCAGTGGAGATTAGGAACTTTTTGGGAGAAAAATTTGTCAGGAAGGTGAGAATGAGGGCTGGTGTTGTCTGTT

CAGTCTCAGCGGGCCAGAAGGACGAGCTGATCCTGGAAGGCAATGACATTGAGCTGGTGTCAAACTCATCTGCTCTGATC

CAGCAGGCCACCACAGTA---GTGTATGTGAAAGCAGTTGGAGGTGAAGTTCCAGCCACTTCATCCTTGGCTCCTAAAAT

TGGTCCTCTGGGTTTGTCTCCAAAGAAGATTGGTGATGACATTGCCAAAGGCACAGCAGATTGGAAAGGCCTAAAAATCA

CGGTGAAGCTTGTGGTCCAGAACCGTCAAGCCAAGGTAGAAGTGGTGCCTAGTGCTTCTTCACTCCTCATCAAGAGCCTG

AAGGAACCACCCAGGGATAGAAAGAAGGTTAAACATGTCAAGCACAGCGGAAACCTCTCTATGGATGACGTTATTGCTAT

TGCACGTCAAATGCGTCCAAGGAGTATGGCCAAAAATCTCGCTGGTACATGTAAAGAAATTTTAGGTACGGCTCAGTCTG

TTGGATGCTCCATTGAGAGATCTCCTCCT---GATGCTCGAGGGCATCTTATGGGAAGACTTGCAGCAATTGTTGCCAAG

ACTGTACTGCAAGGTCAGCGAGTTGTTGTTGTACGATGTGAAGGCATCAACATTTCTGGAAATTTCTACAGAAATAAATT

GAAGTACTTGAAGTATCTGAGGTACAGATGCAATGTGAACCCCTCGAGAGGACCCTTCCATTACCGTGCTCCTTCTAGGA

TCTTCTTCAAGGCTGTAAAGGGTATGATCCCACACACCACAACTCGTGGTAAAGATGCCCTTGGCCGTCTGAAGGTGTTT

GAGGGTGTGCCACCCCCATATGACAAACAGAAGAGGAAGGTAGTTCCAGCTGCCCTCAGAATCCTCAGACTGAAACAAAA

CAGAAGGTTCTGTGATTTGAACGGACTTGCCCATGAAGTTGGCTGGAAATACCAGGGTGTTATTGGTACTCTTGAA---A

TGACGAACACAAAGGGATACAGGCGTGGAACGCGTTACATGTTCTCCAGGCCATTCAAAAAGCATGGAGTCATTCCCCTG

TCAACCTACATGCTCAACTACAAGAGAGGTGACATTGTTGATGTCAAGGGTCATGGAGCTGTTCAAAAGGGCATGCCACA

CAAGTTTTACCATGGCAAGACTGGTAGAGTTTTTAATGTAACTAAACACGCTGTTGGAGTCATCATCAACAAGAGAGTGA

GGGTCATCCCCAAACGAATCAACGTGAGAATTGAGCACGTGAAACACTCCAACTGCCGACTCGAGTTTTTGAAACGAGTG

GCCAATAATGAGCAGGCGAAAAAGGCTGCCAAGGAGGGAGGAGAAAGGAAGAACCTGAAAAGACCCAGAAAGAAGCACTT

TGTCAGCAAGTACAACAAGCCTGAAGTGTTGGAACCTATACCTTATGAATTCATTGCA---GTTTTGACTGAGGTCATCA

CCAAGGAATGCACCATCCACATGCACAAGAGGATCCATGGAATTGGATTCAAGAGGAGGGCACCCCGTGCAATTAAGGAG

ATCCGCAAGTTTGCCGAGAAGATGATGGGTACCCCTGATGTTCGTGTGGACACCAGACTCAACAAACACATCTGGTCTCA

GGGAGGTAGGAATGTTCCATACAGAGTACGTGTCAGGTTAGCGCGTAAAAAGAACGAAGATGAGGACTCTGTTCACAGAC

TGTATACCTTGGTCACGTATGTGCCCTGTGCTTCATTTAAA---ACCTTGGTCACCAGAACTCAAGGAACCAGGATTGCT

TCTGATGGCCTGAAGGGGCGAGTGTTTGAGGTCTCCCTGGCTGATCTTCAGAATGATGAAGTATCCTTCAGGAAGTTCAA

ACTGATGGCTGAAGAAGTTCAGGGACGCAATGTTCTGACCAACTTCCACGGCATGGATCTGACCCGTGACAAACTCTGCT

CTATGGTCAAGAAATGGCAGACCATGATCCAGGCTCATGTTGATGTCAAGACCACTGATGGTTACCTTCTTAGGATGTTC

TGTATTGGTTTCACCAAGAAGCAGTTTCAAGTGAAGAAAACTTGTTACGCCCAAACCACACAGATCAAAACCATCAGAAG

AAAGATGGTTGATATCATCACTAAGGAAGTGACATCAAATGACATGAAAGAAGTTGTCAACAAACTAATCCCAGACAGCA

TTGGCAAGGATATTGAGAAAGCCTGCCAAGGAATTTATCCCCTTCACGATGTTTTCATCCGTAAGGTCAAAGTGCTGAAA

AAACCTAAGTTCGACACAGACTCCGGCGAAACTGTGGCGCGGCCTGAGGGATACGAACCTCCAGTCCAACAATCTGTA--

-GGACGACCACTGGAGCAGGTTGAACCACAGATCTTAAGATACAAGCTCCAGGAGCCCATCCTGCTGCTGGGTAAGGAGA

AGTTTGCTGGGGTGGACATCAGAGTGCGCGTCCGAGGGGGTGGTCATGTGGCCCAGGTCTACGCTATTAGACAGGCTATC

TCCAAGTCTTTGGTAGCTTATTACCAGAAAGTTGATGAGGCCTCCAAGAAAGAGATTATGGATGTCCTGATCAGCTACGA

CAGAACTCTGTTGGTCGCTGACCCCAGACGATGCGAGCCCAAGAAGTTTGGAGGTCCCGGCGCTCGTGCCCGCTACCAGA

AATCTTACCGT---CGATATGCTAATGTTGTCTGCAAGAAAGCTGATGTAGATATCACAAAAAGGGCAGGGGAACTCTCA

GAAGAAGAGAAAATTGTCACAATTATGCAGAACCCTCGTCAGTACAAGATTCCTGACTGGTTCCTTAACAGGCAGAAGGA

CATTAAGGATGGTAAATTCAGCCAGGTCATGTCCAACACACTGGACAACAAACTCCGTGAGGATCTGGAGCGACTAAAGA

AGATCCGAGCACACAGAGGTCTCCGTCACTACTGGGGTCTAAGAGTGAGAGGTCAGCACACAAAGACCACAGGAAGAAGA

GGAAGAACTGTTGGTGTGGCCAAGAAG---ATGAAGATTCCAGACTGGGCTCCCATTGTGAAGCTTGCCAAATACAATGA

GCTTGCTCCATATGATGATGACTGGTACTACACACGGGCCTCTATTTGCCGCCATTTGTACATTAGACCTGTTGGTGTTG

GAGCTTTTACAAATCTGTATTCAGGACGCAAGAGGAACGGAACAGCTCCCAGCCATTTCTGCAGAGGCAACTCATCAGTG

GCACGCAAGGTTCTGCAGTCTCTGGAGGGAATGAAACTGGTAGAGAAGGATACAGCCGGTGGGAGGAAACTCACCCCACA

AGGCAGGAAGGATCTAGACAGAATTGCTGCCCAAGTG

>Lumbri

TCTCACCGTAAATTTTCCGCACCCAGACATGGTTCTTTGGGTTTTCTGCCGAAGAAGAGAAGTCGTCGCCATCGTGGAAA

GGTTAAGGCTTTCCCGAAGGACGACAAGAGCAAACCCGTTCATCCGACGGCCTTCATTGGATACAAGGCTGGCATGACCC

ACGTCGTGAGAGAAGTTGACAGACCTGGATCAAAGGTTCACAAGAAGGAGGTCGTTGAGGCGGTGACAATCCTTGAGACG

CCTCCAATGATCGTTGTTGGTCTCGTTGGATACATTGATACGCCGAAAGGTCTGCGTACGTTCAAGACCGTCTGGGCGGA

ACATCTGAGCGATGAATGCAAACGTCGCTTCTACAAGAACTGGTACTGCTCCAAGAAGAAGGCTTTCACGAAGGCGTCGC

TCAAGTGGCAGGACGAGTCTGGGAAGAAGGAGATCGAGAAAGACCTGAGCAAGATCAAGAGATACTGTTCGGTTGTGAGA

ATCCTTGCCCACACTCAGATGAAGCTGATGAACAGACGTCAGAAGAAGGCGCACATCATGGAGATCCAGTTGAACGGTGG

ATCTGTTCCAGACAAAGTGGAGTGGGCCAAGACTCATCTGGAGAAGCCCATCCCTGTCCAGCAGGTGTTTGACCAGGACG

AAATGATTGATGTTATCGGAGTGACCAAGGGAAAGGGATTCAAGGGTGTGACTTCTCGTTGGCACACAAGGAAGCTTCAG

CGCAAGACGCACAAGGGTCTGCGTAAGGTTGCGTGTATTGGAGCGTGGCATCCGTCTCGTGTGTCGTTCTCTGTCGCTCG

TGCT---CGACCATTAGTAACCGTCTACAATGAGAAGGGTGAGTTGGTGAAGATGCCAGCAGTATTCCAGGCACCAATAC

GACCTGACATTGTCGGCTCCGTTCACATGGACATGATGAAGAATGGACGTCAGCCATATGCTGTCTCTGTGAAAGCTGGA

CACCAGACATCCGCAGAATCTTGGGGAACTGGAAGGGCTGTTGCACGTATTCCTCGTGTGAGTGGTGGCGGTACAAACCG

CTCTGGACAGGGTGCATTTGGAAACATGTGCCGTGGTGGACGCATGTTTGCACCTACCAAGACCTGGCGCCGTTGGCATC

GTCGCATCAACGTCAATCAGAGACGCTATGCCATCTGTTCTGCGATTGCTGCCACTGGTATCCCGGCTCTGGTTATGTCC

AAAGGACACTGCATTGAGGAAATCCCTGAACTCCCTCTGGTTCTGACTGATAAAGTCCAGGAAATCAAGAAGACAAAGGA

GGCTGTTGGAGTTCTGAGAAAGCTGAAGGCCTGGCGTGAAATTGAAAAGGTGAAGAGCACTAATCAAGATAACGGAATCA

CCCGTGCTTTCAGGAACATCCCAGGTATCACCCTCATCAATGTTGACCGCCTGAACCTCCTGAAAATCGCTCCGGGTGGC

CATGTTGGACGCTTTTGCATCTGGACTGAGTCGGCGTTTAAAAAACTGGATTCCATTTAT---CGACGAGAGGGTAAAAC

TGATTACCGTGCTCGTAAGAGACTAGTTGTGCAAGACAAGAACAAGTACAACACGCCGAAGTACCGAATGATTGTGCGCA

TTACCAACAAGGACATCATTTCTCAGATAGCGTATGCCAGAATCGAGGGTGACGTGGTTGTGTGCGCCGCCTACGCACAC

GAGTTGCCCAAGTTTGGCATCAAGGTCGGGCTGACAAACTACTCCGCGGCTTATGCAACTGGACTTTTATTAGGACGCAG

GATTCTAAAGAAATTCAACTTGGACGGGATCTACGGTGGGGCTGAGAAGGTGACGGGTGAGGAGTACCATGTGGAGGTTG

ACGGGCAGCCGGGTGCGTTCCGCTGCTACCTGGACGTGGGTCTGGCCCGGACGACAACCGGGGCGAGGGTCTTTGGCGCC

CTG---GTTAACCCTCTGTTCGAGAAGAAGTCGAAGAATTTTGGAATTGGACAAGATATCCAGCCAAAGCGTGATCTGTC

ACGTTTTGTCCGCTGGCCAAAGAATGTGCGTCTGCAACGTAAGGCTATTCTCTACAAGCGTCTCAAAGTTCCTCCATCAA

TCAACCAGTTCACTCAAGCCCTGGATAGGCAGACAGCCACCCAGTTGTTCCGTCTGATGGAAAAGTATCGTCCAGAGACG

TACCAAGAGAAGATGAGGCGACTGAAGTCTCGTGCCGAGGAGCGCGTCAAGGGCAAGGAAGATGTTCCGACGAAGCGTCC

ACCGGTCATCAGGTCCGGCATCAACACCATCACGTCTCTGATTGAACAGAAGAAAGCACAGCTGGTTGCCATTGCCAACG

ACGTGGAACCGATCGAGATCGTGTTGTTCCTGCCTGCTCTGTGCAGGAAGATGGGTGTTCCTTATTGCATTGTCAAGAAC

AAGTCTCGTCTCGGCCGAGTTGTTCACCGCAAAACGGTCACGAGCTTGGCTTTCACACAGGTTAACGAGGACAAAACCGC

ACTGACCAAGTTGGTTGAAGCTGTGAAGACCAATTTCAATGAACGTGGTGAAGAGATCCCGCAGCACTGGGGTGGTGGTG

TCATGGGCCAGAAGTCTCAGGCTCGC---ATGGGTCGAGTTAATATAAAGGCCCACACAAAAAACCGCAAGGGTGCTGCC

CTGCGAGCTGTTGACTTTGCTGAACGCCATGGCTTCATTAAAGGTGTTGTGAAGGATATTGTCCATGATCCAGGTCGTGG

TGCTCCATTGGCCAAAGTTGTTTTCCGTGACCCATACCGTTATAAGCTGAGGACAGAGACGTTCTACTGTGGAAAGAAGG

CCAACCTCCAGGTCGGAAATGTTCTGCCAATTGGGTCCATGCCAGAAGGTACTGTTATCTGCTGCGTTGAAGAGAAGACC

GGAGATCGTGGTCGTCTTGCACGCACGTCTGGCAACTACACAACTGTTATTGCACATAACGTTGAAACCAAGAAAACAAA

AATTAAGTTGCCGTCTGGCACCAAGACAACTATTCCATCAAACAACCGCGCAATGGTTGGTATTGTTGCCGGTGGTGGTC

GTATTGACAAGCCAATGCTGAAGGCTGGCAGAGCCTACCACAAGTACAAGGCCAAGAGAAACTGCTGGCCTAAAGTCCGT

GGTGTTGCCATGAACCCTGTCGAGCATCCCCATGGTGGTGGTAATCACCAGCATATTGGTAAAGCATCGACTGTTGCTCG

A---TCTGGTGACAGGCTGACTCGTGCTGCCAAGGTCCTTGAACAACTGACTGGCCAGACGCCAGTCTACTCCAAGGCTC

GCTTCACTGTGAGGTCGTTCGGCATCAGAAGAAATGAAAAGATTGCTGTTCACTGCACAGTCCGAGGCGCCAAGGCAGAG

GAGATCCTTGATAAGGGTCTGAAAGTTCGTGAGTATGAACTGAAGAGGGGCAACTTCAGTTGC---TCGTTCAGACGATT

CGTTGAAATTGGGAGAGTTGCCCGTGCGGTGTATGGACCAGACCAGGGCAAGCTTGTAGCCATTGTGGACGTAATTGACC

AGAACAGGGCTTTGGTTGATGGTCCCTGTACACATGTTGCTAGAAAGTCAATGAACTTCAAGGAACTGGAGCTGACTAAT

CTGAAGGCCAAGTTTCCCCACTCAGCCAAGACCGGAGTTGTGAAGAAGGCGTGGGAGAAAGATGAAATCTCCAAGAAATG

GGAAGAGAGCCACTTGGCCAAGAAGATCGAAAGGTTTAAACTGATG---TACAAGTATATACAGGAGATGTTCCGTAAGA

AGCAGAGCGATGGTATGCGCTTCCTGCTGCGCATCCGTTGCTGGAATTTCCGTCAGCTTGCTGCGGTTCACCGGGCTTCT

CGTCCAACTCGCCCAGATAAAGCCAGGAGACTGGGGTACAAAGCCAAACAAGGATTTGTGGTTTACCGTGTCCGTATTCG

TCGTGGTGGTCGCAAGAGGCCAGTTGCAAAGGGACAGGTCTATGGAAAGCCAAAGAGCTGTGGAGTTAACCAACTGAAGA

ACCAACGTTCTCTGCAGGCCGTTGCTGAGGAACGTGTTGGACGTCGTTGCAAGGCGCTGCGTGTGCTGAACAGCTACTGG

GTGGCTGAAGACTCGACGTACAAGTACTACGAGGTTATCTGCGTGGATCCGTTCCACAAGGCCATTCGTCGTGATCCAGA

AATCCAATGGATCTGCAAACCAGTCGCTAAGCACCGTGAAATGCGCGGTCTCACGTCGGCCAATCGCAAGTCCCGTGGTC

TTGGAAAGGGTCACGGCTACAGCAAGACGATCGGCGGATCACGCCGCGCTAACTGGAGGCGCACCAACACCGTGCAAATG

CACCGAAAGAGA---CGATTCTTGGGTAGAAGAACTGGCTCGAAGTTCAACAAAGTTGTTCTTAAGCGACTGTTTATGAG

CAAGACAAACCGGCCTCCTCTTTCTCTGTCACGTTTGCGGCACATGAAGAAGACTGGACGTGAGGGAAAGATCGCTGTTG

TCGTTGGCACAGTCACTGACGATCCTAGGATCTTTGAAATTCCGAAGTTGAATGTGTGTGCTCTGCATGTCACCGATCGC

GCACGGGCTCGCATTCTGAAAAGCGGAGGTCAGATCATTACATTTGATCAGTTGGCTTTGAAGAGTCCAAAGGGCCAGAA

GAGTGTCCTCCTACAAGGACCACGTAAGGCCAGAAAAGCATACAGACACTTTGGAGCTGCTCCAGGAGTTCCACACAGCC

ACACTAAGCCATAT---AAGGAGTACAGAGTGCTCGGGCGTATGATGCCCACGAGCAAGGTGCGGGTGCAGCCGATCTAT

CAGATGAGAATCTTCGCCCCCGACCGTCTGCAGGCAAAGTCTCGCTTCTGGTACTTTGCGTCTATGTTGAGGAAGGTGAA

AAAGACGCAGGGAGAGATTCTTTGCTGCAGCCAAGTTAATGAGAAGAAGCCGACGGCAATCAAGAACTTTGGCATCTGGC

TGCGCTATGACTCTCGCAGTGGGTCACACAACATGTATCGCGAGTACCGCGACCTGACCACATCTGGAGCAGTGACACAG

TGCCGTGACATGGGAGCACGTCACCGTGCCAGAGCCAGTTCCATTCAGATCATGAAGGTTCAGGTCATTGCTGCTAGCAA

GGTCAAGAGCAAGAATCTGAAGCAGTTCCTGGATAGCAAGATCCAGTTTCCTCTGCCACACAGAGTGAACCGCCTGCATC

ATCCACGCTTCACCACTGCCCGGCCACACACG---ATGAGGGCTCAAAAACGCCTAGCCGCGGCGGTTATGAAAACCGGA

ACAAACAAAATCTGGTTGGATCCAAATGAAATCAGCGAAATTGCAAATGCCAACTCCAGGCAGAACATTCGCAAATTGAT

CAAGAACGGTCTTATCATCCGCAAGCCAGAGGCCGTGCACTCGCGAGCGCGCGTCCGCAAGAACCAGCTGGCACGTCGCA

AGGGTCGCCACACCGGCACTGGTAAGAGGAAGGGTACGGCTAACGCCCGTATGCCACAGAAGGTGATCTGGGTTCGGCGC

CAGCGTGTCCTCAGAAGTCTTCTGAAGGCCTACAGAGACGATAAGAAGATTGACAAGCATCTTTACCATGCGCTGTACAT

GAAGTGCAAGGGAAACGTCTTCAAGAACAAGCGTATTCTGATGGATTACATCCACAAGAAGAAGGCCGAGAACGCCCGTT

CCAAGATGCTG---GATCGATATGTTAAGTTATGGAGAAAGCCGAAGGGTATCGACAATCGCGTACGACGTCGCTACAAG

GGTCAGTACTTGATGCCGAACATCGGTTATGGTAGCAACAAGAAGAGCAAGTACGTCTGCCCTGACGGTCTCATCAAGGT

TCTCGTCCACAACATCAAGGACCTCGAAATGCTGCTCATGCAAAACCGAACGTATTCGGCCGAGATCGGTCACGCCGTTG

GCAGCAAGAAGCGCAAGGAGATTGTTGAGCGTGCCGAGCAGCTCGGTATCAAGGTGACGAACGCCAACGCTCGTCTG---

CAGTTGCGTGTGGCGAAAGTCACGGGTGGAGCTGCAGCAAAACTGTCCAAAATCGTTCGCAAGTCCATTGCTCGAGTTTT

GACTGTGATGAGCCAGGCTCAGAAGGAAAACCTGCGGAAATTCTACCAAAACAAAAAATACAAGCCTAAGGATCTTCGCC

CAAAGAAGACCCGAGCCTTGAGACGCGCCCTGACGGAGCACGAGCTGTCCATCAAGTCTGTTAAGTTCGTCAGAAAGCAG

CGTGCATTCCCCATGAGGAAGTTTGCAGTCAAA---TCGCATCAGATGCAGAAGATCAGAATCACTCTGCGTGGAGCAGT

TGTGCTGATGGGCAAGAACACGATGATGCGAAAGGCAATTAGAGGTCACATGGAAAACAACCCAGCCCTTGAAAAACTGC

TTCCTTTCATCAAGGGAAACGTTGGCCTGGTCTTCACGAAGGGAGAACTGTCTGAAGTGCGTAAGATCATTCAGGAGAAC

AAGGTAGCGGCCCCAGCGAAAGCCGGAGTCATTGCTCCTCTTGATGTGACGCTGCCAGCCCAGAACACTTCCCTTGGACC

TGAGAAGACTTCCTTCTTCCAGGCGTTGGCTATTCAGACTAAGATCTCAAAGGGAACTATTGAAATTCTGAACGATGTGA

AGCTGATCAAGGTTGGAGAGAAAGTTGGAGCGTCCGAGGCGACTCTTCTCAACATGTTGAACATCTCACCCTTCACATAT

GGACTACAGATTCAGGTGTACGACAGCGGCACGGTCTTTGATCTGTCCATTCTCGACATCACTGATGATGACATCAGAGC

CAGATTCCTTGAGGGTGTAAGCAACATCACGAGCCTGTCTCTC---ACTCGTGCTGGACAGAGAACTAGGTTCAAGGCTT

TTGTGGCCATTGGAGACTTCAACGGCCACGTTGGTCTTGGTGTGAAATGTTCCAAGGAAGTGGCCACCGCCATCCGTGGA

GCGATCACTCTGGCCAAGCTGTCCATTGTTCCGGTGAGGCGAGGCTACTGGGGTAACAAGATCGGTAAGCCTCACACCGT

GCCGTGCAAGGTGACTGGACGTTGTGGCAGCGTACTCGTGCGTCTGATTCCTGCTCCCAGAGGAACGGGAATCGTTAGTG

CACCTGTTCCCAAGAAGCTGCTGCATATGGCTGAGATTGATGACTGCTACACTTCGGCCAGTGGACAGACGGCTACCCTT

---TTGGCCGAGGATGGATACAGTGGTGTCGAGGTCCGAGTCACTCCAACCCGAACCGAGATCATCATTCTAGCCACTCG

CACCCAGAATGTGCTTGGTGAGAAGGGTCGTCGCATCAGAGAACTAACGGCCGTCGTCCAGAAGAGGTTCAACTTCCCAG

AAGGCACCGTCGAATTGTATGCTGAGAAGGTTGCTACGCGAGGACTGTGTGCCATTGCGCAGGCCGAGTCTCTTAGGTAC

AAGCTCATCGGAGGCCTTGCAGTCAGAAGGGCTTGCTATGGTGTCCTGCGATTCATCATGGAGAGCGGAGCGAAGGGATG

CGAGATTATCGTGTCTGGCAAGCTGCGAGGACAGAGAGCCAAGTCAATGAAGTTCTTGGATGGTCTGATGATCCACTCTG

GTGATCCAATGAATGATTTTGTTGACCAGGCGGTCAGGCATGTCCTCCTCAGACAAGGAGTGTTGGGCATCAAAATTAAG

ATCATGCTTCCGTGGGATCCAACTGGAAAGATTGGTCCAAAGAAGCCGCTGCCCGATCACGTTAGCATCCTGGATCCAAA

AGATCAG---GGTCCGAAGAAGCACCTGAAGCGCGTCAATGCGCCCAAACATTGGATGCTGGACAAGCTGGGCGGTGTCT

TTGCACCCCGTCCAAGCTGCGGTCCACATAAATTGCGAGAATGTCTGCCGTTGTCGATCTTCCTAAGGAACCGGTTGAAA

TACGCGCTGACGTACGATGAGGCGAAGAGGATCCTCAACCAGCGGCTGATCAAGGTCGATGGCAAAATTAGGAGCGACAA

GACCTATCCAGCTGGATTCATGGACGTCGTGAGTATTGAGAAGACGAACGAGAACTTCAGGATCATCTACGACGTCAAGG

GTCGCTTCGCCATCCACCGCATCACGGCCGAGGAAGCGAAGTACAAGATTTGCATGGTAACTGGAGGTCACAACTTGGGA

CGTGTTGGTCTCGTGACACACAGGGAGCGTCACCCGGGGTCCTTCGACATTGTCCACATCAAGGATTCCCTCGGCCACAC

CTTTGCCACCAGGTTGAAACAATGTGTCATCATTGGCAAGGGAAACAAGCCATGGATCTCTCTTCCAAGAGGCAAGGGAG

TCCGTCTGACCATTGCAGAAGAAAGAGATCGTCGCATCCAGTCA---TTGAATATTTCCTTCCCGGCGACGGGCTGCCAG

AAGCTCATTGAAGTTGACGATGAACGCAAGCTTCGAGCCTTTTACGAGAAACGTATGGCTGCCGAAGTTTCAGCGGAGAG

CCTTGGAGATGAATGGAAGGGTTACGTTGTGCGCATCTCGGGAGGTAATGACAAGCAGGGATTCCCAATGAAGCAGGGAG

TCATGACAAACGGACGTGTTCGTCTCCTGCTGTGTGACGGCCACTCGTGCTACCGCATGCGCCGTGATGGAGAAAGACGT

CGCAAGTCTGTCCGAGGATGCATTGTTGATGCTAATCTCAGCGTCCTCAACCTTGTTATCCTGAAAAAAGGTGAACAGGA

AATCCCCGGCCTAACAGATACCACTGTGCCCCGCCGTCTCGGACCTAAGAAAGCCAGCAACATCCGAAAGCTGTTCAATC

TGCAGAAGGCGGATGATGTGCGCCAGTACGTCGTTAAGAAGCCGCAGACCAAGGCACCAAAGATCCAGCGACTCGTGACC

CCGGTTGTGCTT---ATCTCTCGTGATAAATGGCACAAGCGCAGGAAGACCGGAGGTCGTATGGCCCAGGTCCGAAAGAA

GCGAAAGTTCGAGCTTGGTCGCCCAGCTGCCAGCACTAAGATCACGATTCGGACGAGAGGTGGGAACAAGAAGTACCGCG

CCATGAGACTCGACACGGGCAACTTCTCGTCAGAGGGTGTTTCACGCAAAACGCGTATCATGGACGTTGTGTACAACGCG

TCAAACAACGAGCTTGTGAGAACGAAGACGCTTGTGAAGAACGCGATCGTTGTGATTGATGCAGTTCCATTCAGAAACTG

GTACGAGGCTCACTACGCCCTACCTCTCAGCAGGAAGAAGGGCGCTAAGGCCTGTGTTGCCTCTCGTCCGGGACAGTGTG

GGCGTTGCGATGGTTACATCCTGGAGGGCAAAGAGCTCGAATTCTACGCAAGAAAGATCAAGGCTAAGAAG---AAGACC

TACTCGACTCCCCGTCGTCCCTTCGAAAAGGAGCGATTGGACCAGGAGTCTAAGCTTATTGGTGAATTTGGTCTGCGCAA

CAAGAGAGAGGTATGGCGAGTCAAGTACACCTTGACCAAGATCCGTACAGCTGCTCGTGAGCTGCTTACCCTCGATGAGA

AAGATCCAAGACGTCTGTTTGAAGGTAATGCGCTGCTGCGTCGCCTGGTCCGTATTGGAGTGCTGGACGAGCAGAAGATG

AAGCTCGATTACGTGCTCGGTCTGCGACTGGAGGACTTTCTGGAGCGTCGCCTTCAGACGCAGGTCCTCAAGCTGGGATT

GGCGAAGAGCATTCACCATGCTCGCGTTCTGATTCGTCAGAGGCACATCAGAGTGCGTAAGCAAGTCGTGAACCTGCCAT

CCTTCATTGTGCGACTTGATTCCCAGAAGCACATTGACTTCTCCCTGAAGTCGCCATACGGAGGTGGC---TTCCAGAAG

CAGCCAACGGTCTTCTTGAATAAAAAGAGACATGTGAAAAGCATTGGTCTCGGCTTCAAGACCCCACGTGATGGCCATTA

CATCGACAAGAAATGTCCGTTTGCCGGCGCCGTGTCGATTCGCGGTCGCATCTTGACTGGCATCGTTCTGAAGAGGAAGA

TGCAGGGAACCATCGTCATCCGACGTGACTACCTGCACTACGTGCCGAAGTACGAGCGCTTTGAGAAGCGCCACAAAAAC

CTTAGCGTCCATCTCAGTCCATGCTTCAGAGATGTTCAGGCAGGTGATATCGTGACCGTGGGCGAGTGCCGACCTCTGAG

CAAGACTGTA---GGTATTTCCCAATCCGCTCTTCCGTACAGACGAAGTGTGCCAACTTGGCTGAAGCTTGGACCTGATG

ATGTTAAGGAACAGATTTATAAGCTGGCGAAGAAGGGCCTCACCCCATCGCAAATTGGTGTTATCCTTCGAGACTCGCAC

GGTGTCGCACAAACCCGTCATGTGGCCGGTAACAAGATCTTGCGTATCCTGAAGGCGAAAGGTCTCGCTCCGACCATCCC

AGAGGATCTTTACTTCCTCATCAAGAAAGCTGTTGCCATCCGCAAGCATCTGGAACGTAACAGGAAGGACAAGGATTCCA

AGTTCCGCCTGATTCTGGTTGAAAGCCGAATCCACAGACTTGCTCGTTACTACAAGACGAGGAAAGTGCTGCCACCTGTC

TGGAAA---ATGGTGCGTATGAATGCTTTGGCCGATGCCCTTAAGTCGATCTGCAATGCAGAGAAGCGAGGAAAACGTCA

GGTCCTGATCCGACCGTGCTCAAAGGTCACAGTAAAGTTCCTGACCGTCATGATGAAGCATGGTTACATCGAGGATTTTG

AGATCATTGATGACCACCGTGCCGGTAAAATTGTCATAAACTTGACCGGCCGAATCAATAAGTGCGGTGTGATCAGTCCA

CGTTACGATGTCAAGGTTAAAAACATTGAATCATGGACAACGTCTCTACTGCCATCACGTCAATTTGGATTCCTGGTGAT

GACCACATCTGGAGGAATTATGGATCACGAAGAAGCCCGAAGGAAGCATCTTGGAGGAAAGATCCTTGGCTTTTTCTTC-

--GAGGCAACTGTTCATCGCATCCGTATTACGCTGACAAGCCGTAATGTCAAGAGCCTCGAAAAGGTTTGCGCTGACCTT

ATCCGTGGTGCCAAGGACAAGCAGCTGAAGGTGAAGGGACCAATGAGAATGCCAACGAAGACTCTTCGCATCACCACCAG

AAAGACTCCGTGCGGTGAGGGTTCGAAGACGTGGGATCGCTTTCAGATGAGAATCCACAAGCGACTCATCGATCTGCACA

GCCCGTCGGAGATTGTCAAGCAGATTACCTCCATCAGCATTGAGCCTGGAGTGGAAGTTGAAGTCACAATTGCTGAT---

ACAATTAGAACGCGTAAATTTCTGACAAATCGACTGCTTTGCAGAAAGCAGATGGTTGTCGATGTTTTGCATCCAGGCAG

GGCAACTGTTCCAAAGACAGAAATCAGGGAGAAGCTGGCCCGACTCTACAAGACGACTGCTGATGTTGTCTTCTGTTTTG

GATTCAAAACGCAGTTTGGAGGTGGAAAGACAACTGGCTTTGCATTAATCTACGACACTCTGGATTATGCGAAAAAGTTT

GAGCCAAAGTACCGCCTGGCTAGA---GGTAAGCCACGAGGTCTTAGGACTGCTCGCAAGCTGAAGAACCATCGTCGTGA

GCAGAGATGGCATGACAAAGACTACAAGAAGTCTCACTTGGGAACCCGCTGGAATCCTTTTGGTGGAGCCTCCCATGCCA

AGGGAATCGTCCTGGAAAAAGTTGGAGTTGAAGCCAAGCAGCCGAACTCGGCCATCCGTAAGTGCGTAAGGGTGCAGCTG

ATCAAGAACGGAAAGAAGATCACTGCGTTCGTTCCAAGGGATGGTTGTCTCAACTTCATTGAGGAGAACGATGAAGTCTT

GGTAGCTGGATTTGGTCGTAAGGGTCACGCCGTCGGTGATATCCCCGGAGTCCGTTTCAAGGTCGTCAAGGTCGCCAGCG

TTTCCCTGCTTGCCTTGTACAAACAGAAGAAGGAGAGGCCAAGGTCGATG---CGCCAGGCACGCAAGCACAACAACTTC

TACGTTCCACAGGCAGCTAAGGTGGCTTTCGTCATTAGGATCAGAGGTATCAATGGAATCGCTCCAAAGCCGCGCAAAGT

TCTGCAACTTCTTCGCCTGCGTCAGATCAACAACGCTGTCTTCATCAAGCTGAATAAGGCGACAATCAACATGCTGCGCT

TGGCTGAACCATACATCGCTTGGGGATACCCCAACCTGAGTATCACAAAGAAACTTCTGTACAAGCGAGGTTTCGCGAAG

GTTGAAAGATTGCCGCTGTCCAACGAAATCATCGAAAGAAAGCTGCTGATCTGTGTGGAGGACGTTATTCATGAGCTTCT

GACGTGTGGACCGCTCTTCCAGAAGGCAAGCAACATGCTGTGGCCATTCAAGCTATCGAGTCCGAACGGTGGATGGAGGA

AGAAGGGACGTCACTACGTCGATGGCGGTGACTTTGGAAACCGT---CAGGAGAAGAAGAGGAAGTTCACAGAAAGTGTT

GAACTTCAGATTTCCTTGAAGAACTATGATACCCAAAAGGACAAGCGTTTCAGCGGAACCGTCAAGTTGCGTCACGTCCC

ACGGCCCAAGATGTCTGTCTGTGTACTCGGAGATCAGCAGCACTGTGACGAGGCGAAGGCGAACAGTATTCCATGCATGG

ATCAGGACGCTTTGAAGAAGCTGAACAAGCAGAAGAAGCCCATCAAGAAGCTTGCGAAGCAGTACGATGCTTTCCTTGCT

TCGGATTCTCTCATCAAGCAGATCCCGCGTATCGTTGGTCCGGGTCTGAACAAGGCCGGCAAGTTTCCGTCTATGTTGAC

GCACAACGAATCCATGGTTGCGAAAATCGAGGATGTCCGTGCCACCATCAAGTTCCAGATGAAGAAGGTTCTTTGTCTGG

CTGTTGCTGTTGGTCATGTGGCCATGACTCCGGAGGAACTGGCGACGAACATCACGATCGCAATCAACTTCCTCGTGTCT

CTGCTGAAGAAGAACTGGCAGAACGTGCGAGCGCTGTACATCAAGAGCACCATGGGTCCATCCCAGCGCCTTTAC---GG

CCATTTCCACAAGGATTGGCAGACGCATGTGAAGACATGGTTCAACCAGCCAGCCCGCAAGGAGCGCCGAAGGGTCGCCC

GACGCAAGAAGGCCCTTGCCATCGCTCCTCGGCCCGCTGCAGGACTGAGACCGCAAGTACGATGCCAGACTTTCAAATAC

AACACTCGTCTGCGTGAAGGAAGAGGCTTCAGTCTCGACGAACTGAAGGCAGGCATCAACAAGAAGGAGGCTCGCACGAT

TGGAATCTCCGTTGATGTCCGTCGTAGGAACAAGTCCGTGGAGTCGCTCCAGTTGAATGTCCAGCGCCTGAAGGAATATC

GCAACAAGTTGATCCTCTTCAAGAAGCTGAGCAAGCCA---ATGACTCGTTACTCCCTCCCACCCGAGAACCCAGCCAAA

TCGGCTTTGGCTCGTGGCTCACACCTTCGCGTTCACTTCAAGAACACGCGTGAGGCTGCCCAGGTGATCAAGCACATGCA

TCTGCGCCGTGCCGTTGCATTCCTAAAGAATGTGATCGCACACAAGGAATGCGTACCGTACCGTCGCTTCACTGGTGGCG

TTGGAAGGTGCGCTCAGGCCAAGCAGTGGGGAGCCACACAGGGACGTTGGCCAAAGAAATCTGCGGAGTTTCTTCTTCAG

ATGCTGAAGAATGCAGAGAGCAATGCAGAATTCAAGGGATTGGACACGGACCATCTGGTTATTGACCACATCCAGGTCAA

CCGTGCCCCAAAGATGAGAAGGCGTACCTACCGTGCTCACGGACGCATCACATACATGAGCAGTCCTTGCCACATAGAGA

TCATCCTG---ATGAAGTTCAACAAACTAGTAAGTTCGTCCAGCCGCAAGAACCGGCAACGGCACTTCAGCGCACCATCT

CATATCCGTAGAAAGATTATGAGCGCGCCTCTCTCTAAAGAACTGCGCCAGAAGTACAACGTCCGTAGCATCCCAATCCG

CAAAGATGACGAAGTACAGGTAGTTCGTGGTCACCACAAAGGTCAGCAGGGAAAGGTCGTTCAGGTGTACAGGAAGAAGT

ACGTGATCCACATTGAGCGAATTCAGCGTGAGAAGGTTAACGGAGCTACCAGCCACGTTGGCATCCATCCTTCAAAGGTT

GTCATTGTGAAGCTCAAGATGGACAAGGATCGCAAGGCCATTCTTGAACGCAAGGCTGCGTCAAAGGGAAAG---GACGG

AAAAGTTGTGCTTGTCCTCGGTGGACGTTATGCAGGCCGCAAGGCTATCATCGTAAAGAATTACGATGATGGTTCCACGG

ATAAGCCATACGGCCACGCACTCGTTGCCGGTATCTCCAGATACCCGCGCGGCGTCACGAAGAAAATGGGCAAGAAGAAG

ATCAAGCAGAGGTCCAAGGTCAAGGCCTTCATCAAGGTCTACAACTATAACCATCTCCTGCCAACCAGGTATTCCGTTGA

TGTTCGATACAAATCCGGAAAGAACAGATGGTTCTTCCAGAAACTGCGATTC---TTGAGGGGTCATGTCAGCCATGGAC

ATGGTCGTATTGGCAAACACCGTAAGCATCCCGGAGGTCGTGGTAATGCCGGCGGTCAGCATCATCACCGAATCAACTTC

GATAAATACCATCCTGGTTACTTCGGAAAGGTCGGTATGCGTCATTTCCATCTGACTCAGCAAAAGTACTTCTGCCCCAC

GGTCAATCTCGACAAGCTGTGGACCCTCGTCTCTGAACAGACGCGGCTCAAAGCTCCGGTCATCGATGTTGTCCGAGCTG

GATACTACAAAGTTCTTGGCAAGGGTCACCTGCCAAAGCAGCCGGCGATCGTCAAGGCCAAGTTCTTCAGCCGTTCGGCC

GAGGAGAAGATCAAGGCCGCTGGTGGAGTCTGCGTCCTGGTTGCT---ATGGCGGCTCACAAAACATTCAGAATTAAAAT

GAAGTTGGCGAAAAAGATGAAGCAAAATCGTCCAGTTCCACAGTGGGTTCGGATGAAGACCGGAAATACAATCAGGTACA

ACTCGAAAAGACGTCACTGGAAGCGTACTAAGCTGAAGTTG---ATGGCCCGTACCAAGCAAACGGCAAGAAAGAGCACC

GGTGGCAAGGCTCCAAGGAAGCAACTCGCATCGAAGGCTGCACGAAAGTCAGCTCCCGTTTCTGGAGGTTTGAAGAAGCC

GCATCGCTACAAGCCCGGAACTGTCGCTCTCCGTGAAATCAGGCGCTATCAGAAGTCTACGGATCTGCTCATCCGCAAGT

TGCCATTCCAACGGCTGGTGCGTGAAATTGCAAATCAATTCAAAGCTGATCTTCGTTTCCAGTCATCTGCTGTGCTGGCG

CTCCAAGAAGCATCTGAAGCTTACCTGGTTGGACTGTTCGAAGATACCAACTTGTGCGCGATTCATGCCAAGAGAGTCAC

CATCATGCCTAAGGATATTCAACTGGCCCGCCGTATTCGTGGGGAACGTCAG---CTCGCTTGTACCTATGGTGCTTTGA

TCCTCGCCGACGATGAGATTCCGATTACTGCGGAAAAGCTGACCACAATCCTCAAGGCTGCAAACGTCCAGGTTGAGCCC

ATCTGGCCG---ATGCGTTACGTGGCCGCATACCTGTTAGCCGTCCTTGGTGGCAATGAACATCCAGGGGAAGCGGATGT

GAAGAAGATCCTGAGCAGCGTTGGAATCGATGCGGATAACGAATGTGTGAAGAAGGTCGTCGGGGAACTGAAGGGAAAGA

ACCTGGAAGAACTGATCGCTGCTGGT---GACAAGGAATGGGTTCCAGTGACCAAACTGGGTCGTCTCGTGAAAGACCAG

AAAATCAAGACAATTGAGGAAATCTACTTGTTTTCCCTGCCTATCAAGGAGGCTGAGATCATCGATTACCTTACAACGCT

GAAGGATGAAGTGTTGAAGATCATGCCAGTCCAGAAGCAG---AAGTACTTGCCGCACTCTGCTGGTCGTTACCAGGCGA

AGAGATTTAGGAAGGCACAGTGTCCGATCGTTGAACGTCTTACGGACAGTCTTATGATGCACGGCCGCAACAACGGCAAG

AAGCTCATGTCCATGCGTATAGTTAAGCACAGTTTTGAGATCATCCATCTTCTGACAGGAGAGAACCCGCTCCAGATCTT

GGTAAATGCCATCATCAACAGCGGTCCAAGAGAAGATTCCACCCGTATTGGTCGTGCTGGAACGGGAGCGAGAGAAGCGT

CCTTCCGTAACATCAAGAGCATTGCTGAATGCCTTGCTGATGAGCTGATCAATGCCTCCAAGGGTTCATCCAATTCCTAT

GCGATCAAGAAGAAGGATGAG---AGTGCTAAGATTCAGAAGCCGGAAGGCGAAAAGGTAGATGAGTTGGAGAGTAGCAT

CTCTCAGGCTCTTCTTGAGCTGGAGATGAACAGTGACTTGAAGGCTCAGCTCAGAGAACTTGTTTTCACTGGAGCAAAGG

AAGTTGACGTGCAGAACCGAAAGGCCATCATCGTCTACGTTCCAGTTCCCCAGCTGAGAGCGTTCCAGAAGATCCAGCCT

CGTCTCGTGCGTGAAATGGAGAAGAAGTTCTCTGGAAAGCATGTCGTCATTATTGCACAGAGGCGCATTCTGCCCAAGCC

AACAAGGAAGAGCACGAAGAACAAGCAAAAGCGTCCAAGGAGCCGTACTCTGACCCACGTTCATGATGCCATCCTAGAGG

ACCTTTGCTTTCCATCAGAAATCGTTGGAAAACGCATTCGGGTTAAGTTGGACGGAACAAGGTTGATCAAAGTTCACCTA

GACAAGAGTCAGCAGACGAATGTTGAGCACAAGTTGGACACCTTTGCCGCTGTCTACAAGAAACTCACTGGAAAGGACGT

GACCTTTGAGTTCCCAGAGTATGTCTTG---ATGTCTAAAAAGAATCGTGTGATGATTTACGAATACCTCTTCAAGGAGG

GAGTTCTGGTTGCTAAGAAAGACTTTCACCTCCCAAAACATGGTGAAATCGATGTGCCTAACCTGCACGTCATTAGGGCC

ATGCAGTCGCTCGTTTCCCGCGGCTATGTGAAGGAGCAATTCTGCTGGCGCCATTACTACTGGTATCTGCAGAACGAAGG

CATCCAGTATCTGCGTGACTTCCTTCACCTGCCTCCAGAGATTGTCCCAGCAACACTCAAG---CATGTGACCGATCTGT

CTGGAAAGGAAACGATTGTGCGAGTGACGGGTGGAATGAAGGTGAAGGCGGATCGTGATGAGTCGTCTCCATACGCTGCT

ATGTTGGCCGCTCAGGACGTCGCTGAACGATGCAAGATAATCGGTATAACAGCGCTGCACATCAAGGTGCGCGCCACAGG

TGGAAACAGGACTAAGACTCCAGGCCCTGGAGCTCAGTCTGCCCTGCGTGCGTTGGCTCGTGCTGGCATGAAGATCGGTC

GTATCGAGGATGTG---AAGAAGAAGAAGAGGACGTTCAGGAAGTACACCTACCGTGGTGTCGACCTCGATCGACTCTTG

GATATGAGCAATGACTCTCTCATGCTTTTGTTTCCTTGCCGAGTCCGGCGACGATTTGCGCGAGGCCTGAAGCGTAAGCA

CATGGCGCTTCTGAAGAGACTGAGGAAGGCGAAGAAGGAGGCCCCAGCACTTGAGAAGCCAGAGTGCATCAAGACTCACC

TGCGGAACATGATCATCATCCCGGAAATGATCGGATCCATCATTGGTATCTACAATGGAAAAACATTCAACCAGGTGGAA

ATCAAGCCTGAAATGATTGGACACTACTTGGGAGAGTTCAGCATCACGTACAAACCAGTGAAGCACGGCCGACCTGGTAT

TGGTGCCACCCACTCTTCCAGGTTCATTCCGCTCAAG---ATCATCGAGAAGTATTACCCACGTCTTACTCTCGATTTCC

ATGTCAACAAAAGAATATGCGAGGAAATCGCTATTATTCCAAGCAAAAAGCTGAGGAACAAGATTGCTGGATTTGTGACC

CATCTCATGGGCCGCATTCAGAAGGGACCCGTCAGAGGCATCTCCATCAAGCTTCAAGAAGAGGAACGTGAGAGGCGAGA

CAACTTTGTGCCAGAGAAATCGGCGATTGAAGAAGATATCATCGAAATCGATGCAGATACTAAGGAGATGTTGAAGGCA-

--CCGCGTAAGTGCTCATGCAGCAACCGCATCATTTCAGCCAAAGATCATGCATCAATCCAGATCAATCTTGCAGAGGTT

GATCCATCAACTGGTCGCATGACGGGTCAGAACAGGACGTACGCCATCTGTGGAGACATTCGCCGAATGGGTGAATCAGA

TGATTGCATCATTCATCTGGCAAAAAAAGACAGCATGGTTCCACAG---CACGTGAAGCCGGTGCGCTGCACGAACTGCG

CTCGCTGCGTTCCGAAGGACAAGGCCATCAAGAAGTTCGTGATACGTAACATCGTGGAGGCTGCGGCCGTGAGGGATCTG

GCTGAGGCGTCGGTCTACCAAGCCTATGTTCTGCCGAAGCTGTACGCGAAGCTGCACTACTGCGTGAGCTGCGCCATTCA

CAGCAAAGTTGTGCGCAACAGATCGAAGGAGGCTCGCAAGGATCGCAGTCCACCTCCGCGATTCAGGCCA---ATGATAT

TTGTCAAGACCTTAACGGGCAAGACCATCACTCTTGAGGTCGTCAAGGCCAAGATTCAGGAAGGCATTCCCCCTGATCAA

CAGCGACTGATCTTTGCTGGTAAGCAACTGGAAGATGGAGACATTCAGAAGGAGTCAACTCTTCACCTAGGTGGAGCGAA

GAAGCGCAAGAAGAAGAACTACACTACTCCAAAGAAGAACAAGCACCGCAAGAAGAAGGTCAAGCTGCCAGTCCTGAAAT

ACTACAAGGTTGACGACAATGGAAAGATTACTCGTCTTCGCCGCGAGTGTCCGAACGACGAGTGTGGTGCCGGTGTATTC

ATGGCATCGCATTTCGATCGCCAGTACTGCGGCAAGTGTGGATTGACGTACGTCTTCAACAAG---AAGCCGTACCCTAA

GTCTCGTTTCTGTCGAGGTGTTCCAGATGCAAAGATTCGTATCTTTGATTTGGGAAGAAAGAGGGCGAAGGTGGACGAGT

TCCCGCTCTGCATTCATTTAGTGTCCGATGAGTTTGAGCAGATCTCATCAGAGGCGTTGGAAGCTGGACGTATCTGCGCC

AACAAGTACCTGGTGAAGCACTGTGGAAAGGATGCGTTTCATCTGCGTGTCAGGCTCCACCCGTTCCACATCATCCGCAT

CAACAAGATGTTGTCCTGTGCTGGAGCCGATAGGCTTCAGACTGGAATGAGAGGTGCTTTTGGTAAGCCTCAGGGAACAG

TTGCTCGCGTTGACATCGGACAGGTGATCATGTCGGTGCGTGCCAAGGACGGTCACAAAGAGAAGGTCATTGAGGCTCTG

CGACGTGCCAAGTTCAAGTACCCAGGAAGACAAAAGATCCACGTGAGCCGCAAGTGGGGATTCACG---AAACGCACAAA

GAAGGTTGGGATCGTTGGCAAGTACGGTACACGTTACGGTGCCAGTCTTCGTAAGGACATCAAGAAGATGGAAGTCACAC

AGCACAGCAAATACACATGCAGCTTCTGTGGCAAGGAGACAATGAAGAGGAAGGTCGTTGGCATCTGGTCGTGCAACAAA

TGCAAGAAGATTGTTGCTGGCGGTGCCTATGTTTACAGTACAAATGCCGCGGTTACGATTCGAAGCACAATCCGTCGTCT

GAAGGAGGCG---CACATTCAGAAGAAGACTTGTTCCCGATGCGGATATCCTGAAAAGCGCATGCGCAAGTACAATTGGA

GTGAGAAGGCCAAAAGGCGACGAACTCAGGGTGTTGGCCGTATGCAGCACTTAAAGAAGGTGTACCGTCGCTTCAGGAAT

GGATTCCGCGAGGGAACCGTC---AAGGGCAAAGATTCTGTCTTCGTCCAAGGTAAACGTAGATACGACAGAAAACAGTC

CGGTTATGGAGGTCAAACTAAGCCTATTTTCCACAAAAAGGCTAAAACCACAAAGAAGATTGTCTTGAAGATGGAATGCA

CTGAATGCAAGTACAAGAAGCAGCTCGCAATCAAGCGTTGCAAGCACTTTGAGCTGGGAGGAGACAAGAAGAAGAAG---

CTTACCAAGCGTGCTCGTTTTGTGCGCGACCTCGTACGCGAAGTGACTGGATTTGCTCCTTATGAGAAGAGAGTGCAGGA

ATTGCTGAAGGTCAGCAAAGACAAGAGAGCACTGAAGTTCTGCAAAAAGAGGCTCGGCAGAACTCTGCGTGCGAAGCGAA

AGAGGGAAGAGATGTCTGTCGTCTTGCAGAAGATGCGTAAAGCCCAG---AAGACAATAGAGAGCATCAACTCGAGGCTT

GCTCTGGTTATGAAAAGTGGCAAGTATGTGTTGGGCTATCAACAAACCCTAAAGACACTGCGCCAGGGAAAGGCAAAGCT

GGTTATAATTGCCAACAACACACCTCCCCTGAGAAAAAGTGAGATTGAGTACTATGCTATGCTGGCCAAGACCGGAGTGC

ATCACTACAATGGCAACAATCTTGAACTGGGAACGGGTTGTGGAAAGCTCTTCCGCGTGACAACACTGAGCATCACCGAC

CCAGGT---TGGAGGCTGAGAACCTTCAGCCAGCACAAGCGACACCTGAGGGAGTCCATCACCCCAGGAACCGTTCTGAT

TCTTCTGGCTGGAAAGCACAAGGGAAAGAGAGTTGTTTTCCTGAAGCAGCTTGCAACTGGTCTCCTTCTGGTAACAGGTC

CCTACCATCTGAATGGTTGCCCACTGCGAAGAATCAATGCCATTTACGCTATTGCGACCAAGACCAAGTTGGATGTCAGC

AGTGTTAAGCTTCCGGAGAGGGTTAACGACGCTTACTTCAAGCGAAAGGAAACCGAGGGAGAGATCTTTACCACTAAGAA

AGAGTACACCCCAAGTGCCGAGCGCAAAGAGGATCAGGACGTTGTCGACAAGCAACTCCTG---GTGACTGTCAAGGGAC

CACGTGGGACCCTGAGACGTGACTTCCGTCACATGGACATCAAAATGGTCAAGACCGTCTGCTCTCATGTTCAGAATATG

ATCAGAGGTGTCACTGGATTCCGCTACAGGATGAGGTCTGTGTATGCTCACTTTCCCATCAACGTTGCAATCTCCGAAAG

CAACACCGTCGTTGACATTCGTAACTTCCTGGGCGAGAAGTTCAACAGGCAGGTGCGAATGTTGCAAGGAGTGACATGCA

TTGCATCCAAGGACATGAAGGACGAGTTCGTTTTGGAAGGAAACGACATTGAACTCGTTTCTCGTTCAGCTGCCTTGATC

CAGCAGTCGACCACAGTG---GTCTGCCTTCGTGCCGTCGGTGGTGAAGTCGGTGCCACATCGGCTCTGGCTCCAAAGAT

CGGTCCTTTGGGTCTGAGCCCCAAGAAGGTCGGTGAAGATATTGCGAAAGCCTCGCAGGACTGGAAAGGTCTGCGCATTA

CTGTGCAGTTGACCATCCAGAATCGTCAGGCCACGATCGCTGTCATCCCATCGGCTTCCTCACTCATCATCAAGGCCCTG

AAAGAGCCGCCAAGGGACCGCAAGAAAGTTAAAAACATTAAGCACACTGGCAACCTGAGCCTGGATGAAGTCATTGCCAT

TGCCCGGACCATGAGGATACGCAGTATGGCGAAGGCGTTGTCTGGAACCGTAAAGGAAGTTCTTGGCACCTGCCAATCTG

TTGGATGCACGGTCGAGGGATCGCACCCT---GATGCGAAGGACCACCTGATGGGTCGTTTGGCCGCAACTGTGGCCAAG

ACCCTTCTGGAAGGTCAGCGCATTGTTGTTTTGAGATGTGAGGAAATTAACATCTCCGGTAACTTCTACCGAAACAAACA

AAAGTACCTGGATTTCTTGAGAAAAAGAATGAACACAAACCCGTGTCGTGGTCCATACCACTTCCGTGCTCCAAGCAAAA

TCTTCTGGCGAACAGTTAGAGGAATGTTGCCACACAAGCAGTACCGTGGAAAGGATGCGATGGAGAGGCTGAAGGTCTAT

GAAGGTATTCCTCCTCCGTACGACAAGATTAAACGTATTATTGTTCCATCTGCTTTGAGGGTCATCCGCTTGAAGCCAAG

GCGAAAGTACTGCAAGCTGGACCGTCTTGCAAGTGAAGTTGGATGGAAGGCGTCAAAGGTGGTTGGAACCCTGGAG---A

TGACTAACTCGAAGGGTTACCGACGTGGAACTCGGCATTTATTTGCCAGGAAGTTCAGGACACATGGAGTCATTCCATTG

TCTACTTATCTGAAAACGTACAAAAAGGGAGATATTGTGGACATCAAGGGCAATGGTGCCATTCAGAAGGGCATGCCACA

CAAGGTATACCATGGACGCACTGGTGTTGTCTACAATGTTACTGCTCACGCACTTGGTGTCATTGTCAACAAGAGAGTTC

GAATTCTGGAGAAGAGGATCAACGTGCGAGTGGAGCACGTGAAGCACTCTCGTTGTCGTCAGGACTTCCTTGATCGCGTG

AAGGCTAACGAAGTGAAGAAGAGGGACGCCAAGCAACACGGCACTATTGTTCACTGCAAGAGACCAATCCCTGCTCATTT

CGTGAGCAAGTACAACAAGCCAGTCGTTCTCGAGCCAATTCCATACGAATTCATTGCT---GTGACAAATGAGGTTGTCA

CTCGAGAGCACACCATAAACCTGCACAAGCGCATCCATGGCGTTGGATTCAAGAAGAGGGCTCCACGTGCCATTGGGGAA

ATCAGAAGGTTTGCTGAGAAGGCGATGGGCACCCCGGATGTGCGAATTGAGGCACGCTTGAATAAGCACATTTGGTCGCA

GGGAATCAGGAACGTTCCCTTCCGCGTTCGTGTTCGTTTGGCACGCAAGAGGAACGAGGATGAAGATTCACCCAACAAAC

TGTACACCCTGGTTTCCTACGTTCCAGTGACTTCATTCAAG---ACGATCGTCACCCGAACCATCGGAACAAAAATTGCT

TCCGATGGATTGAAAGGCCGTGTTTTTGAAGTCGCGCTTGCGGATCTTCAGAACGACGAAATTGCGTTCCGCAAGTTCAA

GCTAATGAGTGAGGAGGTTCAGGGACGCAACGTGCTCACCAACTTCCACGGTATGGACATGACCACAGACAAGCTCAAGT

CCATGGTCAAGAAGTTCCAGACGTTGATTGAGGCTCACGTTGATGTGAAGACAACCGACGGCTATCTTCTGCGACTCTTT

GCCATTGGCTTCACAGCGCGTCAGAACCAGGACAAGAAGACGTCCTATGCGCAGAGCACGCAGGTCCGAGCGATCCGCAA

GAAGATGGTTGACATCATGACGCGAGAAGTCTCGTCCAGCGATCTGAAGGATGTCGTCAACAAGCTGATTCCAGACAGCA

TTGGAAAAGACATTGAAAAGGTGTCACAGGGAATCTACCCTCTTCACGACGTATACATCCGCAAGGTCAAGGTCCTCAAG

AAGCCCAAGTTTGATGGTGAGGGTGGCGAGAAAATCGAGCGTGCTGACAACTACGAACCACCTGTGCTCGAGTCTGTC--

-GGTCGACCCCTTGAACTGCTCGAGCCAGAAATGCTCCGAGCAAAGGTCCAGGAGCCGATCCTTCTTCTTGGCAAGGAGC

GATTTGCAGGAGTTGACATGCGTGTGCGTGTGAAGGGAGGTGGTAGCAACGCTCAGATCTATGCGGTTCGACAGGCCATC

AGCAAGGCTCTGGTTGCATACTATCAGAAGGTTGATGAGGCTTCAAAGAAGGAGATCCGTGACATCCTCATCTCCTACGA

CCGATCCCTTCTCGTGGCGGATCCACGTCGCTGTGAGCCGAAGAAGTTCGGTGGTCCAGGAGCGCGTGCTCGCTACCAGA

AGTCATACCGA---CGTTACGCTCATGTTGTCTGCAAGAAGGCCAACGTGGATGTGACAAAGCGTGCTGGAGAGCTCTCC

GACGAAGAGAAGCTGATAACGATCATGGGCAACCCTCGTCAGTACAAGATTCCCGACTGGTTCCTGAACAGGCAGAAGGA

CGTTAAGGACGGCAAGTTCGCTCAGGTGATGTCCAACACCCTCGACAGCAAGATCCGTGAGGACCTGGAGAGGCTGAAGA

AGATCAGGACCCACCGTGGCCTCAGACATTTCTGGGGACTTCGTGTTCGTGGTCAGCATACGAAGACGACGGGTAGGAGA

GGACGTACAGTCGGTGTGTCGAAGAAG---TTAAAAGTTCCAGATTGGGCTGATGTAGTCAAGCTTGGAATTCATAAAGA

ACTGGCACCTTATGATGAAGACTGGTTCTTTACCAGAGCATCTGTTGCAAGACACATGTACTTCAGGCCTGTTGGAGTTA

AAGGATTGACCAAAATCTATGGTGGGCGTAAAAACAACGGAACTCGTCCTTCACACTTCAGTCGTGGCTCAAGTTCTGTC

GCTAGAAGGTGCATGCAGGCCTTAGAATCCCTGAAGCTAGTTGAGAAGGATAACAATGGTGGTCGTAAACTGTCCTCTCA

GGGACGTCGAGACTTGGATAGAATCGCCACCCAGATT

>Celegans

TCTCACCGAAAGTTTTCGGCCCCGAGACATGGACACATGGGATTCACCCCAAAGAAGCGCTCCCGCACTTACCGTGGAAG

AATCAAGGCTTTCCCAAAGGATGACAAGAGCAAGCCAATCCATTTGACCGCTTTCCTCGGATACAAGGCCGGTATGACCC

ATATCGTCCGTGATGTCGATAAGCCAGGATCAAAGGTTAACAAGAAGGAGGTTGTCGAAGCCGTCACCATCGTCGAGACC

CCACCAATGGTTATTGCTGGAGTTACCGGATACGTCGACACCCCACAAGGACCACGTGCTCTTACCACCATCTGGGCCGA

GCACTTGTCCGAGGAAGCTCGTCGTCGCTTCTACAGCAACTGGGCTAAATCCAAGAAGAAGGCTTTCACCAAGTACGCCA

AGAAGTGGCAAGATGAGGATGGAAAGAAGCTCATCGAGGCTGATTTCGCCAAGCTGAAGAAGTACTGCTCTTCTATCCGT

GTGATCGCTCACACTCAGATGAAGATCCTTCGCCGTCGCCAAAAGAAGGCTCACCTCGTCGAAATCCAAGTCAACGGAGG

AACCATCGAGCAGAAGGTCGACTGGGCTCGCGAGCATCTCGAGAAACAAGTCCAGGTTGATACCGTCTTCGCTCAGGATG

AGATGATTGACACCATCGGAGTTACCAGAGGTCACGGATTCAAGGGAGTCACCAGCAGATGGCACACCAAGAAGCTTCCT

CGCAAGACGCACAAGGGTCTCCGCAAGGTCGCCTGTATTGGAGCTTGGCATCCATCTCGTGTCGCCTTCACTGTCGCTCG

TGCT---AGACCGCTCGTGACTGTGTACGATGAAAAGTACGAGCAAATCCGGCTCCCAGCCGTTTTCCGTACTCCAATCC

GCCCAGACTTGGTGAGCTTCATCGCCGATCAGGTCCGCAGAAACAGAAGACAGGCCCATGCCGTTAACACCAAGGCCGGA

AAGCAACACTCTGCTGAGTCCTGGGGAACTGGACGTGCCGTTGCCCGTATTCCACGTGTTCGCGGAGGAGGTACCCACCG

TTCCGGACAGGGAGCTTTCGGAAATATGTGCCGTGGAGGACACATGTTCGCCCCACTCAAGGTGTTCCGCCGCTGGCACC

GCAACGTCAACATTGCCCAGAAGAGATACGCTGTTTCCTCAGCTATTGCTGCCTCCGGAATCCCAGCTCTTCTCCAAGCT

CGTGGACACGTCATCGATCAAGTCGCTGAGGTCCCACTTGTTGTTTCCGACAAGGTCGAAAGCTTCCGCAAGACCAAGGA

GGCCGTCGTCTTCCTCCGTCGCTCCCACTTGTGGGCTGATATCGAGAAGGTTTACAACTCGGGACAAGATGCTGAGTGCG

CTCGTGCCTTCCGCAACATCCCAGGAGTCGATGTCATGAATGTTGAGAGACTCAACCTTCTCAAGCTCGCCCCAGGAGGA

CATCTCGGACGTCTTATCATCTGGACCGAGTCTGCCTTCAAGAAGCTTGATACCATCTAC---AGACGTGAGGGAAAGAC

TGACTACTATGCCAGAAAGCGTCTTACCGTCCAGGACAAGAACAAGTACAACACCCCAAAGTACCGTTTGATCGTCCGCA

TCACAAACAAGGATGTTGTCGCTCAGCTCGCTTACTCCAAGATCGAAGGAGACGTCGTTGTCGCTTCGGCTTACTCCCAC

GAACTTCCACGTTACGGACTCAAGGTCGGACTTACCAACTACGCCGCTGCTTACGCCACCGGACTTCTTCTTGCCCGTCG

TCACTTGAAGACCATCGGACTTGACTCCACCTACAAGGGCCACGAAGAGCTTACTGGAGAAGACTACAATGTAGAAGAGG

AGGGAGACCGCGCTCCATTCAAGGCTGTCCTCGACATCGGACTTGCTCGCACCACCACCGGATCCAAGATCTTCGCTGTG

ATG---GTGAACCCACTCTTCGAGAAGAGAGCTCGCAACTTCAACATCGGACAAGACATCCAGCCAAAGAAGGACGTCAC

CCGATTCGTCAAATGGCCAAAGTACATCCGTCTCCAGCGTCAAGCCATTCTCCAGAAGCGCCTGAAGGTTCCACCAACCA

TCAACCAGTTCAGAACCGCTCTTGACAGCCAGTCCGCTAGACAAGCCTTCAAGCTCTTGGACAAGTACCGCCCGGAGTCG

ACCGAGGCCAAGAAGAACCGTCTCCGTGCTCGTGCCGAGGCTCGCGCTGCTGGAAAGAAGGAGGAGGTCACCAAGAGACC

AAACACCGTTCGCCACGGAGTCAACACCATCACCAGACTCGTCGAGACCCGCAGAGCTCAATTGGTTCTCATCGCTCACG

ACGTCAACCCACTCGAGATCGTGCTCCACCTTCCAGCCCTCTGCAGAAAGTACAACGTTCCATACGCCATCATCAAGGGA

AAGGCTTCCCTCGGAACCGTCGTCCGCAGAAAGACCACCGCCGCTGTTGCTCTCGTCGATGTCAACGAGGACAAATCTGC

CTTGAACAAGCTCGTCGAGACCGTCAACAACAACTTCAGCGAGCGTCACGAGGAGATCCGCAAGCACTGGGGAGGAGGTG

TCATGTCCGCCAAGTCTGATGCCAAG---ATGGGACGTCGTATCTTCAAGAGCCACAACAAGCACCGCAAAGGAGCGTCC

CTCCGTCCTCTTGACTATGCCGAGAGACATGGCTACATCAAGGGACTCGTGAAGGACATCATCCACGACCCAGGAAGAGG

AGCTCCACTTGCCATCATCGCTTTCCGCGACCCATACAAGTACAAGACCGTGAAGACTACCGTCCATTGTGGAGCTAAGG

CTCAAATCCAAATCGGAAACATCGTGCCAGTCGGAACCCTCCCAGAAGGAACCACCATCTGCAACGTCGAGAACAAGTCC

GGAGACCGTGGAGTCATCGCCCGTGCCTCTGGTAACTACGCCACCGTCATCGCCCACAACCCAGACACCAAGAAGACACG

TATTCGCCTCCCATCCGGAGCCAAGAAGGTCGTTCAATCGGTCAACCGCGCCATGATTGGACTCGTCGCTGGAGGAGGAC

GTACCGACAAGCCACTTCTCAAGGCTGGACGCTCATACCACAAGTACAAGGCAAAGAGAAACAGCTGGCCACGTGTCAGA

GGAGTTGCCATGAATCCAGTCGAACATCCCCACGGAGGAGGTAACCATCAACATATTGGACATCCATCCACCGTCAGAAG

A---TCTGGAGATAGACTGACCCGTGCCGCTAAGGTGCTCGAGCAGCTCACTGGACAGACCCCAGTGTTCTCCAAGGCCC

GCTACACCGTCCGTACCTTCGGTATCCGCAGAAACGAAAAGATCGCCGTTCACTGCACCGTCCGCGGACCAAAGGCCGAG

GAAATCCTCGAGAAGGGACTCAAGGTCAAGGAGTACGAGCTCTACAAGGAGAACTTCTCCGAC---GTCTTCAACCGTGT

CGTTCAAATTGGGCGCGTCGTCTTCATCGCCTCCGGCAAAGACCAAGGCAAGCTTGCTGCCATCGTCAACGTTATCGACG

GAAACAGAGTTCAAATCGATGGACCATCCTCCGATGTCACTCGCACCGTCAGAAACTTGAAGGATCTTCAGCTCACCAAG

TTCGTCCTCAAGCTCCGTGTTGGACAACGCACCAAGGGAGTTAAGGCAGCTTTCGATGCCGCCAAGGTCACCGAGAACTT

CCAGAAGACTCAATGGGCCAAGAAGATCGAGAGATACAAGCTCATG---TACAAGTATATGCAGGAAATCTGGCGCAAGA

AGCAGTCAGATGCTCTTCGCTACTTGCTCCGTATTCGTACCTGGCACTACAGACAGTTGAGCGCCGTCCACCGTGTCCCA

CGCCCAACCAGACCAGAGAAGGCTCGCCGTCTCGGATACCGTGCCAAGCAAGGATTCGTTGTCTACCGCGTCCGTGTCCG

CAGAGGAAACAGAAAGCGTCCAGTCTGCAAGGGACAAACCTACGGAAAGCCAAAGACCCATGGAGTTAACGAGCTCAAGA

ACGCCAAGTCTAAGCAAGCCGTTGCTGAGGGCCGCGCTGGACGTCGTCTTGGATCCCTCCGTGTTCTCAACTCCTACTGG

GTTGCTGAGGACTCCACCTACAAGTTCTACGAGGTTGTCCTGATCGATCCATTCCACAAGGCTATCCGTCGTAACCCAGA

CACCCAATGGATCACCAAGCCAGTTCACAAGCACCGTGAGCAAAGAGGACTCACCTCTGCTGGACGCAAGTCTCGTGGAC

TCGGAAAGGGATGGCGTTTCTCTGCTACCCGCGGAGGATCCCAAGCCAAGAACTGGAAGAGAAAGAACACCAAAGTTTTC

CACCGCAAGCGA---GCTTTCCTCGCTAGACGCACTGGAGAGAAGTTCAACGCCATCGTCTTGAAGAGACTCCGCATGTC

CCGTCGTAACCGTCAGCCACTCTCGCTCGCCAAGCTTGCCCGCGCCGTCCAGAAGGCTGGAAACGAGAACAAGACTGTTG

TCCTCTCGACTGTCACCGATGATGCCCGTCTCTACACTGTCCCAAAGATCTCCGTCGCCGCTCTTCACGTCACCGAAGGA

GCCCGCGCCAGAATCCTTGCTGCCGGAGGAGAAATCATCACCCTCGACCAGCTCGCCCTCAAGTCGCCAAAGGGAGAAAA

CACCGTCTTCCTCCAGGGACCACGATCTGCTCGTGAAGCCGAGAAGCACTTCGGACCAGCTCCAGGAGTCCCACACTCCC

ACACCAAGCCATAC---AATGAGTACGTCGTCGTCGGACGCAAAATCCCAACAGAGAAGGAGCCAGTGACTCCAATCTGG

AAGATGCAAATCTTCGCCACCAACCACGTCATCGCCAAGTCTCGTTTCTGGTACTTCGTCTCTATGCTCCGTCGTGTGAA

GAAGGCCAACGGAGAGATCCTTTCGATCAAGCAGGTGTTCGAGAAGAACCCAGGAACCGTCAAGAACTACGGAGTCTGGC

TCAAGTACGATTCCCGTACCGGACACCACAACATGTACCGTGAGTACCGTGACACCACCGTTGCCGGAGCCGTCACCCAG

TGCCGTGACATGGGAGCTCGTCATCGTGCCCAAGCTGACCGCATCCACATCCTCAAGGTCCAAACCGTCAAGGCCGAAGA

CACCAAGCGTGCCGGAATCAAGATGTTCCACGACGCCAAGATCAGATTCCCACTCCCACATCGCGTCACCAAGAGAAAGA

ACAGCGTCTTCACAACCGCTCGCCAAAACACC---CTCCGTCTGCAGAAACGTTTGGCTTCGGCCGTATTGAAGTGCGGA

AAGCACCGTGTCTGGCTCGACCCCAATGAGGTCAGCGAGATTTCTGGAGCCAACTCCCGTCAAAGCATCCGCAGACTCGT

CAATGACGGACTTATCATCCGCAAGCCAGTCACTGTCCACTCCCGCTTCCGCGCACGCGAGTACGAGGAGGCCCGCAGAA

AGGGACGTCACACCGGTTACGGAAAGCGTCGTGGTACTGCCAATGCTCGTATGCCAGAAAAGACCCTCTGGATCCGACGC

ATGCGTGTCCTCAGAAACCTTCTGAGAAGATACAGAGATGCCAAGAAGTTGGACAAGCACCTTTACCACGAGCTCTACCT

TCGCGCAAAGGGAAACAACTTCAAGAACAAGAAGAATCTCATCGAGTACATCTTCAAGAAGAAGACTGAGAACAAGAGAG

CCAAGCAACTT---GACAGATACCGTCGTGTATGGCGTAAGCCAAAGGGAATTGATAACCGTGTCCGCAGACGCTTCCGT

GGTATGCGTGCCATGCCAACCATCGGTCACGGATCCGACAGAAGAACCCGCTTCGTCCTTCCAAACGGATACAAGAAGGT

TCTCGTCCAAAATGTTAAGGATTTGGACATGCTCCTCATGCAGTCCTACAAGTACATCGGAGAGATCGGACATGGAGTCT

CCGCCAAGAGCCGCAAGGGAATCGTCGAGCGCGCCGCTCAGTTGAACATCAAGCTGACGAACGGAAACGCTCGTCTT---

ACTCTCCGTGTCTCGAAGGTCACTGGAGGAGCTGCCTCTAAGCTCTCCAAGATTGTCCGCAAGAACATCGCCAGACTCTT

GACTGTCATCAACCAGACCCAAAAGCAAGAGCTCCGCAAGTTCTACGCTGACCACAAGTACAAGCCAATTGATCTTCGCC

TCAAGAAGACCCGCGCCATCCGCAGAAGACTGACCGCTCACGAGCTTTCCCTCCGATCTGCCAAGCAACAGGCTAAGTCT

CGCAATCAAGCCGTCAGAAAGTTCGCAGTCAAG---TCCAAGCAAATGCAAGAGATTCGTCAAGCCATGAGAGGAGCTGA

GATCCTTATGGGAAAGAACACCATGATCCGTAAGGCTCTTCGTGGACACCTCGGAAAGAATCCATCCCTTGAGAAGTTGT

TGCCACACATCGTCGAGAACGTCGGATTCGTTTTCACCAAGGAAGATCTCGGAGAAATCCGCTCCAAGCTTTTGGAAAAC

CGTAAAGGAGCCCCAGCCAAGGCTGGTGCCATCGCTCCATGCGACGTCAAGCTTCCACCACAGAACACTGGAATGGGACC

AGAAAAGACCTCTTTCTTCCAGGCTCTTCAGATCCCAACCAAGATCGCCAGAGGTACCATCGAAATCTTGAACGACGTCC

ATCTCATCAAGGAAGGAGATAAGGTCGGAGCTTCCGAATCTGCTCTTCTCAACATGTTGGGAGTTACTCCGTTCTCATAT

GGATTGGTCGTTCGCGTTTACGATGATGGAACCCTCTACACTCCAGAAGTTTTGGACATGACCACCGAGGAGCTCCGCAA

GCGTTTCCTTTCTGGAGTTCGCAATGTCGCTTCCGTCTCGTTG---ACCACTGCCGGACAACGTACCCGCTTCAAGGCTT

TCGTTGCTATCGGAGATCACGCCGGACACGTCGGACTTGGAGTCAAGTGCTCCAAAGAAGTTGCTACCGCCATCCGCGGA

GCTATCGTTGCTGCCAAGCTCGCCGTTGTCCCAGTCCGCAGAGGATACTGGGGTAACAAGATCGGACTCCCACATACCGT

TCCATGCAAGGTCACTGGAAAGTGCGCCTCCGTAATGGTTAGACTCATCCCAGCCCCACGTGGTACCGGAATCGTGTCAG

CTCCAGTTCCAAAGAAGCTCCTTCACATGGCAGGAATCGAGGATTGCTACACCGCCGCTAAGGGATCCACCGCCACTCTT

---CTTGCTGAGGATGGATACTCCGGAGTCGAGGTTCGCAGTACCCCAGCCAGAGCTGAGGTCATCATCATGGCGACCCG

CACCCAAAACGTTTTGGGAGAGCGAGGACGCAGAATTAAGGAGCTCACCTCGGTTGTCCAAAAGAGATTCGGATTCGAGG

AGGGATCCGTCGAGCTCTACGCCGAGAAGGTTTCCAACAGAGGACTCTGCGCTGTCGCTCAGTGCGAATCCCTCAGATAC

AAGCTCGTCGGAGGTCTTGCCGTCCGTCGTGCTTGCTACGGAGTTCTCCGTTTCATCATGGAGTCTGGAGCTCAAGGAGT

TGAGGTTATCGTCTCTGGAAAGCTTCGTGGACAGCGTGCTAAGGCCATGAAGTTCGTTGATGGACTCATGATTCACTCTG

GACACCCAGTCAACGACTACATCCAACAAGCCGTCAGACACGTTCAACTCCGTCAGGGAGTCATCGGTATCAAGGTCAAG

ATCATGCTTCCATACGACCCACGCGGACAGAACGGACCAAGAAACGCCCTCCCAGACCACGTCCAAATCGTCGAGCCACA

AGAGGAA---GGACCAAAGAAGCATCTTAAGCGTCTCGCTGCTCCAAGCCATTGGATGCTCGACAAACTCGGAGGAGTCT

TCGCTGTTCGTCCAAACCCAGGACCACACAAGCTCCGCGAGTCCCTTCCACTCAGCTTGTTCCTCCGTAACCGTCTGAAG

TACGCTCTCAACTACACCGAGGCCAAGAAGATTCTCACCCAGAGAGTTGTCCGCGTCGACGGAAAGGTCAGAACCTGCCA

CAAGTTCCCAACCGGATTCATGGATGTCGTTGCCATCGAGCGTACCAACGAGTATTTCCGTATGCTCTACGATACCAAGG

GAAGATACGTCGTCCACAGAATCCAAGCTGCCGAGGCCGACTTCAAGTTGGCCTACGTCACTGGAGGACGTAACGTTGGT

CGTGTCGGAATTATCGGACATCGTGAGCGTCTCCCAGGAGCATCTGACATCATCCACATCAAGGATTCTGCTGGACACTC

CTTCGCCACTCGTATCTCCAACGTCTTCGTCATTGGAAAGGGAAACAAGGCTCTCGTCTCCCTCCCAACTGGAGCTGGAA

TCCGTCTCTCAATCGCCGAAGAGCGTGACAAGCGTATGGCCCAG---CTTAACTTCGCCTACCCAGCCACCGGGCTCCAA

AAGTCGTTCGAGGTCGATGAGGAGAAGAAGCTTCGCCTCTTCTTCGAGAAGCGCATGTCCCAGGAGGTCGCCATCGATGC

TCTCGGTGACGAGTGGAAGGGATATGTCGTCAGAATCGGAGGAGGAAACGACAAGCAGGGATTCCCAATGAAGCAAGGAA

TTCTCACCAACGGACGTGTTCGTCTTCTCTTGAAGAAGGGACAATCCTGCTACCGTGAGCGCAAGAACGGAGAGCGCAAG

AGAAAGTCCGTCCGTGGATGCATCGTCGACGCCAACATGTCCGCTCTTTCCCTCGTCATCGTGAAGAAGGGAGATGGAGA

GATCGAAGGACTCACCGACTCCGTTCTCCCACGCAAGCTCGGACCAAAGAGAGCCTCCAAGATCCGCAAGCTCTTCAACT

TGACCAAGCACGACGATGTCACCAAATACGTCATCGACAAGACCAAGACCATCGCACCAAAGATCCAGCGTCTCATCACC

CCAGCTCGTATC---ATCTCGCGCGACTCTTGGCATAAGCGCTACAAGACCGGAGCCACTCAACCGGTCCCTCACAAGAA

GAGGAAGTTCGAGCTCGGACGTCCAGCTGCCAACACCAAGATTCTCGTTCGCACTCGTGGAGGAAACGAAAAGTACAGAG

CTCTTCGCCTCGACTCCGGAAACTTCTCCTCTGAGCAGACTACTCGCAAGACCCGTATCGTTGATACCATGTACAACGCT

ACAAACAACGAGTTGGTCCGTACCAAGACTCTTGTCAAAGGAGCCATCATCAGCGTTGATGCTGCTCCATTCCGTCAGTG

GTATGAGGCTCACTATGCTCTCCCACTTGCCAGAAAGAAGAACGCCAAGGCTCGCATCTCTTCTTCCCCAGGACAAGTCG

GACAAGCCAACGGATACATCCTCGAAGGAAAGGAACTTGATTTCTACCTTCGCAAGATTCGTGCCAAGAAG---AAAGTG

ACAAAGTCGCCACGCCGTCCGTTCGAGAAGGAGCGCCTTGACCAGGAGCTCAAGCTCATCGGAACTTTCGGACTCAAGAA

CAAGCGTGAGGTCTGGCGTGTCAAGTACACCCTCGCCAAGGTCCGTAAGGCGGCTCGTGAGCTTCTTACTCTTGAAGACA

AGGATCCAAAGCGTCTTTTCGAAGGAAATGCCCTTCTCCGTCGTCTTGTCAAGATCGGTGTCCTCGACGAGACCAAGATG

AAGCTCGATTACGTTCTCGGTCTTAAGGTCGAGGACTTCCTTGAGCGCAGGCTCCAAACCCAGGTCTTCAAGCTCGGACT

TGCCAAGTCTATCCACCACGCCCGTATCTTGATCAAGCAGCACCACATCCGTGTCCGCAGACAAGTTGTCGATGTCCCAT

CCTTCATCGTTCGTTTGGACTCCCAGAAGCACATCGACTTCTCTCTTCAATCTCCATACGGAGGTGGC---TTTTTGAAG

CAGCCAACGGTCAACCTTAACAACAAGCGTTACATTCGTGAGGTCGGACTCGGATTCAAGGCTCCACGTGAGGGAACCTA

CATCGACAAGAAGTGCCCATGGGCCGGAAACGTCCCAATCCGCGGAATGATCCTCACTGGAGTCGTCCTCAAGAACAAGA

TGACAAGAACCATCGTCGTTCGTCGTGACTACCTTCACTACATCAAGAAGTACCGTCGTTACGAGAAGAGACATAAGAAC

GTGCCAGCTCACTGCTCCCCAGCCTTCAGAGATATCCACCCAGGAGACCTTGTCACCATCGGAGAGTGCCGACCACTTTC

GAAGACCGTC---GGAATGGCCAAGTCGGCCATCCCATACCGCCGCTCCGTTCCATCATGGCAAAAGATGACCGCCGAGG

AGGTCCAAGACCAAATCGTCAAGATGGCCAAGAAGGGTCTTCGTCCATCCCAAATCGGAGTTATCCTCAGGGATTCCCAC

GGAGTTGGACAAGTCCGCCGTCTTGCCGGAAACAAGATCTTCCGCATCCTCAAGTCCAAGGGAATGGCTCCAGAGCTCCC

AGAGGATTTGTACCATCTCGTCAAGAAGGCCGTCGCCATTCGCAAGCATCTTGAGAGATCCCGCAAGGACATTGACAGCA

AGTACAGACTTATCCTCGTCGAGTCCCGTATCCACCGTTTGGCTAGATACTACAAGACCAAGCGTCAGCTTCCACCAACC

TGGAAG---ATGGTTCGCATGAACGTCCTCGCCGATGCGCTCAACGCCATCAACAACGCCGAGAAGCGCGGAAAGCGTCA

GGTTCTCATCCGTCCAGCCTCCAAGGTCATCGTCCGCTTCTTGACCGTCATGATGAAGCACGGATACATCGGAGAGTTCG

AGATCGTCGACGACCACCGCGCCGGAAAGATCGTCGTCAACCTCACCGGACGTCTCAACAAGGCCTCCGTCATCTCCCCA

CGTCTCAACATCCGTCTAAACGATTTGGAGAAGTACACCAACACCCTCCTCCCATCCCGTCAGTTCGGATATCTCATCCT

TACCACCTCCGCCGGAATCATGGATCACGAGGAGGCCAGAAGAAAGCATTTGGGAGGAAAGATTCTCGGATTCTTCTTC-

--GACAATTCGGAGCACCGCATCCGTCTGACCTTGACCTCCCAAAACGTTAAGCCGCTTGAGAAGGTCTGCGCTCAGCTC

ATCGACGGAGCCAAGAACGAGCACCTTATTGTCAAGGGCCCAATCCGCATGCCGACCAAGGTGCTCCGCATCACCACCCG

CAAGACCCCATGCGGAGAGGGATCCAAGACCTGGGATCGCTTCCAGATGCGCATCCACAAGCGTCTCATCAACCTCCACG

CTCCAGCTGAGGTCCTCCGCCAAATCACTTCGATCTCCATCGAGCCAGGAGTCGATATTGAGGTCACCAGAGCCGAC---

ACTATCAGAACGAGAAAGGTTCTCACCAATAAGCTTCTCTACAGAAAGCAGATGGTTGTCGAGGTGATCCACCCAGGGCG

TCCAACTGTCCCGAAGGCTGATATCCGTGAAAAGATCGCCAAGCTTTATAAGACCACTCCAGACACGGTTATCCCATTCG

GATTCGAATCCAAGATCGGAGGAGGAAAGTCGAAGGGGTTCGCTCTCGTATACGACACGATTGACTTCGCCAAGAAGTTT

GAGCCGAAGTACAGACTCGTTCGC---GGAAAGCCGAAGGGACTCTGCACGGCTCGCAAGCTCAAGACTCATCGTCAAGA

GCAAAGATGGAACGATAAGAGATACAAGAAGGCTCACATTGGAACTCGCTGGAACCCATTCGGAGGAGCTTCTCACGCCA

AGGGAATCGTTCTTGAAAAGATCGGTGTCGAAGCTAAGCAGCCTAACTCTGCTATCCGCAAGTGCGTTCGTGTCCAGCTC

ATCAAGAACGGAAAGAAGATCACCGCCTTCGTCCCAAATGACGGTTGCTTGAACTTTGTTGAAGAAAACGACGAAGTGCT

CGTATCTGGTTTCGGACGTTCCGGTCACGCCGTCGGAGATATTCCCGGAGTTCGTTTCAAGATCGTGAAGGTCGCCAACA

CCTCCCTCATCGCCCTGTTCAAGGGAAAGAAGGAGAGACCACGTTCGATG---CGCGAGGCTGAGGCCAAGGGAGACTTC

TACGTTCCAGCTGAGCACAAGGTCGCCTTCGTCGTCAGAATTCGCGGTATCAACCAGCTTCATCCAAAGCCAAGAAAGGC

TCTCCAGATCCTCCGTCTTCGTCAGATCAACAACGGAGTGTTCGTCAAGCTGAACAAGGCTACTCTTCCACTTCTCCGTA

TCATCGAGCCATACGTAGCTTGGGGTTATCCAAACAACAAGACCATCCACGATCTCCTCTACAAGCGCGGATACGCCAAG

GTTGACGGAGTCCCAATCACCGACAACATCGTCGAGCAGAGCCTCATCATCTGTCTTGAGGATTTGGCCCACGAGATCGC

CACCGTCGGACCACACTTCAAGGAGGCCACCAACTTCTTGTGGCCCTTCAAGCTCAACAACCCAACTGGAGGATGGACCA

AGAAGACCAACCACTTCGTTGAAGGAGGAGACTTCGGAAACAGA---TCGGAAAAGCCACGCAAGTTCCGTGAGACTATC

GAGCTCCAGATCGGTCTCAAGAACTACGACCCACAGAAGGACAAGCGTTTCAGCGGATCCATCAGACTGAAGCACATTCC

ACGCCCAAACATGAAGGTGTGCGTCTTCGGAGACCAGCACCATCTTGACGAGGCTGCCGCCGGAGACATTCCATCGATGA

GCGCCGATGACTTGAAGAAGCTTAACAAGCAGAAGAAGCTCATCAAGAAGCTCGCCAAGAGCTACGATGCTTTCATCGCT

TCCGAATCCTTGATCAAGCAGATCCCACGTATCCTCGGTCCAGGACTCAACAAGGCCGGAAAATTCCCTTCCGTCGTTAC

CCACGGAGAATCTCTCCAAAGCAAGAGTGACGAGATCCGCGCCACCGTCAAGTTCCAGATGAAGAAGGTTCTCTGCCTCT

CCGTCGCCGTCGGTCACGTTGGACTCACCCAGGAGGAGCTTGTCTCCAACATCTCCCTCTCCATCAACTTCCTCGTCTCG

CTCTTGAAGAAGAACTGGCAGAACGTGAGATCGTTGAACATCAAGTCCACCATGGGAAAGCCACAAAGAGTTTAT---GC

CCATTTCCGCAAGCACTGGCACAAGCGCATAAAGACCTGGTTCGACCAGCCAGCTCGCAAACTCCGCAGAAGACAGAACC

GTCAAGCTAAGGCTGTCGAAATCGCTCCTCGCCCAGTTGCTGGACTTAGATCTGTCGTCCGTTGCCCACAGAAGAGATAC

AACACCAAGACTCGTCTTGGACGTGGTTTCTCTCTTCAAGAGCTCAAGGCCGGAATCTCCCAGGCTCAGGCCCGCACCAT

CGGAATCGCCGTTGATGTGAGAAGAACCAACAAGACCGCCGAGGGACTTAAGGCCAACGCCGATCGTCTCAAGGAGTACA

AAGCCAAGCTTATCCTCTTCAAGAAGGCTAGCGCACCA---ATGACGCACTACTCCCGGGCTCCGGAAAACAGCACCAAG

TCGTGCAAGGCTCGCGGATCCGATCTCCGCGTTCACTTCAAGAACACCCACGAGGCCGCCATGGCTCTCCGTGGCATGCC

ACTCAGACGCGCCCAGGCTTTCCTCAACCACGTTAAGGAGCACAAGGAAATCGTTCCATTCAGACGTTTCCACGGAGGAA

TCGGCCGTGCCGCTCAAACCAAGCAATGGAACACCACCCAGGGGAGATGGCCAGTCAAGTCCGCTGACTTCCTCCTCGAT

CTCTTGAAGAACGCTGAGAGCAACGCCGAGTACAAGGGGCTCGATGTTGACCACCTCGTTATTGAGCACATCAACGTTCA

ACGCGCTGCCAAGCTCCGCAGAAGAACATACCGTGCCCACGGAAGAATCAACTACATGTCTTCCCCATGCCACATCGAGG

TCATCTTG---ATGAAGGTCAATCCGTTCGTCTCCAGCGATTCCGGAAAGAGCCGCAAGGCCCACTTCAACGCTCCATCC

CACGAGCGCAGACGCATCATGTCTGCCCCACTCACCAAGGAGCTCCGCACCAAGCACGGAATCCGTGCTATTCCAATCAG

AACCGATGATGAGGTCGTCGTTATGCGCGGACGCCATAAGGGAAACACTGGACGTGTCCTCAGATGCTACCGTAAGAAGT

TCGTTATCCACATCGACAAGATCACCCGCGAGAAGGCTAACGGATCCACTGTGCACATCGGAATCCACCCTTCGAAGGTC

GCTATCACCAAGCTGAAGCTCGATAAGGATCGTCGTGCCCTCGTTGAGCGCAAGGCTGCCGGACGTTCCCGC---CCTGG

AAAGGTCGTCCTCGTCCTCAGAGGAAAGTATGCTGGTCGCAAGGCCGTCGTCGTCAAGCAACAAGACGAGGGAGTTTCGG

ACAGAACCTACCCACACGCCATCATTGCTGGAATCGACAGATACCCATTGAAGGTTACCAAGGACATGGGAAAGAAGAAG

ATCGAGAAGAGAAATAAGCTGAAGCCATTCCTCAAGGTCGTCTCCTACACTCACTTGCTCCCAACTCGTTACTCCGTCGA

TGTCCGTTACAAGACCGGAAAGAACAAGTGGTTCTTCACCAAATTGCGATTC---CTCCGTGGTCACGTCTCGCACGGAC

ACGGACGTATTGGAAAGCACAGGAAGCATCCTGGAGGTCGCGGTAACGCCGGAGGTCAACATCACCACCGTATCAACCGT

GACAAGTACCATCCTGGATACTTCGGAAAGGTTGGAATGAGAGTGTTCCATCTCAACAAGAACCAACACTACTGCCCAAC

CGTCAACGTCGAGCGTTTGTGGTCTCTTGTCCCACAGGAGGTTCGCGACAAATCCCCAGTCATTGACTGCACCAAGCTTG

GATACTTCAAGGTTCTCGGAAAAGGACTCCTCCCAGAGACTCCACTCATCGTTAAAGCCAGATTCTTCTCCCACGAGGCT

GAGCAAAAGATCAAGAAGGCCGGAGGAGCTTGTGTTTTGGTGGCT---ATGTCTGCCAAGAAATCCTTCCTTATCAAGCG

CAAGCTTGCTAAGAAGCAAAAGCAAAATCGTCCAATGCCACAATGGGTCCGCATGAAGACTGGAAACACCATGAAGTACA

ATGCCAAGAGACGCCACTGGAGACGCACCAAGCTGAAGCTC---ATGGCTCGTACCAAGCAAACCGCGCGTAAATCAACT

GGAGGAAAAGCTCCTCGCAAGCAGCTGGCCACTAAGGCGGCTCGCAAGTCAGCTCCAACTACCGGAGGTGTCAAGAAACC

ACATCGTTATCGTCCAGGAACCGTCGCTCTTCGTGAGATTCGTCGTTACCAAAAGTCCACCGAGCTTCTCATCCGCAAGC

TTCCATTCCAACGTCTTGTTCGTGAAATCGCTCAAGATTTCAAGACCGATCTCCGTTTCCAATCCGCCGCCATCGGAGCT

CTGCAAGAGGCTTCTGAGGCCTACCTCGTTGGGCTCTTCGAAGACACCAACTTGTGCGCAATCCACGCCAAACGTGTCAC

CATCATGCCAAAGGACATCCAGCTTGCTCGACGCATCCGTGGAGAACGTGCA---CTGGCTTGCGTCTACGCTGCTCTCA

TCCTTCAAGATGACGAGGTCGCCATCACCGGCGAGAAGATCGCTACCCTTCTCAAGGCCGCCAACGTCGAGTTCGAGCCA

TACTGGCCA---ATGCGTTACGTTTCGGCTTACTTGCTCGCCGTCCTCGGAGGAAATGCCAACCCAAAGGTTGACGACCT

CAAGAACATCTTGAGCGCTGTCGGAGTTGATGCTGATGCTGAGACCGCCAAGCTCGTTGTCTCCAGACTTGCAGGAAAGA

CAGTCGAAGAGCTCATCGCTGAGGGA---GAGACCGAATGGACTCCAGTCACCAAGCTCGGAAGACTCGTCAAGGAGAAG

AAGATCACAACCCTCGAGGAGATCTACCTCAACTCCCTTCCAATCAAGGAGTTCGAGATAATCGACGCCCTTTCCAACCT

TAAAGACGAGGTCCTCAAGATCTCCCCAGTCCAGAAGCAG---AAGTACCTTCCACATTCGGCTGGACGCTTCCAAGTCC

GCCGTTTCCGCAAGGCCGCTTGCCCAATCGTCGAACGTCTCGCCAACTCCTTGATGATGCACGGACGCAACAACGGAAAG

AAGCTTATGACCGTCAGAATCGTCAAGCACGCCTTTGAGATCATCTACTTGCTCACCGGAGAGAACCCAGTCCAAGTTTT

GGTCAACGCCGTTATCAACTCGGGACCACGTGAAGACTCCACCCGTATTGGACGTGCCGGAACTGGAGCTCGTGAGGCTG

CCTTCAGAAACGTTAAGACCATCGCCGAATGCTTGGCTGATGAGCTCATCAACGCCGCCAAGGGATCCTCCAACAGCTAC

GCTATCAAGAAGAAGGATGAG---ATCGGCAAGCTTTTGAAGTCCGACGGAAAGGTCGTCTCTGAGATCGAGAAGCAAGT

CTCACAAGCTCTCATTGACTTGGAGACCAACGATGATGTCCAGAGCCAATTGAAGGAACTGTACATCGTCGGAGTCAAGG

AGGTTGAGCTTGGAAACAAGTCCGCCATCATCATCTACGTTCCAGTCCCACAACTCAAGGCTTTCCACAAGATCCACCCA

GCCCTTGTTCGTGAGTTGGAGAAGAAGTTTGGAGGAAGAGACATCCTTATCCTCGCCAAGAGACGTATCCTTCCAAAGCC

ACAAAGAGGAAGCGCCAGACAAAAGCAAAAGAGACCACGTTCCCGTACTCTTACCGCTGTCCATGATGCTTGGCTTGATG

AGCTCGTCTACCCAGCTGAGGTCGTCGGAAGACGTATTCGTGTGAAGTTGGACGGAAAGAAGGTTTACAAGGTTCACCTC

GACAAGTCCCACCAGACCAACGTCGGACACAAGATTGGAGTCTTCGCTTCGGTTTACCGCAAGCTCACCGGAAAGGATGT

CACCTTCGAGTTCCCAGACCCAATTTTC---ATCCCGAAAAGCCACACCAAGCTCATCTATGAGTACCTCTTCAACGAGG

GAGTGACTGTCGCCAAGAAAGACTTCAATGCCAAGACTCACCCAAACATCGAGGTTTCCAACCTTGAGGTCATCAAGACT

TTGAAGTCTCTTGCCTCCCGCGAGTTGGTTAAGGAGCAATTTGCCTGGCGCCACTACTACTGGTACTTGACCGACGCCGG

AATCTTGTACCTCCGTGAGTACCTCGCTCTTCCAGCTGAGATCGTCCCAGCCACCATCAAG---CACATCACCGATATCT

CAGGACGTGAAACCATCGTTCGAGTTACCGGAGGAATGAAGGTCAAGGCCGATCGTGACGAGTCATCGCCATACGCTGCT

ATGCTCGCCGCTCAAGACGTCGCTGATCGTTGCAAACAACTCGGAATCAACGCTCTTCACATCAAGCTTCGTGCTACTGG

AGGAACCAGAACCAAGACCCCAGGACCAGGAGCTCAGTCTGCTCTTCGTGCCCTCGCTCGCGCTGGAATGAAGATTGGAA

GAATCGAAGACGTT---CTGAAGAAGAAGAGAACTTTCCGCAAGTTCATGTACAGAGGAGTTGACTTGGACCAACTTTTG

GACATGTCCCGTGAACAATTCACCAAGCTTCTCCCCTGCCGCATGCGCAGACGTCTCGATCGCGGACTCAAGAGAAAGCA

CTTGGCTCTCATCGCTAAGGTCCAAAAGGCCAAGAAGGCTGCTGGTGTTCTCGAGAAGCCAGCCACCGTTAAGACCCATC

TCCGAGACATGATCATCCTTCCAGAGCTCGTTGGTGGAGTCATCGGAATCTACAACGGAAAGGTTTTCAACCAGACCGAA

ATCAAGCCAGAGATGATCGGATTCTATCTCGGAGAGTTCGCTATCTCCTACAAGCCTGTCAAGCACGGAAGACCAGGTAT

TGGAGCTACCCACTCTTCCCGATTCATCCCACTCAAG---CTCATCGAGAAGTACTACACTCGCATGACCAATGACTTCC

ATAACAACAAGCGCGTCTGTGACGAGGTTGCCATCATCGGAAGCAAGCCACTCAGAAACAAGATCGCTGGATACATCACT

CACTTGATGAGACGTATCGAGAGAGGACCAGTCAGAGGTATCTCCATCAAGCTTCAAGAAGAGGAGCGTGAGCGCAGAGA

CAACTACATGCCAGAGATCTCCACTGTTGATCAACTCACCATCAAGGTTGACACTGATACCAGCGACATGCTCAAGGCC-

--CCAAGAAAATGCTCTGCTAGCAACAGAATAATTTCTGCCAAAGATCATGCCGCCATTCAAATCAACCTTGCTGAGGTT

GATGAGCAAACTGGAAGAATGACTGGTCAGAACAAGACATATGCCATATGTGGAAACATCAGGAGAATGGGAGAATCAGA

TGATTGCCTCAACTTCTTATGTAAAAGAGATTCGATCCTGCCAAAG---CACGTCGCGTTCATTCGCTGCACCAACTGCG

GACGCTGCTGCCCAAAGGATAAGGCCATCAAGAAGTTCGTTGTCAGAAACATCGTTGAGGCTGCCGCCGTTAGAGACATC

GGAGATGCTTCTGCTTACACCCAGTATGCTCTTCCAAAGCTCTACCACAAGCTCCATTACTGCATCGCCTGCGCCATCCA

CAGCAAGGTCGTCAGAAATCGTTCCCGTGAGGCCAGAAGAGATCGCAACCCACCACCACGTTTCCAGGGA---ATGGTCT

TCGTCAAAACACTCACGAACAGAACCTTGTACCTCGAGGTCATCAAGCAAAAGATCGAGGAGGGTATCCCATCCGCTGAG

CAGAGACTCGTCTTCGCCGGACGTCAGCTCGAGGATAGCGACCTCGACGCCGAGGCCACCATCTACGTCGGAGGAGCCAA

GAAGAGAAAGAAGAAGGTCTACACCACTCCAAAGAAGAACAAGAGAAAGCCAAAGAAGGTCAAGCTCGCTGTCCTCAAGT

ACTACAAGGTCGACGAGAACGGCAAGATCACCCGTCTTCGCAAGGAGTGCCAACAGCCATCGTGCGGAGGAGGAGTCTTC

ATGGCCCAACACGCCAACAGACACTACTGTGGAAGATGCCACGACACCCTCGTCGTCGACAAG---AAGCCATACCCAAA

GTCCCGCTTCTGCAGAGGAGTTCCGGACGCGAAGATCAGAATCTTCGATTTGGGAAACAAGAGGGCTAACGTTGACACCT

TCCCAGCATGTGTGCACATGATGTCCAACGAGCGTGAGCATCTTTCCTCTGAAGCTCTCGAAGCCGCCCGTATTTGCGCC

AACAAGTACATGGTCAAGAACTGCGGAAAGGACGGATTCCATCTCCGTGTTCGCAAGCATCCATTCCACGTCACCCGTAT

CAACAAAATGCTTTCCTGTGCCGGAGCTGATCGTCTCCAAACTGGAATGCGTGGAGCTTACGGAAAGCCACAAGGATTGG

TCGCTCGTGTCGACATTGGAGATATTCTTTTCTCCATGAGAATCAAGGAAGGAAACGTCAAGCACGCTATTGAAGCCTTC

CGTCGTGCCAAGTTCAAGTTCCCAGGACGTCAAATCATCGTCTCTTCCCGCAAGTGGGGATTCACC---AAGAGAACTAA

GAAGGTCGGAATCGTCGGAAAGTACGGAACCCGTTATGGTGCTTCCCTCCGTAAGATGGCCAAGAAGCTCGAAGTCGCAC

AGCACTCCCGCTACACCTGCTCGTTCTGCGGAAAGGAGGCCATGAAGCGAAAGGCCACCGGAATCTGGAACTGTGCCAAG

TGCCACAAGGTCGTCGCCGGAGGAGCCTACGTCTACGGAACCGTCACCGCCGCTACCGTCAGATCCACCATCAGAAGACT

GAGAGATCTT---CATATCCAGAAGAAGCGTTGCGCTTCCTGCGGATATCCAGATGCCAAGAAGAGAACCTACAACTGGG

GAGCCAAGTCCATCCGCAGACGCACCACCGGAACCGGCCGCACCCGTCATCTGAGAGACGTCAACGCTCGCTTCAGAAAC

GGATTCCGCGAGGGAACAACT---AAGGGAAAGGAATCCAAGTTCGCTCAAGGACGTCGTCGTTACGACAGAAAACAATC

TGGATTCGGAGGACAAACCAAGCCAATCTTCAGAAAGAAGGCCAAGACCACCAAGAAAATCGTCTTGAGAATGGAGTGCA

CCGAGTGCAAGCACAAGAAGCAACTCCCAATCAAGAGATGCAAACACTTCGAGCTCGGAGGACAGAAGAAGTCCCGC---

GTCTCCAAGAAGACCAAGATCGTCCGAGAGCTCGTTCGTGAGATCACCGGATTCGCTCCATACGAGCGTCGCGTTCTGGA

AATGCTTCGTATCTCCAAGGACAAGCGCGCCCTCAAGTTCTTGAAGAGACGCATCGGAACCCACCGTCGTGCTAAGGGAA

AGAGAGAGGAACTCCAAAACGTCATCATCGCTCAACGCAAAGCCCAC---AAAAATGCTGAGAACATCAACTCCCGACTC

TCCATGGTCATGAAGACTGGACAATACGTCCTCGGATACAAGCAGACTCTCAAATCCCTTCTCAACGGAAAGGCTAAGCT

TGTCATCATTGCCAACAACACTCCACCACTCAGAAAGTCCGAGATTGAGTACTACGCCATGCTCGCCAAAACTGGAGTTC

ACCACTACAACGGAAACAACATTGAGCTCGGAACCGCCTGTGGACGTCTCTTCCGTGTGTGCACCCTTGCAGTCACTGAT

GCCGGA---CAAAGAAACGGAACGTTCGCTTCCAAGACTGTCACTCTCCGCAAGACTCTCACTCCAGGAACTGTCCTCAT

CGTTCTCGCTGGACGTCATAAGGGAAAGCGTGTTGTCTTCTTGAAGCAGCTCCCATCCGGACTTCTCCTTGTCACTGGAC

CACACAAGATCAACGGATTCCCACTTCGCAGAATCGGACAAGCTTTCGTGATCGCTACCTCACTTAAGGTCAACGTCTCC

GGAGTCAAGATCCCAGAGCACATCAACGACGAGTACTTCAAGAGAAAGTCCACCGGAAAGAACATCTTTGCTTCCGGAAA

GGAGTACACCGTCTCCGAGCAGAGAAAGAAGGACATCAAGACCGTTGATGCCCCAATCCTC---GTCCATGTCACCGGAC

CACGCGGAACCATCCGCAAGGATTTCCGTCACTTGGAGATGGAGCGTATCCGCACTGTCTGCTCCCACATCAAGAACATG

ATCAAGGGAGTCACCGGATTCCGCTACAAGATGCGTTCCGTATATGCCCATTTCCCCATCAACGTTACCCTTCAAGATGG

AAACAGAACTGTTGAGATCCGTAACTTCTTGGGAGAGAAGATCGTTCGTCGTGTCCCACTTCCAGAGGGAGTCATCGCCA

CCATCTCCACCGCCCAAAAGGACGAGATCGTAGTCGAGGGAAATGATGTCCAATTCGTATCTCAAGCCGCTGCCCGTATC

CAACAATCCACCGCCGTT---GTGTACCTCCGTTGCGTCGGAGGAGAAGTCGGAGCCACCTCCGCTCTTGCCCCAAAGGT

CGGACCACTTGGATTGTCTCCAAAGAAGATCGGAGAAGACATCGCCAAGGCCACCCAAGACTGGAAGGGTCTTAAGGTCA

CCTGCAAATTGACCATCCAGAACCGTGTTGCCAAGATCGACGTTGTCCCATCTGCTGCTTCCCTCATCGTCAAGGAGCTC

AAGGAGCCACCACGTGACCGCAAGAAGGTCAAGAACGTGAAGCACAATGGAGACCTCACTGTTGACACCATCATCAAGAT

CGCCAGAATCATGAGACCAAGATCGATGGCCAAGAAACTTGAAGGAACCGTCAAGGAGATCCTTGGAACAGCCCAATCCG

TTGGATGCACAATTGATGGACAACACCCA---GACGGAAAGAACCACCTTCTCGGTCGGTTGGCTTCCATCGTTGCAAAG

AAGCTTCTCCAAGGAGACAAGGTTGTCGTCCTCAGAGCTGAAGAGATCGTCATCTCCGGAAACTTCCACCGTTCCAAGCT

GAAGTACATGAGCTTCCTCCGCAAGCGTTGTAACATCAACCCAGCCCGTGGAGCCTTCCATTACCGTGCTCCAGGAAAGA

TCTTCTGGCGTACCGTCAGAGGAATGCTCCCACACAAGACCAACCGCGGAAACGAGGCTCTCAAGAACCTCCGCGCCTAC

GAAGGAGTTCCAGCCAAATACCAGAAGACCAAGTCTCTCCATGCCCCATCAGCCAGCAGAGTATTCCGACTTCAACCAAG

AAGAAAGTTCTGCGTCGTTGGACGCTTATCGCACGAGGTCGGATGGCAATTCCAGGATGTTGTTGCCAAGCTCGAG---A

TGACTAACTCCAAGGGTCTCAGACGCGGAACGCGTTACATGTTCGCTCGCGACTTCCGCAAGCACGGAGTTGAGCACCTC

TCCACCTACTACACCCAGTACAAGCGCGGAGATCTTGTCGATATCAAGACCAATGGAGCTTTCCAAAAGGGTATGCCATT

CAAGGCTTACCACGGAAGAACCGGAAGAATCTTCAACGTCACCAGAGGAGCCGTCGGAATCATCGTCAACAAGAGAGTCA

ACATTCTTCCAAAGAGAATCAACATCCGTATCGAGCACATCAAGCCATCCAAGTGCAGAACCGACTTCTTGAACCGTGTC

AAGTCCAATGACGAGAAGAGAAAGGCCGCTAAGTCAGCTGGACAAGTCCCAGCTTTGAAGAGACCTAGAGGAGCTCACAC

CGTCACCCAGAACAATGAGCCAGAACTTCTCGCCCCACTCCGTTTCGAGATTGTTGCG---ACCATCAACGAGGTCGTCA

CCCGTGAATACACCATTCACATCCACGCCCGCATCAGAGGAATCGGATCCAAGAAGCGTGCTCCACGTGCCATCGACGAG

ATCAAGAAGTTCGCCAAGATCCAGATGAAGACCAACGATGTGCGTGTCGACACCAAGCTCAACAAGTTCATCTGGTCCAA

GGGAATCAAGAACGTTCCATACAGAGTTCGTGTCCGTCTCTCCCGCCGCCGTAACGAGGATGAGGACTCCGCTCAAAAGC

TCTACACCCTCTGCACCTACGTTCCATGCACCAACTTCCAC---ACCCTTATCAACAGAACCCAAGGAACTAAGATCGCT

TCCGAAGGACTTAAGGGACGTGTCTTCGAGGTTTCACTCGGAGACTTGAACAACTCTGAGGCCGACTTCAGAAAGTTCAA

GCTCATCGCTGAAGATGTCCAGGGAAAGAACGTTCTCACCAACTTCCACGCTATGTCGATGACCCACGATAAGTTGTGCT

CCATCGTCAAGAAGTGGCATACTCTCATCGAGGCCAACACCGCCGTCAAGACCACTGATGGATACACTCTCCGTGTCTTC

GTTATCGCCTTCACCAAGAAGGTCAACCAAGTCAAGAAGACTTCCTACACCAAGACATCCAAGATCCGCAAGATCCGTTC

CGAGATGATCGGATGCATCGAAAAGGAGGTCACCGGATGCGACCTTAAGGAGGTTGTCAGCAAGCTCATCCCAGATTCCA

TCGGAAAAGATATTGAGAAGACCTGCTCCAAGCTCTACCCACTCCAAGAAGTCTACATCCGTAAGGTCAAGATCATCAAG

AGACCAAAGGTTGATGGAGCCGACGGTGAGAAGGTTGATCGCCCAGACGACTACGAGCCACCAGTCCAACAAGAAGTT--

-GGTCGCCCACTTGAGTTCTTGGAGCCACAGATCCTGCGCATCAAGCTTCAGGAGCCACTTCTCCTCGTCGGAAAGGAAC

GCTTCCAGGATGTCGACATCAGAATCCGTGTTAGCGGTGGAGGACACGTTGCCCAGATCTACGCTGTCCGTCAAGCCCTT

GCCAAGGCTCTCGTTGCCTACTACCACAAGGTTGACGAGCAGAGCAAGAGAGAATTGAAGAACATCTTCGCCGCTTACGA

CAAGTCCCTTTTGGTTGCTGACCCAAGACGCCGTGAGTCCAAGAAGTTCGGTGGACCAGGAGCCCGCGCTCGCTACCAGA

AGTCTTACCGT---CGTTTCGCCTTCGTCTGCTGCCGCAAAGCTGACGTTGATGTCAACAAGCGCGCCGGAGAGCTCACT

GAGGAGGACAAGATTGTCACCATCATGCAAAACCCATCTCAATACAAAATCCCAAATTGGTTCCTTAACAGACAAAAGGA

CATCAAGGACGGAAAGACTGGACAGCTCCTCTCCACCGCCGTCGACAACAAGCTCCGTGAGGATCTTGAGCGCATGAAGA

AGATCCGTCTCCACAGAGGACTCAGACATTACTGGGGACTTCGCGTCAGAGGACAGCACACCAAGACCACCGGACGCAAG

GGACGCACCGTCGGAGTGTCCAAGAAG---GTTAAGGTTCCAGAGTGGTCCGATCTTGTTAAGCTCGGAGTCAACAAGGA

GCTTGCCCCAGTCGATCCAGATTGGTTCTACACCCGTGCCAGTCTTGCTCGTCACTTGTATTTCCGCCCCGCCGGAATCG

GAGCCTTCAAGAAGGTCTACGGAGGAAACAAGAGACGTGGTGTTGCACCAAACCATTTCCAAACCTCAGCTGGAAACTGC

CTCAGAAAGGCTGTTCAGCAACTCGAGAAGATCAAGTGGGTTGAGAAGCACCCAGACGGCGGAAGAATTCTCTCCAAGCA

GGGACGCAAGGATCTTGACAGAATCGCCACCAGCCTC

**B. Lophotrochozoan group**

>Capite

TTCGTCAAAGTTGTGAAGAACAAGGCCTACTTCAAGAGGTACCAGGTGAAATACAGGAGACGCAGAGAGGGTAAAACCGA

TTACTATGCTCGCAAACGTCTTGTCATCCAGGACAAAGATAAATACAACACTCCAAAGTACCGCATGATCGTTCGCTTCA

CCAACAAAGATGTTGTTTGCCAGGTAGCGTACGCCAGAATCGAGGGTGATTACATCGTGGCTGCGGCTTACGCCCACGAG

TTGCCCAGATACGGCGTCAAGGTCGGGTTGACAAACTATGCGGCTGCATACTGCACTGGACTTCTGCTTGCACGCAGAAT

TCTCAAGAAGTTCAAGTTGGACTCAATCTATGCTGGACAGAAGGATGTCGATGGTGAGGAGTACCATGTGGAGAGTGAGG

ATGGACAGCCAGGTGCCTTCAGGTGCTACTTGGATGTTGGTCTTGCACGTACCAGCACTGGTGCCAAGGTGTTTGCTGCC

ATGAAGGGAGCCGTTGATGGTGGTCTTGACATTCCTGATGCCGGAGTTCTTCGTGATCACATCATGGGCAAGCACGTTGG

CGACTACATGCGTCTGCTGCAGGAGGACGATGACGAGGCCTTCAAGCGCCAGTTTGCTCAGTACATCAAGAATGGTGTGA

CCGCTGACAACATTGAGGCCGTCTACCAGAAGGCCCACGCTGCCATT---CGTGATGCCCGCAAGCATGGCAACTTCTAC

GTGCCCCCAGAGCCCAAGGTTGCTTTCGTGATGCGTATTCGAGGGGTCAACGGTGTTGCTCCCAAGCCCCGTAAGGTCAT

GCAGCTGTTCCGTCTTAGGCAGATCAACAACGGAACCTTCGTCAAGTTGAACAAGGCCACGCTTCAAATGATCCGTCTGG

CCGATCCCTTCATCACCTGGGGGTACCCCAACCTGAAGAGCGTGCGTGAGCTCATTTACAAGAGAGGATTCGGCAAAGTG

AACGGATGCAGAGTGCCTCTGGCTGACAACATCATTGAGGAGAAACTTATCATCTGTATGGAGGATCTGATCCACGAGAT

CTTCACCTGCGGCCCCAACTTCAAGGCTGCCAACAACTTCCTGTGGCACTTCAAACTGAACACGCCCACTGGAGGCTGGA

GAAAGAAGAACAACCATTTCGTTGATGGTGGTGACTTTGGCAACCGGGAG---GTCAATCCCCTTTTTGAGAAGAGGCCC

AAGAACTTCGGCATTGGTCAAGACATCCAGCCCAAGCGTGATTTGACCCGTTTTGTGAGATGGCCCAAGTATGTGCGCCT

GCAGCGCCAGAAGGCTACCCTTCTGAAGCGTCTGAAGGTCCCTCCCCCAATCAACCAGTTCAACCAGACCCTGGACAGGC

AGACCGCTACCCAGATGTTCCGTGTCCTGGACAAATACAGGCCTGAGAGCAAGCAGGAGAAGAAAGCTCGCCTGAGTGCC

AGAGCTGAGGAACGCGTGAAGGGAGGCGCTGATGTGCCCACCAAGCGCCCCCCAGTTGTCCGCTCGGGAATCAACACCAT

CACCTGCCTGGTTGAGCAGAAGAAGGCTCAGTTGGTCGTCATTGCACACAATGTCGATCCTCTCGAGGTGCGTCTTTTCC

TGCCTGCTCTGTGCAGGAAGATGGGAGTTCCTTATTGCATCGTCAAGGATAAGGCTCGTTTGGGACGTGTCGTGAGACGC

AAGACCGCCACTGCCCTCGCTCTGACCCACGTCAACGAGGACAGAAGCTCCCTGAACAAGTTGGTCGAAGCCGTCAAATG

CAACTACAATGACCGCGTTGATCAGATCAAGAAACACTGGGGAGGTGGCATCAACAGTTCCCGTTCACAGGCCAAA---G

TGACGGTTAAGGGACCCAGGGGTACCCTGTCCCGTAACTTCAGCCACTTGGCTGTCCGCACTGTCTGCTCTCACATCGAG

AACATGATCAAGGGAGTCATCCAGGGCTACCGTTACAAGATGCGCTCCGTGTACGCTCATTTCCCCATCAACATCGCCAT

CGCTGAAGACAAGAAACACGTTGAGGTGCGTAACTTCTTGGGAGAGAAGTACACTCGTCACGTGAAGATGCTGGGAGATG

TCGTCATTAAGGATGAGTTCATTCTGGAAGGAAACGACATCGAGCTGGTCTCTCGCTCAGCTGCTCTGATCCAACAGTCA

ACCACTGTG---AAGCCATACCCCAAGTCTCGTTTCTGCAGGGGTGTGCCTGATCCCAAGATCCGTATCTTCGATTTGGG

TCGCAAGAAGGCGCGTTCTGATGACTTCCCTCTTTGCGTGCATCTCGTGTCTGATGAGTGGGAACAGATCTCCTCTGAGG

CTCTGGAGGCCGGCCGTATCTGCGCCAACAAGTACTTGACCAAGCACTGTGGCAAAGACGCTTTCCATCTGCGTGTGCGC

CTCCATCCCTTCCACGTTGTGCGCATCAACAAGATGTTGTCCTGTGCTGGAGCTGATAGGCTCCAGACCGGTATGCGTGG

TGCCTTTGGCAAACCCCAGGGCACTGTCGCCCGTGTGCACATTGGTCAGGTAATCATGTCCGTGCGCGCCAGGGACACTC

ACACCGCTAAAGTGATTGAGGCCCTGAGGAGAGCCAAGTTCAAGTACCCTGGACGCCAGAAGATCTACATCAGCTCCAAA

TGGGGATTCACC---TTTACCGAGACTGTGGAACTGCAGATCTCCTTGAAGAACTACGATCCCCAGAAGGATAAGCGTTT

CGCTGGTACCGTCAAGCTGAAGCACATTCCCCGTCCTCGCATGAAGGTCTGCATCCTGGGCGATCAGCAGCATTGTGATG

AAGCCAAGGTCAACAACCTTTCCAACATGGATGTCGACTCTCTGAAGAAGCTGAACAAGAACAAGAAATTGGTCAAGAAG

CTTGGTCGGAAGTACAGTGCTTTCCTCGCATCTGACTCCCTCATCAAGCAGATCCCCCGTATCTTGGGACCTGGTCTGAA

CAAGGCTGGCAAGTTCCCCACACCCCTGAGTCATGGAGAATCCATGTCTGCTAAGGTCGAGGAGGTTAAAGCCACCATCA

AGTTCCAGATGAAGAAGGTCCTCTGTCTGTCTGTTGCCGTTGGACACGTCCAAATGAGCGCTGATGAGCTGACCTCCAAC

ATCATTCTGTCGGTCAACTTCCTGGTTTCTCTGCTGAAGAAGAACTGGCAGAACGTCCGCGCTCTGTACATCAAGAGCAC

CATGGGCCCCGCTATCCGCATCTTC---GTGTACATGAGAGCAGTTGGAGGAGAAGTAGGTGCAACTTCTTCACTCGCTC

CCAAGATTGGTCCACTGGGTCTGTCTCCCAAAAAAGTAGGTGATGACATCGCCAAGGCCACTCAGGACTGGAAAGGACTG

AAGATCACTGTCCAACTGACCATCCAGAACAGACAGGCCAAGGTTTCAGTGGTTCCAACTGCCTCTTCCCTCATCATCAA

GGCCCTGAAAGAGCCTCCAAGAGACAGGAAGAAGGTCAAACATGTGAAACATAGTGGAAACGTGTCCTTCGATGAAATTC

TCACCATTGCACGAACCATGAGGCCAMGAAGTATGGCAGTTCACTTGAGTGGCACAGTCAAGGAAGTATTAGGAACAGCC

CAGTCTGTGGGATGCACAGTTGATGGCAACCATCCT---AATGGAATGATTCCGAACTCTCATTTTCACAAGCACTGGCA

AAGGAGAGTAAAGACCTGGTTCGCCCAGCCTGCCCGTAAGGACCGCAGAAGGACTCTGCGTGCTGCCAAAGCCGCTGCTA

TCGCACCTCGTCCCGTTGCTGGCATCAGGCCCATGGTCCGTTGTCCTACCTTCAGGTACCAGACCCAGGTCAGAGCTGGT

AGAGGATTCACCTACGAGGAGCTTAAGGTTGGCATCAACAAGAGAGAGGCCCGCTCGATCGGCATCTCTGTTGACCACCG

TCGCAAGAACCGATCCGTTGAGTCTCTGCAGCATAATGTGCAGCGTCTGAAGGAGTACAAATCCAAGCTGATCCTCTTCC

CAAGGAAGGCGTCCGCACCCAAGAAGGGAGATGCCACCGAGGAGGAGATGAAGGTGGCCACCCAGCACAAGGGCCCCATC

ATG---GATGGTAGGGGTCACCTTATGGGTAGGCTCGCTACCCTTGTGGCCAAATCACTGTTGCAAGGTCAACGCGTTGT

GATTGTGAGATGTGAGGGAATCAATATTTCTGGTACCTTCTACAGGAACAAGTTGAAGTACCTGGCTTACCTTCGCAAGA

GGATGAACACAAACCCCTCCCGTGGCCCATTCCACTTCCGTGCTCCCAGCAAGATCTTCTACCGTGTTGTCAGAGGTATG

CTGCCCCACAAACAGCACCGTGGACGTGAGGCTCTTGGTCGCCTGAAGGTCTTCGAGGGCATCCCACCCCCATATGACAA

GCAGAAGCGCGTTGTTGTTCCCTCTGCACTCAAGGTACTGCGTCTGAAGAGCCGTCGTCCCTTCTGCAGCATTGGCCGTC

TGTCCTCGGAGGTCGGCTGGAAATACCAGGGAGTTGTTGCTACTCTGGAGAAAGCAAAGAAGGCTGTTGCTGCC---ACT

TTCAACAAGTACGTTCAGATCGGCCGTGTGACTCTCGTGGCCTACGGGCCTGACCAGGGAAACCTTGTGGTCATCATTGA

CGTCATTGATCACAACAGAGCCCTGGTTGATGGACCCTGCACTGGCGTCAAGCGCCAGGCCCTGCAGTTCAAGGGCATGC

ACCTGACGAAATTTGTCATTCCCGTGCCCCGTTCTGCTCGTACTTGCATCATTAAGAAGAAGTGGGAGAAGAGCGGCATC

ACTGAGAAGTGGCAGGAGACGTTCTGGTCCAAGCGTCTCAACGATGAGAAGCTGAAGAAAAACATGAACGACTTTGACCG

CTTCAAGCTGATGAAGGCCAAGCAG---ATGACCCGTTACTCGCAGGACGCCGAGAATGCCACAAAAGCGGCGAAGGCCA

AGGGATCTCATCTCCGTGTTCACTTCAAAAACACGCGCGAGACAGCTAATACCATCAAGCGTATGCATCTGCGTCGTGCC

GTTGCTTTCCTGAAGAATGTCACCCAGATGAAGGAGTGCGTACCCTTCCGTAGGTACAATGGAGGTGTCGGACGTTGCGC

CCAGGCAAAGAACTGGAACACCACCCAAGGAAGGTGGCCCAAGAAGAGTGCCGAGTTCTTGCTGCAGCTTCTCAAGAATG

CTGAGAGCAACGCCGAATACAAGGGCCTGGACACTGATCACCTTGTGATTGAGCACGTGCAGGTGAACCGCGCTCCTAAG

ATGCGCAGGAGGACGTACAGAGCGCACGGCCGCATCAACTACATGAGCTCCCCCTGCCACATTGAGATCATCTTG---GG

TAAGAACCTGTACGTCATTGCGGTTCATGGCATCAGAGGTCGTCTGAACAGATTGCCTGCTGCTGGCATGGGAGACATGT

TCGTGGCCACCGTCAAAAAGGGAAAGCCAGAACTCCGTAAAAAGGTAATGCCTGCTGTGGTCATCCGGCAGCGTAAAGCG

ATTCGGAGGATAGACGGGGTGTTCTTATACTTTGAAGACAACGCAGGTGTCATAGTCAATAACAAGGGCGAGATGAAGGG

TTCTGCCATCACTGGTCCTGTTGGTAAGGAGTGTGCTGATCTGTGGCCACGTATCGCCTCGAACGCCAGCTGCATC---C

TGGTGAAGGTACGTCGTCACTTGGCCAGAAGGACCGATGCCAAGTTCAACAAGATCATCCTCAAGAGACTCTTTATGAGC

CGCACCAACAGAGCTCCTCTGTCCATCCACAGACTGCGTTTGATGAAGAAGCCTGGTGAGAACAAAGTCGCTGTGGTCGT

TGGAACCATCACCAATGATGTCAGAATCTTTGAGATCCCCAAAATGAAGGTGTGCGCCCTTCACGTCACTGAGGAGGCTC

GTGCTCGCATCATCAAGAGCGGTGGTGAGATCATGACCTTTGACCAGTTGGCCCTGAAGGCCCCCAAGGGACAGAGGACC

GTCCTCGTCCAGGGTCCCC

>Alvine

TTTGTGAAGGTCGTCAAAAACAAACAGTACTTCAAACGATACCAAGTGAAGTTCAAGAGGAGGAGAGAGGGCAAGACCGA

CTACTATGCCCGCAAGCGCCTCATCTTCCAGGCCAAGAACAAGTACAACACACCCAAGTACAGGATGATTGTACGCTTCA

CCAACAAGGATATCGTATGCCAGATAGCCTATGCCAAGATTGAGGGTGATGTTGTTATCTGTTCTGCCTATGCCCATGAG

CTGCCACGTTATGGGGTCACGGTTGGATTAACCAATTATGCTGCAGCATACTGTGTTGGCCTACTTTTGGCTCGCAGGAT

GCTGAAGAAGTTTGGTTTGGATAAAATATATGCTGGTAGTGTGGATGTGACTGGAGATGAGTACATTGTGGAGCCGATTG

ATGGACAGCCAGCACCATTCCGATGCTACCTAGATGTCGGCTTGGCAAGGACCAGCACTGGTGCCAAGGTGTTTGGTGCT

CTGAAGGGTGCAGTGGATGGTGGTATGAACATCCCCAAGGCTGATGTCCATCGTATGCACATCATGGGACAGCACGTGGC

TGACTACATGCGGCTCCTGCAGCAGGATGATGAGGATGCCTACAAGAGGCAGTTCTCACGCTTCATCAAGAATGGTGTAA

CACCTGACTCAATGGAAGAAATGTACAAGAAGTGCCATGCTGCAATA---AGGTTGGCCAGGAAACATGGTAACTTCTAT

GTGGCTCCAGAGGCCAAGGTTGCCTTTGTAATCAGGATCAGAGGCATCAATGGCCTTCATCCAAGACCACGTAAAGTGCT

GAAGCTATTCCGTCTGCTTCAGATTAACAATGGCACCTTTGTTAGATTAAACAAAGCCACACTGCACATGCTGAGGATAG

CTGACCCATATGTCACATGGGGGTATCCAAATCTGAAGAGTGTTCGTGAACTGATTTACAAACGAGGCTATGGAAGGATT

GGTGGCCGTCGGATACCTCTGACTGACAATCTTATTGAGAGAAGTCTCATCATCTGTATTGAGGACTTGATCCATGAAAT

CTACACATGTGGGCCACATTTCAAGGAAGCCAACAGGTTCCTGTGGACTTTCAAACTGAACACTCCAACAGGAGGATGGG

TGAAGAAGAACAACCACTTTGTTGATGGTGGCGACTTTGGCAACAGAGAA---GTCAATCCGCTGTTTGAGAAGAGGCCT

AAGAATTTTGGAATAGGCCAGGATATTCAGCCGAAGAGAGATTTGAGCCGATTTGTAAAATGGCCAAAATACATCCGACT

GCAGCGTCAGAGGGCAATACTTTACCAGAGGCTAAAGGTTCCGCCTCCAATCAACCAGTTCACACAGGCCCTGGATAGAC

AGACAGCCACACAGCTGTTCAGACTTCTCGATCGCTACAGACCAGAGACCAGACAGGCAAAGAAGATCCGACTGCGACTT

CGTGCTGAGGAGAGAGTCAAGGGCAAGCCAGATGTGCCGACAAAACGTCCTCCAGTTGTGAGATCTGGTGTCAACACAGT

CACATCCTTGATTGAGCAGAAGAAGGCTCAGCTGGTCATCATAGCACATGATGTTGACCCTGTTGAGTTAGTTTTGTTCA

TGCCTGCTCTGTGCCGTAAGATGAACGTCCCTTATTGTATCGTGAAAGGAAAGGCTCGACTTGGGCGTGTCGTACGTCGG

AAGACGGCCACTTGTCTGGCTTTGACACATGTAAACGAGGACAAATCATCATTAAACAAGTTGGTTGAAGCTGTTAAGAC

GAACTTCAATGAGAGATACGATGAGATTCGTCGTCATTGGGGAGGTGGTATCATGGGTGCCAAGTCGCAGGCTCGT---G

TTGTCATTACTGGACCTCGTGGCACACTTAGACGTGACTTCCGTCACATGGCCGTAAGGACGGTGTGCTCCCACATTGAA

AACATGATCAAGGGTGTGACTAAGGGCTACAGGTACAAGATGAGGTCTGTGTATGCCCATTTCCCTATCAACATTAGCAT

CACAGAAGACAACACCATGGTTCAGATCCGCAATTTCTTGGGCGAGAAGTACACTCGCAATGTGAAGATGTTGCCAGGTG

TTACCTGTAAGGATGAATTCATCCTGGAAGGCAATGACATTGAGCTGGTTTCAAGATCAGCTGCTTTGATCCATCAATCA

ACAAAAGTG---AAGCCCTATCCAAAGTCACGTTTCTGTAGAGGTGTGCCTGATCCCAAGATCCGTATCTATGACTTGGG

CAGGAAGAAGGCTCGTGTAGACGAGTTCCCTCTGTGTGTGCATCTGGTATCGGATGAACTGGAGCAGATATCATCCGAAG

CATTGGAGGCTGGCCGTATCTGTGCCAACAAATACATGGTGAAGAACTGTGGCAAAGATGCCTTCCATCTGAGAGTCCGG

GTGCATCCTTTCCATGTGCTGCGCATAAACAAGATGTTGTCATGTGCTGGAGCTGATAGGCTTCAGACAGGCATGCGTGG

TGCTTTTGGTAAGCCACAGGGTACAGTGGCTCGTGTACGTATTGGCCAGGCTCTGATGTCGGTACGCTGTAAAGATCAGC

ACCAAGAAAAGGCTGTGGAAGCTCTGAGGAGGGCCAAGTTCAAGTATCCAGGCAGGCAAAAGATCTACATCAGCCGTAAA

TGGGGCTTCACT---TTCACAGAGAGCGTTGAACTTCAGATATCACTGAAGAACTATGATCCTCAAAAAGACAAGCGTTT

TGCTGGCACTGTCAAGTTGAAGTACATCCCACGTCCAAAGATGAAGGTCTGTGTTTTGGGTGACCAGCAGCATTGTGATG

AAGCTAAAGCCAATGACATCCCATGTATGGATGTAGAGGCCCTGAAGAAGCTTAACAAGAACAAGAAACTTGTGAAAAAG

CTGGCCAAGCGATATGATGCTTTTATCGCCTCGGACAGTTTGATCAAACAGATTCCTCGTATTCTTGGTCCTGGCTTGAA

CAAGGCTGGTAAATTTCCAACCATGATCACTCATTCTGACTCCATGGTTAGCAAGGTTAATGAAGTGAAGGCCACCATCA

GATTCCAGATGAAGAAGGTGTTGTGTCTTGCTGTGGCTGTCGGTCATGTCAACATGAGCACAGAGGAGTTGGTTTCCAAC

ATCATGATGGCCATCAACTTCCTCGTCTCGCTGCTGAAGAAGAACTGGCAGAATGTACGAGCTCTGTACATCAAGTCCAC

CATGGGCCATGCATACCGCATTTAC---GTATGCCTGAGGGCTGTGGGTGGTGAAGTGGGTGCTACGTCTTCGTTGGCTC

CCAAAATTGGTCCACTCGGTCTGTCTCCAAAGAAGGTTGGAGATGATATTGCCAAGGCAACACAGGACTGGAAAGGTTTG

AGAATAACAGTCCAGTTAACAATCCAAAACCGTCAAGCCAAGGTGTCTGTGGTACCCACAGCCTCGGCTCTTGTTATCAA

GGCATTGAAGGAACCACCACGTGACAGAAAGAAGGTGAAACATGTCAAGCACAGTGGTAATATTTCTATGGATGAAATCT

ACACCATTGCCCGTACTATGAGGCAAAGGAGTATGGCAAGGACATTCAAAGGCACTGTCAAAGAAATCTTAGGTACTGCT

CAGTCTGTCGGTTGCACTGTTGATGGTCAGCATCCT---AACAACGTTGTTCCTAATGCCCACTTTCACAAGGACTGGCA

GCGCTATGTGAAGTGCTGGTTCAACCAGCCTGCTCGTAAGAAGAGGAGGAGGAGGAAGCGTATTGAGAAGGCACGGAAGA

TAGCTCCTAGACCGCTTCATACCCTCAGACCGATCGTACGCTGTCAGACCGTCCAATACAACACTCGTGTTCGTCTTGGA

CGGGGATTTACTCTTGAAGAGCTCAAGGCTGGAATCAACAAGCGTGAAGCCAGGACAATTGGCATCTCTGTAGACTTCCG

ACGTAAGAATCGATCAGTTGAGTCACTACAGCATAATGTCCAGCGTCTGAAGGAATATCGCAGCAAATTGATCATCTTCC

CAAGGAAGCGCGGCAAACCAAAGAAGGGAGATGCCACTGAGGAAGAGATGAAGATGGCAAAACAGGTCCTAGGAACTGTC

ATG---GATGCCCGCGGGCATCTGTTGGGCCGTCTCGCCGCAGTTGTCGCTAAAAATATCCTTCAAGGTCAGAGGATAGT

GGTAGTACGATGCGAAGGTATCAACATTTCAGGAAGCTTCTACAGGAACAAGTTAAAATACTTGGCTTTCTTGCGGAAGC

GAATGAACACTAATCCGAGCCGTGGTCCTTTCCACTTCCGAGCTCCAAGCAAGATATTCTGGAGAACTGTGCGTGGCATG

TTGCCACACAAGCTTACTCGTGGAAAGGTGGCTTTGTCCCACCTGAAGGTGTTTGAGGGAATTCCACCTCCATATGACAA

GAAGAAACGCATGGTAGTGCCCTCAGCTCTGAGAGTCCTTAGACTGAAGGCTAGACGCAAGTACTGTGTTCTTGGACGCC

TTTCCCATGAGGTTGGTTGGAAGTACCAGAATGTGATTGAGACTCTAGAAAGCAAAAGGAAGGTGAAGTCCTCC---ACG

TTCAAGCGTTTTGTGGAGATTGGCCGTGTGGCTTACATTGCCACTGGACCAGATGCAGGCAAACTGGTTGCCATTGTGGA

TGTTGTTGATCAGAATCGGGTGCTAGTTGATGGACCATGCACTGGAGTAGGCCGTAGGATTCTGAACCTGAAGCAGCTTG

ATCTAACAAACTTTGTCCTGAAGTTCTCGCACACTGCCAGGACAAGAGTCGTGCGAAAAGAATGGGAGAAGGCCGAGATT

GACAAGAAGTGGGCCGAGACCACCTGGGCCAAGAAGATGGCAAACAAAGAGAAGAGAGCTGCCCTGACTGACTATGAGCG

TTTCAAGCTTATGAAGGCCAAGCAG---ATGACTCGCTACAGTTTAGATCCAGAAAATGCTACGAAATCTGCTAAGGCTA

GAGGGTCTCATCTCCGAGTACATTTCAAGAACACAAGAGAGACTGCTCAGACCATAAAGCATATGCATCTGCGACGTGCT

GTCAGCTTTCTGAAGAATGTAAAAGCTCACAAGGAATGTGTTCCATTCCGCAGATACAATGGTGGAGTCGGTCGCTGTGC

CCAGGCAAAGAACTGGAAGACTACACAAGGCCGTTGGCCACAGAAGAGTGCTGAATTTCTTCTTCAGCTGCTTAAGAATG

CAGAAAGCAATGCTGAATTCAAGGGTTTAGATACTGATCATCTAGTAATTGAACACATCCAAGTGAATAGGGCTCCAAAA

ATGAGAAGACGAACATACAGAGCTCATGGAAGAATTAACTACATGAGTAGTCCATGCCACGTTGAAGTTATCCTT---GC

CAAAAACTTGTATATCATCGCTGTACAGGGAATTAAAGGTCGTTTGAACCGTTTGCCTGCTGCCGCCTCTGGTGACATGG

TTGTTGCTACTGTGAAGAAGGGAAAACCCGAGCTGAGGAAAAAAGTTATACCAGCGGTAGTTATCAGACAGAGAAAGCCA

ATACGACGGAAAAATGGAGTAGTCATATACTTTGAAGACAATGCTGGGGTTATAGTAAACAATAAAGGCGAAATGAAAGG

GTCAGCTATCAATGGTCCTGTGGCAAAGGAATGTGCAGACCTGTGGCCAAGGATTGCATCCAATGCTAGCTCCATA---T

TGGTAAAGTTGTATCGCTTTCTGGCCAGACGGACAGGATGCAAGTTTAACAGAATAATTCTGAAGAGACTTTTCATGAGC

AAGACTAACAGGGCGCCTATGTCTGCTTCAACTTTGAGACAGATGAAGAAGCCTGGACAAGATAAGATAGCTGTTTGTGT

GGGCACAATTACTGATGACTCGCGTGCCTACAAGTTTCCAAAGCTGAAGGTCTGTGCCCTGCGTGTCACTCGTGCTGCTC

GTGCTCGTATCCTGAAGGCTGGTGGTGAATTGATGACCTTTGACCAGTTGGCGCTGAAGTCTCCCAGAGGAAAGAACACT

GTACTTCTACAAGGTCCAC

>Paralv

TTTGTGAAAGTCGTCAAAAATAAACAGTACTTCAAAAGATACCAGGTTAAATTCAAGAGGAGGCGAGAGGGCAGGACTGA

TTACTATGCTCGTAAACGTCTGATATTCCAAGACAAAAACAAGTACAACACGCCCAAGTATCGAATGATAGTAAGATTTA

CCAACACTGACATCATTTGCCAGATTGCTTATGCCAGGATAGAAGGTGACGTGGTCATCTGTGCTGCGTATGCCCACGAA

CTTCCTCATTATGGGGTCAAGGTTGGACTGACAAACTACGCAGCTGCATATTGTGTCGGTCTGTTATTGGCACGAAGAAT

GCTGAAGAAGTTCGGTTTAGACACCATCTACGCTGGCAATCTTGAGGTAGATGGAGATGAGTACATTGTAGAGCCGGTAG

ATGGAGAGCCAGCACCTTTCCGGTGCTACCTCGATGTGGGTTTGGCCAGAACGAGCACCGGCGCCAAGGTTTTTGGTGCA

CTGAAGGGTGCTGCTGATGGTGGCCTGAACATCCCTAAAGCAGACGTACATCGCATGCACATCCTTGGTCAGCATGTTGC

AGACTACATGCGTCTACTGCAGCAAGACGATGAAGATGCTTACAAGAAGCAGTTCTCCCAGTTCATCAAATGTGGAGTCA

CTCCTGACTCTATGGAGGAGATGTACAAGAAGTGTCATGCCAACATC---CGTCAGGCCAGGAAACATGGAAACTTCTAT

GTGAGTCCGGAGGCACAGGTTGCATTTGTGATGAGGATCAGAGGTATTAATGGCATTCTTCCACGACCCCGTAAGGTGAT

GAAACTGTTACTTCTGCTGCAGATCAACAATGGTACATTTGTTAGGTTAAACAAAGCATCATTACACATGTTGAGGATTG

CTGACCCTTACATTACGTGGGGCTATCCAAACCTAAAGAGTGTGCGCGAGTTGATATACAAACGTGGCTATGGTAGGATT

GATGGTCGTAGGATAGCTCTGACAGATAATCTGATTGAGAAGAAGCTTATAATCTGTATTGAGGACTTGATCCATGAGAT

ATACACATGTGGTCCTCACTTCAAGCAGGCCAACATCTTTCTGTGGACCTTCAAACTCAATACACCAACTGGCGGCTGGG

TGAAGAATAATAACCACTTTGTGGATGGTGGCGACTATTGTAACAGGGAA---GTCAATCCACTATTTGAAAAGAGACCC

AAAAACTTTGGAATAGGTCAAGATATTCAGCCACAGAGGGATTTGAGTCGATTTGTAAAATGGCCAAAGTACATCCGCCT

TCAGCGACAGAGAGCCATACTTTATCAGCGACTGAAGGTCCCGCCACCAATCAATCAATTTACACAAACTTTGGACAGAC

AGACAGCCACACAACTGTTCCGATTGCTCGACCGTTATAGGCCGGAGAGCAGGCAGGAGAAAAAGATCAGGCTAAGACTG

CGGGCAGAAGAACGCGCAAAGGGTAAGCCGGATGCACCGACCAAACGTCCTCCTATCGTCAGGTCCGGAGTTAACACGGT

CACTACCCTGGTCGAGCAAAAGAAGGCACAGCTAGTGATCATCGCACATGACATCGATCCAGTTGAGCTTGTTCTGTTTT

TACCCTCTCTGTGTCGTAAGATGAATGTACCATACTGTATTGTGAAAGGAAAGGCTCGTCTGGGCCGTGTGGTACGTCGT

AAGACAGCTACGTGTCTGGCACTGACTCAAGTTAACGAAGACAAGTCGTCTCTAAACAAGTTGGTTGAAGCTGTTAGAAC

GAACTTCAATGAGAGATTTGATGAGATACGTCGTCACTGGGGTGGAGGGATAATGGGTGCCAAGTCTCAGGCTCGT---G

TGGTGATCAAGGGTCCTCGTGGTACCCTGCGTCGCAACTTTCATCACATGGCTGTGAGAACAGTTTGCTCCCACATAGAA

AACATGATCAACGGTGTCATGAAGGGTTACAGATACAAGATGAGGTCTGTCTATGCCCATTTCCCCATCAACATCACTGT

CCTGGAAAACAACACAGTAGTTCAGATACGTAACTTCTTGGGTGAAAAGTTCACCCGTAATGTAAAAATGATGCCAGGGG

TTACTTGCAAGGATGAGTTCATTCTTGAGGGCAATGACATCGAGCTGGTTTCACGATCAGCTGCATTGATTCATCAGTCA

ACAAAGGTG---AAACCCTATCCAAAATCTCGCTTCTGTCGAGGTGTTCCTGATCCTAAGATCCGCATCTATGATCTAGG

TAGAAAAAAGGCTCGTGTTGATGAGTTTCCTCTCTGTGTTCATCTCGTGTCTGATGAACTTGAGCAGATATCTTCCGAGG

CTCTGGAAGCTGGCCGTATCTGTGCCAACAAGTACATGGTGAAGAACTGTGGAAAAGATGCTTTCCACCTCAGAGTGCGG

GTCCATCCCTTCCATGTACTGCGAATAAACAAAATGTTGTCGTGCGCTGGAGCTGATAGGCTCCAGACTGGCATGCGTGG

TGCATTTGGCAAACCACAAGGTACAGTAGCTCGTGTTAGGATTGGTCAGGCCCTGATGTCGATACGCTGCAAAGATCAAC

ATCAGGAGAAGGCTGTAGAGGCACTAAGGAGGGCCAAGTTTAAATATCCAGGACGACAGAAGATCTACATCAGCCGCAAG

TGGGGCTTCACA---TCTACAGAGAGTGTTGAGCTCCAGATCTCGCTGAAGAACTATGATCCTCAGAAGGATAAGCGTTT

TGCTGGCACTGTCAAATTAAAACACATCCCACGTCCGAAGATGAAGGTGTGTGTGCTCGGCGATCAGCAGCATTGTGATG

AAGCAAAGGCTAACGAGGTTCCTTGTATGGACGTTGAAGCACTGAAGAAATTGAACAAAAATAAGAAACTGGTGAAAAAA

CTAGCCAAGAAGTTTGATGCCTTCATTGCTTCAGACAGTTTAATCAAGCAGATCCCCCGTATAGTGGGTCCTGGCCTGAA

CAAAGCAGGCAAATTTCCTACCATGTTAACTCATTCTGATTCCATGATCAGCAAAGTGAATGAGGTTAAAGCCACAATCC

GGTTTCAAATGAAGAAGGTGTTGTGTTTGGCAGTTGCTGTTGGTCATGTTAATATGAGCACAGAGGAGCTGGTGTCCAAC

ATCATGATGGCAATCAACTTCCTCGTCTCACTGCTGAAGAAAAACTGGCAGAATGTCCGTGCGCTCTACATCAAATCCAC

CATGGGACCTGCTCATCGTATCTAC---GTTTGTTTACGAGCTGTCGGTGGAGAGGTTGGTGCCACATCATCGTTAGCGC

CAAAGATTGGCCCTCTTGGTCTGTCTCCCAGAAAGGTAGGAGATGACATCGCCAAGGCCACCCAGGACTGGAAGGGCTTG

AGAATCACTGTTCAGTTGACGATCCAGAACCGACAGGCAAAAGTATCTGTTGTGCCGACAGCATCAGCACTCGTCATAAA

GGCTCTGAAGGAGGCACCTCGCGACAGGAAGAAGTTCAAGCACGTCAAACACAGTGGAAACGTTACCATGGAGGAGATAT

ACACCATCGCACGTACCATGAGGGCAAGGAGTATGGCGCGAACCTTCCGTGGTACCGTGAAAGAGATCCTGGGGACGGCT

CAATCCGTTGGCTGTACTGTCGATGGTATGCATCCT---AATAATGTGGTGCCAAATGCCCATTTCCATAAAGACTGGCA

GCGGTATGTGAAGTGTTGGTTCAATCAGCCCGCTCGTAAAAAGAGGAGGAGGAACAAGCGTATCGAGAAGGGTAGAAAAA

TTGCCCCTAGACCTCTACACACACTCAGACCCGTCATACGTTGTCCAACTGTTAGATACAATACACGCCTTCGTTTGGGA

AGAGGTTTCACTCTGGATGAGCTGAAGGCAGGATTAAACAAATATCAGGCAAAGACCATTGGTATATCTGTGGACTATCG

TCGTAGGAACCGTTCTGTTGAGTCATTACAGCAGAATGTTCAGCGTTTGAAGGAATACCGCAGCAAGTTGATCGTCTTCC

CAAGGAAGATGAGTCAGCCAAAGAAGGGTGATGCAACTGAGGAGGAAATGAAAATGGCAACACAGGTAGTTGGTACTGTA

ATG---GATGCCCGTGGGCATCTGCTTGGCCGTCTTGCATCAGTAGTTGCAAAAACCATCCTCCAAGGACAAAGAGTGGT

GGTGGTGCGATGTGAAGGTATAAATATTTCGGGCAGTTTTTACAGGAACAAACTGAAGTACCTTGCTTTCCTAAGGAAAC

GAATGAACACCAATCCAAGCCGTGGCCCATACCACTTCCGTGCCCCAAGTAAAATATTTTGGAGAACTGTGCGTGGTATG

TTGCCACATAAGCTGACACGTGGTAAGCAAGCTCTGGACCGTCTCAAGGTCTTTGAGGGCATCCCTCCACCATTTGACAA

GCAGAAACGTATGGTGGTTCCCTCGGCTTTGAAGGTCCTTCGACTTAAAGCTAGACGCAAGTCATGTGTTCTTGGCCGCC

TGTCACATGAAGTGGGCTGGAAGTACCAGAATGTCATCACTACATTAGAAAACAAAAGAAAGCTCAAGTCCACT---ACA

TTCACACGTTTTGTAGAAGTAGGCCGGATAGCCTATGTGGCCTTTGGACCAGATGAAGGCAAACTGGTGGCCATCGTCGA

TGTTGTTGATCAGAATCGGGTGTTAGTTGATGGACCGTGTAGTGGTGTGCCTCGTAAGGTCGTTAATCTGAAGCAGCTCC

ATTTGACGAAGTTCTTGATCAAGTTTACACACAGTACCAGGACGAGGGTTGTACGAAAAGCCTGGGAGGATGAGAAGATC

AACGAGAAATGGGCAGAAACCACGTGGGCAAAGAAGATAGCAAGCAGAGAGAGGAGAACACAACTAACAGACTTTGATCG

GTTTAAGCTGATGAAAGCCAAACAA---ATGACTCGCTACAGCTTAGATCCTGAAAATGCCACAAAATCTGCCAAGGCAA

GGGGATCTCATCTGCGTGTTCATTTTAAGAACACACGAGAAACGGCACAGACAATAAAACACATGCATCTGCGTCGTGCT

GTCAGCTTCCTGAAGAATGTAAAGGGACACAAAGAGTGTGTTCCATTCCGTAGATATAATGGTGGCATTGGTCGATGTGC

TCAGGCAAAGAATTGGAAGGCAACTCAAGGTCGTTGGCCACAGAAGAGTGCTGAATTTCTGCTTCAATTACTGAAAAATG

CTGAAAGTAATGCAGAATTTAAGGGTCTGGATACTGACCATCTTGTAATTGAACATATCCAGGTCAACAGGGCACCAAAG

ATGAGGCGAAGAACATACAGAGCTCATGGCAGGATTAATTACATGAGTAGCCCATGCCACGTCGAAGTGATCCTC---GC

CAAAAACTTGTACATTATTGCCGTGCAGGGCATAAAGGGACGTCTGAATCGTTTGCCAGCAGCTGCGTCTGGTGATATGG

TTGTCGCCACTGTCAAGAAGGGAAAGCCAGAGTTAAGAAAGAAAGTTATTCCTGCTGTGGTGATACGACAAAGAAAGCCT

ATACGACGGAAGAACGGTATTGTTATTTACTTCGAAGATAATGCAGGTGTTATAGTCAACAACAAGGGGGAGATGAAAGG

TTCAGCGATCAATGGTCCTGTGGCGAAGGAGTGTGCTGATCTGTGGCCAAGAATTGCTTCTAATGCCAGCTCAATA---C

TTGTAAAGTTGTATCGCTTCTTGGCTAGGAGGACAGGATGTAGGTTTAACAAAATAATTCTGAAAAGGTTATTTATGAGC

AAGACCAACAGAGCACCTATGTCTGTGGCATCTCTGAGACACATGAAGAAACCTGGTGACAACAAGATGGCTGTTTGTGT

TGGCACGATTACTGATGATGTGCGCATTTACAAACTTCCCAAGTTAAAGATATGTGCCCTGCGTGTTACAGGTCCTGCTC

GTGCACATATTTTGAAGGCTGGTGGTGAAATAATTACCTTTGATCAGCTTGCGTTGAAGGCTCCACGTGGACAGAACACT

GTCCTACTACAAGGCCCAC

>Argope

TTCGTCAAAGTAGTGAAGAACAAGGCATACTTTAAGCGATTTCAAGTGAAATTCAGGAGGAGGCGTGAGGGCAAGACAGA

TTTCTATGCCAGGAAACGCCTCGTCATTCAAGAGAAAAACAAGTACAATACACCCAAATACAGGCTTATTGTTCGCTTCA

CCAACAAGGACATCATATGCCAGATAGCATACGCCAGGATCGAAGGTGATCACATTATTTGCAGTGCATATGCACATGAA

CTGCCAAGATATGGCGTGAAGGTAGGGCTGACCAACTATTCTGCTGCTTACTGCACCGGATTGCTGCTTGCAAGAAGGCT

TCTTAAGCAGTTTAAACTGGACTCCGTGTACGCTGGCCAGGAAGAGGTGGACGGTGATGAGTACTACGTTGAGGATGTGG

ACGGTGAGGCTGGTGCCTTCAGGTGTTACCTGGACGCTGGTCTAGTCCGCACTTCCACTGGTGCACGTGTCTTTGGTGCA

TTGAAAGGTGCAGTGGATGGAGGATTAGACATTCCCAACGCCGAGGTACACCGTAACCATATATTTGGTAAACATGTAGC

TGGATATATGGCCCATCTCCAGGAGGAGGATGAAGAAGCCTACAAGAGGCAGTTCTCTAGGTTCATCAAGAACGGCCTCA

ATTCAGAAAATCTTGAGGAGATGTACAAGAAAGGTCATGCTGCCATC---AGAGAAGCAAGGAAACACAACAACTTCTAT

GTACCCGACGAACCCAAACTTGCATTTGTTATGAGAATCAGAGGTATCAACGGTGTCCATCCAAGACCCAGGAAGGTTAT

GCAGCTGTTCCGTCTGAGACAGATCAACAACGGTGTATTCATTCGTCTGAACAAGGCCACTATCAACATGCTCAGAATCG

CCGAGCCTTTCATTACATGGGGATACCCCAACCTGAAGAGTGTGAAGGAGCTGGTTTACAAACGTGGTTACGCCAAGGTT

GATGGAAGGAGAGTACCACTCACAGACAACATCATTGAGGAAAAGTTAATCATCTGTATGGAGGACTTGATCCACGAGAT

CTACACAGTCGGACCCAACTTCAAGCCCGCCACCAACTTCCTGTGGCACTTCAAGATGAACACGCCCAACGGTGGCTGGA

AGAGGAAGTACAACCACTACAATGACGGTGGTGACTTCGGCTGTCGTGAC---GTTAATCCCCTTATTGAGAAACGACCA

AGAAACTTTGGAATTGGTCAGGATATTCAGCCCAAGAGAGATCTTAGCCGTTTTGTCAAGTGGCCCAAGTACATCCGTCT

CCAGAGACAAAAGGCAGTACTCCTACAACGTCTGAAGGTTCCTCCACCAATCAACCAGTTCAAACAGACTTTAGATGGAA

AGACCGCAACACAAGTTTTCCGTCTAATGGACAAGTACAAGCCAGAATCGAAACAGGCCAGGAAGGCTAGGTTAAAGGCT

CGTGCTGAGCAGCGTGCCAGCGGTGGTGAAGATGTACCAACAAAGCGTCCCCCAGTTGTGAGGTCAGGAGTTAACAACAT

CACTTGTCTAGTGGAACAAAAAAAAGCACAGTTGGTCGTCATCGCCCATGATGTGGATCCATTAGAAATTGTGTTGTTCC

TGCCCGCTCTCTGCAGAAAAATGGGAGTTCCCTACTGTATTGTGAAAGGAAAGGCACGTTTAGGACGTGTTGTCAACAGG

AAAAATGCCACCTGTCTGGCTCTCACAAGTGTCAACGAGGACAAGTCATCACTCAGTAAATTGGTTGAGGCTGTCAAGAC

CAACTTCAATGAAAGACATGAAGAGATCCGCCGTCACTGGGGTGGAGGACAGATGGGCAGCAAGTCCCAGGCCCGT---G

TCACTATCACTGGGCCGAGAGGTACACTCACCAGAAATTTCCGTCATCTGGCAGTCAGAACTGTGTGCAGTCACATCGAA

AACATGATGAAGGGCGTCACCAAGGGTTACTTGTACAAGATGAGATCTGTATATGCTCATTTCCCGATCAACATTGCTAT

CCTTGAAGGCGGACACTCTGTGGAGGTCAGGAATTTCTTGGGTGAGAAATACACCCGTAAAGTCAACATGTTGCCTGGAG

TGACCTTCAAGGACGAATTCTTCATCCAGGGAAATGACATCGAGCTGGTATCCCAGTCAGCTGCTCTGGTCCAACAGTCT

ACTACAGTA---AAGCCATACCCCAAGTCAAGGTTTTGTCGTGGTGTCCCAGATCCCAAGATCCGTATTTTTGATCTTGG

CAAGAAAAAGGCCAGGGTAGACGAATTCTCATTATGTGTGCATCTTGTCTCTGACGAGTATGAACAGCTGTCATCTGAGG

CATTGGAAGCCAGCCGTATCTGTGCCAACAAATACCTTGTGAAGAACTGTGGCAAGGACTCCTTCCATTTACGAATGCGA

GTGCATCCCTTCCACGTTTGCAGAATCAACAAGATGTTGTCGTGTGCTGGAGCTGATAGGCTCCAGACAGGGATGCGTGG

TGCCTTCGGCAAACCCCAAGGAACCGTAGCCCGTGTGCACATTGGTCAGCCCATTATGTCCTGCCGTGCCAGGGAAACCA

ACCAGGCTGCTGTCATCGAGGCTCTCAGGAGAGCAAAGTTCAAGTTCCCAGGCAGACAGAAGATCTTTGTCTCCAAGAAA

TGGGGCTTCACC---TTCACTGAATCCATTGAATTGCAGATTGGTTTGAAGAATTATGATCCACAGAAGGACAAGCGTTT

CTCTGGAACCATTCAGTTACGTCATATACCAAAACCTAAGATGAAGGTTTGCATTCTTGGTGATCAGATTCATTGTGATC

AAGCCAAGGCCAATGAAATGCCTTGCATGGATGCTGATATGCTAAAAAAATTAAACAAGGACAAGAAGCTTGTTAAGAAA

TTAGCCAAGAGATACGATGCTTTCCTGGCCTCTGAGACTCTGATTAAACAGATTCCCAGATTATTGGGACCAGGTTTAAA

CAAGGCTGGTAAATTCCCTACCCCAATCTCCCATAATCAATCCCTTGTCAGCAAGGTACAACAAGTAAAGGGAACCATCA

AATTTCAAATGAAAAAGGTCTTGTGTTTGTCTGTCTGTATTGGACACATCAACATGAGCCAAGAAGAAGTTTACACCAAT

GTCAATTTGGCCATTAACTTTTTGGTTTCCCTTTTGAAGAAGAATTGGCAGAACGTGAGAACTCTACACATCAAAAGTTC

CATGGGACCTGTCCAGAGAATTTAT---GTGTACGTGAAGGCTGTGGGAGGTGAAGTACCAGCAACATCATCCCTGGCTC

CAAAGATTGGTCCACTCGGTTTGTCCCCAAAGAAAATTGGTGACGACATCTCCAAAAGCACCCAGGAGTGGAAGGGACTG

AAGATTACCGTGAAGTTGGTCGTCCAGAACCGTCAGGCCAAGGTAGAAGTTGTGCCAAGCGCATCTTCTCTGGTGATCAA

AGCCCTCAAGGAACCCCCAAGGGACAGGAAGAAGGTGAAACATGTTAAACATAATGGCAACATCAGCATGGACGAAATCA

TCAGGATTGCTCGTATCATGAGGCCCAGGAGTATGGCCAGGAACTTAGCCGGAACCTGTAAGGAAATTCTTGGCACTGCC

CAGTCTGTTGGCTGTTCTGTGGAGAAATGCCCTCCT---GCAAATGTGGTGCCGAATGGGCATTTCCACAAGGATTGGCA

GCGTATGGTGAGGACATGGTTCAACCAGCCCATGAGAAAGAAAAGGAGGCACGAGACTCGTGTCAAGAAAGCTCACAGAA

TCGCACCTCGTCCAGTTGCAGGACTAAGACCCATTGTCCGATGCCCAACATTCAAATACAATACAAAACTTAGAGTTGGA

AAGGGATTCACACTTGATGAGCTGAAGGCAGGCATCAATAAGAGAATGGCACTTACCATTGGTATTGCTGTGGACTACAG

GAGGAGGAATAAGTCTGTTGAGTCACTCCAGCAGAATGTTCAGAGACTGAAGGAATACAGGAGCAAACTCATCCTCTTCC

CCAAGAAGCAGAGCAAACCCAGGAAGGGAGATGCTACAGCTGAAGAAATCAAGATGGCTAAGCAGTTGTCAAGCTCTGTT

ATG---GATGCCAGAGGGCATTTATTGGGTCGACTGGCAGCCTTAGTTGCAAAGACCATTTTGCAAGGGCAACGAGTCGT

GGTTTTGAGATGTGAAGGTATCAACATCTCTGGAAACTTCTACAGGAACAAGCTGAAGGTATTGAAATACCTGAAGCTGA

GGTGTAATGTGAAGCCTACAAGAGGACCTTACCATTTCAGGGCTCCAAGCAAGATGTTCTACAAGGCTGTGAAAGGTATG

TTGCCTCACAAACTTGCTAGAGGAAAGGAGGCCCTTGCCAGACTAAAGGTGTTTGAAGGAATTCCAGCTCCTTATGACAA

ACAGAAGAGGATGGTTGTTCCCTCCGCACTCAAAGTCCTAAGGATCCACCCAGGCAGAAAGTTCTGTGACCTCAACCGTC

TGGCCCATGAAGTTGGATGGAAGTATCAGACTGTGATTTCTACCCTTGAAGCCAAGAGGAAGGTCAAATCAAAG---GGC

CAGTTCCACTTCGTAGAAATTGGGCGGGTGGCCCTTTTTGCCTATGGCCCAGATAAAGGCAAACTCTGCGTCATCGTAGA

TGTTATTGACCAGAACAGGGCATTAGTTGATGGACCAGCCACAGGCGTTAAAAGGAAGCAGGTCAACTACAAATCTCTAC

ATCTCACACAGTTTAAGTTAAACATCGGTAGAGCCATGCGTACAGGAAACCTGCTGAAGGTCTGGAATAAAGAAAGCATG

CAGTCCAAGTGGGATGCTACATCCTGGGCCAAGAAGATTGCCAACAAGTCTCTTAGAAAATCACTATCAGACTTTGACCG

CTTCAAGTTGATGAAGGCCAAGCAA---ATGACACGATATTCGTCAGAGCCTGAGAACCCCACAAAGTCTGCCAAGGCTA

GGGGTTCATACCTCAGAGTTCACTTCAAGAACACCAGGGAAACGGCTCAGGCAATCAAGCGTATGCACATCTCGAGAGCC

ACCAAGTACCTTAAAAATGTGATCGACAAGAAGGAATGTATTCCCTTCCGCAGATTCAACGGTGGTGTTGGTAGGAATGC

ACAGACCAAGCCATTCAAAGCATCCCAGGGTAGATGGCCCAAGAAAAGTGCAGAGTTTTTGCTTCAGCTTTTGAAGAATG

CTGAGAGCAATGCTGAGTACAAGGGTCTTGACACCGACCATTTAGTCATTGAACACATCATGGTCAATGAGGCCCCAAAG

ATGAGACGACGAACCTACAGGGCTCACGGACGTATCAATTACATGAGCAGTCCTTGTCACATTGAAGTGATTCTA---GC

AAAGAACCTGTATGTTATTGCTGTAAAAGGCATCAAAGGTCGTCTGAACAGAATGCCAGCAGCTGGAGCAGGTGACATGA

TAGTAGCCACCGTCAAAAAGGGCAAGCCAGAACTTCGAAAAAAAGTAATGCCTGCTGTGGTGGTGCGGCAACGTAAATCA

GTACGGAGGAAGAACGGGGTGTTTATATACTTTGAAGACAATGCAGGGGTGATAGTGAATAACAAGGGGGAAATGAAAGG

GTCTGCTATCACTGGTCCTGTAGCGAAAGAATGTGCAGATTTATGGCCCCGTATCGCATCAAACGCCAGTTCTATT---T

TTGTCAAACTATACAGGTTCCTTGCCAGACGTACCAATGCCAAGTTTAACAAGATAGTGCTAAAAAGGCTCTTTATGAGC

AAGACCAACAGGCCACCAATGTCTTTGGCAAGAGTACGACTCATGAAAAAACCTGGTGATAGTAAGATGGCTGTAGTTGT

TGGAACTATCACTGAAGATTTAAGGTTATTGGAAGTTCCTAAACTTAAAATTTGTGCCCTGAAAGTCACAGATCGTGCTC

GTGCTCGTATCCTTAAATCTGGTGGCGAAATTATTACATTTGATCAGTTGGCACTCCGGTCACCCAAAGGACAAAACACA

GTACTGATGCAAGGAGCCC

>Crassos

TTTGTTAAAGTTGTCAAGAACAAAGCTTATTTCAAGCGTTTTCAAGTCAAGTTCAGGAGGAGGAGAGAGGGCAAGACAGA

TTTTTTTGCAAGAAAACGCCTTGTAATTCAAGAGAAAAATAAGTACAACACTCCAAAGTATCGAATGATTGTTAGATTTA

CCAACAAAGACATCATTTGTCAGATAGCGTACGCTCGTATTGAGGGGGATATTATTATCTGTGCTGCTTATTCCCATGAG

CTGCCTCGATACGGTGTCAAGGTCGGGCTTACTAACTATGCAGCTGCCTACTGTACTGGACTCCTGTTAGCCAGAAGACT

TCTACAAAAACTGAAGTTGGATGACATCTATGAGGGACAGAAGGAAGTTGACGGAGAACAGTTCAATGTTGAGGATGTAG

ATGAAAAACCAGGTGCATTCAGGTGCTACTTGGACGTTGGTCTCGTGCGCACAACCACAGGTGCTCGTGTGTTTGGAGCC

ATGAAAGGTGCTGCCGATGGTGGACTAGATATCCCAAGTGCTGAGGTCCACAGAAACCACATCTTTGGCCAACATGTTGC

CAACTACATGAGGTCCCTTCAAGATGAGGACGAAGATGCATTCAAGAAGCAGTTCTCCCAATTCATCAAAAATAAAATAA

CAGCTGACAATTTGGAGGCCATGTACAAAAAATGCCATGCTGCTATT---AGACAAGCCAGGAAGAATGGCAACTTCTAT

GTCCCTGATGAACCCAAGCTAGCTTTTGTGATGCGAATCAGAGGTATCAATGGTGTTCATCCCAAGCCAAGGAAGGTTAT

GCAGCTTTTCCGTTTGAGGCAGATTAACAATGGTGTTTTTGTGAGACTTAACAAGGCCACGATTCACATGTTGAGAATCG

CTGAACCCTTCATTGCATGGGGGTACCCCAACCTCAAGAGTGTGAGGGAATTGGTGTACAAAAGGGGATATGGAAAAGTT

GGAAATCAGCGTATTCCTCTCACAGACAATCTCATTGAGAAACACCTCATCATCTGTATGGAAGATTTGATCCACGAGAT

CTACACAGTTGGACCCAACTTCAAGTATGCTGCCAACTTCTTGTGGCCATTCAAACTGAACACACCTAATGGTGGATGGA

GAAGGAAGTACAACCACTTCAACGACGGTGGTGACTTTGGCCTGCGTGAA---GTTAACCCCCTGCTTGAAAAAAGACCA

CGAAACTATGGAATAGGTCAAGATATTCAGCCTAAGCGTGACTTGAGTCGCTTTGTAAGATGGCCCAAATACATCCGTCT

CCAGCGCCAACGAGCTGTGTTGATGCAGAGACTGAAGGTTCCCCCGCCAATCAACCAATTCAGTCAAACTCTTGACAGAC

AGACAGCTACTCAGCTGTTCCGTCTGATGGACAAGTACAAGCCTGAGTCTAAGCAACAGAAGAAGGCTCGTCTAAAGGCC

AGAGCTGAGGAACGAGCTGCTGGTAAGGAGGACAAGCCTACCAAACGACCCCCTGTCATGAGGTCTGGAGTCAACACAGT

GACTGCCCTTGTGGAACAGAAAAAGGCACAGCTGGTTGTCATTGCACATGATGTTGATCCTATTGAAGTCGTCATTTTCT

TACCAGCATTGTGTAGGAAGATGGGAGTCCCCTACTGCATCGTGAAAGGAAAAGCAAGACTAGGACGTGTCGTTTACAGG

AAAACTGCCACATGTCTGGCCTTCACAAATGTCAATGAGGACAAAAATGCATTGAGTAAACTCTGTGAAGCTGTCAAGAC

CAACTTCAATGACAGATATGACGAGATTCGTCGACACTGGGGAGGTGGACAGATGGGATCTAAATCTCAAGCTCGC---G

TAATCATCAAAGGACCTCGAGGAACTTTAACAAGAACCTTCCGTCACTTGCCTGTAAGGACTGTGTGCAGTCACATAGAG

AACATGATCAAGGGGGTTACGAAGGGCTTTCTGTACAAAATGAGGTCAGTGTATGCCCATTTCCCCATCAACTGTGCAGT

ACAAGAGGGTGGAGGTTCAGTGGAGATTAGGAACTTTTTGGGAGAAAAATTTGTCAGGAAGGTGAGAATGAGGGCTGGTG

TTGTCTGTAAGGACGAGCTGATCCTGGAAGGCAATGACATTGAGCTGGTGTCAAACTCATCTGCTCTGATCCAGCAGGCC

ACCACAGTA---AAACCATATCCCAAGTCCAGATTTTGTCGTGGTGTCCCAGATCCTAAGATCCGTATCTTTGATCTTGG

GAGGAAGAAGGCCAGGGTAGATGAATTTTCTCTGTGTGTGCATCTAGTTTCTGGTGAGTATGAACAGTTGTCATCAGAAG

CGTTAGAGGCTGGCCGTATCTGTGCCAACAAGTACTTGGTCAAGAACTGTGGTAAAGACGCTTTCCACATGCGGATACGA

GTGCACCCCTTCCACGTCATCAGAATCAACAAGATGTTGTCGTGTGCCGGAGCCGATAGGCTTCAGACAGGAATGAGAGG

AGCTTTTGGTAAGCCACAAGGAACCGTTGCTCGCGTACACATTGGACAGCCCATCATGTCCGTCCGTGCCCGTGAGAACC

ACCGGGCATCAGTGATAGAAGCTCTCCGTAGAGCCAAGTTCAAGTTCCCAGGACGTCAGAAGATCCACATTTCCAAGAAG

TGGGGCTTCACG---TTTACTCAATCCGTAGAACTTCAGATTGTGTTGAAAAATTATGATCCCCAAAAGGACAGGCGTTT

TGCTGGAACAGTGAGATTGAGGAATATTCCAAGACCCAAGATGAAGGTCTGTATCCTTGGAGATCAGATTCATTGTGACC

AAGCAAAAGCTAACAATTTGCCCTGCATGACTGCTGATGACCTGAAGAAGCTAAACAAGGACAAAAAATTGATCAAGAAA

TTGGCCAAGAAGTACGATGCCTTCCTTGCCTCTGATTCCTTGATCAAGCAGATTCCCAAGATCTGTGGGCCTGGATTTAA

CAGAGCTGGAAAGTTCCCTACCCAGGTATCCCATCAGGAATCCTTGGTACAGAAAGTGGAGGAAGTCAAGGCAACCATAA

AGTTCCAGCTTAAAAAAGTGCTGTGTTTGGCAACTTGTGTTGGACATGTAAACATGACCAAAGAGGAGTTGTATGGAAAT

ATTACCCTGGCCATCAACTTTTTGGTTTCCCTGCTGAAGAAGAACTGGCAGAACGTCAAGGCCTTGTACATCAAAGGAAC

CATGACTCCAGCTCAGAGAATCTAC---GTGTATGTGAAAGCAGTTGGAGGTGAAGTTCCAGCCACTTCATCCTTGGCTC

CTAAAATTGGTCCTCTGGGTTTGTCTCCAAAGAAGATTGGTGATGACATTGCCAAAGGCACAGCAGATTGGAAAGGCCTA

AAAATCACGGTGAAGCTTGTGGTCCAGAACCGTCAAGCCAAGGTAGAAGTGGTGCCTAGTGCTTCTTCACTCCTCATCAA

GAGCCTGAAGGAACCACCCAGGGATAGAAAGAAGGTTAAACATGTCAAGCACAGCGGAAACCTCTCTATGGATGACGTTA

TTGCTATTGCACGTCAAATGCGTCCAAGGAGTATGGCCAAAAATCTCGCTGGTACATGTAAAGAAATTTTAGGTACGGCT

CAGTCTGTTGGATGCTCCATTGAGAGATCTCCTCCT---AACAACATTATCCCAAATGGGCATTTCCATAAAGATTGGCA

ACGGTATGTGAGAAACTGGTTCAACCAACCAGCCAGGAAGAAGAGGCGACACGACAACAGAGTCAATAAGGCCAGACGTA

TCGCTCCCAGACCTGTGGCTGGACTGAGGCCCATTGTGAGATGTCCAACCTTCAAATACAACACTAAAATTAGGGCTGGC

AAGGGATTCACTCTGGATGAACTGAAGGCTGGGATCAACAAGAAAGTAGCTCTTACGATCGGGATTGCTGTGGACTACAG

GCGTAGAAACAAGAGCCTTGAATCTTTGCAGCAAAATGCCCAAAGACTGAAGGAGTACAAATCTAAACTGATCCTCTTCC

CCAAGAAACAGAGCAAACCCAAGAAGGGAGATGCTACAGAGGAAGAAATTAAGATGGCGACTCAGCTGTCTGGCCAGGTT

ATG---GATGCTCGAGGGCATCTTATGGGAAGACTTGCAGCAATTGTTGCCAAGACTGTACTGCAAGGTCAGCGAGTTGT

TGTTGTACGATGTGAAGGCATCAACATTTCTGGAAATTTCTACAGAAATAAATTGAAGTACTTGAAGTATCTGAGGTACA

GATGCAATGTGAACCCCTCGAGAGGACCCTTCCATTACCGTGCTCCTTCTAGGATCTTCTTCAAGGCTGTAAAGGGTATG

ATCCCACACACCACAACTCGTGGTAAAGATGCCCTTGGCCGTCTGAAGGTGTTTGAGGGTGTGCCACCCCCATATGACAA

ACAGAAGAGGAAGGTAGTTCCAGCTGCCCTCAGAATCCTCAGACTGAAACAAAACAGAAGGTTCTGTGATTTGAACGGAC

TTGCCCATGAAGTTGGCTGGAAATACCAGGGTGTTATTGGTACTCTTGAAGCCAAGAGAAAAGCCAGGTCAAAG---AGT

CAGTTCCGCTTTGTGGAAGTTGGCCGTGTGGCGTATATTTCCTATGGGCCCGATGCAGGAAAGCTCGCCGTTATTGTTGA

TGTTATAGACCGGAACAGGGCTCTCGTAGATGGACCCTGCTCTGGTGTATCCAGAAAACAAATGAACTTCAAGGCTCTTG

AGCTGACATCAATTGTGATGAAAATTCCACGAGGTCTGCGTCCAGGAGACCTGCGTAAGGCTTGGGAAAAGCAGGAAGTA

CAGAAGAAGTGGGATAGCACAACATGGGCACAGAAAATTGCAAATAAGAAGAAGCGTGCCCAGCTCACAGACTTTGACAG

ATTCAAGCTGATGAAGGCTAAACAG---ATGGTTCGATACGCTACAGAACCAGATAACCCAACCAAATCGGCCAAGGCCA

GGGGGTCTTATCTCCGTGTCCACTTTAAGAACACAAGAGAAACTGCACAGGCTATTAAGCGTATGCACGTTAAGAGAGCC

ACCAGATACCTAAAGAATGTGATGGCGAAGAAGGAGGTGGTTCCTTTCCGTAGATTCAGTGGTGGAGTGGGGCGTTGTGC

TCAGGCTAAGGCCTTCAAGCATTCCCAAGGTCGTTGGCCATCGAAAAGTGCTGAATTCTTGTTGCAGCTTTTGAAGAATG

CTGAGAGTAATGCCGAATACAAGGGATTGGATACAGATCACTTGGTCATTGAACACATCATGGTGAATGCTGCACCCAAG

ATGAGAAGGAGAACCTACCGTGCTCATGGTCGCATTAACTACATGAGCAGTCCCTGCCACATTGAACTGATCCTG---GC

CAAGAATCTGTTTGTGATCGCTGTCAGCGGTATCAAGGGTCGCCTCAACAGAATGCCAGCTGCTGGAGCTGGAGACATGT

TTGTTGCCACAGTCAAGAAAGGAAAGCCAGAACTTAGAAAAAAGGTTATGCCTGCTGTGGTGATACGACAGAGGAAACCT

GTTCGGAGGAAGAATGGGGTGTTCATATACTTCGAAGATAATGCGGGGGTCATAGTCAACAATAAGGGAGAAATGAAAGG

ATCAGCTATCACTGGACCTGTAGCTAAAGAATGTGCAGACTTATGGCCCCGTATTGCATCAAACGCCAGCTCCATT---C

TTGTTAAGTTGTACAGGTTCCTAGCTAGAAGAACTAATGCCAAGTTCAACAAGATTATCCTGAAGCGTCTGTTTATGAGC

AAGACCAACAGACCACCTCTGTCCGTTGCCAGACTGCGTCTGATGAAGAGAACAGGCGACAACAAAACTGCTGTGGTTGT

TGGCACTGTGACTGATGATCTTCGACTTTTAGACGTTCCCAAGCTTAAGTTGTGTGCTCTCAATGTGACAGAGAGAGCCA

GGGCTCGTATCCTGAAGTCTGGGGGACAGATCATCACTTTTGATCAGCTGGCTCTCAAGTCTCCCAAAGGACAAAATACA

GTGCTGATGCAAGGACCCA

>lumbri

TTCGTGAAAGTTGTCAAAAATAAGGCGTACTTCAAACGGTACCAGGTGAAATACAGGAGGCGACGAGAGGGTAAAACTGA

TTACCGTGCTCGTAAGAGACTAGTTGTGCAAGACAAGAACAAGTACAACACGCCGAAGTACCGAATGATTGTGCGCATTA

CCAACAAGGACATCATTTCTCAGATAGCGTATGCCAGAATCGAGGGTGACGTGGTTGTGTGCGCCGCCTACGCACACGAG

TTGCCCAAGTTTGGCATCAAGGTCGGGCTGACAAACTACTCCGCGGCTTATGCAACTGGACTTTTATTAGGACGCAGGAT

TCTAAAGAAATTCAACTTGGACGGGATCTACGGTGGGGCTGAGAAGGTGACGGGTGAGGAGTACCATGTGGAGTCCGTTG

ACGGGCAGCCGGGTGCGTTCCGCTGCTACCTGGACGTGGGTCTGGCCCGGACGACAACCGGGGCGAGGGTCTTTGGCGCC

CTGAAGGGGGCTGCTGATGCTGGTTTGGAAATACCCGACGCCGAGATTCACCGCAAGCACATCTTTGGCCATCATGTGGC

TGATTACATGAAGCTGCTGCAGCAGGACGATGAGGACAGCTACAAGAGACAGTTCTCCAAGTACATCTCCAACAACATTC

AGCCGGACAACATTGAAGAGATATACAAGAAGGCCCATGCTGCAATT---CGCCAGGCACGCAAGCACAACAACTTCTAC

GTTCCACAGGCAGCTAAGGTGGCTTTCGTCATTAGGATCAGAGGTATCAATGGAATCGCTCCAAAGCCGCGCAAAGTTCT

GCAACTTCTTCGCCTGCGTCAGATCAACAACGCTGTCTTCATCAAGCTGAATAAGGCGACAATCAACATGCTGCGCTTGG

CTGAACCATACATCGCTTGGGGATACCCCAACCTGAGTATCACAAAGAAACTTCTGTACAAGCGAGGTTTCGCGAAGGTT

GAAGGTAGAAGATTGCCGCTGTCCAACGAAATCATCGAAAGAAAGCTGCTGATCTGTGTGGAGGACGTTATTCATGAGCT

TCTGACGTGTGGACCGCTCTTCCAGAAGGCAAGCAACATGCTGTGGCCATTCAAGCTATCGAGTCCGAACGGTGGATGGA

GGAAGAAGGGACGTCACTACGTCGATGGCGGTGACTTTGGAAACCGTGAG---GTTAACCCTCTGTTCGAGAAGAAGTCG

AAGAATTTTGGAATTGGACAAGATATCCAGCCAAAGCGTGATCTGTCACGTTTTGTCCGCTGGCCAAAGAATGTGCGTCT

GCAACGTCAGAAGGCTATTCTCTACAAGCGTCTCAAAGTTCCTCCATCAATCAACCAGTTCACTCAAGCCCTGGATAGGC

AGACAGCCACCCAGTTGTTCCGTCTGATGGAAAAGTATCGTCCAGAGACGTACCAAGAGAAGATGAGGCGACTGAAGTCT

CGTGCCGAGGAGCGCGTCAAGGGCAAGGAAGATGTTCCGACGAAGCGTCCACCGGTCATCAGGTCCGGCATCAACACCAT

CACGTCTCTGATTGAACAGAAGAAAGCACAGCTGGTTGCCATTGCCAACGACGTGGAACCGATCGAGATCGTGTTGTTCC

TGCCTGCTCTGTGCAGGAAGATGGGTGTTCCTTATTGCATTGTCAAGAACAAGTCTCGTCTCGGCCGAGTTGTTCACCGC

AAAACGGTCACGAGCTTGGCTTTCACACAGGTTAACGAGGACAAAACCGCACTGACCAAGTTGGTTGAAGCTGTGAAGAC

CAATTTCAATGAACGTGGTGAAGAGATCCCGCAGCACTGGGGTGGTGGTGTCATGGGCCAGAAGTCTCAGGCTCGC---G

TGACTGTCAAGGGACCACGTGGGACCCTGAGACGTGACTTCCGTCACATGGCAGTCAAGACCGTCTGCTCTCATGTTCAG

AATATGATCAGAGGTGTCACTGAGGGATTCCGCTACAGGATGAGGTCTGTGTATGCTCACTTTCCCATCAACGTTGCAAT

CTCCGAAAGCAACACCGTCGTTGACATTCGTAACTTCCTGGGCGAGAAGTTCAACAGGCAGGTGCGAATGTTGCAAGGAG

TGACATGCAAGGACGAGTTCGTTTTGGAAGGAAACGACATTGAACTCGTTTCTCGTTCAGCTGCCTTGATCCAGCAGTCG

ACCACAGTG---AAGCCGTACCCTAAGTCTCGTTTCTGTCGAGGTGTTCCAGATGCAAAGATTCGTATCTTTGATTTGGG

AAGAAAGAGGGCGAAGGTGGACGAGTTCCCGCTCTGCATTCATTTAGTGTCCGATGAGTTTGAGCAGATCTCATCAGAGG

CGTTGGAAGCTGGACGTATCTGCGCCAACAAGTACCTGGTGAAGCACTGTGGAAAGGATGCGTTTCATCTGCGTGTCAGG

CTCCACCCGTTCCACATCATCCGCATCAACAAGATGTTGTCCTGTGCTGGAGCCGATAGGCTTCAGACTGGAATGAGAGG

TGCTTTTGGTAAGCCTCAGGGAACAGTTGCTCGCGTTGACATCGGACAGGTGATCATGTCGGTGCGTGCCAAGGACGGTC

ACAAAGAGAAGGTCATTGAGGCTCTGCGACGTGCCAAGTTCAAGTACCCAGGAAGACAAAAGATCCACGTGAGCCGCAAG

TGGGGATTCACG---TTCACAGAAAGTGTTGAACTTCAGATTTCCTTGAAGAACTATGATACCCAAAAGGACAAGCGTTT

CAGCGGAACCGTCAAGTTGCGTCACGTCCCACGGCCCAAGATGTCTGTCTGTGTACTCGGAGATCAGCAGCACTGTGACG

AGGCGAAGGCGAACAGTATTCCATGCATGGATCAGGACGCTTTGAAGAAGCTGAACAAGCAGAAGAAGCCCATCAAGAAG

CTTGCGAAGCAGTACGATGCTTTCCTTGCTTCGGATTCTCTCATCAAGCAGATCCCGCGTATCGTTGGTCCGGGTCTGAA

CAAGGCCGGCAAGTTTCCGTCTATGTTGACGCACAACGAATCCATGGTTGCGAAAATCGAGGATGTCCGTGCCACCATCA

AGTTCCAGATGAAGAAGGTTCTTTGTCTGGCTGTTGCTGTTGGTCATGTGGCCATGACTCCGGAGGAACTGGCGACGAAC

ATCACGATCGCAATCAACTTCCTCGTGTCTCTGCTGAAGAAGAACTGGCAGAACGTGCGAGCGCTGTACATCAAGAGCAC

CATGGGTCCATCCCAGCGCCTTTAC---GTCTGCCTTCGTGCCGTCGGTGGTGAAGTCGGTGCCACATCGGCTCTGGCTC

CAAAGATCGGTCCTTTGGGTCTGAGCCCCAAGAAGGTCGGTGAAGATATTGCGAAAGCCTCGCAGGACTGGAAAGGTCTG

CGCATTACTGTGCAGTTGACCATCCAGAATCGTCAGGCCACGATCGCTGTCATCCCATCGGCTTCCTCACTCATCATCAA

GGCCCTGAAAGAGCCGCCAAGGGACCGCAAGAAAGTTAAAAACATTAAGCACACTGGCAACCTGAGCCTGGATGAAGTCA

TTGCCATTGCCCGGACCATGAGGATACGCAGTATGGCGAAGGCGTTGTCTGGAACCGTAAAGGAAGTTCTTGGCACCTGC

CAATCTGTTGGATGCACGGTCGAGGGATCGCACCCT---AACAATGCAATTCCAAATGGCCATTTCCACAAGGATTGGCA

GACGCATGTGAAGACATGGTTCAACCAGCCAGCCCGCAAGGAGCGCCGAAGGGTCGCCCGACGCAAGAAGGCCCTTGCCA

TCGCTCCTCGGCCCGCTGCAGGACTGAGACCGCAAGTACGATGCCAGACTTTCAAATACAACACTCGTCTGCGTGAAGGA

AGAGGCTTCAGTCTCGACGAACTGAAGGCAGGCATCAACAAGAAGGAGGCTCGCACGATTGGAATCTCCGTTGATGTCCG

TCGTAGGAACAAGTCCGTGGAGTCGCTCCAGTTGAATGTCCAGCGCCTGAAGGAATATCGCAACAAGTTGATCCTCTTCC

CGAAGAAGCTGAGCAAGCCAAACAAGGGCGATGCCACGGAAGAAGAAATGAAGCTGGCGACCCAGTTGAAGGGCAAGCTG

CTT---GATGCGAAGGACCACCTGATGGGTCGTTTGGCCGCAACTGTGGCCAAGACCCTTCTGGAAGGTCAGCGCATTGT

TGTTTTGAGATGTGAGGAAATTAACATCTCCGGTAACTTCTACCGAAACAAACAAAAGTACCTGGATTTCTTGAGAAAAA

GAATGAACACAAACCCGTGTCGTGGTCCATACCACTTCCGTGCTCCAAGCAAAATCTTCTGGCGAACAGTTAGAGGAATG

TTGCCACACAAGCAGTACCGTGGAAAGGATGCGATGGAGAGGCTGAAGGTCTATGAAGGTATTCCTCCTCCGTACGACAA

GATTAAACGTATTATTGTTCCATCTGCTTTGAGGGTCATCCGCTTGAAGCCAAGGCGAAAGTACTGCAAGCTGGACCGTC

TTGCAAGTGAAGTTGGATGGAAGGCGTCAAAGGTGGTTGGAACCCTGGAGAAACGCCGAAAGCTGAAGGCCGCC---TCG

TTCAGACGATTCGTTGAAATTGGGAGAGTTGCCCGTGCGGTGTATGGACCAGACCAGGGCAAGCTTGTAGCCATTGTGGA

CGTAATTGACCAGAACAGGGCTTTGGTTGATGGTCCCTGTACACATGTTGCTAGAAAGTCAATGAACTTCAAGGAACTGG

AGCTGACTAATCTGAAGGCCAAGTTTCCCCACTCAGCCAAGACCGGAGTTGTGAAGAAGGCGTGGGAGAAAGATGAAATC

TCCAAGAAATGGGAAGAGAGCCACTTGGCCAAGAAGATCGCAGCTAAGGAAAAGAGGAAGACACTGACAGACTTTGAAAG

GTTTAAACTGATGAAGGCCAAGCAA---ATGACTCGTTACTCCCTCCCACCCGAGAACCCAGCCAAATCGGCTTTGGCTC

GTGGCTCACACCTTCGCGTTCACTTCAAGAACACGCGTGAGGCTGCCCAGGTGATCAAGCACATGCATCTGCGCCGTGCC

GTTGCATTCCTAAAGAATGTGATCGCACACAAGGAATGCGTACCGTACCGTCGCTTCACTGGTGGCGTTGGAAGGTGCGC

TCAGGCCAAGCAGTGGGGAGCCACACAGGGACGTTGGCCAAAGAAATCTGCGGAGTTTCTTCTTCAGATGCTGAAGAATG

CAGAGAGCAATGCAGAATTCAAGGGATTGGACACGGACCATCTGGTTATTGACCACATCCAGGTCAACCGTGCCCCAAAG

ATGAGAAGGCGTACCTACCGTGCTCACGGACGCATCACATACATGAGCAGTCCTTGCCACATAGAGATCATCCTG---GC

CAAGAACTTGTACATCATTTCCGTGCATGGAATTCGTGGTCGTCTTAACCGTCTGCCAGCTGCTGGCTGTGGTGACATGG

TCATGGCTACGGTGAAGAAGGGAAAGCCAGATCTACGGAAAAAGGTAATGCCTGCTGTGGTAATACGGCAACGTAAAACC

ATTCGTCGAAAGGACGGCTTGTTTATCTACTTTGAAGACAACGCAGGTGTGATAGTGAATAACAAAGGAGAAATGAAAGG

GTCTGCTATCACAGGACCAGTTGCGAAGGAGTGTGCCGACCTGTGGCCACGTATTGCATCGAACGCCAGCTGCATC---C

TTGTCAAGCTCTACCGATTCTTGGGTAGAAGAACTGGCTCGAAGTTCAACAAAGTTGTTCTTAAGCGACTGTTTATGAGC

AAGACAAACCGGCCTCCTCTTTCTCTGTCACGTTTGCGGCACATGAAGAAGACTGGAGAGGGAAAGATCGCTGTTGTCGT

TGGCACAGTCACTGACGATCCTAGGATCTTTGAAATTCCGAAGTTGAATGTGTGTGCTCTGCATGTCACCGATCGCGCAC

GGGCTCGCATTCTGAAAAGCGGAGGTCAGATCATTACATTTGATCAGTTGGCTTTGAAGAGTCCAAAGGGCCAGAAGAGT

GTCCTCCTACAAGGACCAC

>Areni

TTCGTGAAGGTTGTGAAGAACAAGGCATACTTCAAGAGGTTCCAGGTCAAGTTCAAGAGGAGGAGAGAGGGACGTACGGA

CTACTATGCCCGTAAGCGCCTTGTTATTCAAGAGAAGAATAAGTACAATACTCCTAAGTACCGCATGATTGTGCGCTTTA

CCAACAAGGATGTTACTTGCCAGATAGCGTACGCCAAGATTGAGGGAGACGTGATCGTGAGCGCTGCTTACGCCCATGAG

CTCCCCAGCTATGGCGTCAAGGTCGGGTTGACAAACTATGCCGCTGCCTACTGCACTGGGCTCCTGCTCGCCCGCAGGAC

CCTCGCAAAGTTCGGTCTGGATGGTATCTACGCCGGTAACTCGGATGTGAATGGTGATGAGTACCTGGTGGAGAGCATCG

ACGGACAGCCCGGAGCCTTCCGCTGCTTCCTCGATGTGGGTCTGGCCCGCACCAGCACTGGGGCCCGTGTCTTCGGCGCT

CTNCAGGGAGCTGTGGACGGTGGACTGGACATCCCCGATGCCAACGTGCACCGCCAGCACATCTTCGGCCAGCACGTGGC

TGACTACATGAAGCTGCTGCAGCAAGATGATGAGGATGTCTACAAGAGGCAGTTCTCCAAGTTCATCAAGCTCGGCATCG

AGCCAGACTCCATGGAGGAAATGTACAAGAAATGTCACGCCGCCATC---CGCCAGGCCAAGAAAAAGGACAACTTCTAT

GTGCCTGCCGAGGCCAAGATGGCTTTTGTCATGAGAATCCGTGGTATCAACGGCATCCACCCTCGTCCCCGGAAGGTGAT

GCAGCTGTTCCGCCTCAGGCAGATCAACAATGGAACTTTCCTCAAGCTCAACAAAGCGACGCTTCACATGTTGAGATTGG

CTGATCCCTACATCACTTGGGGATATCCCAACCTGAAGAGTGTGCGAGAGTTGATCTACAAGCGCGGCTACGGCAAGCTC

GACGGCAGGCGGATTCCCCTCACCGACAACATCATTGAGCAGAAACTTATCATCTGTATGGAGGATCTGATCCACGAGAT

CTTCACCGTGGGACCCCACTTCAAGGAGGCCAACAACTTCCTGTGGCACTTCAAGCTGAGCACCCCCACCGGCGGCTGGA

GGAAGAAGAACAACCACTTCACCGACGGCGGAGACTTCGGCAACCGGGAG---GTCAACCCTCTGTTTGAGAAGAGACCC

AAGAACTTTGGAATTGGGCAGGACATTCAGCCAAAGCGAGATCTTTCAAGATTTGTCAGATGGCCGAAGTATATCCGTCT

CCAACGCCAAAAAGCTGTATTGTTGCAGAGGCTGAAGGTTCCACCACCCATTAACCAGTTCAGTCAGACATTGGACAGGC

AAACAGCCACTCAAATCTTCCGCTTGCTGGACCGGTACAGGCCAGAGACCAAGCAGGAAAAGAAGGCACGTCTGCGTAAG

CGGGCTGAGGACCGGGCCAAGGGGAAGGATGACGTGCCCACCAAACGTCCGCCCATGGTTCGCTCTGGCGTCAACACTGT

TACTGCCTTGATTGAGCAGAAGAAGGCCCAAATGGTTATCATTGCTCATGATGTAGACCCCCTTGAGTTGGTCCTGTTCC

TGCCTGCTTTGTGCAGAAAGATGGCAGTGCCTTACTGCATCGTGAAGAACAAGGCCCGTCTTGGCCGCGTTGTCCACCGC

AAGACTGCCACTTGCCTCGCCCTCACCACTGTTAATGAGGACCGTTCTGCCCTAAACAAGGTGATGGATGCTGTCAAGAC

CAACTTCAACGACAGAGTGGACGAGATCCGCCGTCACTGGGGAGGCGGAGTGATGGGCGCCAAGTCTCAGGCTCGG---G

TGGTTGTGTCTGGGCCACGTGGGGTTCTGCGTCGCCAGTTCCAGCATCTTGCCGTACGCACTGTTTGCTCCCATATCCAG

AACATGATCATTGGTGTAACCAAGGGTTACAAGTACAAGATGAGGTCTGTGTACGCCCATTTCCCCATCAACGTTGCAAT

CTCTGAGACCAACACCATGGTGGAGGTTCGCAACTTCTTGGGTGAGAAGTTCACGCGCAGAGTGAACATGTTGCCGGGTG

TCATTTGTAAGGACGAGTTCATTCTGGAGGGAAACGACATTGAGCTCGTATCCAGATCGGCCGCCCTGATCCAGCAGTCG

ACCACCGTG---AAGCCTTACCCAAAGTCTCGGTTCTGCAGGGGTGTTCCTGACTCCAAGATCCGAATCTATGACTTGGG

CAGGAAGAAGGCGAGGGTAGACGACTTCCCTCTGTGCATTCATCTGGTGTCTGATGAGTTCGAACAGATCTCGTCTGAGG

CCCTGGAGGCTGGCCGCATCTGCGCCAACAAGTTCCTGGTGAAGCACTGCGGAAAGGATTCTTTCCATCTCCGCGTGCGC

GTCCACCCCTTCCACGTTCTGCGTATCAACAAGATGTTGTCATGCGCTGGAGCTGATAGGCTCCAGACTGGAATGAGAGG

TGCCTTCGGCAAGCCTCAGGGGACCGTGGCCCGTGTCAGCATCGGGCAGACCATTATGTCCGTTCGGGCCAAGGACCCAC

ACAAGGATAAGGTCATCGAGGCTTTGAGAAGAGCCAAGTTCAAGTACCCTGGCAGGCAAAAGATCTACCTGAGCCGTAAG

TGGGGCTTCACC---TTCCAGGAAACTGTGGAACTCCAAATCGTCCTGAAGAACTACGATCCCCAAAAGGACAAGCGTTT

CTCTGGCACCGTCAAGTTGAGGCACATCCCCAGGCCCAAGATGTCCGTTTGCGTCCTTGGAGACCAACAGCATTGTGATG

AAGCCAAGGCCAACAATGTTGCCTGCATGGATGTTGATGCTTTGAAGAAGCTGAACAAGAACAAGAAACTTGTCAAGAAG

CTTAGCAAATCCTATGATGCGTTTTTGGCTTCTGACTCGTTCATCAAGCAGATTCCACGTATTGTGGGGCCTGGGTTGAA

CAAGGCCGGCAAGTTCCCAGTCATGTTGACCCACCAAGAGTCCATGACTCAGAAGATTGATGAGGTCAAGGCCACCATCA

AGTTCCAGATGAAGAAGGTACTGTGTCTGGCTGTCGCCGTGGGCCACGTTGGCATGACCCAGGAAGAGCTCCTCTCCAAC

ACGATCCTGTCCATCAACTTCCTCGTGTCGCTGCTGAAGAAGAACTGGCAGAACGTGCGCGCCCTCTATATCAAGAGCAC

CATGGGCCCCTCTCAGCGCATCTAC---GTGTGCCTGAGGGCTGTTGGAGGTGAGGTTGGTGCGACATCAACCCTGGCCC

CAAAGATTGGTCCCCTTGGTCTGTCCCCCAAGAAGGTGGGAGATGACATCGCCAAGGCTACTCAGGAGTGGAAGGGTCTA

AAGATCACTGTCCAGTTGACGATTCAGAACCGTCAGGCCAAGGTCTCGGTGGTCCCCTCGGCGTCCTCGCTAGTCATCCG

GGCTCTCAAGGAGCCCCCACGTGACCGCAAGAAGCAGAAGAACATTAAGCACTCGGGAAACATTCCCTTCGACGAGATCC

TGGCCATTGCACGTACCATGCGTCCTCGCAGCATGTCCCGCAAGCTGTCTGGCGTCTGCAAGGAGATCCTCGGCACATGC

CAGTCCGTCGGCTGCACAGTAGACGGATGTCACCCC---AACAATATCATCCCCAACGCCCATTTCCACAAGGATTGGCA

GCGTTACGTACGCACATGGTTCAACCAGCCAGCCCGAAAGAAGAGGAGGCAGCAGAATAGAATCAAGAAAGCCCGCAAGG

TTGCCCCCAGGCCTGTAGGAGGATTGCGCCCCATCGTGAGATGCCCTACCTTCAAATACCACAGTAAAGTGAGGCTGGGA

CGTGGCTTCTCTCTTGAGGAGCTGAAGGCTGGTTTGGCCAAGCTTGAAGCCCGCTCTATTGGTATCTCTGTGGACCATCG

CCGCAAGAACAGGTCGGTTGAGTCCCTCCAGCTTAATGTACAGAGGCTGAAGGAATACAGGAGCAAGCTGATTGTCTTCC

CCAGGAAGGCATCCAAGCCTAAGAAGGGAGATGCCACTGAGGAGGAGATGAAGCTGGCGACCCAGCAGAAGGGCGCCGTC

ATG---GATGGTAGGGGTCACCTTATGGGTAGGCTCGCTACCCTTGTGGCCAAATCACTGTTGCAAGGTCAACGCGTTGT

GATTGTGAGATGTGAGGGAATCAATATTTCTGGTACCTTCTACAGGAACAAGTTGAAGTACCTGGCTTACCTTCGCAAGA

GGATGAACACAAACCCCTCCCGTGGCCCATTCCACTTCCGTGCTCCCAGCAAGATCTTCTACCGTGTTGTCAGAGGTATG

CTGCCCCACAAACAGCACCGTGGACGTGAGGCTCTTGGTCGCCTGAAGGTCTTTGAGGGTATCCCACCCCCGTATGACAA

ACAAAAGCGTCTCGTGGTCCCTTCTGCCCTGAAAGTACTGCGTCTTAAGCCCAGGAGAAAGTTCTGCGCTATGGGTCGCC

TCTCGCACGAGGTAGGATGGAGATACCAGACCGTGGTCAGCACACTGGAAAACAGGAGAAAGGCCAAGTCCACC---ACA

TACACACGTTTTGTAGAAGTAGGCCGTGTGGCCTATGTGGCCTTCGGCCCTGAAAAGGGCAAGTTGGTAGTCATCGTGAA

TATAATCGATCAAAACCGGGCTCTTGTGGATGGTCCATGCTCCGGGGTGGTGCGCCAGTCCATGAACTTCAAGCAGATGC

ACCTGACACCGTTTGTGCTGCCCCTGTCCTTTGGGGCCCGTACAGCTGTAACACGCAAGGCATGGGAGAAGGCTGAGATC

GACAAGAAGTGGGCTGAGAGCGCATGGGCCAAGAAGATTGCTGCTCGCGAAAAGAGGGCCGACTTGACAGACTTCGAGCG

TTTCAAGCTGATGAAGGTCAAACAG---ATGACTCGCTATTCCTTGGATCCCGAGAATGCGAATAAATCGGCCAAGGCAC

GTGGCTCTCACCTGCGGGTTCACTTCAAGAACACCCGTGAGACAGCTCAAGCCATCAAGCATATGCATCTGCGTCGTGCT

GTGAGCTACCTGAAGAATGTGACTGTCAAGAAAGAATGTGTCCCCTTCAGACGATACAATGGAGGTGTCGGCAGATGTTC

TCAGGCTAAGGCCTTCGGTGCCACACAAGGCAGATGGCCCAAGAAGAGTGCAGAGTTTCTCCTCCAGCTCTTGAAGAATG

CTGAGAGCAATGCAGACTACMAGGGTTTGGACACTGATCATCTGGTCATCGAGCACATTCAGGTGAACAGGGCCCCCAAG

ATGAGGAGGAGGACTTACCGCGCTCACGGCCGCATCAACTACATGTCCAGCCCCTGTCACATCGAGGTGATCCTG---GC

AAAAAACCTCTATGTGATTGCTGTGCAGGGAATCAAGGGTCGTCTGAACCGTTTGCCAGCGGCCGGCAGTGGTGACATGA

TTGTGGCCACTGTGAAGAAGGGAAAGCCCGAACTTCGGAAAAAAGTAATTCCTGCTGTGGTCATTCGGCAACGGAAACCT

ATACGGCGGAAGGACGGGGTGTTTATATACTTTGAAGACAATGCAGGGGTGATTGTGAACAACAAAGGGGAAATGAAAGG

GTCTGCCATCACTGGACCGGTAGCCAAGGAGTGCGCCGACCTGTGGCCCAGGATCGCCTCTAACGCCAGTTCAATC---C

TCGTCAAGCTTTACCGCTTTCTGGCCAGGAGAACCAATGCCAAGTTCAATAAGATTGTGCTGAAGAGGCTTTTCATGAGC

AACACCAACAGACCTCCCCTGTCTCTGTCCAGACTGCGACACATGAAGAAGGCTGGTGTTGAGAAGACTGCTGTCGTTGT

TGGCACCATCACCAATGATGTCAGGATCTTTGAGATCCCCAAGATGAAGATTTGCGCCCTGCGTGTGACTGATCGCGCAC

GTGCCCGAATCTTGAAGGCTGGTGGAGAGCTGATCACCTTCGACCAGCTCGCTCTGAGGGCTCCCAAGGGCCAGAAGACT

GTGCTTATTCAGGGTCGTC

>Helobd

TTTGTTAAAGTAGTGAAGAATAAGGCGTACTTCAAGCGTTACCAGGTCAAATTCAGGCGAAGGAGAGAGGGTAAAACCGA

TTATTATGCCAGAAAGCGTTTAGTCGTTCAGGACAAAAACAAGTACAACACCCCCAAGTATCGTTTGATTGTTCGCTTCA

CTAACAAAGACATTGTGTGTCAGATAGCGTACGCCAGAATCGAAGGTGATGTTGTGATATGTGCTGCATATTCACATGAG

TTGCCTAAATATGGTGTCAAGGTCGGGCTGACCAACTATGCGGCTGCTTACTGTACTGGATTACTTATTGGACGCAGGAT

CTTGAAAAAGTTCAACTTGGATGGCATCTATCAAGGAACCGAGGAAGTCACTGGTGATGCCTATCAGGTCGAGTCCCAAG

ATGGACAGCCAGGTGCATTCAGGTGTTACTTGGATGTTGGATTGGCACGTACATCGACTGGAGCTCGTGTCTTTGGTGTC

TTGAAAGGAGCTGTTGATGCTGGCTTGGATATCCCTCATGCCGATGTCCATAGGAAGCACATATTTGGTCTGCACGTTGC

TGAGTACATGAGGAACCTCCAACAGGATGACGAGGAGATGTACAAGAAACAGTTCTCCAAGTTCATCACCAATGGACTCA

ATCCTGATAATATTGAGGAGATGTACAAGAAGGCCCATGCTGAAATC---AGGCAGGCTCGCCAACATGGGAACTTTTAC

GTCCCACCACAGCCAAAACTTGCCTTTGTTATCAGAATCAGAGGGATCAATGGACTGCACCCAAAGCCAAGGAAAATATT

ACAGTTGTTTCGTTTGAGGCAGATCAACAATGGAGTTTTCATCAAGTTGAACAAAGCTACCATCAACATGTTAAGGATCG

TTGAGCCATATGTCACTTGGGGGTACCCAAACTTGAAAAGTGTCAAGGAGTTGATCTACAAAAGAGGATTTGGCAAAGTT

GATGGCAGGAGGATCCCCCTAACTTCCAACATCATTGAAAAGAAATTAATGATTTGTATGGAGGATTTGATCCATGAGAT

CTTCACCGTCGGTCCCAACTTCAAGTTTGCCTCCAACTTCCTGTGGCACTTCAAACTGAACACACCAAACGGTGGATGGC

GTAAGAAGACAAACCATTTTGTTGATGGTGGTGATTTCGGGTGCAGAGAA---GTAAATCCGCTGTTTGAAAAGAGGGCC

AAGAACTTTTGTCAAGGCCAAGACATTCAACCCAAGAGAGATTTGTCCAGATTTGTGAGATGGCCCAAGTACATTCGTTT

GCAAAGGCAAAAACGTATTTTATACCAACGTCTCAAGGTGCCACCACCAATTCATCAGTTCAACCAGGCTCTTGATAGGC

AGAATGGTAACCAGTTATTCCGACTGATGGATAAATATAAACCAGAGACAAAAGTTGAGAAAAAAATAAGACTTGTTGAG

ATGGCAAAGAACAAGGTGAAAGGAAAGAGTGTTGAGCAGACAAAGAGACCACCAGTGATGAGATCAGGCATCAACACAGT

CACAGCTCTCGTTGAAAGAAAGAAAACTGATCTGGTTGTTATTGCCAGCGATGTTGATCCTATCGAGATAGTTCTATTCC

TGCCTGCTTTGTGCAGAAAAATGGGTGTTGCCTATTGCATCGTCAAAAACAAATCTCGACTAGGTAGAGTTGTCCACAGG

AAGACAGTTACATGTCTTGCATTTCCAACAGTCAACGAAGACAGGTCAGCATTGAACAAATTAGTTGAAACTGTGAAGAC

TGACTTCAATGAAAGAGCCGAAGAAGTGCGTCGCCACTGGGGTGGAGGTGTTATGGGCCACAAATCCCAAGCCCGT---G

TAATAGTCAAGGGACCCCGTGGCACCCTAAGACGAAATTTCACTCACATGGCTGTTAAGACAGTTTGTTCCCACATCGAG

AACATGATCAAAGGAGTTACTAAAGGATTCAGATACAAGATGAGGTCTGTCTATGCTCATTTCCCCATCAACGTTTCCAT

TTCTGAAAACAACTCCCTCGTCGAGATTAGAAACTTTTTAGGTGAAAAGATCAACAGGCAAGTTAAAATGTTGCCTGGAG

TTACCTGTAAAGATGAGTTTATACTCGATGGTAACGACATAGAATTGGTTTCCAGATCAGCTGCCCTGATCCAACAGTCG

ACAACGGTT---AAGCCTTACCCCAAGTCAAGGTTTTGCAGGGGTGTGCCAGATCCAAAGATTAGGATCTTTGATCTTGG

GAGGAAGAAGGCCAAAGTGGATGAGTTTCCACTGTGTGTGCACCTGGTGTCAGATGAATGGGAGCAAATTTCTTCGGAGG

CCCTCGAAGCCGGAAGAATTTGTGCTAATAAATATTTAGTGAAACACTGTGGTAAGGATGCCTTTCATCTGAGAGTTAGA

CTTCACCCCTTCCACGTCATCAGAATCAATAAAATGTTATCATGCGCCGGGGCAGATAGGCTCCAAACTGGGATGAGAGG

TGCTTTTGGCAAGCCTCAAGGGACAGTAGCACGTGTGGACATCGGACAGATTATCCTGTCTGTTCGTGCAAAGGATGCAC

ACAAAGATAAGGTCATTGAGGCTCTGAGGAGGGCAAAGTTCAAGTTTCCTGGCAGGCAAAAGATCCACGTGAGCAGGAAA

TGGGGTTTCACA---TTCACTGAGACAGTCGAGTTGCAGATTTCGTTGAAGAACTATGACCCTCAGAAAGATAAACGTTT

CTCCGGCACTGTAAAGTTGAGGAATATTCCAAGGCCAAAGATGGCGATCTGCGTTCTTGGAGATCAGCAGCATTGTGATG

AGGCAAAGGCTAACAGCGTTGAATGCATGGACGCTGAAGCGCTCAAGAAATTGAACAAAAACAAGAAACTCATTAAGAAA

TTAGGTAAAAAGTATGATGCTTTCCTGGCATCTGATTCCCTGATCAAACAGATCCCTCGTATTGTGGGCCCGGGATTGAA

CAAAGCTGGTAAGTTTCCTTCCATGCTGACCCATGGTGAGTCGATGACAGGCAAAATCGAAGAGGTCAAGGCCACAATAA

AGTTTCAAATGAAGAAGGTATTGTGTCTGTCAGTAGCAGTAGGAAATGTGAACATGACCCAAGATGAACTGGTCTCTAAC

ATCACCATGGCCATCAATTTCTTGGTCTCTCTCCTCAAGAAAAACTGGCAAAATGTCAGGTCGCTATACATAAAATCAAC

CATGGGACCCGTTCAGAGATTGTAC---GTATGTTTAAGGGCCGTCGGTGGTGAAGTTGGTGCCACATCAGCTCTTGCCC

CAAAAATTGGTCCCTTGGGTCTTAGTCCAAAAAAGGTTGGTGAAGATATTGCAAAGGCTTCCCAAGATTGGAAGGGCTTG

AGGGTGACTGTCCAGCTGACAATTCAGAATCGTCAGGCCCAAGTTACTGTCATACCTTCTGCTTCTTCTCTCATCATTAA

GGCCCTGAAGGAACCCCCAAGAGATAGAAAGAAAGTGAAAAATGTCAAGCACAGTGGAAACTTGACAATCGAGGAGATTA

TTGGCATTGCCAGGATCATGAAAGCAAGAAGTATGGCTAAAAATTTGGCTGGAACTGTCAAAGAAGTTCTAGGTACCTGC

CAGTCTGTTGGATGCACAGTCGAAGGCAGCCATCCT---AATAATATAATTCCCAATGGGCATTTCCACAAAGATTGGAA

GAAACACGTGAGGACATGGTTTAACCAGCCGGCTAGGAAGTTTCGCCGCTGGACGAAACGTAATGATAAAGCAAAAAAGA

TTGCTCCCAGGCCTGTCGCTGGTTTGAGACCCGTAGTTCGCTGCCCTACCTTCAAATATAACTCCAGAATTAGAGCTGGA

CGAGGATTCACTATTGAAGAGTTGAAAGCTGGTTTCAACAAAAAATATGCCAAAACCATTGGCATTGCAGTTGATTTCAG

ACGCCGCAACAAGTCAGTTGAAGGTTTTCAAGTGAATGTTCAGAGATTGAAGGAGTACAAGGCCAACTTGATCCTCTTCC

CCAAGAAATTGAACAAACCTCAGAAGGGAGATGCTACCAGGAGAAGGCAAGGGCAATCACCCCAGAGGAGAAAAAATCTG

CTT---GATGCCAGAGGTCATCTTCTGGGTCGACTGGCTGCCACCACGGCCAAATCAATTCTGCAAGGTAAACGTATAGT

TGTTCTAAGATGCGAGGGCATAAACATTTCTGGAAATTTTTACAGGAACAAGGTGAAGTATTTAGATTTTTTGAAAAAAA

GAATGAATACCAATCCAAGCCGAGGTCCTTACCATCATAGAGCTCCCAGCAAAATATTTTGGAGAACTGTTAGAGGTATG

TTGCCACACAAGTTACATCGTGGTAAGGAGGCTTTGGATCGTCTGAAAGCATTTGAGGGAGTACCTCCACCTTATGACAA

ACAAAAAAGATTTGTTGTTCCTTCTGCTCTTCGCATAATTAGATTGAAGCCACGAAGAAAGTTTTGCCAGTTAAGCAGGC

TGTCGCATGAAGTTGGTTGGAAGTATCAAGGTGTTGTAGCAACTCTTGAAGCGAAAAAGAATGTTATATCGAAA---ACC

TTTAAGAGGTTCGTTGAGATAGGTAGAGTGGCCTACATTGCGTTCGGTCCTGACGAAGGCAAATTAGTCACCATTGTTGA

TGTCATCGATCAGAACAGGGCCTTGGTTGATGGACCATGCACAAGTGTTGTTCGTCAAGCCATGAATTTTAACAAATTAG

AACTAACAAAGTTTGTTATTAAATTCCCACACTCGGCTAGAACTGGCGTTGTTAAGAAAGCTTGGGAGAAGGCTGACATC

AACAAGCTCTGGTCTGAAACATCATGGGCCAAGAAAATTGAAGCCCAGAATAAAAGAAAGGTAATGACTGACTTTGATCG

ATTCAAATTATTCAAAGCCAAACAA---ATGACTAGGTATTCGTTAAATCCGGAGAATGCGGCAAAAGCTGCCAAAGCCA

GGGGTTCTCATCTTAGAGTTCACTTTAAGAACACAAGAGAAACAGCCCAAAACATTAAACACATGCACCTTAGAAGAGCT

GTTGCCTATTTAAAAAATGTCATTGATCATAAAGAATGTGTTCCATTTAGGAGATTCACTGGTGGAGTTGGACGATGTGC

TCAGGCAAAAGCCTGGGGAACAACACAAGGTCGTTGGCCCAAGAAGAGTGCAGAATTTTTGTTGCAAATGTTGAAAAATG

CAGAAAGCAATGCTGATTTTAAGGGCTTAGATACTGACCATTTGGTGATTGAGCATATCCAAGTCAACAGGGCCCCAAAA

ATGAGAAGGAGGACATACAGAGCACACGGAAGAATTAATTACATGAGCAGTCCCTGCCACATTGAAATCATATTG---GC

CAAAAATTTATACATCATCTCTGTTCATGGCATTAAAGGTAGATTGAATAGATTGCCTGCAGCTGGCAGTGGTGATATGG

TTATGGCAACAGTAAAAAAAGGCAAACCAGAACTTAGGAAAAAGGTAATGCCGGCTGTTGTGGTACGGCAACGTAAAGCC

ATAAGACGAAAAGATGGGACCTTTTTAACATTTGAAGATAATGCTGGTGTTATAGTTAACAATAAAGGCGAAATGAAAGG

TTCCGCCATCACTGGACCTGTTGCCAAAGAATGTGCTGATCTCTGGCCACGTATTGCGTCAAATGCCAGTTGTATT---T

TAGTCAAGTTGTACAAATTTTTGGCAAGAAGGACAAATGCCAAATTCAATCAAATTGTCTTCAAGCGTCTGATCATGAGT

CGCACCAACAAGCCACCTCTCTCTCTGTCAAGATTGCGTCACATGAAAAAACAAGGCGAAAATAAGTTGGCTGTAGTTGT

TGGCACTGTTACAGATGACGAGAGATTGTACACTGTTCCGAAGTTGAAGGTAGTTGCACTTCGTGTCACTGACACAGCCC

GTACTAGAATCTTGAAAGCCGGCGGTGAGATCCTTACATTTGATCAATTAGCATTGAAGGCTCCAAAAGGAGAAAATACT

GTTCTTTTGCAAGGTCAAA

>Strongyl

TTTGTGAAAGTTGTGAAGAACAAGGCGTACTTCAAGCGGTACCAGGTCAAGTTCAGGCGTCGCCGTGAAGGCAGGACTGA

CTACCAGGCCCGCCGCTTCTTGGTTGTACAAGACAAGAACAAGTACAACACACCCAAGTACCGCATGATCGTTCGCTTCA

CCAACAAGAATATCACATGTCAGGTAGCGTACGCCAAGCTGGAGGGAGATGTCATTGTATGCGCCGCATACTCTCATGAG

CTCCCCCGCTATGGTGTCAAGGTCGGGCTGACCAATTATGCTGCAGCATACTGCACAGGACTACTCCTGGCTCGCAGGAT

TCTGAAGAAGTTCAACTTGGACGGTATCTACGAGGGTCAGACAGAGCCAGATGGCGATGACTACATGGTAGAGTCCGAGG

AGGGTAAGGCAGGAGCCTTCCGTTGCTTCCTGGATGTCGGTCTAGCCAGGACCACCACCGGCGCCAAGGTGTTTGGGGCC

CTCAAAGGCGCCGTAGACGGTGGTCTGGAGATCCCAAGCGCTGAGGTCCACAGGAAGTACATCTTTGGAGGTCACGTTTC

TGACTACATGAAGGAACTCCAGGGCGAGGATGACGATGCTTACAAACGCCAGTTCTCTCAGTTCATCAAGAATGACATCA

CACCTGACACACTTGAAGCCATGTACAAGAAGGCGCATGAAACCATC---CGCCAGGCACGCAAGCATGGCAACTTCTAC

GTCGAAGATGAACCTAGACTAGCGTTTGTCATCAGGATCAGGGGTATCAACGGCGTGAGCCCCCGTGTTCGTAAGGTCCT

TCAGCTGCTTCGTCTGCGACAGATCCACAATGGAGTCTTTGTCCGTCTCAACAAGGCTACCCTGCAGATGCTGAGGTTGG

TCGAGCCCTACATTGCCTGGGGGTACCCCAATCTGAAGTCAGTCCGTGAGCTGATCTACAAGCGAGGATTCGCCAAGATT

AAGAACAGCCGCATCCCTATGACAGACAACATCATTGAGGGTGCTCTTATCATCTGTGTGGAGGATCTGATCCATGAGAT

CTTCACCACTGGCGAAAACTTCAAGCAGGCCTCCAACTTCCTCTGGCCCTTCAAACTCAGCTCACCCCGCAACGGCTACC

GCAAGAAGGGCAACCACTTCGTCGAGGGTGGTGACTACGGCAACCGCGAA---GTCAACCCCCTTTTTGAGAAGAGGACA

AGGAACTTTGGAATTGGCAATGAGGTCCAGCCCAAGAGGGACCTGTCCCGCTTTGTCCTCTGGCCCAAGTATGTACGCCT

CCAGCGCCAGAAGGCTGTCCTTTACCAGCGTCTGAAGGTTCCTCCTTCCATCAACCAGTTCAGCCAGGCTCTCGATAGAC

AAACAGCTACACAGCTGTTTCGTCTGATGCACAAGTACCGCCCCGAGACCAAGCAGGAGAAGAAGGCCAGGCTCAGGGCC

CGTGCCGAGGATCGCGTCAAGGGACGTGAAGAGGTTCCCACTAAGCGTGAAGCTCGCATCATGTCCGGTGTCAACACTGT

CACCCGTTTGATCGAGTCCAAGAAAGCTCAGCTTGTTGCCATTGCTCATGATGTCGAACCCATCGAGATCGTGATGTTCC

TCCCAGCACTCTGCCGTAAGATGGGTGTTCCCTACTGCATCGTGAAGGGAAAGTCTCGGCTCGGTCAGGTGGTCCATCGC

CGTAACGCAGCTTGCCTTGCGTTCACCCAGGTCAACGAGGACAAGAGCGCCCTCTCCAAGCTGGTAGACACAGTCAGGAC

CAACTACAATGACAGATTTGAAGAGATCCGTCGTCACTGGGGTGGTGGACAGGTGGGTAGCAAGAGCCAGGCCCGT---G

TGACGGTCAAGGGACCGCGTGGGACTCTTGTGCGGACATTCAAGCATCTCTGTGTCCGAACTCTCTGCTCTCACATCGAG

AACATGATCAAGGGAGTCACTATGGGTTACCGCTACAAGATGAGGTCTGTGTACGCTCACTTCCCCATCAACGTCAACAT

CAAGAATGAGGGAACCCAGGTAGAGATCAGGAATTTCTTGGGAGAGAAGTTCATCAGGAAGGTGGATATGAAGGAAGGAG

TCACCTGTAAGGATGAGATCATCCTTGATGGCAACGATGTCGAGCTCGTCTCACAATGTGCTGCTCTCATCCAGCAATCC

ACCACCGTA---AAGCCCTATCCCAAATCCCGGTTCTGCCGTGGTGTCCCAGACCCCAAGATCCGTATCTTTGACTTGGG

TCGCAAGAGGGCTCTGGTTGACGAGTTCCCTATTTGCATCCATCTTGTGTCTGATGAATGGGAGCAGCTGTCGTCCGAAG

CCTTGGAGGCCGGGCGCATCTGTGCCAACAAGTACATGGTGAAGAACTGCGGTAAGGACGCCTTCCACATCAGGATCAGG

CTCCATCCATTCCACGTCATCCGCATCAACAAAATGTTGTCGTGCGCTGGAGCTGATAGGCTTCAGACTGGAATGCGTGG

AGCTTTTGGCAAGCCCCAGGGTACCGTAGCCCGTGTGAAGATCGGTCAGACCATCATGTCTGTCAGGACCAAGGAGGGTA

ACAAGGCTGCTGCACATGAAGCTCTCAGGAGGGCAAAGTTCAAGTTCCCTGGACGTCAGAAGATCTTTGACTCCAACAAG

TGGGGCTTCACC---TTTCTGGAGACGATCGAGCTTCAGGTCAACCTCAAGAACTACGACCCCCAGAAGGATAAGCGTTT

CTCCGGAACCGTCAAATTGAGGCACATCCCTCGCCCCAAGTTCTCCATGTGCATCCTTGGAGATCAGCAGCATCTTGATG

AGGCCAAGGCCAATGGCATCCCCTGCATGGACATGGAGGCCCTCAAGAAGCTGAACAAGAACAAGAAGCTTGTCAAGAAG

CTAGCTAAGAGGTATGACGCCTTCCTTGCCTCCGACTCGCTCATCAAGCAGATCCCACGTATCCTGGGACCCGGTCTTAA

CAAGGCAGGCAAGTTCCCCACCCTCCTGACCCATACCGACTCCATGGTTTCAAAGGTTGAAGAGGTCAAGGCTACCATCA

AGTTCCAGATGAAGAAGGTTCTGTGCTTGGCCGTTGCCGTCGGACACGTGGAAATGGACGAGGAGGATCTGGTGTCCAAC

ATCAACCTGGCCATCAACTTCCTGGTGTCCCTGCTCAAGAAGAACTGGCAGAACGTGCGTTCCCTGTACATCAAGAGCAC

CATGGGAAAACCACAGCGCCTCTAC---GTCTGCCTGAGGGCTGTAGGAGGTGAGGTCGGAGCAACCTCAACCCTCGCTC

CCAAGATCGGTCCCCTTGGTCTGTCACCTAAGAAGGTAGGAGATGACATCGCCAAGGCCACCCAGGAATGGAAGGGGCTG

AAGATCACCGTCATGTTGACCATTCAGAACCGTCAGGCCAAGGTCAGCGTGGTGCCCAGCGCATCATCTCTCATCATCCG

TGCCCTCAAGGAGCCCCCTCGTGACAGGAAGAAGGTCAAGAACATCTCTCACAGCGGGAACATCGGCCTGGACGTCATCA

TTGAGATCGCCAAGACGATGCGTGAGCGTTCCATGGCCCGTGAGCTGAAGGGAACGGTCAAGGAGGTCCTGGGAACCGCC

CAGTCTGTCGGATGCACCGTGGACGGGGTCAAACCC---AACGGAGTGCTACCAAACGTCCATTTCCGCAAGGAATGGCA

AAACTATGTCAGGACGTGGTTTGACCAACCAGCCCGCAAGAAGAGACGACACAACAACCGTGTCCAGAAGGCCCGCAAGA

TCGCCCCTAGACCCATCGCAGGACTTCGACCCCAGGTTACTTGTCAGACATTCAAGTACCACACCAAGCTGAGGGAGGGA

CGTGGCTTCACGCTTGAGGAGCTCAAGGCTGGTATCCACAAAAAGTATGCCCCAACCATCGGCATCTCTGTTGACCACAG

GAGGAAGAACAGGTCAGTCGAAGGGCTCCAGGCCAACGTCCAGCGCCTCAAGGAGTACAGATCCAAACTCATCCTCTTCC

CCAAGAAGCTGAGCAAACCCAAGAAGGGAGACAGTGATGAGGCCGAGCTTAAGATGGCCACTCAACTGGAGGGACCCGTC

ATG---GATGGGAGAGGTCACTTACTTGGACGCCTGGCGTCCATAGTGGCCAAAAACCTCCTCCAAGGTCAGAAGGTTGT

CGTGGTACGATGTGAGCTCATCAACATCTCAGGCAGCTTCTACAGGAACAAGCTGAAGTACATGCAGTTCATGCGCAAGC

GCACAAACACCAAGCCCTCCCGTGGGCCCTACCATCTCCGCAGTCCTAGCCGTATGTTCTGGAGAGTCATCAGGGGAATG

TTGCCACACAAGAGGACACGTGGCAAGGATGCTCTTGAGAGGCTCAAGGTCTTTGAGGGCTGCCCTGCCCCCTATGACAG

GAAGAAGAGGTTCGTTGTGCCCTCTGCACTGAGAGTGATGCGCCTCAAGCCCAACAGGAAGTTCTGCGTGCTCGGTCGTC

TGGCTCATGAGGTCGGCTGGAAGTACAAGAACATCATCGAGGCTCTCGAGGAGAAGAGAAAGGCACGTGCACAT---GTG

TTCAGGAGGTTCGTCGAGGTCGGCCGTGTGGCCTACATCGCCAGCGGACAAAACAAGGGCAAGCTATGTGTCATCGTTGA

TGTCATTGACCAGAGGAGGGCCCTTATTGATGGACCACTCTCCGGCGTGAAGCGCCAGGGAATGCGCTTCAAGCAGCTTC

ACTTGACAGACTTTGTTATCCGCATTCCTCATTCTGCGCGTAACAGCACCGTAAGGAAGGCGTGGGAAAAGGACGAGATC

ACCAGCAAGTGGGACGCCACCATCTGGGCCAAGAAGCTCGCTGCCAAGCAGAAGAGAAAGCAGATGACCGACTTTGACCG

CTACAAGCTCATGCGTGCCAAGCAA---ATGACGCGCTACTCCCAAGAACCGGAGAATTCTGCAAAGTCCTGCAAGGCAA

GGGGCTCCTACCTTCGTGTTCACTTCAAGAACACCCGCGAGACCGCTCAGGCCATCAAGCACATGCATGTCCGCAAGGCC

ATCCGCTTCCTGAAGGATGTAACCAACAAGAAGCAGTGCGTTCCCTTCCGCAGGTTCAATGCCTGCATTGGACGCAAGGC

CCAGGCTAAGGCATGGAACCACACCCAAGGTCGCTGGCCCAAGAAGAGTGCCGAGTTCCTTCTTCAGCTCCTGAAGAATG

CTGAGAGCAACGCAGAGTACAAGGGTCTCGATGTCGACTCCCTTGTCATCGATCACATCCAAGTCAACGCTGCCCCCAAG

ATGAGGAGGAGGACATACCGTGCCCATGGTCGTATCAATTACATGAGTTCTCCATGCCACATTGAGCTGATCCTT---GG

TAAGAACCTGTACATCATTGCTGTAAGCGGAATCGGGGGTAGGCTGAACAGACTGCCTAATGCTGGTCTCGGTGACATGA

TCGTTGCTACTGTCAAGAAAGGCAAGCCAGAACTCAGGAAAAAGGTCATGCCTGCAGTAGTCATTCGGCAAAGGAAGCCC

ATCAGAAGAAGAGAAGGCATAGTACTCTACTTTGAAGACAATGCGGGAGTCATAGTGAACAACAAGGGAGAAATGAAAGG

TTCAGCCATCACAGGTCCAGTTGCCAAAGAGTGTGCCGACTTATGGCCCCGTATTGCCAGCAACGCGAGTACCATT---T

TGGTGAAGCTCTACAGGTTTCTGGCCAGGCGTACAAATGCCAAGTTCAACAACATCGTCCTCAAGAGGCTGTTTATGAGC

CGCAGCAACAAGGCTGCCATGTCTCTCGCACGTGTGCGCTTCATGAACAAAGATGGCGAGGGCAAGATTGCCGTCGTAGT

CGGGTGCATCACCGATGACGTGCGTATCCACAAGCTGCCCAAGCTGAAGATCTGTGCCCTGAGGGTGACTGCCCGTGCTA

GGGCTAGGATCATCAAGGCTGGAGGAGAGATCATCACCTTCGATCAGCTCGCCCTCCAATCCCCCAAGGGACAGAACACA

GTGCTCATGCAAGGATGCC
