# Supplementary material for: Proteome Adaptation to High Temperatures in the Ectothermic Hydrothermal Vent Pompeii Worm
Source: PLoS One. 2012 Feb 10;7(2):e31150. doi: 10.1371/journal.pone.0031150 (PMC3277501; doi:10.1371/journal.pone.0031150)
Supplement: Dataset S2 — Pairwise alignments of sequences obtained from the 335 orthologous genes used to compare the amino acid composition and codon usage between the two alvinellid worms. (DOC) [file pone.0031150.s005.doc]

**Additional data file 4.**

Pairwise codon alignments of the 335 orthologous coding sequences obtained between Alvinella pompejana and Paralvinella grasslei.

>TERA00007

ATGAGAGGAGGAATGCAGATCTTTGTGAAGACCCTGACAGGCAAGACCATCACACTGGAAGTCGAACCATCAGATACCAT

TGAGAATGTGAAGACCAAGATCCAGGATAAAGAAGGCATTCCCCCAGATCAGCAGCGTCTGATTTTTGCTGGAAAGCAGC

TGGAAGATGCCCGAACCCTGAGTGACTACAACATTCAGAAGGAATCCACACTGCACTTGGTGCTTCGTCTGAGAGGAGGA

ATGCAGATTTTCGTGAAGACCCTGACAGGCAAGACCATCACACTGGAAGTCGAACCATCAGATACCATTGAGAATGTGAA

GACCAAGATCCAGGATAAAGAAGGCATTCCCCCAGATCAGCAGCGTCTGATTTTTGCTGGAAAGCAGCTGGAAGATGCCC

GAACCCTGAGTGACTACAACATTCAGAAGGAATCCACACTGCACTTGGTGCTTCGTCTGAGAGGAGGAATGCAGATTTTC

GTGAAGACCCTGACAGGCAAGACCATCACACTGGAAGTTGAACCGTCAGATACCATTGAGAATGTGAAGACCAAGATCCA

GGACAAAGAAGGCATTCCCCCAGATCAGCAGCGTCTGATTTTTGCTGGAAAGCAGCTGGAAGATGCCCGAACCCTGAGTG

ACTACAACATTCAGAAGGAATCCACACTGCACTTGGTACTTCGTCTGAGAGGAGGAATGCAGATTTTCGTGAAGACCCTG

ACAGGCAAGACCATCACACTGGAAGTCGAACCGTCAGATACCATTGAACATGTGAAGACCAAGATCCAGGACAAAGAAGG

CATTCCCCCAGATCAGCAGTAA----------------------------------------------------------

--------------------------------------------------------------------------------

--------------------------------------------------------------------------------

--------------------------------------------------------------------------------

--------------------------------------------------------------------------------

--------------------------------------------------------------------------------

----------------------------------------------------------------------------

>TERA00014

ATGACCCAGACAAGAACGTTACTGACACAGTCACAGGATAAACAAAACAGCTTAGTACCATACAGAGAAAAAAGTACAGC

CATCACTCCAAAAAAGAAGGGTCGGTATGAATACTCCCCTTTCCAGTCTCCAAGTAACAAAGTTGAGTATGTTCTGGCCA

GAGTTGATGACCTAGTCAACTGGGGAAGAAAGAGTTCTCTTTGGCCTATGACCTTTGGTTTGGCCTGCTGTGCTGTCGAG

ATGATGCACTTTGCTGCCCCCCGTTATGACATGGACAGGTTTGGAGTTGTGTTTCGTGCAAGTCCTCGGCAGGCTGACTG

TATCATAGTTGCTGGAACTGTAACCAACAAAATGGCTCCAGCACTGAGAAAGGTCTATGATCAGATGCCAGACCCACGCT

GGGTTATCTCAATGGGTAGCTGTGCCAATGGAGGTGGTTATTATCATTACTCATACTCAGTGGTACGAGGCTGTGACAGA

ATCATACCAGTTGATATATATGTCCCAGGTTGTCCTCCAAGTGCTGAAGCATTGCTGTATGGAGTGCTACAGCTGCAGAA

AAAGATCAAACGTTGGAATAGCATGCAGATGTGGTACAGGAGATAA----------------------------------

--------------------------------------------------------------------------------

--------------------------------------------------------------------------------

--------------------------------------------------------------------------------

--------------------------------------------------------------------------------

--------------------------------------------------------------------------------

--------------------------------------------------------------------------------

--------------------------------------------------------------------------------

--------------------------------------------------------------------------------

----------------------------------------------------------------------------

>TERA00020

ATGGATGACATGATTTGCGCTTCAGACACGGACACACATGAAGAACGAGACTCCTGTCAGGGTGATTCCGGCGGTCCCAT

GGCAACGAAGGTCAACGATCAGTATTACGTCATCGGTCTGGTGTCCTGGGGTTATGGCTGCGCCAGTGGCTGGCCGGGCG

TCTACAATAGGATTGGATACTAA---------------------------------------------------------

--------------------------------------------------------------------------------

--------------------------------------------------------------------------------

--------------------------------------------------------------------------------

--------------------------------------------------------------------------------

--------------------------------------------------------------------------------

--------------------------------------------------------------------------------

--------------------------------------------------------------------------------

--------------------------------------------------------------------------------

--------------------------------------------------------------------------------

--------------------------------------------------------------------------------

--------------------------------------------------------------------------------

--------------------------------------------------------------------------------

--------------------------------------------------------------------------------

----------------------------------------------------------------------------

>TERA00034

ATGGAGAAGGGCAAGAAGATTTTTGTGACGAGGTGTGCCCAGTGTCACACAGTGGAGGCTGGAGGTAAACATAAGACTGG

CCCCAATCTGCATGGCTTCATAGGAAGGAAGACTGGACAGGCTCCTGGATATACGTTTACAGATGCCAACTTGAACAAAG

GCATAGTCTGGAATCGGGAGACCCTTGATATTTATTTGGAGAACCCCAAGAAGTACATACCCGGAACAAAGATGATTTTT

GCTGGTTTGAAGAAGAAATAA-----------------------------------------------------------

--------------------------------------------------------------------------------

--------------------------------------------------------------------------------

--------------------------------------------------------------------------------

--------------------------------------------------------------------------------

--------------------------------------------------------------------------------

--------------------------------------------------------------------------------

--------------------------------------------------------------------------------

--------------------------------------------------------------------------------

--------------------------------------------------------------------------------

--------------------------------------------------------------------------------

--------------------------------------------------------------------------------

--------------------------------------------------------------------------------

----------------------------------------------------------------------------

>TERA00084

ATGTCATCTACAGTTGCCGCACAGGCAACAGGCATGTCAATGGGTTATATTGCCAGGAGCAAAGGTGTGGAAGATGTCCG

TGTTTACATAAAAGGGATTGGCTCAGGACGCATGCCAGCTCTCAAAGGCCTCCACATGTCTGGCGTCAAAATTGTATCAA

TTACTGATAATACAACTATTTCAACCAATGGCCCACGTCCACGTAAAATGAGAAGACTATAA------------------

--------------------------------------------------------------------------------

--------------------------------------------------------------------------------

--------------------------------------------------------------------------------

--------------------------------------------------------------------------------

--------------------------------------------------------------------------------

--------------------------------------------------------------------------------

--------------------------------------------------------------------------------

--------------------------------------------------------------------------------

--------------------------------------------------------------------------------

--------------------------------------------------------------------------------

--------------------------------------------------------------------------------

--------------------------------------------------------------------------------

--------------------------------------------------------------------------------

----------------------------------------------------------------------------

>TERA00111

ATGCAAGAATACCTGATCGCGATGGGTCAAGTACCGGATGTCTACCACAAAGAGGCCGCCATGCGGCAGGCTTTCGAGCG

GGCGGACAAAAACAAAGACGGAAGCTTGGACATCGGCGAGATCAACGCCATCTTCCGGGAGATGAACACCTTCCTCAATC

CAGACGAGCTCTTTCAGATCGTCCACGAAATCGACAAGGACCACAGCGGACGGATCAACTACAACGAATTCCTGACCTTC

TTCATGAAGCAGCAAAATGTCAACTTCGAGAGCAGTGACAGCGATTGGGATTAA--------------------------

--------------------------------------------------------------------------------

--------------------------------------------------------------------------------

--------------------------------------------------------------------------------

--------------------------------------------------------------------------------

--------------------------------------------------------------------------------

--------------------------------------------------------------------------------

--------------------------------------------------------------------------------

--------------------------------------------------------------------------------

--------------------------------------------------------------------------------

--------------------------------------------------------------------------------

--------------------------------------------------------------------------------

--------------------------------------------------------------------------------

----------------------------------------------------------------------------

>TERA00167

ATGACTGAGGTTAAGTTGACAGACGCCCAGATCAAGGCGGCCGAGGCAGCATTCAAGGCGTTCGACAAGAGGGACGAGCA

GAAGATCAAGGTCGGAGACATCGAGAACGCCTTGAAGAAACTAGGCCACAGCATAAAGGCAGAATGGCTGGAGAAGATGG

AAGACTCCATCGACACAGAAGGTACTGGTTACATAGACTTTAACGAGTTTCTACAAATACTCACGAAGAAGATGCAGGCT

GATGAAGATGAGCGAGAGTTGAGGGAGATCTTCCGCGTTCTGGATAAAGAGAAGAAGGGAGAAGTAAACGTGAACGAATT

GAGATGGATCCTGAAGAACCTGGGAGATGATCTTACAGAGGAGGATATCGATGACATGATAGCTGATGTGGATACCGATG

GATCCGGATGGGTTGATTATGACGAATTTGCTAAGTTGATGGGCGAGTAA------------------------------

--------------------------------------------------------------------------------

--------------------------------------------------------------------------------

--------------------------------------------------------------------------------

--------------------------------------------------------------------------------

--------------------------------------------------------------------------------

--------------------------------------------------------------------------------

--------------------------------------------------------------------------------

--------------------------------------------------------------------------------

--------------------------------------------------------------------------------

--------------------------------------------------------------------------------

----------------------------------------------------------------------------

>TERA00190

ATGGAAGATACATATGCATTGTCTTCCATGAAAACCCTGGAAGAGGCTGTTAAAAATGTTATTCAGTTTATGGGAATGCA

ACCATGTGAGAGATCAGACAAGGTTCCTGAAGGCAAGAGTTCACATACATTGTACTTAGGTGGTGTGTACAGAGGAGGGC

ATGATGTTTTAGTACGTGCTAAGCTAGCTCTGACTGAGAGTGGTGTCACCATGCAAATCACTGTTAGAAGTACTGATGCC

TCAGCTGCTGAGGTAATAGCAAGTGCTGTTGGCTAA--------------------------------------------

--------------------------------------------------------------------------------

--------------------------------------------------------------------------------

--------------------------------------------------------------------------------

--------------------------------------------------------------------------------

--------------------------------------------------------------------------------

--------------------------------------------------------------------------------

--------------------------------------------------------------------------------

--------------------------------------------------------------------------------

--------------------------------------------------------------------------------

--------------------------------------------------------------------------------

--------------------------------------------------------------------------------

--------------------------------------------------------------------------------

----------------------------------------------------------------------------

>TERA00193

ATGTACGCCTACGTCTGCGACACGGGCATCGACATCGACCACAGCGACTTCGGCAATCGGGCCATTTGGGGTATGACGGC

TGGAGACCTGCGTCCGGGCACCGACGACAACGGTCACGGAACGCACTGCGCTGGTACCATCGGCTCCAACTCGTACGGCG

TAGCCAAGGAGTCGACGCTGGTGGCCGTCAAGGTGCTGAACGCACTGGGCAGCGGAACCACCGACGACATCGTGGAGGGA

TTGGAATGGGTGCTGACCGACCACAACTCCAGACCAGACGCCAGGAGCGTGGTCAACCTGTCACTGGGCGGCACGGGAAC

CAACCTGCCCATGGAGGACGCCGTCCAGAATCTGATCGACGGAGGCGTCACCGTCTGCATTGCCGCCGGCAACAGCGACG

ACGACGCCTGCAACTACACGCCGGCCCGCGTGCCTGACGCCCTCACCGTCGGCGCCTCCGACATCTCCGACTGGTCGGCC

ACCTTCACCAACTACTAA--------------------------------------------------------------

--------------------------------------------------------------------------------

--------------------------------------------------------------------------------

--------------------------------------------------------------------------------

--------------------------------------------------------------------------------

--------------------------------------------------------------------------------

--------------------------------------------------------------------------------

--------------------------------------------------------------------------------

--------------------------------------------------------------------------------

--------------------------------------------------------------------------------

----------------------------------------------------------------------------

>TERA00198

ATGTGCCCAGTGGGCGAGAAAATCCAGCTTAATCTACCCTTTATGGTAGCTAAACAACGAAGAAACATGACGGAGCAAAT

GACGCTTCGCGGTACCCTCCAAGGGCACGGAGGATGGGTAACCCAAATTGCTACAACGCCACAATTTCCTGATATCATTT

TGTCGGCTTCTAGAGACAAATCGCTCATTCTGTGGCAGCTGACTCGCGAGGAATCGCGTTACGGCTTCCCTCGCAAGGCC

TTGCGCGGACATGGACACTTCGTGTCTGACGTCGTCATGTCATCAGATGGACAGTTCGCCCTGTCTGGATCCTGGGATGG

AACCCTTCGTTTGTGGGATCTTGGCACTGGTCAGACAACTCGTAGGTTTGTTGGACACACGAAGGATGTGCTAAGTGTGG

CTTTCTCAGCTGATAACCGTCAGATTGTGTCAGGTTCACGTGACAAGACCATCAAGTTGTGGAACACTCTTGGGGTGTGC

AAGTATACCATTCAGGAAGATGGGCACACAGAGTGGGTATCATGTGTTCGATTCTCACCAAACACCCAGAATCCCATCAT

TGTGTCCTGTGGCTGGGACAAACTGGTTAAGGTGTGGAATCTGACAAACTGCAAGCTAAAAACAAACCACTTCGGACACT

CAGGTTATCTGAACTGTGTCACTGTGTCCCCTGATGGATCTTTGTGCGCTTCTGGTGGAAAAGATGGCCAGGCAATGTTA

TGGGATTTGAATGAAGGCAAGCATCTGTACACATTGGATGGTGGTGATGTCATCAACTCACTGTGCTTCAGCCCCAACAG

ATACTGGCTTTGTGCTGCTTCTGGACCAAGCATAAAGATCTGGGATCTGGAAGGCAAGGTTGTTGTGGATGAGCTGCGTC

CAGAAGTGATCAGCACCAGTGCCAGTGCCGAGCCACCTCAGTGTATATCCCTGGCTTGGTCAGCTGATGGCCAGACACTG

TTTGCTGGATACACAGACAACCTGATTCGTGTGTGGCAGGTATCTATGTAA-----------------------------

--------------------------------------------------------------------------------

--------------------------------------------------------------------------------

--------------------------------------------------------------------------------

----------------------------------------------------------------------------

>TERA00288

ATGTACACAAACATTTGGGGAATCAGTAACGCCCAGTGTAGTACAGAATATGACGGCATCACCCAGGCCATGCTGTGTGC

TGATTCGTCTGGTAACGACAGGGATGCTTGCCAGGGTGACTCTGGTGGTCCTCTGGTATACAACAGGAATGGACGTTTTG

AAATAATTGGGCTGGTCTCGTGGGGTATCGGATGTGCCACAAATCCGGGAGTTTACACCCGAGTTACTCACTTCCTGGAC

TGGATAGCTGAGAACGCTGTCTAA--------------------------------------------------------

--------------------------------------------------------------------------------

--------------------------------------------------------------------------------

--------------------------------------------------------------------------------

--------------------------------------------------------------------------------

--------------------------------------------------------------------------------

--------------------------------------------------------------------------------

--------------------------------------------------------------------------------

--------------------------------------------------------------------------------

--------------------------------------------------------------------------------

--------------------------------------------------------------------------------

--------------------------------------------------------------------------------

--------------------------------------------------------------------------------

----------------------------------------------------------------------------

>TERA00318

ATGAATAAATACGACTGGCATTTCCTGCCCGTCATTAATCCAGACGGTTATGTGTATTCATGGACTACAGATAGATACTG

GAGGAAGTCCAGGAATATTAACGTGGGTTCTGAGTGTAACGGAACCGACTTGAATAGGAACTTTGACTCGCACTGGTCAC

TCGACGGGACTGATCCCGATCCGTGCTCCAACAATTACCAAGGAAGTGGCGCCGCCTCCGAGCCAGAGATCAAGGTCATG

CAGAACGAACTGCTCCGACTGGGGCCGACCTTGACCGGTTCAGTTGCCATGCACACTCCAGCTTCGATGTGGCTACATTC

TTGGGGATTCACTGAGGATGGCTCGTGTGCCAGACCCGTGGACTACTAA-------------------------------

--------------------------------------------------------------------------------

--------------------------------------------------------------------------------

--------------------------------------------------------------------------------

--------------------------------------------------------------------------------

--------------------------------------------------------------------------------

--------------------------------------------------------------------------------

--------------------------------------------------------------------------------

--------------------------------------------------------------------------------

--------------------------------------------------------------------------------

--------------------------------------------------------------------------------

--------------------------------------------------------------------------------

----------------------------------------------------------------------------

>TERA00344

ATGAAGACGCTCATCATCCTCTTCGCTTTTGTTGCCGTTGCAACGTGCACCGAATGCGGACCTCTACAGAGGTTGAAGGT

CAAACAGCAGTGGTCGGTAGCCTTTGGCACAGATCATCATAGGATTGACTTTGGCATCGCCATTTGGAGAGGCCTTTTCC

GCCAAGTACCTGATGCCAGGGATCTGTTCAAGCGCGTCAACGGTGATGATCTGTACTCGGGAGCTTTCCGCGCCCATTCC

ATGCGTGTACTCGGCGGCCTGAACATGATCATCTCCGCCATCGACAACGAGGACATCGCCAAGTTCATCCTTAACCATCT

GCACGACCAGCACGTCGACAGACATGTGGCTGCAAGCTACTACCAGGCAATGAAGAACTCTCTGATGAAGGTCATCCCAG

CTGCCATTGGCCGATGCTTCGACGAAGATGCATGGAATGCATGCATGGATGTCATCATCCATGGAATCAGCGGCAACTAA

--------------------------------------------------------------------------------

--------------------------------------------------------------------------------

--------------------------------------------------------------------------------

--------------------------------------------------------------------------------

--------------------------------------------------------------------------------

--------------------------------------------------------------------------------

--------------------------------------------------------------------------------

--------------------------------------------------------------------------------

--------------------------------------------------------------------------------

--------------------------------------------------------------------------------

----------------------------------------------------------------------------

>TERA00363

ATGGTCAACACGTGGATGGCATTCAATGTTGGCAAAGCCCGCAGGCAGTACGGCGTAGAATATCCAAAGATGTACAGTGA

GGATAATACAATGTTCAACTGTATCCAGCGAGCCCACCAGAACACTTTGGAGAACCACCCAACATTCCTATTCTTTTTGT

TGACTGGTGGACTCCAGTATCCAAAAATTAGTGCAGCTGCTGGTTTAGTCTATGTGCTTGGACGCATTGTTTATGCTAAA

GGGTACTACACAGGAGAACCGAAGAACAAGAAGTGGGGAGGAATTGGACAAATTGGGCTGTTGGTCTAA-----------

--------------------------------------------------------------------------------

--------------------------------------------------------------------------------

--------------------------------------------------------------------------------

--------------------------------------------------------------------------------

--------------------------------------------------------------------------------

--------------------------------------------------------------------------------

--------------------------------------------------------------------------------

--------------------------------------------------------------------------------

--------------------------------------------------------------------------------

--------------------------------------------------------------------------------

--------------------------------------------------------------------------------

--------------------------------------------------------------------------------

----------------------------------------------------------------------------

>TERA00380

ATGAAAATGGCTGCTCTGGAGCAGTATGTTAATACAGTTAGAACGCTGTCACAGCAAGGTAATTTTGCTCAGCTGTGCGA

GTTCATCAGTAAAAGTGGAGAAGTGCTTTCAAAGAATGCTGCTCATCTTGATAATGTTCTTGGAACTTTTGACATCCAAC

AGCATTCACTTGGCGTCTTAGGCATATTGTGTATAAAGTATTCACTGCCAAACATACCTGATTTTGAGACCCTGTTTGTT

CAGACCCAGGAGTTCATTAATACCTGCAATGGAGAACAAGTGCGCTTTGCAACAGACAACTATGCTGAACTCTGCCATAA

ATTTACTCAGAACTTAGTTGAAAGAAATCAGCCAATACGTGGCATTGTTATATTGTGTGCTGCCATCAGCAAGATCCAAT

TGTTTCCATCGCAGCTAACATCCATTCATGCAGACCTGTGTCAGCTGTGCCTGTTGTCCAAATGCATGAAGCCAGCCTTA

CCACTTTTAGATGTGGACATCACGGATATATCAAAAGAGGGTGGACAGTATGATGCTAAACATTTTCTTCTCTACTATTA

CTATGGAGGAATGATCTACACAGCTCTGAAGCATTATGACAGGGCACTGTACTTCTTTGAGATAGCTGTGACCACTCCAA

GTATGGCCGTCAGTCACATCTAA---------------------------------------------------------

--------------------------------------------------------------------------------

--------------------------------------------------------------------------------

--------------------------------------------------------------------------------

--------------------------------------------------------------------------------

--------------------------------------------------------------------------------

--------------------------------------------------------------------------------

--------------------------------------------------------------------------------

----------------------------------------------------------------------------

>TERA00410

ATGATAGGTAACATGACCAACGGCAACTACAAGGACCTGATGGCCGAGCTGGACGGCTTGGCGTTCACCACCAAGGACAA

TGACAACGACGAGTGGTGGGACAATAACTGCGCTTATTACTACCGTGGAGCCTTCTGGCTGAAGAACTGCGGCTTTGACC

TGAACAGAGAGTACTGTGACGGCCGAGGCTGTATGACCTGGGGAGACAGGGTGGCCAAGAAGTCTCTGATGAAGATCCGG

TAA-----------------------------------------------------------------------------

--------------------------------------------------------------------------------

--------------------------------------------------------------------------------

--------------------------------------------------------------------------------

--------------------------------------------------------------------------------

--------------------------------------------------------------------------------

--------------------------------------------------------------------------------

--------------------------------------------------------------------------------

--------------------------------------------------------------------------------

--------------------------------------------------------------------------------

--------------------------------------------------------------------------------

--------------------------------------------------------------------------------

--------------------------------------------------------------------------------

----------------------------------------------------------------------------

>TERA00506

ATGACAATGTTTCTCACAAGATCTGAATATGACAGAGGTGTAAACACTTTTTCTCCTGAGGGACGTCTCTTCCAAGTTGA

ATATGCTATAGAGGCAATAAAGCTTGGATCAACAGCTATTGGCATTCAGACATCGGAGGGTGTTGTTTTAGCTGTTGAAA

AGCGAGTCACTTCACCACTGATTGAACCTAACAGCATTGAAAAAATCCTGGAGGTTGACAGTCACATAGCCTGTGCTATG

AGTGGACTGATAGCTGATTCTAGAACACTGATAGACCGAGCACGTGTGGAAGCACAGAATCACTGGTTTACTTACAATGA

GAAGATGAGTATTGAGAGTGTAACTCAGGCTGTCAGTAATTTGGCTCTACAGTTTGGTGATGATGATGCAGGACCTGGTG

CCATGAGTCGACCTTTTGGGGTGGCACTTTTATTTGCTGGTATAGATGAGAAAGGACCACGCTTATTTCATATGGATCCC

TCAGGTACCTTTTTACAATATGATGCAAAAGCCATTGGCTCAGGCTCAGAAGGTGCTCAGCAAGCCTAA-----------

--------------------------------------------------------------------------------

--------------------------------------------------------------------------------

--------------------------------------------------------------------------------

--------------------------------------------------------------------------------

--------------------------------------------------------------------------------

--------------------------------------------------------------------------------

--------------------------------------------------------------------------------

--------------------------------------------------------------------------------

--------------------------------------------------------------------------------

----------------------------------------------------------------------------

>TERA00508

ATGAAGGTCAAAGGAAAGTGTACAACTGATCACATTAGTCCAGCTGGACCATGGCTTAAATACAGAGGTCACCTTGATAA

TATCTCTAACAACCTATTAATTGGAGCTATCAACAGTGAAAACAACAAAGCCAACAAAATTAAGAATCAGCTTACTGGAG

AGTATGACTCGGTGCCTGCTACAGCCAGACATTACAAGAAAAACAATCTAGCTTGGGTTGTTGTTGGAGAGGACAACTAT

GGTGAAGGTAGCAGTCGTGAGCATGCTGCTTTGGAACCTAGACATCTTGGAGGAAGGGCCATCATCGTTAAGAGTTTCGC

TAGAATTCATGAAACCAATTTGAAGAAACAGGGTTTGTTGCCTTTGACATTTGCTAACCCATCGGACTATGACAAGATCC

AGCCTACAGATCGTATATCTATCATAGGTCTCAAAGACTTCACTCCAGGAAAGCCCTTAACTGCTCGCATCACACATCAA

GATGGTAGCACTGAAGAAATCAAACTGAATCATTCATTCAATGAACAACAGATCACATGGTTCAGGGCAGGTAGTGCACT

GAACAGAATGTAA-------------------------------------------------------------------

--------------------------------------------------------------------------------

--------------------------------------------------------------------------------

--------------------------------------------------------------------------------

--------------------------------------------------------------------------------

--------------------------------------------------------------------------------

--------------------------------------------------------------------------------

--------------------------------------------------------------------------------

--------------------------------------------------------------------------------

----------------------------------------------------------------------------

>TERA00548

ATGAGGGCCAAGATGGCAAAACGAAAGGGACGTGCCCCTAAGGGAGGCTTTGTGAAGGTCGTCAAAAACAAACAGTACTT

CAAACGATACCAAGTGAAGTTCAAGAGGAGGAGAGAGGGCAAGACTGACTACTATGCCCGCAAGCGCCTCATCTTCCAGG

CCAAGAACAAGTACAACACACCCAAGTACAGGATGATTGTACGCTTCACCAACAAGGATATCGTATGCCAGATAGCCTAT

GCCAAGATTGAGGGTGATGTTGTTATCTGTTCTGCCTATGCCCATGAGCTGCCACGTTATGGGGTCACGGTTGGATTAAC

CAATTATGCTGCAGCATACTGTGTTGGCCTACTTTTGGCTCGCAGGATGCTGAAGAAGTTTGGTTTGGATAAAATATATG

CTGGTAGTGTGGACGTGACTGGAGATGAGTACATTGTGGAGCCGATTGATGGACAGCCAGCACCATTCCGATGCTACCTA

GATGTCGGCTTGGCAAGGACCAGCACTGGTGCCAAGGTGTTTGGTGCTCTGAAGGGTGCAGTGGATGGTGGTATGAACAT

CCCCCATTGCTGCAAACGATTCCCTGGATATGACAATGAGAATGGTGAGTTCAAGGCTGATGTCCATCGTATGCACATCA

TGGGACAGCACGTGGCTGACTACATGCGGCTCCTGCAGCAGGATGATGAGGATGCCTACAAGAGGCAGTTCTCGCGCTTC

ATCAAGAATGGCATAACACCTGACTCAATGGAAGAAATGTACAAGAAGTGCCATTAA-----------------------

--------------------------------------------------------------------------------

--------------------------------------------------------------------------------

--------------------------------------------------------------------------------

--------------------------------------------------------------------------------

--------------------------------------------------------------------------------

--------------------------------------------------------------------------------

----------------------------------------------------------------------------

>TERA00562

ATGACTCAAGTGACCAGTACTAGCTCTGGTGGTACTGTGGTGAACACGGGAAACAGAGACTGGAAGGCAGACCTACTCGG

CTGCTTCGACGACTGCAAAACAACACTGTGTGGCTTGTGCTGCCCCCTGTGTCTGGCGAGCAGAGTGTCTCAGAGAGTTG

GAGAACATTACTGTGTGCCGTGCTGCGTTCCCGGAGGCCTGATCGCCATCAGAACCAAAATGCGATTGATGCTTGGCATC

CAGGGTTCCATTTGTAACGACTGCGTGGCACTTTCCTGTTGCACACTGTGCGCCCTCTGTCAGATGCAGAGAGAACTAGA

CCTGAACAACTGGCCGCAGTAA----------------------------------------------------------

--------------------------------------------------------------------------------

--------------------------------------------------------------------------------

--------------------------------------------------------------------------------

--------------------------------------------------------------------------------

--------------------------------------------------------------------------------

--------------------------------------------------------------------------------

--------------------------------------------------------------------------------

--------------------------------------------------------------------------------

--------------------------------------------------------------------------------

--------------------------------------------------------------------------------

--------------------------------------------------------------------------------

----------------------------------------------------------------------------

>TERA00565

ATGTATGAAGATTTGCCTGAGCCCAATATGCCAAACCCTTATGCTCGTCCGTTTCGTAAATGTATTATCTGTGAACACAA

CATCGAGCTTGATTACAAGAATATTCGTCTTTTGAGCCAGTTTATATCACCACACACAGGTTTGATATATGGTCGACAGT

ACACAGGCTTGTGTCAATACATGCAGCGACGTGTTAGCAACTTGATCAAGCGTGCAAGATACTTTGGATTTCTTCCATAC

AAAAACAAGGATCCAAGATTTCTTGATGACCCAAAAATATAA--------------------------------------

--------------------------------------------------------------------------------

--------------------------------------------------------------------------------

--------------------------------------------------------------------------------

--------------------------------------------------------------------------------

--------------------------------------------------------------------------------

--------------------------------------------------------------------------------

--------------------------------------------------------------------------------

--------------------------------------------------------------------------------

--------------------------------------------------------------------------------

--------------------------------------------------------------------------------

--------------------------------------------------------------------------------

--------------------------------------------------------------------------------

----------------------------------------------------------------------------

>TERA00619

ATGACTACTATAAACAGTTTGAAGGGGTTAGTCAGCCTGGTTACAGGTGGCGCCAGTGGCTTGGGGAGAGCCACTGTTGA

GAGATTTGTGAAACAAGGTTCTCGTGTTGTCATCTGTGATCTTCCAAAGTCTCAGGGGGAAAAACTAGCTCAAGACCTTG

GAAGCAATGCTGCTTTCAGTCCAATGGATGTGACATCTGAAGAAGATGTAATGAAGGCCATGGAGCTTGCTAAGAAGCAA

TTTGGTGGTTTACATGTTGCTGTCAATTGTGCTGGTGTTGGTGTTGCTGTGGTTACGTATAATGCAAAGAAGAACAGAGT

ACACAAATTGGAAGAATTCCAGAGAGTTATAAATGTGAACCTGGTTGGAACATTTAATGTATAA----------------

--------------------------------------------------------------------------------

--------------------------------------------------------------------------------

--------------------------------------------------------------------------------

--------------------------------------------------------------------------------

--------------------------------------------------------------------------------

--------------------------------------------------------------------------------

--------------------------------------------------------------------------------

--------------------------------------------------------------------------------

--------------------------------------------------------------------------------

--------------------------------------------------------------------------------

--------------------------------------------------------------------------------

----------------------------------------------------------------------------

>TERA00643

ATGACATATGAACAGTCGCAGTATCCACACTTGATAGGAGTAACAAAGTCATGGAACTCATGGAACACAAGTAACCTTGT

TGGGGAGATGACCTTTGCTCCTCATACAACAGTGGAGGATACATTCATCAGAAAATTCATGGCAGGAGCATGGCACGATC

TCTTTCTTTCTGAAGTGATAATTAAGAGAAGACAAAACATGGTTATTATAGCTGGTTTTGTAAAGTAA------------

--------------------------------------------------------------------------------

--------------------------------------------------------------------------------

--------------------------------------------------------------------------------

--------------------------------------------------------------------------------

--------------------------------------------------------------------------------

--------------------------------------------------------------------------------

--------------------------------------------------------------------------------

--------------------------------------------------------------------------------

--------------------------------------------------------------------------------

--------------------------------------------------------------------------------

--------------------------------------------------------------------------------

--------------------------------------------------------------------------------

--------------------------------------------------------------------------------

----------------------------------------------------------------------------

>TERA00708

ATGAAGAAGACGCATGGAGAAATATTACTTTGTCAAGAGATTCGTGAGAAGAGGCCAACAAAAATCAAGAATATTGGCAT

CTGGTTGCGTTATGATTCTCGCAGTGGAACGCACAACATGTACAAAGAATACCGCGACCTTTCTGTTGCTGGAGCTGTCA

CTCAATGTTATCGTGACATGGCCGCAAGGCACAGAGCTAGAGCAGGATCAATCCAGGTGATCCGATGTGAAGTAATCCCA

GCCAGCAAGTGTCGAAGACCCTACATCAAACAGTTCCATGATAGCAAGATCAAGTTCCCTCTGCCACACAGAGTGTCCAG

GAGACTGTAA----------------------------------------------------------------------

--------------------------------------------------------------------------------

--------------------------------------------------------------------------------

--------------------------------------------------------------------------------

--------------------------------------------------------------------------------

--------------------------------------------------------------------------------

--------------------------------------------------------------------------------

--------------------------------------------------------------------------------

--------------------------------------------------------------------------------

--------------------------------------------------------------------------------

--------------------------------------------------------------------------------

--------------------------------------------------------------------------------

----------------------------------------------------------------------------

>TERA00761

ATGCCTAATGGAGCCCGAAAGAACTCCATTCTTTGGGAGTCGCTAATACACGCCCGTGAATGGTTGGCTGGAGCTACGTT

AATGAACATCCTGGATAAGATGGTAAAGGAATACGGAACTGATCCAGACGTGACAAATTTTCTGGATAACTATGACTTCC

ATTTCATTCCTGTCATGAATCCAGATGGGTATCAGCATAGCTGGGATGACGATCGTTACTGGAGGAAGAACAGAAGAGAC

AACGAAGGATCCGTGTGCTTCGGAGTGGACCTGAACAGAAACTGGGACAGCAACTGGAGCGGTCCCGGTGCCAGTTAA--

--------------------------------------------------------------------------------

--------------------------------------------------------------------------------

--------------------------------------------------------------------------------

--------------------------------------------------------------------------------

--------------------------------------------------------------------------------

--------------------------------------------------------------------------------

--------------------------------------------------------------------------------

--------------------------------------------------------------------------------

--------------------------------------------------------------------------------

--------------------------------------------------------------------------------

--------------------------------------------------------------------------------

--------------------------------------------------------------------------------

----------------------------------------------------------------------------

>TERA00766

ATGCTTGGAATGATCGATCCAGATGAAAAAACGCCCTCTGGTGAACCACTGACGTGCAGAGCTGTGTTTGTCATTGGCCC

AGACAAGAAGCTGAAGGCTTCAATCTTGTATCCAGCAACAACTGGAAGGAACTTTGATGAAATCCTTCGGTTGATCGACT

CGCTGCAGTTGACTGCTGTGAAGAAAGTGGCGACACCTGCTGACTGGAAGCCTGGCATGCCTGTCATGGTGGTGCCCTCG

CTGTCGAAACAAGAGGCGGCCAGCCTCTTCAAATCGATCGACACCAAACAGCTGCCGTCGAAACAGGAGTACCTGCGTAC

AACCACGGACTACTAA----------------------------------------------------------------

--------------------------------------------------------------------------------

--------------------------------------------------------------------------------

--------------------------------------------------------------------------------

--------------------------------------------------------------------------------

--------------------------------------------------------------------------------

--------------------------------------------------------------------------------

--------------------------------------------------------------------------------

--------------------------------------------------------------------------------

--------------------------------------------------------------------------------

--------------------------------------------------------------------------------

--------------------------------------------------------------------------------

----------------------------------------------------------------------------

>TERA00785

ATGTCCTGTTTGATGCTTCCAACCCAATCATACAAAGTGAAAACTTCTCTGTACAAACGATGTAAACATTGTTATTTTGT

CCGTAGACAAGGTCGTTTGTTTGTTGAATGTAAAGCAAAGGGTAGACATAAGCAGATGCAGAAAATGTCAAAGTAA----

--------------------------------------------------------------------------------

--------------------------------------------------------------------------------

--------------------------------------------------------------------------------

--------------------------------------------------------------------------------

--------------------------------------------------------------------------------

--------------------------------------------------------------------------------

--------------------------------------------------------------------------------

--------------------------------------------------------------------------------

--------------------------------------------------------------------------------

--------------------------------------------------------------------------------

--------------------------------------------------------------------------------

--------------------------------------------------------------------------------

--------------------------------------------------------------------------------

--------------------------------------------------------------------------------

----------------------------------------------------------------------------

>TERA00824

ATGTCCGGAGGTATCGAGGTTCTCGCCCTCAAAGAGGAGGACGTCACCAAGTTCTTGGCTTGTGGTACTCACCTTGGCTC

CAACAATGTCGACTTCCAGATGGAGCAGTATGTCTACAAACGTAAACCAGATGGTGTTTACATTCTGAACTTAAGGAAGA

CCTGGGAGAAGCTTCTTCTGGCAGCTCGTGCCATTGCAGCCATTGAGAACCCAGCTGATGTCTGTGTCATATCAGCTCGT

CCTTATGGACAACGTGCTGTGCTGAAGTTTGCTTCATATGTTGGTGCCACACCTGTTGCCGGAAGATTCACACCTGGAGC

ATTTACCAATCAGTTCCAGGCTGCCTTCCGTGAACCTCGTCTGCTTGTTGTCACAGATCCTCGTATTGACCATCAGCCAG

TAACAGAAGCATCGTATGTCAATATTCCTGTCATCGCTATGTGCAACACTGACTCGCCTCTGAAGTTTGTTGACATTGCT

ATCCCATGTAACAACAAGAGTCAGCACTCCGTTGGCCTGATGTGGTGGATGCTGGCACGTGAGGTACTCCGTCTGCGTGG

TACAATTTCACGTGAGGTACCATGGGATGTCATGGTTGATCTGTTCTTCTACAGGGATCCAGAAGAGGCTGAGAAGGAAG

AGCAAGCAGCTCTGGAGGCCAAGCAGCAGTAA------------------------------------------------

--------------------------------------------------------------------------------

--------------------------------------------------------------------------------

--------------------------------------------------------------------------------

--------------------------------------------------------------------------------

--------------------------------------------------------------------------------

--------------------------------------------------------------------------------

--------------------------------------------------------------------------------

----------------------------------------------------------------------------

>TERA00831

ATGTCGCATCGCAAGTTCTCCGCGCCTCGTCACGGATCGCTGGGCTTCTTGCCCAAAAAGCGAAGTAGTAGGCATCGGGG

TAAGGTTAAAGCCTTTCCGAAGGATGACAAAAACAAGCCATTACACCTGACTGCTTTCTTGGGATACAAAGCAGGCATGA

CCCACATCGTTAGGGATGTAGACAGGCCCGGATCAAAGGTTAATAAGAAGGAAGTGGTGGAACCTGTCACCATCATCGAG

ACGCCTCCAATGATAATTGTTGGTGTGGTGGGCTACATTGAGACTCCTCATGGTTTGAGGACCTTCAGGACTCTGTTTGC

TGAGCATCTGAGTGACGAATGCAAGCGTCGCTTTTACAAGAACTGGTACAAGTGCAAGAAGAAGGCATTCACCAAGGCCT

GCAAAAAGTACCAGGATGAACTGGGCAAGAAGGAGATCCAGAAGGATTTGAACAAGATGAAGAAATACTGCACTGTCATA

AGGGTGATTGCCCATACCCAGATGAAGATGCTGAAGAAACGCCAGAAGAAGGCCCACATCATGGAGATCCAAATTAATGG

TGGGACAATTGCTGATAAGGTTGACTGGGCGTATGCACATTTTGAGAAGCCAGTACAAGTGTCTGATGTGTTTGCCCAGG

ATGAGATGATAGATATCATTGGTGTGACAAAGGGCAAGGGATTCAAAGGTGTCACTTCACGTTGGCATCCAAAGAAACTG

CCACGCAAAACCCACAAGGGTCTGCGTAAGGTTGCCTGTATTGGAGCCTGGCATCCTGCCAGAGTGGCCTTCTCTGTAGC

TCGTGCTGGACAAAAGGGTTACCACCATCGTACAGAGATGAACAAGAAGATCTACCGTATCGGCCAGGGTATCCACATGA

AGGATGGCAAACTAGTGAAAAACAATGCCTCCACTGAAAGTGATGTTACTGAGAAGACCATCACACCCATGGGTGGATTC

CCTCATTATGGAGAAGTGAATAATGACTTCATCATGATTAAGGGCTGCTGTGTTGGACCCAAGAAGAGGGTGCTTACACT

TCGCAAGTCTTTGCTGGTACACACAAAGAGGCGTGCTCTGGAGAAGATCACTCTGAAGTTCATCGATACATCATCCAAGT

TTGGTCATGGACGCTTCCAGACCCACCAGGAGAAGCGTACTTTCATGGGTCCACTGAAGAAGGACAAGTAA---------

--------------------------------------------------------------------------------

----------------------------------------------------------------------------

>TERA00833

ATGCATGACTTCCAGTGCTGCTCCACGGAGGACCGCAAGGAGATGCAGGCTCTGTGGCACGAGATCTGGAGCGCTCAGTT

CACCGGAAGGAGAGTTCAGGTCGCTCTGTCCGTGTTCGAGGATCTGTTCGAACGTGAACCTGACGCAAAGAACCTGTTCA

AGCGTGTCAACGTCGATGACATGAACAGCCCGGAGTTCAAGGCTCACTGCATCCGTGTGGTTAACGGTCTGGACACAGCC

ATTAGCCTGCTGGATGATCCCTTCGTCATGCTTCACCAACTGGAACATCTCGGCAAGCAGCATCAAGTCAGAGACGGCGT

CAAGAAGGAACATTTCGACATGATGGCTCGATCCTATCTGAAGGTCATGCCACAGGTCAGCTCCTGCTTCAACGCCGACG

CCTGGTCCCGCTGCTTCGATGGCATAGCTCACAAGATCGCCAGCTACCTGTAA---------------------------

--------------------------------------------------------------------------------

--------------------------------------------------------------------------------

--------------------------------------------------------------------------------

--------------------------------------------------------------------------------

--------------------------------------------------------------------------------

--------------------------------------------------------------------------------

--------------------------------------------------------------------------------

--------------------------------------------------------------------------------

--------------------------------------------------------------------------------

--------------------------------------------------------------------------------

----------------------------------------------------------------------------

>TERA00842

ATGTTCCCATGTGTGTGCTACGTACAAGGCGACCCAATGGTGGAGACATACGACAACACTCTGAAACTGATCTTTGACGA

GCAGAAAACCTTGTTGTCACGTGGAATGAATCCAGACGAGATGGACAGGTGCAAGTTCCAGTCACGTGTCCAGACAGATG

ATGACTATCCCGGAAAGGAAGGACAGGTCTATGTTTAA------------------------------------------

--------------------------------------------------------------------------------

--------------------------------------------------------------------------------

--------------------------------------------------------------------------------

--------------------------------------------------------------------------------

--------------------------------------------------------------------------------

--------------------------------------------------------------------------------

--------------------------------------------------------------------------------

--------------------------------------------------------------------------------

--------------------------------------------------------------------------------

--------------------------------------------------------------------------------

--------------------------------------------------------------------------------

--------------------------------------------------------------------------------

--------------------------------------------------------------------------------

----------------------------------------------------------------------------

>TERA00843

ATGTTGGCACAGAGACTACATGGATGGTTGCCAATCTCAGCCATGCATTATGTGGCTGATCTGCTCAAAATGCCCAAGAT

GAGAGTCTATGAAGTGGCTACATTTTATACAATGTTCAACAGAGAACCCATTGGCAAGTACCATATACAAATTTGCACAA

CCACACCATGTATGTTAGGTGGAGTAGGCTGTGGACCAATCCTAGAGGCTATTAAAAAACAATTAGGAATCGAAGTTGGA

GAAACAACAGCAGATAAGATGTTTACTTTATCAGAAGTGGAATGCCTTGGAGCCTGTGTTAATGCTCCTATGGTTCAGAT

CAATGATGATTACTATGAGGATCTGTAA----------------------------------------------------

--------------------------------------------------------------------------------

--------------------------------------------------------------------------------

--------------------------------------------------------------------------------

--------------------------------------------------------------------------------

--------------------------------------------------------------------------------

--------------------------------------------------------------------------------

--------------------------------------------------------------------------------

--------------------------------------------------------------------------------

--------------------------------------------------------------------------------

--------------------------------------------------------------------------------

--------------------------------------------------------------------------------

----------------------------------------------------------------------------

>TERA00845

ATGATCCGCATTGCCCTTGCTCTGTGTCTCGTGGCCGCTGCCACGGCGAACTCCTGCTGCAGTGCAGGAGACATGAAGAA

GGTCAGAGACTCGTGGAACAGCCTCTGGAACAACGCCGACTCTGCCACCACCAAGGTCATCTTCGGCAGAGAGATCTTCC

ACAAGCTTTTCGAGAAGTTCCCTGACTCCAAGGGTATGTTCAGCCGAGTGAACGTTGACGACACCAAGAGTCCGGAATTC

AGCGGCCACATGCTCCGTGTGTTGAACGGCCTTGACCTTTTCATCAACCTGCTCGGAGAAGACGACGCTCTGGACGCTCA

GATTGAACACCTGAACAGGCAGCACTTGAACTACGACGGCATGAAGGCTGCCTACCTCTGGGGTATGCTTGACATCCTGA

CCGAGTCTCTTCCTGGAGTCCTTGACGACTACGATGCTCTTTCCTTCAAGAACTGCTTGGTCCGCGTCTTCGACGACTAC

ACCAAAGGACTGCCTTAA--------------------------------------------------------------

--------------------------------------------------------------------------------

--------------------------------------------------------------------------------

--------------------------------------------------------------------------------

--------------------------------------------------------------------------------

--------------------------------------------------------------------------------

--------------------------------------------------------------------------------

--------------------------------------------------------------------------------

--------------------------------------------------------------------------------

--------------------------------------------------------------------------------

----------------------------------------------------------------------------

>TERA00865

ATGGACCTAAGAGCCAGTTACGACAAGTTTGTTAAAGGAGACCAAGCCACCAGCAAAGACATCACAAAATGGTTTAAAGA

CGCCGGCGTATTGACGAAGAAAACCTGTAACTCTAACAACTTGGATATCGCCTTCAGCAAAGTGAAGGAAAAGGGCAAAA

CTAAGATCACCTTCTAA---------------------------------------------------------------

--------------------------------------------------------------------------------

--------------------------------------------------------------------------------

--------------------------------------------------------------------------------

--------------------------------------------------------------------------------

--------------------------------------------------------------------------------

--------------------------------------------------------------------------------

--------------------------------------------------------------------------------

--------------------------------------------------------------------------------

--------------------------------------------------------------------------------

--------------------------------------------------------------------------------

--------------------------------------------------------------------------------

--------------------------------------------------------------------------------

--------------------------------------------------------------------------------

----------------------------------------------------------------------------

>TERA00891

ATGTGGGACTACAGTGACATTAGTGCCCTCGATTTTGACAGAGCGTGGACTCAGGTTAAGGACACGATTTTGGAGATATT

CGCTGGTCCACCGACCACCGGTCTATTCTCGCCTTCGGTACAGAAGACCCTGTACGATACCCAGAAGTTGGCCATGGCAC

GGATTCCTCAGATAAGTGAGATGCACATCGGCCTTCCGAACATCCACGCCTATAAATATGACTTCAGCAAACACCCAAGA

GTTGGCATTGAAGCAAACGATGAAGTCTTCCAGCCAACGGACAAGCCTTCGGGTGACATATAA-----------------

--------------------------------------------------------------------------------

--------------------------------------------------------------------------------

--------------------------------------------------------------------------------

--------------------------------------------------------------------------------

--------------------------------------------------------------------------------

--------------------------------------------------------------------------------

--------------------------------------------------------------------------------

--------------------------------------------------------------------------------

--------------------------------------------------------------------------------

--------------------------------------------------------------------------------

--------------------------------------------------------------------------------

--------------------------------------------------------------------------------

----------------------------------------------------------------------------

>TERA00948

ATGGGAATACTGAAGCCAAACCCTTATTATGGTTCTTTTCCAAAAATCTCAGCTGCTGTCTTGATTGCTTACTTTGCTGG

AAAATTTTCATACCAGAGTGCTTGCAATGAGAAATTCTTACGATTGGAGAACAGCCCGATTGCAGACATTATATAA----

--------------------------------------------------------------------------------

--------------------------------------------------------------------------------

--------------------------------------------------------------------------------

--------------------------------------------------------------------------------

--------------------------------------------------------------------------------

--------------------------------------------------------------------------------

--------------------------------------------------------------------------------

--------------------------------------------------------------------------------

--------------------------------------------------------------------------------

--------------------------------------------------------------------------------

--------------------------------------------------------------------------------

--------------------------------------------------------------------------------

--------------------------------------------------------------------------------

--------------------------------------------------------------------------------

----------------------------------------------------------------------------

>TERA00967

ATGGCTACCTTACTCCGCCTGTGGCCATTGAAGCGTGGCCTTGGGTGTCCAGGGTTCCTTGGAGCTCTGAGTACTCGGGC

TCAGTCAACGGCTCCAGAAAAAATTCAGGTGTTTGTCAATGGCAAGCCAGTGCAGGTTGAGCGAACTGCAACAGTTCTAC

AGGCCTGTGCTGAAGGAGGAGTTCAAATACCTCGTTTCTGTTTCCATGAACGTCTCTCAATAGCTGGCAACTGTAGAATG

TGTTTAGTGGAAGTGGAAGGATCACCAAAGCCTATTGCATCTTGTGCTATGCCAGTGATGAAGGATATGAAAATAAAAAC

AGACTCACCTGCTACAAGAAAAGCAAGGGAAGGTGTGATGGAGTTCCTGTTAGTTAATCACCCTTTAGACTGTCCAATCT

GTGACCAGGGAGGTGAATGTGACCTTCAGGACCAGTCCATGAATTATGGCAGTGATCGTAGCAGGTTTACTGACATGTAA

--------------------------------------------------------------------------------

--------------------------------------------------------------------------------

--------------------------------------------------------------------------------

--------------------------------------------------------------------------------

--------------------------------------------------------------------------------

--------------------------------------------------------------------------------

--------------------------------------------------------------------------------

--------------------------------------------------------------------------------

--------------------------------------------------------------------------------

--------------------------------------------------------------------------------

----------------------------------------------------------------------------

>TERA00968

ATGGTAACATCCCAGTATGGTTGGTCTGCTAACATGGAGCGCATCATGAAGGCACAGGCCCTTAGGGACAACAGTACCAT

GGGTTACATGGCAGCAAAGAAGCACCTGGAGATCAATCCAGACCACAGCATAATGCAAGCTCTGAAGGAGAAGGCCGAGG

CAGACAAGAATGACAAGTCCGTTAAGGATTTGGTCAACTTGCTCTTTGAAACTTCTCTCCTGTCATCAGGCTTCACATTG

GAAGATCCTCAGATTCATGCCAACAGAATCCACAGAATGATCAAGTTGGGACTTGGCATTGAGGATGATGGCTAA-----

--------------------------------------------------------------------------------

--------------------------------------------------------------------------------

--------------------------------------------------------------------------------

--------------------------------------------------------------------------------

--------------------------------------------------------------------------------

--------------------------------------------------------------------------------

--------------------------------------------------------------------------------

--------------------------------------------------------------------------------

--------------------------------------------------------------------------------

--------------------------------------------------------------------------------

--------------------------------------------------------------------------------

--------------------------------------------------------------------------------

----------------------------------------------------------------------------

>TERA00970

ATGAAGGGCAATTATTCGGAGCGAGTGGGCGCCGGTGCTCCGGTTTACCTAGCCGCCGTTCTGGAGTACCTGGCTGCCGA

GGTATTGGAGTTGGCAGGAAACGCCGCTAGAGACAACAAGAAGTCCAGAATCATACCCCGACACCTGCAGCTTGCTATCC

GGAACGACGAGGAACTGAACAAACTGCTGTCTGGCGTCACGATTGCCCAGGGAGGTGTCCTGCCGAACATCCAAGCCTAA

--------------------------------------------------------------------------------

--------------------------------------------------------------------------------

--------------------------------------------------------------------------------

--------------------------------------------------------------------------------

--------------------------------------------------------------------------------

--------------------------------------------------------------------------------

--------------------------------------------------------------------------------

--------------------------------------------------------------------------------

--------------------------------------------------------------------------------

--------------------------------------------------------------------------------

--------------------------------------------------------------------------------

--------------------------------------------------------------------------------

--------------------------------------------------------------------------------

----------------------------------------------------------------------------

>TERA00981

ATGGCGGGCGAAGTTTTGGTGGATGCATTGCCATATTACGACCAAGGTTATGATGAACCAGGAGTTCGAGAAGCGGCATT

GGCCCTGGTAGAAGAGGAAACTCGACGATACAGACCGACCAAGAACTATCTTGAATATTTGCCTTCAGCTTCTTACAGTG

CATTTGAAACGGAAACAATGAAAACCGAATTTGAAAGGCTACAGGCTAGATTACCAATGGACATGTTGAGCATGAAAAGA

TATGAATTACCACAACCTACAGCAGGTCGCATGAATGATGTAGCTGCATGGATGGAATGTGTTGACAATTCACAGGCCCA

ACTGGAACATCAGGCACTAAGGATAGCCAACCTGGATCTCATGTCTCAGTATGGTGCAGATTCGTGGAAGATGTACAATG

ATATTCTACAGAAAATGGTTGAAGCCTCTCAAAAACAGCTGGTTGAAATCAGGAAACAGATCCAAGATATAAACTGGAGA

CGTAAAGGTGAACAGTGTGAAGCTGGAGCCAAGTTAAAACAGTTAGAGGAAAGTTGGGTAGGCCTTGTGTCCAAGAATTA

TGAAATAGAAAGAGCTTGTGTTGACTTAGAGAATGAAATCGAGATGCTACAACAGAAGAAAAAACAGAGAGCAAAGCAAC

GATAA---------------------------------------------------------------------------

--------------------------------------------------------------------------------

--------------------------------------------------------------------------------

--------------------------------------------------------------------------------

--------------------------------------------------------------------------------

--------------------------------------------------------------------------------

--------------------------------------------------------------------------------

--------------------------------------------------------------------------------

----------------------------------------------------------------------------

>TERA01006

ATGAATATGACCATAAACTTTGGTCCACAGCACCCAGCTGCTCATGGTGTACTAAGACTGGTGTTAACTCTTGATGGGGA

AACTGTAGTTAGGGCTGACCCTCACATTGGATTGTTGCACAGAGCCACTGAGAAACTGATAGAATACAAGACATACCTAC

AGGCTCTGCCATACTTTGACAGATTGGACTACTGTTCCATGATGGTCAATGAGCAGGCTTACTCCCTGGCTGTAGAAAAA

CTACTGAACATTGAGATCCCAGAGAGGGCAAAATGGATACGAACCATGTATGCTGAGATCACACGTATACTGAACCACAT

TCTGGGCATTGCCTCACATGCTCTGGATATTGGTGCCATGACACCATTTTTCTGGTTGTTTGAGGAGAGAGAGAAGCTAT

GTTAA---------------------------------------------------------------------------

--------------------------------------------------------------------------------

--------------------------------------------------------------------------------

--------------------------------------------------------------------------------

--------------------------------------------------------------------------------

--------------------------------------------------------------------------------

--------------------------------------------------------------------------------

--------------------------------------------------------------------------------

--------------------------------------------------------------------------------

--------------------------------------------------------------------------------

--------------------------------------------------------------------------------

----------------------------------------------------------------------------

>TERA01038

ATGAAGATCTTGCAGGATTACAAGTCGCTGCAGGATATCATTGCCATCCTTGGTATGGATGAATTGTCTGAGGATGACAA

ACTGACTGTGGCCAGAGCTCGCAAGATCCAGAGATTCCTGAGCCAGCCTTTCCAAGTTGCTGAGGTGTTCACAGGCTCTG

CTGGCAAACTTGTGCCACTGAAGGAAACAATCCGTGGATTCAAAGAAATCCTCAATGGTACATATGATCATCTGCCTGAA

GTAGCATTCTATATGGTTGGAGGTATCGAAGAAGTTGTGCAGAAGGCTGATAGACTTGCTGAGGAACGTGCATAA-----

--------------------------------------------------------------------------------

--------------------------------------------------------------------------------

--------------------------------------------------------------------------------

--------------------------------------------------------------------------------

--------------------------------------------------------------------------------

--------------------------------------------------------------------------------

--------------------------------------------------------------------------------

--------------------------------------------------------------------------------

--------------------------------------------------------------------------------

--------------------------------------------------------------------------------

--------------------------------------------------------------------------------

--------------------------------------------------------------------------------

----------------------------------------------------------------------------

>TERA01073

ATGAACATGGTGGTAGATAGACCAGTCATGGTTCTTATCAATAAGAGGGGACTGAGGAAACGAAAGACTGTGATGAGCTC

TCGGTTGGAGAGGCTTCCCGATAACACGACACCTGTCTGGCAGCTGGACCTGACAAGCACGGGCTGTTTTGTGTAA----

--------------------------------------------------------------------------------

--------------------------------------------------------------------------------

--------------------------------------------------------------------------------

--------------------------------------------------------------------------------

--------------------------------------------------------------------------------

--------------------------------------------------------------------------------

--------------------------------------------------------------------------------

--------------------------------------------------------------------------------

--------------------------------------------------------------------------------

--------------------------------------------------------------------------------

--------------------------------------------------------------------------------

--------------------------------------------------------------------------------

--------------------------------------------------------------------------------

--------------------------------------------------------------------------------

----------------------------------------------------------------------------

>TERA01081

ATGAACATGCCGAGACAGATAATGGAAATAAAGGACTTTCTCCTTACGGCCAGGAGACCTGATGCTCACTCTGTAAAGAT

CAAGAAGAACAAGGACAATGTCAAGTTTAAGGTGCGATGCAGTCGTTTCCTGTACACCTTGGTCATACACGACACAGAGA

AGGCAGAGAAACTGAAGCAGTCCCTGCCACCAGGTCTCTCCGTCAAGGAACTGAAGTCTAAATCTTAA------------

--------------------------------------------------------------------------------

--------------------------------------------------------------------------------

--------------------------------------------------------------------------------

--------------------------------------------------------------------------------

--------------------------------------------------------------------------------

--------------------------------------------------------------------------------

--------------------------------------------------------------------------------

--------------------------------------------------------------------------------

--------------------------------------------------------------------------------

--------------------------------------------------------------------------------

--------------------------------------------------------------------------------

--------------------------------------------------------------------------------

--------------------------------------------------------------------------------

----------------------------------------------------------------------------

>TERA01092

ATGGGAACAAAGCGATTGTGTCATATTCGTGGCAAGCTTCGAAAGAAGGTTTGGATCAACACATCTGACATAATCCTCAT

TGGTCTTCGTGACTATCAAGACAACAAAGCTGATGTCATTCTAAAGTATACAGCTGATGAGGCCAGGAACTTGAAGGTTT

ATGGAGAACTGCCAGAATCAGCCAAAATCAATGAGATTGATGTTGATGAGGATGAGGAATGCAATGTCTGCTTCCAAGAT

GTTGAAGTGGATGATGATGACATCGATGAGTAA-----------------------------------------------

--------------------------------------------------------------------------------

--------------------------------------------------------------------------------

--------------------------------------------------------------------------------

--------------------------------------------------------------------------------

--------------------------------------------------------------------------------

--------------------------------------------------------------------------------

--------------------------------------------------------------------------------

--------------------------------------------------------------------------------

--------------------------------------------------------------------------------

--------------------------------------------------------------------------------

--------------------------------------------------------------------------------

--------------------------------------------------------------------------------

----------------------------------------------------------------------------

>TERA01106

ATGGCTGGCTTTCCAACTGGAATGAAATGGAGCTTTATGAATAAACCAAGTGATGGCAGGCCAAAGTACCTGGTTGTTAA

TGCTGATGAGGGTGAACCAGGGACATGTAAGGATCGTGAAATAATGAGGCATGATCCACACAAATTGATTGAAGGATGTC

TGGTTGCTGGTAGTGCTATGGGAGCATGTGCTGCTTACATTTATATCCGTGGTGAGTTCTACAATGAAGCCTCCAGTATG

CAAATAGCTATCAAGGAGGCATATGATGCTGGTCTCATTGGCAAGAATGCTTGTGGATCTGGCTATGACTTTGATGTTTA

TCTCCACCGTGGTGCTGGGGCATACATCTGTGGTGAAGAAACTTCTCTGATTGAATCCTTAGAAGGAAAGCCTGGTAAAC

CACGTTTAAAGCCTCCATTCCCAGCAGATGTTGGTGTTTTTGGATGTCCAACAACAGTAAACAATGTCGAGACAATCGCT

GTGTCACCAACAATCTGTTAA-----------------------------------------------------------

--------------------------------------------------------------------------------

--------------------------------------------------------------------------------

--------------------------------------------------------------------------------

--------------------------------------------------------------------------------

--------------------------------------------------------------------------------

--------------------------------------------------------------------------------

--------------------------------------------------------------------------------

--------------------------------------------------------------------------------

--------------------------------------------------------------------------------

----------------------------------------------------------------------------

>TERA01112

ATGGCAGATCAGCTGACAGAAGAACAGATTGCTGAATTCAAGGAGGCTTTCTCCCTCTTTGACAAGGATGGTGATGGTAC

AATCACCACAAAGGAGTTGGGTACTGTTATGAGATCACTGGGGCAGAACCCAACAGAGGCCGAGCTCCAGGACATGATCA

ATGAAGTTGATGCTGATGGTAATGGCACAATTGATTTCCCTGAGTTCCTGACAATGATGGCCAGAAAAATGAAGGACACC

GACAGTGAGGAGGAGATTCGTGAAGCATTCCGTGTCTTTGACAAAGATGGCAATGGTTTCATAAGTGCTGCTGAGCTGAG

GCATGTGATGACAAACCTGGGTGAGAAGTTAACTGATGAAGAGGTCGACGAGATGATTAGGGAAGCTGACATTGATGGTG

ATGGCCAAGTCAACTATGAAGAGTTTGTTACAATGATGACATCCAAGTAA------------------------------

--------------------------------------------------------------------------------

--------------------------------------------------------------------------------

--------------------------------------------------------------------------------

--------------------------------------------------------------------------------

--------------------------------------------------------------------------------

--------------------------------------------------------------------------------

--------------------------------------------------------------------------------

--------------------------------------------------------------------------------

--------------------------------------------------------------------------------

--------------------------------------------------------------------------------

----------------------------------------------------------------------------

>TERA01119

ATGAAGTGTGGCTTCAGTAGAACAATGGTTGGCCTACTGAAGGAGGCAGGAGTCGAGTTTGATTATTTTGATATCTTACA

AGATGAAGAAGTACGTCAGGGATTGAAGTCTTACTCTGACTGGCAAACATATCCACAGCTGTATGTCAATGGAAAATTGA

TTGGTGGAGTAGATATAGCCAAGCAACTGAAGGAATCTGGAGAACTTTAA------------------------------

--------------------------------------------------------------------------------

--------------------------------------------------------------------------------

--------------------------------------------------------------------------------

--------------------------------------------------------------------------------

--------------------------------------------------------------------------------

--------------------------------------------------------------------------------

--------------------------------------------------------------------------------

--------------------------------------------------------------------------------

--------------------------------------------------------------------------------

--------------------------------------------------------------------------------

--------------------------------------------------------------------------------

--------------------------------------------------------------------------------

--------------------------------------------------------------------------------

----------------------------------------------------------------------------

>TERA01120

ATGGATGTTGTTCCCAAAACAGCAGAGAATTTCCGAGCTTTGTGTACGGGAGAGAAGGGTTTTGGCTACAAGGGTAGCAC

TTTCCATAGAATAATTCCAAACTTCATGTGTCAGGGAGGTGACTTCACCAACCACAATGGCACTGGTGGCAAGAGCATCT

ATGGTGAGAAGTTCGAAGATGAGAACTTCCAGCTGAAGCATGATGGTCCAGGCGTGTTGTCCATGGCCAATGCTGGTCCA

AACACAAATGGTTCTCAGTTCTTCCTGTGTACAGTAAAAACTGACTGGCTTGATGGCAAGCATTAA--------------

--------------------------------------------------------------------------------

--------------------------------------------------------------------------------

--------------------------------------------------------------------------------

--------------------------------------------------------------------------------

--------------------------------------------------------------------------------

--------------------------------------------------------------------------------

--------------------------------------------------------------------------------

--------------------------------------------------------------------------------

--------------------------------------------------------------------------------

--------------------------------------------------------------------------------

--------------------------------------------------------------------------------

--------------------------------------------------------------------------------

----------------------------------------------------------------------------

>TERA01161

ATGGGTGCCATGGCTGGCCAGTTCAGGAGCTCCGTTTGGGATCCAGTCTTAATTATTGCCCAGATAATGACAATGCAGAG

CATCTTTTATGTCAGCCTTGGTGTCTGGATTTGCATACTGGATGTTCTTCTTGGGACAAGCAGGTCCTTGGAACAGTTAT

TTGGCTATGAGGCTACACAGTTTACTGATTAA------------------------------------------------

--------------------------------------------------------------------------------

--------------------------------------------------------------------------------

--------------------------------------------------------------------------------

--------------------------------------------------------------------------------

--------------------------------------------------------------------------------

--------------------------------------------------------------------------------

--------------------------------------------------------------------------------

--------------------------------------------------------------------------------

--------------------------------------------------------------------------------

--------------------------------------------------------------------------------

--------------------------------------------------------------------------------

--------------------------------------------------------------------------------

--------------------------------------------------------------------------------

----------------------------------------------------------------------------

>TERA01186

ATGTGTGATGTGAGAGACCCATTTTCTGTTAGAGATGCTGTGTCTGAGTGCATTGACAGATTTCAGCTGCCACACATCAT

CATTAACAATGCTGCCGGTAACTTCATTAGCCCAACAGAAAGGCTTTCACCTAATGCCTGGAAGACGATCATTGACATTG

TCCTGAATGGAACAGCCAATGTGACTTTGGATTAA---------------------------------------------

--------------------------------------------------------------------------------

--------------------------------------------------------------------------------

--------------------------------------------------------------------------------

--------------------------------------------------------------------------------

--------------------------------------------------------------------------------

--------------------------------------------------------------------------------

--------------------------------------------------------------------------------

--------------------------------------------------------------------------------

--------------------------------------------------------------------------------

--------------------------------------------------------------------------------

--------------------------------------------------------------------------------

--------------------------------------------------------------------------------

--------------------------------------------------------------------------------

----------------------------------------------------------------------------

>TERA01213

ATGGGTTACTATCCCCGCTTCCGTGAGCTGGCCACGGCCATCAAAGAGGAAGCTCCGGAAGTCGAAGTAACGGGAAATGT

CGGCAGAAGATCATCCTTTGAGATAACCATTAACGGTAAACTTGTGTTCAGCAAGTTACAGCTTGGTGGTTTTCCCTAA-

--------------------------------------------------------------------------------

--------------------------------------------------------------------------------

--------------------------------------------------------------------------------

--------------------------------------------------------------------------------

--------------------------------------------------------------------------------

--------------------------------------------------------------------------------

--------------------------------------------------------------------------------

--------------------------------------------------------------------------------

--------------------------------------------------------------------------------

--------------------------------------------------------------------------------

--------------------------------------------------------------------------------

--------------------------------------------------------------------------------

--------------------------------------------------------------------------------

--------------------------------------------------------------------------------

----------------------------------------------------------------------------

>TERA01214

ATGGTCAAGGAAGACAAAGCTACTTGGAAGTCAAACTACTTCCTTAAACTTGTGCAACTGTTTGATGAGTATCCAAAATG

CTTCATTGTTGGTGTGGACAATGTCGGCTCAAAGCAGATGCAGCACATCCGCATAGCTCTGCGTGGCCAGGCAGTTGTGA

TGATGGGGAAGAACACCATGATGAGGAAGGCAATACGAGGTCATGTTGAGACTAATCCAGCCCTGGAAAAGCTGTTGCCT

CACATTAAGGGCAATGTTGGTTTTGTCTTTACCAAATGTGATCTGTCTGAAATCAGAAAGGTCATCAGCGAAAACAGGGT

GGCAGCTCCTGCTAGGGCTGGTGCCATTGCCCCACTGGATGTGACACTGCCTGCCCATAACACAGGACTTGGCCCAGAGA

AGACCTCATTCTTCCAGGCTCTTGCTATTCCAACAAAGATTTCCAGAGGCACCATTGAAATCCAGAGTGATGTCAAGTTG

ATTCGTGAAGGAGAGAAAGTTGGAGCCTCTGAAGCAACCCTGCTGAACATGTTGAAGATCTCGCCATTCACATATGGTTT

GGTCATCAAGCAGGTCTATGACTCGGGCACCATCTTTGATCCAGCCATTTTGGATGTGTAA-------------------

--------------------------------------------------------------------------------

--------------------------------------------------------------------------------

--------------------------------------------------------------------------------

--------------------------------------------------------------------------------

--------------------------------------------------------------------------------

--------------------------------------------------------------------------------

--------------------------------------------------------------------------------

--------------------------------------------------------------------------------

----------------------------------------------------------------------------

>TERA01225

ATGTTTCGTAACCATTATGACAACGATGTCACAATATGGAGCCCTCAGGGGCGAATCCATCAGATTGAATATGCTATGGA

AGCCGTCAAACAAGGTTCTGCTACTGTAGCTTTGAAATCTAAAACGCATGCAGTCATCGTAGCATTAAAGAGAGCACCGT

CTGAGCTATCAGCACACCAGAAGAAAGTTATTCCTATTGATAATCACATTGGTATTTCAATAGCAGGACTTACAGCTGAT

GCTAGGTTGTTAAGCAAATTCATGAGGACTGAATGCCTCAACTCACGCTATGCATATGATCAGCCACTGCCAGTAGCAAA

ACTGGTATCTATGATTGGGAGCAAATCTCAGATTCCAACTCAGAGATATGGACGAAGGCCATTTGGTGTTGGTCTGCTCA

TTGCTGGATATGATCATGAAGGGCCACACGTCTACCAAACATGTCCATCAGCAAATTTCTTTGATTGCAAGGCAATGGCC

ATTGGCGCCAGATCTCAGTCAGCACGGACTTATCTTGAGAAGCATCTTGATGAGTTCCCAGACTGTGACTTATAA-----

--------------------------------------------------------------------------------

--------------------------------------------------------------------------------

--------------------------------------------------------------------------------

--------------------------------------------------------------------------------

--------------------------------------------------------------------------------

--------------------------------------------------------------------------------

--------------------------------------------------------------------------------

--------------------------------------------------------------------------------

--------------------------------------------------------------------------------

----------------------------------------------------------------------------

>TERA01247

ATGCTGTCCAAGATGGCGTTGATAAGTTATGTATACCGCACGGCTATCAAAAGAACGTCGGCCTTCGCCTTCGTTATAAT

GGGAGGAGCATATGTTTTTGAGCGGGTGTTTGACCAGGCAGTTGACTCATTTTATGAATATCACAACAGAGGAAAATTGT

GGATTCATATAAGAGATAAATAA---------------------------------------------------------

--------------------------------------------------------------------------------

--------------------------------------------------------------------------------

--------------------------------------------------------------------------------

--------------------------------------------------------------------------------

--------------------------------------------------------------------------------

--------------------------------------------------------------------------------

--------------------------------------------------------------------------------

--------------------------------------------------------------------------------

--------------------------------------------------------------------------------

--------------------------------------------------------------------------------

--------------------------------------------------------------------------------

--------------------------------------------------------------------------------

--------------------------------------------------------------------------------

----------------------------------------------------------------------------

>TERA01252

ATGACTGAGGCTCGTAAGATCTGGTGCTTTGGGCCTGATGGCACAGGACCAAACATGGTCATTGACTGCACAAAGGGTGT

CCAATACCTGAATGAAATCAAAGACAGTGTTGTGGCTGGCTTCCAGTGGGCCAGCAAGGAGGGTGTACTCTGTGAAGAGA

ATATGAGAGGAATCCGCTTCAACATTCTTGATGTCACACTGCATGCTGATGCCATTCACCGTGGTGGTGGCCAGATCATC

CCAACAACCAGAAGGTGTCTCTATGCCTGTGTGCTGACAGCTGAACCAAGATTGATGGAACCAATATACCTGGTCGAGAT

CCAGTGTCCCGAGCAAGCTGTTGGTGGCATCTATGGTGTGCTGAACAGAAGACGAGGTGTTGTCATTGAGGAAAACCAGG

TGGTGGGAACCCCAATGTTCCAGGTCAAGGCATACCTTCCTGTAAATGAATCATTCGGATTCACTGCTGACCTGAGGTCT

AACACTGGTGGCCAGGCGTTCCCACAGTGTGTGTTTGATCACTGGCAGATCCTCCCAGGTGATCCTTTTGTGGATAACTC

CAAGCCTAATTAA-------------------------------------------------------------------

--------------------------------------------------------------------------------

--------------------------------------------------------------------------------

--------------------------------------------------------------------------------

--------------------------------------------------------------------------------

--------------------------------------------------------------------------------

--------------------------------------------------------------------------------

--------------------------------------------------------------------------------

--------------------------------------------------------------------------------

----------------------------------------------------------------------------

>TERA01257

ATGTGGCGATCAGCACAGGCTGCCACACACCAAGAAACATTACTTAACGTCCCCGAAACAAAGGTGTCTAAGCTGGCAAA

TGGCTTGAGGATTGCAACAGAGGACTCTGGTATACCAACAGCTACTGTGGGATTGTGGATTGATGCAGGAAGTCGTTTTG

AGAATGACAACAACAATGGCACAGCTCACTTCCTTGAGCACATGGCATTCAAGGGAACATCTAAGAGATCTCAAATGGAC

CTGGAGCTAGAGGTTGAGAATATGGGAGCCCATCTTAATGCCTACACCTCCAGAGAGCAGACAGTTTATTATGCAAAATG

CTTCTCAAAGGATATTCCCAAGGCTGTTGATATTCTCTCGGATATCATCCAGAACAGTACCTTGGGTGAACAGGAGATAG

AACGTGAGAGGGGCGTCATCCTCCGTGAGATGCAGGAAGTAGAAACCAACCTACAGGAAGTTACCTTTGATTATCTCCAT

GCCACTGCTTACCAGGGCACAGCACTGGGAAGAACTATCCTAGGACCAACTGAGAACATCAAATCATAA-----------

--------------------------------------------------------------------------------

--------------------------------------------------------------------------------

--------------------------------------------------------------------------------

--------------------------------------------------------------------------------

--------------------------------------------------------------------------------

--------------------------------------------------------------------------------

--------------------------------------------------------------------------------

--------------------------------------------------------------------------------

--------------------------------------------------------------------------------

----------------------------------------------------------------------------

>TERA01261

ATGACAGCTTGGCATTTTGATAAGAAGTATGGCAAGCCAGGTGCTGCATATGATGCTTTTAAGCGAGCTGTTGCCAATCC

TGAAATATTCGATGAGTGTGACCTTGATCCAAAGACCAGATCTGTATTAATTGACAACATCAAGCAAAGATTGGCACCAC

AATCTGTTAAAATCCGTTCAGACATAGATGTGTCTTGTTATGGATATGAAGGAGTAGATGCTGTAAAAAATGCATTACGA

GCTGGACTGTAA--------------------------------------------------------------------

--------------------------------------------------------------------------------

--------------------------------------------------------------------------------

--------------------------------------------------------------------------------

--------------------------------------------------------------------------------

--------------------------------------------------------------------------------

--------------------------------------------------------------------------------

--------------------------------------------------------------------------------

--------------------------------------------------------------------------------

--------------------------------------------------------------------------------

--------------------------------------------------------------------------------

--------------------------------------------------------------------------------

--------------------------------------------------------------------------------

----------------------------------------------------------------------------

>TERA01288

ATGCAGATACCATACACAATGATGAAATTTGCCTGCTTTGAGAGAACTGTGGAAGCCTTATACAAATATGTTGTACCTAA

ACCACGATCAGAATGCTCAAAGGGAGAACAGCTGATTGTGACATTTGTTGCTGGCTACATAGCTGGTGTATTCTGTGCTA

TTGTATCTCACCCTGCTGATACAATTGTGTCCAAGCTGAACCAGCAGAAAGGCAGTACCTTCTTTGAGATTGGAAAGAAC

TTGGGATTCTATGGTATGTGGAAAGGACTGTTCCCAAGGATCATCATGATTGGTACTCTGACAGCCCTGCAGTGGTTCAT

CTATGATTCTGTTAAGGTCTACTTCCGCATGCCACGCCCACCACCACCTGAAATGCCAGAGAGCTTAAAGAGGAAGCTTG

AAGCAAAGAACCAGTAA---------------------------------------------------------------

--------------------------------------------------------------------------------

--------------------------------------------------------------------------------

--------------------------------------------------------------------------------

--------------------------------------------------------------------------------

--------------------------------------------------------------------------------

--------------------------------------------------------------------------------

--------------------------------------------------------------------------------

--------------------------------------------------------------------------------

--------------------------------------------------------------------------------

--------------------------------------------------------------------------------

----------------------------------------------------------------------------

>TERA01291

ATGGCTGCCAAGGCATACAGAGTCTATTTCAGTGCCGGACCCAAGGATGAGGATGATGATTACATTGTGGACCACTCAAT

TGTCATGTACCTCGTGAATCCAGATGGTGAATTTGTTGACTACTATGGCCAGAACAAGACAGCTGAGGAAATTGAGTCAA

GCATTGCTGTACACATGATGTAA---------------------------------------------------------

--------------------------------------------------------------------------------

--------------------------------------------------------------------------------

--------------------------------------------------------------------------------

--------------------------------------------------------------------------------

--------------------------------------------------------------------------------

--------------------------------------------------------------------------------

--------------------------------------------------------------------------------

--------------------------------------------------------------------------------

--------------------------------------------------------------------------------

--------------------------------------------------------------------------------

--------------------------------------------------------------------------------

--------------------------------------------------------------------------------

--------------------------------------------------------------------------------

----------------------------------------------------------------------------

>TERA01300

ATGAAGAAAATATGCCGTCTATACAAGACGTCACAATGCCGAGTTTATCGCGGTATTGTATACGATTGGGTCCTGGGCCT

CGGTGGTGCTCCTGATGAGCCCCACGCCGTCAACGTGTCTGAAAAGACAGAGGGCAACGGTGTGGGGGTTTTGAAAGAGC

CCCCTGTCAGCGCCAATGGCCTGCCGGAAAACGTAAACACATTGGCGAAGGCACAGACTGCCTCGAGTTTGTGCTTCCGT

ATCACTCTGACAGATTGCTTGGAGGTCAGCTGGGAAACGCTGCTCCTTGGTCGGAAGCTCATTGTGGAGATCCCCAATGG

AATTCTGCCCGAGGGCTCCAAAGAGAGCTTTGTTACTCTTCTGGAGTACGCTGAGGAAGCTCTGAAATGCACTCATGTCA

TTGTTTGCTTTAAGAAGGCTAGGTTAGATCGAGCGTGTTTGATCCGTACGATGATGTTCTTGGGTTTTGTGGTATAA---

--------------------------------------------------------------------------------

--------------------------------------------------------------------------------

--------------------------------------------------------------------------------

--------------------------------------------------------------------------------

--------------------------------------------------------------------------------

--------------------------------------------------------------------------------

--------------------------------------------------------------------------------

--------------------------------------------------------------------------------

--------------------------------------------------------------------------------

--------------------------------------------------------------------------------

----------------------------------------------------------------------------

>TERA01303

ATGTGTACAGCAAACAGAATGCTTAAACAGATGCTGTTCAGATACCAGGGCTATATTAGTGCAGCCTTAGTCCTTGGTGG

TGTAGATATCACAGGTCCTCATGTGTACAGTGTCTACCCACATGGATCAACAGATAGCTTACCATATGTTACAATGGGAT

CTGGATCTCTGGCTGCTATGTCTGTGTTTGAAGATAGATACAAAGTTGATATGGAGAAAGAATAA---------------

--------------------------------------------------------------------------------

--------------------------------------------------------------------------------

--------------------------------------------------------------------------------

--------------------------------------------------------------------------------

--------------------------------------------------------------------------------

--------------------------------------------------------------------------------

--------------------------------------------------------------------------------

--------------------------------------------------------------------------------

--------------------------------------------------------------------------------

--------------------------------------------------------------------------------

--------------------------------------------------------------------------------

--------------------------------------------------------------------------------

--------------------------------------------------------------------------------

----------------------------------------------------------------------------

>TERA01328

ATGACGATGGCTGAAGCTCTTCTCGAAGCTGTTGATAAAGCTGCTGGCATTGATCGTGACAAGATGGAGGCCCTTGAACT

GCTTGCCCAAGGCAAGCGTAACCTATTATGTGGAGAGATACCTAAGGCAGTTAATCAGCTGCAGGAAGTTTGTCAGAGAT

TGGCAGATAAATTTGGAGAAACGGCTGATGAGGTCGCTGATGCCTATTTTTATTATGGAAAAGCATTGTTGGAGTTGTCT

AGAATGGAAAGTGGCGTCTTGGGCAATGCATTACAAGGCATTGAAGATGATGATGCAGAG---AAATCTGATGAAAAGTC

ATCCACAGATGATGTGGTTGAAAAGGATGACCTGACAGAGGGTGAAACATCACAGGAGGAAGGTGTTGAATCTGAAGATA

TTGGTGATGAGGAGAATGAAGATCAATAA---------------------------------------------------

--------------------------------------------------------------------------------

--------------------------------------------------------------------------------

--------------------------------------------------------------------------------

--------------------------------------------------------------------------------

--------------------------------------------------------------------------------

--------------------------------------------------------------------------------

--------------------------------------------------------------------------------

--------------------------------------------------------------------------------

--------------------------------------------------------------------------------

--------------------------------------------------------------------------------

----------------------------------------------------------------------------

>TERA01332

ATGAAGACACGTGCAGAGATGCGTGGTGGTGGCAGGAAGCCATGGCCACAGAAACGTACAGGTCGAGCTCGCCATGGCAG

CATACGCTCACCTTTGTGGCACAAAGGTGGCAAGAGCTTTGGTCCACGAGGTCCGAAGTCTTTCTTCTTTATGTAA----

--------------------------------------------------------------------------------

--------------------------------------------------------------------------------

--------------------------------------------------------------------------------

--------------------------------------------------------------------------------

--------------------------------------------------------------------------------

--------------------------------------------------------------------------------

--------------------------------------------------------------------------------

--------------------------------------------------------------------------------

--------------------------------------------------------------------------------

--------------------------------------------------------------------------------

--------------------------------------------------------------------------------

--------------------------------------------------------------------------------

--------------------------------------------------------------------------------

--------------------------------------------------------------------------------

----------------------------------------------------------------------------

>TERA01367

ATGAAAATGGCGAGCGGACAGATGCCTCGTATTGACATGGATAAAGAATTCAACAAATTTAACGAAAGAGATCAGCACTT

CATCAAGAAGATCACGGAAATGAACAAGACCAGAGCACTGAATCGCTTTGTTATTCAGCACCGTAACAAGTGGACGGCCG

CTGTAATTACCGGAGCTGTCTTCGGCATTTATGTCTACACAATGTCTGCAGTTCAGCAGGAAGATTTCCTTGATGACTTT

TAA-----------------------------------------------------------------------------

--------------------------------------------------------------------------------

--------------------------------------------------------------------------------

--------------------------------------------------------------------------------

--------------------------------------------------------------------------------

--------------------------------------------------------------------------------

--------------------------------------------------------------------------------

--------------------------------------------------------------------------------

--------------------------------------------------------------------------------

--------------------------------------------------------------------------------

--------------------------------------------------------------------------------

--------------------------------------------------------------------------------

--------------------------------------------------------------------------------

----------------------------------------------------------------------------

>TERA01381

ATGAGAGAGAAGATGACTCAGATCATGTTTGAGACATTCAACTCGCCAGCCATGTATGTAGCCATCCAGGCTGTGCTCTC

TCTTTATGCCTCTGGTCGTACCACAGGTATTGTGTTGGATTCTGGTGATGGTGTCACCCACACTGTACCCATCTATGAGG

GTTATGCCCTGCCCCATGCTATCCTTCGTCTGGATCTGGCTGGCAGAGACCTGACAGACTATCTGATGAAGATCCTGACA

GAGCGTGGTTACAGCTTCACCACCACAGCCGAGCGTGAGATTGTCCGTGACATCAAGGAGAAGCTCTGCTATGTTGCCCT

TGACTTTGAGCAGGAGATGGCTACTGCTGCTGCCTCATCCTCCCTGGAGAAGAGCTATGAGCTGCCTGATGGCCAGGTCA

TCACCATTGGCAATGAGCGATTCAGGTGTCCAGAGGCCATGTTCCAGCCATCCTTCCTGGGCATGGAAGCCTGTGGCATC

CACGAGACAACATACAACAGCATCATGAAGTGTGATGTGGACATCCGTAAGGATCTGTATGCCAACACTGTCCTCTCTGG

TGGCAGCACCATGTTCCCTGGCATTGCTGACCGCATGCAGAAGGAGATCACTGCCCTGGCTCCACCTTCCATGAAGATCA

AGATCATAGCTCCACCTGAGCGCAAGTACTCTGTATGGATTGGCGGATCAATCCTTGCCTCTCTCTCCACCTTCCAGCAG

ATGTGGATCAGCAAGCAAGAGTATGACGAGTCTGGCCCCTCAATTGTTCACAGGAAGTGCTTCTAA--------------

--------------------------------------------------------------------------------

--------------------------------------------------------------------------------

--------------------------------------------------------------------------------

--------------------------------------------------------------------------------

--------------------------------------------------------------------------------

--------------------------------------------------------------------------------

----------------------------------------------------------------------------

>TERA01432

ATGTTCAAAATGATCATTTACAAGGATTTATTTACCGAAGATGAGATGTTTAGCGACATCTATCCTATGGAAGAAGTAGA

AGACGGCCTACTTTATAAAGTAACAGGAAAACTGGTTACAGAGAGCGACAAATTTGATGATGCCTTAATCGGTGCCAATG

CCTCAGCAGAAGAACAGACAGAGGAAGTAGACACGACAACGAGATCAGGAATCAACATTGTACTGAGTAACCGTCTTGTA

GAATTCCCACAGACAAAGAAGAGCTACCAAAGTCACCTCAAGGATTACATGAAGAGCTTAGCAGCCAAACTTGAGGATGC

TGAAAAGGAAAAATTTAAGGCCAAAGCACAAAATTTTGTTGGCAAAAAGTTGCTTCCAAAGTTTAAAGATCTACAATTTT

ACTGTGGAGAGTCGATGAAGCCAGATGGCATGTTGGCAATAGTGGAGTGGGAAGGTGACATACCATACATGTACTTCTTC

AAAGATGGCCTAATTCCAGAGAAAGTGTAA--------------------------------------------------

--------------------------------------------------------------------------------

--------------------------------------------------------------------------------

--------------------------------------------------------------------------------

--------------------------------------------------------------------------------

--------------------------------------------------------------------------------

--------------------------------------------------------------------------------

--------------------------------------------------------------------------------

--------------------------------------------------------------------------------

--------------------------------------------------------------------------------

----------------------------------------------------------------------------

>TERA01448

ATGTTTGGTCTGGCTGGCAAGTCTGAGTATGAAGAACATCGTTGTAACATGATTGTTGACTGTGTCAATGACATGGCCAA

TGCTGAGTTAAAACTGAAGTTTGCAAAAGATTCGGAGAAAGCTGAGATTGAGAAAAAATGGAAAGAAGAGCAAGAACCCA

ATTACTGTCGGATGTTGGAGAAGCTTCTCATCAGTAACAATGGTGGAGATGGATATTTTGTTGGAGATTCACTAACCTGG

GCTGATCTTGCAGTTTGTGTATGGATGGGTTGGGTCAGAGACTTGGATTACACACCATATCCAAAGTTGGCAGCACACAG

GGTCCGTGTGACAAGTCATCCTGGATTGGCAGCTTGGCTTGCCAAAAGACCGGAGACGTCCATGTAA-------------

--------------------------------------------------------------------------------

--------------------------------------------------------------------------------

--------------------------------------------------------------------------------

--------------------------------------------------------------------------------

--------------------------------------------------------------------------------

--------------------------------------------------------------------------------

--------------------------------------------------------------------------------

--------------------------------------------------------------------------------

--------------------------------------------------------------------------------

--------------------------------------------------------------------------------

--------------------------------------------------------------------------------

----------------------------------------------------------------------------

>TERA01452

ATGTTTTCGTTTGGCGCAATGGCGGCTGGGACTCTGTACACGTACCCGGAGAACTTCCGTGCTTATAAGGCGCTCATTGC

TGCCAAGTACAGCAAGGCAGCTTTGACAGTTGTTTCTGGTCCTCCCGACTTCAAATTCGGCGAAACTAACAAGACTGAAG

CATTTCTAAAGAAATTTCCGCTTGGCAAGGTCCCAGCATTTGAGAGCAAAGATGGAGTGATCTTGTTTGAGAGCAATGCC

ATTGCTTATTATGTTGCCAATGATGCCCTGCGTGGAGCCAATCTTACAGACTCAGCCTATGTTCAGCAGTGGATCAACTT

TGCAGACAATGAGATACTGCCAGCATCATGCACTTGGGTATTCCCCTGCATGGGAATCATGCAGTACAACAAGCAGAACA

CTGATAGAGCCAAGGAAGATATCAAGAAGGCTCTGAGTGTATTGAATGACTACCTAAGGACAAGGACATACCTAGTTGGT

GAAAGAATCTCTTTGGCTGACATCTGTGTGTGTTGTAATCTGCTGCATCTGTACCAGTGGGTTCTGGATCCAGAGTTCCG

TAAGCCATACCAGAATGTCAACAGGTGGTTTATTACCTTAATCAACCAGCCCGAGTTCAAAGAGGTCATCGGAGAATTCA

AAATGGCTGAGAAGATGGCTCAGTTTGATGCCAAGAAGTACCAAGAGCTATAA---------------------------

--------------------------------------------------------------------------------

--------------------------------------------------------------------------------

--------------------------------------------------------------------------------

--------------------------------------------------------------------------------

--------------------------------------------------------------------------------

--------------------------------------------------------------------------------

--------------------------------------------------------------------------------

----------------------------------------------------------------------------

>TERA01456

ATGCCGTACCAGATCCAGATGAGCATGGACAGATACTTTGACGTCCAGTTTCATGACCAGTACACGTACACACTGTTTAC

ACGTGGTGGTCCTTACTGGCAATTGTCTCATATTCCGCTGTCAAAGTTCTTCCTGGCAAGTAAAGGACGGATACAAGATT

AA------------------------------------------------------------------------------

--------------------------------------------------------------------------------

--------------------------------------------------------------------------------

--------------------------------------------------------------------------------

--------------------------------------------------------------------------------

--------------------------------------------------------------------------------

--------------------------------------------------------------------------------

--------------------------------------------------------------------------------

--------------------------------------------------------------------------------

--------------------------------------------------------------------------------

--------------------------------------------------------------------------------

--------------------------------------------------------------------------------

--------------------------------------------------------------------------------

--------------------------------------------------------------------------------

----------------------------------------------------------------------------

>TERA01471

ATGACTGTTCCTATTGCCAGTGAAGTATTCAAAAAGAGAGGTACTTATGACCCAAACAGGATATTTGGTGTCAGTACACT

AGATGTCGTTAGAGCAAACACATTTATTGCTGAAGCAAAGGATCTGGATGTGAGTCAGGTGAACTGCCCAGTTGTCGGTG

GTCATTCAGGTATCACAATCGTACCACTGATTTCTCAGTGCACCCCACCTGTATCCTTCCCACAGGAGGTCCGTGACAAA

TTGACAAAAAGGATTCAAAATGCTGGCACAGAAGTAGTTGAAGCCAAAGCTGGTGCTGGTTCAGCTACTCTGTCAATGGC

CTTTGCTGGAGCTAGATTCACTGACTCTGTGCTGCAAGCATTGGCTGGAGAGGAAGGTGTTGTAGAATGTGCTTTTGTCC

GTTCAGATGAAACAGAAGCCAAGTACTTCTCCACACCTCTGTTACTTGGGAAAAATGGTCTGGACAAGAACCTTGGCCTG

GGCAAGGTCCTGGACTATGAGCTAAACCTGATCAAAGCTGCCTTGCCAGAACTGAAGAAAAACTAA--------------

--------------------------------------------------------------------------------

--------------------------------------------------------------------------------

--------------------------------------------------------------------------------

--------------------------------------------------------------------------------

--------------------------------------------------------------------------------

--------------------------------------------------------------------------------

--------------------------------------------------------------------------------

--------------------------------------------------------------------------------

--------------------------------------------------------------------------------

----------------------------------------------------------------------------

>TERA01473

ATGCCTGGAAAACATGGTTTTCATGTCCACGAATTTGGTGACAACACCAACGGTTGCACCAGTGCTGGTGGACATTTCAA

TCCTCATGGGAAGGAACATGGTGCACCAGAGGATGAGAATAGACATGCTGGAGACCTCGGCAATGTTGTTGCTGGAGAAG

ATGGCAAAGCGGTCATCAACATGAAGGACAAGCTTGTGAAGTTGACTGGTCCAGACTCGGTTATTGGCAGGACTCTGGTT

GTGCATGTTGACGAGGATGACCTTGGTAGGGGTGGCCACGAGCAGAGCAAGATCACAGGCAATGCTGGTGGACGTCTTGC

ATGTGGTGTCATTGGTATTTAA----------------------------------------------------------

--------------------------------------------------------------------------------

--------------------------------------------------------------------------------

--------------------------------------------------------------------------------

--------------------------------------------------------------------------------

--------------------------------------------------------------------------------

--------------------------------------------------------------------------------

--------------------------------------------------------------------------------

--------------------------------------------------------------------------------

--------------------------------------------------------------------------------

--------------------------------------------------------------------------------

--------------------------------------------------------------------------------

----------------------------------------------------------------------------

>TERA01476

ATGGCCAAGTTGTCAAAACTTAGCAATGGGGTGATACTGGCTTCGATGGAGAACCACTTCCCAGTAGCCCAGGTGGCTGT

TTTGTACAATGCTGGACCACGTTATGAACCAGGTGGCCAGTGTGGCATCACTCACTGTCTGCGTAATGCATTCAGCCTCA

GCACTAAAAGTTCTACATCTTTTGGAATAACCAGGAGCATACAACAGATAGGTGGTAATATAGGATGTACTTCAACAAGA

GAACAGATGATCTACACCTTGGAGTGTCTGAGAGATGAAACAGATACTGGACTGAAAATCCTTGGTGACATCACCAGTAG

GCCAGCATTCATGCCTTGGGAACTGGATGCTTAA----------------------------------------------

--------------------------------------------------------------------------------

--------------------------------------------------------------------------------

--------------------------------------------------------------------------------

--------------------------------------------------------------------------------

--------------------------------------------------------------------------------

--------------------------------------------------------------------------------

--------------------------------------------------------------------------------

--------------------------------------------------------------------------------

--------------------------------------------------------------------------------

--------------------------------------------------------------------------------

--------------------------------------------------------------------------------

----------------------------------------------------------------------------

>TERA01498

ATGGTGCAGTTGGATTTCACACGAAATGTTAGCGGAATCTTCACTAAAGTGACTGCAAGCCAGCTGTGGGAAAGTGTTAG

CTCTGTCAGCAATGCAGGCAGGAAGAAAGGCAGAGGCCGTGCTGTTGGTCGTAAAAAGGTCACTGATTTAAATAAAGGAC

AAAAACTTGGTGATGGCAAAGCTAACATGGTGTGGCCAGGTCTCAATGCTCCTGTCATGCAGAATCGTGTGGTTGTTCGA

CACCAGCAACTAGCGAAAGATGACAACAGACAACAGAGATTGATAGAACTGAGGGAGAAGATGAGTCAAGTCAAGTATCC

TCCCATGCCTCCATTACTTCGAGGTTTTACTGGAGGCTAA----------------------------------------

--------------------------------------------------------------------------------

--------------------------------------------------------------------------------

--------------------------------------------------------------------------------

--------------------------------------------------------------------------------

--------------------------------------------------------------------------------

--------------------------------------------------------------------------------

--------------------------------------------------------------------------------

--------------------------------------------------------------------------------

--------------------------------------------------------------------------------

--------------------------------------------------------------------------------

--------------------------------------------------------------------------------

----------------------------------------------------------------------------

>TERA01530

ATGGATATCTATAAGAACGTGGACGTCTATCCAGCTGCTGAAGAGATTCCCGGAGTGAAGATCTTCAGATTTGAGTCGTC

GCTGTACTTTGCCAACGTGGAGCACTTTATACACAAACTATTCTCAAAGACCGGAGTCAATCCGAGAAAGCTTCTCTAA-

--------------------------------------------------------------------------------

--------------------------------------------------------------------------------

--------------------------------------------------------------------------------

--------------------------------------------------------------------------------

--------------------------------------------------------------------------------

--------------------------------------------------------------------------------

--------------------------------------------------------------------------------

--------------------------------------------------------------------------------

--------------------------------------------------------------------------------

--------------------------------------------------------------------------------

--------------------------------------------------------------------------------

--------------------------------------------------------------------------------

--------------------------------------------------------------------------------

--------------------------------------------------------------------------------

----------------------------------------------------------------------------

>TERA01573

ATGTGTTCACCCTTCACTGTGCTGCCTGCTTTGATGAATGAGTACAGGGTACCTGAGCTGAATGTGCAGAATGGTGTGCT

GAAATCTCTGTCCTTTATGTTTGAGTACATTGGAGAGATGAGCAAGGATTATGTTTATGCCGTCTCACCTCTGCTGGAGG

ATGCCTTGATGGACAGAGACCTTGTACACAGACAGACGGCCATGTCTGCCATTGGTCATCTGGCACTCGGAGTGTATGGC

TTTGGCTGTGAAGATGCTCTGAATCATATACTCAACTATGTATGGCCTAACATATTTGAGAGCTCACCACACGTTGTGCA

AGCTTTCATGGCAGCAATAGAAGGCATCATGGTTGGACTCGGACCTACAAAGATTCTACAGTACTGCATGCAGGGTTTGT

TCCATGCTGCATAA------------------------------------------------------------------

--------------------------------------------------------------------------------

--------------------------------------------------------------------------------

--------------------------------------------------------------------------------

--------------------------------------------------------------------------------

--------------------------------------------------------------------------------

--------------------------------------------------------------------------------

--------------------------------------------------------------------------------

--------------------------------------------------------------------------------

--------------------------------------------------------------------------------

--------------------------------------------------------------------------------

----------------------------------------------------------------------------

>TERA01589

ATGGATTATGACATGAAGAAGCCGCTCCAAGAAGCCATATCAGAGAGACTTCTAAGTTCTACTCTGATGAGTCAACAACT

TGAATACCTGACGGAGGTGGCCTTCTCACCTGAGGAGGGAGGCAGAGCAAAGGGAAAGAAATCGGCTATCCTGGTTGTAG

ACAACCAATCCATGTATCCAGAAGATGCCTAA------------------------------------------------

--------------------------------------------------------------------------------

--------------------------------------------------------------------------------

--------------------------------------------------------------------------------

--------------------------------------------------------------------------------

--------------------------------------------------------------------------------

--------------------------------------------------------------------------------

--------------------------------------------------------------------------------

--------------------------------------------------------------------------------

--------------------------------------------------------------------------------

--------------------------------------------------------------------------------

--------------------------------------------------------------------------------

--------------------------------------------------------------------------------

--------------------------------------------------------------------------------

----------------------------------------------------------------------------

>TERA01592

ATGAAACACAGCAATAAGGCTCAGATATCTAGTGACTACCCAATTGTGGATCATGACTATGATGCTGTTGTAGTTGGAGC

TGGTGGTGCTGGTTTGCGAGCAGCCTTTGGCTTGGCCAACCAAGGCTTTAAGACTGCCTGCATCACAAAACTGTTCCCAA

CCAGGTCACACACAGTAGCCGCCCAGGGAGGAATCAATGCAGCACTTGGAAATGTGGAGGATGACAAGTGGGAGTATCAC

TTCTTTGACACAGTCAAAGGCTCAGATTGGCTTGGAGACCAGAATGCTATCCAGTACATGTGTGAAGAGGCTCCGAAGGC

TGTTATTGAGCTGGAGAACTATGGCATGCCATTCAGTCGTCTGGAGGATGGCTCCATCTATCAGAGAGCCTTCGGAGGAC

AAAGCTTGGACTATGGCCGTGGTGGTCAGGCACACAGGGCCTGCTGTGTGGCTGACAGGACAGGTCATTCTATGCTTCAC

ACACTCTATTAA--------------------------------------------------------------------

--------------------------------------------------------------------------------

--------------------------------------------------------------------------------

--------------------------------------------------------------------------------

--------------------------------------------------------------------------------

--------------------------------------------------------------------------------

--------------------------------------------------------------------------------

--------------------------------------------------------------------------------

--------------------------------------------------------------------------------

--------------------------------------------------------------------------------

----------------------------------------------------------------------------

>TERA01594

ATGGATGAAATCAAGCCTGGTGTGGTAAGGACTCCTACACAGGTGAAAGACCAGCCAACTGTGGACTGGGATGCTGAATC

AGGGGCCTATTACACTCTGATCATGAATGATCCTGATGCTCCAAGTCGTCAGAATCCCAAGTTTGGTGAGTGGCATCACT

GGCTCGTGACAAACATCCCTGGAAACTCGGTGTCCAGTGGGGATGCCATGTCTGAGTACGTTGGTGCTGGCCCACCGAAA

GGCACTGGCCTCCACCGCTATGTCTTCCTGCTCTTCAAGCAGAAGGCCAAACAGGACTTTGCAGGATTGAACAAGTAA--

--------------------------------------------------------------------------------

--------------------------------------------------------------------------------

--------------------------------------------------------------------------------

--------------------------------------------------------------------------------

--------------------------------------------------------------------------------

--------------------------------------------------------------------------------

--------------------------------------------------------------------------------

--------------------------------------------------------------------------------

--------------------------------------------------------------------------------

--------------------------------------------------------------------------------

--------------------------------------------------------------------------------

--------------------------------------------------------------------------------

----------------------------------------------------------------------------

>TERA01598

ATGACCAAGAAACGCAGGAATAACGGACGTTCAAAGAAGGGCCGAGGACACGTACAACCTGTCCGCTGTACTAACTGTGC

CAGATGTGTACCAAAAGACAAGGCTATTAAAAAGTTTGTCATCCGAAACATAGTGGAGGCAGCAGCTGTTAGAGATATAG

CAGATGCATCTGTCTATGAAAGTTATGCTCTACCCAAGCTGTATGCAAAGCTTCACTACTGTGTATCATGTGCCATTCAC

AGCAAGGTAGTAAGGAACAGATCCCGAGAGGCCCGTAAGGATCGTACACCACCTCCCAGGTTCAGGCCTGGCATGAGGAA

TTAA----------------------------------------------------------------------------

--------------------------------------------------------------------------------

--------------------------------------------------------------------------------

--------------------------------------------------------------------------------

--------------------------------------------------------------------------------

--------------------------------------------------------------------------------

--------------------------------------------------------------------------------

--------------------------------------------------------------------------------

--------------------------------------------------------------------------------

--------------------------------------------------------------------------------

--------------------------------------------------------------------------------

--------------------------------------------------------------------------------

----------------------------------------------------------------------------

>TERA01606

ATGGTCAAAATGTCCTACGCATATCTTTTCAAATATATCATTATAGGCGATACAGGTGTTGGCAAGTCATGTCTACTGTT

GCAGTTCACAGATAAGAGATTTCAGCCAGTCCATGATCTTACCATAGGTGTAGAGTTTGGAGCTCGTATGATTACAATAG

ATGGCAAACAAATCAAGTTACAGATTTGGGACACAGCTGGACAGGAATCATTTAGGTCGATTACTAGATCTTATTATAGA

GGAGCAGCTGGTGCTCTACTCGTTTATGATATTACTAGGAGGGATACATTTAATCATCTTACCACCTGGCTGGAAGATGC

CAGACAACACTCCAGCTCCAACATGGTGATTATGTTGATTGGAAACAAAAGTGATTTGGAAGCAAGGAGAGATGTTAAAA

AAGAAGAAGGTGAAGCCTTTGCTAGAGAACATGGACTCATCTTTATGGAAACATCGGCTAAAACTGCCGCCAATGTGGAA

GAGGCTTTCATTAATACTGCAAAAGAAATCTACCAGAAAATACAGGATGGAGTATTTGACATTAATAATGAATAA-----

--------------------------------------------------------------------------------

--------------------------------------------------------------------------------

--------------------------------------------------------------------------------

--------------------------------------------------------------------------------

--------------------------------------------------------------------------------

--------------------------------------------------------------------------------

--------------------------------------------------------------------------------

--------------------------------------------------------------------------------

--------------------------------------------------------------------------------

----------------------------------------------------------------------------

>TERA01616

ATGGCATCAGGTGTCAAGGTAACAGATGAGTGTAAGCAGGCTTATGTTGACATCCAGCTTGGACACAAATACCAGTATGT

TGTCTATAAACTGAATGATAAAATGACCGAAGTTGTAGTGGAAAAGAAAGGAGAAATTGGTGCTCCATATGAGGAATTTG

TGCAGATATTAAAAGATGCTGAAGAGAAGGCTGAATGTCGTTATGGTGTCTTTGATGTTGCATATACTTCAAAAGAAGGC

CATCCCAAGAGCAAAATAGCATTTATATTCTGGGCTCCAGAGTCAGCAAAAATTAAACAGAAGATGGTGTATTCAGCCAG

CCAGAATGTGCTAAAGAACACACTTGGCCAGGGAATCCACAAGTTTGTCCAGGCCAATGACCACAGTGAGCTGTCTTGGG

ACTATCTGCTAGAGGAATGTCAGCGAAATGCCAGGGACTAA---------------------------------------

--------------------------------------------------------------------------------

--------------------------------------------------------------------------------

--------------------------------------------------------------------------------

--------------------------------------------------------------------------------

--------------------------------------------------------------------------------

--------------------------------------------------------------------------------

--------------------------------------------------------------------------------

--------------------------------------------------------------------------------

--------------------------------------------------------------------------------

--------------------------------------------------------------------------------

----------------------------------------------------------------------------

>TERA01668

ATGCCTGACAATATTGCCCTGATGGTGGCCAGTACTTTGATTGGTACCTGGGATCGTTCACATGGTGGTGGCACCAACTT

GGCCAGTGCTCTGGCTGCCAAGTGTGCCCAGGGTAACCTGTGTCATAGTTTCCAGTCATTTAACACTTGCTACACCGATA

CTGGTCTATGGGGCATGTACTTTGTTACTGACAAATTGAACATTGAAGACATGGTGTTCCATGTCCAGAATGAATGGATG

AAACTCTGCACTAATGTAACTGAGTTTGAAGTTCAGAGAGCCAAAAATTTGCTAAGAACTAACATGTTGCTGATGCTAGA

TGGTTCAACCCCAGTCTGTGAGGATATTGGAAGACAAATGCTGTGCTATGGACGTCGTGTACCACTGCCTGAGCTGGATG

CCAGGATCAATGCAATCACAGCCAAGACAGTCCGTGAAGTCTGCATGAAGTACATCTATGATAAATGTCCTGCTGTTGCT

GGTGTTGGACCAGTAGAACAGCTACCTGACTACAACCGTATCCGCAGTGGAATGTACTGGCTCAGATTGTAA--------

--------------------------------------------------------------------------------

--------------------------------------------------------------------------------

--------------------------------------------------------------------------------

--------------------------------------------------------------------------------

--------------------------------------------------------------------------------

--------------------------------------------------------------------------------

--------------------------------------------------------------------------------

--------------------------------------------------------------------------------

--------------------------------------------------------------------------------

----------------------------------------------------------------------------

>TERA01683

ATGAAAATGGTGAATGTTCCAAAACAGCGCAGAACATTCTGCAAGGGCAGAAAGTGCCGCAAGCACACAGTTCACAAAGT

TACCCAGTACAAGAAGGGTAGAGACTCCCTGTCTGCTCAGGGTAAAAGGCGATATGATAGAAAACAGGCAGGATATGGTG

GACAAACAAAACCCATCTTCAGGAAGAAGGCCAAGACAACAAGGAAAATTGTATTGAGGATGGAGTGTACTAAATGCAAG

TATAAGAAACAGTTGCCTATCAAGCGTTGCAAACACTTTGAGTTGGGAGGTGACAGAAAGAAGAAGGGCCAGATGATCCA

GTACTAA-------------------------------------------------------------------------

--------------------------------------------------------------------------------

--------------------------------------------------------------------------------

--------------------------------------------------------------------------------

--------------------------------------------------------------------------------

--------------------------------------------------------------------------------

--------------------------------------------------------------------------------

--------------------------------------------------------------------------------

--------------------------------------------------------------------------------

--------------------------------------------------------------------------------

--------------------------------------------------------------------------------

--------------------------------------------------------------------------------

----------------------------------------------------------------------------

>TERA01723

ATGAAGAAATCGAAGAAGGCCATAGAGAGTATTAATTCTCGATTGGCTCTTGTTATGAAGAGTGGTAAATATGTGCTTGG

CTATAAGCAGACGTTGAAGACACTCCGACAGGGCAAAGCCAAGCTTGTCATCATTGCAAACAACACGCCACCACTCAGAA

AGAGTGAGATAGAGTACTATGCAATGTTGGCCAAAACAGGTGTTCATCATTATAATGGCAACAACATTGAACTTGGCACA

GCTTGTGGAAAGTACTTCAGAGTTACAACACTGAGTATCACTGATCCTGGTGACTCTGACATCATCCGTAGCATGCCAGC

TGGTGAACAGTAA-------------------------------------------------------------------

--------------------------------------------------------------------------------

--------------------------------------------------------------------------------

--------------------------------------------------------------------------------

--------------------------------------------------------------------------------

--------------------------------------------------------------------------------

--------------------------------------------------------------------------------

--------------------------------------------------------------------------------

--------------------------------------------------------------------------------

--------------------------------------------------------------------------------

--------------------------------------------------------------------------------

--------------------------------------------------------------------------------

----------------------------------------------------------------------------

>TERA01749

ATGGACGTCGCCTACAGACACCTAGAAAGGGTTCTCAATCATGAAAAGATCCTGCAGAAGGTAGCATACAACCGTTACTA

CGAGAAGCCATACCTGACGCGAGGCAGATTGGCATGGGAACGTTGTAAGAGGATCTACAACTCGGAAATGGCTCGCAAAC

TGCAATTCATTTCCCGAAAGAACAGAGTAGATCCATGGCCACGGTAA---------------------------------

--------------------------------------------------------------------------------

--------------------------------------------------------------------------------

--------------------------------------------------------------------------------

--------------------------------------------------------------------------------

--------------------------------------------------------------------------------

--------------------------------------------------------------------------------

--------------------------------------------------------------------------------

--------------------------------------------------------------------------------

--------------------------------------------------------------------------------

--------------------------------------------------------------------------------

--------------------------------------------------------------------------------

--------------------------------------------------------------------------------

--------------------------------------------------------------------------------

----------------------------------------------------------------------------

>TERA01753

ATGAAAATGGCCATTCCACCATCATATGCCGATCTTGGGAAGTCTGCCCGCGATCTCTTCAGCAAAGGCTTCAATTATGG

ATTTTACAAGATTGAAGCCAAAACAAAGACTGCTAATGGAGTAGAATTCACATCCACTGGAAGTTCTAATCATGACACTG

CCAAGTTTAGTGGGAGTTTAGAATCAAAGTACAAGTGGAGTGAATATGGTTTGACTTTCACTGAGAAGTGGAATACTGAC

AACACATTGGCAACGGAGATAACTATTGAGGACCAGCTGTTGAAGGGTCTGAAACTATCCTTTGATACCCAGTTTGCACC

ACAGACTGGCAAGAAGAGTGGCAAGATCAAGACTGGCTACAAACAGGAATACTTCAATGCCAATTGTGATGTGGACTTCG

ACTTTGCAGGTCCCACCATAAATGGAGCAGCTGTTGTTGGCTATAGTGGCTGGTTAGCTGGATACCAGTTCTCCTTTGAT

ACATCTAAATCTAAGCTGTCCAGAAGCAACTTTGCTGTTGGATACAGCACTGATGACTTCTAA-----------------

--------------------------------------------------------------------------------

--------------------------------------------------------------------------------

--------------------------------------------------------------------------------

--------------------------------------------------------------------------------

--------------------------------------------------------------------------------

--------------------------------------------------------------------------------

--------------------------------------------------------------------------------

--------------------------------------------------------------------------------

--------------------------------------------------------------------------------

----------------------------------------------------------------------------

>TERA01757

ATGAAAATGGACAAGAACAAGCTGAACGATCTGGCAGGAAGACTGCAAAAGGGTGGTAGAGGTGCCGGCATCGGTTTGGG

CTTCTTGGCTGCTGCTGGTGGTGTGGCATACGGCCTCTATCAGTCGTTGTATACCGTTGAGGGTGGTCACAGAGCAATCA

TATTTAACCGTATAGGAGGTATCCAACAGGACATATACAGAGAAGGGTTACATTTTCGGATACCGTGGTTGCAGTATCCA

ATCATATATGACATTCGTGCCAGACCTAGAAAAATTTCTTCTCCAACTGGTAGCAAAGATTTACAGATGGTCAACATCTC

TCTTCGTGTCTTGTCACGGCCTGATGCCACTAAGTTACCAACTATTTACCGTCAACTGGGCACAGATTATGATGAGAGAG

TGCTGCCATCTATATGCAATGAAGTACTGAAGAGTGTTGTAGCCAAGTTCAATGCTTCACAGCTGATCACACAACGTTCC

CAGGTATCACTGTTGTAA--------------------------------------------------------------

--------------------------------------------------------------------------------

--------------------------------------------------------------------------------

--------------------------------------------------------------------------------

--------------------------------------------------------------------------------

--------------------------------------------------------------------------------

--------------------------------------------------------------------------------

--------------------------------------------------------------------------------

--------------------------------------------------------------------------------

--------------------------------------------------------------------------------

----------------------------------------------------------------------------

>TERA01775

ATGGAGGAATTTGTGCAGTTCATCAGACACGCCATGATGGACAGGAACTCAAACGAATTCATTGAGTTCTACCAATTCCT

CATCAATTGCTTTGTTCGTGCTGACTCCAACTTAGACGGACAGGTGTCCGTGGACGAATTCGATCAACTTGTTGAAGAGG

CGGCATACCTGCCGAGGAAGCACGGCTTTGCACCGAAAACAGAAGAACTCTACAAGACACCGGAAGCCAGGAAGGAAGCT

CGTGATAAGCTATTCAAGGAGATGGATTCGAACAAAGACGGCCACATCACCTTTGAAGAATGGCTGGCCTTCGCCTAA--

--------------------------------------------------------------------------------

--------------------------------------------------------------------------------

--------------------------------------------------------------------------------

--------------------------------------------------------------------------------

--------------------------------------------------------------------------------

--------------------------------------------------------------------------------

--------------------------------------------------------------------------------

--------------------------------------------------------------------------------

--------------------------------------------------------------------------------

--------------------------------------------------------------------------------

--------------------------------------------------------------------------------

--------------------------------------------------------------------------------

----------------------------------------------------------------------------

>TERA01776

ATGGAAGGTGATGTTGTCAAGAGGACAGGAGCTATTGTAGATGTGCCTGTTGGTATGGAGTTACTGGGCCGTGTTGTTGA

TGCTTTGGGCAACCCCATTGATGGCAAGGGTCCACTGAACACAGCTTCTCGTGCCCGTGTTGGTACCAAGGCTCCAGGTA

TCATCCCTCGCATCTCTGTCAAGGAGCCAATGCAGTCTGGGATCAAGGCTGTTGACAGTTTGGTGCCAATTGGACGAGGA

CAGCGAGAATTGATCATTGGAGACAGACAGACTGGTAAAACTGCTGTAGCCATTGACACAATCATCAATCAGAAACGATT

TAATGATGGTCAGGATGAGAAGGCCAAGCTGTACTGTATCTATGTGGCCATTGGACAGAAGAGATCTACCGTGGCCCAAA

TTGTCAAACGCTTGATTGACACTGATTCAATGAGGTACACCATCGTAGTCAGTGCTACAGCCTCAGATGCTGCACCACTG

CAGTACTTGGCACCATACTCTGGCTGTGCCATGGGAGAGTATTTCAGAGACAATGGCAAACACGCTTTGATCATATACGA

TGATCTGTCCAAACAGGCTGTGGCTTACCGTCAGATGTCGCTGCTGCTGCGTCGTCCTCCCGGTCGTGAGGCCTACCCTG

GTTAA---------------------------------------------------------------------------

--------------------------------------------------------------------------------

--------------------------------------------------------------------------------

--------------------------------------------------------------------------------

--------------------------------------------------------------------------------

--------------------------------------------------------------------------------

--------------------------------------------------------------------------------

--------------------------------------------------------------------------------

----------------------------------------------------------------------------

>TERA01781

ATGAGGAGAGTCAGTTTTGATCCCCTGGCTTTGCTGTTGGATGCTTCACTGGAAGGAGAGGTAGAACTTGTCAAGAAGAC

TGCCCAAGAGGTTCCTGATCCAAGCTGCCCAAACGATGAAGGGATAACCGCCCTCCACAATGCTATCTGTGCTGGACATT

TTGAGATTGTCAAGTTCTTGGTTGAGTTTGGCTGTAACGTTAACGCTCCAGACAGCGATGGATGGACTCCACTTCACTGT

GCTGCCTCCTGTAACAACCTACCAATGGTTAAGGTGCTCGTAGAACATGGAGCTTGTATATTTGCTACAACAATCAGTGA

TCACGAAACAGCTGCTGAGAAATGTGAAGAAGATGAAAATGGCTATGATGGCTAA-------------------------

--------------------------------------------------------------------------------

--------------------------------------------------------------------------------

--------------------------------------------------------------------------------

--------------------------------------------------------------------------------

--------------------------------------------------------------------------------

--------------------------------------------------------------------------------

--------------------------------------------------------------------------------

--------------------------------------------------------------------------------

--------------------------------------------------------------------------------

--------------------------------------------------------------------------------

--------------------------------------------------------------------------------

----------------------------------------------------------------------------

>TERA01785

ATGGGCTTGTCAATGGGAACCATGATCTGTGGCTGGGATAAAAAGGGTCCTGGCCTGTACTATGTGGACAGTGACGGTAC

CAGAGTGACCAATGACATGTTTTCTGTTGGCTCTGGTTCCACATACGCCTATGGTGTGTTGGACAGTGGATACAAATGGG

ACCTTGGTACAGATGAAGCTTATGATCTTGCACAGCGAGCCATTTATCATGCCACACATAGAGATGCTTACAGTGGTGGT

GTTGTAAACATGTACCACATGAAGGAGACGGGATGGATTAAAGTGTCGCAGAATGATGTCGGGGATCTCCACTATCAGTA

CAAGGATGAGAAGTAA----------------------------------------------------------------

--------------------------------------------------------------------------------

--------------------------------------------------------------------------------

--------------------------------------------------------------------------------

--------------------------------------------------------------------------------

--------------------------------------------------------------------------------

--------------------------------------------------------------------------------

--------------------------------------------------------------------------------

--------------------------------------------------------------------------------

--------------------------------------------------------------------------------

--------------------------------------------------------------------------------

--------------------------------------------------------------------------------

----------------------------------------------------------------------------

>TERA01815

ATGTACAAGGAGCAGACAAGATGGTTCTCATGTACCAGCAGATGGTTTGCTGCCCCAGCAGGACAAAAGAAACAATTTGT

GAGAGACAAGCCTCATATCAATATAGGAACAATTGGCCATGTTGACCATGGAAAAACAACACTTTCTGCTGCCATTACAA

AAGTTCTTGCAAAGAAGAATCAGGCAACATTTATGGCTTATGACGCCATTGACAAAGCCCCCGAAGAGAAGAAAAGAGGC

ATTACTATCAATGCTGCTAGTCTGGAATACTCTACGGATAAGAGGCATTACGGACACATAGACTGTCCGGGACATGCAGA

TTACATTAAGAACATGATCACAGGAACAGCCATTATGGATGGTGCCATCCTTGTTGTTGCAGCAACAGATGGAACTATGC

CTCAGACAAGGGAACACTTGCTGCTAGCTAAACAGATTGGTATCACACATTTATAA------------------------

--------------------------------------------------------------------------------

--------------------------------------------------------------------------------

--------------------------------------------------------------------------------

--------------------------------------------------------------------------------

--------------------------------------------------------------------------------

--------------------------------------------------------------------------------

--------------------------------------------------------------------------------

--------------------------------------------------------------------------------

--------------------------------------------------------------------------------

--------------------------------------------------------------------------------

----------------------------------------------------------------------------

>TERA01820

ATGGAAAAACAGATTCAGTATGAGATCATGAAACATGGACCAGTAGAAGCAGCATTCAATGTCTACTCGGATTTCCCCAC

CTACAAGTCAGGTGTGTACAAGCACACTACAGGTGACCTGTTGGGAGGTCATGCTGTCAAGATGATCGGTTGGTAA----

--------------------------------------------------------------------------------

--------------------------------------------------------------------------------

--------------------------------------------------------------------------------

--------------------------------------------------------------------------------

--------------------------------------------------------------------------------

--------------------------------------------------------------------------------

--------------------------------------------------------------------------------

--------------------------------------------------------------------------------

--------------------------------------------------------------------------------

--------------------------------------------------------------------------------

--------------------------------------------------------------------------------

--------------------------------------------------------------------------------

--------------------------------------------------------------------------------

--------------------------------------------------------------------------------

----------------------------------------------------------------------------

>TERA01828

ATGGAAGAAGCCTCCAAAGCAGCTGATGAGAGTGAGAGAGCAAGGAAGGTTCTGGAGAACCGCTCAATTAACGACGAAGA

GCGTATTGTCCAATTGGAAAGAGAACTCGAGCAGACCATTTTGCTTGGAGAAGAGGCAGACCGCAATTACGAAGAGGCTG

CTCGTAAGCTGGCCATCACTGAGGTTGATCTAGAGAGGGCTGAGGCTCGTTTGGAAGCTGCCGAGGCTAAGATTCTTGAG

CTGTAA--------------------------------------------------------------------------

--------------------------------------------------------------------------------

--------------------------------------------------------------------------------

--------------------------------------------------------------------------------

--------------------------------------------------------------------------------

--------------------------------------------------------------------------------

--------------------------------------------------------------------------------

--------------------------------------------------------------------------------

--------------------------------------------------------------------------------

--------------------------------------------------------------------------------

--------------------------------------------------------------------------------

--------------------------------------------------------------------------------

--------------------------------------------------------------------------------

----------------------------------------------------------------------------

>TERA01833

ATGAAGATGCCCAAAAAGTTCAAAGGTGAAAACTCCAAAGCAGCTGTTGCCAGAGCACGGAAAGCAGCACAAAAGGAAGA

GGAAGAAAGAAGAAAACAAGAGCAACTTGAAGATGAATACTGGAAAGATGATGATAAACACATCCTACGTAAACAACAGC

GAAAGGAAGAGAGAGAAAAGAAGCGTTTAGAAGTTTTACAGCGTAAGAAGGAAGCAGCTTAA------------------

--------------------------------------------------------------------------------

--------------------------------------------------------------------------------

--------------------------------------------------------------------------------

--------------------------------------------------------------------------------

--------------------------------------------------------------------------------

--------------------------------------------------------------------------------

--------------------------------------------------------------------------------

--------------------------------------------------------------------------------

--------------------------------------------------------------------------------

--------------------------------------------------------------------------------

--------------------------------------------------------------------------------

--------------------------------------------------------------------------------

--------------------------------------------------------------------------------

----------------------------------------------------------------------------

>TERA01841

ATGTGTCTAGAAGGTGTGTGCAAGATCTACGAAGAGTATTTGAAGCGTACAAACCCCAATAGTCCATCAATCACATATGA

CATCAGTCAGCTGTTTGACTTCATTGACCAGATGGCAGACCTGAGTTGTCTAGTATATGAAAAAACAAGTGGCAAATATG

TGCCACACAACAAGGATTGGATAAAAGAAAAAATATACATACTGCTGCGAAAGCAGGCTGGAAAATAA------------

--------------------------------------------------------------------------------

--------------------------------------------------------------------------------

--------------------------------------------------------------------------------

--------------------------------------------------------------------------------

--------------------------------------------------------------------------------

--------------------------------------------------------------------------------

--------------------------------------------------------------------------------

--------------------------------------------------------------------------------

--------------------------------------------------------------------------------

--------------------------------------------------------------------------------

--------------------------------------------------------------------------------

--------------------------------------------------------------------------------

--------------------------------------------------------------------------------

----------------------------------------------------------------------------

>TERA01870

ATGTTCTCTAAGGCGGCACTTCGCTCCGGTGCCCTGAGACCGGTTTTGTCTACACAGCTACAAAACAGGGTGATACAGTG

TCTCAGTGTAAGATGTGCTTCAGTGGGTGAGCATGTTAAGAACTGGGAGAGGGCTAACAAGATCTATTATGGACCAGACA

GGGACACCAAGAATTACCCAGTGAAAAAACTGCCAGAAACATCACCTCCTGTCAGACATGGCTTCATACCTGAATCATTC

TTTACAGCTTTGTATGACAAGACAGGTGTCACAGGCCCCTATGTATTTGGTATTGGTCTGACTACGTACCTGTTGAGCAG

TGAGCTCTGGATTGCTGAGCATGGCATGATTGAGTTCATAGCTTTCTGGTAA----------------------------

--------------------------------------------------------------------------------

--------------------------------------------------------------------------------

--------------------------------------------------------------------------------

--------------------------------------------------------------------------------

--------------------------------------------------------------------------------

--------------------------------------------------------------------------------

--------------------------------------------------------------------------------

--------------------------------------------------------------------------------

--------------------------------------------------------------------------------

--------------------------------------------------------------------------------

--------------------------------------------------------------------------------

----------------------------------------------------------------------------

>TERA01875

ATGGTTGACGAGTGCTGCAAGATGGCCAAGCAGACAGTTGGTGGAAAAGAGGTTGATGTCAAAAAGGCCACGCCAAAAGG

TCAGGATGGAGGCTACGGATTCGGAAGAGGCCGTGGTGGATGGGGAAGTGGACCACAGAGAGGT---AGAGGTTATAATG

ACTGGAACCAAGGTTATGACTACAATAACTATTACAACCAAGGCTATGGTGGTGACTACTATGGTAACTATGGAAACTAC

GGTGGCTATGACTACAACAACTACTACAATCAGGGCTGGGGTGGTTATAACCAAGGTTATGGCAGCGGCTATGGCAGTAG

TGGTTATGGAAATAACAATTATGGAGGTTATGATTACAACGGATGGTATGGGAACGATCAAGGTGGCCAAGGTGGACAGA

CCAGCACATATGGCAAGGCCAAAAGAGGAGCAAGCACAGCTGGTGGCACTACTGGTTATCACCCCTATAACCGATAA---

--------------------------------------------------------------------------------

--------------------------------------------------------------------------------

--------------------------------------------------------------------------------

--------------------------------------------------------------------------------

--------------------------------------------------------------------------------

--------------------------------------------------------------------------------

--------------------------------------------------------------------------------

--------------------------------------------------------------------------------

--------------------------------------------------------------------------------

--------------------------------------------------------------------------------

----------------------------------------------------------------------------

>TERA01878

ATGAAAATGCGTGAAATCGTCCACATACAAGCTGGCCAGTGCGGCAACCAGATTGGTGCTAAGTTCTGGGAAGTGATCTC

AGACGAACATGGTATCGACCCGACGGGCACTTACCATGGTGACTCCGATCTTCAACTAGAGAGAATCAATGTCTATTACA

ATGAAGCGACCGGTGGCAAATACGTTCCCCGCGCTATTCTCGTCGACCTGGAGCCCGGCACGATGGACTCGGTTCGGTCG

GGACCTTTCGGTCAGATCTTCCGTCCGGACAACTTCGTCTTCGGACAGAGCGGAGCCGGTAACAACTGGGCCAAAGGTCA

CTACACGGAGGGCGCCGAGCTGGTCGACTCCGTTCTGGATGTGGTGCGCAAGGAGGCCGAGAGCTGCGACTGTCTGCAGG

GCTTCCAGCTCACCCACTCACTCGGTGGCGGTACCGGATCGGGAATGGGCACGTTGCTCATCAGCAAGATCCGTGAGGAG

TATCCTGATAGGATTATGAATACCTTCTCGGTCGTGCCTTCTCCCAAGGTATCCGACACAGTAGTAGAGCCATACAATGC

CACATTGTCCGTCCATCAGTTGGTAGAGAATACGGACGAGACGTACTGCATTGACAACGAGGCTCTATACGACATCTGCT

TCAGGACACTGAAGCTGACCACTCCGACGTATGGCGATCTGAACCATCTGGTGTCAGCCACCATGTCCGGAGTGACAACA

TGTCTGCGATTCCCTGGTCAACTGAACGCCGATCTGAGGAAGCTGGCCGTCAACATGGTGCCATTCCCTCGTCTTCACTT

CTTCATGCCCGGATTCGCTCCACTCACATCCCGTGGCAGCCAGCAGTACCGAGCCCTGACCGTGCCCGAGCTGACACAGC

AGATGTTTGATGCCAAGAACATGATGGCTGCCTGCGATCCACGTCATGGACGCTACCTGACCGTGGCGGCCATGTTCCGA

GGTCGTATGTCGATGAAGGAAGTGGACGAGCAGATGTTGAACGTGCAGAACAAGAACAGCAGCTACTTTGTGGAATGGAT

CCCGAACAACGTCAAGACGGCCGTCTGTGATATCCCTCCACGTGGTCTGAAGATGTCCTCCACCTTCGTCGGCAACAGCA

CAGCCATCCAGGAGCTGTTCAAGCGTGTGTCCGAGCAGTTCACGGCCATGTTCCGTCGTAAGGCCTTCCTTCACTGGTAC

ACTGGCGAGGGAATGGACGAGATGGAGTTCACTGAGGCCGAGTCAAACATGAATGATCTGGTGTCTGAGTACCAACAGTA

CCAGGATGCTACTGCTGAGGAAGAGGGCGAGTTTGATGAGGAGGAGGAAGAAGAGTAA------------------

>TERA01906

ATGCTCAGTAACTGCGAGCGACTGGACATACTCGTCAACAAAGAGCCGCCGACGAGGAGAGCAAATGCGAAACTCCGAGT

GGATCAGCTGAAATACGACTGTCAGCATTTACAGGCTTCCATGCGTAACATACAGAACAGGAGAATTATGAGAGAACAAG

AAGAATATGAAAGGGAAGCTTTACTTTCACATCACTTTGAACCAAATGATTCATCCACATCAATTATGATTGATGCAGCT

TTACAACACAATCAGCGACTGTCAGATGCACACCATGGCATAGATGATTTGTTATCCAGTGGCACTAGTATTCTTTCTAA

TTTGAAAGAACAGAGGATGACACTTAAAGGTGCTCATAAGAAAATACTTGACATTGCCAACACACTTGGATTGTCAAACA

CTGTGATGAGACTGATAGAAAAGCGTGCAAGCCAGGATAAAATTATTCTTTTTGGTGGAATGATTGTGACTTGTATTATT

ATGTTTCTGGCATGGAAGTACCTTACATAA--------------------------------------------------

--------------------------------------------------------------------------------

--------------------------------------------------------------------------------

--------------------------------------------------------------------------------

--------------------------------------------------------------------------------

--------------------------------------------------------------------------------

--------------------------------------------------------------------------------

--------------------------------------------------------------------------------

--------------------------------------------------------------------------------

--------------------------------------------------------------------------------

----------------------------------------------------------------------------

>TERA01941

ATGGACTGGTCCGAGTACAAAGGGAAGGTGGTGCTGGTGGTGAACGTCGCCTCGTTCTTAGGCTCCACGCACCAGTATCC

AGCTTTGAATGCACTACAAGATTCCTTCAGGGACTTCAAGATACTCGGCGTGCCGTGCAATCAGTTCGGATGGCAAGAGC

CCGGTGATAACGGTACCGAGATCATGAACTCTCTGAAGTACGTACGACCCGGAGACGGCTTCTAA---------------

--------------------------------------------------------------------------------

--------------------------------------------------------------------------------

--------------------------------------------------------------------------------

--------------------------------------------------------------------------------

--------------------------------------------------------------------------------

--------------------------------------------------------------------------------

--------------------------------------------------------------------------------

--------------------------------------------------------------------------------

--------------------------------------------------------------------------------

--------------------------------------------------------------------------------

--------------------------------------------------------------------------------

--------------------------------------------------------------------------------

--------------------------------------------------------------------------------

----------------------------------------------------------------------------

>TERA01961

ATGGATCTCGGTATGGAAGAAGACGAAGAAGAACCAGTTCCTCTTCCAAACGTCAATGCAGCCATCCTAAAGAAAGTCAT

ACAGTGGTGTTCATATCACAAAGATGATCCACCACCACCAGAAGATGATGAGAACAAGGAGAAGAGAACTGATGACATAA

GCTCATGGGATGCTGAATTCTTAAAAGTTGATCAGGGCACATTGTTCGAGCTTATTTTGGCTGCTAATTACCTTGATATA

AAGGGCCTCCTGGATGTGACCTGTAAGACATAA-----------------------------------------------

--------------------------------------------------------------------------------

--------------------------------------------------------------------------------

--------------------------------------------------------------------------------

--------------------------------------------------------------------------------

--------------------------------------------------------------------------------

--------------------------------------------------------------------------------

--------------------------------------------------------------------------------

--------------------------------------------------------------------------------

--------------------------------------------------------------------------------

--------------------------------------------------------------------------------

--------------------------------------------------------------------------------

--------------------------------------------------------------------------------

----------------------------------------------------------------------------

>TERA01977

ATGTCCAACATGGGGCTGAAGGACGAGGCTGTTACTAACAACGATCCAAATGGAGAGGAGGAGGATTTGGTGGACCCAAT

GGACACACTAAAGGAAAAATGTCAGGGTGATGCCCACTGTACCAAATACAAGGATGAACTACAGCGCTGTACTGAACGAG

TACAGAGTAAACAGAACACTACAGAAACCTGTGCCCAGGAACTGTATGATTTTATACATTGTGTAGACCATTGTGTGTCA

AAGGATTTGTTCAAGCATCTGAAATAA-----------------------------------------------------

--------------------------------------------------------------------------------

--------------------------------------------------------------------------------

--------------------------------------------------------------------------------

--------------------------------------------------------------------------------

--------------------------------------------------------------------------------

--------------------------------------------------------------------------------

--------------------------------------------------------------------------------

--------------------------------------------------------------------------------

--------------------------------------------------------------------------------

--------------------------------------------------------------------------------

--------------------------------------------------------------------------------

--------------------------------------------------------------------------------

----------------------------------------------------------------------------

>TERA01978

ATGTCGAAAATGGGTATTGACATAAGTCATAAACATAAGAGGAAGGTGATTAGGCGAAAGCCTAAGAGTCATGATTTATA

CCTTCAGTTGTTGGTAAAGTTGTATCGCTTTCTGGCCAGACGGACAGGATGCAAGTTTAACAGAATAATTCTGAAGAGAC

TTTTCATGAGCAAGACTAACAGGGCGCCTATGTCTGCTTCAACTTTGGCAAGACAGATGAAGAAGCCTGGACGTCAAGAT

AAGATAGCTGTTTGTGTGGGCACAATTACTGATGACTCGCGTGCCTACAAGTTTCCAAAGCTGAAGGTCTGTGCCCTGCG

TGTCACTCGTGCTGCTCGTGCTCGTATCCTGAAGGCTGGTGGTGAATTGATGACCTTTGACCAGTTGGCGCTGAAGTCTC

CCAGAGGAAAGAACACTGTACTTCTACAAGGTCCACGCAAGGCTCGTGAAGCTTACCGCCACTTTGGTCCAGCTCCTGGT

GTACCCCACAGTCATACAAAGCCCTATGTACGCTCCAAGGGACGTAAATTTGAAAGGGCTCGTTAA--------------

--------------------------------------------------------------------------------

--------------------------------------------------------------------------------

--------------------------------------------------------------------------------

--------------------------------------------------------------------------------

--------------------------------------------------------------------------------

--------------------------------------------------------------------------------

--------------------------------------------------------------------------------

--------------------------------------------------------------------------------

--------------------------------------------------------------------------------

----------------------------------------------------------------------------

>TERA01983

ATGCTCAGTTATAACTGGATCAAGACAAGACAGGCTGGTGGCCAGGATAATTTCTTTGGCTTCCTCTTCCTGTGTGGCAT

GGTGACTGTGTCAAGCATGCTCATCTATGGAGCCATTATGGGTCGGCCTGGATATCTGATGCCATTCTTCTGCATCCAGG

TGTTTAACTTCTGTATGACCTGCCTCAGTGGTATTGGCTACTTTTCATACATACCAAACATCAAACAATGGATTTAA---

--------------------------------------------------------------------------------

--------------------------------------------------------------------------------

--------------------------------------------------------------------------------

--------------------------------------------------------------------------------

--------------------------------------------------------------------------------

--------------------------------------------------------------------------------

--------------------------------------------------------------------------------

--------------------------------------------------------------------------------

--------------------------------------------------------------------------------

--------------------------------------------------------------------------------

--------------------------------------------------------------------------------

--------------------------------------------------------------------------------

--------------------------------------------------------------------------------

----------------------------------------------------------------------------

>TERA02018

ATGCTTGTACCTTTCGTACAGATGCGTTTTGCTTCTGCTGCTGCCGCAACTCAAGCTCAGCCAAGAATGAAGAAGTTTAA

GATCTACAGATATGATCCTGACAAACCTGGAAGCAAACCTTACATGCAGGAATATGAAGTGGATCTTAACACGTGTGGTA

CCATGGTTCTTGATGCACTGATAAAAATTAAGAATGAAATTGACTCTACCCTGACGTTCCGCCGCTCATGCCGTGAAGGA

ATTTGTGGCTCATGTGCCATGAACATTAATGGAGCCAACACCTTGGCCTGTCTGTGCCCCATCGACACCAATACAAGCAA

GACAAGCAAGATATATCCACTGCCTCATATGTATGTTGTGAAGGACTTGGTGCCGGACATGAACAACTTCTATGCCCAGT

ACAAGTCCATTGAACCATACCTGAAGAAGAAGGACATGGGAGAGGATGATATTGGGAAAACCTCACTTAAACAATCGCCA

AAGGACCGTGAGAAGCTGGATGGCATGTATGAGTGCATCCTGTGTGCTTGTTGCAGTACTTCTTGCCCAAGCTACTGGTG

GAATGGCGACAAGTACCTGGGTCCAGCTGTTCTGATGCAGGCTTACAGGTGGGTGATGGATTCACGTGATGACTTCAAAC

AGGAGCGTCTGGAGCAGCTCAGAGATGAGTGGTCTCTGTACAGATGTCACACCATCCAGAACTGTACAAAGACCTGCCCA

AAGGGATTGAACCCTTCTGCTGCCATTGCTGAAATGAAGCGTTAA-----------------------------------

--------------------------------------------------------------------------------

--------------------------------------------------------------------------------

--------------------------------------------------------------------------------

--------------------------------------------------------------------------------

--------------------------------------------------------------------------------

--------------------------------------------------------------------------------

----------------------------------------------------------------------------

>TERA02023

ATGTCAGAAAACCGTTCTCGCGTTTGGGATCCTACTCAATTATTTGACATCTCCCGCGAGGAGAGACGACGAATTGAGGA

AAGAGCAAAGATGAGATCTCAGCTGAGAGCGGAGTTTCAAAGAAAAGCAACCAACCCGTATAGTGGTGTTGGAGGTTACC

TTTTTGACCCTGCTGTGCAGAGGTTCTTGTCAATGAGAGCCAACCATTTTGAACACTTTAAAGCCACTCCAAAGACTGCC

CTTTTTGGCTTTGGCTGGGTCCTTGGGCCATTTTTACTTCTGTGGTAA--------------------------------

--------------------------------------------------------------------------------

--------------------------------------------------------------------------------

--------------------------------------------------------------------------------

--------------------------------------------------------------------------------

--------------------------------------------------------------------------------

--------------------------------------------------------------------------------

--------------------------------------------------------------------------------

--------------------------------------------------------------------------------

--------------------------------------------------------------------------------

--------------------------------------------------------------------------------

--------------------------------------------------------------------------------

--------------------------------------------------------------------------------

----------------------------------------------------------------------------

>TERA02036

ATGGAGACGCTGAACAAGGCTTTGCCGAAGCACGGATTTCTCTACCCTACTGCGACGGTTTCTCTGTCAGGCAAATCTCA

GATTCGTTTGGCTCATACTGATGTCAAGGTGCCCGACTTTTCGGCTTACAGACGTGACAGTACTCAGAGCAGCACAGCCA

AGTCATCTGAGACTGCTGATGCTAGGAGAACCTTTACCTACATGATGTTGGCAGCTGGTACTGTACCCACACTGTATGCT

GGCAAAGCCTTGGCTGCTGAATTTGTACTTTCAATGAGTGCCACGGCCGACGTGTTGGCTCTGGCAAAGGTCGAGGTCAA

CCTAGCTGACATCCCTGAGGGCAAGAACGCAGTATTCAAATGGCGAGGGAAGCCACTGTTTTAA----------------

--------------------------------------------------------------------------------

--------------------------------------------------------------------------------

--------------------------------------------------------------------------------

--------------------------------------------------------------------------------

--------------------------------------------------------------------------------

--------------------------------------------------------------------------------

--------------------------------------------------------------------------------

--------------------------------------------------------------------------------

--------------------------------------------------------------------------------

--------------------------------------------------------------------------------

--------------------------------------------------------------------------------

----------------------------------------------------------------------------

>TERA02062

ATGCCATCAAGAAGGAAGGGTACTTTGACCAAACATAACAAGTTCATACGTGATCTAATCAGAGAAATTGCTGGACAAGC

ACCCTATGAGAAGAGAACAGTGGAGTTGCTGAGAATTTCAAAAGATAAACGTGCATTGAAGTTCTGCAAGAAAAGGTTGG

GATCACATCTTCGTGCCAAGAGGAAGCGTGAAGAGATGGCAAACATCATCCAACAGATGAGGAAAGCACAAGCTCACAAG

TAA-----------------------------------------------------------------------------

--------------------------------------------------------------------------------

--------------------------------------------------------------------------------

--------------------------------------------------------------------------------

--------------------------------------------------------------------------------

--------------------------------------------------------------------------------

--------------------------------------------------------------------------------

--------------------------------------------------------------------------------

--------------------------------------------------------------------------------

--------------------------------------------------------------------------------

--------------------------------------------------------------------------------

--------------------------------------------------------------------------------

--------------------------------------------------------------------------------

----------------------------------------------------------------------------

>TERA02083

ATGGCGTTAAGGTTGTCGAGTGTGTTCTCAGCTTGGAGGAACGTTTGTTCTGTCTCGGTTCGTGCTATGTCACAGACTGG

AGAATGGGGTGAGGGAGCCGGAAAGGGTGGTGGTGCTGGTGGTTCTGTAAGAGACGCTGGTGGCACCTTTGGCAAGAAGC

AGGCAGCCCAGGAAGAACAGTACTTCAAGAGACTGCAAGCGGAACAGCTGAAGAAGTTGAGGGATTCGGTCCATGATGAG

ATCAAGTACCACAAGGAACAGATCGAGGAGCATGAGGAGGCCATTGAGAGACACGAGAGGAAACTGTAA-----------

--------------------------------------------------------------------------------

--------------------------------------------------------------------------------

--------------------------------------------------------------------------------

--------------------------------------------------------------------------------

--------------------------------------------------------------------------------

--------------------------------------------------------------------------------

--------------------------------------------------------------------------------

--------------------------------------------------------------------------------

--------------------------------------------------------------------------------

--------------------------------------------------------------------------------

--------------------------------------------------------------------------------

--------------------------------------------------------------------------------

----------------------------------------------------------------------------

>TERA02086

ATGGATTCCATTGGTAAAGTGGAAATAAAAGATCAGGATGAGCTAGTACAGAGATGCATTATGCAGGCAAATGTGTGGGA

CCCAACTAAGATTAAGCTACCACGGAGAATCAACAAATCCCTTCCTGGCTGGAAATTCAAAGCAGAATATGGCATACCTC

AACAGAAGATGAGGTCTTTATTGATGCAGAATTTGATACGCTAA------------------------------------

--------------------------------------------------------------------------------

--------------------------------------------------------------------------------

--------------------------------------------------------------------------------

--------------------------------------------------------------------------------

--------------------------------------------------------------------------------

--------------------------------------------------------------------------------

--------------------------------------------------------------------------------

--------------------------------------------------------------------------------

--------------------------------------------------------------------------------

--------------------------------------------------------------------------------

--------------------------------------------------------------------------------

--------------------------------------------------------------------------------

--------------------------------------------------------------------------------

----------------------------------------------------------------------------

>TERA02141

ATGTACTTCAAGCAGTTTCCCCAACACCAGAACTTCTTCGAAGCCTACAAGGGCAAGGATCCCGACAGCCTGAATTCCGT

GGCCAAATTCAAGCCCCACGTCACTAAGGTCGTCAGCACGCTGCTGGACATCCTGGAGAAGGCTGACGACGCCGGCGCTC

TCCAGAAGAACTGCGACATGCTGGCCAAGATGTCACAGCACAAGGGACTGAAGGCCACCGAGTTCAAGGATCTCGGCACG

GTCGTGGTCGCCTACGTGAAGCAGACCCTTGGCGGTTCCTGTGACGCTGCCGGTTGGGAGTCGGCCTTCAATGCCCTTAC

CTCCAAGCTGGGACCAATGCTTTAA-------------------------------------------------------

--------------------------------------------------------------------------------

--------------------------------------------------------------------------------

--------------------------------------------------------------------------------

--------------------------------------------------------------------------------

--------------------------------------------------------------------------------

--------------------------------------------------------------------------------

--------------------------------------------------------------------------------

--------------------------------------------------------------------------------

--------------------------------------------------------------------------------

--------------------------------------------------------------------------------

--------------------------------------------------------------------------------

----------------------------------------------------------------------------

>TERA02169

ATGTCCAAGATGGGACAAGATTATTACAGTATTCTTTGCCTAACGCGAAGCGCAACAGATGCAGACATAAAGAAAGCTTA

CAGAAAGCTAGCCTTAAAATATCACCCAGAAAAGAATCCAAATGACCAAGTCGCGGCTGAAAAGTTCAAACAAATTGCCG

AAGCATATGATGTTCTCAGTGATGCTCGCAAAAGGGCTGTTTACGACCAATTTGGCGAGGAGGGTTTGAAGAGTGGTGTT

CCACACGGAACAGCTGAATCTGGAGCTTGGACTCAGGGATACACATTCCATGGAAATGCTGACAAAGTGTTCCGTGACTT

CTTTGGTGGAGACAATCCATTTCAGGAGTTCTATGATCGAGTTGATGGAGACATGCACATGGGTTTTGGTGGATTGCATG

GAAGAGGAGCAAAGAAACAGGACCCACCAATAGAAAGAGAACTACAGCTTTCACTGGAAGAAGTCTACCATGGGTGCACA

AAGAAGATGAAAATCTCAAGAAGAGTTATGAATGAAGATGGTCATACATCAAGTATTCGAGACAAGATACTTACCATCTC

AGTAAAGAAAGGTTGGAAGCCAGGAACAAGGATTACATTTCCACAAGAAGGTGACCAAGGACCGAATAATTAA-------

--------------------------------------------------------------------------------

--------------------------------------------------------------------------------

--------------------------------------------------------------------------------

--------------------------------------------------------------------------------

--------------------------------------------------------------------------------

--------------------------------------------------------------------------------

--------------------------------------------------------------------------------

--------------------------------------------------------------------------------

----------------------------------------------------------------------------

>TERA02175

ATGATTTCGGATGCGGAGAAGGTAAGAATAGTCTCTGACTTCATCATTCATGCCCCACCAGGAGAATTTAATGAAGTGTT

TAATGATGTCCGCATTCTACTGAATGATGATAACTTGCTGAAGGAACAGGTTTCTGGAGCCTTTGCTCAATATAACAAAG

ATCAGTTTACACCAGTGAAAGTAAATGGCTCTGATGAACAGGCACTGATAACTCCACATGCAGATAAAGGAAATGGACGA

TTTCTAGATCCTCGTACAAAACAATCCTTCAGATATGACCATTTAAGGAAGGAAGGTAGTGACTTTAGACCATGTAATGT

AGATCAAACAGCTGAACCCTGGCGAGCAGCACTTGAGTCAACATTTACTGAATATGTCCATGAACATTATAAACAAGGTG

TCTGTAGTGTGTATGGAGCAAGTGATGCTGGCATTATTACCTTGGTAGCTTATATTGAAAGCCACCAATTCAATCCTAAG

AACTACTGGAATGGACGTTGGCGGTCACAGTGGACTGTTGTTTTCAACAAGTCAGGTGGTACAGCTGAAGTGCAGGGAAT

ACTTAAAGTGCAGGTTCATTACTAA-------------------------------------------------------

--------------------------------------------------------------------------------

--------------------------------------------------------------------------------

--------------------------------------------------------------------------------

--------------------------------------------------------------------------------

--------------------------------------------------------------------------------

--------------------------------------------------------------------------------

--------------------------------------------------------------------------------

--------------------------------------------------------------------------------

----------------------------------------------------------------------------

>TERA02230

ATGGTTGCATTACAAACAGCTGTATTTGCCAGAGACTTGGAACTACATCCACCATCCTACCCATGGAGCTACAAGGGCAT

CTTTGATTCATATGACCATGCCAGTGTCCGTCGAGGCTATCAGGTATATAAACAGGTTTGTGCAGCTTGTCACTCCATGG

ACTTCATGTATTATAGAAACCTTGTTGGCACCATTATGACAGAAGATGAAGCCAAGGCAGAAGCTGCAGAGATCCAAGTT

GTTGATGGACCAGATGATGAGGGTAACATGTTCAAGCGCCCAGGCAAATTGTCTGACCGCTTTCCACAGCCATATGCCAA

TGAAGAGGCTGCCAAGGCTGCTAACAGTGGAGCATACCCTCCAGATTTGACATTTATTGTCAATGCAAGGGAAGGTGGAG

CAGATTATATTTTCTCACTGTTGACTGGATACTGCGACCCACCTGCCGGATGTGAAGAACGAGAAGGTCTACACTACTAA

--------------------------------------------------------------------------------

--------------------------------------------------------------------------------

--------------------------------------------------------------------------------

--------------------------------------------------------------------------------

--------------------------------------------------------------------------------

--------------------------------------------------------------------------------

--------------------------------------------------------------------------------

--------------------------------------------------------------------------------

--------------------------------------------------------------------------------

--------------------------------------------------------------------------------

----------------------------------------------------------------------------

>TERA02231

ATGTTCACTGATGCTGTAGAGATGAAACAGGTTGCCCAACAAGAAGCAGAGAAAGCTCGTTATGTTGTAGAAAAGGAAGA

GCAGAGAAAGAAGGCTGCCATCATCCGTGCTGAGGGCGACTCCCAAGCTGCAATCTTGCTAGCAAAGGCTCTGGGTGATG

CCGGAGAGGGTCTGGTCGAGTTGAGAAAAATTGAAGCATCCGAAGATATTGCATACCAAATGTCGCAGTCACCAAATGTC

ATCTAA--------------------------------------------------------------------------

--------------------------------------------------------------------------------

--------------------------------------------------------------------------------

--------------------------------------------------------------------------------

--------------------------------------------------------------------------------

--------------------------------------------------------------------------------

--------------------------------------------------------------------------------

--------------------------------------------------------------------------------

--------------------------------------------------------------------------------

--------------------------------------------------------------------------------

--------------------------------------------------------------------------------

--------------------------------------------------------------------------------

--------------------------------------------------------------------------------

----------------------------------------------------------------------------

>TERA02240

ATGGAAAATATGGCATCTGATGCTGAGCTGGCTTGCGTTTATTCGGCTTTAATTCTTGCCGATGACGATGTACCAATCAC

TGCTGACAAGTTGAACACGATCTTGAAGGCGGCCAAAGTTGAAGTGGAACCATACTGGCCAACACTCTTTGCCAGAGCCC

TTAGTGGCATGAATGTCAAGGATTAA------------------------------------------------------

--------------------------------------------------------------------------------

--------------------------------------------------------------------------------

--------------------------------------------------------------------------------

--------------------------------------------------------------------------------

--------------------------------------------------------------------------------

--------------------------------------------------------------------------------

--------------------------------------------------------------------------------

--------------------------------------------------------------------------------

--------------------------------------------------------------------------------

--------------------------------------------------------------------------------

--------------------------------------------------------------------------------

--------------------------------------------------------------------------------

--------------------------------------------------------------------------------

----------------------------------------------------------------------------

>TERA02261

ATGACGGACCTTAGCCCCAGCGAGATTGAAGACGTACATGAGGTTTTCGACCTCTTTGACTTCTGGGATGGTCGCGACGG

TTTGGTTGATGGATACAAAATTGGTGACTTACTGAGATGTACTGGGCTCAACCCGACCACACAAATAATTTTAAAAAATG

GCGGGACTACCAAAGAGGGTGAGAAGCAGTACAAATTTGAAGAAATCCTTCCCATCTACAAAGCCGTCAGTGCCGAGACC

GACACAGGAACGTTCGCCGACTTCATGGAGGCCTTCAAGACCTTTGACAGAGAAGGACAAGGTTTCGTAGCTGCTGCCGA

AACTAGACACGTCTTACAATCCTTGGGTGATCGTTTAACGGACGAGGAAGTGGAAAACATTTTCAAATTCACTGGAACAG

AGGAAGATTTGGATGGAAACATTAAATATGAGGAGTTCATCAAGAAAGTTATGGCCGGCCCTGGAAACAAATAA------

--------------------------------------------------------------------------------

--------------------------------------------------------------------------------

--------------------------------------------------------------------------------

--------------------------------------------------------------------------------

--------------------------------------------------------------------------------

--------------------------------------------------------------------------------

--------------------------------------------------------------------------------

--------------------------------------------------------------------------------

--------------------------------------------------------------------------------

--------------------------------------------------------------------------------

----------------------------------------------------------------------------

>TERA02289

ATGCAAATGGCCGAGATGACGCAGTGGGTAACTGTGGGTCTGGTCATGTGCCTGGCCTTTCTTACCTGGAGAGATATCAC

TTCTCCAATCGACGCCCAGAAGACGACCAAGGACATTCCAATGCCGAAACTGTCAAAGTTTACCGGTCCGACACTCAGAT

TTCTCTTCTGTTATTCCTAA------------------------------------------------------------

--------------------------------------------------------------------------------

--------------------------------------------------------------------------------

--------------------------------------------------------------------------------

--------------------------------------------------------------------------------

--------------------------------------------------------------------------------

--------------------------------------------------------------------------------

--------------------------------------------------------------------------------

--------------------------------------------------------------------------------

--------------------------------------------------------------------------------

--------------------------------------------------------------------------------

--------------------------------------------------------------------------------

--------------------------------------------------------------------------------

--------------------------------------------------------------------------------

----------------------------------------------------------------------------

>TERA02352

ATGAAAATGCGTGAATGTATCAGTATCCACGTGGGCCAGGCTGGTGTCCAGATGGGCAATGCCTGCTGGGAGCTGTACTG

TCTGGAGCACGGCATACAGCCTGATGGCCAGATGCCAAGCGACAAGACGATCGGAGGCGGTGACGACTCGTTTAACACGT

TCTTCAGTGAGACCGGAGCCGGTAAACACGTACCGAGGGCCGTCTTTGTTGATCTGGAGCCGACCGTCGTCGATGAGGTC

CGCACTGGTACCTATCGCCAGCTATTCCATCCTGAACAGCTCATCACTGGCAAGGAAGATGCTGCCAACAACTATGCCCG

TGGCCATTACACCATTGGCAAAGAATTGATTGATCTTGTTCTGGATCGTACCAGGAAACTGGCTGATCAGTGCACTGGTC

TGCAGGGTTTTCTCATCTTCCACAGTTTTGGTGGTGGTACTGGATCTGGCTTCACCTCTCTGCTCATGGAGAGGCTCAGT

GTTGACTATGGCAAGAAATCCAAGCTGGAGTTCTCTGTCTACCCAGCTCCGCAGATCTCTACTGCTGTGGTTGAGCCATA

CAATTCCATCCTGACCACCCACACCACCCTGGAGCACTCCGACTGTGCTTTCATGGTTGACAACGAGGCCATCTATGACA

TCTGTCGTCGTAATCTGGACATTGAGCGTCCAACCTACACCAACCTGAATCGTCTTATTGGCCAGATTGTCAGCTCCATC

ACTGCCTCTCTGCGCTTTGATGGAGCTCTGAATGTCGATCTGACAGAGTTCCAAACCAACCTGGTTCCCTATCCACGTAT

CCACTTCCCTCTGGCCACCTATGCCCCAGTCATCTCGGCCGAGAAGGCCTACCATGAGCAGCTGACCGTGGCTGAGATCA

CCAATGCCTGCTTCGAGCCAGCCAACCAGATGGTGAAATGTGACCCACGTCATGGCAAGTACATGGCCTGCTGCATGCTG

TACCGTGGAGATGTTGTACCAAAGGATGTCAATGCAGCCATCGCCACCATCAAGACAAAGCGTACCATCCAGTTCGTGGA

TTGGTGTCCAACTGGCTTCAAGGTTGGCATCAACTACCAGCCACCAACCGTTGTACCAGGAGGTGACTTGGCCAAGGTGC

AGCGTGCCGTGTGCATGTTGAGTAACACAACAGCCATCGCTGAGGCCTGGGCTCGTCTGGATCACAAGTTTGATCTGATG

TATGCCAAGCGTGCCTTTGTCCATTGGTACGTCGGTGAGGGTATGGAAGAAGGAGAGTTCTCTGAAGCCCGTGAGGATCT

GGCTGCCCTAGAAAAAGATTACGAAGAGGTTGGTGTCGACTCTGTGGAGGGTGAAGGTGAAGAAGAAGGAGAATAA

>TERA02364

ATGCCTTTTCCGGTTAGCATGGGGTTTCAGAGCAAGCCTATCATTATTGATGCCCGCGGGCATCTGTTGGGCCGTCTCGC

CGCAGTTGTCGCTAAAAATATCCTTCAAGGTCAGAGGATAGTGGTAGTACGATGCGAAGGTATCAACATTTCAGGAAGCT

TCTACAGGAACAAGTTAAAATACTTGGCTTTCTTGCGGAAGCGAATGAACACTAATCCGAGCCGTGGTCCTTTCCACTTC

CGAGCTCCAAGCAAGATATTCTGGAGAACTGTGCGTGGCATGTTGCCACACAAGCTTACTCGTGGAAAGGTGGCTTTGTC

CCACCTGAAGGTGTTTGAGGGAATTCCACCTCCATATGACAAGAAGAAACGCATGGTAGTGCCCTCAGCTCTGAGAGTCC

TTAGACTGAAGGCTAGACGCAAGTACTGTGTTCTTGGACGCCTTTCCCATGAGGTTGGTTGGAAGTACCAGAATGTGATT

GAGACTCTAGAAAGCAAAAGGAAGGTGAAGTCCTCCCAGTACTATAAGAAGAAGAAATTGGAGAATAAACTCCTTGAACA

GGCCAGGAAGGATGTTGCCAAGAGGATAGAGCCATACCAGCAAATCATCGAGAGTTATGGATACCGATAA----------

--------------------------------------------------------------------------------

--------------------------------------------------------------------------------

--------------------------------------------------------------------------------

--------------------------------------------------------------------------------

--------------------------------------------------------------------------------

--------------------------------------------------------------------------------

--------------------------------------------------------------------------------

--------------------------------------------------------------------------------

----------------------------------------------------------------------------

>TERA02369

ATGTTCCTGTTGGTGACGGTGAAGATGACCACGAAGAAGAAGAAGACCAGGAAGCTACGTGGCCACGTCAGTCATGGAAA

GGGCCGTGTTGGCAAACACAGGAAACATCCAGGTGGACGTGGTAATGCTGGTGGCATGCATCATCACAGAATAAATTTTG

ACAAATACCATCCTGGTTACTTTGGTAAATTGGGTATGCGTTACTACCACAAGACCAAACAGAAGTTTTACTGCCCAACA

ATAAATGTTGACAAACTGTGGTCACTAGTACCTGACCGGACACGTGAAGTCTATGCCAAGAAGACTGATGTAGCACCTGT

TATTGATTGCTTGCGTGCTGGTTATTACAAAGTACTGGGCAAGGGTCACCTACCAAGGCAGCCACTCATCGTTAAGGCAA

AGTTCTTCAGTTAA------------------------------------------------------------------

--------------------------------------------------------------------------------

--------------------------------------------------------------------------------

--------------------------------------------------------------------------------

--------------------------------------------------------------------------------

--------------------------------------------------------------------------------

--------------------------------------------------------------------------------

--------------------------------------------------------------------------------

--------------------------------------------------------------------------------

--------------------------------------------------------------------------------

--------------------------------------------------------------------------------

----------------------------------------------------------------------------

>TERA02379

ATGAAGATCGAGTTGTGTGTATTTAGCGGGTATAAAATATACCCTGGGCATGGGAAGAAGCTTGTCAAAGTTGACGGCCG

GGGGTTCCAGTTTGTCAATGGAAAATGTGAGAAGTCCTATTTAATGAAACGTAACCCCCGTAAGATCAACTGGACTGTGC

TATATCGACGCAAGCACAAGAAGGGGCAGACGGAGGAAGTCTCAAAGAAGCGAACCCGTCGTACAACCAAGTTCCAGAGA

GCCATCACTGGTGCCACCTTGACTGATATCTTGGCAAAAAGGAACCAGAAGCCTGAGGTTCGCAAGGCACAGCGTGAGCA

AGCTATCAGAGCTGCTAAGGAGAAGCAGAAGGCAAAAGAAGCTGCCAAGATTGCACAGAGGGTTGCCCAAGCCAAGGCTG

CTTCAAAGCACAAACCTTCCAAACCTGTTATGGGAAGCAAGCCACGTATGGGATAA------------------------

--------------------------------------------------------------------------------

--------------------------------------------------------------------------------

--------------------------------------------------------------------------------

--------------------------------------------------------------------------------

--------------------------------------------------------------------------------

--------------------------------------------------------------------------------

--------------------------------------------------------------------------------

--------------------------------------------------------------------------------

--------------------------------------------------------------------------------

--------------------------------------------------------------------------------

----------------------------------------------------------------------------

>TERA02381

ATGAATGGTTGTCCACTGAGACGTATCAATCAGATCTACGTAATTGCCACAAAAACAAAGATTGACATCAGTGGAATCAA

CATACCTGAAAGGCTGACTGATGATTATTTCCGCCGTAAGAAGCCAAGGAAGCCCAAACAGAATGAGGGCGAGATATTTG

ACACAAAGAAAGAGGAGTAA------------------------------------------------------------

--------------------------------------------------------------------------------

--------------------------------------------------------------------------------

--------------------------------------------------------------------------------

--------------------------------------------------------------------------------

--------------------------------------------------------------------------------

--------------------------------------------------------------------------------

--------------------------------------------------------------------------------

--------------------------------------------------------------------------------

--------------------------------------------------------------------------------

--------------------------------------------------------------------------------

--------------------------------------------------------------------------------

--------------------------------------------------------------------------------

--------------------------------------------------------------------------------

----------------------------------------------------------------------------

>TERA02384

ATGTTTAGGACATTCTTCAAAATTATCAAAGAAAATGGAGGTATAGTTGGGTCCTTCCTGAAGCTCTACAGGATGGAAGA

GCTGAAGGATGGAATACTGGTTGGACAGGACAAATATGGCCACAAGTATTATGAGAACAAGCGTTATTTTATAGGTCGTA

GCAGGTGGGTTGATTATGCTGATGATGTAGGAATGGACTATGATGGAAGTCAGATTCCAGCTGAATGGCATCGCTGGCTG

CACTACATCACTGATGATCCACCAACAAAAGTGCCACCAGTGTCACGCAAGTACATCCTCGATCATAAAGAAAACCCAAC

AGGCACACATGAGCAGTATGTACCTTACAGTTAA----------------------------------------------

--------------------------------------------------------------------------------

--------------------------------------------------------------------------------

--------------------------------------------------------------------------------

--------------------------------------------------------------------------------

--------------------------------------------------------------------------------

--------------------------------------------------------------------------------

--------------------------------------------------------------------------------

--------------------------------------------------------------------------------

--------------------------------------------------------------------------------

--------------------------------------------------------------------------------

--------------------------------------------------------------------------------

----------------------------------------------------------------------------

>TERA02389

ATGCAGAAAATGACAGACAAATGTTACAGGAAATGCATCCATAAACCAGGCACGTCATTAGATAATTCTGAACAGAAATG

TATTGCCATGTGCATGGATCGGTATATGGATGCATGGAATCTGGTATCAAGAACATACACACAAAGACTTCAGAGAGAGA

GAAGCAGGATTGGTTAA---------------------------------------------------------------

--------------------------------------------------------------------------------

--------------------------------------------------------------------------------

--------------------------------------------------------------------------------

--------------------------------------------------------------------------------

--------------------------------------------------------------------------------

--------------------------------------------------------------------------------

--------------------------------------------------------------------------------

--------------------------------------------------------------------------------

--------------------------------------------------------------------------------

--------------------------------------------------------------------------------

--------------------------------------------------------------------------------

--------------------------------------------------------------------------------

--------------------------------------------------------------------------------

----------------------------------------------------------------------------

>TERA02392

ATGACGAATACAAAGGGTTACCGCCGTGGTACCAGGGATCTCTTTTCCCGACCCTTCCGTAGGAAGGGAGTCATCCCACT

GTCTACCTATATGAAAGTGTACAAAGTGGGCGACATAGTGGATATTAAGGGTAATGGAGCTGTCCAAAAAGGCATGCCTC

ACAAATACTACCATGGCAAGACTGGTCGTGTGTACAATGTTACCCAGCATGCTGTTGGTGTGATAGTTAACAAACGAGTT

GGAGGAAGGATCATTGCCAAGAGGATCAACTTGCGCATTGAACATGTCAAGCACTAA-----------------------

--------------------------------------------------------------------------------

--------------------------------------------------------------------------------

--------------------------------------------------------------------------------

--------------------------------------------------------------------------------

--------------------------------------------------------------------------------

--------------------------------------------------------------------------------

--------------------------------------------------------------------------------

--------------------------------------------------------------------------------

--------------------------------------------------------------------------------

--------------------------------------------------------------------------------

--------------------------------------------------------------------------------

--------------------------------------------------------------------------------

----------------------------------------------------------------------------

>TERA02396

ATGAAGATGAAGTTGAACATATCTTACCCAGCCACGGGCTGTCAGAAGCTCATCGAAGTGGACGATGAACACAAACTGCG

ACATTTTTACGAGAAGCGTATGGCAGCCGAGTTGCCGGCAGACCGACTTGGAGACGAATGGAAGGGATACATCGTACGTA

TTTCCGGTGGCAACGACAAACAAGGCTTTCCAATGAAGCAAGGCGTGCTGACGTCGGGTCGTGTCAAGCTGCTGCTGAGT

AAAGGCCACTCATGCTTCAGACCGAGACGTAGCGGTGAGAGGAGGAGGAAATCTGTCCGTGGATGTATAGTGGACGCCAA

CCTCAGTGTGCTTAACCTGGTCATCGTCAGGAAAGGTGTTAATGATATTCCTGGTCTTACTGACAAGACTATCCCCCGTC

GTCTTGGACCCAAAAGGGCCAGCAAAATCCGCAAGCTGTTCAACCTGTCAAAGGAAGATGACGTCAGACAATATGTCGTT

AGGCGCCCACTCCCTGAAAGAGAAGGCAAGAAGCCAAAGACAAAGGCCCCAAAGATCCAACGTCTTGTTACACCTTTGGT

CTTACAGCGCAAACGCCACCGTCTGGCACTGAAGAAGAGACGAGCTGCCAAGCGTGCAGAGGAGGCCGCTGAGTATACAA

AACTGCTTGCTCAGAGAGTGAAGGAGGCCAAGGAACGTAAGGCAGAGCGTCGCAGGACAGCTTAA---------------

--------------------------------------------------------------------------------

--------------------------------------------------------------------------------

--------------------------------------------------------------------------------

--------------------------------------------------------------------------------

--------------------------------------------------------------------------------

--------------------------------------------------------------------------------

--------------------------------------------------------------------------------

----------------------------------------------------------------------------

>TERA02397

ATGGCAATCATGTCGCTGATAATTCCCGAGAAGTTTCAGCACATTTTGCGTGTGCTCAACACGAACATTGACGGTAAAAG

AAAGGTGCCATTTGCCCTTACAGCTATCAAGGGTGTTGGACGTAGATATTCCCACATTATTTTGAAGAAGGCAGATGTTG

ATCCAAGTAAGCGTGCTGGAGAGTTGGACGAGGATGAGGTTGAGAAAGTGGTGACAATTATGTCCAATCCACGCCAGTAC

AAGATTCCGGATTGGTTCCTGAACAGACAGAAAGATATCAAGGATGGAAAATACAGTCAGGTCTTGTCCAATGTGCTTGA

CAGCAAGATCCGTGAAGATTTGGAACGACTAAAGAAGATCCGTGCCCACAGAGGTCTGCGACACTACTGGGGTCTGCGTG

TCCGTGGTCAGCACACGAAGACAACGGGTCGCCGTGGAAGGACTGTTGGTGTGTCCAAGAAGAAGTAA------------

--------------------------------------------------------------------------------

--------------------------------------------------------------------------------

--------------------------------------------------------------------------------

--------------------------------------------------------------------------------

--------------------------------------------------------------------------------

--------------------------------------------------------------------------------

--------------------------------------------------------------------------------

--------------------------------------------------------------------------------

--------------------------------------------------------------------------------

--------------------------------------------------------------------------------

----------------------------------------------------------------------------

>TERA02404

ATGCAGAACGATTCTGGTGAATGTGTGGATATGTACATCCCCAGAAAATGCTCAGCCAGCACAAGAATTATTGGAGCTAA

AGATCATGCTTCTATCCAGATAAACTTGGCAGAGGTTGATGAGACAACAGGTCGTATGACAGGCCAATACAAGACATATG

CCATCTGTGGGCAGATAAGGAGAATGGGTGAATCTGATGACTGCATTACCAGATTGGCAAAGAAAGATGGACTTGTTGCT

AAGAACTAA-----------------------------------------------------------------------

--------------------------------------------------------------------------------

--------------------------------------------------------------------------------

--------------------------------------------------------------------------------

--------------------------------------------------------------------------------

--------------------------------------------------------------------------------

--------------------------------------------------------------------------------

--------------------------------------------------------------------------------

--------------------------------------------------------------------------------

--------------------------------------------------------------------------------

--------------------------------------------------------------------------------

--------------------------------------------------------------------------------

--------------------------------------------------------------------------------

----------------------------------------------------------------------------

>TERA02409

ATGGCTGCCACCGAAGACTGGGATGAGCAGATAGCGGCTGTAGCTGAGATTCCAGAAATCAAACTTTTTGGTAAATGGTC

TTCAGACGATGTCCAAGTCAGTGACATCAGCTTAACAGATTACATTGCTGTCAAGGAGAAGTATGCCAAATATGTACCAC

ACTCTGCTGGTAGATACCAAGTTAAGAGATTCCGCAAGAGTCAATGTCCAATAGTCGAGCGCCTTGCTTGCTCACTGATG

ATGCATGGACGTAACAATGGCAAGAAGCTGATGGCCATACGTATCGTCAAGCATGCTTTCGAGATCATACATCTGCTGAC

TGGAGAGAATCCCCTACAAGTTGTGGTTAATGCCATCATCAACAGTGGTCCCCGTGAGGACTCGACTCGTATAGGACGAG

CTGGTACGGTACGTCGTCAGGCCGTGGACGTGTCCCCACTGAGGCGTGTAAACCAAGCCATCTGGCTTCTGTGCACTGGA

GCTAGGGAAGCAGCCTTCAGGAACATCAAGACAATAGCTGAGTGTTTGGCTGACGAACTGATCAATGCTGCCAAGGGCTC

GTCCAACTCGTATGCCATCAAGAAAAAGGATGAACTGGAGCGAGTAGCCAAGTCCAACAGATAA----------------

--------------------------------------------------------------------------------

--------------------------------------------------------------------------------

--------------------------------------------------------------------------------

--------------------------------------------------------------------------------

--------------------------------------------------------------------------------

--------------------------------------------------------------------------------

--------------------------------------------------------------------------------

--------------------------------------------------------------------------------

----------------------------------------------------------------------------

>TERA02428

ATGGGTGCCTACAAGTATATGCAGGAGCTCTACCGCAAGAAGCAGAGCGACACGATGCGTTTCCTGCTTCGAATCCGTTG

TTGGCAGTACCGTCAGCTGTCAAAGATACACAGAGCACCACGACCTTCACGCCCAGACAAGGCCAGAAGACTTGGATACA

AGGCCAAACAAGGGTTTGTCATCTATCGTGTCCGTATCCGTCGTGGAGGACGCAAGAGGCCAGTACCCAAGGGTCAGGTC

TATGGTAAGCCATCCAACTGTGGCATCAACCAACTGAAGAACCAGAGGTCACTTCAGGCCATTGCTGAGGAACGTGTTGG

TCGTAAATGTAAGGCCCTGCGTGTGTTGAACAGCTACTGGGTTGCCCAGGACTCGACATACAAGTTCTTTGAGGTGATCG

CGATTGATCCGTTCCACAAAGCCATCCGACGCGATCCAACACTGCAGTGGATCTGTAAGCCAACTGCCAAGCACCGTGAG

ATGCGTGGTCTGACATCGGCAAACAGGAAGTCTCGTGGACTGGGCAAGGGACATCGCTTCACGAAGACTATTGGTGGTTC

TCGTCGTGCTGCCTGGAAGAGAAACAACACGCTGCAGGCTCACAGACGACGATAA-------------------------

--------------------------------------------------------------------------------

--------------------------------------------------------------------------------

--------------------------------------------------------------------------------

--------------------------------------------------------------------------------

--------------------------------------------------------------------------------

--------------------------------------------------------------------------------

--------------------------------------------------------------------------------

--------------------------------------------------------------------------------

----------------------------------------------------------------------------

>TERA02433

ATGGTTTTAATTGAGACTGTCAGGAACCTGGTTCGGCCAATCACCCTATCTTTTCGATTAGCCGCAAATATAACAGCAGG

GCACATCGTCCTAACATTAATTGGCGTATACGGAAGAGCCGCTATATTTAACTCTGCTTTCTCATTTATATTTCTTCTAT

CACTACAATCTTTCTACATCTTATTCGAACTAGCAATTTGTTTAATTCAAGCATTCATTTTCTGCCTTCTTCTGTCATTA

TACTCAGAAGATCACCCGCACATTAAAGAATTATAA--------------------------------------------

--------------------------------------------------------------------------------

--------------------------------------------------------------------------------

--------------------------------------------------------------------------------

--------------------------------------------------------------------------------

--------------------------------------------------------------------------------

--------------------------------------------------------------------------------

--------------------------------------------------------------------------------

--------------------------------------------------------------------------------

--------------------------------------------------------------------------------

--------------------------------------------------------------------------------

--------------------------------------------------------------------------------

--------------------------------------------------------------------------------

----------------------------------------------------------------------------

>TERA02442

ATGACCAAGATGGGCAAGCCTCATGGACTTAGGACAGCCCGTAAGATGCGGGACCACCGCCGTGACCAGAGGTGGCATGA

TAAAGACTACAAAAAGAGTCATTTGGGTACTCGCTGGAAGGCCAATCCATTTGGTGGCGCATCCCATGCTAAGGGAATCG

TCCTCGAGAAAGTAGGTGTTGAAGCCAAACAGCCCAACTCTGCCATTCGTAAATGTGTCAGAGTGCAGTTGATAAAAAAT

GGCAAGAAAATTACTGCTTTTGTACCAAGGGACGGTTGTCTGAACTACATCGAGGAGAATGATGAAGTTCTCATTGCTGG

ATTTGGTCGTAAAGGTCATGCTGTAGGAGATATTCCTGGTGTACGCTTCAAGGTTGTTAAAGTGGCAAATGTGTCCCTGT

TGGCCTTGTACAAGGAGAAGAAGGAAAGACCAAGGTCTTAA---------------------------------------

--------------------------------------------------------------------------------

--------------------------------------------------------------------------------

--------------------------------------------------------------------------------

--------------------------------------------------------------------------------

--------------------------------------------------------------------------------

--------------------------------------------------------------------------------

--------------------------------------------------------------------------------

--------------------------------------------------------------------------------

--------------------------------------------------------------------------------

--------------------------------------------------------------------------------

----------------------------------------------------------------------------

>TERA02466

ATGAGGATGCCTGTCCAGCAGACGATGTCGGTTGACTCTCATCGTGCCAAGATGATGACTTTGGTTGAACAGATGCGCAT

ACATTACAAGATGGAGCGTGTACGCATGTCGGAGACTACTCGTGATCTGGTGAAGTACTGCGAGCAGAACATGCCATCAG

ATCCACTGATTTATCCAGTAAAGGATAATCCATTCAAGGAGAAGAAGGGATGTGTCATTTTGTAA---------------

--------------------------------------------------------------------------------

--------------------------------------------------------------------------------

--------------------------------------------------------------------------------

--------------------------------------------------------------------------------

--------------------------------------------------------------------------------

--------------------------------------------------------------------------------

--------------------------------------------------------------------------------

--------------------------------------------------------------------------------

--------------------------------------------------------------------------------

--------------------------------------------------------------------------------

--------------------------------------------------------------------------------

--------------------------------------------------------------------------------

--------------------------------------------------------------------------------

----------------------------------------------------------------------------

>TERA02477

ATGGCCATGAGTATCCCGGCAAGTCAACTGAAGGATTCCGGAGTAGAGCTTGACTCGTCAGAGAACACCGCCCTTGATGA

AGGAAGACGCGCTTCATGGTTGGAGACAAGAGATCTGGAAAATGACTTCAAAGATCTCGTCTTCCTGACGCTACAAGAGC

TCGCCCAGGAAGGACGCATCGACCCGCGCGTTATTGTTGAGGAAAACAGTCTGGATACAAAGGAGAAGCGTGGCAGATGG

CAGGGATTCTGCTTCAGAAGGACCAAGACCGGCCGCTTCCTGCCCTACTAA-----------------------------

--------------------------------------------------------------------------------

--------------------------------------------------------------------------------

--------------------------------------------------------------------------------

--------------------------------------------------------------------------------

--------------------------------------------------------------------------------

--------------------------------------------------------------------------------

--------------------------------------------------------------------------------

--------------------------------------------------------------------------------

--------------------------------------------------------------------------------

--------------------------------------------------------------------------------

--------------------------------------------------------------------------------

--------------------------------------------------------------------------------

----------------------------------------------------------------------------

>TERA02478

ATGTTCTGTCAGCTGTGCCAGGAAGATGCCATCAGCATGCCGTTCCTGCCGCTGATCAAAGAAGGCGAGTTCCGCTTCCT

GATGAGCAAAGGCAAGATCCTGGAGGTCGTACACAAACGTCCCGTCGACGCCAGCGCCTTCACGGCCACGCTGCGCAGTG

GCGCCGTCTACACGATTCTGGATCTGTAA---------------------------------------------------

--------------------------------------------------------------------------------

--------------------------------------------------------------------------------

--------------------------------------------------------------------------------

--------------------------------------------------------------------------------

--------------------------------------------------------------------------------

--------------------------------------------------------------------------------

--------------------------------------------------------------------------------

--------------------------------------------------------------------------------

--------------------------------------------------------------------------------

--------------------------------------------------------------------------------

--------------------------------------------------------------------------------

--------------------------------------------------------------------------------

--------------------------------------------------------------------------------

----------------------------------------------------------------------------

>TERA02479

ATGAGAAGAGTGACTCATGTTGACCATTACGAGCGACCACTCGGTAATTCTCGTACGATGGTCGGCCCATTCCGCCATTC

CGGTGTTGTGGTTACAACTGACGATGGCAAAAGATGGCTAGTACACAAGGGAGACGGTTATGGACGTTCTAGCCAAACGG

TTGTGACGGACGCCAAACACATGAGTGACAGATGGCGTCGTGTAGGTGGACATGACGTATCTAATTCTCGTGTTGGTGAT

TACGTACGAGCTGGTGGCTCAGACTACAGCCTGACAAGAGATAATTGTCATGACGGCAGATGTAGAATGATGGAATTGTA

A-------------------------------------------------------------------------------

--------------------------------------------------------------------------------

--------------------------------------------------------------------------------

--------------------------------------------------------------------------------

--------------------------------------------------------------------------------

--------------------------------------------------------------------------------

--------------------------------------------------------------------------------

--------------------------------------------------------------------------------

--------------------------------------------------------------------------------

--------------------------------------------------------------------------------

--------------------------------------------------------------------------------

--------------------------------------------------------------------------------

----------------------------------------------------------------------------

>TERA02482

ATGGGTCGTATGCATGCACCTGGAAAGGGTATTTCCCAGTCAGCCCTGCCCTATAGGAGAAGTGTTCCAACATGGTTGAA

GTTAACACCTGATGATGTCAAGGAGCAGATCTATAAGTTGGCCAAGAAGGGTTTGACTCCATCTCAGATTGGTGTGATCC

TTAGGGATTCCCATGGAGTTGCCCAGGTTAGATTTGTCACTGGCAACAAGATCCTGAGAATCCTGAAAGCCAAAGGTTTG

GCACCAGACCTGCCAGAGGATTTGTATTACTTGATCAAGAAGGCTGTGGCCATTAGGAAACATCTGGAACGTAATAGGAA

GGACAGAGATGCCAAATTCCGTCTGATTTTGGTTGAAAGCAGAATTCATAGACTGGCTCGATACTACAAGACCAAACGTG

TGCTGCCACCAAACTGGAAGTACGAGTCATCCACTGCCTCGGCTCTTGTTTAA---------------------------

--------------------------------------------------------------------------------

--------------------------------------------------------------------------------

--------------------------------------------------------------------------------

--------------------------------------------------------------------------------

--------------------------------------------------------------------------------

--------------------------------------------------------------------------------

--------------------------------------------------------------------------------

--------------------------------------------------------------------------------

--------------------------------------------------------------------------------

--------------------------------------------------------------------------------

----------------------------------------------------------------------------

>TERA02483

ATGGCGGACCATGAGATCGATTTGGCCGAAGCCGCCGAGAAAAAGAAGAGACGTACCTTCAGAAAGTACACCTATCGTGG

CGTCGACCTCGACCAACTGTTAGATATGTCAAGTGATCAGCTCATGCAACTTTTCCATTGTCGTGCTCGAAGACGTTTTT

CTCGTGGTCTGAAACGCAAACCAATGGCTCTGTTGAAAAGACTACGTAAAGCCAAGAAAGAAGCTCCACCCATGGAAAAG

CCAGAGTGCATCAAGACACATCTGCGTGACATGATCATTGTTCCTGAAATGATTGGCAGCATTGTTGGTGTGTACAATGG

CAAGACTTTCAATCAAGTAGAGATTAAGCCTGAGATGATTGGTCACTATCTTGGAGAGTTCAGCATCACCTACAAGCCAG

TTAAACATGGAAGGCCTGGTATTGGTGCCTAA------------------------------------------------

--------------------------------------------------------------------------------

--------------------------------------------------------------------------------

--------------------------------------------------------------------------------

--------------------------------------------------------------------------------

--------------------------------------------------------------------------------

--------------------------------------------------------------------------------

--------------------------------------------------------------------------------

--------------------------------------------------------------------------------

--------------------------------------------------------------------------------

--------------------------------------------------------------------------------

----------------------------------------------------------------------------

>TERA02485

ATGAAGGTCGACATGGCTTCAAAAGCTGTTCCAGCATGGAGAGAGTCTCTCAGGCGATGGGCCTACTACAAGTCCTACTT

CCCACAGTTAGGTTTAATGCGAGATGACACTCTCCATGAGACTCCAGCGGTAGCTGAGGCAATTCGTCGCCTTCCCAAAC

AGATCCAGGGGGAGAGATCCTTCCGTATTAGTCGTGCTTTTCTCCTTTCAACACAGAAAGCAATTCTTCCCAAAGAAGAG

TGGACTAAATATGAAGAGGATGTGCACTAA--------------------------------------------------

--------------------------------------------------------------------------------

--------------------------------------------------------------------------------

--------------------------------------------------------------------------------

--------------------------------------------------------------------------------

--------------------------------------------------------------------------------

--------------------------------------------------------------------------------

--------------------------------------------------------------------------------

--------------------------------------------------------------------------------

--------------------------------------------------------------------------------

--------------------------------------------------------------------------------

--------------------------------------------------------------------------------

--------------------------------------------------------------------------------

----------------------------------------------------------------------------

>TERA02486

ATGAAAACATACGCCACTCCTAGACGTCCCTTCGAGAAGGAACGTCTCGACCAGGAGTTAAAATTAATCGGAGAGTATGG

TCTGAGGAACAAGCGCGAGGTGTGGAGGGTCAAGTACACGCTGGCCAAAATCCGTAAGGCTGCTAGAGAACTGCTGACCT

TGGACGAGAAGGACCCGAGGCGTCTATTCGAAGGTAATGCTCTGTTGCGAAGACTCGTGCGTACGGGCGTGCTCGACGAG

TCCAAGATGAAGCTCGATTATGTGTTGGGTCTGCGTCTTGAGGACTTTCTGGAACGCCGTCTTCAGACGCAGGTGTTCAA

GCTCGGACTGGCCAAGAGCATTCATCATGCTCGTGTGCTGATTCGCCAGAGACACATCAGAGTACGTAAACAGGTGGTCA

ACATCCCTTCCTACATTGTGAGACTGGACTCGCAGAAACACATAGACTTCTCACTGAGATCACCTTATGGTGGTGGTAGA

CCAGGTCGTGTTAAGAGGAAAAACATGAGAAAGGGAGCTGGTGGTGCAGCTGATAATGAAGATGAAGATTAA--------

--------------------------------------------------------------------------------

--------------------------------------------------------------------------------

--------------------------------------------------------------------------------

--------------------------------------------------------------------------------

--------------------------------------------------------------------------------

--------------------------------------------------------------------------------

--------------------------------------------------------------------------------

--------------------------------------------------------------------------------

--------------------------------------------------------------------------------

----------------------------------------------------------------------------

>TERA02492

ATGGAGATGTCACTAGAATCAATGAATCGCTATGTTAGCCCAATCAATCCTGCTGTGTTCCCACATCTGACTGTAGTCCT

TCTTGGCATTGGAATCTTCTTTATGGCATGGTTCTTTGTATATGAAGTCACATCAACAAAGTTCACAAGAGACCTATTCA

AAGAGTTGCTTGTGGCCCTTGTTGCTTCTGTCTTCATGGGATTTGGAGTGTTGTTCTTATTACTCTGGGTTGGAATCTAT

TAA-----------------------------------------------------------------------------

--------------------------------------------------------------------------------

--------------------------------------------------------------------------------

--------------------------------------------------------------------------------

--------------------------------------------------------------------------------

--------------------------------------------------------------------------------

--------------------------------------------------------------------------------

--------------------------------------------------------------------------------

--------------------------------------------------------------------------------

--------------------------------------------------------------------------------

--------------------------------------------------------------------------------

--------------------------------------------------------------------------------

--------------------------------------------------------------------------------

----------------------------------------------------------------------------

>TERA02493

ATGCAGCAGTCGGTCCAAGTATATGGGCGAAAGAAAACAGCAACTGCTGTTGCCCATTGCAAACGTGGCCGTGGTCTGAT

CAAGGTAAATGGACGTCCACTGGATCAGCTCCAACCAGAAATGCTTCGATACAAGCTTCAAGAACCTGTTCTGCTGCTAG

GAAAGAAGAGATTTGAAGGTGTTGACATCCGTGTCCGTGTGAAGGGTGGTGGTCACATAGCCCAGGTTTATGCTATTAGG

CAAGCCATTTCCAAGGCACTTGTGGCTTATTACCAAAAATATGTTGACGAGGCATCAAAGAAGGAGATAAAGGACATGCT

GATTGCCTATGACAGAACTCTTTTGGTTGCTGATCCAAGAAGATGTGAACCCAAAAAGTTTGGAGGCCCTGGCCCCAGAG

CTCGCTACCAGAAGTCATACCGATAA------------------------------------------------------

--------------------------------------------------------------------------------

--------------------------------------------------------------------------------

--------------------------------------------------------------------------------

--------------------------------------------------------------------------------

--------------------------------------------------------------------------------

--------------------------------------------------------------------------------

--------------------------------------------------------------------------------

--------------------------------------------------------------------------------

--------------------------------------------------------------------------------

--------------------------------------------------------------------------------

----------------------------------------------------------------------------

>TERA02522

ATGAAAGAAGTTCGCATGACCGATAATACAATCCAAAACCTTCAGTCGCGCGACCCATTCGCTGACGATGAAGGTAAACT

TGGAAGTGGCAGTGATGGTTACATCCATATAAGGATTCAACAGAGAAACGGACGGAAAACCTTAACAACAGTTCAGGGTA

TCTCTTCAGAATTTGACTTGAAAAAGATAGTCAAAGTCGCTAAAAAGGAGTTTGCCTGTAACGGAACAGTTGTGGAACAT

CCTGAATACGGGGAGGTTATCCAGCTTCAGGGAGACCAGCGTCAACGCATTTCGCAGTTCCTCAAAAAGTTTGGCTTGGC

CCGTGAGGAGCAGATCAAGGTTCATGGTTTCTAA----------------------------------------------

--------------------------------------------------------------------------------

--------------------------------------------------------------------------------

--------------------------------------------------------------------------------

--------------------------------------------------------------------------------

--------------------------------------------------------------------------------

--------------------------------------------------------------------------------

--------------------------------------------------------------------------------

--------------------------------------------------------------------------------

--------------------------------------------------------------------------------

--------------------------------------------------------------------------------

--------------------------------------------------------------------------------

----------------------------------------------------------------------------

>TERA02543

ATGCCGTTTGAATTTTTGTGTTGGTGGCCGAGAATCCGTGCCATCATGAAATTAGTACGTTTTCTGATGAAGCTCAGTCA

TGAGACTGTTACAATTGAATTAAAGAATGGAACACAAGTCCATGGAACAATTACAGGTGTTGATGTCAGTATGAACACCC

ATCTGAAGTCTGTCAAGATGACAGTCAAGAACAAGGATCCAGTCCAGCTGGAAAGTCTCAGTATCCGTGGTAACAACATC

CGCTACTTCATACTGCCCGACAGTCTTCCACTGGACAACCTGCTCATTGATGACACACCAAGAGCAAAGGCCAGGAAGGA

TGCCAGAGGTTAA-------------------------------------------------------------------

--------------------------------------------------------------------------------

--------------------------------------------------------------------------------

--------------------------------------------------------------------------------

--------------------------------------------------------------------------------

--------------------------------------------------------------------------------

--------------------------------------------------------------------------------

--------------------------------------------------------------------------------

--------------------------------------------------------------------------------

--------------------------------------------------------------------------------

--------------------------------------------------------------------------------

--------------------------------------------------------------------------------

----------------------------------------------------------------------------

>TERA02544

ATGTTGGCACGGGTTATCCTCAATGTTCAGCAGTGTCGTCACTTGCTGTTTAGACAGTTCCGTCGTAACATTGGCATATC

AGCTGTAGTTTCTCAGAAAGCTAGCCAAAACCTAGATCCAATTCAGCAACTATTTATTGAAAAGATCCGAGAATATAATG

AAAGAAAGAAAAGTTCTGGTGGATTAGTTGATGCAACTGATGAAGATAAGAAAGCTTTGCAAGAGGATTTAGATAAGATT

GCCAGGCAGTATGGTGCAGATGGAGCAGATTTTACCAAATTTCCAACATTTTCCTTCTCAGACCCAGAGTTAGAACCAGT

TGGTGTTGAAGTTGAAGTAAAATAA-------------------------------------------------------

--------------------------------------------------------------------------------

--------------------------------------------------------------------------------

--------------------------------------------------------------------------------

--------------------------------------------------------------------------------

--------------------------------------------------------------------------------

--------------------------------------------------------------------------------

--------------------------------------------------------------------------------

--------------------------------------------------------------------------------

--------------------------------------------------------------------------------

--------------------------------------------------------------------------------

--------------------------------------------------------------------------------

----------------------------------------------------------------------------

>TERA02549

ATGAAAATGGCTGACCAACCGAAGAAGCCAGATCTTGGTCTTTTGGAAGAAGACGACGAATTCGAGGAGTTTCCAGCGGA

AGACTGGACAGGTGCTGATGAGGATAAGACAGATGAGAATGTTTGGGAGGACAACTGGGATGATGAGAACATTGAGGATG

ACTAA---------------------------------------------------------------------------

--------------------------------------------------------------------------------

--------------------------------------------------------------------------------

--------------------------------------------------------------------------------

--------------------------------------------------------------------------------

--------------------------------------------------------------------------------

--------------------------------------------------------------------------------

--------------------------------------------------------------------------------

--------------------------------------------------------------------------------

--------------------------------------------------------------------------------

--------------------------------------------------------------------------------

--------------------------------------------------------------------------------

--------------------------------------------------------------------------------

--------------------------------------------------------------------------------

----------------------------------------------------------------------------

>TERA02560

ATGCTTTTCCTCCGTCGGTGTAGGAGTCCGACAATGGGTCGAGTAATTAGGAGTCAGCGTAAAGGCGCTGGTAGTGTGTT

TAAGGCACACACAAAACACAGGAAGGGTCCGGCAAGATTGAGAGCGTTCGACTTTTCCGAGAGACACGGATACATCAAAG

GCGTTATAAGGGACATAATTCATGATCCAGGACGTGGGGCTCCCCTGGCCCATGTAGTGTTCCGTGATCCTTACAGATAC

AAGCTGAAACATGAATTCTTCATCGCCTGTGAGGGAATGTACACAGGACAGTTTGTCTACTGTGGCAAAAAAGCCACACT

GCAGATTGGCAACATCTTGCCAGTGGGTGTGATGCCTGAAGGCACAGTGGTCTGCTCTCTGGAGGAGAAAACTGGTGACC

GTGGACGACTGGCCAAGTGCTCTGGAAATTATGCCACTATCATCTCACACAATCCAGAAACGAAAAGGACGAGGGTAAAG

CTTCCATCTGGCACCAAGAAGGTCATTCCTTCAACCAACAGAGCATGTATTGGTGTTGTTGCTGGAGGTGGTCGTATTGA

CAAGCCAATGTTGAAGGCTGGCCGTGCTTACCACAAGTACAAGGCCAAGAGGAACTGCTGGCCAAAAGTTCGTGGTGTGG

CCATGAACCCTGTTGAGCATCCCCACGGTGGTGGTAACCATCAGCACATTGGAAAAGCATCCACTGTCAGGAGAGATACA

TCTGCTGGTAGAAAGGTTGGTCTGATTGCTGCTCGCCGTACTGGACGTCTACGTGGTACAAAGCAGGTGACAGGCAAGAG

TTAA----------------------------------------------------------------------------

--------------------------------------------------------------------------------

--------------------------------------------------------------------------------

--------------------------------------------------------------------------------

--------------------------------------------------------------------------------

--------------------------------------------------------------------------------

----------------------------------------------------------------------------

>TERA02563

ATGTTCACCCGAGAGCTTGCCGAAGATGGATACAGTGGTGTAGAGGTACGCGTCACACCAGCACGGACAGAAATTATCAT

CCTGGCCACCAGAACACAGAATGTCCTTGGTGAGAAGGGCCGTCGCATCAGAGAACTGACCTCTGTTGTGCAGAAGAGGT

TCAACTTCCAAGAAGGCACTGTTGAGCTGTATGCTGAGAAGGTGGCTACTCGAGGCCTGTGTGCCATTGCCCAAGCTGAA

TCATTAAGGTACAAGCTTATTGGAGGCCTGGCTGTTAGAAGGGCATGTTATGGTGTGCTGAGGTTCATCATGGAATCTGG

AGCCAAGGGCTGCGAGGTTGTGGTTTCTGGTAAGCTGAGAGGTCAGCGAGCCAAGTCCATGAAGTTTGTAGATGGTCTGA

TGATTCACAGTGGTGAACCTTGCAATGAGTATGTGGACACAGCTGTCAGACATGTATTGCTCAGACAAGGTGTTCTGGGA

ATTAAGGTCAAGATCATGTTGCCATGGGATCCATCTGGTAAGATAGGACCAAAGCGTCCCCTTCCTGATAACATCAGCAT

TGTTGAGCCAAAGGATGAAATTCCTCCAGCACAGCCATACAGTGAACACAAGGGCTAA----------------------

--------------------------------------------------------------------------------

--------------------------------------------------------------------------------

--------------------------------------------------------------------------------

--------------------------------------------------------------------------------

--------------------------------------------------------------------------------

--------------------------------------------------------------------------------

--------------------------------------------------------------------------------

--------------------------------------------------------------------------------

----------------------------------------------------------------------------

>TERA02564

ATGGGTCTCCGACCTCTCATCAAGAGGAAGGTGGTGAAGAAGCGAACCAAGAAATTCATCAGACATCAGTCAGACAGATA

TGCAAAATTAAAGCCCAATTGGCGTAAGCCTCGTGGTATTGACAACAGAGTCAGGAGGAAGTTCAAAGGTCAATATTTGA

TGCCAAACATTGGTTATGGCAGCAACAAGAAGACAAAACACATATGCCCTGATGGCTTCAGAAAATTCCTTGTTCACAAT

GTTAGGGAGCTGGAGATTCTTATGATGCAAAACAAACGATTCTCTGCCGAGATAGCTCACACTGTGTCAAGCAAGAAGAG

GAAGGACATTGTGGAGCGAGCTCAACAGCTGTCCATTAAAGTAACTAATGCCAATGCTCGCATCAGATCAGAGGAGAACG

AGTAA---------------------------------------------------------------------------

--------------------------------------------------------------------------------

--------------------------------------------------------------------------------

--------------------------------------------------------------------------------

--------------------------------------------------------------------------------

--------------------------------------------------------------------------------

--------------------------------------------------------------------------------

--------------------------------------------------------------------------------

--------------------------------------------------------------------------------

--------------------------------------------------------------------------------

--------------------------------------------------------------------------------

----------------------------------------------------------------------------

>TERA02574

ATGGACAAATGGGATGGCAATGCCCTGAAAAATGCCTTGGATGATGCAACCAAAAAGGTTCTAATTGAAGACATGTCTTA

TGATGAGTGCCACAAGTTGATGGATGGACGTCTGGCTATATGTACCCTGTCAGTGAGCTTTGCTATCTTCGCCTTGGCCT

GGGATTACCTGCATCCATTTCCAGAATCTCGTCCGGTGCTCATCTTCTGTGTAGCCTCATATTTTGTTATGATGGGTGTT

CTGACTCTGTACACAACACTTAAAGAGAAAGGCTGCTTCCTTGTTGCACTGGATAAGGACAAAGCTGGAATCGACCCACC

AAATGTTTGGAAGTTTGCCTCATAA-------------------------------------------------------

--------------------------------------------------------------------------------

--------------------------------------------------------------------------------

--------------------------------------------------------------------------------

--------------------------------------------------------------------------------

--------------------------------------------------------------------------------

--------------------------------------------------------------------------------

--------------------------------------------------------------------------------

--------------------------------------------------------------------------------

--------------------------------------------------------------------------------

--------------------------------------------------------------------------------

--------------------------------------------------------------------------------

----------------------------------------------------------------------------

>TERA02595

ATGCCACCGGACAAGATGGGTGTTCGTGGTCCAAGGAAGCACTTGAAGAGGCTTCATGCCCCTAAACACTGGATGTTGGA

TAAGCTTGGGGGTGTTTTTGCCCCACGCCCAAGCACTGGTCCACACAAGATGCGAGAGTGTCTCCCTCTGGTGGTGTTCT

TGAGGAATCGCCTAAAGTATGCTTTGACATATGATGAGGTGAAGAAGATTGTAAATCAACGTCTGATCAAGGTTGATGGC

AAAGTGAGAACTGACAAGACGTACCCTGCTGGCTTTATGGATGTGATCACAATAGAGAAGACAGCTGAAAACTTCCGTCT

CATTTATGATGTCAAGGGTCGTTTCACCATTCATCGTATCACTTCCCAAGAAGCCAAGTACAAACTGTGTAAGGTGAAGA

AGATAATCATTGGGCTGAAGGGAGTTCCACATCTGGTCACTCACGATGCTCGTACCATTCGCTATCCAGATCCAATGATC

AAGGTCAATGACACAGTTCAGGTTGACATTGCCACGGGCAAGATAAAGGATTTCATCAAGTTTGAATCAGGAAATCTGTG

CATGATTACTGGTGGGCATAACTTGGGTCGTGTTGGCCTTGTGACACACAGAGAACGTCATCCTGGTGGCTTTGACATTG

TTCACATCAAGGACAGCATGGGCCACTCATTTGCCACACGGTTGAACAACGTGTTCATCATTGGTAAAGGTAACAAGCCA

TGGGTATAA-----------------------------------------------------------------------

--------------------------------------------------------------------------------

--------------------------------------------------------------------------------

--------------------------------------------------------------------------------

--------------------------------------------------------------------------------

--------------------------------------------------------------------------------

--------------------------------------------------------------------------------

----------------------------------------------------------------------------

>TERA02598

ATGCCATGGCAACAGATCCAAGTTCGTGGTCGCTATGCCAACTGGCAGATGCTGCGTGATGTGAAACGAAGAGCCTGTGT

TAAAGAACATGCAACAGAACGGTTGCGAATTAATGCTCTCAGAAGAAACACCATTTTACCTCCTGTCATAAGGGAGATGG

CCACAAAGGAAATAGAGAAGTTTCCTATCGATTCAAATCGTACACGTCTTGTCAATCGTTGTGTGATGACATCACGGCCT

CGTAATGTTCGTAAACATTGGAGGATCAGCAGAATTATGTGGCGCCACCTGGCAGATTACAACAAGTTGTCAGGCATAAC

ACGGTCATAA----------------------------------------------------------------------

--------------------------------------------------------------------------------

--------------------------------------------------------------------------------

--------------------------------------------------------------------------------

--------------------------------------------------------------------------------

--------------------------------------------------------------------------------

--------------------------------------------------------------------------------

--------------------------------------------------------------------------------

--------------------------------------------------------------------------------

--------------------------------------------------------------------------------

--------------------------------------------------------------------------------

--------------------------------------------------------------------------------

----------------------------------------------------------------------------

>TERA02605

ATGAACATGCCTTTTCAGCCTCCAAAAACCGAGAAATGCGCAACCTGCGGCAAGTCAGTCTACGCCGCCGAGAGGATGGA

AGCTGGAGGCAACATTTATCACAAGCTGTGCTTTAAATGTGCTACCTGCAAGATGCCACTTAAGTTAAACAATTATCAGC

AGAACGAAGGCAAACTTCTCTGCAAAACAGACTACCAGAAGGAGATCTTGGCAAAGAACGCACAAATCTGTACATAA---

--------------------------------------------------------------------------------

--------------------------------------------------------------------------------

--------------------------------------------------------------------------------

--------------------------------------------------------------------------------

--------------------------------------------------------------------------------

--------------------------------------------------------------------------------

--------------------------------------------------------------------------------

--------------------------------------------------------------------------------

--------------------------------------------------------------------------------

--------------------------------------------------------------------------------

--------------------------------------------------------------------------------

--------------------------------------------------------------------------------

--------------------------------------------------------------------------------

----------------------------------------------------------------------------

>TERA02607

ATGGGTAAAAATAAACGGCTTTCCAAAGGAGGCAAGAAAGGGGGCAAAAAGAAGGTAGTCGATCCCTTCTCCAAGAAAGA

TTGGTATGATGTAAAGGCTCCTTCGATGTTTAACATTCGTCAGATTGGCAAAACCTTGGTCACAAGAACTCAAGGAACAA

AGATTGCCTCTGATGGTCTAAAGGGTCGTGTATTTGAGGTGTCACTGGCTGATCTACAGAATGATGAAGTTGCCTTCCGT

AAATTCAAGTTGATGGCTGAAGATGTTCAGGGTCGCAATGTGTTGACCAACTTCCATGGCATGAGTTTGACTACTGACAA

GCTACGATCCATGGTCAAAAAGTGGCAGACTCTGATTGAGGCCACCGTCGATGTACGTACAACTGATGGTTATTTGCTGA

GACTGTTCTGTATTGGCTTTACATCCAAGCGACAGAATCAGATCAAAAAGACCTGCTATGCTCAGAGCACTCAAGTTCGT

GCCATCAGGAAGAAAATGGTTGAAATCATGACCCGCGAAGTATCTTCAAATGACCTCAAGGAAGTTGTCAATAAACTGAT

CCCAGATAGCATTGGTAAGGACATTGAGAAGGCCTGCCAGGGTATCTACCCTCTTCATGATGTACTGATACGTAAAGTGA

AGATCCTCAAGAAACCCAAGTTTGATTAA---------------------------------------------------

--------------------------------------------------------------------------------

--------------------------------------------------------------------------------

--------------------------------------------------------------------------------

--------------------------------------------------------------------------------

--------------------------------------------------------------------------------

--------------------------------------------------------------------------------

--------------------------------------------------------------------------------

----------------------------------------------------------------------------

>TERA02617

ATGGTCATCAAGCAGGATGAGCACCTGTTCCAGCTGCGCAGCAACTGCTGCCCGTACGCCTTCAACTGGATGCCGAACGG

CGAGAACACGAAGTGGCAGCTGTACGTCAGCGACGGAGCCAAGTACAAGGACGGCACCATGGAGGGAATGTGCAGCGACT

GCGACGGCAGCGTCATCAACGACTGGACGAAGTGCTTCTAA---------------------------------------

--------------------------------------------------------------------------------

--------------------------------------------------------------------------------

--------------------------------------------------------------------------------

--------------------------------------------------------------------------------

--------------------------------------------------------------------------------

--------------------------------------------------------------------------------

--------------------------------------------------------------------------------

--------------------------------------------------------------------------------

--------------------------------------------------------------------------------

--------------------------------------------------------------------------------

--------------------------------------------------------------------------------

--------------------------------------------------------------------------------

--------------------------------------------------------------------------------

----------------------------------------------------------------------------

>TERA02629

ATGAGGATGGTGCGTATGAACGTGCTGGCCGATGCCTTGAAGTCTATTTGTAATGCCGAGAAACGAGGCAAGAGGCAGGT

TCTGATCCGACCGTGTTCGAAGGTCATCGTCAGATTCCTGACCGTCATGATGAAGCATGGTTACATTGGTGAGTTTGAGA

TAATTGATGACCACAGAGCAGGAAAAATTGTTGTTAATTTGACAGGAAGACTAAACAAGTGTGGTGTGATAAGTCCAAGG

TTTGATGTTTGCATCAGAGATATGGAGAAATGGACAACTAACCTATTGCCATCAAGACAGTTTGGGTACATTGTGCTCAC

AACATCAGGTGGGATTATGGACCATGAGGAGGCCAGACGAAAACATTTGGGTGGAAAAATTCTAGGCTTCTTTTAA----

--------------------------------------------------------------------------------

--------------------------------------------------------------------------------

--------------------------------------------------------------------------------

--------------------------------------------------------------------------------

--------------------------------------------------------------------------------

--------------------------------------------------------------------------------

--------------------------------------------------------------------------------

--------------------------------------------------------------------------------

--------------------------------------------------------------------------------

--------------------------------------------------------------------------------

--------------------------------------------------------------------------------

----------------------------------------------------------------------------

>TERA02635

ATGTTCATCACAGTATCTGGCCTTGGTGGCTTAGTAGCTGGCTACAGAGGTGGCTTCATACGTAAAGCTGTGTATGCTAG

TATAGGCATGGCAGCTGCCGCATCAATCTGCTATCCAGACCAGGCTCTGGATATATCTAAACAAGCCTGGATTCTGACCA

AGGAGCAAGCCAAGGAGTTCTGGAGACCATAA------------------------------------------------

--------------------------------------------------------------------------------

--------------------------------------------------------------------------------

--------------------------------------------------------------------------------

--------------------------------------------------------------------------------

--------------------------------------------------------------------------------

--------------------------------------------------------------------------------

--------------------------------------------------------------------------------

--------------------------------------------------------------------------------

--------------------------------------------------------------------------------

--------------------------------------------------------------------------------

--------------------------------------------------------------------------------

--------------------------------------------------------------------------------

--------------------------------------------------------------------------------

----------------------------------------------------------------------------

>TERA02641

ATGGAGGAACATTTCGACGAATATGAGCACTGGAACTTCGACTACGACAAGCACATGTTCTCCGGTCGGAGTGGCAAGGG

CAGATCTAAGAAAGAAGCCAGCCTAAACACGAACCACCACGACCTGAATGGTCACACCAGAAAATTAACCACGAAATTCG

TGAACACCTATCACAGACGTAACAGGACGGAATCGAAATAA---------------------------------------

--------------------------------------------------------------------------------

--------------------------------------------------------------------------------

--------------------------------------------------------------------------------

--------------------------------------------------------------------------------

--------------------------------------------------------------------------------

--------------------------------------------------------------------------------

--------------------------------------------------------------------------------

--------------------------------------------------------------------------------

--------------------------------------------------------------------------------

--------------------------------------------------------------------------------

--------------------------------------------------------------------------------

--------------------------------------------------------------------------------

--------------------------------------------------------------------------------

----------------------------------------------------------------------------

>TERA02647

ATGGGTGGTTTTCGTGGTGGATTTGGCTCGGGTGGTCGTGGCAGGGGACGCGGCCGTGGCCGTGGCAGAGGACGGGGCCG

AGGACGTGGTGCCAAGGAAGACAAGGAATGGATGCCAGTAACCAAGCTGGGACGACTCGTCAAAGACATGAAGATCAAGT

CCATTGAAGAGATCTACCTGTTCTCGTTGCCCATAAAGGAGTATGAGATCATTGACCATTTCCTGCCCACGCTGAAGGAT

GAAGTGTTGAAAATCATGCCAGTACAGAAGCAAACAAGAGCTGGTCAAAGAACTAGGTTCAAGGCTTTTGTTGCCATTGG

AGATTACAATGGTCATGTTGGTTTGGGTGTGAAGTGTTCTAAGGAAGTAGCCACTGCCATCCGTGGAGCCATCATCTTGG

CCAAACTGTCCATTGTTCCAGTACGCAGAGGTTACTGGGGTAACAAGATCGGAAAGCCCCACACTGTTCCCTGCAAGGTT

ACTGGAAAGTGTGGTAGTGTACTGGTACGTCTGATTCCAGCACCACGTGGTACAGGTATTGTCAGTGCACCTGTGCCAAA

GAAGCTGCTCCAGATGAGTGGCATTGATGACTGCTACACTTCAGCCAGAGGACAGACGGCCACCCTTGGAAACTTTGCAA

AGGCCACCTACGAAGCCATTGCCAACACATACAGTTACCTGACGCCAGACTTCTGGAAGGAGACCATCTTCACCAAGTCT

CCGTACCAGGAGTTCACAGACTACTTGGCTCGCACACATACGCGAGGTGGTGTCCAGAGATTGGAACAAAAGTAA-----

--------------------------------------------------------------------------------

--------------------------------------------------------------------------------

--------------------------------------------------------------------------------

--------------------------------------------------------------------------------

--------------------------------------------------------------------------------

--------------------------------------------------------------------------------

----------------------------------------------------------------------------

>TERA02663

ATGTTCGACTCCTCTCACAAACTCTTCAAGCGATGCTTCAATAAGGGATACGCATGGGAAGTGTTGGAGTTATTCTCAGG

TCCGCCTCGTGTTTCCTTAACATGGCGCCACTGGTCACATTTTACCGGTAACTATGGCGACCACAAGCCAACGGGAGAAC

TGCTGGAAATGATTGGTGCCGCTGTCATCACACTGAACGACAAGAACCAGATGGAGTAA---------------------

--------------------------------------------------------------------------------

--------------------------------------------------------------------------------

--------------------------------------------------------------------------------

--------------------------------------------------------------------------------

--------------------------------------------------------------------------------

--------------------------------------------------------------------------------

--------------------------------------------------------------------------------

--------------------------------------------------------------------------------

--------------------------------------------------------------------------------

--------------------------------------------------------------------------------

--------------------------------------------------------------------------------

--------------------------------------------------------------------------------

--------------------------------------------------------------------------------

----------------------------------------------------------------------------

>TERA02671

ATGTTTTCCGGTTCGCCATCACAGTCGACCAAGATGTTGATGCCAAAGAAGAACCGCGTTGCCATTTATGAGCTGCTCTT

CAAGGAGGGCGTCATGGTAGCCAAGAAGGATTATTATGCCCCCAAACACCCCGAGCTCGATGTACCGAACCTTCAGGTCA

TTAAGGCCATGCAGTCTCTGAAGTCTCGTGGTTTAGTCAACGAGCAGTTTGCCTGGAGACATTACTACTGGTATCTGACG

AACGAAGGCATCCAGTACCTTAGGGACTTTCTCCACCTGCCGCCGGAGATCGTACCAGCCACTCTGAAACGTCAGAGTCG

TCCAGAACCGGCTCGAGCCAGGCCAAAGATGAGTGAGGCCCCACGTAGCCAGGTTCCTGAAGACAGGGCAGCATACAGGA

GAGCCGCTCCAGGCGGTCCAGACAAGAAGGCTGATGTTGGTGCTGGTACTGGCGATTTCTCCTTCCGTGGTGGCTTTGGG

AGAGGACGTGGCGCGCCAAGAGAGTAA-----------------------------------------------------

--------------------------------------------------------------------------------

--------------------------------------------------------------------------------

--------------------------------------------------------------------------------

--------------------------------------------------------------------------------

--------------------------------------------------------------------------------

--------------------------------------------------------------------------------

--------------------------------------------------------------------------------

--------------------------------------------------------------------------------

--------------------------------------------------------------------------------

----------------------------------------------------------------------------

>TERA02688

ATGGATGTACCCATCACAGTGAATCAAGGACGACAAAAATTGAGGGAACTATTTTTAAAAAATAAGCATGTCACCGATCT

TCGGACAATTGACATGTTGGTTATCAAGGGCCAGATGGAGCTGGTGGAGACTGTCCAACTGTGGAAGCAGAAGACCCATG

TGATGAGGTACTTCAGTGATACTCACAACCCACGACCCAGCGACTTCTTGTCCAAATTTTATGATGGATACATGTAA---

--------------------------------------------------------------------------------

--------------------------------------------------------------------------------

--------------------------------------------------------------------------------

--------------------------------------------------------------------------------

--------------------------------------------------------------------------------

--------------------------------------------------------------------------------

--------------------------------------------------------------------------------

--------------------------------------------------------------------------------

--------------------------------------------------------------------------------

--------------------------------------------------------------------------------

--------------------------------------------------------------------------------

--------------------------------------------------------------------------------

--------------------------------------------------------------------------------

----------------------------------------------------------------------------

>TERA02691

ATGCCCATGGCAACAAGTGCTCTTCAGGAGATGAAAAATTTCTGGAAGAAGAACCAGGAACTTGGCCGTCCAAGTTCACC

ATGGGTTATATACAGGCCTCATCTCCCTATGTTGACCTCTCTGACACACAGGATGACAGGCATTGCAATGGGAGTTGCCC

TCTATGGCATTTCTGTTGGCCTGTTCCTGGCTCCAGGTGACTTCCCCAGCTACATTGAATTTGTCAAGTCCCTGAACCTG

TCACCACTCATTCTTTTCCCTATCAAATCTGTAATGGCTTTCCCACTAGTGTACCATTACATGAATGGCATTCGCCATTT

GACATGGGATGCTGGCTATGGATTTAAACTAGCCACACAGTACTAA----------------------------------

--------------------------------------------------------------------------------

--------------------------------------------------------------------------------

--------------------------------------------------------------------------------

--------------------------------------------------------------------------------

--------------------------------------------------------------------------------

--------------------------------------------------------------------------------

--------------------------------------------------------------------------------

--------------------------------------------------------------------------------

--------------------------------------------------------------------------------

--------------------------------------------------------------------------------

--------------------------------------------------------------------------------

----------------------------------------------------------------------------

>TERA02700

ATGAGTACACTCCGTTTGCAGAAGCGGTTGGCCGCTTCAGTTCTGAAATGTGGCCGTAATAAGATTTGGCTTGATCCAAA

TGAAACCAATGAGATAGCTAATGCTAATTCAAGACAAAACATCAGAAAATACATCAAGAATGGTTTGATCATCCGTAAAC

CTGTGGCAGTCCACTCACGTGCACGTGTCCGCAAGAACACAGAGGCTCGCCGCAAGGGTCGTCATACTGGCCATGGTAAA

AGGAAGGGTACGCGCAATGCCCGTATGCCACAGAAAATCATCTGGATGCGTCGCATGAGAGTACTGCGACGTCTTCTGAA

ACGATACAGGGAATCGAAGAAGATTGACAAGCATCTGTATCATCTGCTGTACATGAAGTGCAAAGGTAACGTCTTCAGAA

ACAAGAGGGTCTTGATGGAGCACATCCACAAGGAGAAGGCAAGAAAGGCTAGAATGAAGATGCTGAATGACCAGGCGGAG

GCTCGCAGACAGAAAGTACGTGAGGCTCGCCGTCGTCGTGAGGAGAGAGTGGCCCAGAAGAAGCAGGAACTGTTGAAGAG

CTTTGCCAAGGAATAA----------------------------------------------------------------

--------------------------------------------------------------------------------

--------------------------------------------------------------------------------

--------------------------------------------------------------------------------

--------------------------------------------------------------------------------

--------------------------------------------------------------------------------

--------------------------------------------------------------------------------

--------------------------------------------------------------------------------

--------------------------------------------------------------------------------

----------------------------------------------------------------------------

>TERA02705

ATGCAAATGTCACAGATCCAGAGACAGATCCAACAGGATTGGGCCAACCGAGAGTACATTGAAGTGATAACATGTAGTAT

CAAGAAAATATCGGATTTTCTTAACTCATTTGATGTGTCATGCCGATCAAGACTTGCTACACTCAATGAAAGGCTGTCTG

CTTTGGAAAGGAGAGTTGAATACATTGAAGCAAGAGTGACATAA------------------------------------

--------------------------------------------------------------------------------

--------------------------------------------------------------------------------

--------------------------------------------------------------------------------

--------------------------------------------------------------------------------

--------------------------------------------------------------------------------

--------------------------------------------------------------------------------

--------------------------------------------------------------------------------

--------------------------------------------------------------------------------

--------------------------------------------------------------------------------

--------------------------------------------------------------------------------

--------------------------------------------------------------------------------

--------------------------------------------------------------------------------

--------------------------------------------------------------------------------

----------------------------------------------------------------------------

>TERA02712

ATGGCCGTCAACATGTCCAAGAGAGGTCGTGGAGGTTCGTCAGGTGGTAAATTCCGTATTGCTCTGGCCCTGCCAGTTGG

CGCAGTAATCAACTGTGCCGACAACACAGGTGCCAAAAACTTGTATATCATCGCTGTACAGGGAATTAAAGGTCGTTTGA

ACCGTTTGCCTGCTGCCGCCTCTGGTGACATGGTTGTTGCTACTGTGAAGAAGGGAAAACCCGAGCTGAGGAAAAAAGTT

ATACCAGCGGTAGTTATCAGACAGAGAAAGCCAATACGACGGAAAAATGGAGTAGTCATATACTTTGAAGACAATGCTGG

GGTTATAGTAAACAATAAAGGCGAAATGAAAGGGTCAGCTATCAATGGTCCTGTGGCAAAGGAATGTGCAGACCTGTGGC

CAAGGATTGCATCCAATGCTAGCTCCATACACTAA---------------------------------------------

--------------------------------------------------------------------------------

--------------------------------------------------------------------------------

--------------------------------------------------------------------------------

--------------------------------------------------------------------------------

--------------------------------------------------------------------------------

--------------------------------------------------------------------------------

--------------------------------------------------------------------------------

--------------------------------------------------------------------------------

--------------------------------------------------------------------------------

--------------------------------------------------------------------------------

----------------------------------------------------------------------------

>TERA02727

ATGAGGGCTGGTGCGCATCACTTCGGCAAATATTGTGACCAACAGAGCAAGGAATTCATGTTGTGTAACCAGGAGACAAA

AGATCCACGTAAATGTCTGGAAGAAGGCAAACAGGTGACAAAATGTGGTCTGGAGTTCTTCAAAAAAGTTAAACAACACT

GTGCTTAA------------------------------------------------------------------------

--------------------------------------------------------------------------------

--------------------------------------------------------------------------------

--------------------------------------------------------------------------------

--------------------------------------------------------------------------------

--------------------------------------------------------------------------------

--------------------------------------------------------------------------------

--------------------------------------------------------------------------------

--------------------------------------------------------------------------------

--------------------------------------------------------------------------------

--------------------------------------------------------------------------------

--------------------------------------------------------------------------------

--------------------------------------------------------------------------------

--------------------------------------------------------------------------------

----------------------------------------------------------------------------

>TERA02742

ATGCACGAAGAACGCTTTCTGGCCAAAATTGGCTGGAGCACTGATCAGAGAGGATTTGGTGAGGAGCCTGGAATCAAAGC

ACAAATAATTAACCTGATTAGATCAGTAAGAACAGTTATGAGAATTCCCTTGATTGCAGTAAACACGGTGATGATCTTAT

TGAAGCTGGTGTTTGGATAA------------------------------------------------------------

--------------------------------------------------------------------------------

--------------------------------------------------------------------------------

--------------------------------------------------------------------------------

--------------------------------------------------------------------------------

--------------------------------------------------------------------------------

--------------------------------------------------------------------------------

--------------------------------------------------------------------------------

--------------------------------------------------------------------------------

--------------------------------------------------------------------------------

--------------------------------------------------------------------------------

--------------------------------------------------------------------------------

--------------------------------------------------------------------------------

--------------------------------------------------------------------------------

----------------------------------------------------------------------------

>TERA02758

ATGGCTGTGGGTGGTGAAGTGGGTGCTACGTCTTCGTTGGCTCCCAAAATTGGTCCACTCGGTCTGTCTCCAAAGAAGGT

TGGAGATGATATTGCCAAGGCAACACAGGACTGGAAAGGTTTGAGAATAACAGTCCAGTTAACAATCCAAAACCGTCAAG

CCAAGGTGTCTGTGGTACCCACAGCCTCGGCTCTTGTTATCAAGGCATTGAAGGAACCACCACGTGACAGAAAGAAGGTG

AAACATGTCAAGCACAGTGGTAATATTTCTATGGATGAAATCTACACCATTGCCCGTACTATGAGGCAAAGGAGTATGGC

AAGGACATTCAAAGGCACTGTCAAAGAAATCTTAGGTACTGCTCAGTCTGTCGGTTGCACTGTTGATGGTCAGCATCCTC

ATGATGTTGCTGACAAGGTAGAAGCTGGAGAATATGATGTACCAGATGAGTAA---------------------------

--------------------------------------------------------------------------------

--------------------------------------------------------------------------------

--------------------------------------------------------------------------------

--------------------------------------------------------------------------------

--------------------------------------------------------------------------------

--------------------------------------------------------------------------------

--------------------------------------------------------------------------------

--------------------------------------------------------------------------------

--------------------------------------------------------------------------------

--------------------------------------------------------------------------------

----------------------------------------------------------------------------

>TERA02762

ATGCCAGGCAGAGCTTGCCAAGGTCTACGTGACGATTTGAAAGAATGTTTGTTGAACAGCAAATGTGTCAGGGAGGACAA

AAAGACACCAAAAGGGTGTTTACTTCTTGGAAATCATCCTTCCGTTCCCCCCGAATGTCAGAATCTTCGTATGGCCTTAT

ATGAATGCAAAAGATCATTAGTTGACATGAGGATGAGGTTCAGAGGGCGTAAAGGTGAATAA------------------

--------------------------------------------------------------------------------

--------------------------------------------------------------------------------

--------------------------------------------------------------------------------

--------------------------------------------------------------------------------

--------------------------------------------------------------------------------

--------------------------------------------------------------------------------

--------------------------------------------------------------------------------

--------------------------------------------------------------------------------

--------------------------------------------------------------------------------

--------------------------------------------------------------------------------

--------------------------------------------------------------------------------

--------------------------------------------------------------------------------

--------------------------------------------------------------------------------

----------------------------------------------------------------------------

>TERA02763

ATGAGGAAGTATTCTGACAAACACGAATGGGTGAAGCTTGATGGCAAAATAGGGACAGTTGGAATCAGTCATTATGCACA

GGAAGCTTTAGGAGATATTGTTTTTGCTGAGTTGCCAGAAGTCGGATCTGAATTTGTCAAAGATGATGAATGTGGTACGT

TAGAGAGTGTAAAGGCAGCCAGTGAGCTCTATACCCCTGTCTCAGGATAA------------------------------

--------------------------------------------------------------------------------

--------------------------------------------------------------------------------

--------------------------------------------------------------------------------

--------------------------------------------------------------------------------

--------------------------------------------------------------------------------

--------------------------------------------------------------------------------

--------------------------------------------------------------------------------

--------------------------------------------------------------------------------

--------------------------------------------------------------------------------

--------------------------------------------------------------------------------

--------------------------------------------------------------------------------

--------------------------------------------------------------------------------

--------------------------------------------------------------------------------

----------------------------------------------------------------------------

>TERA02793

ATGGACAACATTGCTGCTGTTCGTGGTGATGTCTCCAACCACGCCATGAACATCTTCGTGGAGTTCTTCAAGAAGTTTCC

CCAGCACCAGAACGTGTTCGCTGATTACAAGGGCAAGGACCCTGACAGCCTGAAGTCGATGGCTAAGTTCAAGACCCACA

CCACCAAGTAA---------------------------------------------------------------------

--------------------------------------------------------------------------------

--------------------------------------------------------------------------------

--------------------------------------------------------------------------------

--------------------------------------------------------------------------------

--------------------------------------------------------------------------------

--------------------------------------------------------------------------------

--------------------------------------------------------------------------------

--------------------------------------------------------------------------------

--------------------------------------------------------------------------------

--------------------------------------------------------------------------------

--------------------------------------------------------------------------------

--------------------------------------------------------------------------------

--------------------------------------------------------------------------------

----------------------------------------------------------------------------

>TERA02806

ATGGGTGGCATCTATTCAGATTTAACGAAGCCAGATGTCACACCTAAATGGCGCGAGGATCCGACATTTGACCCGAATTA

TGGATTCCCAGCTGGCAGGAAGGAACGAAAATGCATAGCCACAGAAGAAGAGATGATAGCTGCAGGTATTCCAGCTCACC

AACGTGACTACTGTGCACATTTGCTGATAGAATTCTACAAATGCAGAAAAGAAAAGTTTCCCTGGGTTGTCAGCTGTAAA

CACATAAAGCATGACTGGGAAAAATGCGAGTATGATGACTGGATCCTACGAATGAAAGAATATGAGAGAGAAAGAAGATT

GTTAGAACGAGCAAACAGGAAAGCTTAA----------------------------------------------------

--------------------------------------------------------------------------------

--------------------------------------------------------------------------------

--------------------------------------------------------------------------------

--------------------------------------------------------------------------------

--------------------------------------------------------------------------------

--------------------------------------------------------------------------------

--------------------------------------------------------------------------------

--------------------------------------------------------------------------------

--------------------------------------------------------------------------------

--------------------------------------------------------------------------------

--------------------------------------------------------------------------------

----------------------------------------------------------------------------

>TERA02816

ATGGCCAGGAAACATGGTAACTTCTATGTGGCTCCAGAGGCCAAGGTTGCCTTTGTAATCAGGATCAGAGGCATCAATGG

CCTTCATCCAAGACCACGTAAAGTGCTGAAGCTATTCCGTCTGCTTCAGATTAACAATGGCACCTTTGTTAGATTAAACA

AAGCCACACTGCACATGCTGAGGATAGCTGACCCATATGTCACATGGGGGTATCCAAATCTGAAGAGTGTTCGTGAACTG

ATTTACAAACGAGGCTATGGAAGGATTGGTGGCCGTCGGATACCTCTGACTGACAATGAGCTTATTGAGAGAAGTCTCCG

TCGATATGGTATCATCTGTATTGAGGACTTGATCCATGAAATCTACACATGTGGGCCACATTTCAAGGAAGCCAACAGGT

TCCTGTGGACTTTCAAACTGAACACTCCAACAGGAGGATGGGTGAAGAAGAACAACCACTTTGTTGATGGTGGCGACTTT

GGCAACAGAGAAGACAAAATCAATGCACTGTTGAGGAGGATGATATAA--------------------------------

--------------------------------------------------------------------------------

--------------------------------------------------------------------------------

--------------------------------------------------------------------------------

--------------------------------------------------------------------------------

--------------------------------------------------------------------------------

--------------------------------------------------------------------------------

--------------------------------------------------------------------------------

--------------------------------------------------------------------------------

--------------------------------------------------------------------------------

----------------------------------------------------------------------------

>TERA02821

ATGTCACTTGTTGGTCGACCGTGGTGGGAAAAGGACATCATGAAGAAGCTTGGCCTTTGTGATCGGGGTTATTACCCTAC

AGTCCACAAGAATACACCTTCAGTCAATGCCATGCTTAGGGAAGTGAAACACCTGGTGGTGATCAAGCCATTGACCTTTC

CTCATGGTATGCCAGAAGATGAATCTGACTTTGACCACATCCACATTAAGCCAACTGGTGAAGCAATTGTAAAGTAA---

--------------------------------------------------------------------------------

--------------------------------------------------------------------------------

--------------------------------------------------------------------------------

--------------------------------------------------------------------------------

--------------------------------------------------------------------------------

--------------------------------------------------------------------------------

--------------------------------------------------------------------------------

--------------------------------------------------------------------------------

--------------------------------------------------------------------------------

--------------------------------------------------------------------------------

--------------------------------------------------------------------------------

--------------------------------------------------------------------------------

--------------------------------------------------------------------------------

----------------------------------------------------------------------------

>TERA02826

ATGTCTGACGAGGCGGATTGGAAATCCCTGTTTGATCAATGGGATTTGGATGGCAATGGCACGGTTAGACTGGCTGAACT

GGTGGAATGGTTTAAGCAAAACAAGGGATTGACTCACGACCAGGTTAAGGACTTCCTCTGTAAAATCTGGCCCCAGATGG

ATTTGGACGGCGACAACAAGTTGACTTGGGAGGAGTTTAAAGAAGCCATGAAGCAGGCTTAA------------------

--------------------------------------------------------------------------------

--------------------------------------------------------------------------------

--------------------------------------------------------------------------------

--------------------------------------------------------------------------------

--------------------------------------------------------------------------------

--------------------------------------------------------------------------------

--------------------------------------------------------------------------------

--------------------------------------------------------------------------------

--------------------------------------------------------------------------------

--------------------------------------------------------------------------------

--------------------------------------------------------------------------------

--------------------------------------------------------------------------------

--------------------------------------------------------------------------------

----------------------------------------------------------------------------

>TERA02842

ATGGAACAGTCAAAGGCTTATATTGCCAAGTTGCAGAAAATGGTTGATGAAAGTAAACAGTTGTTGGCTAACATCAATTC

TGTACCTCCATATGAAGAGATGACACACCAGATGTACTGCGACTACTTCCCAGAACAGGCTCGTAACCCAGAGAAGAGGC

CAACATTCTGGCCTCATACCAACTCTCATCAACCAGAGAATGATCCACATACAATCAACTAA------------------

--------------------------------------------------------------------------------

--------------------------------------------------------------------------------

--------------------------------------------------------------------------------

--------------------------------------------------------------------------------

--------------------------------------------------------------------------------

--------------------------------------------------------------------------------

--------------------------------------------------------------------------------

--------------------------------------------------------------------------------

--------------------------------------------------------------------------------

--------------------------------------------------------------------------------

--------------------------------------------------------------------------------

--------------------------------------------------------------------------------

--------------------------------------------------------------------------------

----------------------------------------------------------------------------

>TERA02850

ATGAGTAAGATCAACCGTGATTCACTTCGCGAGAACGTTGACTCCTTACTGAAATACAGTAAAGAGGAGAAGAAGAGGAA

GTTCACAGAGAGCGTTGAACTTCAGATATCACTGAAGAACTATGATCCTCAAAAAGACAAGCGTTTTGCTGGCACTGTCA

AGTTGAAGTACATCCCACGTCCAAAGATGAAGGTCTGTGTTTTGGGTGACCAGCAGCATTGTGATGAAGCTAAAGCCAAT

GACATCCCATGTATGGATGTAGAGGCCCTGAAGAAGCTTAACAAGAACAAGAAACTTGTGAAAAAGCTGGCCAAGCGATA

TGATGCTTTTATCGCCTCGGACAGTTTGATCAAACAGATTCCTCGTATTCTTGGTCCTGGCTTGAACAAGGCTGGTAAAT

TTCCAACCATGATCACTCATTCTGACTCCATGGTTAGCAAGGTTAATGAAGTGAAGGCCACCATCAGATTCCAGATGAAG

AAGGTGTTGTGTCTTGCTGTGGCTGTTGGTCATGTCAACATGAGCACAGAGGAGTTGGTTTCCAACATCATGATGGCCAT

CAACTTCCTCGTCTCGCTGCTGAAGAAGAACTGGCAGAATGTACGAGCTCTGTACATCAAGTCCACCATGTAA-------

--------------------------------------------------------------------------------

--------------------------------------------------------------------------------

--------------------------------------------------------------------------------

--------------------------------------------------------------------------------

--------------------------------------------------------------------------------

--------------------------------------------------------------------------------

--------------------------------------------------------------------------------

--------------------------------------------------------------------------------

----------------------------------------------------------------------------

>TERA02851

ATGGAACATAGTACCGGGACAACTATCATGGCTGTTGAATACAACGAGGGTGTAGTGATTGCTGCAGATTCTAGAACCAC

TACAGGAGCTTATATAGCAAATCGTGTAACAAATAAGCTGACTAAGATAACTGATAGAATCTACTGCTGTCGATCAGGAT

CTGCTGCAGACACACAAGCCATAGCTGACGTGGTTGCATATAATCTTGGTTTTCACAAAATGCAAATGGAAGAAGAACCT

TTGGTGAAAACTGCAGCTACAGTTTTTCAAGATATGTGCTACAATTACAGAGATTCATTATCAGCTGGAATTATATGTGC

TGGTTGGGATCACAAATTGGGGGGACAGGTATATTCCATACCTCTGGGAGGCATGTGTGTGAGACAGCCATGTACAATAG

GTGGTTCAGGCAGTACCTATATTTATGGTTATGTTGATTCAACCTTCAAGGAACAGATGACTAAGGAAGAATGCCTGAAG

TTTTGTGCTAATGCTGTGGCATTAGCAATGAGTAGGGATGGATCCAGTGGTGGAGTTATTAGAATGGCATCAATCACCAA

GGATGGAGTTGAGAGACTTACTATTCTTCATGATGACCTGCCCAAGTTCTATGAATAA----------------------

--------------------------------------------------------------------------------

--------------------------------------------------------------------------------

--------------------------------------------------------------------------------

--------------------------------------------------------------------------------

--------------------------------------------------------------------------------

--------------------------------------------------------------------------------

--------------------------------------------------------------------------------

--------------------------------------------------------------------------------

----------------------------------------------------------------------------

>TERA02852

ATGAAATTCGTTGCTGGCTTGAGCACTGGTATTGTTTTCTCTGTCCTCTTTTTAAAACGACGACCGTGGCCTGTTGCCTT

TGGAGCAGGTCTTGGCCTTGGCATGGCTTACTCTAATTGTCAGCATGACTTCATGAATCCAAACTATTTCCATGGCTAA-

--------------------------------------------------------------------------------

--------------------------------------------------------------------------------

--------------------------------------------------------------------------------

--------------------------------------------------------------------------------

--------------------------------------------------------------------------------

--------------------------------------------------------------------------------

--------------------------------------------------------------------------------

--------------------------------------------------------------------------------

--------------------------------------------------------------------------------

--------------------------------------------------------------------------------

--------------------------------------------------------------------------------

--------------------------------------------------------------------------------

--------------------------------------------------------------------------------

--------------------------------------------------------------------------------

----------------------------------------------------------------------------

>TERA02854

ATGCTCTTTCTCGCAATGGCTCCCAAGAAGAAGACTGTGTCTCAGAAAAAGGTAGTGACACCTGGCGAGGTGCCGAAGCC

TCTGAAGACCAAGGACACTGCTCCTCCTGCTAAGGCCAAGGAGAAGGCATTGAAAGCCAGGAAGGCCGTCCGTCTTGGTG

TCCATGAAAAGCGTCGGAAGAAGATCCGCACCAGTGTGCACTTCCACCGACCAAAGACGCTGCGCTTGCCAAGGCAGCCA

AAGTATCCTCGAAAAAGTACACCAAGGAGACCTAGGCTAGACAAGTACAAGATTGTTAAGTATCCTTTGACCACTGAGTC

TGCAATGAAAAAGATTGAAGACAACAACACCTTGGTTTTCATTGTTGATAAACGAGCTAACAAACCACAGATCAAAATAG

CAGTGAAGCAGCTTTATGATATTGATGTATCTAAAGTGAACACATTGATACGACCTGATGGTGAAAAGAAAGCCTACGTG

AGACTAGCCCCAGATTTTGATGCTCTTGATGTAGCCAGCAAGATTGGCATCATCTAA-----------------------

--------------------------------------------------------------------------------

--------------------------------------------------------------------------------

--------------------------------------------------------------------------------

--------------------------------------------------------------------------------

--------------------------------------------------------------------------------

--------------------------------------------------------------------------------

--------------------------------------------------------------------------------

--------------------------------------------------------------------------------

--------------------------------------------------------------------------------

----------------------------------------------------------------------------

>TERA02867

ATGAAGATGACTCGCTACAGTTTAGATCCAGAAAATGCTACGAAATCTGCTAAGGCTAGAGGGTCTCATCTCCGAGTACA

TTTCAAGAACACAAGAGAGACTGCTCAGACCATAAAGCATATGCATCTGCGACGTGCTGTCAGCTTTCTGAAGAATGTAA

AAGCTCACAAGGAATGTGTTCCATTCCGCAGATACAATGGTGGAGTCGGTCGCTGTGCCCAGGCAAAGAACTGGAAGACT

ACACAAGGCCGTTGGCCACAGAAGAGTGCTGAATTTCTTCTTCAGCTGCTTAAGAATGCAGAAAGCAATGCTGAATTCAA

GGGTTTAGATACTGATCATCTAGTAATTGAACACATCCAAGTGAATAGGGCTCCAAAAATGAGAAGACGAACATACAGAG

CTCATGGAAGAATTAACCCATACATGAGTAGTCCATGCCACGTTGAAGTTATCCTTGCTTAA------------------

--------------------------------------------------------------------------------

--------------------------------------------------------------------------------

--------------------------------------------------------------------------------

--------------------------------------------------------------------------------

--------------------------------------------------------------------------------

--------------------------------------------------------------------------------

--------------------------------------------------------------------------------

--------------------------------------------------------------------------------

--------------------------------------------------------------------------------

--------------------------------------------------------------------------------

----------------------------------------------------------------------------

>TERA02871

ATGTTAAGTGTTTTAGCGTTTGCTTGCCTGGTTCAATCATCTGATGATGATGAAGCTAGACTGCTAGTGTCCAAGAACAT

CCTGAACAACTTTCTTGTGGAAGGCAAAGACCTTACCATAGAATATAATATTTATAATGTTGGAGGAAGTTCTGCACTAG

ATGTTCATCTCCATGATGGGAGCTTCATGCCTGGTGACTTTGAAAATGTTGCTGGCTCATTGGATGTTAAGTGGGAGCGA

TTGGCACCTGGAAGCAATGTCTCCCATGTTATTGTGCTTCGTCCTCTGAAACCTGGTTACTACAACTTCTCATCTGCTGA

GGTGACATAA----------------------------------------------------------------------

--------------------------------------------------------------------------------

--------------------------------------------------------------------------------

--------------------------------------------------------------------------------

--------------------------------------------------------------------------------

--------------------------------------------------------------------------------

--------------------------------------------------------------------------------

--------------------------------------------------------------------------------

--------------------------------------------------------------------------------

--------------------------------------------------------------------------------

--------------------------------------------------------------------------------

--------------------------------------------------------------------------------

----------------------------------------------------------------------------

>TERA02878

ATGATAAAGCCATCAGCCTGCTTTATATCAACGTCAAAAAAGAACAAAGATGCAATGGTTGTCGAGAAGACTATTTCCAA

ACCAGATGTTGAGTCAAACCTCGATGCCAAGGAAGAGAACTGGATTTCATATGGCTACAGTCTGGTAGACAGGGAGGATG

ATGAGTTGGCCCATCACTTGATAACCTTTTTCTCTGTTACACTGTGCTTGGTGGGATAA---------------------

--------------------------------------------------------------------------------

--------------------------------------------------------------------------------

--------------------------------------------------------------------------------

--------------------------------------------------------------------------------

--------------------------------------------------------------------------------

--------------------------------------------------------------------------------

--------------------------------------------------------------------------------

--------------------------------------------------------------------------------

--------------------------------------------------------------------------------

--------------------------------------------------------------------------------

--------------------------------------------------------------------------------

--------------------------------------------------------------------------------

--------------------------------------------------------------------------------

----------------------------------------------------------------------------

>TERA02887

ATGCAGATCTTCGTGAAGACGTTGACGGGTAAAACAATTACCCTCGAGGTAGAACCCTCGGATACCATAGAGCACGTGAA

GAGTAAAATCCAGGACAAGGAAGGTATTCCACCTGACCAGCAGCGTCTGATCTTTGCTGGGAAACAGCTCGAAGATGGAC

GTACGCTTAGTGACTACAACATCCAGAAGGAATCGACCCTTCATTTGGTCCTTCGTCTCAGAGGTGGTATAATCGAGCCT

TCCCTGCGTATGCTGGCTCAGAAGTACAACTGTGACAAGATGATTTGTCGCAAGTGTTATGCTCGTCTGCACCCGAGAGC

TACAAACTGCCGCAAGAGGAAATGTGGCCACACAAGCAACATCCGCCCGAAGAAGAAGTTAAAGTAA-------------

--------------------------------------------------------------------------------

--------------------------------------------------------------------------------

--------------------------------------------------------------------------------

--------------------------------------------------------------------------------

--------------------------------------------------------------------------------

--------------------------------------------------------------------------------

--------------------------------------------------------------------------------

--------------------------------------------------------------------------------

--------------------------------------------------------------------------------

--------------------------------------------------------------------------------

--------------------------------------------------------------------------------

----------------------------------------------------------------------------

>TERA02894

ATGAGTAAGGCACATCCACCAGAGTTAAAAAAGTATATGGACAAAAAGTTGCAGCTTCGTCTCAATGGCAGTCGCACAGT

GACTGGAGTCCTGAGAGGCTTTGATCCATTCATGAACATTGTCATAGACGAGGCTATCGAGGAAACGAAGGCGGGCCAAA

AGAACAGTATTGGCATGGTGGTGATCCGTGGTAACAGCATTGTGTTGTTAGAAGCTCTGGAGAGGATCGCCTAA------

--------------------------------------------------------------------------------

--------------------------------------------------------------------------------

--------------------------------------------------------------------------------

--------------------------------------------------------------------------------

--------------------------------------------------------------------------------

--------------------------------------------------------------------------------

--------------------------------------------------------------------------------

--------------------------------------------------------------------------------

--------------------------------------------------------------------------------

--------------------------------------------------------------------------------

--------------------------------------------------------------------------------

--------------------------------------------------------------------------------

--------------------------------------------------------------------------------

----------------------------------------------------------------------------

>TERA02899

ATGGTTGAGTCACTACAGCATAATGTCCAGCGTCTGAAGGAATATCGCAGCAAATTGATCATCTTCCCAAGGAAGCGCGG

CAAACCAAAGAAGGGAGATGCCACTGAGGAAGAGATGAAGATGGCAAAACAGGTCCTAGGAACTGTCATGCCAATCAAGG

AAGCAAGGCCATTCAAGAAGGAGAGGCCTCGTCCCATTACAAGGAAAGAAAAGAAGTTCAGTGCTTAA------------

--------------------------------------------------------------------------------

--------------------------------------------------------------------------------

--------------------------------------------------------------------------------

--------------------------------------------------------------------------------

--------------------------------------------------------------------------------

--------------------------------------------------------------------------------

--------------------------------------------------------------------------------

--------------------------------------------------------------------------------

--------------------------------------------------------------------------------

--------------------------------------------------------------------------------

--------------------------------------------------------------------------------

--------------------------------------------------------------------------------

--------------------------------------------------------------------------------

----------------------------------------------------------------------------

>TERA02905

ATGCGCTTTTTCCGTTTGCACAATGGTATGTGGATACGCACGAGAGCTGGCCGACACAAGAAGCTGTGGAAGAAGAAGGG

TGTCAGGGTGGCACACCTGCAGCATCATGTCTTCTGCACACAGCGCCAGTGCTGGATGCTGGATCGAATGGTCAACAAAT

ACTACAAGATACCCAAGTATTATGCCAATGACCCCTATGCATAA------------------------------------

--------------------------------------------------------------------------------

--------------------------------------------------------------------------------

--------------------------------------------------------------------------------

--------------------------------------------------------------------------------

--------------------------------------------------------------------------------

--------------------------------------------------------------------------------

--------------------------------------------------------------------------------

--------------------------------------------------------------------------------

--------------------------------------------------------------------------------

--------------------------------------------------------------------------------

--------------------------------------------------------------------------------

--------------------------------------------------------------------------------

--------------------------------------------------------------------------------

----------------------------------------------------------------------------

>TERA02910

ATGGAGCAGAAGAAGGCTCAGCTGGTCATCATAGCACATGATGTTGACCCTGTTGAGTTAGTTTTGTTCATGCCTGCTCT

GTGCCGTAAGATGAACGTCCCTTATTGTATCGTGAAAGGAAAGGCTCGACTTGGGCGTGTCGTACGTCGGAAGACGGCCA

CTTGTCTGGCTTTGACACATGTAAACCCCGAGGACAAATCATCATTAAACAAGTTGGTTGAAGCTGTTAAGACGAACTTC

AATGAGAGATACGATGAGATTCGTCGTCATTGGGGAGGTGGTATCATGGGTGCCAAGTCGCAGGCTCGTATTGCCAAGCT

GGAGAAGATCAAAGCCAGGGAGCTGGCTCAAAAGATGTAA----------------------------------------

--------------------------------------------------------------------------------

--------------------------------------------------------------------------------

--------------------------------------------------------------------------------

--------------------------------------------------------------------------------

--------------------------------------------------------------------------------

--------------------------------------------------------------------------------

--------------------------------------------------------------------------------

--------------------------------------------------------------------------------

--------------------------------------------------------------------------------

--------------------------------------------------------------------------------

--------------------------------------------------------------------------------

----------------------------------------------------------------------------

>TERA02921

ATGCCTCGTTATGTGAAGAACATTGGTCTTGGCTTTAAAACGCCGAGGGAGGCATATGATGGCACCTACATTGACAAGAA

ATGTCCATTCACTGGACGAATAAGCATCCGTGGCCGTATCCTGACAGGTGTTGTGATGAAGATGAAGATGCAGCGAACGA

TTGTCATCCGACGAGACTACCTGCACTATGTCAGAAAGTACAATCGATTTGAAAAACGGCACAAGAATGTATCAGTCCAC

TGCAGTCCTTGCTTCAGAGATATTGCTGTTGGTGACATCGTTACTGTAGGTGAATGCCGGCCTCTCAGCAAGACTGTCAG

ATTTAATGTTGTTAAAGTTAACAAAAGCAGTAAAAGCAAAAAAGGATAA-------------------------------

--------------------------------------------------------------------------------

--------------------------------------------------------------------------------

--------------------------------------------------------------------------------

--------------------------------------------------------------------------------

--------------------------------------------------------------------------------

--------------------------------------------------------------------------------

--------------------------------------------------------------------------------

--------------------------------------------------------------------------------

--------------------------------------------------------------------------------

--------------------------------------------------------------------------------

--------------------------------------------------------------------------------

----------------------------------------------------------------------------

>TERA02922

ATGAAGTTCAAAGATCATATGCATTTCTACATAATGCTTGGTGTTATTCCACTGACTATAGTTACAACATGGGCCAATAT

ATTTATTGGAAATGCAGAACTTTCAGAGATACCAGAGGGATATGAACCCAAACACTGGGAGTATTACAAACATCCTGTGT

CACGGTTTATCTCCAAACACCTACACAGTCATCCAGCAAAACAGTATGAAATAAACCTGCACTACATCAATGAGGAACAG

AAAAAGGCCCAGATGAGAAAACTGGAGAAGAAAGTGAAACAACTGATGCATGAACGTGGAGACTATAAGGGCTGGTACTA

CATGCCATACAACCATGAACGTATCTATGACAGCTATTAA----------------------------------------

--------------------------------------------------------------------------------

--------------------------------------------------------------------------------

--------------------------------------------------------------------------------

--------------------------------------------------------------------------------

--------------------------------------------------------------------------------

--------------------------------------------------------------------------------

--------------------------------------------------------------------------------

--------------------------------------------------------------------------------

--------------------------------------------------------------------------------

--------------------------------------------------------------------------------

--------------------------------------------------------------------------------

----------------------------------------------------------------------------

>TERA02927

ATGGGGGCTCCGGTAACCTACATGCACCTTTGGGAGGACAGCGTCCTGTCGATGGGCGTCTTCATCGTCAAGCACGGGAT

GTCCATCCCGCTGCACGACCACCCGAGCATGTACGGCATCATCAAGGTGCTCCACGGCACCATGCAGGTCAGCTCGTTCA

GCTAA---------------------------------------------------------------------------

--------------------------------------------------------------------------------

--------------------------------------------------------------------------------

--------------------------------------------------------------------------------

--------------------------------------------------------------------------------

--------------------------------------------------------------------------------

--------------------------------------------------------------------------------

--------------------------------------------------------------------------------

--------------------------------------------------------------------------------

--------------------------------------------------------------------------------

--------------------------------------------------------------------------------

--------------------------------------------------------------------------------

--------------------------------------------------------------------------------

--------------------------------------------------------------------------------

----------------------------------------------------------------------------

>TERA02930

ATGGCGCCAAGAAAAGGCCGTGTTCAAAAGGAGGAACAGGTTTCTCTCCCACAGGTAGCTGATGGTGAAAACCTTTTTGG

TGTTGCACACATATATGCCAGTTTCAATGACACATTCGTCCATGTGACAGATCTGTCTGGAAAGGAGACCATTGTCCGTG

TAACTGGTGGCATGAAAGTCAAGGCAGATCGTGATGAAGCTTCCCCCTACGCTGCTATGTTGGCAGCTCAGGATGTAGCT

GAGAGGTGTAAATCCCTTGGCATTAATGCTCTGCACATTAAACTTAGAGCCACTGGTGGAAACAAAACAAAGACCCCAGG

TCCAGGAGCACAGTCTGCCTTACGTGCATTAGCACGATCTGGAATGAAAATTGGTCGTATAGAGGATGTGACACCAATAC

CATCTGACAGCACAAGAAGGAAGGGAGGTCGCCGTGGACGAAGGCTCTAA------------------------------

--------------------------------------------------------------------------------

--------------------------------------------------------------------------------

--------------------------------------------------------------------------------

--------------------------------------------------------------------------------

--------------------------------------------------------------------------------

--------------------------------------------------------------------------------

--------------------------------------------------------------------------------

--------------------------------------------------------------------------------

--------------------------------------------------------------------------------

--------------------------------------------------------------------------------

----------------------------------------------------------------------------

>TERA02943

ATGAAGATGGTTCAGAGACTGACGTTACGAAGGCGTCTCAGCTACAACACCAATTCTAACAGAAGACGGATTGTTAAGAC

ACCAGGTGGTCGTCTTGTCTATCATTACACAAAGAAGCCAGGAACCATCCCAATATGCAAGTCTGGAGGCTGTAGAACAA

AGCTACATGGTATCCAACCATCTCGTCCAATGGAGAGGCGGCGCATGTCGAAGAGATTAAAGACTGTGAACAGGACATAC

GGAGGAGTTCAATGTCACACCTGTGTCAGAGAAAAGATCATTCGTGCTTTTCTGATTGAAGAACAGAAGATTGTGGTCCG

AGTGCTGAAGGCGCAGGCAGCTCAAGCTAAGAAGTCATAA----------------------------------------

--------------------------------------------------------------------------------

--------------------------------------------------------------------------------

--------------------------------------------------------------------------------

--------------------------------------------------------------------------------

--------------------------------------------------------------------------------

--------------------------------------------------------------------------------

--------------------------------------------------------------------------------

--------------------------------------------------------------------------------

--------------------------------------------------------------------------------

--------------------------------------------------------------------------------

--------------------------------------------------------------------------------

----------------------------------------------------------------------------

>TERA02972

ATGGAAGCCATGACACGCCGTGAGAAGGGCAAGAAGAAGTCTGTCACCAACGAGGTGGTGACGCGGGAATACACCATCAA

CATCCACAAACGAATTCATGGAATAGGATTCAAGAAGAGGGCACCACGAGCTATCAAGGCCATCAGACAGTTTGCCTACA

AGCAAATGGGAACTGAAGATGTACGAATAGAAACAAGACTAAACAAACACATTTGGTCAAAGGGAATTAGAAATGTTCCA

TTCCGAGTACGAGTACGATTGGCTCGTAAGAGGAATGAGGATGAGGACTCGCCTCACAAGCTCTACACCTTGGTGTCCTA

TGTACCTGTCACCACATTCAAAGGTACCCAGACAGTAAATGTGGAGAGCAGCGACTAA----------------------

--------------------------------------------------------------------------------

--------------------------------------------------------------------------------

--------------------------------------------------------------------------------

--------------------------------------------------------------------------------

--------------------------------------------------------------------------------

--------------------------------------------------------------------------------

--------------------------------------------------------------------------------

--------------------------------------------------------------------------------

--------------------------------------------------------------------------------

--------------------------------------------------------------------------------

--------------------------------------------------------------------------------

----------------------------------------------------------------------------

>TERA02984

ATGGAAATGGAAATAGCCAACAGAGATGTGGCTCGAGTCATAGGCAGAGGAGGCTCAAAAATCCGTGAGTTGCAGGATGA

GAGCAGTACTAGAATCCAGATTAACAAGGACAGAGACAATGGTGTGACAACAGTGGTTGAAATCCGTGGCAGTGAGGAAG

GTCAACAGAGATAA------------------------------------------------------------------

--------------------------------------------------------------------------------

--------------------------------------------------------------------------------

--------------------------------------------------------------------------------

--------------------------------------------------------------------------------

--------------------------------------------------------------------------------

--------------------------------------------------------------------------------

--------------------------------------------------------------------------------

--------------------------------------------------------------------------------

--------------------------------------------------------------------------------

--------------------------------------------------------------------------------

--------------------------------------------------------------------------------

--------------------------------------------------------------------------------

--------------------------------------------------------------------------------

----------------------------------------------------------------------------

>TERA02986

ATGGGTGAGAAAGGAAAAAGGAAGGGTTTGGGAGGTCTCTCCCCGGAGAAGAAGAAGCTGCTAAAGCAACTGATCATGCA

AAAAGCAGCCGAGGACCTGAAGAAGCAGCAGGAAGCCGAGGCTGAGGCTAAGAGGAAGCTCATCCAGGAGCGCGTCCCCA

AACTGGAGATCGATGGCCTGGATCAAGCCAAGCTGGAGAAGGTTGTACGAGACCTTTACAAAAGGGTGGTGGCCTTGGAG

GAGGAAAAATATGACTGGGAGGTTAAACTTAGGAAACAAGACCAAGAGATGAATGAGCTTAACATCAAGGTCAACGACAT

CAAGGGCAAATTCGTTAAGCCCGTACTGAAGAAGGTGTCGAAGACAGAACAGAAGATGTCTAAGTTTGACAAGGAGAAGC

TGAAGCGCAAGATGGGAGGCTTCCGTGAACAGCTCAAGTCTACGGGGCAGTCTAAGTTTGCCCTGGACGACAAGGAGGAG

GTTAAGAAGCCAGAATGGGGACAGACTTAA--------------------------------------------------

--------------------------------------------------------------------------------

--------------------------------------------------------------------------------

--------------------------------------------------------------------------------

--------------------------------------------------------------------------------

--------------------------------------------------------------------------------

--------------------------------------------------------------------------------

--------------------------------------------------------------------------------

--------------------------------------------------------------------------------

--------------------------------------------------------------------------------

----------------------------------------------------------------------------

>TERA02988

ATGGCAGGTTACAAAGATGACAAGCAAATTGAGGAAACTCAGATACATCGCATTCGTATTACCCTCACCAGCAGAAATGT

AAAATCGTTAGAAAAAGTCTGTGCTGACCTGATCACTGGGGCAAAGGAGAAGAACCTGAAAGTGAAAGGACCTGTTCGCA

TGCCAACCAAGATTCTCCGTATCACAACACGCAAGACACCTTGTGGTGAAGGATCTAAAACATGGGATCGTTTCCAAATG

AGGATCCACAAGCGTCTCATTGACCTGCACAGTCCATCAGAGATCGTGAAGCAGATCACATCCATCAGCATTGAGCCTGG

TGTAGAGGTGGAAGTTACAATTGCTGATTAA-------------------------------------------------

--------------------------------------------------------------------------------

--------------------------------------------------------------------------------

--------------------------------------------------------------------------------

--------------------------------------------------------------------------------

--------------------------------------------------------------------------------

--------------------------------------------------------------------------------

--------------------------------------------------------------------------------

--------------------------------------------------------------------------------

--------------------------------------------------------------------------------

--------------------------------------------------------------------------------

--------------------------------------------------------------------------------

----------------------------------------------------------------------------

>TERA03040

ATGAAATCCACCAACATGGTGAAGGTCAAGGCAAAGGACTTGCGTGGCAAGAAGAAGGATGAGCTTCAGCGGCAGTTGAA

TGAACTCAAACATGAGTTGGCTCAGCTACGTGTGTCCAAGGTTACCGGAGGTAGTGCCTCCAAGCTGTCAAAGATTGTTG

TGGTTCGTAAATCAATTGCCCGAGTCATGACAGTGATGCACCAGACACAGAAGGAAAACCTGCGCAAGTTTTACAAAGGT

AAAAAGCACAAGCCAAAGGACTTGAGGCGAAAGAAGACGAGGGCCATGCGCAAAGCTCTGACAAAGCATGAACTCAGCCT

GAAGACAGCTAAACAGAGGTAA----------------------------------------------------------

--------------------------------------------------------------------------------

--------------------------------------------------------------------------------

--------------------------------------------------------------------------------

--------------------------------------------------------------------------------

--------------------------------------------------------------------------------

--------------------------------------------------------------------------------

--------------------------------------------------------------------------------

--------------------------------------------------------------------------------

--------------------------------------------------------------------------------

--------------------------------------------------------------------------------

--------------------------------------------------------------------------------

----------------------------------------------------------------------------

>TERA03078

ATGCGTTACGTGGCTGCCTACTTGCTTGCTGTTTTGGGTGGTAATTCCAACCCTACAGCTAACGACATCAAACAGATCCT

CAGTAGCGTTGCTGTTGATGTTGATGAGAATTCTCTGAGCAAGGTTATCAAGGAACTCCAAGGAAAAGATATCAAGGAAC

TTATGGCTGAAGGTCACTCAAAGCTAGCCAGTGTGCCAGCCGCTGGTGCTGTTTAA------------------------

--------------------------------------------------------------------------------

--------------------------------------------------------------------------------

--------------------------------------------------------------------------------

--------------------------------------------------------------------------------

--------------------------------------------------------------------------------

--------------------------------------------------------------------------------

--------------------------------------------------------------------------------

--------------------------------------------------------------------------------

--------------------------------------------------------------------------------

--------------------------------------------------------------------------------

--------------------------------------------------------------------------------

--------------------------------------------------------------------------------

--------------------------------------------------------------------------------

----------------------------------------------------------------------------

>TERA03081

ATGAACATGCCTTTAGCACGAGATTTACTTAACCCGAGTCCAGAGGAGGAGAAGAGGAAGTGCAAGTTGAAACGACTTGT

ACAGAGTCCAAACTCTTATTTCATGGATGTCAAATGTCCAGGTTGTTACAAGATCACGACAGTGTTTAGTCACGCCCAGA

CTGTAGTCCTTTGTGTTGGTTGTTCCACAGTCTTGTGCCAGCCAACAGGAGGCAAAGCCAGATTAACAGAAGGTTGTTCA

TTCAGGAGAAAGTAA-----------------------------------------------------------------

--------------------------------------------------------------------------------

--------------------------------------------------------------------------------

--------------------------------------------------------------------------------

--------------------------------------------------------------------------------

--------------------------------------------------------------------------------

--------------------------------------------------------------------------------

--------------------------------------------------------------------------------

--------------------------------------------------------------------------------

--------------------------------------------------------------------------------

--------------------------------------------------------------------------------

--------------------------------------------------------------------------------

--------------------------------------------------------------------------------

----------------------------------------------------------------------------

>TERA03086

ATGAACAAGTCCATCAATCCTGATGAGGCCGTTGCCTATGGTGCAGCTGTCCAGGCTGCCATCCTTCATGGTGACAAATC

GGAGGAGGTCCAGGATCTCCTACTGCTGGACGTGACTCCACTGTCCCTGGGTATCGAGACGGCTGGTGGCGTCATGACTT

CACTGATCAAGCGTAACACGACCATTCCCACCAAGCAGACCCAGACGTTCACCACCTACTCGGACAACCAGCCGGGTGTG

CTGATCCAGGTCTACGAGGGCGAGCGAGCCATGACCAAGGACAACAACCTGCTAGGAAAGTTCGAGCTGACCGGCATCCC

ACCAGCACCTCGTGGCGTGCCGCAGATCGAGGTTACGTTTGACATTGATGCCAATGGCATCCTCAACGTGTCTGCTGTTG

ACAAGAGCACTGGCAGAGAGAACAAGATCACCATCACAAATGACAAGGGTCGTCTGAGCAAGGAAGAGATTGAGCGCATG

GTGAAGGAAGCCGAGAAATACAAGGCAGAGGATGAAAGCCAGAAGGAACGAATCCAAGCCAAGAATGCCCTGGAGAGCTA

TGCCTTCAACATGAAGTCCACTGTGGAGGATGAGAAACTGAAGGACAAGATCAGCGAATCTGACAAGAAGTTGATCACTG

ACAAATGTAACGAGATCTAA------------------------------------------------------------

--------------------------------------------------------------------------------

--------------------------------------------------------------------------------

--------------------------------------------------------------------------------

--------------------------------------------------------------------------------

--------------------------------------------------------------------------------

--------------------------------------------------------------------------------

--------------------------------------------------------------------------------

----------------------------------------------------------------------------

>TERA03090

ATGGAGAAAGCTGGTGGCAAGTGTCTGCCGTGTGTGGTCGACATACGACACGAAGACCAAGTCCAAAAGGCCGTCGATGA

GACGGTCAGGAAATTCGGTGGCATCGATATCTTGATCAACAATGCTAGTGCTATAAGCCTTACGCCTACTGAAGCGACCA

GCATGAAGCGATACGATCTGATGAACAACATCAACGCAAGGGGAACGTTCTTATGCTCGTAA------------------

--------------------------------------------------------------------------------

--------------------------------------------------------------------------------

--------------------------------------------------------------------------------

--------------------------------------------------------------------------------

--------------------------------------------------------------------------------

--------------------------------------------------------------------------------

--------------------------------------------------------------------------------

--------------------------------------------------------------------------------

--------------------------------------------------------------------------------

--------------------------------------------------------------------------------

--------------------------------------------------------------------------------

--------------------------------------------------------------------------------

--------------------------------------------------------------------------------

----------------------------------------------------------------------------

>TERA03095

ATGGGTTCTTTGGCTGATATTGACAGAAAATACATCGAAAAGTACAATCGGCTGCTGAAGGAGAGTCTTGAAAGGGAACG

AAGACTACTGCAGCACTATGAGTGGCGCGGTAACAAAGTGCCACCCTTCAGCATCGAGCCCTTAGCTCATGAACGGGACC

GGTTGTCCGGGTCTGGAATGACCCCGGAGGAAAGAGCGGCCAGACTTCAATGGGTTAAAGACCAAGAACTGGCTCCGTAA

--------------------------------------------------------------------------------

--------------------------------------------------------------------------------

--------------------------------------------------------------------------------

--------------------------------------------------------------------------------

--------------------------------------------------------------------------------

--------------------------------------------------------------------------------

--------------------------------------------------------------------------------

--------------------------------------------------------------------------------

--------------------------------------------------------------------------------

--------------------------------------------------------------------------------

--------------------------------------------------------------------------------

--------------------------------------------------------------------------------

--------------------------------------------------------------------------------

----------------------------------------------------------------------------

>TERA03117

ATGCGTATTGACCATATTGTTGGCAATCAGCCTGATGATGAGATGGTTCCTGTTGCTGATTGGTATGAGAAGACTCTGAT

GTTCCATCGCTTCTGGTCAGTAGATGATAAGCAGCTGCACACCGAGTTCTCAGCTCTTCGTTCGATTGTTGTTGCCAATT

ATGAGGAAACAATTAAAATGCCCATCAATGAACCTGCCCCTGGCAAGCGCAAGAGTCAGATCCAGGAATATGTCGAGTAC

TATGGTTAA-----------------------------------------------------------------------

--------------------------------------------------------------------------------

--------------------------------------------------------------------------------

--------------------------------------------------------------------------------

--------------------------------------------------------------------------------

--------------------------------------------------------------------------------

--------------------------------------------------------------------------------

--------------------------------------------------------------------------------

--------------------------------------------------------------------------------

--------------------------------------------------------------------------------

--------------------------------------------------------------------------------

--------------------------------------------------------------------------------

--------------------------------------------------------------------------------

----------------------------------------------------------------------------

>TERA03134

ATGGCCGAATTTGAGAAGTGGAAGAACCCGACCTCTCCGAACTTCGTCATCACAAGGAAGGATCGTCCTGCAGGTGGAGC

CGGCGGGCAGGAAGAGGGTGTTGAGGGAGGCGGCGAGAAGAAGTCCAAAGAACAGCTCGAGGCAGAGAAGAGAGCCATCC

TCAAGCAGAGGATCCCGCCACTGGAGATCGACGGATTCGATGCCGCCAAGCTGGCAGAAAAGGCCCGAGAACTGCACGCT

CAGATCTACCGTCTGGAAAGCGAGAAGTATGACCTGGAAAGACGCTTCAAGAGTCAACTCAGCGACATGATGGAATTGGC

AGAACGTGCTAGGAATATGAACAAAGTCGGCAAACACGGAAGCATTAGGAGAGTACAACTGAGTGATGGAGAGACTGATA

AATTCCAAGAGCGTTTTGCTGGCTGCCCGGCTAAGATCGTTATGTACAGTCAGTATGAGCGCCAGAAGGACAAGAGATAA

--------------------------------------------------------------------------------

--------------------------------------------------------------------------------

--------------------------------------------------------------------------------

--------------------------------------------------------------------------------

--------------------------------------------------------------------------------

--------------------------------------------------------------------------------

--------------------------------------------------------------------------------

--------------------------------------------------------------------------------

--------------------------------------------------------------------------------

--------------------------------------------------------------------------------

----------------------------------------------------------------------------

>TERA03150

ATGGCACGTACGAAGCAGACTGCCCGTAAGTCAACCGGAGGAAAGGCTCCGCGTAAGCAGCTGGCCACGAAGGCTGCTCG

TAAGAGCGCCCCGTCCACCGGTGGAGTGAAGAAGCCCCATCGTTACAGGCCCGGTACCGTCGCCCTCCGAGAGATCAGGC

GTTACCAGAAGTCGACTGAGCTGCTTATCAGGAAGCTGCCATTCCAGCGACTCGTCCGTGAGATTGCTCAGGACTTCAAG

ACGGATCTGCGTTTCCAGAGTGCTGCTATCGGTGCACTTCAGGAGGCCAGTGAAGCATACCTGGTTGGTCTGTTTGAGGA

CACTAACTTGTGCGCCATCCACGCCAAGCGTGTAACCATCATGCCAAAAGACATCCAGCTGGCACGAAGAATACGTGGAG

AGCGTGCTTAA---------------------------------------------------------------------

--------------------------------------------------------------------------------

--------------------------------------------------------------------------------

--------------------------------------------------------------------------------

--------------------------------------------------------------------------------

--------------------------------------------------------------------------------

--------------------------------------------------------------------------------

--------------------------------------------------------------------------------

--------------------------------------------------------------------------------

--------------------------------------------------------------------------------

--------------------------------------------------------------------------------

----------------------------------------------------------------------------

>TERA03165

ATGACGAGAAAATTTATGACAAACCGTCTTCTATCAAGACGACAAATGGTTGTGGATGTTTTACATCCTGGACGTGCAAC

GGTGCCAAAAACAGAGATCCGTGAGAAGTTGGCCCGCATGTACAAGACCACTTCTGACGTCATATTCTGTTTTGGATTCC

GTACACAATTCGGTGGCGGAAAGACGACTGGCTTCGCTCTGATCTATGATAATCTGGACTATGCCAAGAAATTCGAGCCC

AAATACAGATTAGCAAGGCATGGGCACTTAGAAATTAAAAAGACGGCCAGAAAGCAGAGGAAAGAACGAAAGAATCGACA

AAAGAAGGTCAGAGGTACAAAGAAGGCTAAGGTTGGACAGGCATCAAAGAAGGGTCGATAA-------------------

--------------------------------------------------------------------------------

--------------------------------------------------------------------------------

--------------------------------------------------------------------------------

--------------------------------------------------------------------------------

--------------------------------------------------------------------------------

--------------------------------------------------------------------------------

--------------------------------------------------------------------------------

--------------------------------------------------------------------------------

--------------------------------------------------------------------------------

--------------------------------------------------------------------------------

--------------------------------------------------------------------------------

----------------------------------------------------------------------------

>TERA03166

ATGCTGGTCCATGCTGAAGAGGATGCCTTGGAAGAAGATGATGATGAAGAAGATGGAGATGCCACTGTTGAAACAGATGA

TGGCATGGATCCAGGATCTGAGGCTGTTCCGGTAGAAACTGAAACAGCGGAAGAAGAAGAGGAGGAAGAGGATAAGCCCC

TGCAACCATCTCCCAATGGGGACACATACATCCTCTTCACAAAGCCCAATACACCTACTGATCTGCCAGCTGGTAAGGAA

GTCAGGTTTCTGGTTGGCTTCACCAACAAGGGATCCAGTGACTTCATCATTGAATCCATGGATGCTGCTTTCCGTTATCC

CCAGGATTACAGCTTCTACATTCAGAATTTCTAA----------------------------------------------

--------------------------------------------------------------------------------

--------------------------------------------------------------------------------

--------------------------------------------------------------------------------

--------------------------------------------------------------------------------

--------------------------------------------------------------------------------

--------------------------------------------------------------------------------

--------------------------------------------------------------------------------

--------------------------------------------------------------------------------

--------------------------------------------------------------------------------

--------------------------------------------------------------------------------

--------------------------------------------------------------------------------

----------------------------------------------------------------------------

>TERA03177

ATGGTGCCCATCTACGAGGGTTACGCCCTGCCCCATGCCATCCTCCGTCTGGACTTGGCCGGCCGTGATCTGACCGACTA

CCTGATGAAGATCCTGACCGAGCGTGGCTACAGCTTCACCACCACGGCCGAGCGCGAGATCGTCCGTGACATCAAGGAGA

AGCTCTGCTACGTCGCCCTCGACTTCGAGCAGGAGATGGCCACCGCCGCCTCCTCTTCCTCCCTGGAGAAGTCCTACGAG

CTGCCCGACGGTCAGGTGATCACCATCGGAAACGAGCGCTTCAGGTGCCCAGAGGCTCTCTTCCAGCCATCCTTCTTGGG

TATGGAGTCCGCCGGCATCCACGAGACGACCTACAACAGCATCATGAAGTGCGATGTCGACATCCGTAAGGATCTGTACG

CCAACACCGTCCTGTCCGGTGGCACGACCATGTACCCGGGTATCGCCGACCGCATGCAGAAGGAGATCACGGCCCTGGCC

CCGAGCACCATGAAGATCAAGATCATCGCTCCACCAGAGCGCAAGTACTCCGTCTGGATCGGTGGCTCCATCCTGGCCTC

GCTGTCCACCTTCCAGCAGATGTGGATCAGCAAGCAGGAGTACGACGAGTCTGGACCATCCATCGTTCACAGGAAGTGCT

TCTAA---------------------------------------------------------------------------

--------------------------------------------------------------------------------

--------------------------------------------------------------------------------

--------------------------------------------------------------------------------

--------------------------------------------------------------------------------

--------------------------------------------------------------------------------

--------------------------------------------------------------------------------

--------------------------------------------------------------------------------

----------------------------------------------------------------------------

>TERA03228

ATGGAGACGATGAAGAAGGCGATAAACGACCCGAGCCTGAAGCCGACGATCGAGGGCCCGCTGCCGCTGTTCTTCCACGC

CGTCGACTCGAACAACGACGGCTTCATCTCGGAGGACGAGTACTGCGAGTTCTTCAAGATCCTCGGCCTTGACCCGAGCC

TGGCACCGCCCTCTTTCAAGGCCATCGACACGAACAACGACGGCCTGCTCAGCGAGGACGAGTTCCGCGTCGCCGGCACC

GACTTCTTCCTCAGCGACGACCAGAACTGCCCGACGAAATTCTTCTGGGGGCCCCTCGTCTAA-----------------

--------------------------------------------------------------------------------

--------------------------------------------------------------------------------

--------------------------------------------------------------------------------

--------------------------------------------------------------------------------

--------------------------------------------------------------------------------

--------------------------------------------------------------------------------

--------------------------------------------------------------------------------

--------------------------------------------------------------------------------

--------------------------------------------------------------------------------

--------------------------------------------------------------------------------

--------------------------------------------------------------------------------

--------------------------------------------------------------------------------

----------------------------------------------------------------------------

>TERA03233

ATGAAGTCTCTGAATCCAAAAGCTGAAGTGGCAAGGGCTGCCCAGGCGTTGGGACTAAATATCAGCGCCGCTAAGGGACT

CCAGGATGTCCTACGATCTAATCTTGGCCCAAAAGGCACATTGAAGATGTTGGTGTCTGGTTCCGGTGACATTAAACTGA

CTAAGGATGGGAATGTACTCCTCCATGAGATGCAAATTCAGCATCCTACAGCATCCTTGATTGCTCGAGTGGCAACAGCC

CAGGATGACATCACTGGAGATGGTACTACGTCTAATGTGCTAATCATTGGGGAACTGCTGAAACAGGCTGACCTCTATAT

ATCTGAGGGTCTACATCCTCGACTGGTCACAGAAGGTTTTGAAATGGCCAAGAACAAAGCATTAGAGGTACTGGAGCAAA

TAAAGGTTAAATGTGACATAGACAGAGATTTACTTACCAATGTGGCCAGGACATCCCTGAGAACAAAGGTTCATCCTGAA

TTAGCTGATCACCTAACTGAGCATGTAGTTGATGCTTAA-----------------------------------------

--------------------------------------------------------------------------------

--------------------------------------------------------------------------------

--------------------------------------------------------------------------------

--------------------------------------------------------------------------------

--------------------------------------------------------------------------------

--------------------------------------------------------------------------------

--------------------------------------------------------------------------------

--------------------------------------------------------------------------------

--------------------------------------------------------------------------------

----------------------------------------------------------------------------

>TERA03238

ATGTTTACTGGATTCAAGAGAGGCCTTCGCAATCAGCATGAGAACACTGCTTTGTTGAAGATTGAAGGTGTAAACAGACG

GAAAGAAACTGAATTCTACTTAGGGAAGAGATGTGCCTATGTGTATAAGGCAAAAAACAAGACGACATGTCCAGGCTACA

AAAGGCCAACCAAACTGCGTGTAATCTGGGGCAAGGTGACGCGCCCACATGGTAACAGTGGTGCAGTCAGAGCAAAGTTC

AGGAAGAACCTTCCACCAGCTGCCATGGGAAAGCGTATCAGAGTGATGCTCTATCCATCAAGAATATAA-----------

--------------------------------------------------------------------------------

--------------------------------------------------------------------------------

--------------------------------------------------------------------------------

--------------------------------------------------------------------------------

--------------------------------------------------------------------------------

--------------------------------------------------------------------------------

--------------------------------------------------------------------------------

--------------------------------------------------------------------------------

--------------------------------------------------------------------------------

--------------------------------------------------------------------------------

--------------------------------------------------------------------------------

--------------------------------------------------------------------------------

----------------------------------------------------------------------------

>TERA03249

ATGATCATGTCTGGTTTAGTGAAAGCTAAGAAGTATGACTGGAAGGACAGCAATCTGGCACTGTTTGGCTCCGACACCGA

GAAACAAGTAAAAAAGGAGTCTGCTGAAGCTGAACCAGCTTGGCAAGGAGTGGGACAGGAGGTTGGCCTTAAGATCTGGA

GAATTGTTAAATTTGAGGTCACCGATTGGCCAGAAGAGGACTATGGTGACTTCTTTAGTGGAGATTCATACATCATTCTG

AATACATACAAGCCAAATGAGGACAGTGAGGAGCTAGCTTATGATGTACACTTCTGGATTGGCAAGTACAGCACTCAGGA

CGAGTACGGAACTGTGGCTTATAAAACTGTTGAGCTGGACACGTACCTCGATGACAAAGCCGTCCAGCACAGAGAAGTGC

AGGGCTTTGAATCTGACCTTTTCAAGTCTTACTTTGATCCGCTGACCATCATGGAAGGCGGGGCTGAGTCTGGCTTCAGA

CACGTGACACCAGAAGAATACAAGAAACGTCTGTAA--------------------------------------------

--------------------------------------------------------------------------------

--------------------------------------------------------------------------------

--------------------------------------------------------------------------------

--------------------------------------------------------------------------------

--------------------------------------------------------------------------------

--------------------------------------------------------------------------------

--------------------------------------------------------------------------------

--------------------------------------------------------------------------------

--------------------------------------------------------------------------------

----------------------------------------------------------------------------

>TERA03260

ATGGACCAGAAAGGAGGAAAGGCAGCTCCTGCCAAGAAGAAGGAGGGTTCTTCTGGTGGTAAAGCCAAGAAGAAGAAGTG

GTCCAAGGGAAAAGTTCGTGACAAGCTGAATAACTTGGTACTCTTTGATAAAGCAACTTATGACAAGCTTTACAAAGAGG

TGCCAAACTACAAGCTGATCACACCATCAGTCGTCTCAGAGAGATTAAAGATCCGTGGATCCCTTGCCAGAGCTGGTCTG

CAAGAACTTCTCAGTAAAGGTTTGATCAAATTGGTTGAGAAACATCATGCCCAAGTGATCTACACACGGAATCCATAA--

--------------------------------------------------------------------------------

--------------------------------------------------------------------------------

--------------------------------------------------------------------------------

--------------------------------------------------------------------------------

--------------------------------------------------------------------------------

--------------------------------------------------------------------------------

--------------------------------------------------------------------------------

--------------------------------------------------------------------------------

--------------------------------------------------------------------------------

--------------------------------------------------------------------------------

--------------------------------------------------------------------------------

--------------------------------------------------------------------------------

----------------------------------------------------------------------------

>TERA03275

ATGGGCAAAGTTCATGGATCACTAGCTCGTGCTGGAAAGGTCAAGGGCCAGACCCCCAAGGTGGAGAAGCAGGAAAAGAA

AAAGAAGAAGACTGGACGTGCTAAGCGTCGTATCCAGTACAACAGACGATTTGTTAATGTTGTGCAGACCTTTGGCAGAA

GGAAGTAA------------------------------------------------------------------------

--------------------------------------------------------------------------------

--------------------------------------------------------------------------------

--------------------------------------------------------------------------------

--------------------------------------------------------------------------------

--------------------------------------------------------------------------------

--------------------------------------------------------------------------------

--------------------------------------------------------------------------------

--------------------------------------------------------------------------------

--------------------------------------------------------------------------------

--------------------------------------------------------------------------------

--------------------------------------------------------------------------------

--------------------------------------------------------------------------------

--------------------------------------------------------------------------------

----------------------------------------------------------------------------

>TERA03278

ATGTCTGACGCGGAAGGAGATGATGTTCCCTCTGCCAGAGGGGGAGGCGGTATGGATATAAATGCCGCTATTCAGGAAGT

CCTCAAGACATCTTTGATACACGACGGATTAGCTCGTGGCCTTCACGAATGCGCCAAAGCTCTGGACAAACGCCAGGCTC

ATCTGTGTATCCTTGCCAACAACTGCGACGAGCCGATGTACGTCAAGTTGGTCGAAGCCTTGTGTGCCGAGCACGGCATT

AACCTGATTAAGGTTGATGATAACAAGAAGCTGGGTGAATGGGCTGGTCTTTGCAAGATAGACAAGGAAGGCAAGGCCCG

GAAGGTTGTTGGTTGCAGTTGTGTTGTTGTTAAGGATTATGGCAAGGAATCCATGGCTGTAGATGTGATCAATGACTACT

AA------------------------------------------------------------------------------

--------------------------------------------------------------------------------

--------------------------------------------------------------------------------

--------------------------------------------------------------------------------

--------------------------------------------------------------------------------

--------------------------------------------------------------------------------

--------------------------------------------------------------------------------

--------------------------------------------------------------------------------

--------------------------------------------------------------------------------

--------------------------------------------------------------------------------

--------------------------------------------------------------------------------

----------------------------------------------------------------------------

>TERA03279

ATGAAGATGGCTCTTTTCTCGGAGAGGGCGAAGATAATTAAGCCTCGCGGCAGAGAACCTGATGAATTTGAAACGTCGAT

CTCCCAGGCTCTCCTGGAGCTGGAGATGAACAGTGACCTAAAGGCACAGCTTAGAGAATTGTACATCACTGCGGCTAAAG

AGATAGATGTCCAGGGTAAGAAGGCCATCATTTTATTTGTGCCTGTGCCACAGCTGAAAGCCTTCCAGAAGATCCAGACT

CGTTTAGTCAGAGAGCTTGAAAAGAAGTTCAGTGGGAAGCATGTTGTTGTAATAGCACAGAGAAGAATTCTGCCCAAGCC

TACAAGGAAAAGTAAACGAAAGAATAAACAAAAGCGACCTAGAAGTCGAACTCTGACTGCAGTACACAACAACATTCTGG

ATGACCTGTGTTTTCCAGCAGAAATTGTTGGTAAAAGGATTAGAATTTGTCTTGATGGATCACGAGTGATTAAAGTTCAT

CTGGACAAGACACAGCAAACCAATGTGGAACATAAGTTGGACACATTCTCTGCGGTGTATAAGAAGCTTACTTCAAAGGA

AGTCACCTTTGAATTCCGATAA----------------------------------------------------------

--------------------------------------------------------------------------------

--------------------------------------------------------------------------------

--------------------------------------------------------------------------------

--------------------------------------------------------------------------------

--------------------------------------------------------------------------------

--------------------------------------------------------------------------------

--------------------------------------------------------------------------------

--------------------------------------------------------------------------------

----------------------------------------------------------------------------

>TERA03306

ATGGTTGAGGATTTAAAGGCTCATTTTCTCCATCTGCTACAAATGGTGGATGGACTCCATGCAATCATCATCACAGACAG

AGATGGAGTACCTGTCCTAAAAGTGTCAGATGAGCATCTACCTCATGTTGCAATGCAGCCACGTCTCCTAGCAACTGCTG

CCATGGCAATAGAACAAGCAAACAAACTTGGTGTATCTAATAATAAGAAGATCATCACATTCTATAAAAACCACCAACTG

ATAAGTCTCAATAAGCAGCCACTGATTGTCAACATGATTGCTGGAGCTGATGCAAATACTGGTTTGATTCTGAGCCTCGA

GTCTGACATGCAGGAGCCTTTAGATGATATATGTTAA-------------------------------------------

--------------------------------------------------------------------------------

--------------------------------------------------------------------------------

--------------------------------------------------------------------------------

--------------------------------------------------------------------------------

--------------------------------------------------------------------------------

--------------------------------------------------------------------------------

--------------------------------------------------------------------------------

--------------------------------------------------------------------------------

--------------------------------------------------------------------------------

--------------------------------------------------------------------------------

--------------------------------------------------------------------------------

----------------------------------------------------------------------------

>TERA03340

ATGCATAATCAAATTGTACGTCTGTACAGCAGGTTTACAAGCCTTGATAAAAGCAACAATGGATATCTCAGTCGTGAAGA

TTTCCTCAGAATTCCAGAGCTTGCTATTAACCCTTTAGGAGACCGCATTGTTCATGCTTTCTTTATGTATGGCAATGAAG

AAGGTGTGAACTTCCGTGACTTCATGAAGACCCTAGCAAGGTTCAGGCCGGTCAAACAAGGCACAAACGAAGGCATTAAC

AGCCGTGAAGAAAAGCTAAAATTTGCCTTTAAGATGTATGACTTGGATGGTGATGATAAGATATCAAGAGAGGAGCTCTT

GGCTGTTCTGCACATGATGGTAGGAGCTAACATATCAGAAGAGCAGCTTGGTAATATCGCTGATCGCACAATAGAGGAAG

CTGATCAAGATAGGGATGGAGCCATTGCATTTTCTGAGTTTAAAAAGGTGCTAGAGAAGGTCGATATTGAACAGAAGATG

AGCATCCGGTTTCTGAGCTAA-----------------------------------------------------------

--------------------------------------------------------------------------------

--------------------------------------------------------------------------------

--------------------------------------------------------------------------------

--------------------------------------------------------------------------------

--------------------------------------------------------------------------------

--------------------------------------------------------------------------------

--------------------------------------------------------------------------------

--------------------------------------------------------------------------------

--------------------------------------------------------------------------------

----------------------------------------------------------------------------

>TERA03382

ATGCTGATGCGAGCTCTTGCTAAGCTGATTAAGGTAAATGAGCCCGACTTGGTGCTCTTTGTGGGAGAAGCTCTTGTAGG

TAATGAAGCTGTTGATCAGCTGACAAAGTTTAACCAGGCTCTTGCCGATTATTCTAATGTTGAGAAACCTCACATCATAG

ATGGCATTCTTCTGACCAAGTTTGACACCATAGATGATCAGGTTGGTGCAGCTATATCTATGACATACATCACTGGCCAA

CCAATTGTATTTGTTGGCACTGGGCAGACCTACACTGACTTGAAGAATCTTAATGCCAAAGCTGTTGTTCATTAA-----

--------------------------------------------------------------------------------

--------------------------------------------------------------------------------

--------------------------------------------------------------------------------

--------------------------------------------------------------------------------

--------------------------------------------------------------------------------

--------------------------------------------------------------------------------

--------------------------------------------------------------------------------

--------------------------------------------------------------------------------

--------------------------------------------------------------------------------

--------------------------------------------------------------------------------

--------------------------------------------------------------------------------

--------------------------------------------------------------------------------

----------------------------------------------------------------------------

>TERA03444

ATGCGCGTCAACGAGCTGAACATCACGGTGACGACTCTGACCGGCGACAAGCGCCGCATGGAGGGCGACATCGCCGCCAT

GCAGGCCGACCTTGATGAGGCCCTGAACGCCCGCAGGGCAGCCGAGGAGAGAGCCGATCGCCTCGCCGCCGAGCTGCACC

GCGTCACCGAGGAGCTGCGTCAGGAGCAGGAGAACTACAAGAATGCCGAGTCCCTGCGCAAACAGCTCGAGATCGAAATC

AGAGAGATCACCGTCAGATTGGAGGAGGCCGAGGCGTTCGCTCAGAAGGAGGGAAAGCGTATGGTGGCTAAGCTACAGGC

CAGGCTGCGTGACTTGGAAGCCGAGTTGGAAGTTGAGCAGCGCCGCAGCCGTGAGCTGGCAGCTGCTAACAGGAAGCTCG

AGCGTACACTGAACGAACTCCGTGTCCAGTACGAAGAGGATAGGCGTGTCAACGTGGAGCTGGCCGAACAAGTTAACACA

CTGACAATCAGAATCAAGACCTAA--------------------------------------------------------

--------------------------------------------------------------------------------

--------------------------------------------------------------------------------

--------------------------------------------------------------------------------

--------------------------------------------------------------------------------

--------------------------------------------------------------------------------

--------------------------------------------------------------------------------

--------------------------------------------------------------------------------

--------------------------------------------------------------------------------

--------------------------------------------------------------------------------

----------------------------------------------------------------------------

>TERA03495

ATGATAACAGGTACCAATGAAGTTACAGTACAGAAAAATGATGGTAGCCAAGAGAAAGTGAAAACAAAAAATATTCTTAT

TGCCACCGGCTCAGAGGTGACACCATTTCCAGGTATTGAGATTGATGAAGATACCATTGTGTCATCCACTGGTGCACTGT

CCCTGAAGAAAGTCCCTGAGAAGATGATTGTTATTGGTGCTGGTGTTATTGGTGTTGAATTGGGCTCAGTATGGTCACGC

CTAGGTGCAGAAGTAACTCTTATTGAGTTCCTTGGGAACATCGGTGGAGTTGGAATTGATCTGGAAATTGCCAAGACCCT

GCAGAGGTCACTGACAAAACAGGGTCTAAAATTCAAGCTATCAACGAAGGTGCTTAGTTCATCAAAGGATGGCTCCACAA

TTACAGTTGTTGCTGAGGATACAAAGAAAAACAAACAAGAAGAGTTGACATGTGACACTCTGTAA---------------

--------------------------------------------------------------------------------

--------------------------------------------------------------------------------

--------------------------------------------------------------------------------

--------------------------------------------------------------------------------

--------------------------------------------------------------------------------

--------------------------------------------------------------------------------

--------------------------------------------------------------------------------

--------------------------------------------------------------------------------

--------------------------------------------------------------------------------

--------------------------------------------------------------------------------

----------------------------------------------------------------------------

>TERA03520

ATGGGCGACTCGGAAATCCGATCCCAGATCCTGATGCCAAAGGACCTGTGTGTGGCGCTGACAGAGGAAACAGACGGCTC

CGTGTTCAACACGCTCCAGTACATCAACAGCCGGCCATTCATCCAGAAGCGCTTCATGGACGTCCTAGTCCGAGTGATAG

CTGACAAAGCTGAGCCTTCGGAGTGCCAGATCTGTGAATGTCGTCCGGATAACGTAGGAGCAGGCAAGGCCGACTGTCGT

CACTGCATCTAA--------------------------------------------------------------------

--------------------------------------------------------------------------------

--------------------------------------------------------------------------------

--------------------------------------------------------------------------------

--------------------------------------------------------------------------------

--------------------------------------------------------------------------------

--------------------------------------------------------------------------------

--------------------------------------------------------------------------------

--------------------------------------------------------------------------------

--------------------------------------------------------------------------------

--------------------------------------------------------------------------------

--------------------------------------------------------------------------------

--------------------------------------------------------------------------------

----------------------------------------------------------------------------

>TERA03546

ATGGATATACCAACATTTAAATTGGTGCTAGTCGGAGATGGTGGTGTTGGCAAGACAACGTTTGTGAAGAGGCATTTGAC

AGGAGAATTCGAAAAGAAATACATTGCTACCCTTGGAGTTGAAGTTCATCCTCTTGTGTTCCACACCAACAGAGGGCCAA

TAAGGTTTAATGTTTGGGATACTGCAGGCCAGGAGAAATTTGGAGGACTACGTGATGGATATTATATTCAAGGTCAATGT

GCTATCATTATGTTTGATGTCACTGCTCGTATCACATACAAAAATGTACCAAACTGGCATCGTGATTTAGTAAGAGTGTG

TGAGAATATCCCCATTGTTCTCTGTGGTAACAAGGTTGACATCAAAGACAGAAAAGTTAAAGCCAAATCAATTGTGTTTC

ACAGGAAAAAGAATCTCCAGTACTATGATATCAGTGCAAAAAGTAACTACAATTTTGAGAAGCCATTCCTTTGGCTGGCA

AGGAAGTTGACAGGTGATCCAAACTTGGAATTTGTTGCAATGCCTGCTTTAGCCCCATAA--------------------

--------------------------------------------------------------------------------

--------------------------------------------------------------------------------

--------------------------------------------------------------------------------

--------------------------------------------------------------------------------

--------------------------------------------------------------------------------

--------------------------------------------------------------------------------

--------------------------------------------------------------------------------

--------------------------------------------------------------------------------

--------------------------------------------------------------------------------

----------------------------------------------------------------------------

>TERA03592

ATGAAAGGAGGAGCTACAATTGGTGTTATGATCACAGCCTCGCATAACCCAGAGGAGGATAATGGAGTCAAATTGGTTGA

TCCAGCTGGTGAGATGCTTGAGGCAGCTTGGGAGAAGCATGCTGCCCACTTAGCTAATGTTACTGATGCTGATGTGCCAG

ACTGCTTAAAGTCCTAA---------------------------------------------------------------

--------------------------------------------------------------------------------

--------------------------------------------------------------------------------

--------------------------------------------------------------------------------

--------------------------------------------------------------------------------

--------------------------------------------------------------------------------

--------------------------------------------------------------------------------

--------------------------------------------------------------------------------

--------------------------------------------------------------------------------

--------------------------------------------------------------------------------

--------------------------------------------------------------------------------

--------------------------------------------------------------------------------

--------------------------------------------------------------------------------

--------------------------------------------------------------------------------

----------------------------------------------------------------------------

>TERA03644

ATGAAGGAATGTGACCTGGATGAGGACAACAAGCCAATCTGTGTCTGTACAAGGAAGTGTCCAGAGGAAAATGATCCCAG

AGCTAAGGTGTGCAGTACAATGAACGTGACCTTTGCCAGTGAGTGCGAGTTGTACAGACAGAAGTGCTTGTGCAGACGTA

ACCAGGCTGGCTGTGCTGATCGTGAGTACAGGAGAGGACACCTGGACTACTTTGGCGAGTGCAGAGAAATCCCGCCCTGT

AAGGATTGGGAACTCGAAGAATATCCGATCCGTATGAGGCAGTGGTTGTACCTTGTTATGGAGGAACTGGCCAGCCGCCG

TGACCTACAAGGACGGGCCTTGAAGCTGGCCCAGGAAGCCAAGGCCAGACACAACAAGAAGTGGGTCATCCCTGTCATCT

GGAAGTTCTGTGACCTTGACATTTCCAAGGACAAGATGATTTCGACCAAAGAACTGCTGCCGATCAGTGCACCTTTAAAA

CCGTTGGAACACTGCACTGGTCCGTTCCTGGAATCGTGTGACAAGGACGGAAGTGGTGATATCACCCTGACAGAATGGGG

ACAGTGTCTGGGACTTGATGCAGATGACATCGAGGATCTGTGTGATTAA-------------------------------

--------------------------------------------------------------------------------

--------------------------------------------------------------------------------

--------------------------------------------------------------------------------

--------------------------------------------------------------------------------

--------------------------------------------------------------------------------

--------------------------------------------------------------------------------

--------------------------------------------------------------------------------

--------------------------------------------------------------------------------

----------------------------------------------------------------------------

>TERA03652

ATGGAGCATCCGCCTGTACCGAGCAAAACTTGGGAACTCAGCCTTTATGAGCTGAACAGGACGCCACAAGATGCGATTAC

TGATAATACAGAGATAGCGGTTTCGCCAAGAAGTTTGCATAGCGAGCTAATGTGCCCGATATGCCTGGATATGCTGAAAA

ATACAATGACGACGAAGGAGTGTTTACACAGGTTCTGTTCCGAGTGCATAATAACGGCCCTGCGTAGTGGCAACAAGGAA

TGCCCGACGTGCAGGAAGAAACTCGTCTCAAAGCGGTCTCTCCGACCGGATCCTAACTTCGACGCGCTCATATCGAAGAT

CTATCCGAGCCGCGAGGAGTACGAGGCTCACCAGGAGCGGGTGCTGGCAAAGCTAAAACAGTCCGTCAACCAGAATGCCT

TGTCGCAGAGCATCGAAAAGGGCATCCAGCTGCAGGCCATGTACAGGGGCCAGAGGGTGAAGAAGTAA------------

--------------------------------------------------------------------------------

--------------------------------------------------------------------------------

--------------------------------------------------------------------------------

--------------------------------------------------------------------------------

--------------------------------------------------------------------------------

--------------------------------------------------------------------------------

--------------------------------------------------------------------------------

--------------------------------------------------------------------------------

--------------------------------------------------------------------------------

--------------------------------------------------------------------------------

----------------------------------------------------------------------------

>TERA03703

ATGGAATTATCTGAGTATGGTTTAACAGCTGGTGATAAACCTGTGATAGCAGCTCGTGATACCAGTGACAAGAAATATGT

GATGTCTGGGGAATTCAGCATGGAAAATCTAGAGAAATTTGTTAATGATCTGCTAGATGGCAAATTGAAGCCATACCTGA

AGAGTGAACCTATACCTGAAGACAATGATGGACCAGTTAAGGTTGTTGTAGCAGAAACATTTGATTAA------------

--------------------------------------------------------------------------------

--------------------------------------------------------------------------------

--------------------------------------------------------------------------------

--------------------------------------------------------------------------------

--------------------------------------------------------------------------------

--------------------------------------------------------------------------------

--------------------------------------------------------------------------------

--------------------------------------------------------------------------------

--------------------------------------------------------------------------------

--------------------------------------------------------------------------------

--------------------------------------------------------------------------------

--------------------------------------------------------------------------------

--------------------------------------------------------------------------------

----------------------------------------------------------------------------

>TERA03839

ATGGAGGTGTATGTTGATGATGACTCCAAGCTGACACTTCATGGACTACAACAGCATTATGTCAAACTGAAGGACAATGA

GAAAAACCGCAAACTCTTTGAGCTTCTGGATGCCCTGGAGTTTAATCAGGTTATTATCTTTGTAAAATCAGTACCAAGAT

GCATGGCGCTCTGTCAGTTACTCGTTGAACAGAACTTCCCAGCAATTGCTATACACAGGGCTATGTCACAAGAAGACAGA

CTGAGCCGATATCAGCAATTTAAAGATTTCCAGAAGCGTATACTGGTTGCTACCAACCTGTTTGGCCGTGGTATGGATAT

AGAACGAGTCAACATTGTGTTTAACTATGACATGCCAGAAGACTCGGACACATACCTGCACAGGGTGGCTCGTGCTGGCA

GGTTTGGTACAAAGGGTCTCGCTATAACCTTTGTGTCTGATGAAACTGATGCCAAGGTACTTAATGATGTCCAAGACAGG

TTTGAAGTGAACATCACTGAGCTGCCAGAAGAGATTGACATTTCATCATACATTGAAGGCCGATAA--------------

--------------------------------------------------------------------------------

--------------------------------------------------------------------------------

--------------------------------------------------------------------------------

--------------------------------------------------------------------------------

--------------------------------------------------------------------------------

--------------------------------------------------------------------------------

--------------------------------------------------------------------------------

--------------------------------------------------------------------------------

--------------------------------------------------------------------------------

----------------------------------------------------------------------------

>TERA03898

ATGAAAATGTGGTATGAAATATTGCCAAGTTTAGCTATAGTCTGGGTGTTCATCGCCCTGCCACCGTATGGAGGACTGGC

ATGCAACTGGCTTTTTAACAACAGAAAGACAGTTGCACGTCATTGGGAAGGAAATGTACATGACTTCCAATCATTCTTGC

GCGACAGACGGTTAACAGGCAGTGAATAA---------------------------------------------------

--------------------------------------------------------------------------------

--------------------------------------------------------------------------------

--------------------------------------------------------------------------------

--------------------------------------------------------------------------------

--------------------------------------------------------------------------------

--------------------------------------------------------------------------------

--------------------------------------------------------------------------------

--------------------------------------------------------------------------------

--------------------------------------------------------------------------------

--------------------------------------------------------------------------------

--------------------------------------------------------------------------------

--------------------------------------------------------------------------------

--------------------------------------------------------------------------------

----------------------------------------------------------------------------

>TERA03941

ATGGATCAGCCAGAACGCTGGAATCGCATAAAACTTGTTGTCAGTGAGGATGATGTACGGAAGGCATACCAGAGGGCAAT

GTTTAGTATGGCAAGGCTAAATAGAATGGGTGCACATTTGATGCACAAATACAATGCACATGGTGCAACTGATGTCACAG

GATTTGGACTCCTTGGTCATGCCAATAACTTGGCTCGTGTCCAGAAAAATGAAGTTGGTTTTGTCATCCATAACCTACCA

ATCATAGCCAAAATGGCCGCAGTGAGCAAGGCATGTGGAAACATGTTTGGTCTTCTCCAAGGTCATTCAGCAGAAACATC

AGGTGGTTTGCTGATTGCATTGCCCAGAGAACAGGCTGCAGCATTCTGCAAGGACATTGAGAAACAAGAAGGATACCAAG

CTTGGATCATTGGTATAGTTGAGAAGGGTACACGAACAGCAAGAATTATTGACAAGCCCCGTGTTATTGAGGTCCCAGCT

AAAGAAAAGGATGGTGAACTTTGGTAA-----------------------------------------------------

--------------------------------------------------------------------------------

--------------------------------------------------------------------------------

--------------------------------------------------------------------------------

--------------------------------------------------------------------------------

--------------------------------------------------------------------------------

--------------------------------------------------------------------------------

--------------------------------------------------------------------------------

--------------------------------------------------------------------------------

--------------------------------------------------------------------------------

----------------------------------------------------------------------------

>TERA03964

ATGGCTGGCAGCATATCCAGAAACAGTCCAGCAGCCCGATATCTTGCCAGCAAAGGGTTAACACCCCGTGAGTTCAACTC

ATATGGCTCCCGTCGTGGTAATGATGCTGTCATGGCTCGTGGCACCTTTGCTAACATTCGTCTGCTGAACAAATTTATTG

GCAAGGCTGCTCCAAAGACTGTATACATTCCAACAGGCGAAGTGATGGATGTGTTTGATGCGTCCCAGAGATACTTGTCA

GATGGTCACCAAGTGATCATTTTAGCTGGCAAGGAATATGGTTCAGGTTCTTCTCGAGACTGGGCTGCTAAAGGACCATG

GATGTTGGGCATCAAGGCTGTTATTGCTGAGAGCTATGAACGGATACATCGCAGTAACCTAGTTGGGATGGGCATAATAC

CTCTGCAGTACCGTCCAGGTGACACTGCCGAATCGCTTGGTTTAACAGGCAAAGAACGGTACACTATTGAAATACCAGAA

GATTTGAAAACAGGGCAGCTTGTTACTGTGAAGCTGAATGATGGACGCAGCTTCCAGGTGACAGCACGTTTTGACACTGA

TGTTGAACTGACCTATTTCCATCATGGGGGAATCCTCAACTACATGATCAGGACCATGCTGTAA----------------

--------------------------------------------------------------------------------

--------------------------------------------------------------------------------

--------------------------------------------------------------------------------

--------------------------------------------------------------------------------

--------------------------------------------------------------------------------

--------------------------------------------------------------------------------

--------------------------------------------------------------------------------

--------------------------------------------------------------------------------

----------------------------------------------------------------------------

>TERA03983

ATGGGTGCCTATACCATGTGTGCTGCATCAAATTTCAATGGAATGCCAAAGCCTCGCTGTTTCTATGTCATTCAAGATGT

GTCATGGCACCAGTTGAATTCAAAGAAATGTGGAAGTGGTTTGCTGCGAGCTGTGTCAGAGATGAAGTCAGGCCATGAGA

TCCATGCTGATGCTGACCACTGCGATGTCTGCCCAGCAATAACCGTTTAA------------------------------

--------------------------------------------------------------------------------

--------------------------------------------------------------------------------

--------------------------------------------------------------------------------

--------------------------------------------------------------------------------

--------------------------------------------------------------------------------

--------------------------------------------------------------------------------

--------------------------------------------------------------------------------

--------------------------------------------------------------------------------

--------------------------------------------------------------------------------

--------------------------------------------------------------------------------

--------------------------------------------------------------------------------

--------------------------------------------------------------------------------

--------------------------------------------------------------------------------

----------------------------------------------------------------------------

>TERA03999

ATGGGTCGTGTAAGGACTAAGACGGTGAAGAAGGCATCGCGAGTGATAATCGAGAAGTATTACACTCGCTTGACCCTGGA

CTTCCACACAAACAAGAGAATATGTGAAGAAATCGCCATAATTCCCAGCAAGAAGCTGAGAAACAAGATCGCAGGCTTTG

TCACTCACCTGATGAAGAGGATCCAGAAAGGTCCAGTGCGAGGTATTTCCATCAAGTTACAGGAAGAAGAAAGGGAGAGG

AGAGATAACTATGTGCCTGAGATATCAGCTATTGAGCAAGATATCATTGAAGTTGATCCAGACACTAAGGAGATGTTGAA

GTTACTGGATATGAATAATATCCCAGTCCAAGTGACACAGCCCATGGCACAGACGACAGGCTTCAACAGGCCACCAAGGT

AA------------------------------------------------------------------------------

--------------------------------------------------------------------------------

--------------------------------------------------------------------------------

--------------------------------------------------------------------------------

--------------------------------------------------------------------------------

--------------------------------------------------------------------------------

--------------------------------------------------------------------------------

--------------------------------------------------------------------------------

--------------------------------------------------------------------------------

--------------------------------------------------------------------------------

--------------------------------------------------------------------------------

----------------------------------------------------------------------------

>TERA04100

ATGTCTAAGTACGAGGGTATCGCCGCCATGTACATGTCGATGCCGATGGCAGCCCAAGCCTTGCCCATCCTCGGCAGCTG

TACCGTGGAAGACAAGAAGATTTCGCTGCGCTTTCCTCTCAGCAATGTCTCCTTTGACCTGCCCGAGGCACCGAGGGAAG

GCGGCCGAGACGTCGAGTTCAAGATGGCCGGCCCGAAGGGCGAGATGAACTTAAAGATCTCCTACAAGTCCGACCTTCAG

GGCTTCGTGGGTCAAGGCCAGCAGGACGGACACAACGTGCTAACCTTTGTCTTCTACAGACCTGGATCAGGACTCAGTAA

CCTGAAGTAA----------------------------------------------------------------------

--------------------------------------------------------------------------------

--------------------------------------------------------------------------------

--------------------------------------------------------------------------------

--------------------------------------------------------------------------------

--------------------------------------------------------------------------------

--------------------------------------------------------------------------------

--------------------------------------------------------------------------------

--------------------------------------------------------------------------------

--------------------------------------------------------------------------------

--------------------------------------------------------------------------------

--------------------------------------------------------------------------------

----------------------------------------------------------------------------

>TERA04145

ATGCGTCCCTTTGACCCGACTGATAAATGCTACGAGTGTGGCGAGCGAGGCCATTATGCATACGACTGTAAGCGTAACAG

CCATGGACGGAAACGATCAAGGTCTAGGTCCAGGTCTTATGAGCGTGGTGGCCGTCGCAGCTACACCCCATCCAGAAGTC

GTAGCAGGGAGGCATCAAGATCTCCTGGCAGATACAGCAGGTCAAGGTCTCGAAGTCCATAA------------------

--------------------------------------------------------------------------------

--------------------------------------------------------------------------------

--------------------------------------------------------------------------------

--------------------------------------------------------------------------------

--------------------------------------------------------------------------------

--------------------------------------------------------------------------------

--------------------------------------------------------------------------------

--------------------------------------------------------------------------------

--------------------------------------------------------------------------------

--------------------------------------------------------------------------------

--------------------------------------------------------------------------------

--------------------------------------------------------------------------------

--------------------------------------------------------------------------------

----------------------------------------------------------------------------

>TERA04146

ATGGTACCATTTTACAGGAGAATGCCCCAAGAGCCCTATTACAAAGATCATGCCTTTCGTAAGCAGTATCCACCAGTGTC

GTTGTTACAGTTACAGCGTTTGATAGATCTGGGTCGAATTGATACCACAGAACCAATAGATTTGACAACTATTTGTAACA

CAAAGATTGTTACAGTCAATCCACACCTTAAACATTATGGCATCAACTTAACAGATGAAGGAGCTGATATTTTTGCTGCC

AAAGTCAACATAGAAGTCCAGTGGACCAATGAGATCACAATAGCTGCCATTGAAAAATGTGGTGGCACAATCACAACAAA

GTTCTATGACCTCCAGTGTGTGGAAGCCATGGTAAACCCTTCTCTGTTCTTTAAGCGTGGCTTACCAATTCCAAGATGCA

AGCTGCCATCTATGGATGTCATTGAGTATTACAGTGATGCAAGGAATCGAGGTTATCTGGCTGATCCAGAACTGATTCAA

GAGGCACGCATTGAACTTGCTCAGAAGATGGGCTATTAA-----------------------------------------

--------------------------------------------------------------------------------

--------------------------------------------------------------------------------

--------------------------------------------------------------------------------

--------------------------------------------------------------------------------

--------------------------------------------------------------------------------

--------------------------------------------------------------------------------

--------------------------------------------------------------------------------

--------------------------------------------------------------------------------

--------------------------------------------------------------------------------

----------------------------------------------------------------------------

>TERA04198

ATGAGCACTGCATTCTCCAGAGACCAGGCTCACAAGATCTATGTTCAGCATCGTATTGCTGAGCATCCAGAGCTGATCTA

TGACTATCTGTGGAAGCGCAAGGGTTACTTTTATTTGTGTGGCCCAGCTGGCAATGTGCCAATGTCTGTCAGGAAGGCTG

TTGTTGATGCATTCGTCAGTCAGGGTGGCCACTCCCTGGCAGAGGCAGACAAGATGGTCACCCAGATGCAGATTGAAGGG

AGATACAATGTTGAGGCTTGGTAA--------------------------------------------------------

--------------------------------------------------------------------------------

--------------------------------------------------------------------------------

--------------------------------------------------------------------------------

--------------------------------------------------------------------------------

--------------------------------------------------------------------------------

--------------------------------------------------------------------------------

--------------------------------------------------------------------------------

--------------------------------------------------------------------------------

--------------------------------------------------------------------------------

--------------------------------------------------------------------------------

--------------------------------------------------------------------------------

--------------------------------------------------------------------------------

----------------------------------------------------------------------------

>TERA04235

ATGGAGCCGGTCTGGAACTGGCTAAATCTCGTCGTGTCCGTGGCGTCGATCTTCTGCTGCTCTGTCTGCGGAGTGCTGGC

CACCGTCACCTCGGTGCTCGCTTACGTCGACCACCGGATCGGAGACTTCAGCGCCGCGCACCACAAGCGAGTGGCCTCCT

ACGCGCTGGCTCTGTCCGCCCTCATCGCTGGCCTCATCTGCATGATCATCATCTAA------------------------

--------------------------------------------------------------------------------

--------------------------------------------------------------------------------

--------------------------------------------------------------------------------

--------------------------------------------------------------------------------

--------------------------------------------------------------------------------

--------------------------------------------------------------------------------

--------------------------------------------------------------------------------

--------------------------------------------------------------------------------

--------------------------------------------------------------------------------

--------------------------------------------------------------------------------

--------------------------------------------------------------------------------

--------------------------------------------------------------------------------

--------------------------------------------------------------------------------

----------------------------------------------------------------------------

>TERA04265

ATGTATGATTCTGTGCATAATCAGATCTTCTCACTTCCTGAAGACACTGCTCTGTATCCTGCTCACGACTACAAAGGCAT

GACGATGACTACTGTTGGTGAAGAGAAGAAACTGAACCCTCGTCTGACAAAGTCTAAATCGGACTTTATCAAGCTCATGA

ACGAGTTGAACCTGCCATATCCAAAGCAGATAGACAAGGCCCTCCCAGCCAACATGGTTTGTTAA---------------

--------------------------------------------------------------------------------

--------------------------------------------------------------------------------

--------------------------------------------------------------------------------

--------------------------------------------------------------------------------

--------------------------------------------------------------------------------

--------------------------------------------------------------------------------

--------------------------------------------------------------------------------

--------------------------------------------------------------------------------

--------------------------------------------------------------------------------

--------------------------------------------------------------------------------

--------------------------------------------------------------------------------

--------------------------------------------------------------------------------

--------------------------------------------------------------------------------

----------------------------------------------------------------------------

>TERA04270

ATGATACGCTTGTTCCACCCAAGTGTGGACAAGGGTCCAAAGGGCAAAGGGCATCCAATAAACCCATCCATAAGAAAAGA

CTGCCCCAAGGTCGTTGACATACTGGATGTTGAAGACCTTGACAAGGAGAAAGTGTCGTACTGTAGATGCTGGAAGTCGA

AGAAGTTTCCACTCTGTGATGGCAGTCATAATGCCCACAATGAGGAGACAGGTGATAACGTTGGTCCAGTTGTGATCAAG

AAGAAGATCTAA--------------------------------------------------------------------

--------------------------------------------------------------------------------

--------------------------------------------------------------------------------

--------------------------------------------------------------------------------

--------------------------------------------------------------------------------

--------------------------------------------------------------------------------

--------------------------------------------------------------------------------

--------------------------------------------------------------------------------

--------------------------------------------------------------------------------

--------------------------------------------------------------------------------

--------------------------------------------------------------------------------

--------------------------------------------------------------------------------

--------------------------------------------------------------------------------

----------------------------------------------------------------------------

>TERA04503

ATGATAACAGCTACGTCTGGGTTTATGAGAGGTCATGGAACCTACACTGAAAGTGAAAAGTTGCTGGCTTCTGTCGCAGG

AATTGTAGAACGATACAATAAACTTGTATGTGTGAAACCTTTGAAAACAAGGTATAATGGAGAGATTGGAGATGTGGTTG

TTGGCAGGATCATTTCTGTTGGACAGCATCGGTGGAAAGTTGACACACACTCAAAGTTAGATTCCTTTCTGATGTTGTCT

TCTGTGAACCTTCCTGGAGGAGAACTGAGGCGAAGGTCAGAACAGGATGAACAAATGATGAGACACTATCTGTCAGAGGG

CGATGTCATCAGTGCTGAAATCCAAAATGTCTTCTCAGATGGTTCATTATCATTACACACACGGAGTCTGAAATATGGAA

AGCTTGGACAAGGTATACTAGTACAAGTATCACCATCACTTGTAAAAAGACGTAAAACACATTTTCACAATCTGCCTTGT

TAA-----------------------------------------------------------------------------

--------------------------------------------------------------------------------

--------------------------------------------------------------------------------

--------------------------------------------------------------------------------

--------------------------------------------------------------------------------

--------------------------------------------------------------------------------

--------------------------------------------------------------------------------

--------------------------------------------------------------------------------

--------------------------------------------------------------------------------

--------------------------------------------------------------------------------

----------------------------------------------------------------------------

>TERA04607

ATGGGAAATCACACACCTGTCTTTAAACATTCTGGCTTGGATGTGAAGTCCTATAAATATTATGACCCTAACACATGTGG

ATTTGATGCTGCAGGTGCCATGGATGACATCTCTAAAATCCCAGAGGGTTCAATCATTCTCTTACATGCCTGTGCACATA

ATCCAACTGGTGTAGATCCTAAGCCAGAACAGTGGAAAGAACTCAGTGCTCTGATCAAGAAAAAGAAATTGTTCCCATTC

TTTGATATGGCTTACCAAGGGTTTGCTAGTGGTGATGTGAACAGAGATGCATTTGCCCTGAGACAATTTGTGCAGGATGG

ACACAAAGTTGCTTTAGCACAGTCATTTGCCAAGAACATGGGTCTTTATGGTGAGAGAGCTGGAGCATTCTCTCTTGTCT

GCGCTGATGAGGATGAAGCAAAGCGAGTCATGTCACAAATCAAGATACTGATACGACCAATGTACTCAAATCCACCAATA

AATGGAGCCCGTATTGTCCAAACAATACTCAGTGATCCAGGACTGACTAAACAATGGTTGGCTGATGTGAAGGTGATGGC

AGATCGTATTATTTCAATGAGAACCAAGCTCAGAGACAACCTGGCCAAGGAAGGTTCAAGTAAAAACTGGCAGCACATCA

CAGATCAGATTGGAATGTTTTGTTACACCGGGCTGAAGCCAGACCAGGTGGAGAGGCTGACCAAAGAATTTTCTGTTTAC

CTGACTAAAGACGGTCGTATTTCTATGGCTGGTGTGACATCTGGATAA--------------------------------

--------------------------------------------------------------------------------

--------------------------------------------------------------------------------

--------------------------------------------------------------------------------

--------------------------------------------------------------------------------

--------------------------------------------------------------------------------

--------------------------------------------------------------------------------

----------------------------------------------------------------------------

>TERA04631

ATGAATGAAATTTCCAAAAACAAAGTTTTTGGTGGCTGGCAGAAAGTCTTCAGTCATGAGAGCAAGGAGCTGAAATGCAA

GATGAATTTTGGAATATATTTACCTCCTCAGGCAGAAGATAGCAAAGTTCCTGTTATTTATTGGCTCTCAGGACTGACAT

GCACAGAACAGAACTTCGTAACAAAAGCTGGTGCCCAGAAGTATGCATCTGAACATGGCATTGCTATTGTGGCACCTGAC

ACCAGTCCAAGATAA-----------------------------------------------------------------

--------------------------------------------------------------------------------

--------------------------------------------------------------------------------

--------------------------------------------------------------------------------

--------------------------------------------------------------------------------

--------------------------------------------------------------------------------

--------------------------------------------------------------------------------

--------------------------------------------------------------------------------

--------------------------------------------------------------------------------

--------------------------------------------------------------------------------

--------------------------------------------------------------------------------

--------------------------------------------------------------------------------

--------------------------------------------------------------------------------

----------------------------------------------------------------------------

>TERA04698

ATGGCCATGGCTAAGCTTGTTGGACAGTGGAAGCTGGAAAGCTCGGAAAATTTCGATGAATACATGAAGGCGGTTGGTGT

TGGATTCGCCACTCGTAAGCTAGGCAACCTGGCCAAGCCCTCGCAGATCATTAAAGTTGATGGCGACACCTGGACAATTG

AAACCCACTCAACCTTTAAGAACACAATACTGAAGTTTGAATTGGGAAAGGAATTTGAGGAAACCACTGCTGATGGTAGA

AAGGTCAAGACTACCTTCACAGCTGAGGGTGACACTAAGCTGATTCAGAGCCAGAAGGGCGAAATTGATTCTACATTAAC

CAGAGAATTAACAGATGACAACACTCTGGTTATGACTTGTGTGGCCAAGGATTAA-------------------------

--------------------------------------------------------------------------------

--------------------------------------------------------------------------------

--------------------------------------------------------------------------------

--------------------------------------------------------------------------------

--------------------------------------------------------------------------------

--------------------------------------------------------------------------------

--------------------------------------------------------------------------------

--------------------------------------------------------------------------------

--------------------------------------------------------------------------------

--------------------------------------------------------------------------------

--------------------------------------------------------------------------------

----------------------------------------------------------------------------

>TERA04705

ATGTTCGACAACTGTGTATCCGAGTCTGAGGTCACTTTTGGTGATTGTCAGGGCACATGCAGCATCAAGGACACATACAC

GAAATTAGACGGCTACAACATCTTGAGACTGACCAGAGACTGCCAGTGCTGCCAACCGGCGGAAGTACGCTACATTACCG

TCCCATTCAAATGTGAAGGAGTAGCGGAAGTACAAGTGTACAAGGTACCGACTGCTGTCTCTTGTGCCTGCTTTGGCTAA

--------------------------------------------------------------------------------

--------------------------------------------------------------------------------

--------------------------------------------------------------------------------

--------------------------------------------------------------------------------

--------------------------------------------------------------------------------

--------------------------------------------------------------------------------

--------------------------------------------------------------------------------

--------------------------------------------------------------------------------

--------------------------------------------------------------------------------

--------------------------------------------------------------------------------

--------------------------------------------------------------------------------

--------------------------------------------------------------------------------

--------------------------------------------------------------------------------

----------------------------------------------------------------------------

>TERA04852

ATGTCTAAGGTTGGACCTTTGCCGTGGCTTGGTCCTCAGACAGATGAGGTGATCACAGCTCTTGCCAAACAAGGTAGAAA

GAATGTTTTACTTGTGCCTGTTGCTTTCACTAGTGACCATATCGAGACCCTGTATGAACTGGACTTGGAATATGCTAAGC

ATCTTGGTGAAAAGGTTGGAATGAAAATGATACGCAGAGCTGCTGCACTAAATGATAATCAGACATTCATAAGGGCTCTG

GCTGATATTGTGAAGGAACACCTGGACAGCTTCAAGTCATCCACTAAGCAGTTTGAACTGTGCTGCCCTCTGTGCACAAA

TCCCAAATGTAAGAAAACAAAGGAGTTCTTCTTTGACATGCAAGGAATCCTGGACCATTTCAAAGAGCGTAGAGAACACC

TGCTTACAAGAGAGGGTTAA------------------------------------------------------------

--------------------------------------------------------------------------------

--------------------------------------------------------------------------------

--------------------------------------------------------------------------------

--------------------------------------------------------------------------------

--------------------------------------------------------------------------------

--------------------------------------------------------------------------------

--------------------------------------------------------------------------------

--------------------------------------------------------------------------------

--------------------------------------------------------------------------------

--------------------------------------------------------------------------------

----------------------------------------------------------------------------

>TERA04884

ATGACAAAGATGTCTGCTTTCAAGAAGTTCCTGCCCATGTTCGACCGTGTCCTCGTTCAGAGATTTGCGGCTGAGACAAA

GACAAAGGGAGGTGTATTGATACCAGAGAAGGCACAGTCCAAAGTGCTCAATGCCAAAGTTGTAGCTGTTGGTGGTGGAG

CCAGAAATGAGAAGGGTGAAACAGTACCACTACAGGTAAAGGTTGGAGATCAAGTTTTGCTGCCTGAATTTGGAGGAACT

AAAGTTGTAATTGATGACGAGGAGCTGCATTTATTCCGCGAAAGTGACATCCTAGGAAAGTTTGAGTAA-----------

--------------------------------------------------------------------------------

--------------------------------------------------------------------------------

--------------------------------------------------------------------------------

--------------------------------------------------------------------------------

--------------------------------------------------------------------------------

--------------------------------------------------------------------------------

--------------------------------------------------------------------------------

--------------------------------------------------------------------------------

--------------------------------------------------------------------------------

--------------------------------------------------------------------------------

--------------------------------------------------------------------------------

--------------------------------------------------------------------------------

----------------------------------------------------------------------------

>TERA05061

ATGCAAGTGGATGACACATTTACGATAGACAACATTGAACTGAACCAGGTTACAATTGTAGGTATTGTGCGATCAGTGAA

GGAGTCAGCCACAAAAATAGAATACTTCATTGATGATATGACAGGTCCACCACTGGATGTTAGACAGTTTGTTGACAATG

ATGAAAATACCCCAGATGAAGACAGGGTGGTTGCTCTGAGGGAGAATATGTATGTCCGAGTTCATGGTCACTTGCGATCC

TTCCATGGCAAGCAACATGTGATGGCATTTAGAATAGTTCCCCTCACAGACATGAATGAATAA-----------------

--------------------------------------------------------------------------------

--------------------------------------------------------------------------------

--------------------------------------------------------------------------------

--------------------------------------------------------------------------------

--------------------------------------------------------------------------------

--------------------------------------------------------------------------------

--------------------------------------------------------------------------------

--------------------------------------------------------------------------------

--------------------------------------------------------------------------------

--------------------------------------------------------------------------------

--------------------------------------------------------------------------------

--------------------------------------------------------------------------------

----------------------------------------------------------------------------

>TERA05241

ATGGAAGATGAGAAAGAAACACTAGCATTTAAAGTTTTGTCTGCTGAAAATACTAAATTATTAGCTGAAATAGACGATGT

GGATGAACTTCAGAACAAAGTTGCAGAAGTACTGCAGATTTCCAACCACAGCATAGATCTGAAGGATGGTTCCACACTTG

ACTACTATGTGGCTGGTCTGTGGTGGGCGAAGGAGCAGTGCTATAACCAAGAACAGATTTCGGCTTTCTTCACTGTATTA

CACACCCTCTTGGAAAATGTCAAAGAAAAGCAAATGACACTGATTGACAACTTGAGGGAGTTCAAGAAGATGCTGGCTGG

GATTGGTGTTGAACCTAATCCAGAAACCCCAATAAAAAGTGGAGGGCTTGATGGAATTTTTGACTAA-------------

--------------------------------------------------------------------------------

--------------------------------------------------------------------------------

--------------------------------------------------------------------------------

--------------------------------------------------------------------------------

--------------------------------------------------------------------------------

--------------------------------------------------------------------------------

--------------------------------------------------------------------------------

--------------------------------------------------------------------------------

--------------------------------------------------------------------------------

--------------------------------------------------------------------------------

--------------------------------------------------------------------------------

----------------------------------------------------------------------------

>TERA05482

ATGACTGTTGATGGCGATTTCAAGTTGGCGCAAAGTTTGGCTATTGCTAGATTCCTGGCAAAGCGATTTGGATTTCTTGG

AAAGGATGAATTTGAGGAAGCTAAATGTAATATGGTGCTGGAATGTATTGAAGACATGATGCACGCATTCTTCAAAGTAC

TATTTGGGGATGATGCTTCAAAGGCTGCAGCCAAGGAGAAATGGCAAGGTGGTCAGAGGGACGAATTTCTGACCAAGTTT

GAAAAACTCCTCACCACAAACAACAATGGTGATGGCTTCTTTGTTGGAGATGGGATCACTGTTGCTGATATGTAA-----

--------------------------------------------------------------------------------

--------------------------------------------------------------------------------

--------------------------------------------------------------------------------

--------------------------------------------------------------------------------

--------------------------------------------------------------------------------

--------------------------------------------------------------------------------

--------------------------------------------------------------------------------

--------------------------------------------------------------------------------

--------------------------------------------------------------------------------

--------------------------------------------------------------------------------

--------------------------------------------------------------------------------

--------------------------------------------------------------------------------

----------------------------------------------------------------------------

>TERA05680

ATGAAATATCACATGTCATATCTAACAGATGGCTGTTGGTCACCTGTTCGTCCTGCTGTCTTCTTTACAACAAAGCGAGA

TGGTACCCTTGATATTTGGGATATAATCTTCAAACAGAACGACCCAACTCTCAGCATCCAGGTCTGTGATGAACCATTAC

ACAGTATTCGTGTCCAAGATGGTGGCCGTCTGATTGCAGCTGGCTCACACAGTGGTACGACAACTTTACTTGAGTTGTCT

GATGGCCTCTGCATGCAGTCAAAGTCCGAGAAGAATCTCGTAACCATGATGTTTGATCGAGAGACTAGGAGAGAGAGGAT

TCTTGAAGCTAGGCACAGAGAGATGAAACTATAA----------------------------------------------

--------------------------------------------------------------------------------

--------------------------------------------------------------------------------

--------------------------------------------------------------------------------

--------------------------------------------------------------------------------

--------------------------------------------------------------------------------

--------------------------------------------------------------------------------

--------------------------------------------------------------------------------

--------------------------------------------------------------------------------

--------------------------------------------------------------------------------

--------------------------------------------------------------------------------

--------------------------------------------------------------------------------

----------------------------------------------------------------------------

>TERA05820

ATGGGTATCTCCCGGGACAAATGGCACAAGCGCAGGAAGACCGGTGGGCGTATGAACCCCATCCGGAAGAAGAGGAAGTT

CGAATTGGGAAGACCCGCTGCAAACACGAAGCTTATGCCAAAGCGCATACACACCGTAAGAACTCGAGGTGGTAACAAGA

AGTACAGAGCTTTGAGGCTGGACATGGGAAACTTCTCTTGGGGATCTGAAGCCATCACCAAGAAGACACGTATCACTGAT

GTTGTATACAATGCCAGCAGTAACGAGCTTGTACGTACAAAGACCCTCGTTAAAAACTGCATAGTTGTCATTGATGCATC

ACCCTTCAGATTGTGGTATGAGGCTCACTATGCCATGCCTCTGATTAAGAAGTAA-------------------------

--------------------------------------------------------------------------------

--------------------------------------------------------------------------------

--------------------------------------------------------------------------------

--------------------------------------------------------------------------------

--------------------------------------------------------------------------------

--------------------------------------------------------------------------------

--------------------------------------------------------------------------------

--------------------------------------------------------------------------------

--------------------------------------------------------------------------------

--------------------------------------------------------------------------------

--------------------------------------------------------------------------------

----------------------------------------------------------------------------

>TERA05878

ATGTTCGCCACTCCAGGATTCAACTACAGTGATCCAGAAGGCTCGACGTATCCACCTAACATCATCTGTCAGTGGTCGGT

CTTTGTGGAACCTGGTACGGAGCTTCAACTTGGCTTCTTTAACTTCTCAGTCGAGTACACGACTGACTGCAACGAGGAAT

CAGTCACTGTATACGATGGACCTGATGACTCGTAA---------------------------------------------

--------------------------------------------------------------------------------

--------------------------------------------------------------------------------

--------------------------------------------------------------------------------

--------------------------------------------------------------------------------

--------------------------------------------------------------------------------

--------------------------------------------------------------------------------

--------------------------------------------------------------------------------

--------------------------------------------------------------------------------

--------------------------------------------------------------------------------

--------------------------------------------------------------------------------

--------------------------------------------------------------------------------

--------------------------------------------------------------------------------

--------------------------------------------------------------------------------

----------------------------------------------------------------------------

>TERA05882

ATGGATTTTACATTTGTTTGTCCAACTGAAATCACTGCCTTCAGTGACAGGGTGGACGAATTCAAGAAAATTAACACTGA

AGTACTGGCTGCTTCTGTTGATTCGCCATTCGCACATTTAGCATGGATAAACACGCCTCGGTCAGAAGGTGGACTTGGAT

AA------------------------------------------------------------------------------

--------------------------------------------------------------------------------

--------------------------------------------------------------------------------

--------------------------------------------------------------------------------

--------------------------------------------------------------------------------

--------------------------------------------------------------------------------

--------------------------------------------------------------------------------

--------------------------------------------------------------------------------

--------------------------------------------------------------------------------

--------------------------------------------------------------------------------

--------------------------------------------------------------------------------

--------------------------------------------------------------------------------

--------------------------------------------------------------------------------

--------------------------------------------------------------------------------

----------------------------------------------------------------------------

>TERA05904

ATGGAATATCCACCACTGATTGAGTCCGAGATTGAGGAAAAGTTTATCCGTGGCTCAGGACCTGGTGGACAGAAGATCAA

CAAAACCAGTAACTGTGTCATGCTGAAACACTTGCCAACTGGAATTGTTGTCAAGTGTCAAGAAAGTAGATCTCTGGAGA

CAAATCGTGCCAGAGCCCGCAGACATCTACAAGAGAGGCTAGACTGGTACTACAATGGAGAACAGAGTGTATTGGCATAA

--------------------------------------------------------------------------------

--------------------------------------------------------------------------------

--------------------------------------------------------------------------------

--------------------------------------------------------------------------------

--------------------------------------------------------------------------------

--------------------------------------------------------------------------------

--------------------------------------------------------------------------------

--------------------------------------------------------------------------------

--------------------------------------------------------------------------------

--------------------------------------------------------------------------------

--------------------------------------------------------------------------------

--------------------------------------------------------------------------------

--------------------------------------------------------------------------------

----------------------------------------------------------------------------

>TERA05943

ATGGACTGTGAAGTCAACAATCCCTGCACTCCAGAGAACATCGAAGAAGGCAACTTCTACTTCCCACATCACGATCCCAC

CAAATTCGTCCAGTGTGACGAACATGGACAGTGCTTTGTAATGCCATGTGCTCCGGGAACAGTCTGGGATCCAGACGCCA

ACACCTGCAATCATGCACCATAA---------------------------------------------------------

--------------------------------------------------------------------------------

--------------------------------------------------------------------------------

--------------------------------------------------------------------------------

--------------------------------------------------------------------------------

--------------------------------------------------------------------------------

--------------------------------------------------------------------------------

--------------------------------------------------------------------------------

--------------------------------------------------------------------------------

--------------------------------------------------------------------------------

--------------------------------------------------------------------------------

--------------------------------------------------------------------------------

--------------------------------------------------------------------------------

--------------------------------------------------------------------------------

----------------------------------------------------------------------------

>TERA06017

ATGGCTTTGCTCAGTAAAATACTTCGTGTTGATCATGCTGGGGAGCTAGGAGCTGTCAGAATATATGAGGGTCAATTAGC

TGTACTTGGACGTACAAAGGATGGACCACTGCTTCAAGAAATGAAAGGCCAGGAAGAACATCATTTGGCAACATTTAGTG

AGCTTCTTCCGAAAAATCGGGTCAGACCGACGGCCCTGACACCTATATGGAATGTGGCTGGCTTTGCACTAGGTGCAGGT

ACTGCACTCTTAGGCAGGGAGACTGCCATGGCTTGTACAGTTGCTATAGAAACAGCCATTGGAGAACATTATGACAGTCA

GATCCGAGAATTGATGGCCGATGGTGCAGATGGTTTAGAAAAACACAAAGAATTAATTGAGACCATCAGAAAGTTTCGGG

ATGATGAGCTTGGGCATTTAGAGGCGGGATTGGAAAATGATGCAGAAAAGGCCCCTTTTTATGATGCACTCAGCAAAGCA

ATACAGTTTGGGTGTAAGGGTGCCATATGGCTGTCTGAATAA--------------------------------------

--------------------------------------------------------------------------------

--------------------------------------------------------------------------------

--------------------------------------------------------------------------------

--------------------------------------------------------------------------------

--------------------------------------------------------------------------------

--------------------------------------------------------------------------------

--------------------------------------------------------------------------------

--------------------------------------------------------------------------------

--------------------------------------------------------------------------------

----------------------------------------------------------------------------

>TERA06135

ATGCCAGTCAACAGGAAGCGTATCTATGGACCAGAGGACCTGTGTGAGAGGAAGGATGAGGTTCATCACTGGAGAAATGA

GCGCATTGTTGCCGTTGCTCTTCTGCCTCTCATACCAACAGCCTTGGCTTATCCCAACATGCTGCTTGACATGACACTGT

GTTCTGTCATGGTTCTGCATTCACACTGGCGTCTGACTGGTGTGTGCCAGGATTACATTCATGGTGAAGTACTGCCAACC

ATTGCCAAGTACACAGTGCTCTTTTTCTCCATTGCTGCATTTGGCAGTCTCTGTTACTTCAACTACACTGACATCGGCTT

TGCCAAAGCTGTCAGACTCATCTAA-------------------------------------------------------

--------------------------------------------------------------------------------

--------------------------------------------------------------------------------

--------------------------------------------------------------------------------

--------------------------------------------------------------------------------

--------------------------------------------------------------------------------

--------------------------------------------------------------------------------

--------------------------------------------------------------------------------

--------------------------------------------------------------------------------

--------------------------------------------------------------------------------

--------------------------------------------------------------------------------

--------------------------------------------------------------------------------

----------------------------------------------------------------------------

>TERA06209

ATGCGTCTTGATAGGAAGATAGAGTTTCCAATGCCCGACAGGAGACAGAAGCGTCTGATCTTTGCCACGATCACCAACAA

AATGAACCTCAGTGAGGATGTAGATCTGGATGACTATGTTGCCCGTCCAGACAAGATCAGTGGTGCCGACATCAACGCCA

TCTGCCAGGAGGCTGGCATGCAGGCTGTCAGAGAGAACCGCTATGTTGTACTACCTAAAGACTTCGAGAAAGGCTACAAG

AATAATATCAAGAAGGATGAAATGGAGCACGAGTTCTATAAATAA-----------------------------------

--------------------------------------------------------------------------------

--------------------------------------------------------------------------------

--------------------------------------------------------------------------------

--------------------------------------------------------------------------------

--------------------------------------------------------------------------------

--------------------------------------------------------------------------------

--------------------------------------------------------------------------------

--------------------------------------------------------------------------------

--------------------------------------------------------------------------------

--------------------------------------------------------------------------------

--------------------------------------------------------------------------------

--------------------------------------------------------------------------------

----------------------------------------------------------------------------

>TERA06214

ATGTGTGACTGGTTGTTAACAACACGCACAGAAGTCTGGGAACAGGAAAAGAGTAACAGTGGTGACTTCTCTGTGACAAC

TGTTTCACAGAAGGAGCTTGCTGCATTCCACCAGGATCTCGGCAGCCTGAGAAAGGTCTGTCAGCACCTGAAGGCTGCTC

TGCCCAGGGTCTTCCTACATGAGGCAACAAGTCGAGCTATGGCTGGGGCCAGTCCAGCTAAGACTAGACAGCTTCTGCAC

AAAAGCCTCAGACGTAGACATATTGGAAAAAATGTAGACAGCTCCGAGAGAGATTTTGATGATATCACAGAGATTGAGAA

CATTGGACAGCGTGAGCATGCTACAGCTCTGCTGATGGCTTGCAGACATCTGCCAGCTCAGCTTACATCACCTGGTCAGC

GAGCCACCATGTTAACAGAAGCAGCCAAGACATATGAGAAGCTTGGTGATAAGAAGGGTGTTCAGGAGTGCAGGAACCTC

TTGATAAAGCACAGCAACACAACATCATGTGTGACAACACGCATGGCTTAA-----------------------------

--------------------------------------------------------------------------------

--------------------------------------------------------------------------------

--------------------------------------------------------------------------------

--------------------------------------------------------------------------------

--------------------------------------------------------------------------------

--------------------------------------------------------------------------------

--------------------------------------------------------------------------------

--------------------------------------------------------------------------------

--------------------------------------------------------------------------------

----------------------------------------------------------------------------

>TERA06269

ATGAACATGTCGAGTGATCCAGAAAGATTCGATGGCCTTCTCATGTCAATGGCCCAGCAGTGTGAAGAGGCTGGAATACA

CGAGATGCTTGATTATATATTTGGATTCCTGGCCAGAAAAACGGATTTCTATACTGGAGGTGGAAAAGATGCTGCTGAAA

AGATAATAAAAGAGAAATTCGTGAAGTATCAGCAGGTTTAA---------------------------------------

--------------------------------------------------------------------------------

--------------------------------------------------------------------------------

--------------------------------------------------------------------------------

--------------------------------------------------------------------------------

--------------------------------------------------------------------------------

--------------------------------------------------------------------------------

--------------------------------------------------------------------------------

--------------------------------------------------------------------------------

--------------------------------------------------------------------------------

--------------------------------------------------------------------------------

--------------------------------------------------------------------------------

--------------------------------------------------------------------------------

--------------------------------------------------------------------------------

----------------------------------------------------------------------------

>TERA06421

ATGGAAGAAGCTATAGATTATGTCAAACTAGGAAAGGAGTTGGAAGACCAAGAAATTGAGTCTCCAGGTGGCGTAGCAAC

AGCCCAAGTGTATGGTCAGCTTCTAGCCATTTACCTGCTGCAGAATGACATGCCAAATGCAAAGTTTCTCTGGAAGAGGA

TACCATCCTCTGTGAAAAATGCAAATCCTGAACTTGCTCAGGTTTGGACTGTGGGACAAAAGATGTGGAACAGGGACTTC

CCTGGAATATATGAAGCATTAAATAATGAATGGCCAAAGCACCTGAAGCCTATTATGGATGCTGTCTTAGAACACACACG

AGAACGAGGATTCAATCTTGTTGGTCAAGCATACTCATCTTAA-------------------------------------

--------------------------------------------------------------------------------

--------------------------------------------------------------------------------

--------------------------------------------------------------------------------

--------------------------------------------------------------------------------

--------------------------------------------------------------------------------

--------------------------------------------------------------------------------

--------------------------------------------------------------------------------

--------------------------------------------------------------------------------

--------------------------------------------------------------------------------

--------------------------------------------------------------------------------

--------------------------------------------------------------------------------

----------------------------------------------------------------------------

>TERA06677

ATGGGTCATGACCTTTACTCCGGAAAGTTCCAGGCCCATTCAATGCGCGTGCTCGGTGGCCTAAATATGGTCTTCTCCGT

AATGGACGATGAGGCTGTACTGCAAAGCGTCCTGGGTCACCTTCGGGATCAGCACAAAGAGAGGGCAATTCCCAGGAACT

ACTAA---------------------------------------------------------------------------

--------------------------------------------------------------------------------

--------------------------------------------------------------------------------

--------------------------------------------------------------------------------

--------------------------------------------------------------------------------

--------------------------------------------------------------------------------

--------------------------------------------------------------------------------

--------------------------------------------------------------------------------

--------------------------------------------------------------------------------

--------------------------------------------------------------------------------

--------------------------------------------------------------------------------

--------------------------------------------------------------------------------

--------------------------------------------------------------------------------

--------------------------------------------------------------------------------

----------------------------------------------------------------------------

>TERA06743

ATGGCTCTGTCTTGTGATGACGTGAAGAGCCACAAGGAATGGGCCAAGGATGTTCTGGCTTATGCTGGTTCCAATGATGA

GTTTCCGTACCCCATCGTGGATGACTCCAGTCGCAAGCTGGCTACAAGCCTTGGAATGATCGATCCAGATGAAAAAACGC

CCTCTGGTGAACCACTGACGTGCAGAGCTGTGTTTCTCATTGGCCCAGACAAGAAGCTGAAGGCTTCAATCTTGTATCCA

GCAACAACTGGAAGGAACTTTGATGAAATCCTTCGGTTGATCGACTCGCTGCAGTTGACTGCTGTGAAGAAAGTGGCGAC

ACCTGCTGACTGGAAGGCGGCAGAATAA----------------------------------------------------

--------------------------------------------------------------------------------

--------------------------------------------------------------------------------

--------------------------------------------------------------------------------

--------------------------------------------------------------------------------

--------------------------------------------------------------------------------

--------------------------------------------------------------------------------

--------------------------------------------------------------------------------

--------------------------------------------------------------------------------

--------------------------------------------------------------------------------

--------------------------------------------------------------------------------

--------------------------------------------------------------------------------

----------------------------------------------------------------------------

>TERA06951

ATGGTCCAGACCGGCTTCGGTCGAACTGGCGACCATTACTGGGGCTTCGAGGCGCACGGCGTCCAGCCGGACATCGTCAC

CATGGCCAAAGGCATCGGCAACGGCTTTCCGTTGGCAGCTGTCGTCACTCGGCCGGAGATAGCCGCCACGATGGGAGGCG

CCCTGCACTTCAATACCTACGGCGGCAATCCGATGTCGTGCGCCGTCGGCTCGTAA------------------------

--------------------------------------------------------------------------------

--------------------------------------------------------------------------------

--------------------------------------------------------------------------------

--------------------------------------------------------------------------------

--------------------------------------------------------------------------------

--------------------------------------------------------------------------------

--------------------------------------------------------------------------------

--------------------------------------------------------------------------------

--------------------------------------------------------------------------------

--------------------------------------------------------------------------------

--------------------------------------------------------------------------------

--------------------------------------------------------------------------------

--------------------------------------------------------------------------------

----------------------------------------------------------------------------

>TERA07146

ATGGAAATCAGATTCTTCTTTCCTGATAGCATAGTAGAACCAATTCCCATTGGACAAGCAGCTCGTGATTACCTGGCCAA

GACTGTAAATCCAACCCTGATGAAAGGACTTACTGAACTATGCAAAAAGAAGCCAGAAGACCCTGTGATTTGGTTGGCTG

ACTGGTTGCTGAGCAACAATCCAAACAAACCCTAA---------------------------------------------

--------------------------------------------------------------------------------

--------------------------------------------------------------------------------

--------------------------------------------------------------------------------

--------------------------------------------------------------------------------

--------------------------------------------------------------------------------

--------------------------------------------------------------------------------

--------------------------------------------------------------------------------

--------------------------------------------------------------------------------

--------------------------------------------------------------------------------

--------------------------------------------------------------------------------

--------------------------------------------------------------------------------

--------------------------------------------------------------------------------

--------------------------------------------------------------------------------

----------------------------------------------------------------------------

>TERA07154

ATGAGCATGTCAAACGATAAAATTCCACCACCAGTTATTTGGGCTGAGAGGACAGACAAGCTTTATGTAACAATATGTGT

TGAAGATTGTAAAGATCCAGTAATAAAGTTTGATTCAAACTCACTATACTTCAGTGGTAAGGGTGGCCCAGAAAACAAAG

AGTATGAAGTCAAAATGGAATTCTTCAAAGAAATTGACCCAGAGGCATCGCACTATGCTGTGTTAGCTCGGCACATACCT

ATGGTGATAAAGAAGAAGGAAGAGGGACCTTATTGGTCAAGGTTGTTGAAGGAAAAGGGCAAGGTTCACTGGTTGAAAAC

AGACTTTAACAAGTGGAAGGATGAAGATGATTCTGATTTTGATGAAGCAGAGGATATGCAGTTTGAAAATATGATGAAAA

AGATGGGTAACTTTAACACAGAGGACACAGAAGGCCCACCTTTGGATGATGAA---GATAGTGATGATGAAGATTTGCCG

GACTTGTAA-----------------------------------------------------------------------

--------------------------------------------------------------------------------

--------------------------------------------------------------------------------

--------------------------------------------------------------------------------

--------------------------------------------------------------------------------

--------------------------------------------------------------------------------

--------------------------------------------------------------------------------

--------------------------------------------------------------------------------

--------------------------------------------------------------------------------

--------------------------------------------------------------------------------

----------------------------------------------------------------------------

>TERA07596

ATGAACAGAGGTGTACGTCTTACCGAGCTGTTGAAACAGGGCCAGTATGTACCGATGGCCATTGAGGAACAAGTTGCTGT

CATCTATGCTGGTGTCCGTGGTTACTTGGACAAAGTCGATCCTGCTCGCATAACAACCTTTGAGAAGGAGTTCTTGGCAC

ACATGCTGAGTTCACAGAAGGACCTACTAGAAACAATTCGTAAGGATGGGCAGATAAAACCAGAGACTGATGCCAAGCTG

AAGGAAGTTGTTGTGAGCTTCATTGCTGGCTTCAAATAA-----------------------------------------

--------------------------------------------------------------------------------

--------------------------------------------------------------------------------

--------------------------------------------------------------------------------

--------------------------------------------------------------------------------

--------------------------------------------------------------------------------

--------------------------------------------------------------------------------

--------------------------------------------------------------------------------

--------------------------------------------------------------------------------

--------------------------------------------------------------------------------

--------------------------------------------------------------------------------

--------------------------------------------------------------------------------

--------------------------------------------------------------------------------

----------------------------------------------------------------------------

>TERA07707

ATGGTGGCTGGGAAGGTGACCTTGAAGCATGTTTATGAAATTGCAAAGATAAAAAGTCAAGATCCAGTTTGGTTTGGTGT

ACCAATGGAGAACATCTGCAAGTCAATCATTGGAACTGCCCACTCAATAGGCATTGAGGTGATCAAACGGGATCTATCAG

CTAAAGAATATGGAGCATTTCTTGCTGAAAGACGCAGGATCGTTGCTGAACAAGAAAGAGAATTACAGGAAAAGAAGGCT

GCAAAATTGTTGAGAATTAGTTAA--------------------------------------------------------

--------------------------------------------------------------------------------

--------------------------------------------------------------------------------

--------------------------------------------------------------------------------

--------------------------------------------------------------------------------

--------------------------------------------------------------------------------

--------------------------------------------------------------------------------

--------------------------------------------------------------------------------

--------------------------------------------------------------------------------

--------------------------------------------------------------------------------

--------------------------------------------------------------------------------

--------------------------------------------------------------------------------

--------------------------------------------------------------------------------

----------------------------------------------------------------------------

>TERA07846

ATGGGCAAATCAAAAGAAGAGGCACAACTGGAAAAACGGCAGGAGTTGGAGAGAAGACTAGAGGATGTCAAAGGTCAACT

TGGTACACCACAACCTAAGAAGACACCGAAGAAAGGTGAAAAGTCACATGGTGAAGTGGCTGGTGGTAGTCGACTCAGTG

CCAGCAGTAGTAGCTCGACAGACACAGACAGCAGCTCCGACAGCAGTTCATCTAGTTCATCTGATACCAGCGAGTCAGAG

TCAGGTTAA-----------------------------------------------------------------------

--------------------------------------------------------------------------------

--------------------------------------------------------------------------------

--------------------------------------------------------------------------------

--------------------------------------------------------------------------------

--------------------------------------------------------------------------------

--------------------------------------------------------------------------------

--------------------------------------------------------------------------------

--------------------------------------------------------------------------------

--------------------------------------------------------------------------------

--------------------------------------------------------------------------------

--------------------------------------------------------------------------------

--------------------------------------------------------------------------------

----------------------------------------------------------------------------

>TERA08080

ATGAAAATGGCGGCTACTGCCATGTATTTGGAGCACTATTTGGACAGTCTGGAAGGACTGCCCATGGAATTACAGAGGAA

TTTCAATCTCATGAGGGACCTGGATACACGCTCTCAAGAAATTCTTAAAGAAATTGACAACAAAGCTGACAAGTACATGA

AGGAAGTTAGGAATTATCCACCAGCCAAGAGATCTGACTACATAGCAACCATTCAGAAGCTGTTCAATAAAAGTAAAGAA

TATGGAGATGATAAAGTTCAATTAGCCATGCAGACATATGAAATGGTGGACAAACACATCAGGAAGCTTGATTCAGACCT

GGCTCGTTTTGAAGCTGAACTTAAGGATAAAAGCAGATGTTCCCCATAA-------------------------------

--------------------------------------------------------------------------------

--------------------------------------------------------------------------------

--------------------------------------------------------------------------------

--------------------------------------------------------------------------------

--------------------------------------------------------------------------------

--------------------------------------------------------------------------------

--------------------------------------------------------------------------------

--------------------------------------------------------------------------------

--------------------------------------------------------------------------------

--------------------------------------------------------------------------------

--------------------------------------------------------------------------------

----------------------------------------------------------------------------

>TERA08108

ATGCGGCCTCGTATGAGTGCAGCCAGAGAAAACATGATAAAGAAAGCCTTCGCCAAGTTGGACGTGGACGGTAGCGGAGA

GGTGACGGTGGAAGACCTAGAGAAGATCTACAACGTCAAAAATCACCCCAAATACCAGTCCGGAGAGATGACAAGAAAAC

AAATATTACAGGAATTTCTCAACAACTTCCAGGCTGGTCCCAAGGACGAAAAGGTCACATATGAAGAGTTTTATAACTAC

TACTGTGGTGTCAGTGCCTCCATAGATCACGATACATACTTCGATCTCATGATGAGGAACAGCTAA--------------

--------------------------------------------------------------------------------

--------------------------------------------------------------------------------

--------------------------------------------------------------------------------

--------------------------------------------------------------------------------

--------------------------------------------------------------------------------

--------------------------------------------------------------------------------

--------------------------------------------------------------------------------

--------------------------------------------------------------------------------

--------------------------------------------------------------------------------

--------------------------------------------------------------------------------

--------------------------------------------------------------------------------

--------------------------------------------------------------------------------

----------------------------------------------------------------------------

>TERA08283

ATGGAAGCTGGTGGAAAACACAAACAAGGACCAAACCTTCATGGCTTCATTGGTCGTAAAACAGGACAAGCTCCTGGCTA

TACCTTTACAGAGGCAAACCTAAAGAAAGGTATTATTTGGGACAAGGAAACCCTTGACATCTACTTAGAGAACCCAAAGA

AGTACATACCCGGAACAAAGATGATCTTTCCAGGTCTTAAAAAGAAGTAA------------------------------

--------------------------------------------------------------------------------

--------------------------------------------------------------------------------

--------------------------------------------------------------------------------

--------------------------------------------------------------------------------

--------------------------------------------------------------------------------

--------------------------------------------------------------------------------

--------------------------------------------------------------------------------

--------------------------------------------------------------------------------

--------------------------------------------------------------------------------

--------------------------------------------------------------------------------

--------------------------------------------------------------------------------

--------------------------------------------------------------------------------

--------------------------------------------------------------------------------

----------------------------------------------------------------------------

>TERA08382

ATGGCTGATTACTGGAAATCCCAGCCACGAAAATTCTGTGAATTTTGCAAATGCTGGATAGCTGACAACAAACCCAGTCG

GGAATTCCATGAAAAGGGCAAGAAGCATCAAGAGAATGTACAGAAAAAAATTGATGAGGTGATGAAAAAAGGAAGGCAGG

CAGCTGCTGCAAAAGCTGAAATGAGTGAGACACTCAAAGAAATGGAAAAGGCAGCATTGAAGGCCTTGAAGAATGATCTA

CAAAGAGATCCTAGTTTGGCAGCACAGTATGGTGTGAAAATAAAACAGAAAGAGTAA-----------------------

--------------------------------------------------------------------------------

--------------------------------------------------------------------------------

--------------------------------------------------------------------------------

--------------------------------------------------------------------------------

--------------------------------------------------------------------------------

--------------------------------------------------------------------------------

--------------------------------------------------------------------------------

--------------------------------------------------------------------------------

--------------------------------------------------------------------------------

--------------------------------------------------------------------------------

--------------------------------------------------------------------------------

--------------------------------------------------------------------------------

----------------------------------------------------------------------------

>TERA08396

ATGGCCGACACAGCAGCCCCAGCCAAGCCGAAGAAGGCCAGCAAACCGCGGAAGCCGGCCAACCACCCGAAGTACTCGGA

GATGATCAAGGCGGCGCTGGCCAACCTGAAGGAACGCGGCGGCTCCTCGCGACAGGCCATCCTCAAGTACATCCTCAAAC

ACTACAACGTCGGCAACGACGAGAAGTAA---------------------------------------------------

--------------------------------------------------------------------------------

--------------------------------------------------------------------------------

--------------------------------------------------------------------------------

--------------------------------------------------------------------------------

--------------------------------------------------------------------------------

--------------------------------------------------------------------------------

--------------------------------------------------------------------------------

--------------------------------------------------------------------------------

--------------------------------------------------------------------------------

--------------------------------------------------------------------------------

--------------------------------------------------------------------------------

--------------------------------------------------------------------------------

--------------------------------------------------------------------------------

----------------------------------------------------------------------------

>TERA08446

ATGTTCAAGATGCTGCGTATCATAGAGAAGCCTTCGCCCCGAAAGTTGGTGATGTCACCGGGGCAAAAGGTCAGCCGGAA

GTACAAGATAGCTCAACAGAAGAGAATGATCAAACGGAAGGAATACAAGAAACTGAAAGCCATCGTGCCGTCGGTGTCCA

AGAAGTGTAAAGTACCAAAGATAACCGTCATAGAAGAGGCCATTAAGTACATCGACGAGCTGCACTAA------------

--------------------------------------------------------------------------------

--------------------------------------------------------------------------------

--------------------------------------------------------------------------------

--------------------------------------------------------------------------------

--------------------------------------------------------------------------------

--------------------------------------------------------------------------------

--------------------------------------------------------------------------------

--------------------------------------------------------------------------------

--------------------------------------------------------------------------------

--------------------------------------------------------------------------------

--------------------------------------------------------------------------------

--------------------------------------------------------------------------------

--------------------------------------------------------------------------------

----------------------------------------------------------------------------

>TERA08453

ATGGGAGATGGTAATACATTCCCGAAGACTGGTCAGACTGTGACCGTGCACTATGTAGGAACCCTACCTGATGGCTCAAA

ATTCGACTCCTCACGGGACCGTGGCAAACCATTTCAATTCAAAATAGGAGAGGGCCAGGTCATTAAAGGTTGGGATGAAG

GTGTTGCCCAGATGAGTGTAGGTCAAGTTGCCAAGCTGACCTGTTCTCCAGATTATGCATATGGAGCCAAGGGTTATCCC

GGAGTTATCCCACCTAATGCAACACTGATATTTGAAGTGTAA--------------------------------------

--------------------------------------------------------------------------------

--------------------------------------------------------------------------------

--------------------------------------------------------------------------------

--------------------------------------------------------------------------------

--------------------------------------------------------------------------------

--------------------------------------------------------------------------------

--------------------------------------------------------------------------------

--------------------------------------------------------------------------------

--------------------------------------------------------------------------------

--------------------------------------------------------------------------------

--------------------------------------------------------------------------------

--------------------------------------------------------------------------------

----------------------------------------------------------------------------

>TERA08485

ATGCCAATACTATTTGCCACTGTATCACGAGGTAACACCATTTTAGCTAAATATGCCAGCTGCTCTGGTAACTTTTCTGA

GGTTGTTGAACAAGTGCTGGCAAAGATATCACCAGAGAATGCTAAGCTGACATACTCACATGGCAATTATTTATTCCACT

ACATATCAGAGAACCGAATAATATACTTGGCTATAACAGATGATGATTTTGAAAGATCCAAGGCATTTGCCTTTCTTAAT

GATATCAAGAGAAGATTTCAAATACAGTATGGAGATCGTGCACAAACAGCGCTTCCATTTTCCATGAATAGTGAGTTCAG

CAGAACTCTAAATGCTCAGATGCAACATTATAGCAGTGACATGGCAGGTCCAGATAAACTCACAGAAGTTCAGGAACAAG

CAGATGAACTAAAGGGAATTTTAGTTCGAAATATTGACAGTATAGCATCCAGGGGAGAACGTTAA---------------

--------------------------------------------------------------------------------

--------------------------------------------------------------------------------

--------------------------------------------------------------------------------

--------------------------------------------------------------------------------

--------------------------------------------------------------------------------

--------------------------------------------------------------------------------

--------------------------------------------------------------------------------

--------------------------------------------------------------------------------

--------------------------------------------------------------------------------

--------------------------------------------------------------------------------

----------------------------------------------------------------------------

>TERA08527

ATGGAATGGATGAATTATCAAAAGGGTCACTGTAGTGCCTACGAGCTAGATTTTGCACGTTGTGCAAGTCATGTAGGTGC

AGTCCGGGCAATAAAAGACTGCAGGAAGTTCATGGAAGACATGCAGGAGTGTGCACGCCACACAAAATCGCTGGAGAGGT

ACAAGATCATGCAAGCTGAAAGGAAGAAGCAGGGAAGACCTTACCTTCCAACACCTCCAAAAGACAGTGTATAA------

--------------------------------------------------------------------------------

--------------------------------------------------------------------------------

--------------------------------------------------------------------------------

--------------------------------------------------------------------------------

--------------------------------------------------------------------------------

--------------------------------------------------------------------------------

--------------------------------------------------------------------------------

--------------------------------------------------------------------------------

--------------------------------------------------------------------------------

--------------------------------------------------------------------------------

--------------------------------------------------------------------------------

--------------------------------------------------------------------------------

--------------------------------------------------------------------------------

----------------------------------------------------------------------------

>TERA08550

ATGTATTACGACATAGCCACTGACTTCTTCGAGTACGGCTGGGGCGAAGGATTTCACTTTGCCACGATCAACAAGGGCGA

GTCTAGGGAGCACGCCTTCGCCAGACACGAATATAAGTTGGCCCTGAAGCTCGGGTTGAAGAAGGGGGATACCGTATTGG

ACATAGGCTGTGGTGTCGGCGGTCCAGGCAGACATATTGCAGCATTTACCGAAGCAAAGGTTATTGGTTTGAACATCAAT

GAATACCAAATAAAAAGGGCGAGATCTCTGACGAAAAAAGTCGGCTTGGAGCACTTGTGTGATTATGTCAAGGGAGACTT

CTGTTAA-------------------------------------------------------------------------

--------------------------------------------------------------------------------

--------------------------------------------------------------------------------

--------------------------------------------------------------------------------

--------------------------------------------------------------------------------

--------------------------------------------------------------------------------

--------------------------------------------------------------------------------

--------------------------------------------------------------------------------

--------------------------------------------------------------------------------

--------------------------------------------------------------------------------

--------------------------------------------------------------------------------

--------------------------------------------------------------------------------

----------------------------------------------------------------------------

>TERA08632

ATGAAAGTGAAGACACTGACAGGTAAAGAGATTGAAATAGATATTGAACCCACAGATAAGGTTGAACGTATAAAGGAACG

CGTAGAAGAAAAAGAAGGCATCCCACCTCCTCAGCAAAGGTTAATCTTTAGTGGAAAACAAATGAATGATGAAAAGACAG

CAGCAGATTACAAAGTTACTGGTGGTTCCGTGTTACACTTGTAA------------------------------------
[truncated: 539,511 more chars]
